# Bioinformatic Analysis and Post-Translational Modification Crosstalk Prediction of Lysine Acetylation

**DOI:** 10.1371/journal.pone.0028228

**Published:** 2011-12-02

**Authors:** Zhike Lu, Zhongyi Cheng, Yingming Zhao, Samuel L. Volchenboum

**Affiliations:** 1 Ben May Department for Cancer Research, University of Chicago, Chicago, Illinois, United States of America; 2 Department of Pediatrics, University of Chicago, Chicago, Illinois, United States of America; 3 Computation Institute, University of Chicago, Chicago, Illinois, United States of America; University College Dublin, Ireland

## Abstract

Recent proteomics studies suggest high abundance and a much wider role for lysine acetylation (K-Ac) in cellular functions. Nevertheless, cross influence between K-Ac and other post-translational modifications (PTMs) has not been carefully examined. Here, we used a variety of bioinformatics tools to analyze several available K-Ac datasets. Using gene ontology databases, we demonstrate that K-Ac sites are found in all cellular compartments. KEGG analysis indicates that the K-Ac sites are found on proteins responsible for a diverse and wide array of vital cellular functions. Domain structure prediction shows that K-Ac sites are found throughout a wide variety of protein domains, including those in heat shock proteins and those involved in cell cycle functions and DNA repair. Secondary structure prediction proves that K-Ac sites are preferentially found in ordered structures such as alpha helices and beta sheets. Finally, by mutating K-Ac sites *in silico* and predicting the effect on nearby phosphorylation sites, we demonstrate that the majority of lysine acetylation sites have the potential to impact protein phosphorylation, methylation, and ubiquitination status. Our work validates earlier smaller-scale studies on the acetylome and demonstrates the importance of PTM crosstalk for regulation of cellular function.

## Introduction

Protein post-translational modification has emerged as a major contributor to variation, localization and control of proteins. It has been suggested that the incongruity between the complexity of vertebrate organisms and the size of their encoded genomes is compensated by the large number of PTMs available [1]. Of the hundreds of PTMs identified, the most intensively studied is protein phosphorylation, in which protein kinases (PK) attach phosphate moieties to Ser, Thr, or Tyr residues. Protein phosphorylation appears to play an essential role in many diverse and critical cellular processes, and inhibitors of their function have emerged as important modes of therapy for malignant diseases.

Acetylation of histone proteins was discovered almost 50 years ago [2] and has long been associated with regulation of chromatin structure [3]. Recent proteomics studies identified almost 5000 acetylation sites across almost 2000 proteins [4], [5], [6]. These substrates of lysine acetylation are prevalent not only in the nuclei, but also in other cellular compartments, such as the cytoplasm, mitochondria, and the plasma membrane [4], [5], [6]. Furthermore, this PTM is known to involve such diverse cellular processes such as signal transduction, cytoskeletal dynamics, and membrane stability, prompting the observation that acetylation is analogous to phosphorylation [7], [8].

In addition to acetylation, the lysine side chain has been found to be the target of numerous other modifications such as ubiquitination, sumoylation, methylation, neddylation, propionylation, and butylation [9], [10], [11], [12], [13]. Evidence suggests that acetylation and other PTMs form complex regulatory networks, and this “crosstalk” is the basis for a protein modification code [1], [14], [15]. Hunter describes both positive and negative crosstalk. In the former, a PTM signals the addition or removal of another PTM or creates conditions favorable for the binding of a protein that performs the second modification. Negative crosstalk can result from direct competition for modification of a single residue (e.g., among acetylation, butyrylation, methylation, and ubiquitination at the same lysine residue), while another mechanism is the masking of a binding site, preventing the action of a modifying protein. Multisite PTMs appear to act in sequential and/or combinatorial ways [16], [17] and seem to generate complex programs of regulation and signaling [18], [19]. For example, multiple enzymes appear to phosphorylate histone protein H3 at residue Ser10 [20], [21], [22], which appears to stimulate acetylation at Lys14 (positive crosstalk) [20]. Conversely, this phosphorylation blocks acetylation and methylation at Lys9 in the same protein (negative crosstalk) [23].

Recently, a genome-wide analysis of single nucleotide polymorphisms (SNPs) found that around 70% of SNPs that lead to a change in peptide sequence (non-synonymous SNPs, nsSNPs) potentially affect protein phosphorylation status and play an important role in cancer and other diseases [24]. Based on the abundance of potential acetylation sites and their apparent importance in a wide variety of cellular functions, the evidence of PTM crosstalk, and these recent findings relating genome-wide SNP changes to changes in phosphorylation status, we sought to better define the acetylome. Here we present the first comprehensive global analysis of potential acetylation sites, including an analysis of cross-species conservation, a survey of domain and secondary structure, gene ontology and pathway analysis, and a study of potential crosstalk between and among these and other PTMs. This comprehensive global survey of acetylation sites and their interaction with other PTMs will serve to provide valuable substrate for future study of protein modifications in health and disease.

## Results

### Data Integration

**Figure 1:**
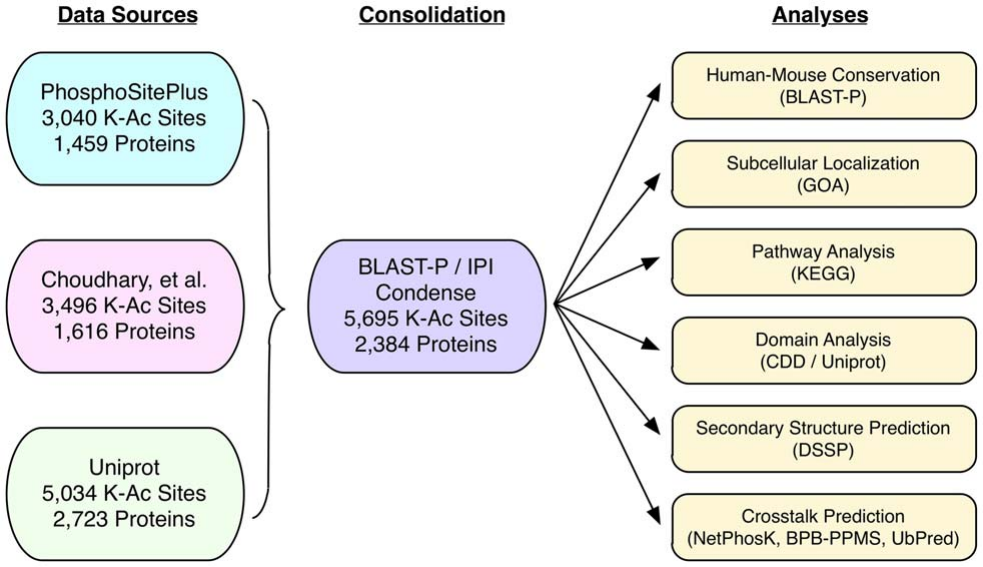
Data source and analyses. Lysine acetylation sites were collected from three sources; PhosphositePlus, Uniprot, and from the work of Choudhary, *et al*. BLAST-P was used to map each acetylation site to the corresponding IPI protein. The data were condensed to remove redundant sites. Subsequent analyses included conservation studies, subcellular localization, pathway analysis, domain analysis, secondary structure prediction, and crosstalk prediction. Details and methods are outlined in the text.

Several sources are available for lysine acetylation substrates, and we collected information from three sources with the largest datasets (Figure 1): (i) the PhosphoSitePlus database, [25]) (a manually curated database of PTMs), (ii) Uniprot [26], [27] (a comprehensive database of protein sequence and annotation data), and (iii) 3,600 lysine acetylation sites from 1,750 proteins, identified by mass spectrometry [5]. Because of inconsistencies and slight variations present in the exact location reported for the acetylated lysine residue, we used BLAST-P to map protein sequences to the International Protein Index (IPI) database (v. 3.69) [28]. Sites redundant across the datasets were condensed. In all, we identified 5,695 unique lysine acetylation sites across 2,834 IPI entries. The full list of acetylation sites and corresponding IPI numbers are listed in Table S1. We derived a list of phosphorylation sites in a similar manner, using three sources: PhosphoSitePlus, Uniprot, and Phospho.ELM, [29]. We standardized the phosphorylation position according to the IPI designation using BLAST-P. We identified 32,702 serine phosphorylation sites across 6,957 IPI entries, 9,293 threonine phosphorylation sites across 4,026 IPI entries, and 10,277 tyrosine phosphorylation sites across 4,656 IPI entries.

### Conservation of lysine acetylation sites between mouse and human

A high degree of interspecies conservation has been reported for lysine acetylation [30] as well as histone deacetylases [31]. We were interested in demonstrating the evolutionary conservation of our identified lysine acetylation sites between human and mouse genomes. We used BLAST-P to compare 87,130 human protein sequences to 56,737 mouse protein sequences downloaded from IPI. Overall, we found an amino acid conservation of 84.8%. Lysine residues were conserved at a rate of 87.0%. Of the 5,695 acetylated lysine residues in 2,384 proteins identified above, we were able to identify homologous sites in mouse for 5,290 lysine residues (92.9%, *p* = 1.78×10^−6^) over 2,256 proteins (94.6%, *p* = 7.70×10^−4^), demonstrating a very high degree of conservation for this PTM and implicating these as likely functional lysine acetylation sites in the mouse.

### Subcellular localization of K-Ac substrate proteins

**Figure 2:**
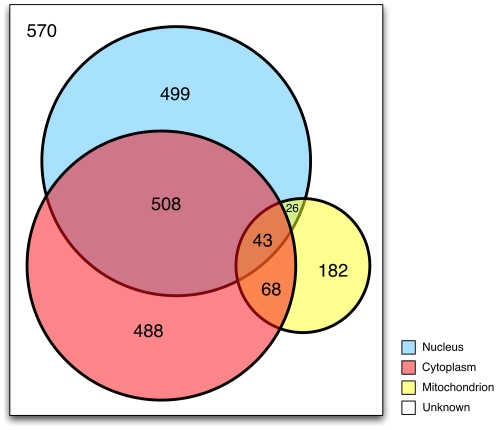
Subcellular localization of acetylated lysine residues. The gene ontology annotations for identified IPI genes were obtained from the GOA database. Of the 2384 lysine acetylation genes identified, 488 (20.4%) are exclusively nuclear, 182 (7.6%) are mitochondrial only, and 499 (20.9%) are only found in the cytoplasm. 570 proteins (23.9%) were not assigned to any compartment.

In order to further characterize lysine acetylation across the proteome, we investigated the cellular localizations for our identified acetylated lysine PTMs. The gene ontology annotations for identified IPI genes were obtained from the GOA database [32]. Of the 2,384 human proteins found with acetylated lysine residues, 499 (20.9%) localized solely to the nucleus, 488 (20.5%) were found only in the cytoplasm, and 182 (7.6%) were only in mitochondria (Figure 2). The remaining proteins were either in an unknown location (570, 23.9%) or in some combination of the above locales (645, 27.2%). Therefore, as many as 1,885 (79.0%) of identified proteins may be found outside of the nucleus, supporting the recent evidence that non-histone proteins are a major group of K-Ac targets [4], [5], [6].

### Biologic pathway analysis

**Figure 3:**
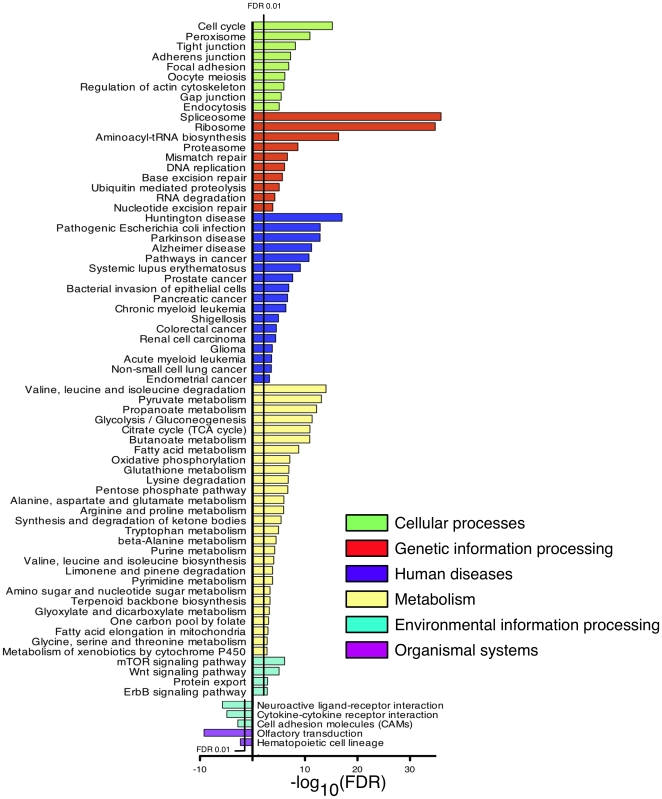
Biologic pathway analysis. Using the Kyoto Encyclopedia of Genes and Genomes (KEGG), we identified pathways enriched for the acetylated proteins identified. False discovery rate control was used to correct for multiple hypothesis testing, the KEGG pathways with a corrected *p*-value<0.01 were considered significant. A diverse array of cellular pathways and functions were identified including those involved in cellular processes (light green), genetic information processing (red), human diseases (indigo), metabolism (yellow).

We surveyed the Kyoto Encyclopedia of Genes and Genomes (KEGG) database [33] to determine which pathways were enriched for the proteins we identified above. Our data reveal that acetylation of lysine is a common PTM across a diverse array of cellular processes such as those involved in cell cycle and peroxisome metabolism (Figure 3). Additionally, enrichment of K-Ac substrates was observed for cellular processes involving cellular communication, cytoskeletal assembly and maintenance, as well as those important in maintaining localization. Several pathways important in genetic information processing were also enriched, including spliceosome, proteasome and ribosome assembly, tRNA synthesis as well as DNA repair and replication. A wide and diverse array of important metabolic pathways were found to be enriched, including those involving amino acid metabolism, glycolysis and gluconeogeneis, and fatty acid metabolism. Interestingly, among environmental information processing pathways, the mTOR signaling pathway was found to be enriched, which is highly connected with cellular energy metabolism. We also found significant enrichment of K-Ac proteins in both the monogenetic disease Huntington's chorea as well as complex, multifactorial disorders such as Parkinson's disease, Alzheimer's disease, and systemic lupus erythematosus (SLE). Finally, proteins with acetylated lysine were found enriched in several oncologic processes such as prostate and pancreatic cancers as well as chronic myelogenous leukemia (CML). Among the processes found to have underrepresentation of K-Ac proteins were cytokine-cytokine receptor interactions and neuroactive ligand-receptor interactions. The underrepresentation of these processes may be due in part from the fact that the proteins involved may not be expressed in the cell lines or tissues from which the data are derived. In addition, as with other global measures of gene and protein expression, results may be biased by the abundance of substrate. In all, lysine acetylation was found to be a common PTM in a wide variety of crucial cellular functions and in several important diseases and oncologic processes.

### Domain analysis

**Figure 4:**
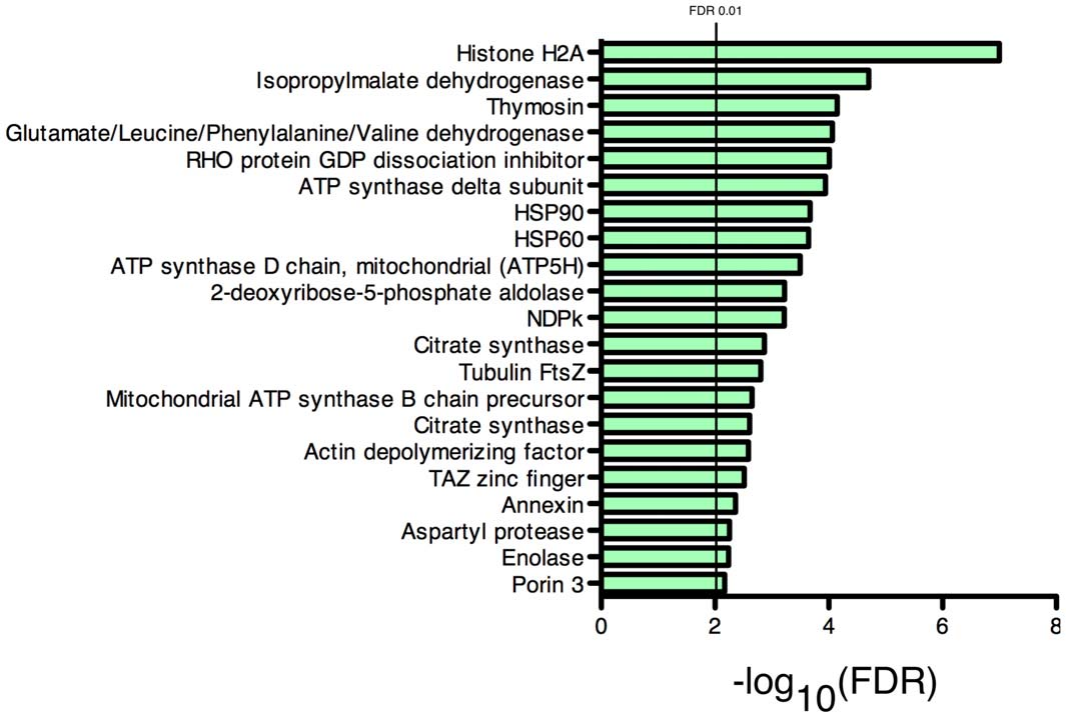
Domain analysis. Proteins with acetylated lysines were aligned to the distribution of domains in the NCBI Conserved Domain Database using PRS-BLAST. Correction for multiple hypothesis testing was carried out using standard false discovery rate control methods, and domains with a corrected *p*-value<0.01 were considered significant. Domains with more than the number expected K-Ac sites were considered “overrepresented.”

The domain structure surrounding acetylation sites may give important insight into the function, regulation and importance of K-Ac as a PTM. We investigated the functional domains containing K-Ac proteins. Domain information was obtained from the NCBI Conserved Domain Database (CDD [34], [35]), a collection containing domain information from NCBI-curated domains as well as domain models from other sources. Proteins containing K-Ac were aligned to the distribution of CDD domains using Reverse-PSI BLAST (RPS-BLAST) [28], and a two-tailed Fisher's exact test was used to test the distribution of K-Ac sites within each domain against all IPI proteins. In total, 737 domains were observed to contain K-Ac sites, and of these, 26 were over-represented for K-Ac sites (Figure 4, Table S2). False discovery rate calculations were performed to account for multiple hypothesis testing, and domains with an FDR<0.01 were considered significant. One of the domains found to be most enriched was pfm00183 (Hsp90). Heat shock protein 90 is a molecular chaperone essential for the regulation of many signaling proteins and is subjected to various PTMs, including phosphorylation [36] and acetylation [37], [38]. Not surprisingly, the cl0074 domain of the core histone proteins H2A/H2B/H3 and H4 was found to be enriched for K-Ac. Several domains enriched for K-Ac were found within metabolic enzymes, such as pfam00285 (citrate synthase), pfam00026 (aspartyl protease), and cl00445 (isocitrate dehydrogenase). Other enriched domains were found to be within the cytoskeletal protein actin (pfam00022)and the membrane protein annexin (cl02574).

### Secondary structure prediction

**Figure 5:**
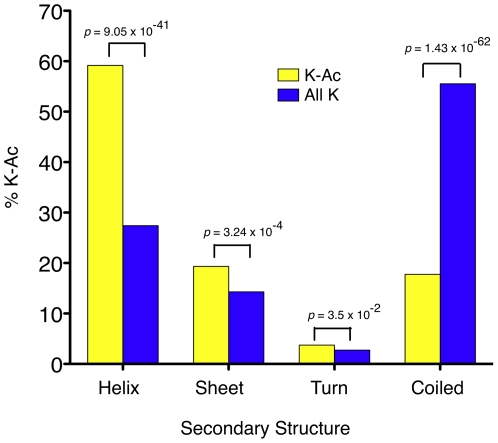
Secondary structure prediction. The secondary structure of acetylated proteins was derived from Uniprot and the Dictionary of Protein Secondary Structure (DSSP). There was significant enrichment of acetylated lysine sites in alpha helices and beta-sheets only.

To further characterize lysine acetylation, we surveyed secondary structure predictions using Uniprot and the Dictionary of Protein Secondary Structure (DSSP), a database of secondary structure assignment prediction based on primary sequence [39]. We found significant enrichment for K-Ac sites in alpha helices (*p* = 9.05×10^−41^), beta-sheets (*p*<3.24×10^−4^), and turns (*p*<3.50×10^−2^), while no enrichment was found in coiled-coils (Figure 5). Given the evolutionary conservation of K-Ac sites found and their importance in critical cellular functions, the high degree of enrichment of K-Ac sites in ordered secondary structure is not surprising.

### Crosstalk

We are keenly interested in understanding how acetylation of lysine residues can influence other PTMs, either at the same location or at a nearby site, facilitating PTM crosstalk [15]. Our data reveal that 1,165 (20.5%) of K-Ac sites are within ten residues of known phosphorylation sites and that 2,121 (37.2%) of K-Ac sites form acetylated lysine clusters with nearby K-Ac modifications (Table S3). These frequent associations suggest that PTM crosstalk is common and likely represents a codified crosstalk among and between these modifications.

**Figure 6:**
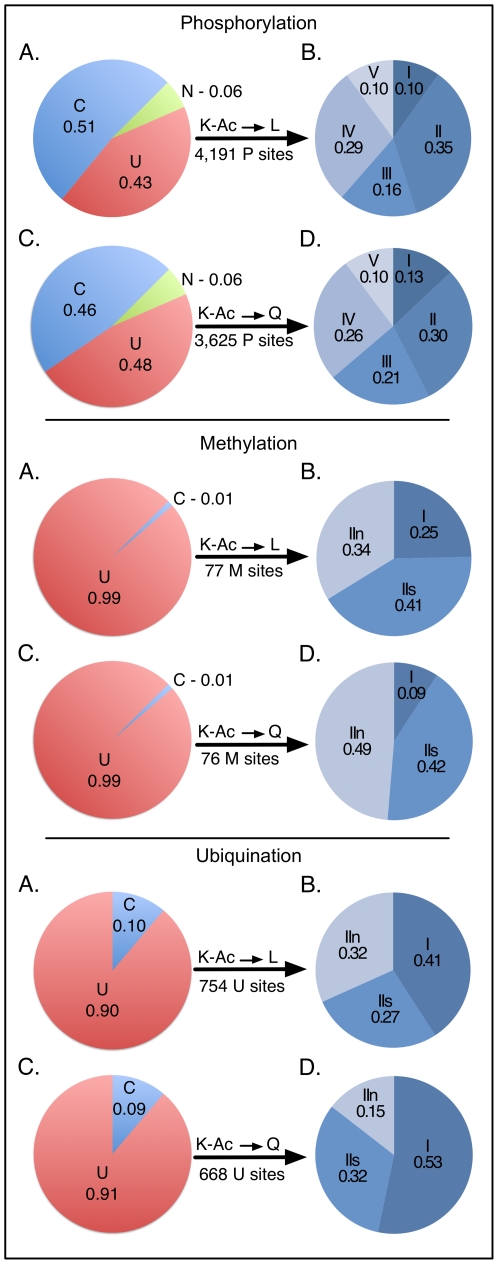
Crosstalk prediction. Top: (A) The influence of acetylated lysine on neighboring phosphorylation sites was predicted by substituting a leucine for a lysine at all predicted K-Ac sites and then predicting potential phosphorylation sites using NetPhosK 1.0. (B) In all, 51% of nearby phosphorylation sites were affected, and these were classified as Type I (gain of a phosphorylation site, 10%), Type II (loss of a phosphorylation site, 35%), Type III (retention of a phosphorylation site with gain of kinase binding site, 16%), Type IV (retention of a phosphorylation site with loss of kinase binding site, 29%), and Type V (loss of an endogenous kinase binding site with concomitant gain of a new one, 10%). This analysis was repeated by substituting glutamine for lysine (Panels C and D). This analysis was repeated for methylation using BPB-PPMS (middle) and ubiquitination using UbPred (bottom). For further details, see text.

Upon acetylation, the lysine side chain is changed as the acetyl group neutralizes the positive charge and influences the ability to form hydrogen bonds. Consequently, the nearby microenvironment is modified compared to acetylation-free lysine. To better model the impact of acetylation and its implications on PTM crosstalk, we chose to perturb the microenvironment *in silico* through substitution of lysine with glutamine (neutral side chain) or leucine (hydrophobic side chain). To predict the impact of these substitutions, we used NetPhosK 1.0, a software tool for modeling phosphorylation sites based on kinase-specific predictions [40], [41]. We compared the software-predicted phosphorylation sites and kinase binding sites near acetylated lysines to the predictions following lysine substitution with either glutamine or leucine. When a leucine is substituted for lysine at a K-Ac site, 4,191 neighboring phosphorylation sites in the vicinity of 2,926 (51.4%) sites are affected (Figure 6). We classified the affected phosphorylation sites into five categories: Types I and II: gain and loss of a phosphorylation site, respectively; Type III: retention of a phosphorylation site and gain of a new kinase binding site; Type IV: retention of a phosphorylation site but loss of kinase binding site; Type V: loss of an endogenous kinase binding site with simultaneous gain of a new one. When leucine is substituted for lysine, a significant number of sites lose their phosphorylation propensity (35%, Type II) or lose their kinase binding site (29%, Type IV). Conversely, the number of phosphorylation sites gained is small (10%, Type I), as is the number of kinase binding sites gained (16%, Type III). The phosphorylation site influenced is most often distributed within five residues of the acetylation site, with upstream sites being more affected than downstream ones (Figure S1). When glutamine was substituted, similar results were obtained (Figure 6, Figure S1). Of 5,695 K-Ac sites, 2,647 phosphorylation sites were directly affected (46.0%), as well as 3,625 neighboring sites.

Similarly, we extended our crosstalk analysis to examine the effects of lysine acetylation on ubiquitination and methylation (Table S4 and S5). To accomplish this, we used BPB-PPMS, an *in silico* tool for methylation prediction [42] and UbPred, a predictor of potential ubiquitination sites [43]. We compared the predicted methylation and ubiquination sites around acetylated lysines to the predictions after substitution with leucine or glutamine. We used the author-suggested cutoffs of 0.8 (methylation prediction) and 0.84 (ubiquitination prediction), and tallied the sites where methylation and ubiquitination were affected by changing the lysine to either leucine or glutamine. Because leucine and glutamine cannot be methylated or ubiquitinated, a substitution of lysine to leucine or glutamine eliminates the potential for self-modification. We classified the changes into three groups: Type I: gain of a methylation or ubiquitination on a nearby lysine; Type IIs (self): loss of methylation or ubiquitination on the lysine itself; Type IIn (neighbor): loss of methylation or ubiquitination on a nearby lysine. Seventy-six (1.3%) and 75 (1.3%) of the 5,695 acetylated lysines are predicted to have a direct or indirect effect on methylation sites when the lysine is substituted to leucine or glutamine respectively (Figure 6, middle). In the case of leucine substitution, 25% of the changes are predicted to be the gain of a surrounding methylation site (Type I), 41% are the loss of a methylation site on the lysine itself (Type IIs), and 34% are the loss of a nearby methylation site (Type IIn). Likewise, glutamine substitution results a change of 75 methylation sites, of which 9.2% are the gain of a surrounding site (Type I), 42% the direct loss of a site on the lysine itself (Type IIs), and 49% loss of a nearby methylation site (Type IIn). The methylation site affected is most often within five residues of the lysine (Figure S2). Comparatively, the predicted crosstalk between lysine acetylation and ubiquitination is much greater (Figure 6, bottom). In all, 552 (9.7%) K-Ac sites and 517 (9.1%) K-Ac sites show direct or indirect crosstalk with ubiquitination, respectively. For leucine substitution, 41% of the changes are the gain of a nearby ubiquitination site (Type 1), while the substitution results in the loss of a ubiquitination site on the lysine itself 27% of the time (Type IIs) and on a nearby lysine 32% of the time (Type IIn). Substitution by glutamine yields similar results as outlined in Figure 6. The affected ubiquitination site is most often within ten residues of the lysine, but can be as far away as 40 residues (Figure S3). These results implicate the acetyl group as having a drastic effect on the lysine microenvironment, leading the way for crosstalk between lysine acetylation and nearby phosphorylation methylation, or ubiquitination status.

## Discussion

Our early proteomics studies of lysine acetylation indicate that K-Ac is a highly abundant PTM that is enriched in mitochondria and in metabolic enzymes [4]. Similar studies in *E. coli* suggest an evolutionarily conserved role for K-Ac in metabolism [30]. Recent studies from others, taking advantage of more advanced mass spectrometer systems, describe the distribution of acetylated lysine residues and their involved functions in three human cell lines [5], human liver tissue [6], and *Salmonella enterica* [44]. To understand their true contribution to spatial and temporal functions, a comprehensive map of K-Ac sites along with their dynamic profiling under diverse physiologic conditions needs to be constructed.

Our integrated data analyses reveal that a large fraction of lysine acetylation sites are in known functional domains and involved in multiple known pathways. This supports the theory that various domains can communicate to couple PTMs to the organization of the cell [45]. Furthermore, the high conservation of K-Ac sites suggests that lysine acetylation events are under positive selection pressure because of their essential functions. One must be careful in interpreting these results, as they may be affected by the abundance of proteins. As with gene expression and proteomics studies, results may be biased towards the most prevalent genes and proteins. Nevertheless, with this caveat, the widespread presence of lysine acetylation sites is revealing.

Using a substantially larger and more diverse dataset, we recapitulated several exercises performed by Choudhary, *et al.*, and several important differences are noted. First, our data reveal a more substantial overlap of localization across subcellular domains (Figure 2), likely owing to the diverse array of proteins represented. In the Choudhary analysis, mention of localization overlap is only made for the nuclear and cytosolic compartments, while in our analysis, we found significant overlap additionally with the mitochondrial compartment. Indeed, we found 1,133 (47.2%) of the proteins to be localized in more than one compartment (Figure 2). Second, our findings suggest that while the majority of lysine sites are in coiled regions, most K-Ac sites are in alpha helices (Figure 5), while Choudhary identified most K-Ac sites in coiled regions. While it is difficult to make generalizations about the implications of this finding, one intriguing explanation for at least part of this enrichment is that the acetylation of lysine is known to be destabilizing to alpha helices [46], [47], and this may be responsible for changes in higher-order folding such as seen in nucleosomes [48]. Finally, our analysis (Figure 4) revealed significant enrichment for K-Ac residues in chaperones HSP70 and HSP90, likely reflecting the importance of post-translational modification in their regulation.

Several models have been proposed to interpret the crosstalk between acetylation of lysine and other PTMs. Yang, et al. has summarized these potential interactions and postulates the mechanisms by which lysine acetylation can program protein function [31]. A cluster of autoacetylated lysines within the acetyltransferase CBP/p300 forms a charged patch that can act as an activation switch [49]. Indeed, in our data, 34.2% of K-Ac sites have neighboring lysines with the potential to be acetylated. A more difficult analysis is to predict autoacetylation between adjacent subunits of a homodimer, as in the case of CBP/p300, which appears also to be acetylated via intermolecular mechanisms [50].

Lysine acetylation can alter protein function through bromodomain binding, as when p53 forms docking sites for transcriptional coactivators such as the CBP [15], [51]. Another study reports that *Salmonella enterica* synthetase is modulated through lysine acetylation [52]. Finally, some nuclear receptors appear to be regulated by acetylation status, such as deacetylation of Liver X receptor by SIRT1 [53].

Beyond affecting neighboring lysine residues, acetylation has the potential to communicate with surrounding phosphorylation, methylation and ubiquitination sites. Acetylation of p53 at Lys370 and Lys372 impacts phosphorylation of Ser371, affecting downstream signaling and function [54]. We identified over 4,000 phosphorylation sites that could potentially be influenced by nearby lysine acetylation. Phosphorylation could be affected through the creation or destruction of a phosphorylation site or through alterations in kinase binding (Figure 6). These findings are supported by earlier work demonstrating similar changes in phosphorylation sites and kinase binding domains due to alterations in phosphorylation status [24], [55]. Positive crosstalk has been reported between acetylation of Lys14 and phosphorylation of Ser10 in histone H3 [20], and our findings correctly predict this interaction. We found less than 80 methylation sites potentially affected by lysine acetylation, but this number may increase as more PTMs are identified. Acetylation and methylation on H3 Lys-9 have been reported to be mutually exclusive and associated with active and repressive gene expression, respectively [23]. Our predictions for this protein are consistent with these findings. Further, our simulations predict that over 600 ubiquitination sites may be affected by lysine acetylation, and this may have a profound impact on cellular degradation pathways. These findings are supported by recent work identifying extensive overlap between lysine acetylation and ubiquitination [56], [57]. Finally, the dataset described in this paper will assist researchers in the community in testing the possible interactions between lysine acetylation and other modifications.

Cellular localization can also be influenced through lysine acetylation. Acetylation by CBP of FoxO transcription factors promotes phosphorylation of Ser256, leading to cytoplasmic retention [58]. In addition, the acetylated lysines are in the DNA binding domain and could thus impede nuclear import. Our work demonstrates that acetylation sites are distributed throughout the cytoplasm, mitochondria, and nucleus, providing fertile substrate for such localization control (Figure 2).

It is therefore apparent that acetylation of lysine is one of many PTMs involved in a complicated language of crosstalk and communication that maintains delicate control within the cell. This PTM code was first described for p53 [15] but has now been extended to many other genes and pathways [59], [60], [61]. Indeed, our work has demonstrated the broad range of biologic pathways (Figure 3) and domains (Figure 4) that harbor acetylated lysine residues. This wide distribution of PTMs and the dependent relationship between acetylation, phosphorylation, methylation, ubiquitination, and other PTMs strongly implies that crosstalk is a common mechanism and enables the organism to finely tune protein functions at the translational level. In fact, the high degree of evolutionary conservation of lysine acetylation [62] implies a similar degree of conservation and more broad implications for the crosstalk mechanisms described here. In transcriptional regulation, it is known that factors with opposing activity can bind regulatory elements of the same gene [63], and these “dual genes” can tune expression more precisely and under more stringent control. In a similar fashion, it is likely that PTMs may exert an analogous effect, yet it is unclear how extensive this control is, and therefore a more complete PTM dataset and further investigation is warranted.

## Materials and Methods

### Data collection and integration

All scripts were written in Perl and are available upon request. Acetylated lysine sites were collected from three sources: PhosphoSitePlus [25], Uniprot [26], [27], and from a paper reported by Choudhary and his colleagues [5]. Protein-BLAST (BLAST-P, basic local alignment search tool) was used to map protein sequences to an IPI database (v3.69) [28]. Phosphorylation sites were derived in a similar manner, using PhosphoSitePlus, Uniprot and Phospho.ELM [29]. The lists were consolidated to remove any redundant sites.

### Conservation of K-Ac sites between human and mouse

Using BLAST-P, we compared 87,130 human protein sequences and 56,737 mouse protein sequences from the IPI database. Using a significance e-value of 10^−6^, we compared the amino acid sequence conservation between human and mouse in order to determine the conservation across the K-Ac residues identified during our data integration phase. A Fisher's exact test was used to determine the significance of the conservation rate to all amino acids and to only lysine residues.

### Subcellular localization of acetylated lysine residues

We queried the Gene Ontology Annotation database (GOA) [32] with the IPI numbers of our identified K-Ac genes to get the cellular component annotation. Using this information, we determined the subcellular localizations of these gene products.

### Biologic pathway analysis

We used the Kyoto Encyclopedia of Genes and Genomes (KEGG [64]) to identify enriched pathways. A two-tailed Fisher's exact test was employed to test the enrichment of the K-Ac-containing IPI entries against all IPI proteins. Correction for multiple hypothesis testing was carried out using standard false discovery rate control methods. The KEGG pathways with a corrected *p*-value<0.01 were considered significant. These pathways were classified into hierarchical categories according to the KEGG website.

### Domain analysis

Using our K-Ac-containing proteins, we searched the NCBI Conserved Domain Database (CDD) using reverse PSI-BLAST (reverse position specific iterative BLAST, RPS-BLAST) [34], [35]. A two-tailed Fisher's exact test was used to compare the distribution of K-Ac sites within each domain to the ratio over all proteins. Correction for multiple hypothesis testing was carried out using standard false discovery rate control methods, and domains with a corrected *p*-value<0.01 were considered significant. Domains with more than the number expected K-Ac sites were considered “overrepresented,” while those with fewer-than-expected K-Ac sites were categorized as “underrepresented.”

### Secondary structure prediction

We derived secondary structure information from the Dictionary of Protein Secondary Structure (DSSP [39]) as well as the Uniprot database.

### Crosstalk prediction

To survey the contribution of K-Ac to the modification capacity of nearby sites, we choose two other amino acids – glutamine (Q) and leucine (L) as substitutes for lysine. NetPhosK 1.0 [40], [41] was employed to predict kinase-specific phosphorylation for nearby sites, using the default program settings. The sites where the phosphorylation binding site or the kinase binding were affected by the change from K to Q or L were tallied. We classified the affected phosphorylation sites into five categories: Types I and II: gain and loss of a phosphorylation site, respectively; Type III: retention of a phosphorylation site and gain of a new kinase binding site; Type IV: retention of a phosphorylation site but loss of kinase binding site; Type V: loss of an endogenous kinase binding site with simultaneous gain of a new one.

In addition to phosphorylation, we studied the effects on ubiquitination using UbPred (cutoff 0.84) [43] and on methylation, using BPB-PPMS (cutoff 0.8) [42]. In a similar fashion, the contribution of lysine substitution by either leucine or glutamine was studied. Since neither can be methylated or ubiquitinated, substitution resulted in a loss of self-modification. We classified the changes into three groups: Type I: gain of a methylation or ubiquitination on a nearby lysine; Type IIs (self): loss of methylation or ubiquitination on the lysine itself; Type IIn (neighbor): loss of methylation or ubiquitination on a nearby lysine.

## Supporting Information

Figure S1 **Distance distribution of altered phosphorylation sites.** Each acetylated lysine was substituted with leucine (top) or glutamine (bottom). The neighborhood surrounding the K-Ac was searched for potential phosphorylation sites. The number of altered phosphorylation sites within 10 residues of the K-Ac site are plotted. For further details, refer to the text.
(TIFF)

Figure S2 **Distance distribution of altered methylation sites.** Each acetylated lysine was substituted with leucine (top) or glutamine (bottom). The neighborhood surrounding the K-Ac was searched for potential methylation sites, and these are plotted. All changes were found within five residues of the K-Ac. For further details, refer to the text.
(TIFF)

Figure S3 **Distance distribution of altered ubiquitination sites.** Each acetylated lysine was substituted with leucine (top) or glutamine (bottom). The neighborhood surrounding the K-Ac was searched for potential ubiquination sites, and these are plotted. For further details, refer to the text.
(TIFF)

Table S1 **All identified acetylation sites.** These are the complete data for the identified acetylation sites. For details, refer to text.
(PDF)

Table S2 **Domain enrichment.** These are the complete data for Figure 4. Proteins with acetylated lysines were aligned to the distribution of domains in the NCBI Conserved Domain Database using PRS-BLAST. The distribution was tested against that of all IPI proteins using a two-tailed Fisher's exact test.
(PDF)

Table S3 **Phosphorylation sites affected.** NetPhosK 1.0 was employed to predict kinase-specific phosphorylation for nearby sites, using the default program settings. The sites where the phosphorylation binding site or the kinase binding were affected by the change from K to Q or L were tallied.
(PDF)

Table S4 **Methylation sites affected.** We studied the effects on methylation using BPB-PPMS (cutoff 0.8). The contribution of lysine substitution by either leucine or glutamine was studied.
(PDF)

Table S5 **Ubiquitination sites affected.** We studied the effects on ubiquitination using UbPred (cutoff 0.84). The contribution of lysine substitution by either leucine or glutamine was studied.
(PDF)
