# Supplementary figures and images for: Bioinformatic Analysis and Post-Translational Modification Crosstalk Prediction of Lysine Acetylation
Source: PLoS One. 2011 Dec 2;6(12):e28228. doi: 10.1371/journal.pone.0028228 (PMC3229533; doi:10.1371/journal.pone.0028228)

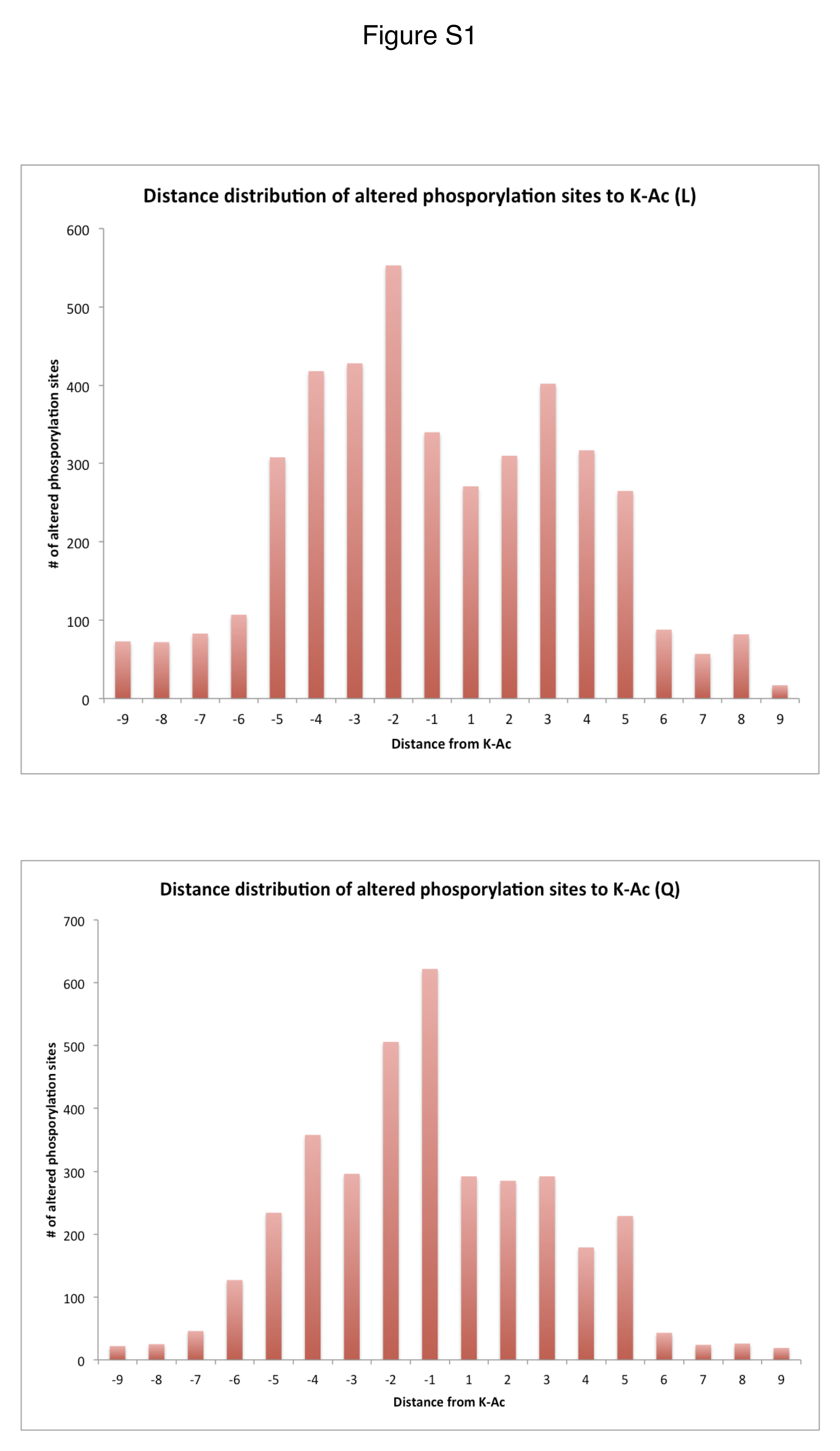

Supplement: Figure S1 — Distance distribution of altered phosphorylation sites. Each acetylated lysine was substituted with leucine (top) or glutamine (bottom). The neighborhood surrounding the K-Ac was searched for potential phosphorylation sites. The number of altered phosphorylation sites within 10 residues of the K-Ac site are plotted. For further details, refer to the text. (TIFF) [file pone.0028228.s001.tiff]

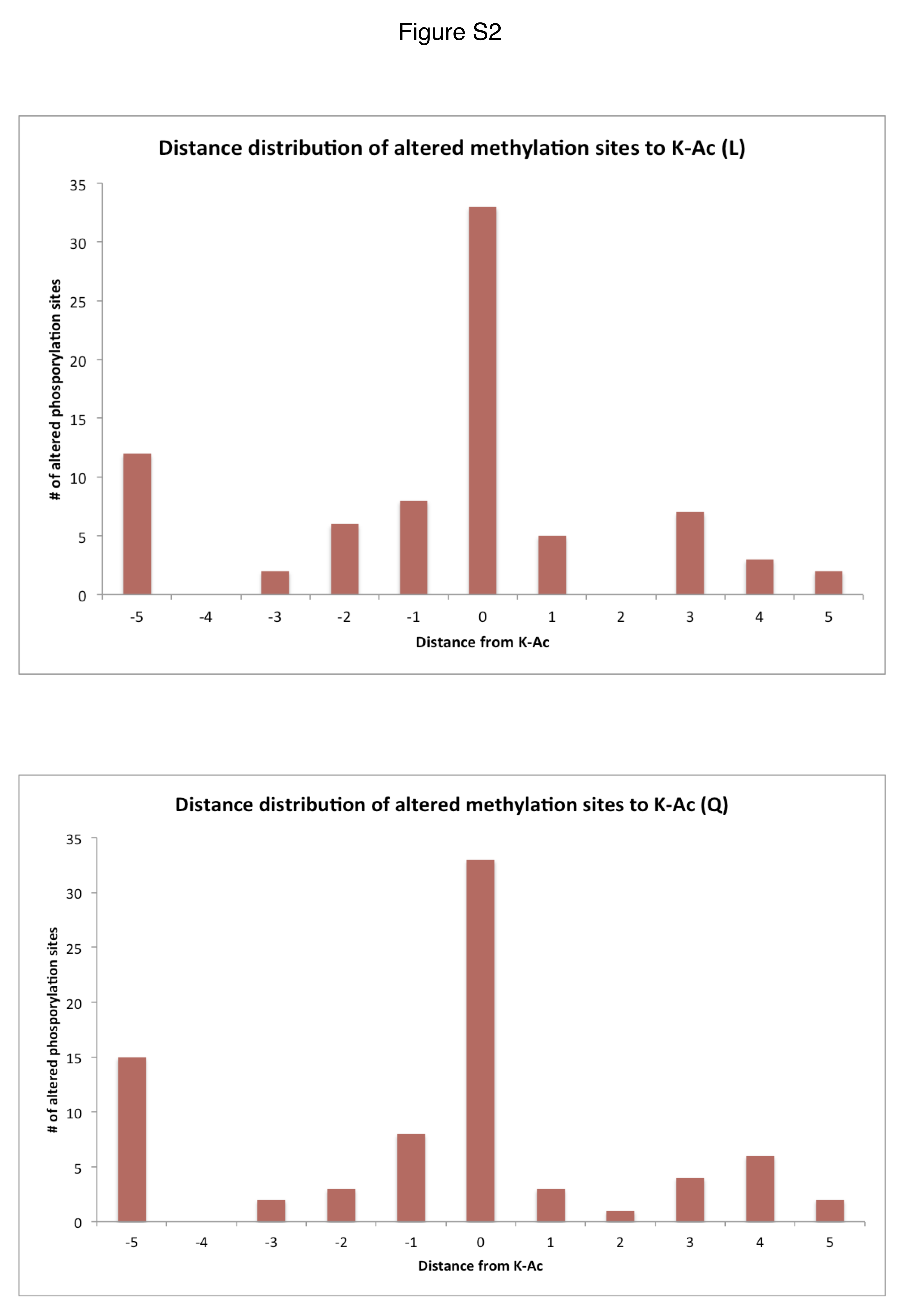

Supplement: Figure S2 — Distance distribution of altered methylation sites. Each acetylated lysine was substituted with leucine (top) or glutamine (bottom). The neighborhood surrounding the K-Ac was searched for potential methylation sites, and these are plotted. All changes were found within five residues of the K-Ac. For further details, refer to the text. (TIFF) [file pone.0028228.s002.tiff]

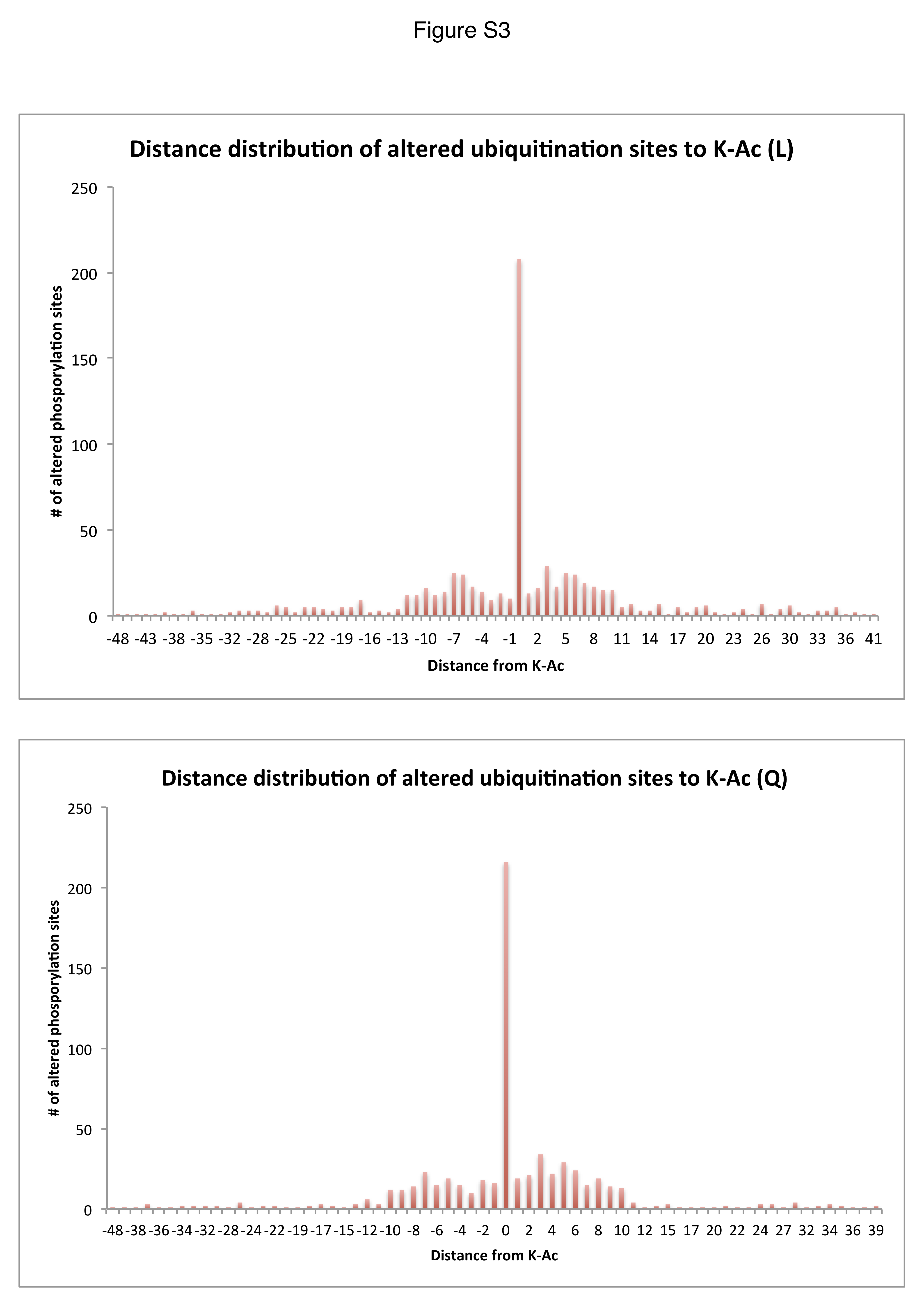

Supplement: Figure S3 — Distance distribution of altered ubiquitination sites. Each acetylated lysine was substituted with leucine (top) or glutamine (bottom). The neighborhood surrounding the K-Ac was searched for potential ubiquination sites, and these are plotted. For further details, refer to the text. (TIFF) [file pone.0028228.s003.tiff]
