# Supplementary material for: Bioinformatic Analysis and Post-Translational Modification Crosstalk Prediction of Lysine Acetylation
Source: PLoS One. 2011 Dec 2;6(12):e28228. doi: 10.1371/journal.pone.0028228 (PMC3229533; doi:10.1371/journal.pone.0028228)
Supplement: Table S1 — All identified acetylation sites. These are the complete data for the identified acetylation sites. For details, refer to text. (PDF) [file pone.0028228.s004.pdf]

Table S1

IPI	Acetylation	Position
IPI00000001	K	270
IPI00000057	K	3
IPI00000104	K	395
IPI00000104	K	397
IPI00000155	K	25
IPI00000155	K	72
IPI00000155	K	77
IPI00000279	K	27
IPI00000352	K	57
IPI00000398	K	411
IPI00000425	K	279
IPI00000494	K	5
IPI00000494	K	48
IPI00000494	K	221
IPI00000606	K	227
IPI00000643	K	111
IPI00000684	K	180
IPI00000690	K	295
IPI00000690	K	593
IPI00000712	K	438
IPI00000712	K	443
IPI00000712	K	445
IPI00000712	K	462
IPI00000712	K	472
IPI00000712	K	487
IPI00000712	K	506
IPI00000712	K	508
IPI00000712	K	516
IPI00000712	K	518
IPI00000712	K	533
IPI00000712	K	536
IPI00000712	K	538
IPI00000712	K	541
IPI00000712	K	543
IPI00000712	K	548
IPI00000712	K	554
IPI00000712	K	555
IPI00000784	K	4
IPI00000784	K	116
IPI00000787	K	53
IPI00000787	K	109
IPI00000792	K	23
IPI00000792	K	208
IPI00000816	K	50
IPI00000816	K	69
IPI00000816	K	118
IPI00000816	K	123
IPI00000873	K	420
IPI00000873	K	423
IPI00000873	K	645
IPI00000874	K	7

IPI00000874	K	16
IPI00000874	K	27
IPI00000874	K	35
IPI00000874	K	37
IPI00000874	K	168
IPI00000874	K	197
IPI00000874	K	199
IPI00000948	K	314
IPI00000948	K	315
IPI00001091	K	306
IPI00001091	K	308
IPI00001091	K	543
IPI00001146	K	59
IPI00001364	K	278
IPI00001364	K	280
IPI00001432	K	299
IPI00001453	K	290
IPI00001458	K	1316
IPI00001458	K	1318
IPI00001539	K	25
IPI00001539	K	81
IPI00001539	K	137
IPI00001539	K	181
IPI00001539	K	270
IPI00001600	K	203
IPI00001600	K	209
IPI00001639	K	211
IPI00001639	K	835
IPI00001639	K	867
IPI00001639	K	871
IPI00001699	K	21
IPI00001699	K	22
IPI00001712	K	860
IPI00001735	K	878
IPI00001735	K	959
IPI00001735	K	1218
IPI00001735	K	1248
IPI00001735	K	1795
IPI00001735	K	1970
IPI00001735	K	2037
IPI00001883	K	288
IPI00001952	K	407
IPI00001960	K	130
IPI00001985	K	362
IPI00002144	K	321
IPI00002188	K	1708
IPI00002214	K	22
IPI00002255	K	2116
IPI00002255	K	2117
IPI00002270	K	40
IPI00002270	K	73
IPI00002324	K	46

Table S1

IPI00002324	K	299
IPI00002335	K	6
IPI00002335	K	444
IPI00002349	K	140
IPI00002372	K	260
IPI00002372	K	399
IPI00002408	K	216
IPI00002424	K	44
IPI00002460	K	233
IPI00002503	K	343
IPI00002503	K	348
IPI00002519	K	271
IPI00002520	K	103
IPI00002520	K	181
IPI00002520	K	196
IPI00002520	K	269
IPI00002520	K	297
IPI00002520	K	356
IPI00002520	K	464
IPI00002520	K	469
IPI00002520	K	474
IPI00002521	K	41
IPI00002521	K	46
IPI00002521	K	94
IPI00002521	K	99
IPI00002521	K	105
IPI00002525	K	136
IPI00002538	K	304
IPI00002564	K	256
IPI00002564	K	260
IPI00002564	K	271
IPI00002852	K	84
IPI00002852	K	86
IPI00002894	K	1007
IPI00002922	K	254
IPI00002938	K	88
IPI00002966	K	272
IPI00002966	K	430
IPI00002966	K	603
IPI00002966	K	679
IPI00002993	K	5
IPI00002993	K	108
IPI00003031	K	198
IPI00003168	K	188
IPI00003168	K	194
IPI00003168	K	251
IPI00003362	K	97
IPI00003362	K	114
IPI00003362	K	269
IPI00003362	K	377
IPI00003362	K	548
IPI00003362	K	586

IPI00003362	K	652
IPI00003377	K	24
IPI00003377	K	185
IPI00003419	K	174
IPI00003419	K	179
IPI00003420	K	271
IPI00003438	K	146
IPI00003442	K	389
IPI00003442	K	532
IPI00003443	K	86
IPI00003443	K	208
IPI00003443	K	214
IPI00003482	K	110
IPI00003482	K	230
IPI00003494	K	48
IPI00003495	K	601
IPI00003527	K	32
IPI00003527	K	34
IPI00003588	K	12
IPI00003588	K	138
IPI00003588	K	166
IPI00003766	K	66
IPI00003766	K	172
IPI00003768	K	98
IPI00003814	K	82
IPI00003815	K	105
IPI00003815	K	127
IPI00003815	K	141
IPI00003815	K	178
IPI00003817	K	21
IPI00003817	K	25
IPI00003817	K	30
IPI00003817	K	33
IPI00003817	K	40
IPI00003817	K	47
IPI00003817	K	95
IPI00003817	K	102
IPI00003817	K	124
IPI00003817	K	175
IPI00003833	K	158
IPI00003865	K	71
IPI00003865	K	88
IPI00003865	K	108
IPI00003865	K	112
IPI00003865	K	246
IPI00003865	K	319
IPI00003865	K	348
IPI00003865	K	512
IPI00003865	K	524
IPI00003865	K	526
IPI00003865	K	531
IPI00003865	K	589

IPI00003865	K	597
IPI00003865	K	601
IPI00003870	K	211
IPI00003881	K	87
IPI00003881	K	224
IPI00003918	K	14
IPI00003918	K	106
IPI00003918	K	163
IPI00003918	K	333
IPI00003918	K	412
IPI00003923	K	162
IPI00003925	K	354
IPI00003933	K	229
IPI00003935	K	6
IPI00003935	K	12
IPI00003935	K	13
IPI00003935	K	16
IPI00003935	K	17
IPI00003935	K	21
IPI00003935	K	109
IPI00003935	K	121
IPI00003944	K	295
IPI00003949	K	10
IPI00003949	K	82
IPI00003949	K	92
IPI00003949	K	94
IPI00003965	K	595
IPI00003965	K	869
IPI00003965	K	1084
IPI00003965	K	1096
IPI00003968	K	370
IPI00003970	K	119
IPI00004233	K	993
IPI00004233	K	1165
IPI00004233	K	1178
IPI00004233	K	1185
IPI00004233	K	1639
IPI00004233	K	1938
IPI00004233	K	2005
IPI00004233	K	2264
IPI00004233	K	2421
IPI00004312	K	375
IPI00004344	K	79
IPI00004350	K	12
IPI00004358	K	316
IPI00004358	K	811
IPI00004363	K	351
IPI00004461	K	56
IPI00004461	K	275
IPI00004521	K	256
IPI00004527	K	1054
IPI00004527	K	1059

IPI00004566	K	296
IPI00004655	K	199
IPI00004845	K	45
IPI00004845	K	48
IPI00004845	K	166
IPI00004859	K	755
IPI00004859	K	863
IPI00004859	K	1411
IPI00004860	K	60
IPI00004860	K	205
IPI00004860	K	287
IPI00004860	K	288
IPI00004924	K	225
IPI00004968	K	122
IPI00004968	K	261
IPI00005036	K	542
IPI00005038	K	101
IPI00005045	K	7
IPI00005045	K	8
IPI00005045	K	9
IPI00005045	K	304
IPI00005079	K	64
IPI00005132	K	502
IPI00005154	K	33
IPI00005154	K	90
IPI00005154	K	233
IPI00005154	K	413
IPI00005154	K	542
IPI00005154	K	548
IPI00005159	K	299
IPI00005159	K	322
IPI00005160	K	44
IPI00005160	K	82
IPI00005161	K	275
IPI00005161	K	295
IPI00005162	K	56
IPI00005162	K	61
IPI00005292	K	118
IPI00005347	K	260
IPI00005367	K	9
IPI00005367	K	10
IPI00005492	K	112
IPI00005502	K	642
IPI00005511	K	3
IPI00005578	K	327
IPI00005614	K	90
IPI00005614	K	1815
IPI00005614	K	1913
IPI00005614	K	1989
IPI00005614	K	2269
IPI00005615	K	122
IPI00005630	K	117

IPI00005630	K	120
IPI00005630	K	125
IPI00005648	K	293
IPI00005648	K	424
IPI00005648	K	429
IPI00005648	K	616
IPI00005648	K	618
IPI00005648	K	829
IPI00005657	K	21
IPI00005657	K	66
IPI00005658	K	48
IPI00005658	K	54
IPI00005668	K	4
IPI00005668	K	31
IPI00005668	K	33
IPI00005668	K	161
IPI00005668	K	179
IPI00005668	K	246
IPI00005677	K	128
IPI00005677	K	643
IPI00005677	K	644
IPI00005724	K	142
IPI00005744	K	30
IPI00005792	K	207
IPI00005822	K	467
IPI00005966	K	43
IPI00005966	K	47
IPI00005969	K	19
IPI00005969	K	97
IPI00005969	K	273
IPI00005978	K	36
IPI00005996	K	26
IPI00006034	K	23
IPI00006034	K	138
IPI00006077	K	30
IPI00006077	K	32
IPI00006077	K	38
IPI00006077	K	39
IPI00006077	K	461
IPI00006077	K	638
IPI00006077	K	735
IPI00006077	K	738
IPI00006079	K	152
IPI00006079	K	335
IPI00006079	K	421
IPI00006079	K	437
IPI00006079	K	637
IPI00006091	K	1017
IPI00006092	K	149
IPI00006108	K	138
IPI00006108	K	188
IPI00006108	K	405

IPI00006108	K	407
IPI00006108	K	822
IPI00006160	K	321
IPI00006160	K	327
IPI00006160	K	331
IPI00006164	K	210
IPI00006167	K	383
IPI00006171	K	1262
IPI00006176	K	207
IPI00006176	K	494
IPI00006181	K	53
IPI00006204	K	315
IPI00006211	K	3
IPI00006213	K	399
IPI00006252	K	33
IPI00006379	K	441
IPI00006379	K	460
IPI00006379	K	461
IPI00006379	K	463
IPI00006440	K	208
IPI00006440	K	228
IPI00006475	K	144
IPI00006579	K	53
IPI00006579	K	60
IPI00006601	K	153
IPI00006615	K	589
IPI00006658	K	57
IPI00006663	K	81
IPI00006663	K	355
IPI00006663	K	368
IPI00006725	K	642
IPI00006863	K	166
IPI00006865	K	38
IPI00006907	K	50
IPI00006932	K	188
IPI00006935	K	47
IPI00006935	K	68
IPI00006937	K	8
IPI00006937	K	17
IPI00006952	K	102
IPI00006952	K	103
IPI00006952	K	247
IPI00006980	K	20
IPI00006980	K	41
IPI00006980	K	58
IPI00006980	K	62
IPI00006980	K	98
IPI00006987	K	17
IPI00006987	K	71
IPI00007004	K	208
IPI00007009	K	44
IPI00007009	K	47

IPI00007049	K	131
IPI00007058	K	233
IPI00007074	K	146
IPI00007074	K	147
IPI00007074	K	197
IPI00007074	K	206
IPI00007074	K	272
IPI00007074	K	474
IPI00007074	K	482
IPI00007074	K	490
IPI00007084	K	484
IPI00007088	K	169
IPI00007123	K	86
IPI00007128	K	197
IPI00007156	K	95
IPI00007188	K	23
IPI00007188	K	92
IPI00007188	K	94
IPI00007188	K	96
IPI00007188	K	105
IPI00007188	K	163
IPI00007188	K	166
IPI00007188	K	199
IPI00007188	K	268
IPI00007188	K	272
IPI00007189	K	135
IPI00007189	K	144
IPI00007247	K	60
IPI00007311	K	53
IPI00007311	K	60
IPI00007311	K	71
IPI00007311	K	76
IPI00007334	K	359
IPI00007334	K	717
IPI00007346	K	38
IPI00007395	K	40
IPI00007404	K	306
IPI00007423	K	86
IPI00007611	K	54
IPI00007611	K	60
IPI00007611	K	70
IPI00007611	K	158
IPI00007611	K	162
IPI00007611	K	172
IPI00007611	K	176
IPI00007611	K	192
IPI00007641	K	3
IPI00007641	K	34
IPI00007675	K	429
IPI00007694	K	6
IPI00007694	K	30
IPI00007722	K	516

IPI00007750	K	40
IPI00007750	K	163
IPI00007750	K	280
IPI00007750	K	311
IPI00007750	K	394
IPI00007750	K	401
IPI00007752	K	58
IPI00007752	K	103
IPI00007752	K	297
IPI00007752	K	379
IPI00007755	K	115
IPI00007756	K	101
IPI00007757	K	288
IPI00007765	K	121
IPI00007765	K	135
IPI00007765	K	138
IPI00007765	K	143
IPI00007765	K	234
IPI00007765	K	288
IPI00007765	K	300
IPI00007765	K	345
IPI00007765	K	567
IPI00007765	K	595
IPI00007765	K	610
IPI00007765	K	646
IPI00007765	K	675
IPI00007797	K	17
IPI00007797	K	55
IPI00007811	K	35
IPI00007913	K	128
IPI00007927	K	114
IPI00007927	K	222
IPI00007927	K	320
IPI00007927	K	321
IPI00007927	K	330
IPI00007927	K	677
IPI00007927	K	1158
IPI00007927	K	1160
IPI00007928	K	1463
IPI00007940	K	269
IPI00007941	K	165
IPI00007983	K	282
IPI00007993	K	333
IPI00008054	K	105
IPI00008054	K	447
IPI00008054	K	449
IPI00008054	K	546
IPI00008054	K	550
IPI00008054	K	554
IPI00008054	K	671
IPI00008091	K	118
IPI00008164	K	157

IPI00008215	K	60
IPI00008215	K	84
IPI00008223	K	45
IPI00008240	K	726
IPI00008248	K	263
IPI00008274	K	81
IPI00008274	K	209
IPI00008359	K	193
IPI00008380	K	4
IPI00008422	K	729
IPI00008433	K	47
IPI00008433	K	182
IPI00008438	K	17
IPI00008438	K	24
IPI00008454	K	43
IPI00008454	K	47
IPI00008475	K	46
IPI00008475	K	246
IPI00008475	K	273
IPI00008475	K	321
IPI00008475	K	330
IPI00008475	K	409
IPI00008477	K	305
IPI00008524	K	104
IPI00008524	K	108
IPI00008524	K	213
IPI00008524	K	259
IPI00008524	K	512
IPI00008528	K	49
IPI00008528	K	54
IPI00008529	K	21
IPI00008529	K	24
IPI00008529	K	49
IPI00008530	K	77
IPI00008530	K	246
IPI00008531	K	418
IPI00008552	K	92
IPI00008570	K	152
IPI00008570	K	153
IPI00008575	K	165
IPI00008575	K	169
IPI00008575	K	175
IPI00008579	K	202
IPI00008599	K	221
IPI00008603	K	63
IPI00008603	K	328
IPI00008708	K	461
IPI00008708	K	468
IPI00008728	K	178
IPI00008728	K	437
IPI00008868	K	506
IPI00008868	K	685

IPI00008868	K	686
IPI00008868	K	693
IPI00008868	K	694
IPI00008868	K	790
IPI00008934	K	83
IPI00008934	K	437
IPI00008943	K	229
IPI00008965	K	268
IPI00008965	K	271
IPI00008965	K	273
IPI00008982	K	649
IPI00009032	K	116
IPI00009032	K	128
IPI00009032	K	328
IPI00009032	K	360
IPI00009104	K	417
IPI00009213	K	851
IPI00009236	K	5
IPI00009247	K	31
IPI00009253	K	94
IPI00009253	K	203
IPI00009286	K	636
IPI00009286	K	1130
IPI00009286	K	1133
IPI00009286	K	1235
IPI00009305	K	50
IPI00009305	K	51
IPI00009305	K	64
IPI00009328	K	60
IPI00009328	K	296
IPI00009328	K	321
IPI00009373	K	69
IPI00009373	K	74
IPI00009373	K	91
IPI00009439	K	237
IPI00009448	K	81
IPI00009532	K	333
IPI00009604	K	466
IPI00009634	K	135
IPI00009634	K	173
IPI00009634	K	180
IPI00009688	K	89
IPI00009688	K	91
IPI00009688	K	145
IPI00009713	K	61
IPI00009713	K	253
IPI00009713	K	398
IPI00009724	K	169
IPI00009724	K	172
IPI00009724	K	175
IPI00009724	K	179
IPI00009724	K	188

IPI00009753	K	176
IPI00009771	K	81
IPI00009771	K	520
IPI00009790	K	395
IPI00009790	K	486
IPI00009790	K	688
IPI00009790	K	753
IPI00009822	K	81
IPI00009844	K	13
IPI00009844	K	291
IPI00009867	K	178
IPI00009889	K	26
IPI00009895	K	121
IPI00009901	K	4
IPI00009904	K	256
IPI00009904	K	366
IPI00009904	K	484
IPI00009904	K	533
IPI00009931	K	15
IPI00009943	K	93
IPI00009943	K	97
IPI00009943	K	112
IPI00009958	K	47
IPI00009958	K	326
IPI00009960	K	211
IPI00009960	K	222
IPI00009960	K	451
IPI00009960	K	506
IPI00009992	K	272
IPI00010080	K	42
IPI00010080	K	46
IPI00010085	K	250
IPI00010085	K	269
IPI00010085	K	356
IPI00010090	K	263
IPI00010133	K	339
IPI00010133	K	449
IPI00010157	K	81
IPI00010158	K	102
IPI00010188	K	90
IPI00010188	K	93
IPI00010190	K	151
IPI00010190	K	291
IPI00010193	K	399
IPI00010196	K	111
IPI00010196	K	158
IPI00010196	K	286
IPI00010196	K	310
IPI00010196	K	446
IPI00010196	K	481
IPI00010196	K	528
IPI00010196	K	606

IPI00010196	K	931
IPI00010204	K	23
IPI00010214	K	27
IPI00010252	K	252
IPI00010252	K	763
IPI00010252	K	769
IPI00010252	K	951
IPI00010252	K	953
IPI00010270	K	147
IPI00010290	K	46
IPI00010320	K	33
IPI00010320	K	35
IPI00010320	K	41
IPI00010320	K	84
IPI00010320	K	139
IPI00010346	K	664
IPI00010349	K	102
IPI00010349	K	169
IPI00010349	K	347
IPI00010365	K	197
IPI00010404	K	17
IPI00010414	K	22
IPI00010415	K	78
IPI00010415	K	168
IPI00010415	K	198
IPI00010415	K	283
IPI00010420	K	104
IPI00010471	K	76
IPI00010471	K	88
IPI00010471	K	294
IPI00010471	K	297
IPI00010471	K	361
IPI00010471	K	468
IPI00010471	K	472
IPI00010471	K	542
IPI00010471	K	579
IPI00010586	K	522
IPI00010700	K	27
IPI00010700	K	29
IPI00010700	K	1196
IPI00010706	K	186
IPI00010720	K	223
IPI00010720	K	232
IPI00010720	K	399
IPI00010740	K	319
IPI00010740	K	330
IPI00010740	K	338
IPI00010740	K	421
IPI00010740	K	472
IPI00010796	K	103
IPI00010796	K	375
IPI00010796	K	444

IPI00010833	K	607
IPI00010847	K	122
IPI00010847	K	125
IPI00010847	K	131
IPI00010863	K	57
IPI00010863	K	60
IPI00010872	K	149
IPI00010896	K	13
IPI00010896	K	49
IPI00010896	K	119
IPI00010896	K	131
IPI00010896	K	135
IPI00011051	K	236
IPI00011062	K	55
IPI00011062	K	119
IPI00011062	K	287
IPI00011062	K	527
IPI00011062	K	841
IPI00011062	K	892
IPI00011062	K	1291
IPI00011069	K	5
IPI00011069	K	295
IPI00011075	K	129
IPI00011102	K	242
IPI00011107	K	67
IPI00011107	K	80
IPI00011107	K	106
IPI00011107	K	133
IPI00011107	K	155
IPI00011107	K	166
IPI00011107	K	180
IPI00011107	K	256
IPI00011107	K	263
IPI00011107	K	272
IPI00011107	K	275
IPI00011107	K	282
IPI00011107	K	413
IPI00011107	K	442
IPI00011126	K	258
IPI00011201	K	94
IPI00011201	K	156
IPI00011201	K	224
IPI00011201	K	240
IPI00011201	K	272
IPI00011201	K	346
IPI00011253	K	62
IPI00011268	K	165
IPI00011274	K	216
IPI00011276	K	241
IPI00011285	K	84
IPI00011307	K	50
IPI00011416	K	83

IPI00011416	K	95
IPI00011416	K	318
IPI00011416	K	327
IPI00011511	K	69
IPI00011518	K	126
IPI00011518	K	275
IPI00011518	K	279
IPI00011518	K	285
IPI00011518	K	299
IPI00011518	K	300
IPI00011518	K	307
IPI00011568	K	136
IPI00011603	K	14
IPI00011631	K	777
IPI00011696	K	222
IPI00011696	K	252
IPI00011696	K	716
IPI00011770	K	55
IPI00011857	K	494
IPI00011875	K	83
IPI00011875	K	98
IPI00011875	K	386
IPI00011875	K	408
IPI00011923	K	250
IPI00011970	K	321
IPI00012007	K	188
IPI00012007	K	401
IPI00012007	K	408
IPI00012011	K	13
IPI00012011	K	19
IPI00012011	K	44
IPI00012011	K	73
IPI00012011	K	92
IPI00012011	K	95
IPI00012011	K	114
IPI00012011	K	121
IPI00012011	K	132
IPI00012011	K	144
IPI00012028	K	41
IPI00012069	K	59
IPI00012069	K	209
IPI00012069	K	262
IPI00012079	K	591
IPI00012119	K	138
IPI00012119	K	147
IPI00012149	K	609
IPI00012174	K	114
IPI00012174	K	115
IPI00012174	K	117
IPI00012174	K	120
IPI00012199	K	212
IPI00012199	K	213

IPI00012341	K	167
IPI00012345	K	101
IPI00012382	K	60
IPI00012442	K	353
IPI00012442	K	357
IPI00012442	K	376
IPI00012446	K	34
IPI00012446	K	38
IPI00012446	K	72
IPI00012535	K	32
IPI00012535	K	44
IPI00012535	K	66
IPI00012567	K	322
IPI00012567	K	327
IPI00012567	K	329
IPI00012567	K	332
IPI00012587	K	6
IPI00012587	K	125
IPI00012587	K	128
IPI00012587	K	402
IPI00012726	K	104
IPI00012726	K	108
IPI00012728	K	543
IPI00012728	K	632
IPI00012750	K	52
IPI00012750	K	60
IPI00012750	K	66
IPI00012750	K	94
IPI00012756	K	150
IPI00012759	K	14
IPI00012772	K	46
IPI00012773	K	626
IPI00012788	K	176
IPI00012795	K	264
IPI00012816	K	265
IPI00012816	K	274
IPI00012816	K	288
IPI00012820	K	379
IPI00012837	K	28
IPI00012837	K	32
IPI00012837	K	166
IPI00012912	K	510
IPI00012912	K	537
IPI00012912	K	544
IPI00012948	K	95
IPI00012948	K	96
IPI00012948	K	97
IPI00012948	K	99
IPI00012948	K	104
IPI00012972	K	91
IPI00013068	K	275
IPI00013070	K	270

IPI00013122	K	45
IPI00013122	K	78
IPI00013122	K	154
IPI00013122	K	240
IPI00013122	K	330
IPI00013146	K	211
IPI00013146	K	255
IPI00013146	K	258
IPI00013159	K	11
IPI00013159	K	302
IPI00013160	K	333
IPI00013160	K	337
IPI00013180	K	125
IPI00013184	K	136
IPI00013185	K	205
IPI00013205	K	602
IPI00013215	K	326
IPI00013290	K	39
IPI00013296	K	8
IPI00013296	K	78
IPI00013296	K	94
IPI00013296	K	106
IPI00013297	K	132
IPI00013396	K	52
IPI00013404	K	37
IPI00013404	K	45
IPI00013404	K	106
IPI00013404	K	428
IPI00013404	K	507
IPI00013415	K	70
IPI00013415	K	74
IPI00013415	K	169
IPI00013452	K	300
IPI00013452	K	417
IPI00013452	K	497
IPI00013452	K	498
IPI00013452	K	535
IPI00013452	K	542
IPI00013452	K	637
IPI00013452	K	788
IPI00013452	K	1156
IPI00013452	K	1503
IPI00013466	K	330
IPI00013468	K	179
IPI00013485	K	263
IPI00013485	K	275
IPI00013508	K	492
IPI00013683	K	58
IPI00013683	K	297
IPI00013721	K	358
IPI00013721	K	656
IPI00013721	K	717

IPI00013721	K	905
IPI00013721	K	907
IPI00013723	K	46
IPI00013743	K	47
IPI00013774	K	74
IPI00013774	K	89
IPI00013774	K	218
IPI00013774	K	220
IPI00013774	K	361
IPI00013774	K	432
IPI00013774	K	438
IPI00013774	K	439
IPI00013774	K	441
IPI00013808	K	114
IPI00013808	K	214
IPI00013808	K	217
IPI00013808	K	417
IPI00013808	K	592
IPI00013808	K	625
IPI00013809	K	32
IPI00013830	K	115
IPI00013830	K	213
IPI00013830	K	216
IPI00013830	K	217
IPI00013847	K	111
IPI00013847	K	138
IPI00013847	K	248
IPI00013862	K	169
IPI00013871	K	17
IPI00013871	K	376
IPI00013871	K	496
IPI00013881	K	167
IPI00013894	K	8
IPI00013894	K	68
IPI00013894	K	73
IPI00013894	K	100
IPI00013894	K	246
IPI00013894	K	301
IPI00013894	K	312
IPI00013894	K	325
IPI00013894	K	344
IPI00013894	K	388
IPI00013894	K	446
IPI00013895	K	3
IPI00013914	K	983
IPI00013949	K	137
IPI00013957	K	148
IPI00013957	K	244
IPI00013991	K	118
IPI00013999	K	245
IPI00013999	K	246
IPI00013999	K	312

IPI00013999	K	314
IPI00013999	K	315
IPI00013999	K	316
IPI00014068	K	455
IPI00014068	K	467
IPI00014149	K	255
IPI00014197	K	15
IPI00014198	K	116
IPI00014199	K	274
IPI00014213	K	236
IPI00014230	K	91
IPI00014230	K	95
IPI00014230	K	179
IPI00014256	K	751
IPI00014266	K	304
IPI00014305	K	135
IPI00014305	K	140
IPI00014311	K	412
IPI00014344	K	105
IPI00014361	K	147
IPI00014424	K	41
IPI00014424	K	44
IPI00014424	K	179
IPI00014424	K	439
IPI00014456	K	689
IPI00014474	K	538
IPI00014513	K	174
IPI00014533	K	61
IPI00014575	K	92
IPI00014575	K	105
IPI00014575	K	109
IPI00014577	K	138
IPI00014898	K	187
IPI00014898	K	2841
IPI00014898	K	3091
IPI00014898	K	3420
IPI00014898	K	4491
IPI00014903	K	7
IPI00014911	K	405
IPI00014911	K	1104
IPI00014925	K	376
IPI00014925	K	380
IPI00014925	K	382
IPI00014925	K	385
IPI00014925	K	386
IPI00014938	K	142
IPI00015018	K	57
IPI00015018	K	228
IPI00015029	K	7
IPI00015029	K	33
IPI00015077	K	58
IPI00015077	K	61

IPI00015105	K	4
IPI00015180	K	1341
IPI00015262	K	8
IPI00015262	K	25
IPI00015286	K	1602
IPI00015286	K	1605
IPI00015361	K	42
IPI00015361	K	112
IPI00015388	K	176
IPI00015602	K	185
IPI00015602	K	441
IPI00015602	K	470
IPI00015609	K	61
IPI00015671	K	401
IPI00015671	K	408
IPI00015811	K	124
IPI00015811	K	208
IPI00015811	K	209
IPI00015811	K	211
IPI00015811	K	213
IPI00015811	K	214
IPI00015826	K	265
IPI00015833	K	142
IPI00015842	K	86
IPI00015856	K	41
IPI00015864	K	684
IPI00015865	K	109
IPI00015905	K	165
IPI00015911	K	127
IPI00015911	K	143
IPI00015911	K	267
IPI00015911	K	320
IPI00015911	K	410
IPI00015911	K	417
IPI00015911	K	420
IPI00015934	K	369
IPI00015947	K	44
IPI00015947	K	46
IPI00015953	K	779
IPI00015972	K	60
IPI00015973	K	514
IPI00016007	K	864
IPI00016077	K	148
IPI00016284	K	537
IPI00016284	K	538
IPI00016287	K	281
IPI00016339	K	23
IPI00016405	K	96
IPI00016405	K	131
IPI00016457	K	261
IPI00016457	K	268
IPI00016458	K	104

IPI00016458	K	155
IPI00016513	K	102
IPI00016532	K	27
IPI00016532	K	186
IPI00016532	K	233
IPI00016559	K	68
IPI00016568	K	179
IPI00016568	K	186
IPI00016572	K	16
IPI00016589	K	23
IPI00016610	K	23
IPI00016613	K	102
IPI00016621	K	35
IPI00016637	K	174
IPI00016780	K	1426
IPI00016780	K	1432
IPI00016783	K	115
IPI00016801	K	84
IPI00016801	K	346
IPI00016801	K	415
IPI00016801	K	457
IPI00016801	K	480
IPI00016801	K	503
IPI00016801	K	527
IPI00016801	K	545
IPI00016862	K	110
IPI00016862	K	401
IPI00016910	K	558
IPI00017283	K	189
IPI00017283	K	233
IPI00017283	K	241
IPI00017283	K	661
IPI00017283	K	664
IPI00017283	K	775
IPI00017283	K	781
IPI00017292	K	19
IPI00017292	K	49
IPI00017292	K	345
IPI00017297	K	3
IPI00017297	K	146
IPI00017297	K	473
IPI00017297	K	522
IPI00017297	K	571
IPI00017297	K	836
IPI00017303	K	555
IPI00017303	K	635
IPI00017305	K	75
IPI00017334	K	202
IPI00017344	K	22
IPI00017376	K	320
IPI00017381	K	6
IPI00017381	K	13

IPI00017412	K	163
IPI00017412	K	304
IPI00017448	K	81
IPI00017450	K	407
IPI00017451	K	55
IPI00017451	K	251
IPI00017469	K	247
IPI00017558	K	219
IPI00017558	K	225
IPI00017592	K	597
IPI00017596	K	60
IPI00017596	K	66
IPI00017596	K	220
IPI00017617	K	32
IPI00017617	K	33
IPI00017617	K	40
IPI00017659	K	81
IPI00017672	K	95
IPI00017704	K	102
IPI00017704	K	110
IPI00017704	K	126
IPI00017799	K	183
IPI00017816	K	99
IPI00017816	K	101
IPI00017855	K	50
IPI00017855	K	520
IPI00017855	K	573
IPI00017855	K	605
IPI00017895	K	634
IPI00018027	K	664
IPI00018098	K	227
IPI00018120	K	175
IPI00018120	K	207
IPI00018140	K	100
IPI00018140	K	221
IPI00018140	K	363
IPI00018146	K	3
IPI00018146	K	9
IPI00018146	K	49
IPI00018146	K	68
IPI00018146	K	115
IPI00018146	K	120
IPI00018206	K	73
IPI00018206	K	90
IPI00018206	K	94
IPI00018206	K	150
IPI00018206	K	159
IPI00018206	K	185
IPI00018206	K	234
IPI00018206	K	296
IPI00018206	K	345
IPI00018206	K	363

IPI00018206	K	396
IPI00018206	K	404
IPI00018214	K	40
IPI00018214	K	66
IPI00018214	K	153
IPI00018214	K	154
IPI00018251	K	31
IPI00018251	K	636
IPI00018251	K	640
IPI00018251	K	780
IPI00018251	K	785
IPI00018251	K	788
IPI00018278	K	5
IPI00018278	K	8
IPI00018278	K	12
IPI00018278	K	14
IPI00018279	K	247
IPI00018279	K	249
IPI00018279	K	251
IPI00018279	K	254
IPI00018279	K	255
IPI00018349	K	220
IPI00018349	K	450
IPI00018349	K	627
IPI00018350	K	392
IPI00018350	K	396
IPI00018350	K	696
IPI00018352	K	195
IPI00018402	K	451
IPI00018402	K	463
IPI00018452	K	171
IPI00018465	K	67
IPI00018465	K	172
IPI00018465	K	217
IPI00018465	K	320
IPI00018465	K	366
IPI00018534	K	6
IPI00018534	K	12
IPI00018534	K	13
IPI00018534	K	16
IPI00018534	K	17
IPI00018534	K	21
IPI00018534	K	24
IPI00018534	K	109
IPI00018534	K	121
IPI00018627	K	34
IPI00018627	K	37
IPI00018627	K	140
IPI00018627	K	152
IPI00018627	K	155
IPI00018755	K	82
IPI00018755	K	157

IPI00018768	K	187
IPI00018768	K	199
IPI00018823	K	512
IPI00018824	K	152
IPI00018829	K	131
IPI00018842	K	33
IPI00018842	K	38
IPI00018842	K	90
IPI00018931	K	555
IPI00018931	K	556
IPI00018968	K	6
IPI00019004	K	110
IPI00019004	K	143
IPI00019046	K	212
IPI00019148	K	241
IPI00019226	K	481
IPI00019329	K	36
IPI00019353	K	6
IPI00019380	K	204
IPI00019380	K	698
IPI00019400	K	58
IPI00019400	K	77
IPI00019427	K	5
IPI00019450	K	224
IPI00019450	K	359
IPI00019472	K	537
IPI00019502	K	8
IPI00019502	K	74
IPI00019502	K	102
IPI00019502	K	299
IPI00019502	K	545
IPI00019502	K	821
IPI00019502	K	992
IPI00019502	K	1024
IPI00019502	K	1274
IPI00019502	K	1301
IPI00019502	K	1357
IPI00019502	K	1392
IPI00019502	K	1404
IPI00019502	K	1410
IPI00019502	K	1441
IPI00019502	K	1442
IPI00019502	K	1459
IPI00019502	K	1638
IPI00019502	K	1828
IPI00019548	K	19
IPI00019548	K	20
IPI00019548	K	39
IPI00019600	K	72
IPI00019755	K	57
IPI00019755	K	65
IPI00019755	K	66

IPI00019755	K	122
IPI00019755	K	143
IPI00019755	K	148
IPI00019755	K	152
IPI00019755	K	160
IPI00019770	K	125
IPI00019770	K	126
IPI00019812	K	40
IPI00019812	K	42
IPI00019848	K	288
IPI00019848	K	813
IPI00019848	K	836
IPI00019848	K	2005
IPI00019868	K	15
IPI00019868	K	102
IPI00019868	K	103
IPI00019868	K	105
IPI00019868	K	108
IPI00019868	K	112
IPI00019868	K	116
IPI00019869	K	27
IPI00019888	K	126
IPI00019888	K	135
IPI00019888	K	358
IPI00019912	K	46
IPI00019912	K	139
IPI00019912	K	140
IPI00019912	K	565
IPI00019912	K	663
IPI00019912	K	669
IPI00019912	K	707
IPI00019927	K	204
IPI00019927	K	214
IPI00019977	K	67
IPI00019981	K	52
IPI00019992	K	407
IPI00020008	K	48
IPI00020021	K	326
IPI00020039	K	286
IPI00020042	K	238
IPI00020042	K	397
IPI00020042	K	401
IPI00020042	K	418
IPI00020101	K	6
IPI00020101	K	12
IPI00020101	K	13
IPI00020101	K	16
IPI00020101	K	17
IPI00020101	K	21
IPI00020101	K	24
IPI00020101	K	25
IPI00020101	K	109

IPI00020127	K	163
IPI00020127	K	167
IPI00020127	K	259
IPI00020127	K	267
IPI00020416	K	708
IPI00020416	K	880
IPI00020416	K	883
IPI00020510	K	104
IPI00020546	K	406
IPI00020559	K	331
IPI00020567	K	80
IPI00020567	K	212
IPI00020599	K	48
IPI00020599	K	62
IPI00020599	K	64
IPI00020599	K	153
IPI00020599	K	159
IPI00020599	K	206
IPI00020599	K	207
IPI00020599	K	209
IPI00020599	K	238
IPI00020602	K	97
IPI00020618	K	6
IPI00020618	K	9
IPI00020618	K	13
IPI00020632	K	203
IPI00020898	K	81
IPI00020906	K	49
IPI00020956	K	44
IPI00020965	K	60
IPI00020965	K	64
IPI00020985	K	77
IPI00020985	K	79
IPI00020985	K	291
IPI00020985	K	292
IPI00020985	K	336
IPI00020985	K	350
IPI00020985	K	373
IPI00020985	K	386
IPI00020985	K	404
IPI00020985	K	418
IPI00020985	K	423
IPI00020985	K	489
IPI00020985	K	569
IPI00020985	K	601
IPI00020985	K	614
IPI00020985	K	636
IPI00020985	K	970
IPI00020985	K	977
IPI00020985	K	981
IPI00020985	K	1001
IPI00020985	K	1020

IPI00020985	K	1024
IPI00020985	K	1045
IPI00020985	K	1046
IPI00020985	K	1047
IPI00020985	K	1094
IPI00020985	K	1103
IPI00020985	K	1105
IPI00020985	K	1144
IPI00020985	K	1167
IPI00020985	K	1180
IPI00020985	K	1203
IPI00020985	K	1228
IPI00020985	K	1331
IPI00020985	K	1336
IPI00020985	K	1340
IPI00020985	K	1427
IPI00020985	K	1473
IPI00020985	K	1499
IPI00020985	K	1518
IPI00020985	K	1528
IPI00020985	K	1542
IPI00020985	K	1546
IPI00020985	K	1549
IPI00020985	K	1550
IPI00020985	K	1551
IPI00020985	K	1554
IPI00020985	K	1555
IPI00020985	K	1558
IPI00020985	K	1560
IPI00020985	K	1568
IPI00020985	K	1569
IPI00020985	K	1570
IPI00020985	K	1583
IPI00020985	K	1590
IPI00020985	K	1592
IPI00020985	K	1637
IPI00020985	K	1674
IPI00020985	K	1699
IPI00020985	K	1704
IPI00020985	K	1707
IPI00020985	K	1760
IPI00020985	K	1762
IPI00020985	K	1769
IPI00020985	K	1772
IPI00020985	K	1774
IPI00020985	K	1783
IPI00020985	K	1794
IPI00020985	K	1800
IPI00020985	K	1810
IPI00020985	K	1812
IPI00020985	K	2086
IPI00020985	K	2091

IPI00021048	K	884
IPI00021062	K	70
IPI00021062	K	78
IPI00021062	K	88
IPI00021062	K	145
IPI00021076	K	625
IPI00021088	K	124
IPI00021088	K	362
IPI00021129	K	592
IPI00021175	K	504
IPI00021187	K	2
IPI00021187	K	453
IPI00021263	K	3
IPI00021263	K	49
IPI00021263	K	68
IPI00021263	K	115
IPI00021263	K	120
IPI00021263	K	122
IPI00021266	K	70
IPI00021290	K	86
IPI00021290	K	546
IPI00021290	K	554
IPI00021290	K	948
IPI00021290	K	962
IPI00021290	K	968
IPI00021290	K	978
IPI00021290	K	1077
IPI00021327	K	6
IPI00021327	K	38
IPI00021327	K	50
IPI00021327	K	109
IPI00021338	K	466
IPI00021347	K	9
IPI00021347	K	131
IPI00021347	K	138
IPI00021369	K	92
IPI00021370	K	14
IPI00021405	K	97
IPI00021405	K	108
IPI00021405	K	114
IPI00021405	K	233
IPI00021405	K	260
IPI00021405	K	261
IPI00021405	K	270
IPI00021405	K	311
IPI00021405	K	316
IPI00021405	K	378
IPI00021405	K	417
IPI00021405	K	450
IPI00021405	K	470
IPI00021428	K	52
IPI00021428	K	63

IPI00021428	K	70
IPI00021428	K	193
IPI00021428	K	328
IPI00021428	K	330
IPI00021435	K	116
IPI00021435	K	407
IPI00021435	K	422
IPI00021439	K	50
IPI00021439	K	61
IPI00021439	K	326
IPI00021440	K	50
IPI00021440	K	61
IPI00021440	K	191
IPI00021440	K	326
IPI00021518	K	278
IPI00021570	K	123
IPI00021700	K	77
IPI00021700	K	80
IPI00021700	K	248
IPI00021700	K	254
IPI00021728	K	265
IPI00021728	K	293
IPI00021766	K	1104
IPI00021785	K	121
IPI00021805	K	42
IPI00021805	K	55
IPI00021808	K	443
IPI00021812	K	884
IPI00021828	K	78
IPI00021828	K	91
IPI00021831	K	367
IPI00021840	K	30
IPI00021840	K	203
IPI00021840	K	211
IPI00021885	K	620
IPI00021885	K	625
IPI00021924	K	47
IPI00021924	K	179
IPI00021924	K	180
IPI00021924	K	182
IPI00021924	K	185
IPI00021926	K	15
IPI00021926	K	20
IPI00021926	K	72
IPI00021926	K	206
IPI00021978	K	43
IPI00022018	K	197
IPI00022019	K	104
IPI00022055	K	416
IPI00022055	K	428
IPI00022055	K	430
IPI00022055	K	441

IPI00022055	K	442
IPI00022055	K	733
IPI00022145	K	9
IPI00022145	K	35
IPI00022145	K	52
IPI00022145	K	175
IPI00022145	K	184
IPI00022145	K	188
IPI00022145	K	196
IPI00022202	K	99
IPI00022202	K	209
IPI00022202	K	214
IPI00022215	K	1035
IPI00022215	K	1042
IPI00022229	K	2004
IPI00022264	K	247
IPI00022275	K	456
IPI00022276	K	133
IPI00022305	K	117
IPI00022314	K	68
IPI00022314	K	130
IPI00022314	K	132
IPI00022348	K	487
IPI00022348	K	515
IPI00022373	K	343
IPI00022421	K	69
IPI00022442	K	92
IPI00022443	K	413
IPI00022449	K	67
IPI00022449	K	304
IPI00022449	K	738
IPI00022449	K	1058
IPI00022465	K	1721
IPI00022498	K	51
IPI00022542	K	647
IPI00022542	K	718
IPI00022542	K	719
IPI00022597	K	3
IPI00022613	K	772
IPI00022630	K	11
IPI00022648	K	55
IPI00022744	K	158
IPI00022744	K	574
IPI00022744	K	824
IPI00022774	K	8
IPI00022774	K	18
IPI00022774	K	60
IPI00022774	K	81
IPI00022774	K	164
IPI00022774	K	251
IPI00022774	K	315
IPI00022774	K	336

IPI00022774	K	386
IPI00022774	K	505
IPI00022774	K	512
IPI00022774	K	524
IPI00022774	K	529
IPI00022774	K	614
IPI00022774	K	658
IPI00022774	K	663
IPI00022774	K	668
IPI00022774	K	677
IPI00022774	K	696
IPI00022774	K	754
IPI00022790	K	340
IPI00022793	K	72
IPI00022793	K	188
IPI00022793	K	201
IPI00022793	K	348
IPI00022820	K	238
IPI00022832	K	19
IPI00022865	K	54
IPI00022865	K	68
IPI00022865	K	95
IPI00022865	K	112
IPI00022881	K	450
IPI00022881	K	764
IPI00022881	K	1449
IPI00022891	K	92
IPI00022891	K	96
IPI00022891	K	272
IPI00023006	K	63
IPI00023006	K	70
IPI00023084	K	456
IPI00023087	K	191
IPI00023161	K	230
IPI00023161	K	265
IPI00023234	K	253
IPI00023234	K	271
IPI00023234	K	324
IPI00023234	K	617
IPI00023339	K	1014
IPI00023339	K	1203
IPI00023339	K	1216
IPI00023339	K	1535
IPI00023339	K	1583
IPI00023339	K	1586
IPI00023339	K	1587
IPI00023339	K	1588
IPI00023339	K	1591
IPI00023339	K	1592
IPI00023339	K	1595
IPI00023339	K	1597
IPI00023339	K	1711

IPI00023339	K	1741
IPI00023339	K	1744
IPI00023339	K	1797
IPI00023340	K	350
IPI00023340	K	355
IPI00023340	K	415
IPI00023340	K	604
IPI00023340	K	815
IPI00023340	K	1007
IPI00023340	K	1011
IPI00023343	K	765
IPI00023510	K	22
IPI00023529	K	43
IPI00023529	K	264
IPI00023530	K	56
IPI00023591	K	273
IPI00023598	K	103
IPI00023640	K	63
IPI00023647	K	544
IPI00023649	K	149
IPI00023649	K	413
IPI00023649	K	415
IPI00023649	K	1222
IPI00023756	K	378
IPI00023860	K	116
IPI00023860	K	194
IPI00023860	K	197
IPI00023860	K	264
IPI00023860	K	266
IPI00023860	K	271
IPI00023919	K	222
IPI00023972	K	448
IPI00023972	K	451
IPI00024067	K	450
IPI00024067	K	506
IPI00024067	K	507
IPI00024067	K	637
IPI00024067	K	764
IPI00024067	K	856
IPI00024067	K	1441
IPI00024067	K	1449
IPI00024067	K	1501
IPI00024071	K	80
IPI00024097	K	101
IPI00024157	K	160
IPI00024157	K	170
IPI00024157	K	200
IPI00024157	K	201
IPI00024163	K	445
IPI00024175	K	227
IPI00024214	K	169
IPI00024214	K	173

IPI00024214	K	447
IPI00024214	K	488
IPI00024279	K	1679
IPI00024282	K	3
IPI00024291	K	36
IPI00024305	K	19
IPI00024305	K	29
IPI00024305	K	378
IPI00024316	K	186
IPI00024316	K	189
IPI00024316	K	407
IPI00024387	K	238
IPI00024387	K	241
IPI00024387	K	265
IPI00024387	K	268
IPI00024387	K	271
IPI00024466	K	1034
IPI00024547	K	203
IPI00024568	K	1057
IPI00024623	K	284
IPI00024661	K	1087
IPI00024662	K	40
IPI00024662	K	55
IPI00024662	K	91
IPI00024662	K	106
IPI00024662	K	143
IPI00024664	K	184
IPI00024670	K	162
IPI00024670	K	164
IPI00024672	K	308
IPI00024787	K	291
IPI00024787	K	325
IPI00024871	K	11
IPI00024884	K	261
IPI00024913	K	164
IPI00024913	K	219
IPI00024915	K	83
IPI00024915	K	116
IPI00024919	K	91
IPI00024919	K	93
IPI00024933	K	40
IPI00024933	K	54
IPI00024933	K	83
IPI00024971	K	103
IPI00024975	K	797
IPI00024975	K	1009
IPI00024990	K	55
IPI00024990	K	117
IPI00024990	K	331
IPI00024993	K	101
IPI00024993	K	118
IPI00025019	K	204

IPI00025039	K	205
IPI00025084	K	179
IPI00025087	K	120
IPI00025087	K	164
IPI00025087	K	292
IPI00025087	K	305
IPI00025087	K	319
IPI00025087	K	320
IPI00025087	K	321
IPI00025087	K	370
IPI00025087	K	372
IPI00025087	K	373
IPI00025087	K	381
IPI00025087	K	382
IPI00025087	K	386
IPI00025091	K	38
IPI00025091	K	45
IPI00025091	K	48
IPI00025158	K	621
IPI00025176	K	219
IPI00025252	K	94
IPI00025252	K	104
IPI00025252	K	362
IPI00025273	K	350
IPI00025307	K	123
IPI00025307	K	283
IPI00025307	K	496
IPI00025329	K	80
IPI00025329	K	180
IPI00025341	K	132
IPI00025341	K	275
IPI00025346	K	34
IPI00025366	K	327
IPI00025366	K	366
IPI00025366	K	375
IPI00025366	K	382
IPI00025366	K	393
IPI00025366	K	459
IPI00025416	K	62
IPI00025416	K	327
IPI00025491	K	54
IPI00025491	K	118
IPI00025491	K	174
IPI00025491	K	291
IPI00025491	K	309
IPI00025512	K	123
IPI00025546	K	227
IPI00025710	K	283
IPI00025753	K	450
IPI00025807	K	551
IPI00025849	K	101
IPI00025874	K	226

IPI00025974	K	6
IPI00025974	K	114
IPI00025974	K	117
IPI00026087	K	64
IPI00026089	K	141
IPI00026089	K	333
IPI00026089	K	554
IPI00026089	K	562
IPI00026089	K	1067
IPI00026105	K	132
IPI00026105	K	142
IPI00026105	K	173
IPI00026105	K	183
IPI00026105	K	282
IPI00026105	K	341
IPI00026105	K	438
IPI00026105	K	453
IPI00026105	K	454
IPI00026105	K	470
IPI00026156	K	41
IPI00026156	K	123
IPI00026156	K	192
IPI00026156	K	241
IPI00026182	K	86
IPI00026182	K	97
IPI00026182	K	273
IPI00026185	K	235
IPI00026202	K	76
IPI00026215	K	80
IPI00026215	K	109
IPI00026215	K	200
IPI00026215	K	267
IPI00026215	K	354
IPI00026215	K	375
IPI00026215	K	377
IPI00026215	K	380
IPI00026216	K	48
IPI00026219	K	809
IPI00026230	K	167
IPI00026272	K	6
IPI00026272	K	96
IPI00026302	K	75
IPI00026309	K	27
IPI00026314	K	61
IPI00026314	K	648
IPI00026337	K	21
IPI00026337	K	23
IPI00026358	K	24
IPI00026516	K	173
IPI00026519	K	73
IPI00026519	K	167
IPI00026559	K	199

IPI00026665	K	327
IPI00026665	K	638
IPI00026689	K	6
IPI00026689	K	33
IPI00026689	K	34
IPI00026781	K	70
IPI00026781	K	213
IPI00026781	K	235
IPI00026781	K	298
IPI00026781	K	436
IPI00026781	K	528
IPI00026781	K	673
IPI00026781	K	786
IPI00026781	K	1239
IPI00026781	K	1704
IPI00026781	K	1752
IPI00026781	K	1771
IPI00026781	K	1847
IPI00026781	K	1878
IPI00026781	K	1995
IPI00026848	K	287
IPI00026928	K	106
IPI00026940	K	59
IPI00026940	K	83
IPI00026940	K	275
IPI00026940	K	276
IPI00026952	K	752
IPI00026970	K	139
IPI00026970	K	141
IPI00026970	K	196
IPI00026970	K	223
IPI00026970	K	335
IPI00026970	K	337
IPI00026970	K	513
IPI00026970	K	732
IPI00026970	K	786
IPI00026970	K	904
IPI00026970	K	907
IPI00027009	K	117
IPI00027032	K	190
IPI00027107	K	82
IPI00027107	K	91
IPI00027107	K	259
IPI00027107	K	421
IPI00027146	K	415
IPI00027146	K	457
IPI00027146	K	527
IPI00027146	K	545
IPI00027165	K	348
IPI00027223	K	81
IPI00027223	K	224
IPI00027223	K	321

IPI00027228	K	181
IPI00027230	K	75
IPI00027230	K	613
IPI00027230	K	682
IPI00027232	K	1088
IPI00027252	K	250
IPI00027269	K	197
IPI00027270	K	77
IPI00027280	K	365
IPI00027280	K	367
IPI00027280	K	373
IPI00027280	K	992
IPI00027415	K	845
IPI00027415	K	853
IPI00027415	K	947
IPI00027442	K	19
IPI00027442	K	876
IPI00027443	K	586
IPI00027444	K	137
IPI00027444	K	177
IPI00027445	K	419
IPI00027445	K	444
IPI00027448	K	24
IPI00027448	K	54
IPI00027448	K	66
IPI00027451	K	365
IPI00027451	K	371
IPI00027463	K	40
IPI00027497	K	12
IPI00027497	K	142
IPI00027497	K	211
IPI00027497	K	252
IPI00027497	K	466
IPI00027547	K	68
IPI00027547	K	85
IPI00027569	K	39
IPI00027626	K	5
IPI00027626	K	10
IPI00027626	K	199
IPI00027626	K	365
IPI00027626	K	377
IPI00027626	K	388
IPI00027681	K	39
IPI00027681	K	43
IPI00027729	K	242
IPI00027776	K	415
IPI00027834	K	59
IPI00027834	K	269
IPI00027834	K	475
IPI00027834	K	552
IPI00027970	K	55
IPI00027987	K	53

IPI00027987	K	64
IPI00027987	K	67
IPI00027988	K	18
IPI00027988	K	20
IPI00027996	K	472
IPI00028004	K	77
IPI00028006	K	68
IPI00028006	K	185
IPI00028031	K	285
IPI00028031	K	377
IPI00028051	K	761
IPI00028051	K	762
IPI00028091	K	240
IPI00028091	K	244
IPI00028091	K	251
IPI00028091	K	254
IPI00028109	K	35
IPI00028127	K	2
IPI00028127	K	8
IPI00028127	K	15
IPI00028127	K	18
IPI00028127	K	305
IPI00028160	K	87
IPI00028277	K	216
IPI00028414	K	35
IPI00028414	K	119
IPI00028520	K	81
IPI00028520	K	104
IPI00028520	K	375
IPI00028635	K	460
IPI00028888	K	165
IPI00028888	K	197
IPI00028888	K	251
IPI00028888	K	341
IPI00028888	K	353
IPI00028912	K	362
IPI00028957	K	393
IPI00029012	K	68
IPI00029073	K	65
IPI00029073	K	69
IPI00029073	K	76
IPI00029073	K	77
IPI00029079	K	9
IPI00029079	K	416
IPI00029081	K	316
IPI00029095	K	3
IPI00029133	K	115
IPI00029133	K	131
IPI00029133	K	162
IPI00029133	K	221
IPI00029133	K	233
IPI00029133	K	238

IPI00029196	K	384
IPI00029250	K	113
IPI00029400	K	54
IPI00029444	K	267
IPI00029447	K	124
IPI00029447	K	126
IPI00029484	K	439
IPI00029484	K	645
IPI00029485	K	230
IPI00029534	K	81
IPI00029557	K	157
IPI00029557	K	215
IPI00029558	K	114
IPI00029561	K	181
IPI00029561	K	242
IPI00029561	K	243
IPI00029561	K	350
IPI00029601	K	87
IPI00029601	K	124
IPI00029601	K	144
IPI00029601	K	161
IPI00029601	K	198
IPI00029601	K	203
IPI00029601	K	218
IPI00029601	K	235
IPI00029601	K	272
IPI00029601	K	304
IPI00029601	K	309
IPI00029601	K	319
IPI00029623	K	102
IPI00029623	K	104
IPI00029623	K	181
IPI00029629	K	273
IPI00029629	K	320
IPI00029629	K	567
IPI00029631	K	12
IPI00029697	K	297
IPI00029705	K	827
IPI00029731	K	8
IPI00029733	K	246
IPI00029737	K	89
IPI00029737	K	92
IPI00029741	K	206
IPI00029744	K	103
IPI00029744	K	104
IPI00029744	K	113
IPI00029745	K	276
IPI00029750	K	37
IPI00029764	K	92
IPI00029764	K	219
IPI00029764	K	489
IPI00029772	K	384

IPI00029795	K	280
IPI00029997	K	180
IPI00030090	K	449
IPI00030131	K	207
IPI00030179	K	29
IPI00030179	K	31
IPI00030179	K	124
IPI00030179	K	161
IPI00030247	K	390
IPI00030247	K	492
IPI00030274	K	499
IPI00030275	K	87
IPI00030275	K	332
IPI00030275	K	382
IPI00030275	K	424
IPI00030275	K	431
IPI00030275	K	432
IPI00030275	K	466
IPI00030320	K	73
IPI00030363	K	83
IPI00030363	K	124
IPI00030363	K	174
IPI00030363	K	181
IPI00030363	K	190
IPI00030363	K	202
IPI00030363	K	230
IPI00030363	K	251
IPI00030363	K	263
IPI00030363	K	338
IPI00030702	K	343
IPI00030706	K	3
IPI00030706	K	212
IPI00030770	K	264
IPI00030781	K	410
IPI00030781	K	413
IPI00030820	K	144
IPI00030915	K	601
IPI00031023	K	21
IPI00031410	K	1218
IPI00031420	K	107
IPI00031420	K	173
IPI00031420	K	434
IPI00031489	K	58
IPI00031489	K	61
IPI00031517	K	599
IPI00031517	K	643
IPI00031517	K	646
IPI00031517	K	775
IPI00031519	K	235
IPI00031519	K	239
IPI00031519	K	1173
IPI00031519	K	1175

IPI00031519	K	1177
IPI00031519	K	1179
IPI00031519	K	1181
IPI00031519	K	1183
IPI00031521	K	20
IPI00031522	K	129
IPI00031522	K	132
IPI00031522	K	295
IPI00031522	K	303
IPI00031522	K	309
IPI00031522	K	326
IPI00031522	K	350
IPI00031522	K	353
IPI00031522	K	359
IPI00031522	K	406
IPI00031522	K	460
IPI00031522	K	505
IPI00031522	K	540
IPI00031522	K	569
IPI00031522	K	570
IPI00031522	K	644
IPI00031522	K	728
IPI00031526	K	118
IPI00031526	K	146
IPI00031545	K	608
IPI00031556	K	462
IPI00031562	K	6
IPI00031562	K	10
IPI00031562	K	96
IPI00031563	K	65
IPI00031570	K	21
IPI00031570	K	165
IPI00031583	K	206
IPI00031627	K	710
IPI00031681	K	6
IPI00031691	K	121
IPI00031801	K	96
IPI00031812	K	64
IPI00031812	K	81
IPI00031820	K	311
IPI00031820	K	349
IPI00032103	K	385
IPI00032164	K	69
IPI00032214	K	368
IPI00032214	K	418
IPI00032214	K	516
IPI00032214	K	519
IPI00032214	K	523
IPI00032214	K	903
IPI00032230	K	405
IPI00032313	K	7
IPI00032313	K	18

IPI00032313	K	22
IPI00032313	K	35
IPI00032316	K	528
IPI00032355	K	830
IPI00032358	K	714
IPI00032358	K	717
IPI00032401	K	89
IPI00032423	K	15
IPI00032561	K	297
IPI00032598	K	388
IPI00032826	K	153
IPI00032827	K	29
IPI00032827	K	41
IPI00032831	K	124
IPI00032849	K	90
IPI00032875	K	96
IPI00032875	K	153
IPI00032875	K	223
IPI00032875	K	344
IPI00032881	K	102
IPI00032892	K	25
IPI00032904	K	6
IPI00032904	K	12
IPI00032955	K	102
IPI00032955	K	112
IPI00032957	K	65
IPI00033016	K	158
IPI00033016	K	163
IPI00033016	K	172
IPI00033016	K	290
IPI00033016	K	332
IPI00033016	K	338
IPI00033016	K	386
IPI00033022	K	598
IPI00033030	K	21
IPI00033036	K	427
IPI00033054	K	26
IPI00033054	K	57
IPI00033217	K	707
IPI00033486	K	288
IPI00033487	K	11
IPI00033561	K	218
IPI00033561	K	221
IPI00034308	K	884
IPI00036578	K	1014
IPI00036742	K	166
IPI00043429	K	165
IPI00044461	K	183
IPI00044587	K	226
IPI00044666	K	376
IPI00044761	K	131
IPI00045109	K	6

IPI00045550	K	774
IPI00045914	K	1050
IPI00045914	K	1998
IPI00045914	K	1999
IPI00045914	K	2001
IPI00045914	K	2005
IPI00045922	K	373
IPI00045946	K	237
IPI00046828	K	83
IPI00046828	K	100
IPI00056505	K	248
IPI00059242	K	70
IPI00059242	K	191
IPI00059279	K	9
IPI00059292	K	116
IPI00060181	K	233
IPI00060473	K	484
IPI00061009	K	403
IPI00061009	K	413
IPI00061206	K	111
IPI00061525	K	152
IPI00061651	K	29
IPI00061680	K	288
IPI00061680	K	291
IPI00062037	K	5
IPI00063106	K	3
IPI00063106	K	6
IPI00063106	K	11
IPI00063130	K	149
IPI00063302	K	189
IPI00063302	K	191
IPI00063404	K	474
IPI00063404	K	475
IPI00063635	K	292
IPI00063635	K	572
IPI00063673	K	127
IPI00063903	K	16
IPI00063903	K	17
IPI00064162	K	408
IPI00064202	K	599
IPI00064212	K	490
IPI00064212	K	617
IPI00064457	K	928
IPI00064765	K	208
IPI00064767	K	295
IPI00065500	K	283
IPI00068506	K	304
IPI00069084	K	2543
IPI00069084	K	3078
IPI00069750	K	251
IPI00069750	K	454
IPI00069817	K	416

IPI00069817	K	426
IPI00069817	K	1335
IPI00070943	K	930
IPI00071483	K	432
IPI00072377	K	83
IPI00072377	K	132
IPI00072377	K	150
IPI00072377	K	172
IPI00072534	K	70
IPI00072534	K	483
IPI00072534	K	487
IPI00074876	K	843
IPI00074893	K	834
IPI00074893	K	835
IPI00074893	K	836
IPI00074893	K	843
IPI00075248	K	14
IPI00075248	K	22
IPI00075248	K	76
IPI00075248	K	116
IPI00075248	K	149
IPI00081097	K	163
IPI00081836	K	6
IPI00083708	K	27
IPI00083708	K	49
IPI00083708	K	394
IPI00084571	K	126
IPI00084571	K	204
IPI00084571	K	234
IPI00084571	K	236
IPI00093057	K	371
IPI00093057	K	404
IPI00093253	K	343
IPI00096066	K	73
IPI00096066	K	227
IPI00096066	K	291
IPI00096066	K	338
IPI00097532	K	176
IPI00098902	K	970
IPI00099179	K	303
IPI00099433	K	440
IPI00099433	K	584
IPI00099433	K	590
IPI00099433	K	1038
IPI00099433	K	1042
IPI00099433	K	1044
IPI00099463	K	353
IPI00099810	K	144
IPI00099834	K	222
IPI00099834	K	261
IPI00100151	K	796
IPI00100160	K	55

IPI00100160	K	971
IPI00100160	K	1168
IPI00100460	K	235
IPI00100460	K	382
IPI00100460	K	627
IPI00100630	K	252
IPI00100630	K	333
IPI00100656	K	22
IPI00100656	K	116
IPI00100731	K	69
IPI00100980	K	324
IPI00101664	K	353
IPI00101968	K	176
IPI00101968	K	297
IPI00102069	K	213
IPI00102069	K	254
IPI00102165	K	6
IPI00102165	K	96
IPI00102339	K	58
IPI00102339	K	99
IPI00102339	K	102
IPI00102339	K	104
IPI00102580	K	219
IPI00102580	K	229
IPI00102661	K	305
IPI00102752	K	450
IPI00102804	K	229
IPI00102804	K	230
IPI00102804	K	234
IPI00102815	K	23
IPI00102815	K	34
IPI00102815	K	40
IPI00103023	K	298
IPI00103142	K	157
IPI00103415	K	701
IPI00103467	K	364
IPI00103483	K	519
IPI00103525	K	206
IPI00103599	K	229
IPI00103654	K	252
IPI00103867	K	105
IPI00104050	K	221
IPI00104050	K	401
IPI00104050	K	420
IPI00104050	K	421
IPI00104050	K	455
IPI00104050	K	519
IPI00104050	K	709
IPI00104050	K	811
IPI00104341	K	43
IPI00104907	K	113
IPI00105407	K	27

IPI00105407	K	69
IPI00105407	K	95
IPI00105407	K	98
IPI00105407	K	125
IPI00105407	K	179
IPI00105407	K	263
IPI00105598	K	418
IPI00106567	K	46
IPI00107366	K	58
IPI00107722	K	114
IPI00107745	K	78
IPI00107745	K	166
IPI00107745	K	231
IPI00107745	K	233
IPI00107752	K	265
IPI00107752	K	809
IPI00107753	K	283
IPI00140420	K	193
IPI00140420	K	339
IPI00140420	K	641
IPI00140420	K	752
IPI00143753	K	98
IPI00143753	K	105
IPI00143753	K	106
IPI00143753	K	760
IPI00146935	K	283
IPI00147874	K	79
IPI00147874	K	290
IPI00147874	K	293
IPI00149276	K	270
IPI00149849	K	447
IPI00150269	K	27
IPI00151977	K	611
IPI00152182	K	9
IPI00152182	K	10
IPI00152216	K	202
IPI00152535	K	100
IPI00152535	K	102
IPI00152535	K	106
IPI00152535	K	107
IPI00152535	K	174
IPI00152535	K	175
IPI00152535	K	858
IPI00152542	K	1049
IPI00152653	K	310
IPI00152653	K	320
IPI00152653	K	322
IPI00152688	K	419
IPI00152692	K	65
IPI00152695	K	98
IPI00152695	K	181
IPI00152785	K	12

IPI00152785	K	13
IPI00152785	K	16
IPI00152785	K	17
IPI00152785	K	21
IPI00152785	K	24
IPI00152785	K	109
IPI00152906	K	6
IPI00152906	K	12
IPI00152906	K	13
IPI00152906	K	16
IPI00152906	K	21
IPI00152906	K	24
IPI00152906	K	109
IPI00152906	K	117
IPI00152906	K	121
IPI00152981	K	41
IPI00152981	K	608
IPI00154451	K	517
IPI00154915	K	1005
IPI00154915	K	1007
IPI00155601	K	103
IPI00156649	K	477
IPI00157734	K	39
IPI00159322	K	1267
IPI00160622	K	1048
IPI00162330	K	127
IPI00162563	K	20
IPI00163084	K	420
IPI00163496	K	801
IPI00163496	K	804
IPI00163505	K	103
IPI00163851	K	1259
IPI00164352	K	1117
IPI00165092	K	355
IPI00165189	K	290
IPI00165230	K	103
IPI00165931	K	1350
IPI00166055	K	21
IPI00166153	K	108
IPI00166293	K	6
IPI00166293	K	12
IPI00166293	K	13
IPI00166395	K	439
IPI00166500	K	114
IPI00166500	K	125
IPI00166528	K	582
IPI00166555	K	511
IPI00166704	K	46
IPI00166704	K	47
IPI00166708	K	73
IPI00166749	K	299
IPI00166865	K	131

IPI00167196	K	54
IPI00167419	K	171
IPI00167535	K	346
IPI00167535	K	1472
IPI00167535	K	1553
IPI00167535	K	1634
IPI00167535	K	2349
IPI00167535	K	2356
IPI00167904	K	103
IPI00167904	K	111
IPI00167941	K	1683
IPI00168378	K	194
IPI00168388	K	452
IPI00168603	K	496
IPI00168839	K	153
IPI00168899	K	135
IPI00169267	K	314
IPI00169267	K	321
IPI00169383	K	11
IPI00169383	K	30
IPI00169383	K	48
IPI00169383	K	75
IPI00169383	K	86
IPI00169383	K	91
IPI00169383	K	97
IPI00169383	K	131
IPI00169383	K	146
IPI00169383	K	156
IPI00169383	K	199
IPI00169383	K	267
IPI00169383	K	291
IPI00169383	K	322
IPI00169383	K	323
IPI00169383	K	406
IPI00170596	K	469
IPI00170596	K	723
IPI00170596	K	727
IPI00170596	K	875
IPI00170596	K	934
IPI00170786	K	13
IPI00170867	K	512
IPI00171127	K	126
IPI00171390	K	23
IPI00171445	K	114
IPI00171573	K	332
IPI00171611	K	10
IPI00171611	K	15
IPI00171611	K	19
IPI00171611	K	24
IPI00171611	K	57
IPI00171611	K	80
IPI00171692	K	87

IPI00171779	K	260
IPI00171779	K	262
IPI00171798	K	152
IPI00171821	K	391
IPI00171844	K	25
IPI00171903	K	239
IPI00171903	K	277
IPI00171903	K	698
IPI00172487	K	101
IPI00172656	K	167
IPI00173359	K	562
IPI00174345	K	156
IPI00174345	K	159
IPI00174775	K	142
IPI00175151	K	53
IPI00176157	K	538
IPI00176706	K	21
IPI00176903	K	109
IPI00177437	K	381
IPI00177728	K	9
IPI00177888	K	363
IPI00177888	K	474
IPI00177965	K	171
IPI00178150	K	868
IPI00178366	K	961
IPI00178366	K	963
IPI00178431	K	193
IPI00178431	K	206
IPI00178431	K	514
IPI00178431	K	522
IPI00178440	K	7
IPI00178440	K	60
IPI00178440	K	185
IPI00178512	K	792
IPI00178611	K	191
IPI00178611	K	304
IPI00178744	K	95
IPI00178744	K	263
IPI00178744	K	300
IPI00178744	K	355
IPI00178744	K	506
IPI00178750	K	134
IPI00178750	K	135
IPI00178750	K	139
IPI00178750	K	144
IPI00178750	K	146
IPI00179026	K	51
IPI00179298	K	509
IPI00179298	K	1840
IPI00179298	K	2248
IPI00179330	K	6
IPI00179330	K	11

IPI00179330	K	48
IPI00179330	K	104
IPI00179330	K	107
IPI00179330	K	113
IPI00179330	K	152
IPI00179709	K	40
IPI00179709	K	163
IPI00179709	K	280
IPI00179709	K	394
IPI00179709	K	401
IPI00179851	K	123
IPI00180240	K	4
IPI00180240	K	32
IPI00180240	K	39
IPI00180675	K	40
IPI00180675	K	164
IPI00180675	K	326
IPI00180675	K	401
IPI00180764	K	171
IPI00180764	K	199
IPI00181702	K	49
IPI00181728	K	276
IPI00182116	K	118
IPI00182289	K	48
IPI00182304	K	396
IPI00182533	K	101
IPI00182632	K	274
IPI00182728	K	432
IPI00182728	K	434
IPI00182757	K	215
IPI00182757	K	287
IPI00183208	K	194
IPI00183208	K	197
IPI00183500	K	151
IPI00183530	K	237
IPI00183626	K	259
IPI00183626	K	266
IPI00183626	K	515
IPI00183626	K	554
IPI00183695	K	23
IPI00183695	K	28
IPI00183695	K	37
IPI00183695	K	54
IPI00183695	K	57
IPI00183968	K	13
IPI00183968	K	119
IPI00184284	K	54
IPI00184330	K	216
IPI00184330	K	896
IPI00184533	K	245
IPI00185027	K	1158
IPI00185361	K	444

IPI00185361	K	517
IPI00185361	K	519
IPI00185361	K	523
IPI00185374	K	221
IPI00185374	K	368
IPI00185374	K	448
IPI00186224	K	141
IPI00186224	K	144
IPI00186290	K	235
IPI00186290	K	239
IPI00186290	K	272
IPI00186290	K	275
IPI00186290	K	308
IPI00186290	K	318
IPI00186290	K	426
IPI00186290	K	445
IPI00186290	K	857
IPI00215611	K	9
IPI00215611	K	22
IPI00215637	K	118
IPI00215637	K	130
IPI00215719	K	154
IPI00215780	K	23
IPI00215780	K	77
IPI00215780	K	111
IPI00215790	K	9
IPI00215790	K	67
IPI00215790	K	70
IPI00215878	K	21
IPI00215893	K	39
IPI00215901	K	14
IPI00215901	K	93
IPI00215911	K	3
IPI00215911	K	6
IPI00215911	K	197
IPI00215914	K	36
IPI00215963	K	496
IPI00215963	K	739
IPI00215965	K	3
IPI00215965	K	52
IPI00215965	K	113
IPI00215965	K	144
IPI00215965	K	350
IPI00215999	K	225
IPI00216008	K	89
IPI00216008	K	171
IPI00216008	K	432
IPI00216008	K	449
IPI00216008	K	478
IPI00216008	K	543
IPI00216008	K	560
IPI00216047	K	326

IPI00216048	K	215
IPI00216092	K	6
IPI00216092	K	160
IPI00216132	K	304
IPI00216137	K	111
IPI00216138	K	17
IPI00216164	K	219
IPI00216164	K	346
IPI00216164	K	584
IPI00216199	K	293
IPI00216199	K	305
IPI00216199	K	347
IPI00216230	K	656
IPI00216237	K	62
IPI00216293	K	14
IPI00216293	K	136
IPI00216298	K	3
IPI00216298	K	39
IPI00216298	K	94
IPI00216308	K	20
IPI00216308	K	28
IPI00216308	K	61
IPI00216308	K	109
IPI00216308	K	224
IPI00216308	K	266
IPI00216318	K	70
IPI00216318	K	117
IPI00216319	K	120
IPI00216383	K	261
IPI00216402	K	10
IPI00216402	K	15
IPI00216402	K	19
IPI00216402	K	24
IPI00216402	K	28
IPI00216402	K	80
IPI00216456	K	6
IPI00216456	K	10
IPI00216456	K	119
IPI00216457	K	6
IPI00216457	K	10
IPI00216457	K	96
IPI00216457	K	119
IPI00216457	K	120
IPI00216470	K	150
IPI00216654	K	33
IPI00216654	K	425
IPI00216654	K	673
IPI00216691	K	38
IPI00216691	K	105
IPI00216691	K	108
IPI00216691	K	126
IPI00216694	K	335

IPI00216704	K	1258
IPI00216704	K	1262
IPI00216730	K	6
IPI00216737	K	20
IPI00216873	K	99
IPI00216932	K	396
IPI00216932	K	642
IPI00216951	K	74
IPI00216951	K	374
IPI00216975	K	112
IPI00216975	K	118
IPI00216975	K	161
IPI00216975	K	213
IPI00216975	K	220
IPI00216975	K	251
IPI00217013	K	655
IPI00217018	K	1259
IPI00217030	K	22
IPI00217030	K	120
IPI00217030	K	122
IPI00217030	K	134
IPI00217049	K	378
IPI00217049	K	433
IPI00217223	K	79
IPI00217223	K	273
IPI00217223	K	309
IPI00217223	K	311
IPI00217236	K	28
IPI00217240	K	466
IPI00217259	K	511
IPI00217259	K	515
IPI00217324	K	482
IPI00217354	K	231
IPI00217354	K	273
IPI00217413	K	87
IPI00217413	K	93
IPI00217465	K	63
IPI00217466	K	17
IPI00217466	K	64
IPI00217467	K	17
IPI00217467	K	46
IPI00217467	K	63
IPI00217467	K	90
IPI00217467	K	136
IPI00217467	K	168
IPI00217468	K	109
IPI00217468	K	168
IPI00217468	K	199
IPI00217468	K	202
IPI00217468	K	204
IPI00217468	K	207
IPI00217469	K	88

IPI00217469	K	93
IPI00217471	K	60
IPI00217477	K	12
IPI00217477	K	59
IPI00217477	K	145
IPI00217536	K	92
IPI00217660	K	113
IPI00217660	K	238
IPI00217686	K	20
IPI00217686	K	233
IPI00217686	K	234
IPI00217732	K	538
IPI00217773	K	701
IPI00217773	K	725
IPI00217773	K	726
IPI00217773	K	738
IPI00217801	K	533
IPI00217871	K	99
IPI00217871	K	114
IPI00217871	K	402
IPI00217872	K	34
IPI00217872	K	437
IPI00217872	K	475
IPI00217920	K	50
IPI00217949	K	68
IPI00217950	K	3
IPI00217950	K	5
IPI00217950	K	11
IPI00217950	K	54
IPI00217950	K	56
IPI00217950	K	59
IPI00217950	K	82
IPI00217950	K	90
IPI00217963	K	162
IPI00217963	K	169
IPI00217975	K	33
IPI00217975	K	123
IPI00217975	K	124
IPI00217975	K	157
IPI00217975	K	181
IPI00217975	K	182
IPI00217975	K	271
IPI00217975	K	483
IPI00218054	K	20
IPI00218087	K	220
IPI00218087	K	222
IPI00218087	K	224
IPI00218094	K	1272
IPI00218130	K	316
IPI00218187	K	147
IPI00218240	K	779
IPI00218240	K	781

IPI00218342	K	56
IPI00218342	K	553
IPI00218343	K	40
IPI00218343	K	60
IPI00218343	K	112
IPI00218343	K	163
IPI00218343	K	164
IPI00218343	K	280
IPI00218343	K	311
IPI00218343	K	370
IPI00218343	K	394
IPI00218343	K	401
IPI00218371	K	176
IPI00218371	K	194
IPI00218448	K	5
IPI00218448	K	8
IPI00218448	K	12
IPI00218448	K	14
IPI00218448	K	116
IPI00218465	K	529
IPI00218488	K	31
IPI00218568	K	41
IPI00218570	K	100
IPI00218591	K	38
IPI00218591	K	179
IPI00218606	K	135
IPI00218624	K	16
IPI00218624	K	288
IPI00218624	K	2095
IPI00218624	K	2103
IPI00218693	K	114
IPI00218728	K	53
IPI00218733	K	71
IPI00218733	K	123
IPI00218748	K	76
IPI00218748	K	120
IPI00218748	K	121
IPI00218823	K	926
IPI00218823	K	2296
IPI00218829	K	108
IPI00218829	K	196
IPI00218829	K	469
IPI00218829	K	490
IPI00218829	K	493
IPI00218831	K	126
IPI00218836	K	25
IPI00218836	K	36
IPI00218836	K	72
IPI00218836	K	94
IPI00218847	K	156
IPI00218914	K	65
IPI00218914	K	91

IPI00218914	K	128
IPI00218914	K	252
IPI00218914	K	353
IPI00218914	K	367
IPI00218914	K	410
IPI00218914	K	412
IPI00218914	K	419
IPI00218914	K	435
IPI00218914	K	495
IPI00218918	K	53
IPI00218918	K	161
IPI00218918	K	239
IPI00218918	K	242
IPI00218918	K	250
IPI00218918	K	281
IPI00218918	K	312
IPI00218922	K	527
IPI00218971	K	202
IPI00219005	K	274
IPI00219005	K	282
IPI00219018	K	61
IPI00219018	K	117
IPI00219018	K	186
IPI00219018	K	194
IPI00219018	K	259
IPI00219018	K	334
IPI00219037	K	6
IPI00219037	K	10
IPI00219037	K	119
IPI00219038	K	10
IPI00219038	K	15
IPI00219038	K	19
IPI00219038	K	24
IPI00219038	K	28
IPI00219038	K	37
IPI00219038	K	38
IPI00219038	K	57
IPI00219038	K	80
IPI00219068	K	233
IPI00219072	K	44
IPI00219072	K	48
IPI00219072	K	96
IPI00219072	K	97
IPI00219072	K	296
IPI00219072	K	311
IPI00219072	K	416
IPI00219072	K	423
IPI00219072	K	431
IPI00219077	K	73
IPI00219077	K	337
IPI00219077	K	414
IPI00219077	K	573

IPI00219078	K	464
IPI00219097	K	3
IPI00219097	K	12
IPI00219097	K	30
IPI00219097	K	43
IPI00219097	K	44
IPI00219097	K	59
IPI00219097	K	139
IPI00219097	K	157
IPI00219097	K	172
IPI00219097	K	173
IPI00219097	K	182
IPI00219155	K	27
IPI00219155	K	93
IPI00219155	K	98
IPI00219155	K	117
IPI00219155	K	128
IPI00219156	K	26
IPI00219160	K	36
IPI00219160	K	37
IPI00219173	K	482
IPI00219179	K	289
IPI00219217	K	7
IPI00219217	K	58
IPI00219217	K	60
IPI00219217	K	82
IPI00219217	K	119
IPI00219217	K	318
IPI00219217	K	319
IPI00219217	K	329
IPI00219219	K	29
IPI00219299	K	1543
IPI00219306	K	114
IPI00219365	K	35
IPI00219365	K	63
IPI00219365	K	64
IPI00219365	K	79
IPI00219365	K	139
IPI00219365	K	209
IPI00219365	K	211
IPI00219365	K	253
IPI00219365	K	254
IPI00219365	K	258
IPI00219365	K	263
IPI00219365	K	344
IPI00219365	K	388
IPI00219368	K	343
IPI00219381	K	64
IPI00219383	K	23
IPI00219383	K	34
IPI00219420	K	105
IPI00219420	K	106

IPI00219420	K	114
IPI00219420	K	140
IPI00219420	K	215
IPI00219420	K	336
IPI00219420	K	1190
IPI00219445	K	208
IPI00219446	K	80
IPI00219446	K	132
IPI00219497	K	592
IPI00219497	K	601
IPI00219525	K	38
IPI00219525	K	59
IPI00219525	K	76
IPI00219525	K	154
IPI00219525	K	309
IPI00219532	K	100
IPI00219532	K	105
IPI00219538	K	72
IPI00219553	K	674
IPI00219563	K	972
IPI00219563	K	976
IPI00219563	K	1074
IPI00219568	K	91
IPI00219568	K	156
IPI00219568	K	267
IPI00219575	K	391
IPI00219601	K	35
IPI00219616	K	194
IPI00219622	K	70
IPI00219622	K	171
IPI00219673	K	71
IPI00219673	K	169
IPI00219678	K	141
IPI00219691	K	166
IPI00219691	K	168
IPI00219691	K	180
IPI00219691	K	225
IPI00219691	K	226
IPI00219695	K	38
IPI00219695	K	312
IPI00219729	K	73
IPI00219757	K	121
IPI00219757	K	128
IPI00219757	K	191
IPI00219757	K	209
IPI00219894	K	22
IPI00219913	K	291
IPI00219913	K	313
IPI00219913	K	449
IPI00219919	K	28
IPI00219919	K	35
IPI00219919	K	36

IPI00219919	K	37
IPI00219953	K	87
IPI00220014	K	233
IPI00220219	K	318
IPI00220219	K	627
IPI00220289	K	186
IPI00220289	K	198
IPI00220301	K	63
IPI00220301	K	209
IPI00220360	K	8
IPI00220360	K	32
IPI00220360	K	36
IPI00220362	K	56
IPI00220362	K	70
IPI00220362	K	86
IPI00220362	K	99
IPI00220373	K	308
IPI00220373	K	425
IPI00220373	K	427
IPI00220403	K	6
IPI00220403	K	12
IPI00220403	K	13
IPI00220403	K	16
IPI00220403	K	17
IPI00220403	K	21
IPI00220403	K	24
IPI00220403	K	47
IPI00220403	K	109
IPI00220403	K	121
IPI00220416	K	12
IPI00220416	K	78
IPI00220477	K	136
IPI00220477	K	143
IPI00220484	K	82
IPI00220486	K	418
IPI00220487	K	63
IPI00220487	K	72
IPI00220487	K	73
IPI00220487	K	78
IPI00220487	K	85
IPI00220487	K	95
IPI00220487	K	99
IPI00220487	K	117
IPI00220487	K	148
IPI00220487	K	149
IPI00220566	K	153
IPI00220637	K	323
IPI00220642	K	120
IPI00220656	K	295
IPI00220710	K	103
IPI00220710	K	157
IPI00220710	K	250

IPI00220710	K	407
IPI00220726	K	215
IPI00220766	K	148
IPI00220827	K	4
IPI00220827	K	15
IPI00220827	K	17
IPI00220827	K	26
IPI00220827	K	39
IPI00220828	K	4
IPI00220828	K	12
IPI00220828	K	17
IPI00220828	K	26
IPI00220828	K	32
IPI00220828	K	39
IPI00220834	K	144
IPI00220834	K	155
IPI00220834	K	156
IPI00220834	K	195
IPI00220834	K	265
IPI00220834	K	332
IPI00220834	K	338
IPI00220834	K	532
IPI00220834	K	660
IPI00220834	K	665
IPI00220871	K	10
IPI00220901	K	477
IPI00220906	K	104
IPI00220994	K	12
IPI00221088	K	155
IPI00221089	K	27
IPI00221089	K	39
IPI00221089	K	93
IPI00221089	K	94
IPI00221091	K	124
IPI00221092	K	60
IPI00221093	K	19
IPI00221106	K	275
IPI00221222	K	35
IPI00221222	K	68
IPI00221226	K	63
IPI00221226	K	68
IPI00221226	K	75
IPI00221226	K	81
IPI00221226	K	299
IPI00221226	K	306
IPI00221226	K	370
IPI00221226	K	418
IPI00221226	K	442
IPI00221226	K	445
IPI00221226	K	483
IPI00221226	K	607
IPI00221226	K	620

IPI00221300	K	35
IPI00221325	K	115
IPI00221325	K	123
IPI00221394	K	472
IPI00221394	K	474
IPI00221394	K	475
IPI00221394	K	477
IPI00232533	K	3
IPI00232533	K	5
IPI00232533	K	7
IPI00234252	K	345
IPI00234252	K	346
IPI00234252	K	354
IPI00234252	K	359
IPI00234252	K	948
IPI00234446	K	357
IPI00234446	K	374
IPI00239077	K	21
IPI00239077	K	30
IPI00239406	K	955
IPI00240812	K	417
IPI00240812	K	1136
IPI00241860	K	126
IPI00245135	K	264
IPI00245135	K	295
IPI00246058	K	48
IPI00246058	K	215
IPI00246188	K	47
IPI00250153	K	116
IPI00251559	K	350
IPI00251749	K	481
IPI00251749	K	493
IPI00251749	K	495
IPI00251749	K	496
IPI00255316	K	6
IPI00256605	K	302
IPI00256861	K	90
IPI00256861	K	4252
IPI00257882	K	493
IPI00288947	K	102
IPI00289034	K	397
IPI00289034	K	398
IPI00289034	K	401
IPI00289092	K	4
IPI00289159	K	311
IPI00289271	K	828
IPI00289271	K	831
IPI00289334	K	677
IPI00289334	K	681
IPI00289334	K	2170
IPI00289334	K	2524
IPI00289334	K	2576

IPI00289344	K	1336
IPI00289344	K	1412
IPI00289499	K	199
IPI00289499	K	356
IPI00289499	K	357
IPI00289524	K	75
IPI00289524	K	246
IPI00289524	K	270
IPI00289601	K	169
IPI00289601	K	184
IPI00289746	K	256
IPI00289758	K	7
IPI00289758	K	10
IPI00289773	K	264
IPI00289773	K	265
IPI00289776	K	1790
IPI00289776	K	2422
IPI00289807	K	402
IPI00289819	K	2352
IPI00289862	K	306
IPI00289866	K	245
IPI00289866	K	248
IPI00289866	K	262
IPI00289866	K	265
IPI00290094	K	315
IPI00290142	K	100
IPI00290184	K	250
IPI00290204	K	118
IPI00290204	K	162
IPI00290314	K	633
IPI00290416	K	216
IPI00290416	K	242
IPI00290416	K	294
IPI00290416	K	297
IPI00290460	K	78
IPI00290547	K	1228
IPI00290548	K	119
IPI00290548	K	122
IPI00290548	K	127
IPI00290566	K	199
IPI00290566	K	400
IPI00290566	K	532
IPI00290652	K	1050
IPI00290652	K	1061
IPI00290652	K	1339
IPI00290857	K	208
IPI00290952	K	652
IPI00291006	K	91
IPI00291006	K	157
IPI00291006	K	165
IPI00291006	K	185
IPI00291006	K	215

IPI00291006	K	239
IPI00291006	K	296
IPI00291006	K	297
IPI00291006	K	301
IPI00291006	K	314
IPI00291006	K	329
IPI00291006	K	335
IPI00291006	K	338
IPI00291016	K	223
IPI00291131	K	36
IPI00291136	K	121
IPI00291416	K	440
IPI00291419	K	229
IPI00291419	K	262
IPI00291419	K	264
IPI00291467	K	23
IPI00291467	K	92
IPI00291467	K	96
IPI00291467	K	105
IPI00291467	K	268
IPI00291467	K	272
IPI00291483	K	246
IPI00291510	K	436
IPI00291510	K	511
IPI00291608	K	64
IPI00291608	K	67
IPI00291608	K	70
IPI00291608	K	150
IPI00291608	K	152
IPI00291643	K	95
IPI00291643	K	130
IPI00291646	K	174
IPI00291646	K	189
IPI00291669	K	71
IPI00291669	K	117
IPI00291669	K	245
IPI00291669	K	246
IPI00291669	K	306
IPI00291751	K	180
IPI00291751	K	182
IPI00291764	K	6
IPI00291764	K	10
IPI00291764	K	119
IPI00291783	K	1363
IPI00291800	K	486
IPI00291916	K	1497
IPI00291916	K	1533
IPI00291930	K	643
IPI00291939	K	282
IPI00291939	K	437
IPI00291939	K	536
IPI00291939	K	648

IPI00291939	K	713
IPI00292012	K	614
IPI00292012	K	620
IPI00292025	K	161
IPI00292026	K	120
IPI00292059	K	384
IPI00292059	K	718
IPI00292059	K	954
IPI00292059	K	1120
IPI00292135	K	55
IPI00292135	K	190
IPI00292135	K	594
IPI00292135	K	601
IPI00292140	K	82
IPI00292238	K	128
IPI00292445	K	63
IPI00292537	K	432
IPI00292537	K	441
IPI00292537	K	442
IPI00292746	K	249
IPI00292753	K	238
IPI00292763	K	495
IPI00292771	K	379
IPI00292771	K	891
IPI00292771	K	1511
IPI00292771	K	2070
IPI00292771	K	2071
IPI00292812	K	271
IPI00292812	K	274
IPI00292812	K	276
IPI00293009	K	282
IPI00293026	K	528
IPI00293126	K	219
IPI00293276	K	78
IPI00293331	K	40
IPI00293331	K	46
IPI00293350	K	68
IPI00293350	K	252
IPI00293426	K	188
IPI00293426	K	455
IPI00293464	K	153
IPI00293464	K	383
IPI00293464	K	1067
IPI00293523	K	209
IPI00293523	K	213
IPI00293523	K	218
IPI00293564	K	48
IPI00293564	K	93
IPI00293568	K	123
IPI00293613	K	584
IPI00293613	K	587
IPI00293655	K	239

IPI00293655	K	268
IPI00293655	K	281
IPI00293657	K	29
IPI00293657	K	31
IPI00293657	K	32
IPI00293657	K	75
IPI00293657	K	78
IPI00293746	K	53
IPI00293845	K	1274
IPI00293921	K	581
IPI00294084	K	385
IPI00294084	K	685
IPI00294084	K	741
IPI00294158	K	248
IPI00294158	K	253
IPI00294158	K	269
IPI00294159	K	97
IPI00294159	K	160
IPI00294159	K	255
IPI00294186	K	264
IPI00294186	K	342
IPI00294186	K	379
IPI00294186	K	380
IPI00294186	K	459
IPI00294211	K	260
IPI00294211	K	521
IPI00294211	K	522
IPI00294495	K	122
IPI00294603	K	1282
IPI00294742	K	325
IPI00294742	K	581
IPI00294744	K	220
IPI00294744	K	499
IPI00294744	K	566
IPI00294779	K	20
IPI00294779	K	28
IPI00294779	K	62
IPI00294779	K	64
IPI00294779	K	91
IPI00294840	K	747
IPI00294842	K	134
IPI00294842	K	136
IPI00294879	K	26
IPI00294879	K	524
IPI00294879	K	528
IPI00294943	K	142
IPI00294943	K	144
IPI00294982	K	266
IPI00294982	K	268
IPI00294982	K	299
IPI00294982	K	302
IPI00294982	K	303

IPI00295004	K	533
IPI00295209	K	275
IPI00295363	K	46
IPI00295363	K	88
IPI00295363	K	231
IPI00295363	K	238
IPI00295400	K	366
IPI00295485	K	272
IPI00296022	K	42
IPI00296022	K	85
IPI00296053	K	66
IPI00296053	K	80
IPI00296053	K	94
IPI00296053	K	115
IPI00296053	K	256
IPI00296053	K	292
IPI00296053	K	477
IPI00296069	K	867
IPI00296183	K	3
IPI00296183	K	178
IPI00296183	K	194
IPI00296183	K	269
IPI00296337	K	117
IPI00296337	K	254
IPI00296337	K	828
IPI00296337	K	832
IPI00296337	K	1057
IPI00296337	K	1074
IPI00296337	K	1209
IPI00296337	K	1970
IPI00296337	K	2259
IPI00296337	K	2702
IPI00296337	K	2703
IPI00296337	K	3241
IPI00296337	K	3260
IPI00296337	K	3608
IPI00296337	K	3621
IPI00296337	K	3638
IPI00296337	K	3642
IPI00296388	K	680
IPI00296441	K	54
IPI00296441	K	232
IPI00296441	K	312
IPI00296635	K	68
IPI00296830	K	297
IPI00296907	K	89
IPI00296907	K	255
IPI00296907	K	267
IPI00296907	K	272
IPI00296907	K	437
IPI00296907	K	500
IPI00296907	K	504

IPI00296907	K	643
IPI00296907	K	651
IPI00296913	K	42
IPI00296913	K	210
IPI00296913	K	218
IPI00296934	K	112
IPI00297145	K	85
IPI00297211	K	440
IPI00297254	K	1448
IPI00297333	K	288
IPI00297333	K	312
IPI00297333	K	314
IPI00297333	K	319
IPI00297455	K	257
IPI00297477	K	172
IPI00297477	K	179
IPI00297550	K	74
IPI00297550	K	222
IPI00297550	K	678
IPI00297572	K	576
IPI00297572	K	1052
IPI00297579	K	10
IPI00297579	K	21
IPI00297579	K	34
IPI00297579	K	44
IPI00297579	K	50
IPI00297579	K	143
IPI00297779	K	13
IPI00297779	K	154
IPI00297779	K	181
IPI00297851	K	1338
IPI00297851	K	1346
IPI00297921	K	238
IPI00297931	K	513
IPI00297931	K	744
IPI00298007	K	7
IPI00298007	K	50
IPI00298007	K	414
IPI00298041	K	105
IPI00298058	K	328
IPI00298202	K	52
IPI00298202	K	318
IPI00298301	K	1877
IPI00298301	K	1880
IPI00298308	K	886
IPI00298308	K	903
IPI00298406	K	238
IPI00298406	K	244
IPI00298406	K	261
IPI00298406	K	317
IPI00298406	K	388
IPI00298423	K	194

IPI00298520	K	274
IPI00298547	K	148
IPI00298558	K	179
IPI00298612	K	184
IPI00298612	K	192
IPI00298612	K	233
IPI00298612	K	245
IPI00298731	K	207
IPI00298731	K	239
IPI00298731	K	274
IPI00298860	K	321
IPI00298935	K	361
IPI00298946	K	131
IPI00298961	K	446
IPI00298961	K	455
IPI00298961	K	568
IPI00298961	K	686
IPI00298961	K	693
IPI00298994	K	2031
IPI00298994	K	2043
IPI00298994	K	2115
IPI00299048	K	1467
IPI00299048	K	1471
IPI00299048	K	1474
IPI00299095	K	447
IPI00299095	K	469
IPI00299145	K	173
IPI00299147	K	11
IPI00299147	K	41
IPI00299147	K	44
IPI00299149	K	11
IPI00299149	K	42
IPI00299149	K	45
IPI00299155	K	127
IPI00299155	K	176
IPI00299155	K	180
IPI00299155	K	238
IPI00299214	K	220
IPI00299254	K	784
IPI00299254	K	923
IPI00299263	K	223
IPI00299263	K	228
IPI00299263	K	229
IPI00299313	K	158
IPI00299402	K	1090
IPI00299417	K	24
IPI00299463	K	208
IPI00299463	K	233
IPI00299463	K	334
IPI00299463	K	441
IPI00299463	K	504
IPI00299463	K	544

IPI00299463	K	665
IPI00299463	K	690
IPI00299463	K	698
IPI00299463	K	715
IPI00299465	K	259
IPI00299465	K	263
IPI00299507	K	637
IPI00299507	K	640
IPI00299512	K	1581
IPI00299571	K	154
IPI00299571	K	293
IPI00299573	K	34
IPI00299573	K	48
IPI00299573	K	97
IPI00299573	K	150
IPI00299573	K	217
IPI00299608	K	310
IPI00299749	K	487
IPI00300026	K	197
IPI00300027	K	106
IPI00300052	K	175
IPI00300052	K	182
IPI00300060	K	452
IPI00300060	K	520
IPI00300074	K	329
IPI00300078	K	700
IPI00300127	K	426
IPI00300186	K	360
IPI00300341	K	32
IPI00300371	K	109
IPI00300371	K	126
IPI00300371	K	936
IPI00300386	K	453
IPI00300567	K	89
IPI00300567	K	283
IPI00300631	K	294
IPI00300631	K	475
IPI00300631	K	607
IPI00300631	K	805
IPI00300725	K	173
IPI00301058	K	283
IPI00301154	K	213
IPI00301154	K	259
IPI00301224	K	236
IPI00301263	K	747
IPI00301263	K	1411
IPI00301323	K	7
IPI00301323	K	458
IPI00301323	K	460
IPI00301323	K	571
IPI00301364	K	163
IPI00301434	K	113

IPI00301518	K	149
IPI00301609	K	731
IPI00301844	K	193
IPI00301994	K	203
IPI00301994	K	234
IPI00302238	K	383
IPI00302742	K	133
IPI00302829	K	427
IPI00302829	K	548
IPI00302829	K	640
IPI00302829	K	652
IPI00302829	K	873
IPI00302829	K	874
IPI00302829	K	896
IPI00302927	K	139
IPI00302927	K	288
IPI00302927	K	302
IPI00302927	K	319
IPI00302927	K	326
IPI00302927	K	489
IPI00303133	K	6
IPI00303133	K	12
IPI00303133	K	13
IPI00303133	K	16
IPI00303133	K	21
IPI00303133	K	24
IPI00303133	K	109
IPI00303133	K	121
IPI00303135	K	244
IPI00303135	K	247
IPI00303135	K	250
IPI00303152	K	1401
IPI00303152	K	1404
IPI00303152	K	1410
IPI00303160	K	262
IPI00303174	K	98
IPI00303207	K	121
IPI00303207	K	191
IPI00303207	K	210
IPI00303207	K	349
IPI00303207	K	431
IPI00303402	K	112
IPI00303402	K	183
IPI00303476	K	133
IPI00303476	K	198
IPI00303476	K	259
IPI00303476	K	426
IPI00303476	K	485
IPI00303476	K	522
IPI00303797	K	418
IPI00303832	K	413
IPI00303882	K	65

IPI00303954	K	34
IPI00304071	K	296
IPI00304306	K	93
IPI00304409	K	68
IPI00304417	K	154
IPI00304417	K	201
IPI00304417	K	207
IPI00304417	K	382
IPI00304431	K	258
IPI00304435	K	51
IPI00304435	K	146
IPI00304493	K	423
IPI00304527	K	418
IPI00304596	K	5
IPI00304596	K	11
IPI00304596	K	64
IPI00304596	K	68
IPI00304596	K	96
IPI00304596	K	107
IPI00304596	K	109
IPI00304596	K	198
IPI00304596	K	467
IPI00304612	K	25
IPI00304612	K	191
IPI00304692	K	30
IPI00304740	K	472
IPI00304740	K	475
IPI00304740	K	495
IPI00304814	K	47
IPI00304925	K	71
IPI00304925	K	88
IPI00304925	K	108
IPI00304925	K	112
IPI00304925	K	246
IPI00304925	K	319
IPI00304925	K	348
IPI00304925	K	500
IPI00305068	K	585
IPI00305144	K	217
IPI00305166	K	179
IPI00305166	K	335
IPI00305166	K	485
IPI00305166	K	498
IPI00305166	K	538
IPI00305166	K	541
IPI00305166	K	547
IPI00305227	K	7
IPI00305227	K	16
IPI00305227	K	18
IPI00305289	K	146
IPI00305360	K	217
IPI00305374	K	133

IPI00305374	K	300
IPI00305383	K	159
IPI00305383	K	250
IPI00305545	K	128
IPI00306043	K	245
IPI00306043	K	401
IPI00306048	K	135
IPI00306048	K	427
IPI00306280	K	59
IPI00306290	K	634
IPI00306301	K	115
IPI00306301	K	121
IPI00306301	K	359
IPI00306301	K	374
IPI00306311	K	64
IPI00306332	K	27
IPI00306332	K	77
IPI00306332	K	93
IPI00306369	K	586
IPI00306380	K	79
IPI00306430	K	33
IPI00306430	K	116
IPI00306642	K	201
IPI00306667	K	175
IPI00306708	K	64
IPI00306708	K	65
IPI00306723	K	695
IPI00306749	K	793
IPI00306959	K	101
IPI00306959	K	199
IPI00306959	K	296
IPI00306960	K	244
IPI00306960	K	490
IPI00307092	K	116
IPI00307092	K	169
IPI00307092	K	430
IPI00307162	K	173
IPI00307162	K	496
IPI00307162	K	1070
IPI00307246	K	289
IPI00307259	K	84
IPI00307430	K	1072
IPI00307659	K	240
IPI00307755	K	69
IPI00328156	K	52
IPI00328161	K	377
IPI00328257	K	318
IPI00328258	K	239
IPI00328293	K	140
IPI00328306	K	764
IPI00328319	K	4
IPI00328319	K	22

IPI00328361	K	110
IPI00328391	K	270
IPI00328409	K	891
IPI00328415	K	42
IPI00328415	K	120
IPI00328526	K	58
IPI00328798	K	36
IPI00328798	K	108
IPI00328798	K	110
IPI00328798	K	112
IPI00328798	K	137
IPI00328815	K	968
IPI00328847	K	14
IPI00328868	K	337
IPI00328918	K	31
IPI00328918	K	33
IPI00328918	K	35
IPI00328929	K	814
IPI00328987	K	15
IPI00329132	K	233
IPI00329142	K	12
IPI00329213	K	371
IPI00329331	K	438
IPI00329338	K	8
IPI00329389	K	5
IPI00329389	K	210
IPI00329389	K	239
IPI00329512	K	8
IPI00329512	K	32
IPI00329512	K	36
IPI00329528	K	701
IPI00329633	K	243
IPI00329633	K	306
IPI00329633	K	660
IPI00329665	K	6
IPI00329665	K	12
IPI00329665	K	13
IPI00329665	K	16
IPI00329665	K	17
IPI00329665	K	21
IPI00329665	K	24
IPI00329665	K	25
IPI00329665	K	35
IPI00329665	K	47
IPI00329665	K	86
IPI00329665	K	109
IPI00329665	K	121
IPI00329692	K	55
IPI00329742	K	203
IPI00329742	K	234
IPI00329755	K	380
IPI00329791	K	263

IPI00329791	K	271
IPI00329791	K	776
IPI00329791	K	779
IPI00329791	K	848
IPI00329801	K	70
IPI00329801	K	76
IPI00329801	K	79
IPI00329801	K	97
IPI00329801	K	101
IPI00332376	K	8
IPI00332376	K	144
IPI00333010	K	18
IPI00333010	K	239
IPI00333010	K	879
IPI00333010	K	901
IPI00333143	K	484
IPI00333201	K	84
IPI00333215	K	84
IPI00333533	K	631
IPI00333533	K	633
IPI00333533	K	634
IPI00333541	K	504
IPI00333541	K	508
IPI00333541	K	578
IPI00333541	K	700
IPI00333541	K	704
IPI00333541	K	781
IPI00333541	K	837
IPI00333541	K	891
IPI00333541	K	973
IPI00333541	K	994
IPI00333541	K	1071
IPI00333541	K	1538
IPI00333541	K	2024
IPI00333541	K	2513
IPI00333541	K	2515
IPI00333541	K	2607
IPI00333541	K	2621
IPI00333770	K	834
IPI00333770	K	1593
IPI00333913	K	1057
IPI00334159	K	95
IPI00334190	K	145
IPI00334190	K	233
IPI00334587	K	318
IPI00334587	K	330
IPI00334715	K	193
IPI00335001	K	81
IPI00335385	K	138
IPI00335385	K	142
IPI00335421	K	339
IPI00335541	K	299

IPI00336008	K	126
IPI00336008	K	371
IPI00336016	K	329
IPI00336081	K	608
IPI00336081	K	1744
IPI00336081	K	1747
IPI00337315	K	7
IPI00337315	K	1571
IPI00337386	K	233
IPI00337386	K	717
IPI00337386	K	907
IPI00337386	K	910
IPI00337386	K	911
IPI00337397	K	586
IPI00337494	K	336
IPI00337494	K	437
IPI00337541	K	70
IPI00337541	K	100
IPI00337541	K	331
IPI00337541	K	394
IPI00337541	K	397
IPI00339269	K	321
IPI00339269	K	502
IPI00339274	K	6
IPI00339384	K	112
IPI00373877	K	247
IPI00374657	K	125
IPI00374975	K	5
IPI00374975	K	100
IPI00374975	K	106
IPI00374975	K	113
IPI00375127	K	291
IPI00375339	K	45
IPI00375358	K	634
IPI00375380	K	300
IPI00375531	K	56
IPI00375531	K	81
IPI00375676	K	98
IPI00375731	K	448
IPI00376005	K	57
IPI00376005	K	77
IPI00376005	K	97
IPI00376005	K	98
IPI00376119	K	314
IPI00376317	K	125
IPI00376379	K	181
IPI00376798	K	52
IPI00376798	K	85
IPI00376798	K	159
IPI00376976	K	182
IPI00376976	K	184
IPI00377245	K	1974

IPI00377245	K	2196
IPI00377245	K	2883
IPI00377245	K	3161
IPI00377245	K	4193
IPI00377245	K	4244
IPI00377245	K	4504
IPI00377245	K	5004
IPI00382470	K	191
IPI00382470	K	222
IPI00382470	K	346
IPI00382470	K	414
IPI00382470	K	416
IPI00382470	K	436
IPI00382470	K	449
IPI00382470	K	484
IPI00382470	K	529
IPI00382470	K	532
IPI00382470	K	558
IPI00382470	K	565
IPI00382470	K	580
IPI00382470	K	600
IPI00382470	K	611
IPI00382470	K	668
IPI00382470	K	680
IPI00382470	K	695
IPI00382470	K	698
IPI00382470	K	707
IPI00382470	K	753
IPI00383046	K	36
IPI00383105	K	490
IPI00383105	K	499
IPI00383163	K	81
IPI00384028	K	657
IPI00384028	K	666
IPI00384028	K	752
IPI00384028	K	756
IPI00384047	K	98
IPI00384202	K	552
IPI00384233	K	43
IPI00384428	K	126
IPI00384428	K	257
IPI00384456	K	70
IPI00384456	K	504
IPI00384471	K	368
IPI00384471	K	418
IPI00384471	K	516
IPI00384471	K	519
IPI00384471	K	521
IPI00384471	K	523
IPI00384471	K	887
IPI00384471	K	1034
IPI00384643	K	321

IPI00384707	K	486
IPI00384857	K	8
IPI00384872	K	350
IPI00384972	K	104
IPI00384972	K	180
IPI00385042	K	103
IPI00385042	K	522
IPI00385055	K	890
IPI00385267	K	163
IPI00385267	K	169
IPI00385267	K	170
IPI00385449	K	570
IPI00385449	K	604
IPI00385449	K	628
IPI00385631	K	2667
IPI00386122	K	140
IPI00386189	K	34
IPI00386189	K	262
IPI00386189	K	735
IPI00386189	K	756
IPI00386448	K	122
IPI00386448	K	123
IPI00386448	K	218
IPI00386448	K	221
IPI00386448	K	310
IPI00386448	K	314
IPI00386448	K	315
IPI00386803	K	23
IPI00386803	K	36
IPI00386803	K	42
IPI00386803	K	128
IPI00386930	K	262
IPI00386930	K	384
IPI00386998	K	36
IPI00387159	K	164
IPI00387159	K	165
IPI00387159	K	181
IPI00387159	K	264
IPI00394657	K	85
IPI00394926	K	286
IPI00394926	K	287
IPI00394926	K	298
IPI00394926	K	302
IPI00395337	K	294
IPI00395627	K	8
IPI00395627	K	19
IPI00395627	K	85
IPI00395627	K	118
IPI00395627	K	134
IPI00395865	K	4
IPI00395865	K	21
IPI00395865	K	119

IPI00395939	K	217
IPI00396008	K	582
IPI00396015	K	1371
IPI00396015	K	1617
IPI00396015	K	2068
IPI00396048	K	245
IPI00396089	K	85
IPI00396154	K	124
IPI00396154	K	296
IPI00396174	K	23
IPI00396258	K	321
IPI00396258	K	377
IPI00396276	K	108
IPI00396276	K	117
IPI00396286	K	133
IPI00396321	K	111
IPI00396329	K	47
IPI00396329	K	56
IPI00396341	K	848
IPI00396341	K	859
IPI00396378	K	3
IPI00396378	K	112
IPI00396378	K	168
IPI00396378	K	173
IPI00396435	K	17
IPI00396435	K	18
IPI00396435	K	132
IPI00396435	K	488
IPI00396435	K	754
IPI00396435	K	760
IPI00396485	K	41
IPI00396485	K	44
IPI00396485	K	55
IPI00396485	K	79
IPI00396485	K	146
IPI00396485	K	172
IPI00396485	K	179
IPI00396485	K	180
IPI00396485	K	255
IPI00396485	K	318
IPI00396485	K	386
IPI00396485	K	392
IPI00396485	K	395
IPI00396485	K	439
IPI00396485	K	443
IPI00396552	K	279
IPI00396577	K	1619
IPI00396577	K	1830
IPI00396627	K	152
IPI00397358	K	70
IPI00397366	K	309
IPI00397366	K	310

IPI00397366	K	322
IPI00397376	K	357
IPI00397383	K	7
IPI00397571	K	253
IPI00397740	K	466
IPI00397860	K	19
IPI00397860	K	24
IPI00398406	K	4
IPI00398406	K	249
IPI00398406	K	270
IPI00398406	K	282
IPI00398406	K	403
IPI00398749	K	672
IPI00399266	K	168
IPI00401962	K	48
IPI00402077	K	50
IPI00402231	K	41
IPI00402231	K	56
IPI00402759	K	20
IPI00409671	K	5
IPI00409671	K	25
IPI00409675	K	143
IPI00409698	K	862
IPI00409717	K	293
IPI00409750	K	232
IPI00410039	K	235
IPI00410039	K	240
IPI00410256	K	780
IPI00410287	K	40
IPI00410287	K	42
IPI00410287	K	80
IPI00410351	K	1352
IPI00410351	K	1428
IPI00410402	K	40
IPI00410402	K	60
IPI00410402	K	326
IPI00410496	K	102
IPI00410496	K	117
IPI00410590	K	291
IPI00410616	K	50
IPI00410616	K	154
IPI00410618	K	108
IPI00410693	K	68
IPI00410693	K	122
IPI00410693	K	140
IPI00410693	K	211
IPI00410714	K	17
IPI00410717	K	801
IPI00411303	K	342
IPI00411303	K	345
IPI00411303	K	348
IPI00411356	K	8

IPI00411559	K	363
IPI00411559	K	381
IPI00411559	K	679
IPI00411559	K	1037
IPI00411614	K	962
IPI00411614	K	1127
IPI00411706	K	10
IPI00411706	K	39
IPI00411706	K	200
IPI00411706	K	278
IPI00411937	K	505
IPI00411937	K	507
IPI00412344	K	585
IPI00412368	K	136
IPI00412368	K	138
IPI00412368	K	156
IPI00412368	K	157
IPI00412404	K	99
IPI00412404	K	220
IPI00412415	K	973
IPI00412441	K	253
IPI00412441	K	689
IPI00412579	K	62
IPI00412579	K	106
IPI00412579	K	118
IPI00412607	K	13
IPI00412607	K	14
IPI00412607	K	19
IPI00412607	K	43
IPI00412672	K	454
IPI00412741	K	197
IPI00413022	K	482
IPI00413108	K	52
IPI00413265	K	548
IPI00413324	K	74
IPI00413344	K	19
IPI00413344	K	92
IPI00413344	K	96
IPI00413365	K	1275
IPI00413611	K	172
IPI00413611	K	280
IPI00413611	K	712
IPI00413627	K	367
IPI00413627	K	406
IPI00413627	K	409
IPI00413641	K	86
IPI00413641	K	95
IPI00413641	K	222
IPI00413641	K	263
IPI00413730	K	418
IPI00413778	K	90
IPI00413880	K	1426

Table S1

IPI00414127	K	150
IPI00414127	K	183
IPI00414320	K	348
IPI00414320	K	355
IPI00414320	K	579
IPI00414463	K	204
IPI00414629	K	24
IPI00414676	K	275
IPI00414676	K	284
IPI00414676	K	306
IPI00414676	K	354
IPI00414676	K	399
IPI00414676	K	402
IPI00414676	K	435
IPI00414676	K	481
IPI00414676	K	568
IPI00414676	K	573
IPI00414676	K	623
IPI00414676	K	624
IPI00414717	K	747
IPI00414872	K	698
IPI00414973	K	507
IPI00418169	K	122
IPI00418169	K	133
IPI00418169	K	166
IPI00418169	K	170
IPI00418169	K	175
IPI00418169	K	194
IPI00418169	K	245
IPI00418169	K	297
IPI00418169	K	299
IPI00418169	K	320
IPI00418169	K	331
IPI00418174	K	40
IPI00418213	K	329
IPI00418213	K	348
IPI00418238	K	7
IPI00418238	K	173
IPI00418262	K	234
IPI00418313	K	81
IPI00418313	K	100
IPI00418313	K	214
IPI00418313	K	460
IPI00418316	K	440
IPI00418471	K	104
IPI00418471	K	120
IPI00418471	K	139
IPI00418471	K	235
IPI00418471	K	236
IPI00418471	K	292
IPI00418471	K	373
IPI00418471	K	445

IPI00419235	K	137
IPI00419237	K	221
IPI00419249	K	57
IPI00419249	K	110
IPI00419249	K	206
IPI00419249	K	230
IPI00419249	K	238
IPI00419258	K	3
IPI00419258	K	12
IPI00419258	K	29
IPI00419258	K	30
IPI00419258	K	43
IPI00419258	K	44
IPI00419258	K	59
IPI00419258	K	82
IPI00419258	K	146
IPI00419258	K	147
IPI00419258	K	154
IPI00419258	K	157
IPI00419273	K	555
IPI00419273	K	641
IPI00419373	K	29
IPI00419373	K	134
IPI00419373	K	148
IPI00419373	K	151
IPI00419541	K	404
IPI00419585	K	28
IPI00419585	K	44
IPI00419585	K	49
IPI00419585	K	76
IPI00419585	K	82
IPI00419585	K	118
IPI00419585	K	125
IPI00419585	K	131
IPI00419643	K	221
IPI00419802	K	92
IPI00419802	K	353
IPI00419833	K	6
IPI00419833	K	12
IPI00419833	K	13
IPI00419833	K	16
IPI00419833	K	21
IPI00419833	K	109
IPI00419844	K	428
IPI00419880	K	27
IPI00419880	K	34
IPI00419880	K	144
IPI00419880	K	145
IPI00419880	K	249
IPI00419884	K	10
IPI00419884	K	15
IPI00419919	K	33

IPI00419919	K	82
IPI00419928	K	110
IPI00419979	K	38
IPI00419979	K	128
IPI00419979	K	502
IPI00420014	K	469
IPI00420014	K	971
IPI00420014	K	1176
IPI00420065	K	378
IPI00420065	K	385
IPI00420071	K	396
IPI00420071	K	398
IPI00420108	K	267
IPI00420108	K	272
IPI00428484	K	153
IPI00430411	K	190
IPI00430472	K	572
IPI00430803	K	134
IPI00430803	K	135
IPI00430812	K	103
IPI00433834	K	77
IPI00436632	K	1247
IPI00436748	K	208
IPI00438229	K	266
IPI00438229	K	304
IPI00438229	K	340
IPI00438229	K	377
IPI00438229	K	770
IPI00438229	K	774
IPI00438701	K	422
IPI00439194	K	4
IPI00439194	K	109
IPI00439194	K	141
IPI00439548	K	392
IPI00440484	K	1096
IPI00440493	K	132
IPI00440493	K	161
IPI00440493	K	230
IPI00440493	K	239
IPI00440493	K	240
IPI00440493	K	261
IPI00440493	K	305
IPI00440493	K	427
IPI00440493	K	434
IPI00440493	K	498
IPI00440493	K	506
IPI00440493	K	531
IPI00440493	K	539
IPI00440502	K	10
IPI00440502	K	31
IPI00440703	K	71
IPI00440703	K	225

IPI00440727	K	1111
IPI00440727	K	1114
IPI00441344	K	493
IPI00441473	K	200
IPI00442073	K	108
IPI00442073	K	112
IPI00442073	K	131
IPI00442997	K	59
IPI00442997	K	69
IPI00444452	K	148
IPI00444646	K	647
IPI00444646	K	878
IPI00446767	K	237
IPI00446767	K	281
IPI00446986	K	3
IPI00446986	K	149
IPI00446986	K	265
IPI00446986	K	425
IPI00448465	K	39
IPI00448798	K	8
IPI00449049	K	97
IPI00449049	K	105
IPI00449049	K	131
IPI00449049	K	498
IPI00449049	K	505
IPI00449049	K	508
IPI00449049	K	521
IPI00449049	K	524
IPI00449049	K	548
IPI00449049	K	551
IPI00449049	K	600
IPI00449049	K	621
IPI00449197	K	13
IPI00449197	K	352
IPI00449923	K	774
IPI00449923	K	1083
IPI00450472	K	65
IPI00451401	K	14
IPI00451401	K	156
IPI00452463	K	663
IPI00452463	K	664
IPI00453473	K	6
IPI00453473	K	9
IPI00453473	K	13
IPI00453473	K	17
IPI00453473	K	21
IPI00453473	K	32
IPI00453473	K	60
IPI00453473	K	92
IPI00453474	K	19
IPI00453474	K	25
IPI00454695	K	6

IPI00454695	K	12
IPI00454695	K	13
IPI00455210	K	115
IPI00455210	K	116
IPI00455210	K	273
IPI00455210	K	275
IPI00455210	K	285
IPI00455210	K	304
IPI00455210	K	884
IPI00455210	K	987
IPI00455210	K	1671
IPI00455268	K	344
IPI00455268	K	348
IPI00455423	K	146
IPI00455518	K	767
IPI00455620	K	181
IPI00455623	K	136
IPI00456363	K	348
IPI00456429	K	88
IPI00456429	K	93
IPI00456620	K	902
IPI00456635	K	511
IPI00456758	K	47
IPI00456758	K	55
IPI00456758	K	110
IPI00456887	K	471
IPI00456887	K	553
IPI00456887	K	571
IPI00456919	K	509
IPI00456919	K	1840
IPI00456919	K	2248
IPI00456969	K	371
IPI00456969	K	394
IPI00456969	K	754
IPI00456969	K	1125
IPI00456969	K	1286
IPI00456969	K	3480
IPI00456969	K	4283
IPI00456970	K	556
IPI00457284	K	1142
IPI00464979	K	78
IPI00464979	K	143
IPI00465028	K	51
IPI00465028	K	225
IPI00465028	K	231
IPI00465028	K	275
IPI00465044	K	92
IPI00465044	K	293
IPI00465044	K	377
IPI00465070	K	10
IPI00465070	K	15
IPI00465070	K	19

IPI00465070	K	24
IPI00465070	K	28
IPI00465070	K	37
IPI00465070	K	57
IPI00465070	K	80
IPI00465070	K	116
IPI00465070	K	123
IPI00465084	K	240
IPI00465084	K	241
IPI00465170	K	248
IPI00465222	K	234
IPI00465222	K	235
IPI00465233	K	480
IPI00465233	K	488
IPI00465233	K	508
IPI00465233	K	592
IPI00465248	K	5
IPI00465248	K	60
IPI00465248	K	64
IPI00465248	K	71
IPI00465248	K	80
IPI00465248	K	81
IPI00465248	K	89
IPI00465248	K	126
IPI00465248	K	193
IPI00465248	K	197
IPI00465248	K	199
IPI00465248	K	202
IPI00465248	K	221
IPI00465248	K	228
IPI00465248	K	233
IPI00465248	K	256
IPI00465248	K	281
IPI00465248	K	285
IPI00465248	K	306
IPI00465248	K	326
IPI00465248	K	343
IPI00465248	K	406
IPI00465248	K	420
IPI00465256	K	34
IPI00465256	K	189
IPI00465256	K	190
IPI00465275	K	405
IPI00465294	K	626
IPI00465315	K	100
IPI00465345	K	170
IPI00465345	K	585
IPI00465361	K	16
IPI00465361	K	174
IPI00465361	K	177
IPI00465363	K	7
IPI00465363	K	13

IPI00465363	K	14
IPI00465363	K	17
IPI00465363	K	18
IPI00465363	K	22
IPI00465363	K	25
IPI00465363	K	110
IPI00465363	K	122
IPI00465373	K	108
IPI00465373	K	116
IPI00465428	K	3538
IPI00465436	K	237
IPI00465436	K	476
IPI00465439	K	13
IPI00465439	K	42
IPI00465439	K	108
IPI00465439	K	147
IPI00465439	K	230
IPI00465439	K	330
IPI00470416	K	220
IPI00470502	K	53
IPI00470502	K	231
IPI00470502	K	239
IPI00470502	K	240
IPI00470502	K	276
IPI00470502	K	291
IPI00470528	K	83
IPI00470528	K	153
IPI00470528	K	157
IPI00470573	K	327
IPI00470779	K	194
IPI00470779	K	195
IPI00470811	K	11
IPI00470883	K	607
IPI00470891	K	81
IPI00470891	K	434
IPI00472160	K	447
IPI00472724	K	41
IPI00472724	K	44
IPI00472724	K	172
IPI00472724	K	179
IPI00472724	K	318
IPI00472724	K	392
IPI00472724	K	395
IPI00472782	K	435
IPI00472939	K	169
IPI00472939	K	191
IPI00473014	K	19
IPI00473014	K	112
IPI00473014	K	114
IPI00473047	K	273
IPI00473136	K	181
IPI00473136	K	186

IPI00473136	K	866
IPI00477040	K	38
IPI00477259	K	93
IPI00477313	K	39
IPI00477313	K	50
IPI00477313	K	170
IPI00477313	K	216
IPI00477495	K	6
IPI00477495	K	12
IPI00477495	K	13
IPI00477495	K	16
IPI00477495	K	21
IPI00477495	K	109
IPI00477505	K	106
IPI00477526	K	171
IPI00477535	K	8
IPI00477686	K	22
IPI00477686	K	33
IPI00477686	K	137
IPI00477923	K	636
IPI00477923	K	786
IPI00477971	K	26
IPI00478003	K	1162
IPI00478410	K	55
IPI00478410	K	79
IPI00478410	K	90
IPI00478410	K	115
IPI00478410	K	154
IPI00478410	K	197
IPI00478718	K	28
IPI00478758	K	61
IPI00478772	K	17
IPI00479125	K	226
IPI00479125	K	227
IPI00479125	K	230
IPI00479125	K	232
IPI00479125	K	234
IPI00479145	K	97
IPI00479186	K	62
IPI00479186	K	89
IPI00479186	K	135
IPI00479186	K	166
IPI00479186	K	266
IPI00479186	K	305
IPI00479186	K	433
IPI00479191	K	167
IPI00479262	K	1096
IPI00479262	K	1189
IPI00479385	K	583
IPI00479545	K	470
IPI00479545	K	895
IPI00479571	K	31

IPI00479571	K	33
IPI00479571	K	35
IPI00479786	K	87
IPI00479789	K	290
IPI00479877	K	322
IPI00479997	K	9
IPI00479997	K	80
IPI00479997	K	95
IPI00479997	K	100
IPI00479997	K	119
IPI00479997	K	128
IPI00480159	K	1120
IPI00513768	K	320
IPI00513791	K	1962
IPI00513827	K	245
IPI00513827	K	312
IPI00513827	K	334
IPI00514053	K	233
IPI00514053	K	309
IPI00514053	K	351
IPI00514587	K	323
IPI00514648	K	604
IPI00514648	K	994
IPI00514648	K	996
IPI00514648	K	997
IPI00514648	K	999
IPI00514648	K	1543
IPI00514648	K	1547
IPI00514648	K	1551
IPI00514648	K	1553
IPI00514648	K	1555
IPI00514983	K	274
IPI00515061	K	6
IPI00515061	K	12
IPI00515061	K	13
IPI00515097	K	179
IPI00549171	K	33
IPI00549171	K	276
IPI00549189	K	229
IPI00549189	K	230
IPI00549189	K	257
IPI00549189	K	538
IPI00549205	K	270
IPI00549205	K	965
IPI00549205	K	1291
IPI00549232	K	384
IPI00549248	K	27
IPI00549248	K	32
IPI00549248	K	141
IPI00549248	K	150
IPI00549248	K	154
IPI00549248	K	212

IPI00549248	K	215
IPI00549248	K	229
IPI00549248	K	230
IPI00549248	K	257
IPI00549248	K	267
IPI00549248	K	273
IPI00549248	K	292
IPI00549357	K	272
IPI00549664	K	889
IPI00549666	K	142
IPI00549672	K	298
IPI00549725	K	5
IPI00549725	K	100
IPI00549725	K	106
IPI00549725	K	113
IPI00549725	K	176
IPI00549730	K	206
IPI00549730	K	208
IPI00549955	K	377
IPI00550020	K	4
IPI00550020	K	15
IPI00550020	K	92
IPI00550021	K	294
IPI00550021	K	366
IPI00550021	K	373
IPI00550243	K	26
IPI00550308	K	451
IPI00550363	K	17
IPI00550363	K	20
IPI00550364	K	492
IPI00550451	K	147
IPI00550655	K	7
IPI00550689	K	496
IPI00550703	K	750
IPI00550703	K	754
IPI00550746	K	239
IPI00550900	K	93
IPI00550917	K	14
IPI00550917	K	19
IPI00550991	K	260
IPI00550991	K	261
IPI00550991	K	262
IPI00550991	K	441
IPI00550991	K	446
IPI00552413	K	161
IPI00552413	K	166
IPI00552440	K	126
IPI00552701	K	797
IPI00552873	K	6
IPI00552873	K	10
IPI00552873	K	119
IPI00552897	K	812

IPI00552897	K	1402
IPI00552978	K	607
IPI00553153	K	83
IPI00553153	K	84
IPI00553164	K	52
IPI00553164	K	89
IPI00553185	K	222
IPI00553185	K	248
IPI00553214	K	13
IPI00554436	K	605
IPI00554560	K	442
IPI00554579	K	443
IPI00554648	K	11
IPI00554648	K	101
IPI00554648	K	117
IPI00554648	K	122
IPI00554648	K	207
IPI00554648	K	295
IPI00554648	K	325
IPI00554648	K	347
IPI00554648	K	393
IPI00554648	K	472
IPI00554648	K	483
IPI00554652	K	455
IPI00554681	K	30
IPI00554681	K	46
IPI00554681	K	60
IPI00554681	K	98
IPI00554723	K	121
IPI00554723	K	208
IPI00554737	K	280
IPI00554761	K	3
IPI00554761	K	5
IPI00554761	K	14
IPI00554761	K	18
IPI00554761	K	27
IPI00554761	K	31
IPI00554761	K	37
IPI00554761	K	42
IPI00554761	K	48
IPI00554761	K	53
IPI00554761	K	55
IPI00554761	K	59
IPI00554761	K	61
IPI00554761	K	82
IPI00554777	K	385
IPI00554788	K	81
IPI00554788	K	111
IPI00554788	K	131
IPI00554788	K	167
IPI00554788	K	187
IPI00554788	K	214

IPI00554788	K	426
IPI00554798	K	6
IPI00554798	K	12
IPI00554798	K	13
IPI00554798	K	16
IPI00554798	K	17
IPI00554798	K	21
IPI00554798	K	24
IPI00554798	K	109
IPI00554798	K	121
IPI00554811	K	35
IPI00554811	K	89
IPI00555719	K	136
IPI00555744	K	71
IPI00555744	K	79
IPI00555744	K	85
IPI00555876	K	266
IPI00555876	K	267
IPI00555902	K	41
IPI00556027	K	319
IPI00556365	K	503
IPI00556369	K	173
IPI00556553	K	217
IPI00556553	K	252
IPI00604400	K	324
IPI00604590	K	37
IPI00604590	K	81
IPI00604590	K	125
IPI00604590	K	189
IPI00604590	K	196
IPI00604590	K	225
IPI00604590	K	264
IPI00604590	K	268
IPI00604620	K	9
IPI00604620	K	15
IPI00604620	K	79
IPI00604620	K	102
IPI00604620	K	109
IPI00604620	K	116
IPI00604620	K	124
IPI00604620	K	132
IPI00604620	K	318
IPI00604620	K	333
IPI00604620	K	370
IPI00604620	K	377
IPI00604620	K	398
IPI00604620	K	403
IPI00604620	K	444
IPI00604620	K	449
IPI00604620	K	467
IPI00604620	K	513
IPI00604620	K	572

IPI00604620	K	577
IPI00604620	K	646
IPI00604624	K	109
IPI00604756	K	134
IPI00607554	K	122
IPI00607584	K	71
IPI00607584	K	158
IPI00607584	K	1317
IPI00607591	K	230
IPI00607591	K	421
IPI00607708	K	5
IPI00607708	K	14
IPI00607708	K	22
IPI00607708	K	57
IPI00607708	K	81
IPI00607708	K	118
IPI00607708	K	126
IPI00607708	K	222
IPI00607708	K	278
IPI00607708	K	318
IPI00607799	K	48
IPI00607799	K	151
IPI00639841	K	51
IPI00639841	K	62
IPI00639841	K	92
IPI00640088	K	231
IPI00640136	K	704
IPI00640703	K	396
IPI00641026	K	63
IPI00641109	K	647
IPI00641384	K	622
IPI00641436	K	604
IPI00641743	K	288
IPI00641743	K	813
IPI00641743	K	836
IPI00641743	K	2050
IPI00641743	K	2073
IPI00641743	K	2074
IPI00641788	K	73
IPI00641829	K	36
IPI00641829	K	53
IPI00641829	K	203
IPI00641829	K	349
IPI00641924	K	287
IPI00642097	K	237
IPI00642097	K	325
IPI00642097	K	619
IPI00642097	K	622
IPI00642156	K	486
IPI00642186	K	951
IPI00642211	K	454
IPI00642238	K	190

IPI00642329	K	36
IPI00642329	K	243
IPI00642374	K	170
IPI00642550	K	649
IPI00642816	K	30
IPI00642816	K	52
IPI00642904	K	104
IPI00642971	K	383
IPI00642971	K	473
IPI00642971	K	483
IPI00643027	K	892
IPI00643027	K	1010
IPI00643027	K	1017
IPI00643041	K	60
IPI00643041	K	71
IPI00643041	K	99
IPI00643041	K	159
IPI00643435	K	183
IPI00643435	K	539
IPI00643435	K	543
IPI00643465	K	2884
IPI00643591	K	101
IPI00643591	K	167
IPI00643722	K	997
IPI00643722	K	1007
IPI00643722	K	1612
IPI00643722	K	1905
IPI00643920	K	6
IPI00643920	K	11
IPI00643920	K	144
IPI00644055	K	103
IPI00644055	K	369
IPI00644055	K	392
IPI00644055	K	400
IPI00644127	K	844
IPI00644127	K	848
IPI00644231	K	1197
IPI00644648	K	779
IPI00644708	K	139
IPI00644712	K	31
IPI00644712	K	282
IPI00644712	K	317
IPI00644712	K	331
IPI00644712	K	338
IPI00644712	K	461
IPI00644712	K	468
IPI00644712	K	539
IPI00644712	K	542
IPI00644712	K	544
IPI00644712	K	553
IPI00644712	K	556
IPI00645078	K	89

IPI00645078	K	671
IPI00645078	K	980
IPI00645078	K	984
IPI00645192	K	461
IPI00645446	K	52
IPI00645446	K	100
IPI00645446	K	104
IPI00645616	K	507
IPI00645616	K	530
IPI00645643	K	398
IPI00645805	K	75
IPI00645947	K	811
IPI00646058	K	475
IPI00646058	K	607
IPI00646304	K	98
IPI00646304	K	165
IPI00646304	K	209
IPI00646350	K	6
IPI00646361	K	143
IPI00646377	K	944
IPI00646645	K	1647
IPI00646689	K	78
IPI00646762	K	42
IPI00646762	K	223
IPI00646762	K	231
IPI00646839	K	559
IPI00646909	K	370
IPI00646909	K	394
IPI00646909	K	401
IPI00646917	K	23
IPI00646917	K	29
IPI00646917	K	56
IPI00646917	K	73
IPI00646917	K	189
IPI00647217	K	51
IPI00647217	K	78
IPI00647217	K	79
IPI00647217	K	198
IPI00647217	K	379
IPI00647217	K	748
IPI00647217	K	796
IPI00647491	K	512
IPI00647915	K	38
IPI00647915	K	41
IPI00651737	K	135
IPI00654555	K	91
IPI00654555	K	682
IPI00654731	K	858
IPI00654731	K	909
IPI00654755	K	145
IPI00654777	K	253
IPI00655631	K	1033

IPI00655650	K	66
IPI00655650	K	70
IPI00655650	K	113
IPI00656021	K	110
IPI00656021	K	360
IPI00657642	K	127
IPI00657682	K	87
IPI00657687	K	371
IPI00657967	K	52
IPI00658023	K	198
IPI00658023	K	199
IPI00658023	K	280
IPI00658210	K	121
IPI00658210	K	214
IPI00658210	K	578
IPI00658210	K	581
IPI00718888	K	179
IPI00719285	K	1763
IPI00719680	K	176
IPI00719680	K	736
IPI00719752	K	209
IPI00719752	K	213
IPI00719752	K	288
IPI00719752	K	364
IPI00719752	K	436
IPI00719752	K	729
IPI00737057	K	1024
IPI00738216	K	1218
IPI00738216	K	2147
IPI00739119	K	171
IPI00739386	K	1365
IPI00740057	K	1773
IPI00740961	K	175
IPI00742682	K	252
IPI00742682	K	312
IPI00742682	K	315
IPI00742682	K	345
IPI00742682	K	428
IPI00742682	K	457
IPI00742682	K	713
IPI00742682	K	723
IPI00742682	K	748
IPI00742682	K	755
IPI00742743	K	1672
IPI00743143	K	112
IPI00743143	K	127
IPI00743143	K	129
IPI00743143	K	130
IPI00743143	K	131
IPI00743143	K	146
IPI00743143	K	148
IPI00743143	K	156

IPI00743143	K	160
IPI00743143	K	161
IPI00743143	K	236
IPI00743157	K	790
IPI00743335	K	285
IPI00743335	K	647
IPI00743335	K	704
IPI00743509	K	168
IPI00743509	K	171
IPI00743509	K	176
IPI00743594	K	372
IPI00743813	K	1903
IPI00743879	K	409
IPI00744211	K	134
IPI00744507	K	102
IPI00744692	K	219
IPI00744692	K	269
IPI00744692	K	286
IPI00744692	K	321
IPI00744711	K	264
IPI00744711	K	275
IPI00744711	K	285
IPI00744711	K	289
IPI00744711	K	591
IPI00744810	K	108
IPI00744851	K	15
IPI00744858	K	127
IPI00745087	K	200
IPI00745266	K	437
IPI00745266	K	465
IPI00745266	K	549
IPI00745568	K	106
IPI00745568	K	207
IPI00745793	K	73
IPI00745872	K	223
IPI00745872	K	229
IPI00745872	K	438
IPI00745872	K	499
IPI00746165	K	28
IPI00746165	K	81
IPI00746165	K	95
IPI00746165	K	115
IPI00746165	K	180
IPI00746165	K	182
IPI00746165	K	480
IPI00746251	K	6
IPI00746251	K	12
IPI00746251	K	13
IPI00746310	K	7
IPI00746310	K	15
IPI00746310	K	18
IPI00746310	K	23

IPI00746310	K	31
IPI00746310	K	46
IPI00746310	K	55
IPI00746310	K	62
IPI00746310	K	65
IPI00746337	K	210
IPI00746351	K	18
IPI00746351	K	922
IPI00746398	K	274
IPI00746412	K	1392
IPI00746655	K	689
IPI00746655	K	827
IPI00746684	K	818
IPI00746777	K	366
IPI00746934	K	1084
IPI00747327	K	408
IPI00747478	K	203
IPI00747810	K	471
IPI00747913	K	47
IPI00748037	K	203
IPI00748360	K	819
IPI00748532	K	616
IPI00748532	K	619
IPI00748532	K	620
IPI00749113	K	179
IPI00749237	K	14
IPI00749245	K	223
IPI00749245	K	224
IPI00749245	K	231
IPI00749245	K	239
IPI00749406	K	4
IPI00749406	K	10
IPI00759562	K	132
IPI00759562	K	235
IPI00759691	K	496
IPI00759749	K	503
IPI00761080	K	154
IPI00761160	K	712
IPI00782935	K	114
IPI00782935	K	154
IPI00782935	K	226
IPI00782950	K	614
IPI00782966	K	1350
IPI00782966	K	1351
IPI00782966	K	1352
IPI00782966	K	1358
IPI00782992	K	37
IPI00782992	K	169
IPI00782992	K	1186
IPI00782992	K	1467
IPI00783004	K	943
IPI00783004	K	945

IPI00783017	K	412
IPI00783017	K	417
IPI00783097	K	204
IPI00783097	K	219
IPI00783097	K	309
IPI00783097	K	501
IPI00783271	K	155
IPI00783271	K	187
IPI00783271	K	292
IPI00783271	K	613
IPI00783271	K	726
IPI00783271	K	750
IPI00783271	K	864
IPI00783302	K	126
IPI00783359	K	55
IPI00783502	K	228
IPI00783559	K	454
IPI00783781	K	41
IPI00783781	K	44
IPI00783835	K	305
IPI00783835	K	308
IPI00783874	K	179
IPI00784090	K	7
IPI00784090	K	20
IPI00784090	K	318
IPI00784090	K	400
IPI00784090	K	466
IPI00784154	K	31
IPI00784154	K	82
IPI00784154	K	87
IPI00784154	K	125
IPI00784154	K	130
IPI00784154	K	156
IPI00784154	K	157
IPI00784154	K	191
IPI00784154	K	202
IPI00784154	K	218
IPI00784154	K	233
IPI00784154	K	236
IPI00784154	K	249
IPI00784154	K	250
IPI00784154	K	269
IPI00784154	K	352
IPI00784154	K	359
IPI00784154	K	389
IPI00784154	K	396
IPI00784154	K	462
IPI00784154	K	469
IPI00784154	K	473
IPI00784154	K	523
IPI00784154	K	551
IPI00784154	K	554

IPI00784161	K	402
IPI00784161	K	743
IPI00784161	K	1676
IPI00784224	K	317
IPI00784224	K	509
IPI00784224	K	910
IPI00784366	K	265
IPI00784366	K	318
IPI00784414	K	49
IPI00784414	K	87
IPI00784414	K	685
IPI00784473	K	3523
IPI00784614	K	352
IPI00784614	K	431
IPI00785015	K	1148
IPI00785110	K	880
IPI00785113	K	568
IPI00785113	K	570
IPI00788907	K	116
IPI00788907	K	144
IPI00788907	K	191
IPI00788925	K	44
IPI00788925	K	47
IPI00788925	K	322
IPI00788925	K	378
IPI00789041	K	238
IPI00789101	K	37
IPI00789101	K	39
IPI00789159	K	108
IPI00789442	K	488
IPI00789551	K	3
IPI00789551	K	473
IPI00789551	K	522
IPI00789551	K	524
IPI00789551	K	571
IPI00789551	K	877
IPI00789551	K	879
IPI00789551	K	884
IPI00789605	K	50
IPI00789605	K	81
IPI00789618	K	25
IPI00789618	K	97
IPI00789618	K	123
IPI00789699	K	1062
IPI00789798	K	774
IPI00789848	K	78
IPI00789941	K	244
IPI00789941	K	249
IPI00789941	K	357
IPI00790342	K	5
IPI00790342	K	211
IPI00790342	K	219

IPI00790342	K	240
IPI00790503	K	442
IPI00790503	K	552
IPI00790503	K	1245
IPI00790503	K	1268
IPI00790503	K	1366
IPI00790503	K	1666
IPI00790644	K	78
IPI00790739	K	50
IPI00790739	K	598
IPI00790739	K	630
IPI00791537	K	1173
IPI00791574	K	29
IPI00792191	K	438
IPI00792333	K	981
IPI00792333	K	984
IPI00792352	K	57
IPI00792352	K	58
IPI00792352	K	80
IPI00792352	K	91
IPI00792352	K	119
IPI00792352	K	179
IPI00792743	K	401
IPI00792743	K	406
IPI00792743	K	1024
IPI00792788	K	818
IPI00792984	K	45
IPI00792984	K	50
IPI00792984	K	128
IPI00793199	K	215
IPI00793199	K	227
IPI00793199	K	295
IPI00793199	K	302
IPI00793201	K	57
IPI00793375	K	173
IPI00793375	K	174
IPI00793375	K	347
IPI00793696	K	77
IPI00794402	K	186
IPI00794402	K	223
IPI00794461	K	6
IPI00794461	K	12
IPI00794461	K	13
IPI00794461	K	16
IPI00794461	K	17
IPI00794461	K	21
IPI00794461	K	109
IPI00794461	K	121
IPI00794779	K	990
IPI00794806	K	253
IPI00795015	K	943
IPI00795015	K	945

IPI00795015	K	2495
IPI00795015	K	2501
IPI00795043	K	354
IPI00795043	K	363
IPI00795292	K	127
IPI00795292	K	146
IPI00795292	K	164
IPI00795292	K	171
IPI00795292	K	200
IPI00795292	K	215
IPI00795292	K	239
IPI00795292	K	243
IPI00795922	K	138
IPI00796331	K	64
IPI00796331	K	66
IPI00796331	K	68
IPI00796331	K	70
IPI00796333	K	96
IPI00796333	K	162
IPI00796333	K	201
IPI00796333	K	284
IPI00796333	K	384
IPI00796513	K	294
IPI00796864	K	232
IPI00796934	K	33
IPI00796934	K	82
IPI00797126	K	818
IPI00797126	K	852
IPI00797126	K	854
IPI00797230	K	46
IPI00797279	K	412
IPI00797279	K	559
IPI00797574	K	473
IPI00797574	K	2430
IPI00797720	K	208
IPI00797720	K	209
IPI00797720	K	211
IPI00797720	K	213
IPI00797945	K	399
IPI00797945	K	546
IPI00798011	K	11
IPI00798155	K	6
IPI00798155	K	48
IPI00807573	K	530
IPI00807625	K	27
IPI00807625	K	66
IPI00807625	K	110
IPI00807625	K	132
IPI00815642	K	30
IPI00815642	K	38
IPI00815642	K	41
IPI00815642	K	52

IPI00815642	K	58
IPI00815642	K	65
IPI00815707	K	1685
IPI00815713	K	155
IPI00815713	K	296
IPI00815713	K	600
IPI00815713	K	755
IPI00815713	K	1451
IPI00816288	K	32
IPI00816288	K	39
IPI00816773	K	38
IPI00816773	K	43
IPI00827535	K	15
IPI00827535	K	18
IPI00827535	K	103
IPI00827535	K	104
IPI00827930	K	416
IPI00827930	K	419
IPI00827930	K	421
IPI00827967	K	663
IPI00828125	K	507
IPI00829826	K	15
IPI00829826	K	1076
IPI00829826	K	1177
IPI00829826	K	1309
IPI00829826	K	1311
IPI00829826	K	1354
IPI00829826	K	1502
IPI00829826	K	1504
IPI00829826	K	1529
IPI00843975	K	60
IPI00843975	K	79
IPI00843975	K	139
IPI00843975	K	253
IPI00844000	K	571
IPI00844214	K	91
IPI00844264	K	127
IPI00844310	K	243
IPI00844508	K	579
IPI00844578	K	191
IPI00844578	K	193
IPI00844578	K	199
IPI00844578	K	1024
IPI00845227	K	185
IPI00845355	K	967
IPI00845355	K	1930
IPI00845355	K	1933
IPI00845355	K	1935
IPI00845355	K	1936
IPI00845355	K	1939
IPI00845461	K	418
IPI00845479	K	471

IPI00845508	K	222
IPI00847436	K	196
IPI00848226	K	130
IPI00848321	K	185
IPI00848321	K	186
IPI00852603	K	1053
IPI00852603	K	1059
IPI00852685	K	1057
IPI00852685	K	1103
IPI00852960	K	129
IPI00852979	K	1845
IPI00853009	K	462
IPI00853077	K	738
IPI00853077	K	910
IPI00853077	K	917
IPI00853240	K	266
IPI00853240	K	621
IPI00853240	K	625
IPI00853240	K	628
IPI00853240	K	776
IPI00853400	K	92
IPI00854642	K	974
IPI00854642	K	1146
IPI00854642	K	1211
IPI00854642	K	1290
IPI00855767	K	231
IPI00855767	K	422
IPI00855833	K	1223
IPI00855833	K	1226
IPI00855856	K	51
IPI00855856	K	251
IPI00855924	K	27
IPI00855924	K	68
IPI00855980	K	91
IPI00855998	K	1673
IPI00855998	K	2757
IPI00855998	K	2875
IPI00856045	K	1510
IPI00856049	K	70
IPI00856115	K	90
IPI00856115	K	333
IPI00867509	K	390
IPI00867509	K	499
IPI00867714	K	171
IPI00869087	K	21
IPI00871134	K	28
IPI00871535	K	592
IPI00871535	K	613
IPI00871535	K	637
IPI00871535	K	779
IPI00871535	K	1022
IPI00871535	K	1023

IPI00871535	K	1519
IPI00871535	K	2057
IPI00871535	K	2426
IPI00871535	K	2431
IPI00871539	K	405
IPI00871539	K	1104
IPI00871780	K	319
IPI00871780	K	895
IPI00871851	K	225
IPI00871870	K	56
IPI00871870	K	61
IPI00872071	K	106
IPI00872359	K	235
IPI00872411	K	170
IPI00872762	K	54
IPI00872780	K	213
IPI00872780	K	225
IPI00872780	K	293
IPI00872780	K	300
IPI00872874	K	51
IPI00873029	K	681
IPI00873029	K	809
IPI00873082	K	381
IPI00873495	K	1126
IPI00873518	K	61
IPI00873622	K	28
IPI00873622	K	81
IPI00873622	K	115
IPI00873622	K	481
IPI00873622	K	512
IPI00873703	K	1462
IPI00873810	K	53
IPI00874020	K	125
IPI00876931	K	47
IPI00877014	K	203
IPI00878183	K	452
IPI00878213	K	1055
IPI00878215	K	152
IPI00878910	K	32
IPI00878910	K	38
IPI00878910	K	298
IPI00878931	K	150
IPI00879588	K	891
IPI00879588	K	925
IPI00879819	K	27
IPI00879819	K	61
IPI00879819	K	373
IPI00879819	K	500
IPI00880048	K	252
IPI00883857	K	352
IPI00883857	K	565
IPI00883857	K	609

IPI00883857	K	635
IPI00883857	K	814
IPI00884896	K	243
IPI00884896	K	409
IPI00885078	K	536
IPI00885104	K	163
IPI00885104	K	169
IPI00885104	K	170
IPI00885104	K	171
IPI00885104	K	172
IPI00885104	K	177
IPI00888429	K	527
IPI00890837	K	1349
IPI00893197	K	112
IPI00893197	K	352
IPI00894296	K	278
IPI00895801	K	216
IPI00895801	K	283
IPI00895801	K	305
IPI00902533	K	317
IPI00902533	K	319
IPI00902560	K	46
IPI00902560	K	54
IPI00902560	K	87
IPI00902560	K	89
IPI00902560	K	135
IPI00902906	K	993
IPI00903139	K	338
IPI00903145	K	79
IPI00903145	K	253
IPI00903145	K	258
IPI00903145	K	263
IPI00903145	K	344
IPI00908586	K	241
IPI00909140	K	58
IPI00909140	K	103
IPI00909140	K	379
IPI00910610	K	7
IPI00914566	K	123
IPI00914566	K	353
IPI00914930	K	720
IPI00915324	K	118
IPI00915334	K	1168
IPI00915334	K	1174
IPI00915400	K	263
IPI00915400	K	270
IPI00915400	K	429
IPI00915400	K	555
IPI00916027	K	471
IPI00916027	K	472
IPI00916027	K	474
IPI00916111	K	121

IPI00916111	K	136
IPI00916111	K	316
IPI00916144	K	267
IPI00916144	K	270
IPI00916332	K	758
IPI00916332	K	827
IPI00916332	K	1508
IPI00916332	K	1772
IPI00916332	K	2009
IPI00916332	K	2802
IPI00916332	K	2809
IPI00916332	K	2814
IPI00916332	K	2832
IPI00916332	K	3714
IPI00921422	K	48
IPI00921844	K	264
IPI00922697	K	77
IPI00922697	K	83
IPI00922697	K	328
IPI00922697	K	343
IPI00924816	K	4
IPI00924816	K	11
IPI00924816	K	24
IPI00924816	K	97
IPI00925046	K	309
IPI00925046	K	620
IPI00925255	K	28
IPI00925345	K	198
IPI00925924	K	804
IPI00926625	K	24
IPI00926625	K	265
IPI00926625	K	272
IPI00926625	K	279
IPI00926625	K	528
IPI00927606	K	148
IPI00929648	K	86
IPI00930290	K	373
IPI00930609	K	51
IPI00930609	K	269
IPI00930609	K	318
IPI00930609	K	323
IPI00930609	K	333
IPI00930688	K	40
IPI00930688	K	163
IPI00930688	K	280
IPI00930688	K	311
IPI00930688	K	394
IPI00930688	K	401
IPI00935252	K	1166
IPI00935252	K	1170
IPI00935252	K	1171
IPI00935252	K	1174

IPI00935252	K	1178
IPI00936931	K	267
IPI00937545	K	1136
IPI00937615	K	132
IPI00937615	K	147
IPI00937615	K	212
IPI00937615	K	434
IPI00939163	K	68
IPI00939163	K	234
IPI00939163	K	272
IPI00939163	K	430
IPI00939238	K	51
IPI00939238	K	70
IPI00939265	K	262
IPI00939320	K	173
IPI00939493	K	174
IPI00939493	K	189
IPI00939776	K	39
IPI00939879	K	144
IPI00940148	K	54
IPI00940148	K	112
IPI00940148	K	269
IPI00940180	K	159
IPI00940237	K	35
IPI00940237	K	187
IPI00940237	K	333
IPI00940437	K	135
IPI00940535	K	61
IPI00940685	K	18
IPI00940744	K	84
IPI00940842	K	101
IPI00941161	K	202
IPI00941161	K	815
IPI00941221	K	418
IPI00941255	K	238
IPI00941331	K	196
IPI00941331	K	367
IPI00941501	K	394
IPI00941534	K	187
IPI00941534	K	209
IPI00941534	K	214
IPI00941747	K	137
IPI00941747	K	458
IPI00941928	K	496
IPI00941972	K	40
IPI00942092	K	147
IPI00942092	K	295
IPI00942246	K	516
IPI00942458	K	510
IPI00942495	K	236
IPI00942495	K	241
IPI00942668	K	44

IPI00942760	K	340
IPI00942976	K	43
IPI00942979	K	6
IPI00942979	K	11
IPI00942979	K	144
IPI00942979	K	204
IPI00942979	K	232
IPI00942979	K	241
IPI00942979	K	260
IPI00942979	K	314
IPI00942979	K	352
IPI00942979	K	456
IPI00942979	K	597
IPI00942979	K	603
IPI00943074	K	459
IPI00943093	K	305
IPI00943167	K	1022
IPI00943173	K	337
IPI00943173	K	446
IPI00945964	K	175
IPI00946154	K	168
IPI00946154	K	261
IPI00946154	K	267
IPI00946154	K	271
IPI00946154	K	277
IPI00953461	K	724
IPI00953625	K	353
IPI00953689	K	225
