# Supplementary material for: Bioinformatic Analysis and Post-Translational Modification Crosstalk Prediction of Lysine Acetylation
Source: PLoS One. 2011 Dec 2;6(12):e28228. doi: 10.1371/journal.pone.0028228 (PMC3229533; doi:10.1371/journal.pone.0028228)
Supplement: Table S2 — Domain enrichment. These are the complete data for Figure 4. Proteins with acetylated lysines were aligned to the distribution of domains in the NCBI Conserved Domain Database using PRS-BLAST. The distribution was tested against that of all IPI proteins using a two-tailed Fisher's exact test. (PDF) [file pone.0028228.s005.pdf]

Table S2

Overrepresented

domainID	Domain Name	Domain Description	n	m	N	M	p-value	FC	FDR	-log10(FDR)
cl09938	cond_enzymes.	cond_enzymes. Thiolase is reported to be structurally related to beta-ketoacyl synthase (pfam00109), and also chalcone synthase.	230	39	115824	5696	5.440E-10	3.448	9.390E-09	8.027
cl00074	H2A.	H2A. histone H2B; Provisional	406	52	115824	5696	6.780E-09	2.604	1.000E-07	7.000
cl02777	chaperonin_like.	chaperonin_like. Thermosome is the name given to the archaeal rather than eukaryotic form of the group II chaperonin (counterpart to the group I chaperonin, GroEL/GroES, in bacterial), a torroidal, ATP-dependent molecular chaperone that assists in the folding or refolding of nascent or denatured proteins. Various homologous subunits, one to five per archaeal genome, may be designated alpha, beta, etc., but phylogenetic analysis does not show distinct alpha subunit and beta subunit lineages traceable to ancient paralogs.	509	56	115824	5696	2.310E-07	2.237	2.870E-06	5.542
cl00445	Iso_dh,Isocitrate/isopropylmalate dehydrogenase	Iso_dh.Isocitrate/isopropylmalate dehydrogenase. Several NAD- or NADP-dependent dehydrogenases, including 3-isopropylmalate dehydrogenase, tartrate dehydrogenase, and the dimeric forms of isocitrate dehydrogenase, share a nucleotide binding domain unrelated to that of lactate dehydrogenase and its homologs. These enzymes dehydrogenate their substates at a H-C-OH site adjacent to a H-C-COOH site; the latter carbon, now adjacent to a carbonyl group, readily decarboxylates.Among these decarboxylating dehydrogenases of hydroxyacids, overall sequence homology indicates evolutionary history rather than actual substrate or cofactor specifity, which may be toggled experimentally by replacement of just a few amino acids. 3-isopropylmalate dehydrogenase is an NAD-dependent enzyme and should have a sequence resembling HGSAPDI around residue 340. The subtrate binding loop should include a sequence resembling E[KQR]X(0,1)LLXXR around residue 115. Other contacts of importance are known from crystallography but not detailed here.This HMM will not find all isopropylmalate dehydrogenases; the enzyme from Sulfolobus sp. strain 7 is	106	20	115824	5696	1.850E-06	3.837	1.980E-05	4.703
cl11598	Thymosin,Thymosin beta-4 family	Thymosin.Thymosin beta-4 family.	35	11	115824	5696	7.000E-06	6.391	7.080E-05	4.150
pfam00208	ELFV_dehydrog,Glutamate/Leucine/Phenylalanine/Valine dehydrogenase	ELFV_dehydrog.Glutamate/Leucine/Phenylalanine/Valine dehydrogenase.	36	11	115824	5696	8.750E-06	6.213	8.600E-05	4.066
pfam02115	Rho_GDI,RHO protein GDP dissociation inhibitor	Rho_GDI.RHO protein GDP dissociation inhibitor.	80	16	115824	5696	1.010E-05	4.067	9.800E-05	4.009
pfam00213	OSCP,ATP synthase delta (OSCP) subunit	OSCP.ATP synthase delta (OSCP) subunit. The ATP D subunit from E. coli is the same as the OSCP subunit which is this family. The ATP D subunit from metazoa are found in family pfam00401.	17	8	115824	5696	1.230E-05	9.569	1.146E-04	3.941
pfam00183	HSP90,Hsp90 protein	HSP90.Hsp90 protein.	361	39	115824	5696	2.380E-05	2.197	2.133E-04	3.671
pfam00118	Cpn60_TCP1,TCP-1/cpn60 chaperonin family	Cpn60_TCP1.TCP-1/cpn60 chaperonin family. This family includes members from the HSP60 chaperone family and the TCP-1 (T-complex protein) family.	152	22	115824	5696	2.540E-05	2.943	2.248E-04	3.648
pfam05873	Mt_ATP-synt_D,ATP synthase D chain, mitochondrial (ATP5H)	Mt_ATP-synt_D.ATP synthase D chain, mitochondrial (ATP5H). This family consists of several ATP synthase D chain, mitochondrial (ATP5H) proteins. Subunit d has no extensive hydrophobic sequences, and is not apparently related to any subunit described in the simpler ATP synthases in bacteria and chloroplasts.	35	10	115824	5696	3.620E-05	5.810	3.164E-04	3.500
cl09108	TIM_phosphate_binding.	TIM_phosphate_binding. 2-deoxyribose-5-phosphate aldolase (DERA) of the DeoC family. DERA belongs to the class I aldolases and catalyzes a reversible aldol reaction between acetaldehyde and glyceraldehyde 3-phosphate to generate 2-deoxyribose 5-phosphate. DERA is unique in catalyzing the aldol reaction between two aldehydes, and its broad substrate specificity confers considerable utility as a biocatalyst, offering an environmentally benign alternative to chiral transition metal catalysis of the asymmetric aldol reaction.	509	48	115824	5696	7.480E-05	1.918	6.018E-04	3.221
cl00335	NDPk.	NDPk. nucleoside diphosphate kinase; Provisional	107	17	115824	5696	7.320E-05	3.231	6.026E-04	3.220
pfam06001	DUF902,Domain of Unknown Function (DUF902)	DUF902.Domain of Unknown Function (DUF902).	6	5	115824	5696	8.260E-05	16.945	6.426E-04	3.192

cl11406	LDH_MDH_like,NAD-dependent, lactate dehydrogenase-like, 2-hydroxycarboxylate dehydrogenase family	LDH_MDH_like.NAD-dependent, lactate dehydrogenase-like, 2-hydroxycarboxylate dehydrogenase family. L-lactate dehydrogenases are metabolic enzymes which catalyse the conversion of L-lactate to pyruvate, the last step in anaerobic glycolysis. L-2-hydroxyisocaproate dehydrogenases are also members of the family. Malate dehydrogenases catalyse the interconversion of malate to oxaloacetate. The enzyme participates in the citric acid cycle. L-lactate dehydrogenase is also found as a lens crystallin in bird and crocodile eyes. N-terminus (this family) is a Rossmann NAD-binding fold. C-terminus is an unusual alpha+beta fold	247	28	115824	5696	1.441E-04	2.305	1.063E-03	2.974
pfam00285	Citrate synt,Citrate synthase	Citrate_synt.Citrate synthase.	44	10	115824	5696	1.845E-04	4.621	1.347E-03	2.871
cl10017	Tubulin_FtsZ.	Tubulin_FtsZ. This domain is found in all tubulin chains, as well as the bacterial FtsZ family of proteins. These proteins are involved in polymer formation. Tubulin is the major component of microtubules, while FtsZ is the polymer-forming protein of bacterial cell division, it is part of a ring in the middle of the dividing cell that is required for constriction of cell membrane and cell envelope to yield two daughter cells. FtsZ and tubulin are GTPases, this entry is the GTPase domain. FtsZ can polymerise into tubes, sheets, and rings in vitro and is ubiquitous in bacteria and archaea	552	49	115824	5696	2.179E-04	1.805	1.574E-03	2.803
pfam05405	Mt_ATP-synt_B,Mitochondrial ATP synthase B chain precursor (ATP-synt_B)	Mt_ATP-synt_B.Mitochondrial ATP synthase B chain precursor (ATP-synt_B). The Fo sector of the ATP synthase is a membrane bound complex which mediates proton transport. It is composed of nine different polypeptide subunits (a, b, c, d, e, f, g F6, A6L).	15	6	115824	5696	3.132E-04	8.134	2.218E-03	2.654
cl00416	CS ACL-C CCL.	CS_ACL-C_CCL. citrate synthase; Provisional	58	11	115824	5696	3.540E-04	3.856	2.457E-03	2.610
cl00029	ADF.	ADF. actin depolymerizing factor; Provisional	199	23	115824	5696	3.775E-04	2.350	2.595E-03	2.586
cl02660	zf-TAZ,TAZ zinc finger	zf-TAZ.TAZ zinc finger. The TAZ2 domain of CBP binds to other transcription factors such as the p53 tumour suppressor protein, E1A oncoprotein, MyoD, and GATA-1. The zinc coordinating motif that is necessary for binding to target DNA sequences consists of HCCC.	10	5	115824	5696	4.581E-04	10.167	3.060E-03	2.514
cl02574	Annexin,Annexin	Annexin.Annexin. This family of annexins also includes giardin that has been shown to function as an annexin.	132	17	115824	5696	6.706E-04	2.619	4.356E-03	2.361
pfam00026	Asp,Eukaryotic aspartyl protease	Asp.Eukaryotic aspartyl protease. Aspartyl (acid) proteases include pepsins, cathepsins, and renins. Two-domain structure, probably arising from ancestral duplication. This family does not include the retroviral nor retrotransposon proteases (pfam00077), which are much smaller and appear to be homologous to a single domain of the eukaryotic asp proteases.	27	7	115824	5696	8.755E-04	5.272	5.584E-03	2.253
pfam03952	Enolase N,Enolase, N-terminal domain	Enolase_N.Enolase, N-terminal domain.	36	8	115824	5696	9.104E-04	4.519	5.755E-03	2.240
pfam01459	Porin_3,Eukaryotic porin	Porin_3.Eukaryotic porin.	126	16	115824	5696	1.086E-03	2.582	6.744E-03	2.171
cl03224	Porin3,Eukaryotic porin family that forms channels in the mitochondrial outer membrane	Porin3.Eukaryotic porin family that forms channels in the mitochondrial outer membrane. The voltage-dependent anion channel (VDAC) regulates the flux of mostly anionic metabolites through the outer mitochondrial membrane, which is highly permeable to small molecules. VDAC is the most abundant protein in the outer membrane, and membrane potentials can toggle VDAC between open or high-conducting and closed or low-conducting forms. VDAC binds to and is regulated in part by hexokinase, an interaction that renders mitochondria less susceptible to pro-apoptotic signals, most likely by intefering with VDACs capability to respond to Bcl-2 family proteins. While VDAC appears to play a key role in mitochondrially induced cell death, a proposed involvement in forming the mitochondrial permeability transition pore, which is characteristic for damaged mitochondria and apoptosis, has	128	16	115824	5696	1.260E-03	2.542	7.757E-03	2.110
pfam00012	HSP70,Hsp70 protein	HSP70.Hsp70 protein. Hsp70 chaperones help to fold many proteins. Hsp70 assisted folding involves repeated cycles of substrate binding and release. Hsp70 activity is ATP dependent. Hsp70 proteins are made up of two regions: the amino terminus is the ATPase domain and the carboxyl terminus is the substrate binding region.	549	45	115824	5696	1.793E-03	1.667	1.076E-02	1.968

pfam06723	MreB_Mbl,MreB/Mbl protein	MreB_Mbl.MreB/Mbl protein. This family consists of bacterial MreB and Mbl proteins as well as two related archaeal sequences. MreB is known to be a rod shape-determining protein in bacteria and goes to make up the bacterial cytoskeleton. Genes coding for MreB/Mbl are only found in elongated bacteria, not in coccoid forms. It has been speculated that constituents of the eukaryotic cytoskeleton (tubulin, actin) may have evolved from prokaryotic precursor proteins closely related to todays bacterial proteins FtsZ and MreB/Mbl	190	20	115824	5696	2.745E-03	2.140	1.606E-02	1.794
cl02786	Translation_factor_III.	Translation_factor_III. Elongation factor Tu consists of three structural domains, this is the third domain. This domain adopts a beta barrel structure. This the third domain is involved in binding to both charged tRNA and binding to EF-Ts pfam00889.	101	13	115824	5696	2.740E-03	2.617	1.616E-02	1.791
pfam00390	malic,Malic enzyme, N-terminal domain	malic.Malic enzyme, N-terminal domain.	25	6	115824	5696	2.841E-03	4.880	1.649E-02	1.783
cl02423	LRRNT,Leucine rich repeat N-terminal domain	LRRNT.Leucine rich repeat N-terminal domain. Leucine Rich Repeats pfam00560 are short sequence motifs present in a number of proteins with diverse functions and cellular locations. Leucine Rich Repeats are often flanked by cysteine rich domains. This domain is often found at the N-terminus of tandem leucine rich repeats.	4	3	115824	5696	3.128E-03	15.251	1.786E-02	1.748
cl00876	Ribosomal_S27,Ribosomal protein S27a	Ribosomal_S27.Ribosomal protein S27a. 30S ribosomal protein S27ae; Validated	4	3	115824	5696	3.128E-03	15.251	1.801E-02	1.745
pfam03730	Ku_C,Ku70/Ku80 C-terminal arm	Ku_C.Ku70/Ku80 C-terminal arm. The Ku heterodimer (composed of Ku70 and Ku80) contributes to genomic integrity through its ability to bind DNA double-strand breaks and facilitate repair by the non-homologous end-joining pathway. This is the C terminal arm. This alpha helical region embraces the beta-barrel domain pfam02735 of the opposite subunit.	26	6	115824	5696	3.359E-03	4.693	1.873E-02	1.728
cl00365	ATP-synt,ATP synthase	ATP-synt.ATP synthase. F0F1 ATP synthase subunit gamma; Provisional	26	6	115824	5696	3.359E-03	4.693	1.888E-02	1.724
cl09943	Ribosomal L29 HIP.	Ribosomal_L29_HIP. 50S ribosomal protein L29P; Provisional	18	5	115824	5696	3.751E-03	5.648	2.043E-02	1.690
pfam10036	RLL,Putative carnitine deficiency-associated protein	RLL.Putative carnitine deficiency-associated protein. This family of proteins conserved from nematodes to humans is of approximately 250 amino acids. It is purported to be carnitine deficiency-associated protein but this could not be confirmed. It carries a characteristic RLL sequence-motif. The function is unknown.	19	5	115824	5696	4.557E-03	5.351	2.390E-02	1.622
pfam00108	Thiolase_N,Thiolase, N-terminal domain	Thiolase_N.Thiolase, N-terminal domain. Thiolase is reported to be structurally related to beta-ketoacyl synthase (pfam00109), and also chalcone synthase.	71	10	115824	5696	4.601E-03	2.864	2.395E-02	1.621
pfam02866	Ldh_1_C,lactate/malate dehydrogenase, alpha/beta C-terminal domain	Ldh_1_C.lactate/malate dehydrogenase, alpha/beta C-terminal domain. L-lactate dehydrogenases are metabolic enzymes which catalyse the conversion of L-lactate to pyruvate, the last step in anaerobic glycolysis. L-2-hydroxyisocaproate dehydrogenases are also members of the family. Malate dehydrogenases catalyse the interconversion of malate to oxaloacetate. The enzyme participates in the citric acid cycle. L-lactate dehydrogenase is also found as a lens crystallin in bird and crocodile eyes.	134	15	115824	5696	5.309E-03	2.276	2.724E-02	1.565
cl00217	pyrophosphatase.	pyrophosphatase. inorganic pyrophosphatase; Provisional	40	7	115824	5696	6.019E-03	3.558	3.001E-02	1.523
pfam00306	ATP-synt_ab_C,ATP synthase alpha/beta chain, C terminal domain	ATP-synt_ab_C.ATP synthase alpha/beta chain, C terminal domain.	41	7	115824	5696	6.766E-03	3.472	3.350E-02	1.475
cl02666	KU.	KU. This is a single stranded DNA- and ATP-depedent helicase that has a role in chromosome translocation. This is a domain of unknown function C-terminal to its von Willebrand factor A domain, that also occurs in bacterial hypothetical proteins.	79	10	115824	5696	8.897E-03	2.574	4.228E-02	1.374
pfam05911	DUF869,Plant protein of unknown function (DUF869)	DUF869.Plant protein of unknown function (DUF869). This family consists of a number of sequences found in Arabidopsis thaliana, Oryza sativa and Lycopersicon esculentum (Tomato). The function of this family is unknown.	92	11	115824	5696	9.079E-03	2.431	4.285E-02	1.368

pfam04716	ETC_C1_NDUFA5,ETC complex I subunit conserved region	ETC_C1_NDUFA5.ETC complex I subunit conserved region. Family of eukaryotic NADH-ubiquinone oxidoreductase subunits (EC:1.6.5.3) (EC:1.6.99.3) from complex I of the electron transport chain initially identified in Neurospora crassa as a 29.9 kDa protein. The conserved region is found at the N-terminus of the member proteins.	7	3	115824	5696	9.646E-03	8.715	4.493E-02	1.347
pfam09340	NuA4,Histone acetyltransferase subunit NuA4	NuA4.Histone acetyltransferase subunit NuA4. The NuA4 histone acetyltransferase (HAT) multisubunit complex is responsible for acetylation of histone H4 and H2A N-terminal tails in yeast. NuA4 complexes are highly conserved in eukaryotes and play primary roles in transcription, cellular response to DNA damage, and cell cycle control.	7	3	115824	5696	9.646E-03	8.715	4.523E-02	1.345
pfam02172	KIX,KIX domain	KIX.KIX domain. CBP and P300 bind to the CREB via a domain known as KIX. The KIX domain of CBP also binds to transactivation domains of other nuclear factors including Myb and Jun.	15	4	115824	5696	1.063E-02	5.422	4.825E-02	1.316
cl11603	Basic,Myogenic Basic domain	Basic.Myogenic Basic domain. This basic domain is found in the MyoD family of muscle specific proteins that control muscle development. The bHLH region of the MyoD family includes the basic domain and the Helix-loop-helix (HLH) motif. The bHLH region mediates specific DNA binding. With 12 residues of the basic domain involved in DNA binding. The basic domain forms an extended alpha helix in the structure.	15	4	115824	5696	1.063E-02	5.422	4.856E-02	1.314

Underrepresented

domainID	Domain Name	Doman Description	n	m	N	M	p-value	FC	FDR	-log10(FDR)
cl09925	PKc_like,Protein Kinases, catalytic domain	PKc_like.Protein Kinases, catalytic domain. lipopolysaccharide core heptose(I) kinase RfaP; Provisional	11908	66	115824	5696	6.090E-151	0.113	4.310E-148	147.366
pfam00069	Pkinase,Protein kinase domain	Pkinase.Protein kinase domain.	9227	49	115824	5696	9.510E-121	0.108	3.370E-118	117.472
pfam12128	DUF3584,Protein of unknown function (DUF3584)	DUF3584.Protein of unknown function (DUF3584). This protein is found in bacteria and eukaryotes. Proteins in this family are typically between 943 to 1234 amino acids in length. There are two conserved sequence motifs: GKT and YLP.	7428	34	115824	5696	2.010E-103	0.093	4.740E-101	100.324
pfam07714	Pkinase Tyr,Protein tyrosine kinase	Pkinase_Tyr.Protein tyrosine kinase.	7152	42	115824	5696	2.880E-91	0.119	5.100E-89	88.292
pfam02463	SMC_N,RecF/RecN/SMC N terminal domain	SMC_N.RecF/RecN/SMC N terminal domain. This domain is found at the N terminus of SMC proteins. The SMC (structural maintenance of chromosomes) superfamily proteins have ATP-binding domains at the N- and C-termini, and two extended coiled-coil domains separated by a hinge in the middle. The eukaryotic SMC proteins form two kind of heterodimers: the SMC1/SMC3 and the SMC2/SMC4 types. These heterodimers constitute an essential part of higher order complexes, which are involved in chromatin and DNA dynamics. This family also includes the RecF and RecN proteins that are involved in DNA metabolism and recombination.	6987	49	115824	5696	1.470E-82	0.143	2.080E-80	79.682
pfam01576	Myosin_tail_1,Myosin tail	Myosin_tail_1.Myosin tail. The myosin molecule is a multi-subunit complex made up of two heavy chains and four light chains it is a fundamental contractile protein found in all eukaryote cell types. This family consists of the coiled-coil myosin heavy chain tail region. The coiled-coil is composed of the tail from two molecules of myosin. These can then assemble into the macromolecular thick filament. The coiled-coil region provides the structural backbone the thick filament.	5263	44	115824	5696	1.260E-57	0.170	1.490E-55	54.827

Table S2

cl00273	PH-like,Pleckstrin homology-like domain	PH-like.Pleckstrin homology-like domain. AIDA-1b Phosphotyrosine-binding (PTB) domain. AIDA-1b is an amyloid-beta precursor protein interacting protein. It consists of ankyrin repeats, a SAM domain and a C-terminal PTB domain. PTB domains have a PH-like fold and are found in various eukaryotic signaling molecules. They were initially identified based upon their ability to recognize phosphorylated tyrosine residues In contrast to SH2 domains, which recognize phosphotyrosine and adjacent carboxy-terminal residues, PTB-domain binding specificity is conferred by residues amino-terminal to the phosphotyrosine. More recent studies have found that some types of PTB domains can bind to peptides which are not tyrosine phosphorylated or lack tyrosine residues altogether.	3956	25	115824	5696	3.120E-50	0.129	3.160E-48	47.500
cl02567	WD40.	WD40. Note that these repeats are permuted with respect to the structural repeats (blades) of the beta propeller domain.	3888	25	115824	5696	3.780E-49	0.131	3.350E-47	46.475
cl00286	Motor_domain.	Motor_domain. ATPase; molecular motor. Muscle contraction consists of a cyclical interaction between myosin and actin. The core of the myosin structure is similar in fold to that of kinesin.	3065	13	115824	5696	2.350E-45	0.086	1.850E-43	42.733
pfam05557	MAD,Mitotic checkpoint protein	MAD.Mitotic checkpoint protein. This family consists of several eukaryotic mitotic checkpoint (Mitotic arrest deficient or MAD) proteins. The mitotic spindle checkpoint monitors proper attachment of the bipolar spindle to the kinetochores of aligned sister chromatids and causes a cell cycle arrest in prometaphase when failures occur. Multiple components of the mitotic spindle checkpoint have been identified in yeast and higher eukaryotes. In S.cerevisiae, the existence of a Mad1-dependent complex containing Mad2, Mad3, Bub3 and Cdc20 has been demonstrated.	2834	11	115824	5696	4.520E-43	0.079	3.200E-41	40.495
cl10444	Ras_like_GTPase.	Ras_like_GTPase. Members of this protein family are a GTPase associated with ribosome biogenesis, typified by YsxC from Bacillus subutilis. The family is widely but not universally distributed among bacteria. Members commonly are called EngB based on homology to EngA, one of several other GTPases of ribosome biogenesis. Cutoffs as set find essentially all bacterial members, but also identify large numbers of eukaryotic (probably organellar) sequences. This protein is found in about 80 percent of bacterial genomes.	4853	60	115824	5696	1.110E-40	0.251	7.140E-39	38.146
cl02553	Peptidase_C19.	Peptidase_C19. A subfamily of peptidase C19. Peptidase C19 contains ubiquitinyl hydrolases. They are intracellular peptidases that remove ubiquitin molecules from polyubiquinated peptides by cleavage of isopeptide bonds. They hydrolyze bonds involving the carboxyl group of the C-terminal Gly residue of ubiquitin. The purpose of the de-ubiquitination is thought to be editing of the ubiquitin conjugates, which could rescue them from degradation, as well as recycling of the ubiquitin. The ubiquitin/proteasome system is responsible for most protein turnover in the mammalian cell, and with over 50 members, family C19 is one of the largest families of peptidases in the human genome.	1858	6	115824	5696	4.450E-30	0.066	2.630E-28	27.580
cl00084	homeodomain.	homeodomain. DNA-binding factors that are involved in the transcriptional regulation of key developmental processes	1406	1	115824	5696	8.490E-28	0.014	4.620E-26	25.335
cl02529	ANK.	ANK. Ankyrin repeats generally consist of a beta, alpha, alpha, beta order of secondary structures. The repeats associate to form a higher order structure.	1645	6	115824	5696	4.910E-26	0.074	2.480E-24	23.606
pfam10174	Cast,RIM-binding protein of the cytomatrix active zone	Cast.RIM-binding protein of the cytomatrix active zone. This is a family of proteins that form part of the CAZ (cytomatrix at the active zone) complex which is involved in determining the site of synaptic vesicle fusion. The C-terminus is a PDZ-binding motif that binds directly to RIM (a small G protein Rab-3A effector). The family also contains four coiled-coil domains.	2261	19	115824	5696	1.090E-25	0.171	5.140E-24	23.289
cl02468	CA.	CA. This cadherin domain is usually the most N-terminal copy of the domain.	1296	1	115824	5696	1.290E-25	0.016	5.710E-24	23.243

Table S2

cl09099	P-loop NTPase,P-loop containing Nucleoside Triphosphate Hydrolases	P-loop NTPase.P-loop containing Nucleoside Triphosphate Hydrolases. This domain family is found in bacteria and archaea, and is approximately 50 amino acids in length.	3199	47	115824	5696	1.460E-23	0.299	6.080E-22	21.216
pfam05622	HOOK,HOOK protein	HOOK.HOOK protein. This family consists of several HOOK1, 2 and 3 proteins from different eukaryotic organisms. The different members of the human gene family are HOOK1, HOOK2 and HOOK3. Different domains have been identified in the three human HOOK proteins, and it was demonstrated that the highly conserved NH2-domain mediates attachment to microtubules, whereas the central coiled-coil motif mediates homodimerization and the more divergent C-terminal domains are involved in binding to specific organelles (organelle-binding domains). It has been demonstrated that endogenous HOOK3 binds to Golgi membranes, whereas both HOOK1 and HOOK2 are localized to discrete but unidentified cellular structures. In mice the Hook1 gene is predominantly expressed in the testis. Hook1 function is necessary for the correct positioning of microtubular structures within the haploid germ cell. Disruption of Hook1 function in mice causes abnormal sperm head shape and fragile attachment of the	1725	12	115824	5696	6.010E-22	0.141	2.360E-20	19.627
cl12013	BAR,The Bin/Amphiphysin/Rvs (BAR) domain, a dimerization module that binds membranes and detects membrane curvature	BAR.The Bin/Amphiphysin/Rvs (BAR) domain, a dimerization module that binds membranes and detects membrane curvature. F-BAR domains are dimerization modules that bind and bend membranes and are found in proteins involved in membrane dynamics and actin reorganization. Fer (Fes related) is a cytoplasmic (or nonreceptor) tyrosine kinase expressed in a wide variety of tissues, and is found to reside in both the cytoplasm and the nucleus. It plays important roles in neuronal polarization and neurite development, cytoskeletal reorganization, cell migration, growth factor signaling, and the regulation of cell-cell interactions mediated by adherens junctions and focal adhesions. Fer kinase also regulates cell cycle progression in malignant cells. It contains an N-terminal F-BAR domain, an SH2 domain, and a C-terminal catalytic kinase domain. F-BAR domains form banana-shaped dimers with a positively-charged concave surface that binds to negatively-charged lipid membranes. They can induce	1865	20	115824	5696	1.350E-18	0.218	5.030E-17	16.298
cl12029	DEXDc.	DEXDc. CRISPR (Clustered Regularly Interspaced Short Palindromic Repeats) and associated Cas proteins comprise a system for heritable host defense by prokaryotic cells against phage and other foreign DNA; Diverged DNA helicase Cas3; signature gene for Type I and subtype I-D	2240	30	115824	5696	1.600E-18	0.272	5.660E-17	16.247
pfam06160	EzrA,Septation ring formation regulator, EzrA	EzrA.Septation ring formation regulator, EzrA. During the bacterial cell cycle, the tubulin-like cell-division protein FtsZ polymerizes into a ring structure that establishes the location of the nascent division site. EzrA modulates the frequency and position of FtsZ ring formation.	989	2	115824	5696	7.390E-18	0.041	2.490E-16	15.604
cl00057	vWFA.	vWFA. hypothetical protein; Provisional	1206	6	115824	5696	9.600E-18	0.101	3.090E-16	15.510
cl02570	RhoGAP.	RhoGAP. This is a yeast domain of unknown function.	894	1	115824	5696	2.040E-17	0.023	6.280E-16	15.202
cl00137	SERPIN.	SERPIN. serine protease inhibitor-like protein; Provisional	1175	6	115824	5696	4.270E-17	0.104	1.260E-15	14.900
pfam05483	SCP-1,Synaptonemal complex protein 1 (SCP-1)	SCP-1.Synaptonemal complex protein 1 (SCP-1). Synaptonemal complex protein 1 (SCP-1) is the major component of the transverse filaments of the synaptonemal complex. Synaptonemal complexes are structures that are formed between homologous chromosomes during meiotic prophase.	1622	17	115824	5696	1.800E-16	0.213	5.100E-15	14.292
pfam00201	UDPGT,UDP-glucoronosyl and UDP-glucosyl transferase	UDPGT.UDP-glucoronosyl and UDP-glucosyl transferase.	840	1	115824	5696	3.150E-16	0.024	8.580E-15	14.067
cl02571	RhoGEF.	RhoGEF. Guanine nucleotide exchange factor for Rho/Rac/Cdc42-like GTPases Also called Dbl-homologous (DH) domain. It appears that pfam00169 domains invariably occur C-terminal to RhoGEF/DH domains.	951	3	115824	5696	5.470E-16	0.064	1.430E-14	13.845
cl10013	Glycosyltransferase_GTB_type.	Glycosyltransferase_GTB_type. ADP-heptose:LPS heptosyl transferase I; Provisional	982	4	115824	5696	1.790E-15	0.083	4.530E-14	13.344
cl12078	p450,Cytochrome P450	p450.Cytochrome P450. fatty acid omega-hydroxylase; Provisional	742	1	115824	5696	3.140E-14	0.027	7.670E-13	12.115

Table S2

cl11394	Glyco_tranf_GTA_type,Glycosyltransferase family A (GT-A) includes diverse families of glycosyl transferases with a common GT-A type structural fold	Glyco_tranf_GTA_type.Glycosyltransferase family A (GT-A) includes diverse families of glycosyl transferases with a common GT-A type structural fold. This gene is one of the glycosyl transferases involved in the biosynthesis of colanic acid, an exopolysaccharide expressed in Enterobacteraceae species.	956	5	115824	5696	4.200E-14	0.106	9.910E-13	12.004
cl12011	AdoMet_MTases.	AdoMet_MTases. This model recognizes the CbiT methylase which is responsible, in part (along with CbiE), for methylating precorrin-6y (or cobalt-precorrin-6y) at both the 5 and 15 positions as well as the concomitant decarbozylation at C-12. In many organisms, this protein is fused to the CbiE subunit. The fused protein, when found in organisms catalyzing the oxidative version of the cobalamin biosynthesis pathway, is called CobL.	1034	7	115824	5696	8.330E-14	0.138	1.900E-12	11.721
cl12031	Esterase lipase.	Esterase_lipase. putative hydrolase; Provisional	889	4	115824	5696	9.820E-14	0.091	2.170E-12	11.664
cl00117	PDZ.	PDZ. PDZ domains are found in diverse signaling proteins.	787	3	115824	5696	9.370E-13	0.078	2.010E-11	10.697
pfam07888	CALCOCO1,Calcium binding and coiled-coil domain (CALCOCO1) like	CALCOCO1.Calcium binding and coiled-coil domain (CALCOCO1) like. Proteins found in this family are similar to the coiled-coil transcriptional coactivator protein coexpressed by Mus musculus (CoCoA/CALCOCO1). This protein binds to a highly conserved N-terminal domain of p160 coactivators, such as GRIP1, and thus enhances transcriptional activation by a number of nuclear receptors. CALCOCO1 has a central coiled-coil region with three leucine zipper motifs, which is required for its interaction with GRIP1 and may regulate the autonomous transcriptional activation activity of the C-terminal region.	1086	10	115824	5696	2.020E-12	0.187	4.210E-11	10.376
cl00053	PTPc.	PTPc. protein tyrosine phosphatase; Provisional	748	3	115824	5696	3.800E-12	0.082	7.690E-11	10.114
pfam03028	Dynein_heavy,Dynein heavy chain	Dynein_heavy.Dynein heavy chain. This family represents the C-terminal region of dynein heavy chain. The chain also contains ATPase activity and microtubule binding ability and acts as a motor for the movement of organelles and vesicles along microtubules. Dynein is also involved in cilia and flagella movement. The dynein subunit consists of at least two heavy chains and a number of intermediate and light chains (see pfam01221).	628	1	115824	5696	6.670E-12	0.032	1.310E-10	9.883
pfam02029	Caldesmon,Caldesmon	Caldesmon.Caldesmon.	699	3	115824	5696	3.440E-11	0.087	6.580E-10	9.182
pfam09770	PAT1,Topoisomerase II-associated protein PAT1	PAT1.Topoisomerase II-associated protein PAT1. Members of this family are necessary for accurate chromosome transmission during cell division.	804	6	115824	5696	1.640E-10	0.152	3.060E-09	8.514
pfam07111	HCR,Alpha helical coiled-coil rod protein (HCR)	HCR.Alpha helical coiled-coil rod protein (HCR). This family consists of several mammalian alpha helical coiled-coil rod HCR proteins. The function of HCR is unknown but it has been implicated in psoriasis in humans and is thought to affect keratinocyte proliferation.	710	4	115824	5696	1.860E-10	0.115	3.380E-09	8.471
pfam03154	Atrophin-1,Atrophin-1 family	Atrophin-1.Atrophin-1 family. Atrophin-1 is the protein product of the dentatorubral-pallidoluysian atrophy (DRPLA) gene. DRPLA OMIM:125370 is a progressive neurodegenerative disorder. It is caused by the expansion of a CAG repeat in the DRPLA gene on chromosome 12p. This results in an extended polyglutamine region in atrophin-1, that is thought to confer toxicity to the protein, possibly through altering its interactions with other proteins. The expansion of a CAG repeat is also the underlying defect in six other neurodegenerative disorders, including Huntingtons disease. One interaction of expanded polyglutamine repeats that is thought to be pathogenic is that with the short glutamine repeat in the transcriptional coactivator CREB binding protein, CBP. This interaction draws CBP away from its usual nuclear location to the expanded polyglutamine repeat protein aggregates that are characteristic of the polyglutamine neurodegenerative disorders. This interferes with CBP-	748	5	115824	5696	3.150E-10	0.136	5.580E-09	8.253
pfam00501	AMP-binding,AMP-binding enzyme	AMP-binding.AMP-binding enzyme.	761	6	115824	5696	9.170E-10	0.160	1.550E-08	7.810
pfam09726	Macoilin,Transmembrane protein	Macoilin.Transmembrane protein. This entry is a highly conserved protein present in eukaryotes.	562	2	115824	5696	1.380E-09	0.072	2.270E-08	7.644

Table S2

cl00281	metallo-dependent_hydrolases.	metallo-dependent_hydrolases. imidazolonepropionase; Provisional	700	5	115824	5696	1.740E-09	0.145	2.800E-08	7.553
cl00065	FN3.	FN3. One of three types of internal repeat within the plasma protein, fibronectin. The tenth fibronectin type III repeat contains a RGD cell recognition sequence in a flexible loop between 2 strands. Type III modules are present in both extracellular and intracellular proteins.	504	1	115824	5696	1.930E-09	0.040	3.040E-08	7.517
pfam05110	AF-4,AF-4 proto-oncoprotein	AF-4.AF-4 proto-oncoprotein. This family consists of AF4 (Proto-oncogene AF4) and FMR2 (Fragile X E mental retardation syndrome) nuclear proteins. These proteins have been linked to human diseases such as acute lymphoblastic leukaemia and mental retardation. The family also contains a Drosophila AF4 protein homologue Lilliputian which contains an AT-hook domain. Lilliputian represents a novel pair-rule gene that acts in cytoskeleton regulation, segmentation and morphogenesis in Drosophila.	590	3	115824	5696	3.840E-09	0.103	5.910E-08	7.228
cl00357	NAT_SF,N-Acyltransferase superfamily: Various enzymes that characteristically catalyze the transfer of an acyl group to a substrate	NAT_SF.N-Acyltransferase superfamily: Various enzymes that characteristically catalyze the transfer of an acyl group to a substrate. Members of this family belong to the GNAT family (pfam00583), in which numerous characterized examples, though not all, are are shown to be N-acetyltransferases or to interact with acetyl-CoA. This family occurs in a sparsely distributed biosynthetic cluster that occurs in Actinobacteria, Cyanobacteria, and Proteobacteria.	472	1	115824	5696	5.530E-09	0.043	8.330E-08	7.079
pfam00685	Sulfotransfer_1,Sulfotransferase domain	Sulfotransfer_1.Sulfotransferase domain.	576	3	115824	5696	8.130E-09	0.106	1.170E-07	6.932
cl09950	SH3.	SH3. SH3 (Src homology 3) domains are often indicative of a protein involved in signal transduction related to cytoskeletal organisation. First described in the Src cytoplasmic tyrosine kinase. The structure is a partly opened beta barrel.	520	2	115824	5696	8.690E-09	0.078	1.230E-07	6.910
cl00490	Exo_endo_phos,Endonuclease/Exonuclease/phosphatase family	Exo_endo_phos.Endonuclease/Exonuclease/phosphatase family. The model brings in reverse transcriptases at scores below 50, model also contains eukaryotic apurinic/apyrimidinic endonucleases which group in the same family	444	1	115824	5696	2.590E-08	0.046	3.600E-07	6.444
cl02563	PX_domain,The Phox Homology domain, a phosphoinositide binding module	PX_domain.The Phox Homology domain, a phosphoinositide binding module. PX domains bind to phosphoinositides.	477	2	115824	5696	5.420E-08	0.085	7.380E-07	6.132
pfam10243	MIP-T3,Microtubule-binding protein MIP-T3	MIP-T3.Microtubule-binding protein MIP-T3. This protein, which interacts with both microtubules and TRAF3 (tumour necrosis factor receptor-associated factor 3), is conserved from worms to humans. The N-terminal region is the microtubule binding domain and is well-conserved; the C-terminal 100 residues, also well-conserved, constitute the coiled-coil region which binds to TRAF3. The central region of the protein is rich in lysine and glutamic acid and carries KKE motifs which may also be necessary for tubulin-binding, but this region is the least well-conserved	733	8	115824	5696	6.480E-08	0.222	8.660E-07	6.062
pfam00176	SNF2_N,SNF2 family N-terminal domain	SNF2_N.SNF2 family N-terminal domain. This domain is found in proteins involved in a variety of processes including transcription regulation (e.g., SNF2, STH1, brahma, MOT1), DNA repair (e.g., ERCC6, RAD16, RAD5), DNA recombination (e.g., RAD54), and chromatin unwinding (e.g., ISWI) as well as a variety of other proteins with little functional information (e.g., lodestar, ETL1).	570	4	115824	5696	7.890E-08	0.143	1.020E-06	5.991
pfam05955	Herpes_gp2,Equine herpesvirus glycoprotein gp2	Herpes_gp2.Equine herpesvirus glycoprotein gp2. This family consists of a number of glycoprotein gp2 sequences from equine herpesviruses.	418	1	115824	5696	7.880E-08	0.049	1.030E-06	5.987
cl02569	RasGAP.	RasGAP. All alpha-helical domain that accelerates the GTPase activity of Ras, thereby switching it into an off position.	401	1	115824	5696	1.660E-07	0.051	2.100E-06	5.678
pfam00102	Y_phosphatase,Protein-tyrosine phosphatase	Y_phosphatase.Protein-tyrosine phosphatase.	491	3	115824	5696	2.860E-07	0.124	3.490E-06	5.457

Table S2

cl02488	SPEC.	SPEC. Spectrin repeats are found in several proteins involved in cytoskeletal structure. These include spectrin, alpha-actinin and dystrophin. The sequence repeat used in this family is taken from the structural repeat in reference. The spectrin repeat forms a three helix bundle. The second helix is interrupted by proline in some sequences. The repeats are defined by a characteristic tryptophan (W) residue at position 17 in helix A and a leucine (L) at 2 residues from the carboxyl end of helix C.	563	5	115824	5696	4.240E-07	0.181	5.090E-06	5.293
cl02544	VHS_ENTH_ANTH.	VHS_ENTH_ANTH. AP180 is an endocytotic accessory proteins that has been implicated in the formation of clathrin-coated pits. The domain is involved in phosphatidylinositol 4,5-bisphosphate binding and is a universal adaptor for nucleation of clathrin coats.	426	2	115824	5696	4.840E-07	0.095	5.710E-06	5.243
cl06868	FNR_like.	FNR_like. Xanthine dehydrogenases, that also bind FAD/NAD, have essentially no similarity.	374	1	115824	5696	5.070E-07	0.054	5.880E-06	5.231
pfam08337	Plexin_cytopl,Plexin cytoplasmic RasGAP domain	Plexin_cytopl.Plexin cytoplasmic RasGAP domain. This family features the C-terminal regions of various plexins. Plexins are receptors for semaphorins, and plexin signalling is important in path finding and patterning of both neurons and developing blood vessels. The cytoplasmic region, which has been called a SEX domain in some members of this family, is involved in downstream signalling pathways, by interaction with proteins such as Rac1, RhoD, Rnd1 and other plexins. This domain acts as a RasGAP domain.	365	1	115824	5696	7.370E-07	0.056	8.420E-06	5.075
cl00081	HLH.	HLH. Helix-loop-helix domain, found in specific DNA- binding proteins that act as transcription factors; 60-100 amino acids long. A DNA-binding basic region is followed by two alpha-helices separated by a variable loop region; HLH forms homo- and heterodimers, dimerization creates a parallel, left-handed, four helix bundle; the basic region N-terminal to the first amphipathic helix mediates high-affinity DNA-binding; there are several groups of HLH proteins: those (E12/E47) which bind specific hexanucleotide sequences such as E-box (5-CANNTG-3) or StRE 5-ATCACCCCAC-3), those lacking the basic domain (Emc, Id) function as negative regulators since they fail to bind DNA, those (hairy, E(spl), deadpan) which repress transcription although they can bind specific hexanucleotide sequences such as N-box (5-CACGc/aG-3), those which have a COE domain (Collier/Olf-1/EBF) which is involved in both in dimerization and in DNA binding, and those which bind pentanucleotides ACGTG or GCGTG and have a PAS domain which allows the dimerization between PAS proteins, the binding of small molecules (e.g., dioxin), and interactions	580	6	115824	5696	1.080E-06	0.210	1.210E-05	4.917
pfam09606	Med15,ARC105 or Med15 subunit of Mediator complex non-fungal	Med15.ARC105 or Med15 subunit of Mediator complex non-fungal. The approx. 70 residue Med15 domain of the ARC-Mediator co-activator is a three-helix bundle with marked similarity to the KIX domain. The sterol regulatory element binding protein (SREBP) family of transcription activators use the ARC105 subunit to activate target genes in the regulation of cholesterol and fatty acid homeostasis. In addition, Med15 is a critical transducer of gene activation signals that control early metazoan development.	502	4	115824	5696	1.260E-06	0.162	1.390E-05	4.857
cl00030	CH.	CH. This domain is the N-terminal CH domain from the CAMSAP proteins.	692	9	115824	5696	1.400E-06	0.264	1.520E-05	4.818
cl02614	SPRY,SPRY domain	SPRY.SPRY domain. Domain of unknown function. Distant homologues are domains in butyrophilin/marenostrin/pyrin homologues.	549	6	115824	5696	4.080E-06	0.222	4.310E-05	4.366
cl02429	TPR.	TPR. This Pfam entry includes outlying Tetratricopeptide-like repeats (TPR) that are not matched by pfam00515.	760	12	115824	5696	4.510E-06	0.321	4.700E-05	4.328

Table S2

cl08267	ISOPREN_C2_like.	ISOPREN_C2_like. Squalene cyclase (SQCY) domain; found in class II terpene cyclases that have an alpha 6 - alpha 6 barrel fold. Squalene cyclase (SQCY) and 2,3-oxidosqualene cyclase (OSQCY) are integral membrane proteins that catalyze a cationic cyclization cascade converting linear triterpenes to fused ring compounds. Bacterial SQCY catalyzes the convertion of squalene to hopene or diplopterol. Eukaryotic OSQCY transforms the 2,3-epoxide of squalene to compounds such as, lanosterol (a metabolic precursor of cholesterol and steroid hormones) in mammals and fungi or, cycloartenol in plants. Deletion of a single glycine residue of Alicyclobacillus acidocaldarius SQCY alters its substrate specificity into that of eukaryotic OSQCY. Both enzymes have a second minor domain, which forms an alpha-alpha barrel that is inserted into the major domain. This group also contains SQCY-like archael sequences and some bacterial SQCYs which	316	1	115824	5696	6.950E-06	0.064	7.130E-05	4.147
pfam05109	Herpes_BLLF1,Herpes virus major outer envelope glycoprotein (BLLF1)	Herpes_BLLF1.Herpes virus major outer envelope glycoprotein (BLLF1). This family consists of the BLLF1 viral late glycoprotein, also termed gp350/220. It is the most abundantly expressed glycoprotein in the viral envelope of the Herpesviruses and is the major antigen responsible for stimulating the production of neutralising antibodies in vivo.	322	1	115824	5696	7.420E-06	0.063	7.400E-05	4.131
cl00138	SH2.	SH2. Src homology 2 domains bind phosphotyrosine-containing polypeptides via 2 surface pockets. Specificity is provided via interaction with residues that are distinct from the phosphotyrosine. Only a single occurrence of a SH2 domain has been found in S. cerevisiae.	596	8	115824	5696	1.050E-05	0.273	1.005E-04	3.998
cl00082	HMG-box.	HMG-box. high mobility group protein; Provisional	627	9	115824	5696	1.170E-05	0.292	1.104E-04	3.957
pfam00373	FERM_M,FERM central domain	FERM_M.FERM central domain. This domain is the central structural domain of the FERM domain.	354	2	115824	5696	1.270E-05	0.115	1.168E-04	3.933
pfam03344	Daxx,Daxx Family	Daxx.Daxx Family. The Daxx protein (also known as the Fas-binding protein) is thought to play a role in apoptosis, but precise role played by Daxx remains to be determined. Daxx forms a complex with Axin.	304	1	115824	5696	1.520E-05	0.067	1.380E-04	3.860
cl00047	CAP_ED.	CAP_ED. Catabolite gene activator protein (CAP) is a prokaryotic homologue of eukaryotic cNMP-binding domains, present in ion channels, and cNMP-dependent kinases.	278	1	115824	5696	4.580E-05	0.073	3.954E-04	3.403
cl00155	UBQ,Ubiquitin-like proteins	UBQ.Ubiquitin-like proteins. ubiquitin; Provisional	959	21	115824	5696	4.970E-05	0.445	4.239E-04	3.373
pfam00928	Adap_comp_sub,Adaptor complexes medium subunit family	Adap_comp_sub.Adaptor complexes medium subunit family. This family also contains members which are coatomer subunits.	318	2	115824	5696	5.220E-05	0.128	4.400E-04	3.357
cl00135	SEC14.	SEC14. The original profile has been extended to include the carboxyl domain from the known structure of Sec14.	321	2	115824	5696	5.310E-05	0.127	4.423E-04	3.354
cl09501	EFh.	EFh. The EF-hands can be divided into two classes: signaling proteins and buffering/transport proteins. The first group is the largest and includes the most well-known members of the family such as calmodulin, troponin C and S100B. These proteins typically undergo a calcium-dependent conformational change which opens a target binding site. The latter group is represented by calbindin D9k and do not undergo calcium dependent conformational changes.	355	3	115824	5696	7.600E-05	0.172	6.046E-04	3.219
cl12021	Guanylate kin,Guanylate kinase	Guanylate_kin.Guanylate kinase. guanylate kinase; Provisional	314	2	115824	5696	7.710E-05	0.130	6.065E-04	3.217
cl00388	Thioredoxin_like,Protein Disulfide Oxidoreductases and Other Proteins with a Thioredoxin fold	Thioredoxin_like.Protein Disulfide Oxidoreductases and Other Proteins with a Thioredoxin fold. This model describes a domain of eukaryotic protein disulfide isomerases, generally found in two copies. The high cutoff for total score reflects the expectation of finding both copies. The domain is similar to thioredoxin but the redox-active disulfide region motif is APWCGHCK.	1550	43	115824	5696	7.460E-05	0.564	6.071E-04	3.217

Table S2

pfam09731	Mitofilin,Mitochondrial inner membrane protein	Mitofilin.Mitochondrial inner membrane protein. Mitofilin controls mitochondrial cristae morphology. Mitofilin is enriched in the narrow space between the inner boundary and the outer membranes, where it forms a homotypic interaction and assembles into a large multimeric protein complex. The first 78 amino acids contain a typical amino-terminal-cleavable mitochondrial presequence rich in positive-charged and hydroxylated residues and a membrane anchor domain. In addition, it has three centrally located coiled coil domains.	459	6	115824	5696	9.790E-05	0.266	7.453E-04	3.128
cl02556	Bromodomain.	Bromodomain. Bromodomains are 110 amino acid long domains, that are found in many chromatin associated proteins. Bromodomains can interact specifically with acetylated lysine.	595	10	115824	5696	9.760E-05	0.342	7.511E-04	3.124
cl00042	CASc.	CASc. Cysteine aspartases that mediate programmed cell death (apoptosis). Caspases are synthesised as zymogens and activated by proteolysis of the peptide backbone adjacent to an aspartate. The resulting two subunits associate to form an (alpha)2(beta)2-tetramer which is the active enzyme. Activation of caspases can be mediated by other caspase homologues.	265	1	115824	5696	1.022E-04	0.077	7.701E-04	3.113
cl02464	ArfGap,Putative GTPase activating protein for Arf	ArfGap.Putative GTPase activating protein for Arf. Putative zinc fingers with GTPase activating proteins (GAPs) towards the small GTPase, Arf. The GAP of ARD1 stimulates GTPase hydrolysis for ARD1 but not ARFs.	304	2	115824	5696	1.080E-04	0.134	8.046E-04	3.094
cl00283	ADP_ribosyl.	ADP_ribosyl. Members of this family, which are found in prokaryotic exotoxin A, catalyse the transfer of ADP ribose from nicotinamide adenine dinucleotide (NAD) to elongation factor-2 in eukaryotic cells, with subsequent inhibition of protein synthesis.	239	1	115824	5696	3.031E-04	0.085	2.167E-03	2.664
cl02434	CNH,CNH domain	CNH.CNH domain. Unpublished observations.	280	2	115824	5696	3.157E-04	0.145	2.213E-03	2.655
cl00298	Peptidase_C1.	Peptidase_C1. This family is closely related to the Peptidase_C1 family pfam00112, containing several prokaryotic and eukaryotic aminopeptidases and bleomycin hydrolases.	226	1	115824	5696	4.135E-04	0.090	2.788E-03	2.555
cl02844	Arrestin_N,Arrestin (or S-antigen), N-terminal domain	Arrestin_N.Arrestin (or S-antigen), N-terminal domain. Ig-like beta-sandwich fold. Scop reports duplication with N-terminal domain.	225	1	115824	5696	4.117E-04	0.090	2.802E-03	2.552
cl09931	NADB_Rossmann,Rossmann-fold NAD(P)(+)-binding proteins	NADB_Rossmann.Rossmann-fold NAD(P)(+)-binding proteins. Members of this protein subfamily are putative oxidoreductases belonging to the larger SDR family. Members of the present subfamily may occur several to a genome and are largely restricted to genomes that contain members of families TIGR03962, TIGR03967, and TIGR03969. Many members have been annotated by homology as carveol dehydrogenases.	1279	36	115824	5696	4.713E-04	0.572	3.119E-03	2.506
pfam00702	Hydrolase,haloacid dehalogenase-like hydrolase	Hydrolase.haloacid dehalogenase-like hydrolase. This family are structurally different from the alpha/ beta hydrolase family (pfam00561). This family includes L-2-haloacid dehalogenase, epoxide hydrolases and phosphatases. The structure of the family consists of two domains. One is an inserted four helix bundle, which is the least well conserved region of the alignment, between residues 16 and 96 of (S)-2-haloacid dehalogenase from Pseudomonas sp. CBS3. The rest of the fold is composed of the core alpha/beta domain.	299	3	115824	5696	5.706E-04	0.204	3.741E-03	2.427
pfam01496	V_ATPase_I,V-type ATPase 116kDa subunit family	V_ATPase_I.V-type ATPase 116kDa subunit family. This family consists of the 116kDa V-type ATPase (vacuolar (H+)-ATPases) subunits, as well as V-type ATP synthase subunit i. The V-type ATPases family are proton pumps that acidify intracellular compartments in eukaryotic cells for example yeast central vacuoles, clathrin-coated and synaptic vesicles. They have important roles in membrane trafficking processes. The 116kDa subunit (subunit a) in the V-type ATPase is part of the V0 functional domain responsible for proton transport. The a subunit is a transmembrane glycoprotein with multiple putative transmembrane helices it has a hydrophilic amino terminal and a hydrophobic carboxy terminal. It has roles in proton transport and assembly of the V-type ATPase complex. This subunit is encoded by two	295	3	115824	5696	8.116E-04	0.207	5.224E-03	2.282

Table S2

pfam00481	PP2C,Protein phosphatase 2C	PP2C.Protein phosphatase 2C. Protein phosphatase 2C is a Mn++ or Mg++ dependent protein serine/threonine phosphatase.	257	2	115824	5696	9.336E-04	0.158	5.849E-03	2.233
pfam04147	Nop14,Nop14-like family	Nop14.Nop14-like family. Emg1 and Nop14 are novel proteins whose interaction is required for the maturation of the 18S rRNA and for 40S ribosome production.	320	4	115824	5696	1.337E-03	0.254	8.158E-03	2.088
pfam00769	ERM,Ezrin/radixin/moesin family	ERM.Ezrin/radixin/moesin family. This family of proteins contain a band 4.1 domain (pfam00373), at their amino terminus. This family represents the rest of these proteins.	274	3	115824	5696	1.549E-03	0.223	9.376E-03	2.028
pfam01593	Amino_oxidase,Flavin containing amine oxidoreductase	Amino_oxidase.Flavin containing amine oxidoreductase. This family consists of various amine oxidases, including maze polyamine oxidase (PAO) and various flavin containing monoamine oxidases (MAO). The aligned region includes the flavin binding site of these enzymes. The family also contains phytoene dehydrogenases and related enzymes. In vertebrates MAO plays an important role regulating the intracellular levels of amines via there oxidation; these include various neurotransmitters, neurotoxins and trace amines. In lower eukaryotes such as aspergillus and in bacteria the main role of amine oxidases is to provide a source of ammonium. PAOs in plants, bacteria and protozoa oxidase spermidine and spermine to an aminobutyral, diaminopropane and hydrogen peroxide and are involved in the catabolism of polyamines. Other members of this family include tryptophan 2-monooxygenase, putrescine oxidase, corticosteroid binding proteins and antibacterial	195	1	115824	5696	1.836E-03	0.104	1.092E-02	1.962
cl00120	PP2Cc.	PP2Cc. Protein phosphatase 2c; Provisional	288	4	115824	5696	3.353E-03	0.282	1.899E-02	1.721
cl02305	Snf7,Snf7	Snf7.Snf7. SNF-7-like protein; Provisional	221	2	115824	5696	3.611E-03	0.184	1.982E-02	1.703
cl02652	MIF4G,MIF4G domain	MIF4G.MIF4G domain. Also occurs in NMD2p and CBP80. The domain is rich in alpha-helices and may contain multiple alpha-helical repeats. In eIF4G, this domain binds eIF4A, eIF3, RNA and DNA. Ponting (TiBS) Novel eIF4G domain homologues (in press)	221	2	115824	5696	3.611E-03	0.184	1.997E-02	1.700
cl12071	TFIIFa.	TFIIFa. Transcription initiation factor IIF, alpha subunit (TFIIF-alpha) or RNA polymerase II-associating protein 74 (RAP74) is the large subunit of transcription factor IIF (TFIIF), which is essential for accurate initiation and stimulates elongation by RNA polymerase II.	179	1	115824	5696	3.847E-03	0.114	2.079E-02	1.682
pfam09756	DDRGK,DDRGK domain	DDRGK.DDRGK domain. This is a family of proteins of approximately 300 residues, found in plants and vertebrates. They contain a highly conserved DDRGK motif.	180	1	115824	5696	3.905E-03	0.113	2.094E-02	1.679
cl00187	Fascin.	Fascin. This family consists of several eukaryotic fascin or singed proteins. The fascins are a structurally unique and evolutionarily conserved group of actin cross-linking proteins. Fascins function in the organisation of two major forms of actin-based structures: dynamic, cortical cell protrusions and cytoplasmic microfilament bundles. The cortical structures, which include filopodia, spikes, lamellipodial ribs, oocyte microvilli and the dendrites of dendritic cells, have roles in cell-matrix adhesion, cell interactions and cell migration, whereas the cytoplasmic actin bundles appear to participate in cell architecture. Dictyostelium hisactophilin, another actin-binding protein, is a submembranous pH sensor that signals slight changes of the H+ concentration to actin by inducing actin polymerisation and binding to microfilaments only at pH values below seven. Members of this family are histidine	181	1	115824	5696	3.973E-03	0.112	2.115E-02	1.675
cl00549	ABC_membrane,ABC transporter transmembrane region	ABC_membrane.ABC transporter transmembrane region. microcin B17 transporter; Reviewed	253	3	115824	5696	4.261E-03	0.241	2.251E-02	1.648
cl11966	NT_Pol-beta-like,Nucleotidyltransferase (NT) domain of DNA polymerase beta and similar proteins	NT_Pol-beta-like.Nucleotidyltransferase (NT) domain of DNA polymerase beta and similar proteins. aminoglycoside resistance protein; Provisional	211	2	115824	5696	5.005E-03	0.193	2.586E-02	1.587

Table S2

cl10010	CBS_pair.	CBS_pair. The CBS domain, named after human CBS, is a small domain originally identified in cystathionine beta-synthase and is subsequently found in a wide range of different proteins. CBS domains usually occur in tandem repeats. They associate to form a so-called Bateman domain or a CBS pair based on crystallographic studies in bacteria. The CBS pair was used as a basis for this cd hierarchy since the human CBS proteins can adopt the typical core structure and form an intramolecular CBS pair. The interface between the two CBS domains forms a cleft that is a potential ligand binding site. The CBS pair coexists with a variety of other functional domains and this has been used to help in its classification here. It has been proposed that the CBS domain may play a regulatory role, although its exact function is unknown. Mutations of conserved residues within this domain are associated with a variety of human hereditary diseases, including congenital myotonia, idiopathic generalized epilepsy, hypercalciuric nephrolithiasis, and classic Bartter syndrome (CLC chloride channel family members), Wolff-Parkinson-White syndrome (gamma 2 subunit of AMP-activated protein kinase), retinitis pigmentosa (IMP dehydrogenase-1), and homocystinuria (cystathionine beta-[illegible]	168	1	115824	5696	5.383E-03	0.121	2.742E-02	1.562
pfam06920	Ded_cyto,Dedicator of cytokinesis	Ded_cyto.Dedicator of cytokinesis. This family represents a conserved region approximately 200 residues long within a number of eukaryotic dedicator of cytokinesis proteins. These are potential guanine nucleotide exchange factors, which activate some small GTPases by exchanging bound GDP for free GTP.	240	3	115824	5696	5.737E-03	0.254	2.901E-02	1.537
pfam09728	Taxilin,Myosin-like coiled-coil protein	Taxilin.Myosin-like coiled-coil protein. Taxilin contains an extraordinarily long coiled-coil domain in its C-terminal half and is ubiquitously expressed. It is a novel binding partner of several syntaxin family members and is possibly involved in Ca2+-dependent exocytosis in neuroendocrine cells. Gamma-taxilin, described as leucine zipper protein Factor Inhibiting ATF4-mediated Transcription (FIAT), localizes to the nucleus in osteoblasts and dimerizes with ATF4 to form inactive dimers, thus inhibiting ATF4-mediated transcription.	244	3	115824	5696	5.872E-03	0.250	2.948E-02	1.530
cl11528	ACTIN.	ACTIN. actin-like protein; Provisional	201	2	115824	5696	6.956E-03	0.202	3.420E-02	1.466
cl00067	FYVE.	FYVE. The FYVE zinc finger is named after four proteins that it has been found in: Fab1, YOTB/ZK632.12, Vac1, and EEA1. The FYVE finger has been shown to bind two Zn++ ions. The FYVE finger has eight potential zinc coordinating cysteine positions. Many members of this family also include two histidines in a motif R+HHC+XCG, where + represents a charged residue and X any residue. We have included members which do not conserve these histidine residues but are clearly related.	158	1	115824	5696	7.669E-03	0.129	3.719E-02	1.430
pfam01268	FTHFS,Formate--tetrahydrofolate ligase	FTHFS.Formate--tetrahydrofolate ligase.	158	1	115824	5696	7.669E-03	0.129	3.745E-02	1.427
pfam08648	DUF1777,Protein of unknown function (DUF1777)	DUF1777.Protein of unknown function (DUF1777). This is a family of eukaryotic proteins of unknown function. Some of the proteins in this family are putative nucleic acid binding proteins.	163	1	115824	5696	8.044E-03	0.125	3.874E-02	1.412

Table S2

cl02554	PWWP.	PWWP. The PWWP domain is named after a conserved Pro-Trp-Trp-Pro motif. The domain binds to Histone-4 methylated at lysine-20, H4K20me, suggesting that it is methyl-lysine recognition motif. Removal of two conserved aromatic residues in a hydrophobic cavity created by this domain within the full-lemgth protein, Pdp1, abolishes the interaction o f the protein with H4K20me3. In fission yeast, Set9 is the sole enzyme that catalyses all three states of H4K20me, and Set9-mediated H4K20me is required for efficient recruitment of checkpoint protein Crb2 to sites of DNA damage. The methylation of H4K20 is involved in a diverse array of cellular processes, such as organising higher-order chromatin, maintaining genome stability, and regulating cell-cycle progression	329	6	115824	5696	8.893E-03	0.371	4.254E-02	1.371
pfam01370	Epimerase,NAD dependent epimerase/dehydratase family	Epimerase.NAD dependent epimerase/dehydratase family. This family of proteins utilize NAD as a cofactor. The proteins in this family use nucleotide-sugar substrates for a variety of chemical reactions.	190	2	115824	5696	9.663E-03	0.214	4.442E-02	1.352
pfam02758	PAAD_DAPIN,PAAD/DAPIN/Pyrin domain	PAAD_DAPIN.PAAD/DAPIN/Pyrin domain. This domain is predicted to contain 6 alpha helices and to have the same fold as the pfam00531 domain. This similarity may mean that this is a protein-protein interaction domain.	190	2	115824	5696	9.663E-03	0.214	4.471E-02	1.350
