# Supplementary material for: Bioinformatic Analysis and Post-Translational Modification Crosstalk Prediction of Lysine Acetylation
Source: PLoS One. 2011 Dec 2;6(12):e28228. doi: 10.1371/journal.pone.0028228 (PMC3229533; doi:10.1371/journal.pone.0028228)
Supplement: Table S3 — Phosphorylation sites affected. NetPhosK 1.0 was employed to predict kinase-specific phosphorylation for nearby sites, using the default program settings. The sites where the phosphorylation binding site or the kinase binding were affected by the change from K to Q or L were tallied. (PDF) [file pone.0028228.s006.pdf]

Table S3

IPI	KacPos	PhosphPos	Kac->L	Kac->Q	KnownPhosph	GeneID	Symbol	Seq
IPI00000057	3	4	PKA->CKI+PKA			22796	COG2	-------mekSrmnlpkgpdt
IPI00000104	395	390	unsp+GSK3->unsp+GSK3+p38MAPK	unsp+GSK3->unsp+GSK3+p38MAPK		8732	RNGTT	rlqciereiiSprhekmktgl
IPI00000104	397	390	unsp+GSK3->unsp+GSK3+p38MAPK	unsp+GSK3->unsp+GSK3+p38MAPK		8732	RNGTT	rlqciereiiSprhekmktgl
IPI00000352	57	55	PKC->	PKC->		9149	DYRK1B	lrklsvdlikTykhinevyya
IPI00000425	279	275	PKC+cdc2->			23545	ATP6V0A2	glntriqdlyTvlhktedylr
IPI00000425	279	280	->CKII	->CKII		23545	ATP6V0A2	iqdlytvlhkTedylrqvlck
IPI00000425	279	283	unsp->	unsp->		23545	ATP6V0A2	lytvlhktedYlrqvlckaae
IPI00000494	48	46	unsp->	unsp->		6125	RPL5	lviqdknkynTpkyrmivrvt
IPI00000494	48	49	unsp->	unsp->	PhosphoSite	6125	RPL5	qdknkyntpkYrmivrvtnrd
IPI00000494	221	219	INSR+SRC+unsp+EGFR->INSR+SRC+unsp			6125	RPL5	rylmeededaYkkqfsqyikn
IPI00000494	221	224	DNAPK->ATM+DNAPK			6125	RPL5	ededaykkqfSqyiknsvtpd
IPI00000643	111	109	unsp+PKC+cdc2->cdc2	unsp+PKC+cdc2->cdc2		9532	BAG2	etirnpqqqeSlkhatriide
IPI00000684	180	179		PKC->		6675	UAP1	rtmestkeffTkhkyfglkke
IPI00000690	295	292	unsp->	unsp->		9131	AIFM1	tlfrkigdfrSlekisrevks
IPI00000690	295	297	unsp->			9131	AIFM1	igdfrslekiSrevksitiig
IPI00000712	438	439	unsp+cdk5->unsp+cdk5+p38MAPK			4780	NFE2L2	elpvspghrkTpftkdkhssr
IPI00000712	443	442		PKC->PKC+DNAPK		4780	NFE2L2	vspghrktpfTkdkhssrlea
IPI00000712	443	447		unsp+CKII->unsp		4780	NFE2L2	rktpftkdkhSsrleahltrd
IPI00000712	445	439		unsp+cdk5->unsp+GSK3+cdk5		4780	NFE2L2	elpvspghrkTpftkdkhssr
IPI00000712	487	486	unsp+CKII->CKII	unsp+CKII->ATM+unsp+CKII+DNAPK		4780	NFE2L2	lpvvdfnemmSkeqfneaqla
IPI00000712	554	549	->PKA			4780	NFE2L2	llkekgendkSlhllkkqlst
IPI00000712	554	558		PKA->	PhosphoSite	4780	NFE2L2	kslhllkkqlStlylevfsml
IPI00000712	554	559	PKC+cdc2+PKA->cdc2	PKC+cdc2+PKA->cdc2+PKA	PhosphoSite	4780	NFE2L2	slhllkkqlsTlylevfsmlr
IPI00000712	555	558	PKA->	PKA->	PhosphoSite	4780	NFE2L2	kslhllkkqlStlylevfsml
IPI00000712	555	559	PKC+cdc2+PKA->cdc2+PKA	PKC+cdc2+PKA->PKC+cdc2	PhosphoSite	4780	NFE2L2	slhllkkqlsTlylevfsmlr
IPI00000784	116	117	unsp->SRC+unsp		PhosphoSite	4209	MEF2D	leqsplledkYrraseeldgl
IPI00000787	53	55	->cdk5+p38MAPK	->cdk5		5698	PSMB9	avvnrvfdklSplheriycal
IPI00000787	109	107	unsp+PKC+PKA->PKA	unsp+PKC+PKA->PKA		5698	PSMB9	laaanvvrniSykyredlsah
IPI00000792	23	26	PKA->	PKA->		1429	CRYZ	fggpevlklrSdiavpipkdh
IPI00000816	50	46	PKC->PKA			7531	YWHAE	eltveernllSvayknvigar
IPI00000816	69	65	PKC+CKII->CKII	PKC+CKII->CKII		7531	YWHAE	arraswriisSieqkeenkgg
IPI00000816	118	114		unsp->		7531	YWHAE	ldkhlipaanTgeskvfyykm
IPI00000816	118	117		->DNAPK		7531	YWHAE	hlipaantgeSkvfyykmkgd
IPI00000816	118	121	unsp->	unsp->		7531	YWHAE	aantgeskvfYykmkgdyhry
IPI00000816	123	121	unsp->			7531	YWHAE	aantgeskvfYykmkgdyhry
IPI00000874	7	2	CKI->	CKI->		5052	PRDX1	---------mSsgnakighpa
IPI00000874	7	3	PKC->	PKC->		5052	PRDX1	--------msSgnakighpap
IPI00000874	16	18	PKG->	PKG->		5052	PRDX1	ighpapnfkaTavmpdgqfkd
IPI00000874	27	32	unsp+PKC+CKII->unsp+cdc2	unsp+PKC+CKII->unsp+cdc2+CKII		5052	PRDX1	pdgqfkdislSdykgkyvvff
IPI00000874	35	32	unsp+PKC+CKII->unsp+CKII	unsp+PKC+CKII->unsp+CKII		5052	PRDX1	pdgqfkdislSdykgkyvvff
IPI00000874	35	34	unsp->			5052	PRDX1	gqfkdislsdYkgkyvvfffy
IPI00000874	35	38	unsp->			5052	PRDX1	dislsdykgkYvvfffypldf
IPI00000874	37	32	unsp+PKC+CKII->unsp	unsp+PKC+CKII->unsp+CKII		5052	PRDX1	pdgqfkdislSdykgkyvvff
IPI00000874	37	34		unsp->		5052	PRDX1	gqfkdislsdYkgkyvvfffy
IPI00000874	197	196		->ATM+DNAPK		5052	PRDX1	pdvqkskeyfSkqk-------
IPI00000948	314	312	SRC+unsp->SRC			26608	TBL2	twklwdtdveYkkkqdpyllk
IPI00000948	315	312		SRC+unsp->EGFR		26608	TBL2	twklwdtdveYkkkqdpyllk
IPI00000948	315	319	unsp->	unsp->		26608	TBL2	dveykkkqdpYllktgrfeea
IPI00001091	543	539	PKC->	PKC->unsp		10939	AFG3L2	aliaarhlsdSinqkhfeqai
IPI00001146	59	60	INSR->			11157	LSM6	eyvngqlknkYgdafirgnnv
IPI00001364	278	281	unsp+PKC+DNAPK->ATM+unsp+DNAPK			94104	C21orf66	rrivfsvkekSqrqkiaeeig
IPI00001364	280	281	unsp+PKC+DNAPK->ATM+unsp+PKC+DNAPK			94104	C21orf66	rrivfsvkekSqrqkiaeeig
IPI00001432	299	297	unsp+PKC+PKA->unsp+cdc2+PKA	unsp+PKC+PKA->unsp+PKA		56133	PCDHB2	sedirktfrlSaksgelllrq
IPI00001453	290	289		->ATM		9118	INA	nlqsaeewykSkfanlneqaa
IPI00001539	25	30	CKII->			10449	ACAA2	yggllkdftaTdlsefaakaa
IPI00001539	25	33	unsp->unsp+cdc2			10449	ACAA2	llkdftatdlSefaakaalsa
IPI00001539	81	83	unsp+cdk5+p38MAPK->unsp+GSK3+cdk5+p38MAPK	unsp+cdk5+p38MAPK->unsp+GSK3+cdk5+p38MAPK		10449	ACAA2	vglrvgipkeTpaltinrlcg
IPI00001539	137	136	unsp->unsp+PKA			10449	ACAA2	pycvrnvrfgTklgsdikled
IPI00001539	137	140	unsp+cdc2+CKII->cdc2+CKII	unsp+cdc2+CKII->cdc2+CKII		10449	ACAA2	rnvrfgtklgSdikledslwv
IPI00001539	270	274	p38MAPK->			10449	ACAA2	sedavkkhnfTplarivgyfv
IPI00001600	203	206	PKC->		PhosphoSite	79447	C16orf53	krearldkvlSdmkrhkklee
IPI00001600	209	206	PKC->	PKC->	PhosphoSite	79447	C16orf53	krearldkvlSdmkrhkklee
IPI00001639	211	213	->PKC			3837	KPNB1	eftkanfdkeSerhfimqvvc
IPI00001639	867	863	PKC->			3837	KPNB1	sktnkaktlaTwatkelrklk
IPI00001639	867	866	->DNAPK	->DNAPK		3837	KPNB1	nkaktlatwaTkelrklknqa
IPI00001735	1218	1215	cdc2->PKA+cdc2	cdc2->PKA+cdc2		9612	NCOR2	gtalgsvpggSitkgipstrv
IPI00001735	1218	1217	cdc2->CKI+cdc2	cdc2->ATM+cdc2		9612	NCOR2	algsvpggsiTkgipstrvps
IPI00001735	1795	1786	unsp+CKI+GSK3+cdk5+p38MAPK->unsp+CKI+GSK3+cdk5+cdc2+p38MAPK		Uniprot	9612	NCOR2	fssrhsssplSpggpthltkp
IPI00001735	1795	1794	PKC+cdc2->cdc2	PKC+cdc2->cdc2		9612	NCOR2	plspggpthlTkptttssser
IPI00001735	1795	1797		PKC->		9612	NCOR2	pggpthltkpTttsssererd
IPI00001735	1970	1975	unsp+PKG+cdc2->unsp+cdc2		PhosphoSite	9612	NCOR2	haflakpparSglepasspsk
IPI00001735	2037	2034	unsp+PKG+RSK+DNAPK->unsp+PKG+DNAPK	unsp+PKG+RSK+DNAPK->unsp+PKG+DNAPK		9612	NCOR2	asasdphrekTqskpfsiqel
IPI00001735	2037	2036	unsp+PKC+PKB->DNAPK	unsp+PKC+PKB->ATM+unsp+PKB+DNAPK		9612	NCOR2	asdphrektqSkpfsiqelel
IPI00001960	130	128	EGFR->	EGFR->unsp+EGFR		25932	CLIC4	gmdifakfsaYiknsrpeane
IPI00001960	130	132	PKG+CKII->CKII	PKG+CKII->CKII		25932	CLIC4	fakfsayiknSrpeanealer
IPI00002144	321	320	CKII->	CKII->CKII+DNAPK		57574	4-Mar	qqwkvlnydkTkdledqkagg
IPI00002255	2117	2125	->INSR	->INSR		987	LRBA	fkkidpkilaYteglhgkwlf
IPI00002270	40	41		unsp+RSK->RSK		79624	C6orf211	kvidtlhrhkSeffekhgeeg
IPI00002270	73	71	unsp+PKC->CKII	unsp+PKC->		79624	C6orf211	llsklrnelqTdkpfiplvek
IPI00002324	299	298		unsp->ATM+unsp+DNAPK		27430	MAT2B	qrprnaqldcSkletlgigqr
IPI00002349	140	138	unsp+PKC->PKC	unsp+PKC->PKC		57532	NUFIP2	qqvvdtslkqTvkantfgkag
IPI00002372	260	261	unsp->SRC+unsp	unsp->SRC+unsp		5825	ABCD3	igkmtiteqkYegeyryvnsr
IPI00002424	44	43	PKC->	PKC->		79666	PLEKHF2	grvligegvlTklcrkkpkar
IPI00002460	233	229		unsp->		310	ANXA7	qrqkikaafkTsygkdlikdl
IPI00002460	233	230	PKC->	PKC->		310	ANXA7	rqkikaafktSygkdlikdlk
IPI00002460	233	231	INSR+unsp->INSR			310	ANXA7	qkikaafktsYgkdlikdlks
IPI00002519	271	275	unsp+PKC->PKC			6470	SHMT1	gmifyrkgvkSvdpktgkeil
IPI00002520	103	100		unsp->INSR		6472	SHMT2	sclnnkysegYpgkryyggae
IPI00002520	181	178	->CKII			6472	SHMT2	dgghlthgymSdvkrisatsi
IPI00002520	181	184	unsp+PKA->unsp+PKG+PKA			6472	SHMT2	hgymsdvkriSatsiffesmp
IPI00002520	269	266	unsp+cdk5+p38MAPK->p38MAPK	unsp+cdk5+p38MAPK->unsp+p38MAPK		6472	SHMT2	sglvaakvipSpfkhadivtt
IPI00002520	464	460		->unsp		6472	SHMT2	egvniglevkSktaklqdfks
IPI00002520	474	478		->cdc2		6472	SHMT2	fksfllkdseTsqrlanlrqr
IPI00002520	474	479	unsp+DNAPK->DNAPK	unsp+DNAPK->DNAPK		6472	SHMT2	ksfllkdsetSqrlanlrqrv
IPI00002521	94	92	PKC->	PKC->		522	ATP5J	fgnadmntfpTfkfedpkfev
IPI00002525	136	133		->INSR		29937	NENF	ealdevftkvYkakypivgyt
IPI00002564	256	257	unsp+GSK3+cdk5+p38MAPK->unsp+GSK3+p38MAPK	unsp+GSK3+cdk5+p38MAPK->unsp+cdk5+p38MAPK	Uniprot|PhosphoSite	7515	XRCC1	kldlnqeekkTpskppaqlsp
IPI00002564	256	259	PKC+cdc2+PKA->PKC+cdc2	PKC+cdc2+PKA->PKC+cdc2	Uniprot|PhosphoSite	7515	XRCC1	dlnqeekktpSkppaqlspsv
IPI00002564	260	257	unsp+GSK3+cdk5+p38MAPK->unsp+GSK3+p38MAPK	unsp+GSK3+cdk5+p38MAPK->unsp+GSK3+p38MAPK	Uniprot|PhosphoSite	7515	XRCC1	kldlnqeekkTpskppaqlsp
IPI00002564	260	259	PKC+cdc2+PKA->cdc2+PKA+DNAPK	PKC+cdc2+PKA->cdc2+DNAPK	Uniprot|PhosphoSite	7515	XRCC1	dlnqeekktpSkppaqlspsv
IPI00002564	260	266	unsp+GSK3+cdk5+cdc2+p38MAPK->unsp+cdk5+cdc2+p38	unsp+GSK3+cdk5+cdc2+p38MAPK->unsp+cdk5+cdc2	Uniprot|PhosphoSite	7515	XRCC1	ktpskppaqlSpsvpkrpklp
IPI00002564	271	266	unsp+GSK3+cdk5+cdc2+p38MAPK->unsp+cdk5+cdc2+p38	unsp+GSK3+cdk5+cdc2+p38MAPK->unsp+cdk5+cdc2	Uniprot|PhosphoSite	7515	XRCC1	ktpskppaqlSpsvpkrpklp
IPI00002852	84	79	PKC->			5828	PEX2	iysknatvgqSvlnikykndf
IPI00002852	84	90	cdk5->	cdk5->		5828	PEX2	vlnikykndfSpnlryqppsk
IPI00002852	86	90		cdk5->cdk5+p38MAPK		5828	PEX2	vlnikykndfSpnlryqppsk
IPI00002922	254	253	unsp+cdc2->unsp+PKA+cdc2	unsp+cdc2->unsp+cdc2+DNAPK		79718	TBL1XR1	gsydgfariwTkdgnlastlg
IPI00002966	272	267	ATM+unsp+DNAPK->ATM+unsp+PKA+DNAPK			3308	HSPA4	kskirallrlSqeceklkklm
IPI00002966	430	421	->cdc2			3308	HSPA4	eegssdcevfSknhaapfskv
IPI00002966	430	429		->ATM+DNAPK		3308	HSPA4	vfsknhaapfSkvltfyrkep
IPI00002993	5	3	PKC->	PKC->		6880	TAF9	--------meSgktaspksmp
IPI00002993	5	6	->PKC	->unsp+PKC		6880	TAF9	-----mesgkTaspksmpkda
IPI00003168	188	189		unsp+GSK3+cdk5+p38MAPK->unsp+cdk5+p38MAPK		5636	PRPSAP2	dyrnavivakSpasakraqsf
IPI00003168	194	198	unsp+PKC+PKG+PKA+RSK->unsp+PKG	unsp+PKC+PKG+PKA+RSK->unsp+PKC+PKG+RSK		5636	PRPSAP2	kspasakraqSfaerlrlgia
IPI00003362	586	588	unsp+CKII->unsp+CKI+CKII	unsp+CKII->unsp+CKI+CKII		3309	HSPA5	gdkeklggklSsedketmeka
IPI00003362	586	589	PKC+CKII->CKII			3309	HSPA5	dkeklggklsSedketmekav
IPI00003377	24	20	PKC->	PKC->		6432	SFRS7	etkvyvgnlgTgagkgelera
IPI00003377	24	32	unsp->unsp+cdc2			6432	SFRS7	agkgelerafSyygplrtvwi
IPI00003377	185	183	unsp+PKC+PKB+RSK->unsp+PKB+RSK	unsp+PKC+PKB+RSK->unsp+PKB+RSK	niprot|PHOsphoELM|PhosphoSi	6432	SFRS7	saslrrsrsgSikgsryfqsp
IPI00003377	185	187	unsp+PKC+cdc2->PKC+cdc2	unsp+PKC+cdc2->unsp+cdc2	Uniprot	6432	SFRS7	rrsrsgsikgSryfqspsrsr
IPI00003377	185	189	unsp->	unsp->		6432	SFRS7	srsgsikgsrYfqspsrsrsr
IPI00003419	174	171	unsp+PKC->PKA	unsp+PKC->unsp		10944	C11orf58	edpknkkdakSnykmmfvkss
IPI00003419	174	173	unsp->	unsp->		10944	C11orf58	pknkkdaksnYkmmfvkssgs
IPI00003419	179	180	->PKC	->PKC		10944	C11orf58	ksnykmmfvkSsgs-------
IPI00003419	179	181	PKA->PKC	PKA->		10944	C11orf58	snykmmfvksSgs--------
IPI00003442	389	384	unsp+CKII->CKII	unsp+CKII->CKII		3091	HIF1A	lftkvesedtSslfdklkkep
IPI00003442	532	525	CKII->cdc2+CKII			3091	HIF1A	spseycfyvdSdmvnefklel
IPI00003443	208	211	unsp+PKC->unsp			3428	IFI16	qkrpvivkvlSttkpfeyetp

IPI00003443	214	211	unsp+PKC->unsp	unsp+PKC->unsp		3428	IFI16	qkrpvivkvlSttkpfeyetp
IPI00003443	214	212	unsp+PKC->PKC	unsp+PKC->PKC		3428	IFI16	krpvivkvlsTtkpfeyetpe
IPI00003443	214	213		->DNAPK		3428	IFI16	rpvivkvlstTkpfeyetpem
IPI00003443	214	218	INSR+unsp->INSR	INSR+unsp->INSR+unsp+EGFR		3428	IFI16	kvlsttkpfeYetpemekkim
IPI00003482	110	105	ATM+PKC+DNAPK->ATM+DNAPK	ATM+PKC+DNAPK->ATM+DNAPK		1666	DECR1	vlkataeqisSqtgnkvhaiq
IPI00003482	110	107	PKC->			1666	DECR1	kataeqissqTgnkvhaiqcd
IPI00003494	48	39	unsp->unsp+cdc2			9232	PTTG1	gpsikaldgrSqvstprfgkt
IPI00003494	48	43	unsp+PKC+cdk5+p38MAPK->unsp+cdk5+p38MAPK	unsp+PKC+cdk5+p38MAPK->unsp+cdk5+p38MAPK		9232	PTTG1	kaldgrsqvsTprfgktfdap
IPI00003494	48	49		PKC->unsp+PKC		9232	PTTG1	sqvstprfgkTfdappalpka
IPI00003495	601	599	unsp->	unsp->		9258	MFHAS1	asphaayygvSdknlrrrkah
IPI00003588	12	13	->CKI			9521	EEF1E1	aaaelsllekSlglskgnkys
IPI00003588	12	17	unsp+PKC->	unsp+PKC->		9521	EEF1E1	lsllekslglSkgnkysaqge
IPI00003588	138	139	unsp+EGFR->unsp			9521	EEF1E1	vdltvqekekYlnvsrwfchi
IPI00003588	166	161	unsp+PKC+RSK->unsp+PKC	unsp+PKC+RSK->PKC		9521	EEF1E1	hypgirqhlsSvvfiknrlyt
IPI00003588	166	170	unsp->			9521	EEF1E1	ssvvfiknrlYtnsh------
IPI00003588	166	171	PKA+RSK->RSK	PKA+RSK->		9521	EEF1E1	svvfiknrlyTnsh-------
IPI00003766	172	173		->unsp		23474	ETHE1	rtdfqqgcakTlyhsvhekif
IPI00003766	172	175	unsp->			23474	ETHE1	dfqqgcaktlYhsvhekiftl
IPI00003766	172	177	unsp+CKII->unsp+cdc2	unsp+CKII->unsp+cdc2+CKII		23474	ETHE1	qqgcaktlyhSvhekiftlpg
IPI00003814	82	87	PKC+PKA->PKC	PKC+PKA->PKC		5608	MAP2K6	qimavkriraTvnsqeqkrll
IPI00003815	127	128	unsp->	unsp->		396	ARHGDIA	vnreivsgmkYiqhtyrkgvk
IPI00003815	141	144	INSR+unsp->INSR		PhosphoSite	396	ARHGDIA	rkgvkidktdYmvgsygprae
IPI00003815	178	174	unsp+PKC+DNAPK->unsp+PKA+DNAPK	unsp+PKC+DNAPK->unsp+PKC+PKA+DNAPK	PHOsphoELM|PhosphoSite	396	ARHGDIA	eapkgmlargSysiksrftdd
IPI00003815	178	176	unsp+PKC->	unsp+PKC->		396	ARHGDIA	pkgmlargsySiksrftdddk
IPI00003815	178	179	unsp->	unsp->		396	ARHGDIA	mlargsysikSrftdddktdh
IPI00003815	178	182	unsp+PKC+CKII->unsp+CKII			396	ARHGDIA	rgsysiksrfTdddktdhlsw
IPI00003817	21	20	unsp+CKII->	unsp+CKII->ATM+unsp+CKII		397	ARHGDIB	veeddddeldSklnykpppqk
IPI00003817	21	24	unsp->		Uniprot|PHOsphoELM|PhosphoSi	397	ARHGDIB	dddeldsklnYkpppqkslke
IPI00003817	25	20	unsp+CKII->	unsp+CKII->CKII		397	ARHGDIB	veeddddeldSklnykpppqk
IPI00003817	30	31	unsp+PKG->unsp	unsp+PKG->unsp		397	ARHGDIB	klnykpppqkSlkelqemdkd
IPI00003817	33	31	unsp+PKG->	unsp+PKG->		397	ARHGDIB	klnykpppqkSlkelqemdkd
IPI00003817	40	44		unsp+CKI+PKC->unsp+CKI+PKC+cdc2		397	ARHGDIB	elqemdkddeSlikykktllg
IPI00003817	40	48	->INSR	->INSR		397	ARHGDIB	mdkddeslikYkktllgdgpv
IPI00003817	47	44	unsp+CKI+PKC->unsp+CKI			397	ARHGDIB	elqemdkddeSlikykktllg
IPI00003817	102	98	->PKA	->PKA		397	ARHGDIB	tgdlealkkeTivlkegseyr
IPI00003817	124	121	PKC->			397	ARHGDIB	ihfkvnrdivSglkyvqhtyr
IPI00003817	175	171	PKC->PKC+PKA			397	ARHGDIB	eapkgmlargTyhnksfftdd
IPI00003817	175	172	unsp->	unsp->EGFR		397	ARHGDIB	apkgmlargtYhnksfftddd
IPI00003817	175	176	unsp->	unsp->		397	ARHGDIB	mlargtyhnkSfftdddkqdh
IPI00003833	158	157	unsp+PKA->unsp+PKA+DNAPK	unsp+PKA->ATM+unsp+PKA+DNAPK		23788	MTCH2	rsmvqfigreSkycglcdsii
IPI00003865	71	66	unsp+PKC->unsp		PhosphoSite	3312	HSPA8	knqvamnptnTvfdakrligr
IPI00003865	108	111	PKC->			3312	HSPA8	pkvqveykgeTksfypeevss
IPI00003865	108	113	unsp+cdc2->cdc2	unsp+cdc2->cdc2	Uniprot	3312	HSPA8	vqveykgetkSfypeevssmv
IPI00003865	112	111	PKC->CKI	PKC->DNAPK		3312	HSPA8	pkvqveykgeTksfypeevss
IPI00003865	112	113		unsp+cdc2->CKII+cdc2	Uniprot	3312	HSPA8	vqveykgetkSfypeevssmv
IPI00003865	246	254	->cdc2			3312	HSPA8	efkrkhkkdiSenkravrrlr
IPI00003865	319	313	unsp->unsp+PKA	unsp->unsp+PKA	PhosphoSite	3312	HSPA8	eelnadlfrgTldpvekalrd
IPI00003865	512	511	unsp+CKII->unsp+PKA	unsp+CKII->ATM+unsp+CKII+DNAPK		3312	HSPA8	ititndkgrlSkediermvqe
IPI00003865	531	537	unsp+PKC+PKG->unsp+PKC+PKG+PKA			3312	HSPA8	aedekqrdkvSsknslesyaf
IPI00003865	589	586	->CKII	->CKII		3312	HSPA8	eiinwldknqTaekeefehqq
IPI00003870	211	213		PKC->		8192	CLPP	kqlyniyakhTkqslqviesa
IPI00003870	211	216	PKC+PKA->PKC			8192	CLPP	yniyakhtkqSlqviesamer
IPI00003881	87	88		unsp->		3185	HNRNPF	mghryievfkShrtemdwvlk
IPI00003918	14	9	unsp+PKC->PKA	unsp+PKC->PKC		6124	RPL4	--macarpliSvysekgessg
IPI00003918	14	12	unsp->	unsp->		6124	RPL4	acarplisvySekgessgknv
IPI00003918	14	18	unsp+PKC+PKA->unsp	unsp+PKC+PKA->unsp+PKC		6124	RPL4	isvysekgesSgknvtlpavf
IPI00003918	106	105	PKC->	PKC->DNAPK		6124	RPL4	mcrggrmfapTktwrrwhrrv
IPI00003918	163	164		PKC->unsp+PKC		6124	RPL4	vedkvegykkTkeavlllkkl
IPI00003925	354	356	PKA->	PKA->		5162	PDHB	vkdiifaikkTlni-------
IPI00003935	6	7		unsp->		8349	HIST2H2BE	----mpepakSapapkkgskk
IPI00003935	12	7	unsp->	unsp->		8349	HIST2H2BE	----mpepakSapapkkgskk
IPI00003935	12	15	unsp+PKC->PKC	unsp+PKC->PKC	Uniprot	8349	HIST2H2BE	aksapapkkgSkkavtkaqkk
IPI00003935	13	15	unsp+PKC->PKC		Uniprot	8349	HIST2H2BE	aksapapkkgSkkavtkaqkk
IPI00003935	16	15	unsp+PKC->unsp+PKC+PKA	unsp+PKC->ATM+unsp+PKC+DNAPK	Uniprot	8349	HIST2H2BE	aksapapkkgSkkavtkaqkk
IPI00003935	17	15	unsp+PKC->PKC+PKA	unsp+PKC->PKC+PKA	Uniprot	8349	HIST2H2BE	aksapapkkgSkkavtkaqkk
IPI00003935	21	15	unsp+PKC->unsp+PKC+PKA	unsp+PKC->unsp+PKC+PKA	Uniprot	8349	HIST2H2BE	aksapapkkgSkkavtkaqkk
IPI00003935	21	20		PKC->PKC+DNAPK		8349	HIST2H2BE	apkkgskkavTkaqkkdgkkr
IPI00003935	109	113	unsp->			8349	HIST2H2BE	lpgelakhavSegtkavtkyt
IPI00003935	121	120		->ATM+DNAPK		8349	HIST2H2BE	havsegtkavTkytssk----
IPI00003935	121	123		PKC->		8349	HIST2H2BE	segtkavtkyTssk-------
IPI00003949	10	12	unsp->PKC			7334	UBE2N	aglprriikeTqrllaepvpg
IPI00003949	94	96	->p38MAPK			7334	UBE2N	icldilkdkwSpalqirtvll
IPI00003965	595	592	PKC->PKA	PKC->PKC+PKA		7874	USP7	dmydeekvkyTvfkvlknssl
IPI00003965	595	600	PKA->cdc2	PKA->cdc2		7874	USP7	kytvfkvlknSslaefvqsls
IPI00003965	1096	1091	EGFR->	EGFR->		7874	USP7	hfnkapkrsrYtylekaikih
IPI00003965	1096	1092	unsp+PKC->unsp+PKA	unsp+PKC->unsp		7874	USP7	fnkapkrsryTylekaikihn
IPI00004233	993	988	PKC+cdc2->cdc2	PKC+cdc2->cdc2		4288	MKI67	dtargqnllqTqdhakapkse
IPI00004233	1165	1169	unsp+PKC->			4288	MKI67	eflalrkltpSagkamltpkp
IPI00004233	1639	1635	unsp+PKC->unsp+RSK	unsp+PKC->PKG		4288	MKI67	npasskrrlkTslgkvgvkee
IPI00004233	1639	1636	PKC->PKA	PKC->PKC+PKA	Uniprot	4288	MKI67	passkrrlktSlgkvgvkeel
IPI00004233	1938	1937		unsp+PKC+cdc2->ATM+unsp+PKC+cdc2+DNAPK	Uniprot|PhosphoSite	4288	MKI67	kldllgnlpgSkrrpqtpkek
IPI00004233	2264	2259	unsp+PKG->unsp+PKG+PKA			4288	MKI67	eeeslalrkrTpsvgkamdtp
IPI00004233	2421	2420		unsp+PKC->ATM+unsp+PKC+DNAPK	Uniprot|PhosphoSite	4288	MKI67	kldllgnlpgSkrqpqtpkek
IPI00004344	79	81	PKG->PKC	PKG->		27125	AFF4	ipklvaipkpTvppsadeksn
IPI00004344	79	85		unsp+CKII->unsp		27125	AFF4	vaipkptvppSadeksnpnff
IPI00004350	12	4	->cdc2			2957	GTF2A1	-------manSantntvpkly
IPI00004350	12	7	PKC->			2957	GTF2A1	----mansanTntvpklyrsv
IPI00004350	12	16	unsp+PKG->unsp+PKG+CKII			2957	GTF2A1	ntntvpklyrSviedvindvr
IPI00004358	316	315	unsp+PKC->PKC+PKA	unsp+PKC->ATM+unsp		5834	PYGB	lqdiirrfksSkfgcrdpvrt
IPI00004358	811	809	unsp+PKC->PKC	unsp+PKC->PKC		5834	PYGB	tkkvirniacSgkfssdrtit
IPI00004358	811	813	unsp->CKI	unsp->unsp+CKI		5834	PYGB	irniacsgkfSsdrtiteyar
IPI00004363	351	356		->p38MAPK	niprot|PHOsphoELM|PhosphoSi	27347	STK39	eyliekllrtTpdiaqrakkv
IPI00004461	56	52	->PKA			1716	DGUOK	siegniavgkStfvklltkty
IPI00004461	56	53	PKC->	PKC->		1716	DGUOK	iegniavgksTfvklltktyp
IPI00004461	56	59	PKC+p38MAPK->PKC			1716	DGUOK	vgkstfvkllTktypewhvat
IPI00004461	275	272	PKC->	PKC->		1716	DGUOK	kqedlmrevnTfvknl-----
IPI00004845	45	41	->PKA			25934	NIPSNAP3A	qydgifyefrSyylkpskmne
IPI00004845	45	43	EGFR->			25934	NIPSNAP3A	dgifyefrsyYlkpskmnefl
IPI00004845	45	47	unsp+PKA->	unsp+PKA->unsp		25934	NIPSNAP3A	yefrsyylkpSkmneflenfe
IPI00004845	48	43		EGFR->		25934	NIPSNAP3A	dgifyefrsyYlkpskmnefl
IPI00004845	48	47	unsp+PKA->PKA	unsp+PKA->unsp		25934	NIPSNAP3A	yefrsyylkpSkmneflenfe
IPI00004859	755	754	unsp+PKC->unsp	unsp+PKC->ATM+unsp+DNAPK		641	BLM	seatniylqlSkkdpiiklly
IPI00004859	863	856	PKC->PKC+cdc2			641	BLM	kilrpqvfsmSfnrhnlkyyv
IPI00004859	1411	1413	PKG+PKA->	PKG+PKA->		641	BLM	kpinrpflkpSyafs------
IPI00004859	1411	1414	unsp->	unsp->		641	BLM	pinrpflkpsYafs-------
IPI00004860	60	61		unsp+PKA->PKA		5917	RARS	lkyrlnilrkSlqaernkptk
IPI00004860	205	200		CKI+p38MAPK->p38MAPK		5917	RARS	enkkvivdfsSpniakemhvg
IPI00004924	225	222	->EGFR	->EGFR		50619	DEF6	eliqdvlkqgYlwkrghlrrn
IPI00004968	122	121		unsp->unsp+DNAPK		27339	PRPF19	daacrviarlTkevtaareal
IPI00004968	261	264	unsp+PKC->PKC			27339	PRPF19	eqilatlkghTkkvtsvvfhp
IPI00005045	7	3	PKC->	PKC->		10061	ABCF2	--------mpSdlakkkaakk
IPI00005045	8	3	PKC->	PKC->		10061	ABCF2	--------mpSdlakkkaakk
IPI00005045	304	305	unsp->	unsp->		10061	ABCF2	iihmhnkklkYytgnydqyvk
IPI00005132	502	511	unsp+GSK3+PKA+RSK->unsp+GSK3+cdc2+PKA+RSK	unsp+GSK3+PKA+RSK->unsp+GSK3+cdc2+PKA+RSK		54552	GNL3L	akrnvdhrpkSnsmvdvcsvd
IPI00005154	33	26	unsp+PKA->unsp+cdc2+PKA			6749	SSRP1	gsmndgrlrlSrqgiifknsk
IPI00005154	33	35	unsp+PKC->PKC	unsp+PKC->		6749	SSRP1	lsrqgiifknSktgkvdniqa
IPI00005154	33	37	PKC->			6749	SSRP1	rqgiifknskTgkvdniqage
IPI00005154	90	86	CKII+cdc2->PKA+CKII+cdc2			6749	SSRP1	gfresefeklSdffkthyrle
IPI00005154	233	232		unsp->unsp+EGFR		6749	SSRP1	flhlhgktfdYkipyttvlrl
IPI00005154	233	236	INSR+unsp+EGFR->	INSR+unsp+EGFR->		6749	SSRP1	hgktfdykipYttvlrlfllp
IPI00005154	413	411	SRC+unsp+EGFR->SRC			6749	SSRP1	ytfssiereeYgklfdfvnak
IPI00005154	548	552	unsp+PKG+PKA+RSK->unsp+PKG+PKA			6749	SSRP1	kdpnapkrpmSaymlwlnasr
IPI00005159	299	295	PKC->	PKC->		10097	ACTR2	tiqaadidtrSefykhivlsg
IPI00005159	299	298	unsp->	unsp->		10097	ACTR2	aadidtrsefYkhivlsggst
IPI00005159	299	304	unsp+PKC+PKA->PKC	unsp+PKC+PKA->PKC		10097	ACTR2	rsefykhivlSggstmypglp
IPI00005159	322	315	CKII->cdc2+CKII			10097	ACTR2	ggstmypglpSrlerelkqly
IPI00005160	82	80	PKC->	PKC->		10095	ARPC1B	cgtdrnayvwTlkgrtwkptl
IPI00005161	275	271	PKC->PKA	PKC->PKA		10109	ARPC2	yihtrmraktSdflkvlnrar

IPI00005161	295	293	PKC->	PKC->		10109	ARPC2	daekkemktiTgktfssr---
IPI00005161	295	298	unsp+PKC->PKC	unsp+PKC->PKC		10109	ARPC2	emktitgktfSsr--------
IPI00005162	56	58		->unsp		10094	ARPC3	yfkanvffknYeikneadrtl
IPI00005347	260	262	PKG->PKC	PKG->		54764	ZRANB1	lkqiknrmkkTdwlflnacvg
IPI00005367	9	16	unsp+CKI+GSK3->unsp+CKI+GSK3+p38MAPK			51422	PRKAG2	mdtkkkkdvsSpggsggkkna
IPI00005367	10	8	unsp+PKC->cdc2	unsp+PKC->		51422	PRKAG2	---mgsavmdTkkkkdvsspg
IPI00005492	112	110	PKC->	PKC->		11091	WDR5	nllvsasddkTlkiwdvssgk
IPI00005502	642	633	unsp+GSK3+cdk5+p38MAPK->unsp+GSK3+cdk5+cdc2+p38MAPK			1822	ATN1	asspagyktaSppgpppygkr
IPI00005502	642	640	EGFR->			1822	ATN1	ktasppgpppYgkrapspgay
IPI00005502	642	646	unsp+GSK3+cdk5+PKA->unsp+GSK3+cdk5	unsp+GSK3+cdk5+PKA->unsp+GSK3+cdk5+cdc2		1822	ATN1	gpppygkrapSpgayktatpp
IPI00005578	327	323	unsp->unsp+PKA			30844	EHD4	iisylkkempSvfgkenkkre
IPI00005578	327	336	->cdc2	->cdc2		30844	EHD4	gkenkkreliSrlpeiyiqlq
IPI00005614	1913	1918	unsp+PKA->	unsp+PKA->	Uniprot	6711	SPTBN1	vdtgdkfrffSmvrdlmlwme
IPI00005630	117	121	unsp+GSK3+cdk5->unsp+cdk5			1869	E2F1	rgrhpgkgvkSpgeksryets
IPI00005630	120	121	unsp+GSK3+cdk5->unsp+CKII+p38MAPK	unsp+GSK3+cdk5->unsp+cdk5+CKII		1869	E2F1	rgrhpgkgvkSpgeksryets
IPI00005630	120	126		unsp+CKII->unsp		1869	E2F1	gkgvkspgekSryetslnltt
IPI00005630	125	121		unsp+GSK3+cdk5->unsp+cdk5		1869	E2F1	rgrhpgkgvkSpgeksryets
IPI00005630	125	128	INSR+unsp->	INSR+unsp->unsp		1869	E2F1	gvkspgeksrYetslnlttkr
IPI00005630	125	131	->PKA+cdc2	->cdc2		1869	E2F1	spgeksryetSlnlttkrfle
IPI00005648	424	415	PKA->cdc2+PKA			9667	SAFB2	sgrnlwvsglSsttratdlkn
IPI00005648	424	417	unsp+PKC->unsp+PKC+cdc2			9667	SAFB2	rnlwvsglssTtratdlknlf
IPI00005648	424	421	PKC+cdc2->	PKC+cdc2->		9667	SAFB2	vsglssttraTdlknlfskyg
IPI00005648	424	428	PKC->			9667	SAFB2	tratdlknlfSkygkvvgakv
IPI00005648	429	421		PKC+cdc2->PKC		9667	SAFB2	vsglssttraTdlknlfskyg
IPI00005648	429	428	PKC->	PKC->DNAPK		9667	SAFB2	tratdlknlfSkygkvvgakv
IPI00005648	616	613	PKC->PKC+CKII	PKC->CKI+PKC+CKII	Uniprot|PhosphoSite	9667	SAFB2	seskekrdilSfdkikeqrer
IPI00005648	829	825		->EGFR		9667	SAFB2	grdsrdgwggYgsdkrlsegr
IPI00005648	829	827	PKC->	PKC->		9667	SAFB2	dsrdgwggygSdkrlsegrgl
IPI00005648	829	832	unsp+PKA->unsp+cdc2	unsp+PKA->unsp+cdc2	PHOsphoELM|PhosphoSite	9667	SAFB2	wggygsdkrlSegrglpppr
IPI00005657	21	24	unsp+PKC->unsp+cdc2	unsp+PKC->unsp+PKC+cdc2		10471	PFDN6	ekyqqlqkdlSksmsgrqkle
IPI00005657	21	26	unsp+cdc2->cdc2	unsp+cdc2->cdc2		10471	PFDN6	yqqlqkdlskSmsgrqkleaq
IPI00005658	54	57		unsp+PKA->unsp+PKC+PKA		8266	UBL4A	gkaladgkrlSdysigpnskl
IPI00005658	54	59	INSR+unsp->unsp	INSR+unsp->unsp		8266	UBL4A	aladgkrlsdYsigpnsklnl
IPI00005668	4	3		->ATM+DNAPK		1646	AKR1C2	--------mdSkyqcvklndg
IPI00005668	33	32	->PKA	->ATM+DNAPK		1646	AKR1C2	gtyapaevpkSkaleavklai
IPI00005668	161	166	unsp+PKA->	unsp+PKA->		1646	AKR1C2	daglaksigvSnfnhrllemi
IPI00005668	246	251	PKG+PKA+p38MAPK->p38MAPK			1646	AKR1C2	lcalakkhkrTpalialryql
IPI00005677	128	125	PKC->	PKC->CKI		8443	GNPAT	rlgairfcafTlskvfkqifs
IPI00005677	128	127	PKC+cdc2->cdc2	PKC+cdc2->ATM+cdc2+DNAPK		8443	GNPAT	gairfcaftlSkvfkqifskv
IPI00005744	30	32	PKA->			6473	SHOX	gggggggkkdSityrevlesg
IPI00005744	30	34	unsp+PKC->unsp	unsp+PKC->unsp+PKC+cdc2		6473	SHOX	gggggkkdsiTyrevlesgla
IPI00005792	207	209	PKC+PKG->PKC	PKC+PKG->		8106	PABPN1	nrvtilcdkfSghpkgfayie
IPI00005966	47	44		SRC+unsp->SRC+unsp+EGFR		55967	NDUFA12	kvgtlvgedkYgnkyyednnk
IPI00005969	97	95	unsp+PKC+PKG->PKG	unsp+PKC+PKG->		829	CAPZA1	srfldprnkiSfkfdhlrkea
IPI00005969	273	267		PKC->PKC+cdc2		829	CAPZA1	alrrqlpvtrTkidwnkilsy
IPI00005969	273	276	unsp+PKC->PKC	unsp+PKC->PKC		829	CAPZA1	rtkidwnkilSykigkemqna
IPI00005978	36	44	EGFR->INSR+EGFR	EGFR->INSR+EGFR	PhosphoSite	6427	SFRS2	fekygrvgdvYiprdrytkes
IPI00006034	23	19	->PKA	->unsp		1397	CRIP2	dktvyfaekvSslgkdwhkfc
IPI00006034	23	20	PKC->	PKC->		1397	CRIP2	ktvyfaekvsSlgkdwhkfcl
IPI00006077	30	25	unsp+PKC+DNAPK->unsp+DNAPK	unsp+PKC+DNAPK->PKC+DNAPK		9767	PHF16	espstsftsgSmyrikskipn
IPI00006077	30	31	unsp+PKC+PKG->unsp+PKC	unsp+PKC+PKG->unsp+PKC		9767	PHF16	ftsgsmyrikSkipnehkkpa
IPI00006077	32	25	unsp+PKC+DNAPK->unsp+PKC+cdc2+DNAPK			9767	PHF16	espstsftsgSmyrikskipn
IPI00006077	32	31	unsp+PKC+PKG->unsp+PKC	unsp+PKC+PKG->ATM+unsp+DNAPK		9767	PHF16	ftsgsmyrikSkipnehkkpa
IPI00006077	638	635	PKC+PKA+cdc2->PKA+cdc2	PKC+PKA+cdc2->PKA+cdc2		9767	PHF16	tpssecyhgqSlgkplvlqaa
IPI00006077	735	733	EGFR->			9767	PHF16	wvkntedlqcYvkptknmspk
IPI00006077	735	737	PKC+PKG->PKC	PKC+PKG->		9767	PHF16	tedlqcyvkpTknmspkeqfw
IPI00006077	738	733	EGFR->	EGFR->		9767	PHF16	wvkntedlqcYvkptknmspk
IPI00006077	738	737	PKC+PKG->PKC	PKC+PKG->		9767	PHF16	tedlqcyvkpTknmspkeqfw
IPI00006077	738	741	unsp->unsp+PKG	unsp->unsp+PKG		9767	PHF16	qcyvkptknmSpkeqfwgrqv
IPI00006079	152	148	unsp+cdk5+cdc2+RSK->unsp+cdk5+RSK		Uniprot|PHOsphoELM|PhosphoSi	9774	BCLAF1	sprssssrssSpyskspvskr
IPI00006079	152	151		unsp->unsp+DNAPK	iprot|PHOsphoELM|PhosphoSi	9774	BCLAF1	ssssrssspySkspvskrrgs
IPI00006079	152	153	unsp+cdk5+p38MAPK->cdk5+cdc2+p38MAPK	unsp+cdk5+p38MAPK->cdk5+cdc2+p38MAPK	iprot|PHOsphoELM|PhosphoSi	9774	BCLAF1	ssrssspyskSpvskrrgsqe
IPI00006079	335	333	unsp+PKC->	unsp+PKC->		9774	BCLAF1	pdggdqetakTgkflkrftde
IPI00006079	437	431	unsp+CKI+CKII->unsp+CKII			9774	BCLAF1	ksfatashrnTeeeglkyksk
IPI00006079	437	438	INSR+unsp->			9774	BCLAF1	hrnteeeglkYkskvslkgnr
IPI00006079	437	440	unsp+cdc2->cdc2	unsp+cdc2->cdc2		9774	BCLAF1	nteeeglkykSkvslkgnres
IPI00006079	637	633	unsp->unsp+PKA			9774	BCLAF1	saamtlnerfTsyqkateehs
IPI00006079	637	634	unsp+PKA+RSK->PKA+RSK	unsp+PKA+RSK->unsp+PKA		9774	BCLAF1	aamtlnerftSyqkateehst
IPI00006079	637	635	EGFR->	EGFR->unsp+EGFR		9774	BCLAF1	amtlnerftsYqkateehstr
IPI00006091	1017	1012	unsp+PKC->unsp			1756	DMD	tvkemskkapSeisrkyqsef
IPI00006091	1017	1015	unsp+PKC->	unsp+PKC->		1756	DMD	emskkapseiSrkyqsefeei
IPI00006108	138	140		unsp+PKC+PKG->unsp+PKG		9794	MAML1	ygdlfpghkkTrreaplgvai
IPI00006160	321	319	unsp+cdc2->cdc2	unsp+cdc2->cdc2		7161	TP73	yreqqalnesSakngaaskra
IPI00006160	327	326		unsp+PKC+cdc2->ATM+unsp+PKC+cdc2+DNAPK		7161	TP73	nessakngaaSkrafkqsppa
IPI00006164	210	207	cdc2->PKA	cdc2->		80895	ILKAP	htdeeflkqaSsqkpawkdgs
IPI00006164	210	208	unsp+PKC+cdc2+DNAPK->cdc2+DNAPK	unsp+PKC+cdc2+DNAPK->cdc2+DNAPK		80895	ILKAP	tdeeflkqasSqkpawkdgst
IPI00006164	210	217	->PKA	->PKA		80895	ILKAP	ssqkpawkdgStatcvlavdn
IPI00006171	1262	1265	->cdc2	->cdc2		4798	NFRKB	tkliagnkpvSfltaqqlqql
IPI00006176	494	485		unsp+cdc2+CKII->unsp+CKII		9146	HGS	aqirdargalSalreehrekl
IPI00006181	53	49	PKC->	PKC->		8664	EIF3D	lgkvadwtgaTyqdkrytnky
IPI00006181	53	55	INSR+unsp->unsp	INSR+unsp->unsp+EGFR		8664	EIF3D	wtgatyqdkrYtnkyssqfgg
IPI00006181	53	56	unsp+PKC->PKC	unsp+PKC->PKC		8664	EIF3D	tgatyqdkryTnkyssqfggg
IPI00006204	315	320	unsp+PKC->	unsp+PKC->		9196	KCNAB3	gyqwlkdkvqSedgkkqqakv
IPI00006213	399	398	PKC->CKI	PKC->ATM		5108	PCM1	rlpdekvelfSkmrvlqekkq
IPI00006252	33	30	PKC+cdc2->PKA+cdc2	PKC+cdc2->cdc2		9255	AIMP1	qiieylkqqvSllkekailqa
IPI00006379	441	440		unsp+PKC->unsp+PKC+DNAPK		51602	NOP58	psgdstlptcSkkrkieqvdk
IPI00006379	460	455	unsp+CKI+PKC->unsp+CKI	unsp+CKI+PKC->unsp+PKC		51602	NOP58	ieqvdkedeiTekkakkakik
IPI00006379	461	455	unsp+CKI+PKC->unsp+PKC+CKII	unsp+CKI+PKC->unsp+PKC		51602	NOP58	ieqvdkedeiTekkakkakik
IPI00006440	208	199	->cdc2			51081	MRPS7	tecrdkkhqrTlmpeklshkl
IPI00006440	208	206	unsp->cdc2+PKA	unsp->PKA		51081	MRPS7	hqrtlmpeklShklleafhnq
IPI00006475	144	142	unsp->	unsp->DNAPK		51619	UBE2D4	ekynrlarewTqkyam-----
IPI00006579	60	56	->PKA	->PKA		1327	COX4I1	lpevahvkhlSasqkalkeke
IPI00006579	60	58	ATM+unsp+PKC+DNAPK->ATM+PKC+DNAPK	ATM+unsp+PKC+DNAPK->ATM+PKC+DNAPK		1327	COX4I1	evahvkhlsaSqkalkekeka
IPI00006601	153	154		->unsp		1114	CHGB	sdsqvseevkTrhseksqred
IPI00006601	153	157	unsp+PKC+PKA->unsp+PKA	unsp+PKC+PKA->unsp+PKC		1114	CHGB	qvseevktrhSeksqredeee
IPI00006601	153	160	unsp+CKI+CKII+DNAPK->ATM+unsp+CKI+CKII+DNAPK	unsp+CKI+CKII+DNAPK->ATM+unsp+CKI+CKII+DNAPK		1114	CHGB	eevktrhsekSqredeeeeeg
IPI00006663	355	354		unsp+CKII->unsp+CKII+DNAPK		217	ALDH2	ksrvvgnpfdSkteqgpqvde
IPI00006863	166	161	unsp+PKC+cdc2->unsp+cdc2	unsp+PKC+cdc2->unsp+cdc2	PhosphoSite	9552	SPAG7	qqgpvvvspaSdykdkyshli
IPI00006865	38	35	PKC+cdc2+DNAPK->ATM+cdc2+DNAPK	PKC+cdc2+DNAPK->ATM+cdc2+DNAPK	Uniprot|PhosphoSite	9554	SEC22B	qsgrdlqqyqSqakqlfrkln
IPI00006932	188	182		unsp+CKII->unsp		51631	LUC7L2	krlaetqeeiSaevaakaerv
IPI00006935	47	44	unsp+PKC->unsp	unsp+PKC->unsp		56648	EIF5A2	kgrpckivemStsktgkhgha
IPI00006935	47	45	unsp+PKC->	unsp+PKC->		56648	EIF5A2	grpckivemsTsktgkhghak
IPI00006935	47	46		->ATM+DNAPK		56648	EIF5A2	rpckivemstSktgkhghakv
IPI00006935	47	48		PKC->unsp+PKC		56648	EIF5A2	ckivemstskTgkhghakvhl
IPI00006935	68	65	PKC->PKC+CKII	PKC->PKC+CKII		56648	EIF5A2	kvhlvgidifTgkkyedicps
IPI00006937	8	5	->EGFR	->EGFR	PhosphoSite	51465	UBE2J1	------metrYnlkspavkrl
IPI00006937	8	9	unsp+GSK3+cdk5+p38MAPK->GSK3+p38MAPK	unsp+GSK3+cdk5+p38MAPK->cdk5+p38MAPK		51465	UBE2J1	--metrynlkSpavkrlmkea
IPI00006952	247	246	unsp->unsp+EGFR	unsp->unsp+EGFR		51110	LACTB2	ftvmelvkiiYkntpenlhem
IPI00006952	247	249	unsp->			51110	LACTB2	melvkiiyknTpenlhemakh
IPI00006980	20	23	->CKII	->CKII		51637	C14orf166	npagfnckdeTefrnfivwle
IPI00006987	17	13	->unsp+PKA	->unsp+PKG		57062	DDX24	lkdtksrpkqSscgkfqtkgi
IPI00006987	17	14	PKC->			57062	DDX24	kdtksrpkqsScgkfqtkgik
IPI00006987	71	67	PKC->	PKC->unsp		57062	DDX24	qlvspaknpsSlfskeapkrk
IPI00006987	71	70	unsp+PKC->unsp+CKI+PKC	unsp+PKC->ATM+unsp+PKC+DNAPK		57062	DDX24	spaknpsslfSkeapkrkaqa
IPI00007004	208	203	PKC->			51018	RRP15	ssmrkrakliStvskkdfisv
IPI00007004	208	206	unsp+PKC->unsp	unsp+PKC->unsp		51018	RRP15	rkraklistvSkkdfisvlrg
IPI00007058	233	236	PKC->			57175	CORO1B	aifladgkvfTtgfsrmserq
IPI00007058	233	240	unsp->unsp+cdc2	unsp->unsp+cdc2		57175	CORO1B	adgkvfttgfSrmserqlalw
IPI00007074	146	138	unsp+cdc2+RSK->unsp+PKA+cdc2+RSK			8565	YARS	eytldvyrlsSvvtqhdskka
IPI00007074	146	141	CKI+PKC->	CKI+PKC->		8565	YARS	ldvyrlssvvTqhdskkagae
IPI00007074	146	145		unsp->ATM+unsp		8565	YARS	rlssvvtqhdSkkagaevvkq
IPI00007074	147	141	CKI+PKC->PKC	CKI+PKC->PKC		8565	YARS	ldvyrlssvvTqhdskkagae
IPI00007074	147	145	unsp->cdc2	unsp->		8565	YARS	rlssvvtqhdSkkagaevvkq
IPI00007074	197	193	PKC+PKG->PKG	PKC+PKG->PKG		8565	YARS	fggidqrkifTfaekylpalg
IPI00007074	197	198	unsp->	unsp->		8565	YARS	qrkiftfaekYlpalgyskrv
IPI00007074	197	205	->cdc2	->cdc2		8565	YARS	aekylpalgySkrvhlmnpmv
IPI00007074	206	205	->cdc2+PKA	->ATM		8565	YARS	aekylpalgySkrvhlmnpmv
IPI00007074	474	472	EGFR->			8565	YARS	apgehvfvkgYekgqpdeelk

IPI00007088	169	165	unsp+CKI->unsp			10393	ANAPC10	qikiytpveeSsigkfprctt
IPI00007088	169	166	unsp+CKI->CKI			10393	ANAPC10	ikiytpveesSigkfprctti
IPI00007088	169	174	unsp+PKC+PKG+PKA->unsp	unsp+PKC+PKG+PKA->unsp+PKG		10393	ANAPC10	essigkfprcTtidfmmyrsi
IPI00007088	169	175		->CaM-II		10393	ANAPC10	ssigkfprctTidfmmyrsir
IPI00007123	86	83	->PKA		PhosphoSite	27330	RPS6KA6	fellkvlgqgSfgkvflvrkk
IPI00007128	197	192	->PKA			5582	PRKCG	nlipmdpnglSdpyvklklip
IPI00007188	23	22	->PKA	->DNAPK		292	SLC25A5	flaggvaaaiSktavapierv
IPI00007188	96	95		INSR+unsp->INSR+unsp+EGFR		292	SLC25A5	qalnfafkdkYkqiflggvdk
IPI00007188	105	107	PKG->	PKG->		292	SLC25A5	qiflggvdkrTqfwryfagnl
IPI00007188	166	167	->PKC	->PKC		292	SLC25A5	lgdclvkiykSdgikglyqgf
IPI00007189	135	138	p38MAPK->GSK3+p38MAPK			998	CDC42	klaknkqkpiTpetaeklard
IPI00007189	144	138		p38MAPK->GSK3+p38MAPK		998	CDC42	klaknkqkpiTpetaeklard
IPI00007247	60	65	unsp+PKC+PKG->unsp+PKC			5096	PCCB	idaqhkrgklTarerisllld
IPI00007311	53	45	unsp->unsp+PKA			4097	MAFG	relnqhlrglSkeeivqlkqr
IPI00007311	60	58		unsp+PKC+PKG+PKA->unsp+PKG+PKA		4097	MAFG	eivqlkqrrrTlknrgyaasc
IPI00007311	60	64	unsp+EGFR->	unsp+EGFR->EGFR		4097	MAFG	qrrrtlknrgYaascrvkrvt
IPI00007311	71	74	unsp+PKC->unsp+PKC+CKII	unsp+PKC->unsp+PKC+CKII		4097	MAFG	yaascrvkrvTqkeelekqka
IPI00007311	76	74	unsp+PKC->unsp+CKII	unsp+PKC->unsp+CKII+DNAPK		4097	MAFG	yaascrvkrvTqkeelekqka
IPI00007334	359	350	unsp+PKC->unsp+PKC+cdc2			22985	ACIN1	ftrsqeearkShlarqqqeke
IPI00007334	359	363		CKII->		22985	ACIN1	arqqqekemkTtspleeeere
IPI00007334	717	720	PKC->		Uniprot|PhosphoSite	22985	ACIN1	sqpesaekhvTqrlqpergsp
IPI00007611	54	50	PKC->	PKC->unsp		539	ATP5O	ryatalysaaSkqnkleqvek
IPI00007611	158	163	PKC->PKC+cdc2	PKC->PKC+cdc2		539	ATP5O	tlselktvlkSflsqgqvlkl
IPI00007611	162	159	PKC->			539	ATP5O	leeatlselkTvlksflsqgq
IPI00007611	162	163	PKC->PKC+cdc2	PKC->PKC+cdc2		539	ATP5O	tlselktvlkSflsqgqvlkl
IPI00007611	162	166		CKI+DNAPK->ATM+CKI+DNAPK		539	ATP5O	elktvlksflSqgqvlkleak
IPI00007611	172	163	PKC->PKC+cdc2			539	ATP5O	tlselktvlkSflsqgqvlkl
IPI00007611	172	166	CKI+DNAPK->DNAPK	CKI+DNAPK->ATM+CKI+DNAPK		539	ATP5O	elktvlksflSqgqvlkleak
IPI00007641	3	6	GSK3->GSK3+cdk5	GSK3->p38MAPK		51135	IRAK4	-----mnkpiTpstyvrclnv
IPI00007641	3	8	PKC+PKA->PKC	PKC+PKA->PKA		51135	IRAK4	---mnkpitpStyvrclnvgl
IPI00007641	34	43	unsp->unsp+PKA			51135	IRAK4	kklavaikkpSgddrynqfhi
IPI00007675	429	427	unsp+PKC->	unsp+PKC->		51143	DYNC1LI1	vasvspipagSkkidpnmkag
IPI00007694	6	7	unsp->	unsp->		51400	PPME1	----msalekSmhlgrlpsrp
IPI00007694	30	22	->cdc2			51400	PPME1	rlpsrpplpgSggsqsgakmr
IPI00007694	30	25	ATM+unsp+CKI+DNAPK->ATM+CKI+DNAPK	ATM+unsp+CKI+DNAPK->ATM+CKI+DNAPK		51400	PPME1	srpplpgsggSqsgakmrmgp
IPI00007722	516	514	unsp+PKC->unsp+PKA	unsp+PKC->unsp+PKA		271	AMPD2	eifiktdnrvSgkyfahiike
IPI00007750	40	38	PKC->	PKC->		7277	TUBA4A	hgiqpdgqmpSdktigggdds
IPI00007750	40	41	->CKI	->unsp		7277	TUBA4A	qpdgqmpsdkTigggddsftt
IPI00007750	163	158	unsp+cdc2->unsp+PKA			7277	TUBA4A	gftsllmerlSvdygkkskle
IPI00007750	163	165	unsp+PKC+PKG+CKII->unsp+PKC+CKII	unsp+PKC+PKG+CKII->unsp+CKII		7277	TUBA4A	erlsvdygkkSklefsiypap
IPI00007750	280	282	EGFR->unsp	EGFR->unsp+EGFR	PhosphoSite	7277	TUBA4A	yapvisaekaYheqlsvaeit
IPI00007750	311	312		->INSR		7277	TUBA4A	mvkcdprhgkYmacclIyrgd
IPI00007750	401	399	unsp->			7277	TUBA4A	arldhkfdlmYakrafvhwyv
IPI00007752	58	51	SRC+INSR+EGFR->SRC+EGFR	SRC+INSR+EGFR->SRC+EGFR	Uniprot|PhosphoSite	10383	TUBB2C	dlqlerinvyYneatggkyvp
IPI00007752	58	59	unsp->	unsp->		10383	TUBB2C	vyyneatggkYvpravlvdle
IPI00007752	103	106	unsp->		PhosphoSite	10383	TUBB2C	gagnnwakghYtegaelvdsv
IPI00007752	103	107	->CKII			10383	TUBB2C	agnnwakghyTegaelvdsvl
IPI00007752	379	382	unsp+PKA->PKA	unsp+PKA->PKA	PhosphoSite	10383	TUBB2C	taiqelfkriSeqftamfrrk
IPI00007756	101	94	PKC->PKC+cdc2			57403	RAB22A	vyditkeetfStlknwvkelr
IPI00007757	288	279		unsp+PKC+PKG+cdc2+PKA+RSK->unsp+PKC+PKG+PKA+RSK		22807	IKZF2	ltgnmgkrksStpqkfvgekl
IPI00007765	121	116	PKC->	PKC->		3313	HSPA9	krqavtnpnnTfyatkrligr
IPI00007765	121	118		unsp+EGFR->EGFR		3313	HSPA9	qavtnpnntfYatkrligrry
IPI00007765	300	299	->PKA	->DNAPK		3313	HSPA9	efkretgvdlTkdnmalqrvr
IPI00007765	345	347		PKC->		3313	HSPA9	sgpkhlnmklTraqfegivtd
IPI00007765	595	594	unsp+PKC+CKII->unsp+CKII	unsp+PKC+CKII->unsp+CKII+DNAPK		3313	HSPA9	maegiihdteTkmeefkdqlp
IPI00007765	646	639	unsp+PKC->unsp+PKC+cdc2			3313	HSPA9	tgenirqaasSlqqaslklfe
IPI00007765	646	644	unsp->	unsp->		3313	HSPA9	rqaasslqqaSlklfemaykk
IPI00007797	17	16		->ATM+DNAPK		2171	FABP5	qlegrwrlvdSkgfdeymkel
IPI00007797	55	53	PKC->	PKC->		2171	FABP5	ciitcdgknlTiktestlktt
IPI00007797	55	58	unsp+PKC->PKC	unsp+PKC->PKC		2171	FABP5	dgknltikteStlkttqfsct
IPI00007797	55	59	unsp+PKC->unsp+CKI	unsp+PKC->unsp+CKI+PKC		2171	FABP5	gknltiktesTlkttqfsctl
IPI00007811	35	36	PKC->PKC+p38MAPK	PKC->PKC+p38MAPK		1019	CDK4	phsghfvalkSvrvpnggggg
IPI00007927	114	115	unsp->	unsp->		10592	SMC2	rqvviggrnkYlingvnannt
IPI00007927	320	313	ATM+unsp+DNAPK->unsp+DNAPK	ATM+unsp+DNAPK->unsp+DNAPK		10592	SMC2	laeaqrvntkSqsafdlkkkn
IPI00007927	321	313	ATM+unsp+DNAPK->unsp+DNAPK			10592	SMC2	laeaqrvntkSqsafdlkkkn
IPI00007927	330	329		->DNAPK		10592	SMC2	lkkknlaceeSkrkeleknmv
IPI00007927	677	669		DNAPK->ATM+DNAPK		10592	SMC2	phgtlsggarSqaasiltkfq
IPI00007927	677	676	PKC->	PKC->		10592	SMC2	garsqaasilTkfqelkdvqd
IPI00007927	1158	1159		PKC->unsp+PKC		10592	SMC2	mfnnanvlfkTkfvdgvstva
IPI00007927	1160	1159	PKC->	PKC->		10592	SMC2	mfnnanvlfkTkfvdgvstva
IPI00007940	269	265		unsp->SRC+unsp		10613	ERLIN1	rekakadaeyYaahkyatsnk
IPI00007940	269	270	unsp->			10613	ERLIN1	adaeyyaahkYatsnkhkltp
IPI00007941	165	168	unsp->	unsp->		10614	HEXIM1	skkkrhwkpyYkltweekkkf
IPI00007941	165	171		unsp+PKC+CKII->unsp+PKC		10614	HEXIM1	krhwkpyyklTweekkkfdek
IPI00007983	282	279	unsp+cdk5+p38MAPK->cdk5+p38MAPK	unsp+cdk5+p38MAPK->cdk5+p38MAPK		64236	PDLIM2	sslpasralaTppklhtcekc
IPI00008054	105	102	unsp+cdc2->unsp+PKA+cdc2	unsp+cdc2->unsp+PKA+cdc2		27154	BRPF3	kpsskgkkkeScskhasgtsf
IPI00008054	105	104	unsp+PKC->PKC	unsp+PKC->ATM+unsp+DNAPK		27154	BRPF3	sskgkkkescSkhasgtsfhl
IPI00008054	105	108	PKC->			27154	BRPF3	kkkescskhaSgtsfhlpqps
IPI00008054	447	440	unsp+CKI+PKC->unsp+CKI+PKC+cdc2			27154	BRPF3	eqeaqggvsgSlkgvpkksk m
IPI00008054	447	451	unsp+PKC+PKA->unsp+PKC	unsp+PKC+PKA->unsp+PKC		27154	BRPF3	lkgvpkkskmSlkqkikkepe
IPI00008054	449	440	unsp+CKI+PKC->unsp+CKI+PKC+cdc2			27154	BRPF3	eqeaqggvsgSlkgvpkkskm
IPI00008054	449	448	unsp->unsp+PKA			27154	BRPF3	sgslkgvpkkSkmslkqkikk
IPI00008054	449	451	unsp+PKC+PKA->unsp+CKI+PKC	unsp+PKC+PKA->unsp+CKI+PKC		27154	BRPF3	lkgvpkkskmSlkqkikkepe
IPI00008054	546	543	unsp+CKII->CKII			27154	BRPF3	aeqreqdektSavkeelkywq
IPI00008054	546	551	unsp->unsp+EGFR			27154	BRPF3	ktsavkeelkYwqklrhdler
IPI00008054	550	543	unsp+CKII->unsp+cdc2+CKII			27154	BRPF3	aeqreqdektSavkeelkywq
IPI00008054	554	551		unsp->unsp+EGFR		27154	BRPF3	ktsavkeelkYwqklrhdler
IPI00008054	671	673		->unsp		27154	BRPF3	tncmkynakdTifhraavrlr
IPI00008091	118	117		PKC->ATM+PKC+DNAPK		54617	INO80	kcdkgnlynfSklkksrkwlk
IPI00008091	118	122	unsp+PKC+PKG+PKA->unsp+PKC+PKG	unsp+PKC+PKG+PKA->unsp+PKC+PKG+cdc2		54617	INO80	nlynfsklkkSrkwlkslls
IPI00008240	726	731	unsp+PKC+PKA->PKC	unsp+PKC+PKA->		4141	MARS	vnepwkrikgSeadrqragtv
IPI00008248	263	256		unsp+RSK->unsp+PKA+RSK		51434	ANAPC7	vhtgdnsraiSticslekksl
IPI00008248	263	260	PKC+DNAPK->DNAPK			51434	ANAPC7	dnsraisticSlekksllrdn
IPI00008274	209	208	unsp+CKI+cdc2->CKI+cdc2	unsp+CKI+cdc2->ATM+unsp+CKI+cdc2+DNAPK		10487	CAP1	kefhttglawSktgpvakels
IPI00008274	209	210	->PKC	->PKC		10487	CAP1	fhttglawskTgpvakelsgl
IPI00008359	193	189	PKC->	PKC->		51350	KRT76	vkaqereqikTlnnkfasfid
IPI00008359	193	196	unsp+PKC->unsp			51350	KRT76	qiktlnnkfaSfidkvrfleq
IPI00008380	4	7	->CKII	->CKII		5515	PPP2CA	----mdekvfTkeldqwieql
IPI00008433	47	52		unsp->unsp+cdc2		6193	RPS5	kekyakylphSagryaakrfr
IPI00008433	182	184	PKG+PKA->	PKG+PKA->		6193	RPS5	adelinaakgSsnsyaikkkd
IPI00008433	182	187		CKI+PKC+DNAPK->PKC+DNAPK		6193	RPS5	linaakgssnSyaikkkdele
IPI00008454	43	45		->EGFR		51726	DNAJB11	sasikdikkaYrklalqlhpd
IPI00008475	46	47	unsp->	unsp->		3157	HMGCS1	ekydgvdagkYtiglgqakmg
IPI00008475	330	328	unsp+PKC->	unsp+PKC->DNAPK		3157	HMGCS1	afmkasselfSqktkasllvs
IPI00008475	330	334		PKA->		3157	HMGCS1	selfsqktkaSllvsnqngnm
IPI00008475	409	414	unsp+PKG+PKA+RSK->unsp+PKA+CaM-II+RSK			3157	HMGCS1	slcdlksrldSrtgvapdvfa
IPI00008524	104	96	unsp->unsp+PKA		Uniprot|PhosphoSite	26986	PABPC1	wsqrdpslrkSgvgnifiknl
IPI00008524	104	109	unsp->	unsp->		26986	PABPC1	gnifiknldkSidnkalydtf
IPI00008524	259	262	unsp->	unsp->		26986	PABPC1	ngkelngkqiYvgraqkkver
IPI00008528	54	56		PKC->		4509	ATP8	nynkpwepkwTkicslhslpp
IPI00008528	54	60	->PKA			4509	ATP8	pwepkwtkicSlhslppqs--
IPI00008529	21	19	unsp+PKC->unsp	unsp+PKC->unsp	PhosphoSite	6181	RPLP2	laalggnsspSakdikkilds
IPI00008529	24	19	unsp+PKC->unsp		PhosphoSite	6181	RPLP2	laalggnsspSakdikkilds
IPI00008529	24	29	PKA->	PKA->		6181	RPLP2	sakdikkildSvgieadddrl
IPI00008529	49	44	unsp->	unsp->		6181	RPLP2	adddrlnkviSelngkniedv
IPI00008531	418	413	PKC->	PKC->		23186	RCOR1	qaisdvignkSvvqvknffvn
IPI00008552	92	83	->cdc2			10539	GLRX3	vpevsekyeiSsvptflffkn
IPI00008552	92	87	PKC->			10539	GLRX3	sekyeissvpTflffknsqki
IPI00008575	175	183	unsp+PKC->unsp+PKC+cdc2			10657	KHDRBS1	vgkilgpqgnTikrlqeetga
IPI00008599	221	217	unsp+PKC->unsp	unsp+PKC->PKC		10682	EBP	avkhlthaqsTldakatkaks
IPI00008599	221	223		PKC->unsp+PKC		10682	EBP	haqstldakaTkakskkn---
IPI00008603	63	54	unsp->unsp+cdc2			59	ACTA2	gvmvgmgqkdSyvgdeaqskr
IPI00008603	63	62	unsp+PKC+cdc2->unsp+CKI+cdc2	unsp+PKC+cdc2->ATM+unsp+cdc2+DNAPK		59	ACTA2	kdsyvgdeaqSkrgiltlkyp
IPI00008603	328	325	unsp+PKC+cdc2->cdc2	unsp+PKC+cdc2->unsp+cdc2	Uniprot	59	ACTA2	mqkeitalapStmkikiiapp
IPI00008603	328	326	PKC->	PKC->		59	ACTA2	qkeitalapsTmkikiiappe
IPI00008708	461	464	PKC->		PhosphoSite	26156	RSL1D1	tpkkpeakffTtpsksvrkas
IPI00008708	461	465		unsp+cdk5->unsp+cdk5+p38MAPK	iiprot|PHOsphoELM|PhosphoSi	26156	RSL1D1	pkkpeakffTpsksvrkash
IPI00008708	468	464	PKC->	PKC->	PhosphoSite	26156	RSL1D1	tpkkpeakffTtpsksvrkas

Table S3

IPI00008708	468	465	unsp+cdk5->unsp+p38MAPK	unsp+cdk5->unsp	niprot|PHOsphoELM|PhosphoSi	26156	RSL1D1	pkkpeakfftTpsksvrkash
IPI00008708	468	467	PKC+cdc2->PKC+cdc2+DNAPK	PKC+cdc2->PKC+cdc2+DNAPK	Uniprot|PhosphoSite	26156	RSL1D1	kpeakffttpSksvrkashtp
IPI00008708	468	474	unsp+PKC+PKG+RSK->unsp+PKC+PKG+PKA+RSK			26156	RSL1D1	ttpsksvrkaShtpkkwpkkp
IPI00008728	178	179	INSR+unsp->unsp			10845	CLPX	pkkiynyldkYvvgqsfakkv
IPI00008934	83	76	INSR+unsp->unsp	INSR+unsp->unsp		3158	HMGCS2	qyvdqtdlekYnnveagkytv
IPI00008934	83	84	unsp->	unsp->		3158	HMGCS2	ekynnveagkYtvglgqtrmg
IPI00008934	437	440	->cdc2	->cdc2		3158	HMGCS2	apgspldklvSstsdlpkrla
IPI00008934	437	442		->cdc2		3158	HMGCS2	gspldklvssTsdlpkrlasr
IPI00008965	268	267	unsp+PKC+cdc2->PKC+cdc2	unsp+PKC+cdc2->unsp+PKC+cdc2+DNAPK		3725	JUN	rkrmrnriaaSkcrkrkleri
IPI00008965	271	267	unsp+PKC+cdc2->unsp+PKC			3725	JUN	rkrmrnriaaSkcrkrkleri
IPI00009032	116	112	PKC->PKA	PKC->unsp+PKA		6741	SSB	deykndvknrSvyikgfptda
IPI00009032	116	114	EGFR->			6741	SSB	ykndvknrsvYikgfptdatl
IPI00009032	328	331	unsp+PKC->PKC	unsp+PKC->PKC		6741	SSB	dqqeslnkwkSkgrrfkgkgk
IPI00009032	360	362		PKC->	niprot|PHOsphoELM|PhosphoSi	6741	SSB	kgkvqfqgkkTkfasddehde
IPI00009104	417	410	PKC->PKC+cdc2			10856	RUVBL2	ryaiqlitaaSlvcrkrkgte
IPI00009104	417	419	unsp+PKG+PKB+PKA+RSK->unsp+PKB	unsp+PKG+PKB+PKA+RSK->unsp+PKB		10856	RUVBL2	aslvcrkrkgTevqvddikrv
IPI00009236	5	2	PKC->PKA	PKC->		857	CAV1	---------mSggkyvdsegh
IPI00009236	5	9	PKA->	PKA->		857	CAV1	--msggkyvdSeghlytvpir
IPI00009247	31	34	INSR+unsp+EGFR->	INSR+unsp+EGFR->SRC+unsp		9556	C14orf2	kniwipmkpyYtkvyqeiwig
IPI00009286	636	633	cdc2->	cdc2->		4297	MLL	khsrsepqyfSsakyakegli
IPI00009286	636	634	PKC->cdc2	PKC->cdc2		4297	MLL	hsrsepqyfsSakyakeglir
IPI00009286	1133	1136	PKC->			4297	MLL	appikpikpvTrnkapqeppv
IPI00009286	1235	1231	cdc2->			4297	MLL	ktsekkdskeSsvvknvvdss
IPI00009286	1235	1232	unsp+PKC->unsp	unsp+PKC->unsp		4297	MLL	tsekkdskesSvvknvvdssq
IPI00009286	1235	1240	unsp->	unsp->cdc2		4297	MLL	essvvknvvdSsqkptpsare
IPI00009305	64	62	unsp+PKC->PKC	unsp+PKC->PKC		10007	GNPDA1	lieyykngdlSfkyvktfnmd
IPI00009328	60	62	unsp+PKG->	unsp+PKG->unsp		9775	EIF4A3	giyaygfekpSaiqqraikqi
IPI00009328	296	294	unsp+PKC->CKII	unsp+PKC->		9775	EIF4A3	cntkrkvdwlTekmreanftv
IPI00009328	321	328	->PKA	->PKA		9775	EIF4A3	simkefrsgaSrvlistdvwa
IPI00009373	69	64	unsp+EGFR->unsp	unsp+EGFR->unsp		64769	MEAF6	ygniirgwdrYltnqknsnsk
IPI00009373	69	66	PKC+RSK->	PKC+RSK->PKC		64769	MEAF6	niirgwdrylTnqknsnsknd
IPI00009373	69	71	unsp+cdc2->unsp+PKC+cdc2			64769	MEAF6	wdryltnqknSnskndrrnrk
IPI00009373	74	71	unsp+cdc2->unsp+PKA+cdc2			64769	MEAF6	wdryltnqknSnskndrrnrk
IPI00009373	74	73	unsp->unsp+DNAPK	unsp->unsp+DNAPK		64769	MEAF6	ryltnqknsnSkndrrnrkfk
IPI00009373	91	90	unsp+PKC+PKA+RSK->unsp+PKA+RSK	unsp+PKC+PKA+RSK->unsp+PKA+DNAPK+RSK		64769	MEAF6	rkfkeaerlfSkssvtsaaav
IPI00009373	91	92	->cdc2			64769	MEAF6	fkeaerlfskSsvtsaaavsa
IPI00009373	91	93	cdc2->PKC+cdc2			64769	MEAF6	keaerlfsksSvtsaaavsal
IPI00009373	91	95	PKC->			64769	MEAF6	aerlfskssvTsaaavsalag
IPI00009439	237	236	unsp+CKI->unsp+CKI+PKA	unsp+CKI->ATM+unsp+CKI+DNAPK		6857	SYT1	vmavydfdrfSkhdiigefkv
IPI00009448	81	76	PKC->	PKC->		7128	TNFAIP3	kalidrniqaTlesqkklnwc
IPI00009448	81	79	PKC->	PKC->DNAPK		7128	TNFAIP3	idrniqatleSqkklnwcrev
IPI00009634	135	137	unsp->			58472	SQRDL	cihtdddekiSyryliialgi
IPI00009634	135	138	INSR->	INSR->		58472	SQRDL	ihtdddekisYryliialgiq
IPI00009634	173	168	PKC+DNAPK->PKA+DNAPK	PKC+DNAPK->PKC+PKA+DNAPK		58472	SQRDL	pegfahpkigSnysvktvekt
IPI00009634	173	171	unsp->	unsp->		58472	SQRDL	fahpkigsnySvktvektwka
IPI00009634	180	178	unsp+PKC->	unsp+PKC->		58472	SQRDL	snysvktvekTwkalqdfkeg
IPI00009688	89	86	PKC->	PKC->		5305	PIP4K2A	nhlfnkenmpShfkfkeycpm
IPI00009688	91	86	PKC->	PKC->		5305	PIP4K2A	nhlfnkenmpShfkfkeycpm
IPI00009688	145	138	INSR->	INSR->		5305	PIP4K2A	arsgarfhtsYdkryiiktit
IPI00009688	145	142	unsp->unsp+EGFR	unsp->EGFR		5305	PIP4K2A	arfhtsydkrYiiktitsedv
IPI00009688	145	146		->unsp		5305	PIP4K2A	tsydkryiikTitsedvaemh
IPI00009688	145	148	unsp->	unsp->unsp+CKII		5305	PIP4K2A	ydkryiiktiTsedvaemhni
IPI00009688	145	149	unsp+CKII->CKII	unsp+CKII->unsp+cdc2+CKII		5305	PIP4K2A	dkryiiktitSedvaemhnil
IPI00009713	61	52		cdc2->		6662	SOX9	dtentrpqenTfpkgepdlkk
IPI00009713	398	395	PKC+cdc2->cdc2	PKC+cdc2->cdc2		6662	SOX9	lssepgqsqrThikteqlsps
IPI00009713	398	405	unsp->unsp+cdc2			6662	SOX9	thikteqlspShyseqqqhsp
IPI00009724	172	176	PKC->			64800	EFCAB6	vgekvfknikTvmkafelidv
IPI00009724	175	176		PKC->unsp+PKC		64800	EFCAB6	vgekvfknikTvmkafelidv
IPI00009724	179	176	PKC->			64800	EFCAB6	vgekvfknikTvmkafelidv
IPI00009724	188	189		->PKC		64800	EFCAB6	kafelidvnkTglvrpqelrr
IPI00009771	81	79	unsp+CKII->cdc2+CKII	unsp+CKII->CKII		84823	LMNB2	lendrlllkiSekeevttrev
IPI00009771	520	518		->p38MAPK		84823	LMNB2	legeeiaykfTpkyilragqm
IPI00009790	395	393	PKC->	PKC->		5214	PFKP	rgrsfagnlnTykrlaiklpd
IPI00009790	688	687	PKC->	PKC->		5214	PFKP	apspfdrnfgTkisaramewi
IPI00009790	688	690		PKC->		5214	PFKP	pfdrnfgtkiSaramewitak
IPI00009844	291	288	unsp->unsp+PKA	unsp->unsp+PKA		51292	GMPR2	yaggvaeyraSegktvevpfk
IPI00009844	291	292		->unsp		51292	GMPR2	vaeyrasegkTvevpfkgdve
IPI00009867	178	174	PKC->	PKC->		3852	KRT5	vrteereqikTlnnkfasfid
IPI00009867	178	181	unsp+PKC->unsp			3852	KRT5	qiktlnnkfaSfidkvrfleq
IPI00009889	26	27	unsp->	unsp->		6303	SAT1	ilrlikelakYeymeeqvilt
IPI00009889	26	29	unsp+EGFR->	unsp+EGFR->		6303	SAT1	rlikelakyeYmeeqviltek
IPI00009895	121	117	DNAPK->PKA+DNAPK	DNAPK->PKA+DNAPK		51304	ZDHHC3	kgnatkefieSlqlkpgqvvy
IPI00009904	256	250	unsp->unsp+PKA	unsp->unsp+PKA		9601	PDIA4	etdlakrfdvSgyptlkifrk
IPI00009904	256	252		->EGFR		9601	PDIA4	dlakrfdvsgYptlkifrkgr
IPI00009904	256	254	unsp+PKC->	unsp+PKC->		9601	PDIA4	akrfdvsgypTlkifrkgrpy
IPI00009904	366	365	unsp->unsp+PKA	unsp->ATM+unsp+DNAPK		9601	PDIA4	lvvmqpekfqSkyeprshmmd
IPI00009904	484	482	unsp+PKC->	unsp+PKC->		9601	PDIA4	edvnaaildeSgkkfamepee
IPI00009931	15	11	unsp+PKC->unsp+PKA	unsp+PKC->unsp+PKA		81932	HDHD3	mahrlqirllTwdvkdtllrl
IPI00009943	93	98	unsp+PKC->PKC	unsp+PKC->PKC		7178	TPT1	ykkyikdymkSikgkleeqrp
IPI00009943	97	98	unsp+PKC->PKC	unsp+PKC->PKC		7178	TPT1	ykkyikdymkSikgkleeqrp
IPI00009958	47	46		->DNAPK		10987	COPS5	qqeilaakpwTkdhhyfkyck
IPI00009958	326	320		ATM+DNAPK->ATM+cdc2+DNAPK		10987	COPS5	ttieaihglmSqvikdklfnq
IPI00009960	222	219	PKC+DNAPK->PKA+DNAPK	PKC+DNAPK->PKA+DNAPK		10989	IMMT	qekqeqvkieSlaksledalr
IPI00009960	222	223	unsp->unsp+CKI			10989	IMMT	eqvkieslakSledalrqtas
IPI00009960	222	231	unsp+PKA->unsp+cdc2+PKA	unsp+PKA->unsp+cdc2+PKA		10989	IMMT	aksledalrqTasvtlqaiaa
IPI00009960	451	447	->PKA			10989	IMMT	qkleekrafdSavakalehhr
IPI00009960	506	507	unsp+CKII->unsp+cdc2+CKII	unsp+CKII->unsp+cdc2+CKII		10989	IMMT	vlrvqeqelkSefeqnlsekl
IPI00009992	272	267	PKC->	PKC->		11033	ADAP1	gymektgpkqTegfrkrwftm
IPI00010085	250	243	ATM+unsp+DNAPK->unsp+DNAPK	ATM+unsp+DNAPK->unsp+DNAPK		5326	PLAGL2	hltrhvkkshSqellkiktep
IPI00010085	269	265	PKC+cdc2->cdc2			5326	PLAGL2	dmlgllscssTvsvkeelspv
IPI00010085	269	267	unsp+PKC+CKII->unsp+CKII	unsp+PKC+CKII->unsp+CKII		5326	PLAGL2	lgllscsstvSvkeelspvlc
IPI00010085	356	347	->cdc2			5326	PLAGL2	qlppkyqlgsTsylpdklpkv
IPI00010090	263	262		PKC->ATM+DNAPK		2730	GCLM	vivksrgiikSkgyilqakrr
IPI00010090	263	265		->EGFR		2730	GCLM	ksrgiikskgYilqakrrgs-
IPI00010157	81	79	EGFR->	EGFR->unsp+EGFR		4144	MAT2A	geitsraavdYqkvvreavkh
IPI00010158	102	101		PKC+PKA->ATM+PKC+PKA+DNAPK		54108	CHRAC1	adilpkkilaSkylkmlkeek
IPI00010188	90	89	unsp+PKC->PKC	unsp+PKC->ATM+PKC		57380	MRS2	lasvapvftvTkfdkqgnvts
IPI00010188	93	89	unsp+PKC->unsp	unsp+PKC->PKC		57380	MRS2	lasvapvftvTkfdkqgnvts
IPI00010188	93	98	PKC+CKII->CKII	PKC+CKII->CKII		57380	MRS2	vtkfdkqgnvTsferkktely
IPI00010188	93	99	PKC->CKI+PKC	PKC->CKI+PKC		57380	MRS2	tkfdkqgnvtSferkktelyq
IPI00010190	291	287	PKC->	PKC->		26063	DECR2	ngvkglpdfaSfsakl-----
IPI00010190	291	289	PKC->	PKC->		26063	DECR2	vkglpdfasfSakl-------
IPI00010196	158	150	ATM+DNAPK->cdc2+DNAPK			8204	NRIP1	fssrlqtvalSqqirqslkeq
IPI00010196	158	156	unsp+PKC+PKG+PKA->unsp+PKG+PKA	unsp+PKC+PKG+PKA->unsp+PKA		8204	NRIP1	tvalsqqirqSlkeqgyalsh
IPI00010196	158	162	unsp->			8204	NRIP1	qirqslkeqgYalshdslkve
IPI00010196	286	279	INSR->	INSR->		8204	NRIP1	llsseahlqqYsrehalktqn
IPI00010196	310	314	PKC+cdc2->cdc2			8204	NRIP1	lqengqkdvgSyqlpkgmssh
IPI00010196	446	444	PKC->	PKC->		8204	NRIP1	sysncvpidlSckhrtekses
IPI00010196	446	449	unsp+PKC->			8204	NRIP1	vpidlsckhrTeksesdqpvs
IPI00010196	481	487	->cdc2	->cdc2	Uniprot	8204	NRIP1	dvdikedqdtSknsklnshqk
IPI00010196	528	527	->PKA	->ATM+DNAPK		8204	NRIP1	tspqgvhndvSkfntqnyart
IPI00010196	606	603	unsp+PKC->	unsp+PKC->PKC		8204	NRIP1	ntasnhsmdlTkskdppgekp
IPI00010196	606	605	PKC->	PKC->ATM+DNAPK		8204	NRIP1	asnhsmdltkSkdppgekpaq
IPI00010196	931	925	unsp->unsp+CKII			8204	NRIP1	hgsasesehrSwareskspnv
IPI00010196	931	930	unsp+PKC+cdc2+PKA->unsp+cdc2+PKA+DNAPK	unsp+PKC+cdc2+PKA->unsp+DNAPK		8204	NRIP1	esehrswareSksfnvlkqll
IPI00010204	23	24		CKII->unsp+CKII		6428	SFRS3	vgnlgnngnkTelerafgyyg
IPI00010214	27	32	INSR->		Uniprot|PhosphoSite	57402	S100A14	ietliknfhqYsveggketlt
IPI00010214	27	33	unsp+CKI->unsp+CKI+cdc2			57402	S100A14	etliknfhqySveggketltp
IPI00010252	252	251	PKG->	PKG->		51592	TRIM33	cieahqrvkfTkdhlirkked
IPI00010252	763	764		PKC->unsp+PKC		51592	TRIM33	cgssgrtaekTslsfksdqvk
IPI00010252	763	765	unsp+cdc2->cdc2			51592	TRIM33	gssgrtaektSlsfksdqvkv
IPI00010252	763	767		unsp+PKC->unsp+PKC+cdc2		51592	TRIM33	sgrtaektslSfksdqvkvkq
IPI00010252	769	760	->cdc2			51592	TRIM33	srgscgssgrTaektslsfks
IPI00010252	769	764	PKC->	PKC->		51592	TRIM33	cgssgrtaekTslsfksdqvk
IPI00010252	769	765	unsp+cdc2->unsp+PKA			51592	TRIM33	gssgrtaektSlsfksdqvkv
IPI00010252	769	767	unsp+PKC->PKC+cdc2	unsp+PKC->PKC		51592	TRIM33	sgrtaektslSfksdqvkvkq
IPI00010252	951	949	unsp+PKC->unsp+cdc2	unsp+PKC->unsp		51592	TRIM33	veydcdnlqhSkkgktaqgls
IPI00010252	953	959	unsp+cdk5+p38MAPK->unsp+cdk5			51592	TRIM33	skkgktaqglSpvdqrkcerl

Table S3

IPI00010320	41	46		unsp->unsp+CKII		10951	CBX1	veyllkwkgfSedntwepee
IPI00010320	84	89	unsp+PKA+CKII->unsp+CKII	unsp+PKA+CKII->unsp+CKII	niprot|PHOsphoELM|PhosphoSi	10951	CBX1	kseggkrkadSdsedkgeesk
IPI00010320	139	141	unsp+PKA->unsp+CKII	unsp+PKA->unsp+CKII		10951	CBX1	elmflmkwknSdeadlvpake
IPI00010349	102	98		unsp->unsp+EGFR		8540	AGPS	qevmkwngwgYndskfifnkk
IPI00010349	102	101		->ATM+DNAPK		8540	AGPS	mkwngwgyndSkfifnkkgqi
IPI00010349	169	171	->PKC			8540	AGPS	nedflhdlkeTnisysqeadd
IPI00010349	169	174	unsp+PKA->cdc2	unsp+PKA->cdc2		8540	AGPS	flhdlketniSysqeaddrvf
IPI00010404	17	9	ATM+unsp+CKII+DNAPK->ATM+unsp+cdc2+CKII+DNAPK		Uniprot|PhosphoSite	83443	SF3B5	--mtdrytihSqlehlqskyi
IPI00010404	17	16	->PKA	->ATM+DNAPK		83443	SF3B5	tihsqlehlqSkyigtghadt
IPI00010414	22	31	->cdc2	->cdc2		9124	PDLIM1	gkdfeqplaiSrvtpgskaal
IPI00010415	168	166	unsp+PKC->PKA	unsp+PKC->PKA		11332	ACOT7	nkatlwyvplSlknvdkvlev
IPI00010415	198	194		->EGFR		11332	ACOT7	qeqeeegrkrYeaqklermet
IPI00010420	104	107	INSR+unsp->unsp	INSR+unsp->unsp		83447	SLC25A31	qalnfafkdkYkqlfmsgvnk
IPI00010471	76	70		unsp+PKG+PKA+CKII->unsp+PKG+cdc2+PKA+CKII		3936	LCP1	tgdldqdgriSfdefikifhg
IPI00010471	88	83	unsp+PKC->PKC	unsp+PKC->PKC		3936	LCP1	efikifhglkStdvaktfrka
IPI00010471	88	84	PKC->	PKC->		3936	LCP1	fikifhglkSTdvaktfrkai
IPI00010471	294	290	->PKA			3936	LCP1	nagcnkignfStdikdskayy
IPI00010471	294	291	->CKII	->CKII		3936	LCP1	agcnkignfsTdikdskayyh
IPI00010471	294	296	PKC+PKA->PKC	PKC+PKA->		3936	LCP1	ignfstdikdSkayyhllеqv
IPI00010471	297	296	PKC+PKA->PKA	PKC+PKA->PKA+DNAPK		3936	LCP1	ignfstdikdSkayyhlleqv
IPI00010471	468	474	->cdc2			3936	LCP1	velgknqakfSlvgiggqdln
IPI00010471	472	474	->PKC	->CKI		3936	LCP1	velgknqakfSlvgiggqdln
IPI00010471	542	537	unsp+PKC+cdc2->unsp+cdc2	unsp+PKC+cdc2->unsp+cdc2		3936	LCP1	etlreaekssSissfkdpkis
IPI00010471	542	539	unsp+PKC->unsp	unsp+PKC->unsp+CKI		3936	LCP1	lreaeksssiSsfkdpkists
IPI00010471	542	540	unsp+PKC->unsp+PKC+cdc2			3936	LCP1	reaeksssisSfkdpkistsl
IPI00010471	542	547	unsp+PKA->PKA	unsp+PKA->PKA		3936	LCP1	sissfkdpkiStslpvldlid
IPI00010471	579	588	PKC->PKC+cdc2	PKC->PKC+cdc2		3936	LCP1	eklnnakyaiSmarkigarvy
IPI00010700	27	25	unsp+PKC->	unsp+PKC->		7916	BAT2	kkysslnlfdTykgksleiqk
IPI00010700	29	25	unsp+PKC->unsp			7916	BAT2	kkysslnlfdTykgksleiqk
IPI00010700	1196	1191	PKC->	PKC->		7916	BAT2	vcpgwsppakSlapkkpptgp
IPI00010700	1196	1199	->GSK3			7916	BAT2	akslapkkppTgplppskepl
IPI00010706	186	181	PKC->			2937	GSS	sktkeagkilSnnpskglalg
IPI00010706	186	185	->PKA	->DNAPK		2937	GSS	eagkilsnnpSkglalgiaka
IPI00010720	399	401	unsp+PKA->unsp	unsp+PKA->unsp		22948	CCT5	nkmiieeakrSlhdalcvirn
IPI00010740	421	420		->DNAPK		6421	SFPQ	stgkgivefaSkpaarkafer
IPI00010740	472	470	unsp->			6421	SFPQ	peklaqknpmYqkeretpprf
IPI00010740	472	476	unsp->unsp+cdk5	unsp->unsp+cdk5+p38MAPK		6421	SFPQ	knpmyqkereTpprfaqhgtf
IPI00010796	103	99		->EGFR		5034	P4HB	dlaqqygvrgYptikffrngd
IPI00010796	103	101	unsp+PKC->	unsp+PKC->		5034	P4HB	aqqygvrgypTikffrngdta
IPI00010796	444	439	->PKA			5034	P4HB	aneveavkvhSfptlkffpas
IPI00010796	444	449	unsp->	unsp->		5034	P4HB	sfptlkffpaSadrtvidyng
IPI00010833	607	606	->PKA			7707	ZNF148	sdkanmlqeySkflqqaldrt
IPI00010847	122	124	unsp->	unsp->		1411	CRYBA1	rpicsanhkeSkmtifekenf
IPI00010847	125	124	unsp->	unsp->ATM+unsp+DNAPK		1411	CRYBA1	rpicsanhkeSkmtifekenf
IPI00010863	57	61	PKC+PKA->PKA			475	ATOX1	llatlkktgkTvsylgle---
IPI00010863	60	58	PKC->	PKC->		475	ATOX1	mdtllatlkkTgktvsylgle
IPI00010863	60	63	unsp->	unsp->		475	ATOX1	atlkktgktvSylgle-----
IPI00010872	149	147	unsp+PKG->PKG	unsp+PKG->PKG		8535	CBX4	ragkppppgkSgkyyyqlnsk
IPI00010896	13	16	unsp+PKC+cdc2->cdc2	unsp+PKC+cdc2->PKC+cdc2		1192	CLIC1	pqvelfvkagSdgakigncpf
IPI00010896	49	54	unsp+PKC+RSK->unsp+PKC+CaM-II+RSK	unsp+PKC+RSK->unsp+RSK		1192	CLIC1	ttvdtkrrteTvqklcpggql
IPI00010896	119	115	PKG->PKG+PKA			1192	CLIC1	tagldifakfSayiknsnpal
IPI00010896	119	117		->unsp		1192	CLIC1	gldifakfsaYiknsnpalnd
IPI00011051	236	241	PKG->			3195	TLX1	raalakalkmTdaqvktwfqn
IPI00011062	55	54		->DNAPK		1373	CPS1	qtahivledgTkmkgysfghp
IPI00011062	119	110	->cdc2			1373	CPS1	iignggapdtTaldelglsky
IPI00011062	119	118	CKII->PKA	CKII->ATM+CKII+DNAPK		1373	CPS1	dttaldelglSkylesngikv
IPI00011062	119	123	PKC->	PKC->		1373	CPS1	delglskyleSngikvsgllv
IPI00011062	841	847	unsp->unsp+cdc2	unsp->unsp+cdc2		1373	CPS1	ldlrkelsepSstriyaiaka
IPI00011062	892	890	unsp->	unsp->		1373	CPS1	kmrdilnmekTlkglnsesmt
IPI00011062	892	896	unsp+CKII->CKI+CKII	unsp+CKII->unsp+CKI+CKII		1373	CPS1	nmektlkglnSesmteetlkr
IPI00011069	5	9	PKC+cdc2->cdc2			7374	UNG	--migqktlySffspsparkr
IPI00011069	295	294		unsp+PKA->unsp+PKA+DNAPK		7374	UNG	yrgffgcrhfSktnellqksg
IPI00011107	80	81	unsp->	unsp->		3418	IDH2	ilphvdiqlkYfdlglpnrdq
IPI00011107	133	134	unsp->unsp+p38MAPK	unsp->		3418	IDH2	eefklkkmwkSpngtirnilg
IPI00011107	133	138	PKC->	PKC->		3418	IDH2	lkkmwkspngTirnilggtvf
IPI00011107	155	146	PKC->PKC+cdc2			3418	IDH2	ngtirnilggTvfrepiickn
IPI00011107	180	179	INSR+unsp->unsp			3418	IDH2	tigrhahgdqYkatdfvadra
IPI00011107	180	182	->PKC			3418	IDH2	rhahgdqykaTdfvadragtf
IPI00011107	256	253	PKC->	PKC->		3418	IDH2	kwplymstknTilkaydgrfk
IPI00011107	282	285	INSR->	INSR->		3418	IDH2	ktdfdknkiwYehrliddmva
IPI00011107	413	412	unsp+CKII->unsp	unsp+CKII->unsp+CKII+DNAPK		3418	IDH2	cvetvesgamTkdlagcihgl
IPI00011107	442	440	unsp+PKC->	unsp+PKC->		3418	IDH2	hflnttdfldTiksnldralg
IPI00011201	94	97	EGFR->	EGFR->		4200	ME2	giqerneklfYrilqddiesl
IPI00011201	156	161	PKG+CKII->CKII			4200	ME2	penhvkavvvTdgerilglgd
IPI00011201	224	222		->unsp		4200	ME2	llkdpfymglYqkrdrtqqyd
IPI00011201	224	228		PKG+PKA+DNAPK->PKG+DNAPK		4200	ME2	ymglyqkrdrTqqyddlidef
IPI00011201	346	347		->INSR		4200	ME2	aqkkiwmfdkYgllvkgrkak
IPI00011268	165	160	unsp+PKG->unsp+PKG+PKA			22913	RALY	vtvplvrrvkTnvpvklfars
IPI00011268	165	170	PKA->	PKA->		22913	RALY	tnvpvklfarStavttssaki
IPI00011285	84	78	CKI->	CKI->	PHOsphoELM	823	CAPN1	lgykdlgpnsSktygikwkrp
IPI00011285	84	80	PKC->	PKC->	PHOsphoELM	823	CAPN1	ykdlgpnsskTygikwkrpte
IPI00011416	95	97	PKA->	PKA->		1891	ECH1	remvecfnkiSrdadcravvi
IPI00011416	318	315	->CKII	->CKII		1891	ECH1	qdlvksvqatTenkelktvtf
IPI00011416	327	326	PKC->PKA	PKC->ATM+DNAPK		1891	ECH1	enkelktvtfSkl--------
IPI00011518	126	129	unsp->	unsp->		23621	BACE1	styrdlrkgvYvpytqgkweg
IPI00011518	126	132	INSR->	INSR->		23621	BACE1	rdlrkgvyvpYtqgkwegelg
IPI00011518	307	315	PKC->PKC+cdc2			23621	BACE1	avksikaassTekfpdgfwlg
IPI00011568	136	143	->cdc2			6736	SRY	rkakmlpkncSllpadpasvl
IPI00011857	494	493		->ATM+DNAPK		8208	CHAF1B	rvtlntlqawSkttprrinlt
IPI00011857	494	496		unsp+PKC+p38MAPK->unsp+p38MAPK		8208	CHAF1B	lntlqawsktTprrinltplk
IPI00011875	83	79	unsp+PKC->unsp+PKC+PKA	unsp+PKC->unsp+PKC+PKA		3550	IK	dpaarrrkkkSyyakIrqqei
IPI00011875	98	99	unsp->	unsp->		3550	IK	iererelaekYrdrakerrdg
IPI00011875	386	382	unsp+PKC+PKA->unsp+PKA+RSK	unsp+PKC+PKA->unsp+PKA		3550	IK	rereeekkrhSyfekpkvdde
IPI00011875	408	409	unsp+CKII->CKII	unsp+CKII->CKII		3550	IK	gpgstkelikSinekfagsag
IPI00011923	250	255	unsp+CKII->CKII	unsp+CKII->CKII		145258	GSC	reeegksdldSds--------
IPI00011970	321	326	unsp+PKC+PKA->unsp+PKC			51478	HSD17B7	llelekhirvTiqktdnqarl
IPI00012007	188	183	unsp+PKC->unsp			191	AHCY	lkvpainvndSvtkskfdnly
IPI00012007	188	185	PKC->			191	AHCY	vpainvndsvTkskfdnlygc
IPI00012007	188	187		unsp+PKC->ATM+unsp		191	AHCY	ainvndsvtkSkfdnlygcre
IPI00012007	188	193	INSR->SRC	INSR->	PhosphoSite	191	AHCY	svtkskfdnlYgcreslidgi
IPI00012007	408	407	PKC->	PKC->		191	AHCY	ahlgklnvklTkltekqaqyl
IPI00012007	408	410	unsp+PKC+PKG->unsp+PKC	unsp+PKC+PKG->unsp+PKC		191	AHCY	gklnvkltklTekqaqylgms
IPI00012011	19	23		unsp+PKA->unsp		1072	CFL1	kvfndmkvrkSstpeevkkrk
IPI00012011	19	25	unsp+p38MAPK->unsp		PHOsphoELM	1072	CFL1	fndmkvrkssTpeevkkrkka
IPI00012011	92	89		unsp->INSR+unsp	PhosphoSite	1072	CFL1	dcryalydatYetkeskkedl
IPI00012011	92	91		CKI+PKC->CKI+DNAPK		1072	CFL1	ryalydatyeTkeskkedlvf
IPI00012011	92	94	unsp+PKC+PKG+CKII->unsp+PKC+CKII	unsp+PKC+PKG+CKII->unsp+CKII		1072	CFL1	lydatyetkeSkkedlvfifw
IPI00012011	95	91	CKI+PKC->CKI	CKI+PKC->CKI		1072	CFL1	ryalydatyeTkeskkedlvf
IPI00012011	95	94	unsp+PKC+PKG+CKII->unsp+PKA	unsp+PKC+PKG+CKII->ATM+unsp+CKII		1072	CFL1	lydatyetkeSkkedlvfifw
IPI00012011	114	113	PKG->PKA	PKG->ATM		1072	CFL1	fwapesaplkSkmiyasskda
IPI00012011	114	117	unsp->		PhosphoSite	1072	CFL1	esaplkskmiYasskdaikkk
IPI00012011	121	113	PKG->PKG+PKA			1072	CFL1	fwapesaplkSkmiyasskda
IPI00012011	121	119	unsp+PKC->unsp	unsp+PKC->unsp		1072	CFL1	aplkskmiyaSskdaikkklt
IPI00012011	121	120		PKC->ATM+PKC+DNAPK		1072	CFL1	plkskmiyasSkdaikkkltg
IPI00012011	132	129	PKC->PKC+PKA			1072	CFL1	sskdaikkklTgikhelqanc
IPI00012011	144	140	unsp->EGFR	unsp->unsp+EGFR	PHOsphoELM|PhosphoSite	1072	CFL1	gikhelqancYeevkdrctla
IPI00012011	144	148	unsp+PKC+PKA->PKA	unsp+PKC+PKA->PKC		1072	CFL1	ncyeevkdrcTlaeklggsav
IPI00012069	59	57	PKC->	PKC->		1728	NQO1	fnpiisrkdiTgkldkpanfq
IPI00012069	262	266	PKC->			1728	NQO1	vghhlgksipTdnqikark--
IPI00012079	591	590		->ATM+DNAPK		1975	EIF4B	epkkpeenpaSkfssaskyaa
IPI00012079	591	593	PKG->	PKG->		1975	EIF4B	kpeenpaskfSsaskyaalsv
IPI00012079	591	596	unsp+PKC->PKC	unsp+PKC->		1975	EIF4B	enpaskfssaSkyaalsvdge
IPI00012119	138	137	PKC->	PKC->DNAPK		1634	DCN	plvklerlylSknqlkelpek
IPI00012149	609	611	PKG->	PKG->		10199	MPHOSPH1(	kssvdqagkySktvaseklkq
IPI00012174	114	110	unsp+PKC+RSK->unsp+PKA+RSK	unsp+PKC+RSK->unsp+PKA+RSK		6366	CCL21	aqgcrkdrgaSktgkkgkgsk
IPI00012174	114	112	unsp+PKC->PKC	unsp+PKC->PKC		6366	CCL21	gcrkdrgaskTgkkgkgskgc
IPI00012174	115	110	unsp+PKC+RSK->unsp+PKA	unsp+PKC+RSK->unsp+PKC+PKA		6366	CCL21	aqgcrkdrgaSktgkkgkgsk
IPI00012174	115	112	unsp+PKC->PKC	unsp+PKC->PKC		6366	CCL21	gcrkdrgaskTgkkgkgskgc

Table S3

IPI00012174	117	110	unsp+PKC+RSK->unsp+PKC+cdc2+PKA+RSK	unsp+PKC+RSK->unsp+PKC+PKA+RSK		6366	CCL21	aqgcrkdrgaSktgkkgkgsk
IPI00012174	120	119		unsp+PKC+PKA->ATM+unsp+PKC+PKA+DNAPK		6366	CCL21	asktgkkgkgSkgckrtersq
IPI00012199	212	218	unsp->unsp+PKA			79080	CCDC86	gfgakkrkgsSsqapaskkln
IPI00012199	213	217	unsp+PKC+PKG+PKA+RSK->unsp+PKG+PKA+RSK	unsp+PKC+PKG+PKA+RSK->unsp+PKC+PKG+cdc2+PKA+RSK		79080	CCDC86	ggfgakkrkgSssqapaskkl
IPI00012199	213	218	unsp->	unsp->		79080	CCDC86	gfgakkrkgsSsqapaskkln
IPI00012341	167	165	unsp+PKC+CKII->CKII	unsp+PKC+CKII->unsp+CKII		6430	SFRS5	gdlknaieklSgkeingrkik
IPI00012345	101	97	unsp+PKC+PKA+RSK->unsp+PKA+RSK	unsp+PKC+PKA+RSK->unsp+PKA+RSK		6431	SFRS6	ggggyssrrtSgrdkygppvr
IPI00012345	101	102	INSR->			6431	SFRS6	ssrrtsgrdkYgppvrteyrl
IPI00012382	60	64	unsp->			6626	SNRPA	qafvifkevsSatnalrsmqg
IPI00012442	353	354	->CKII	->CKII		10146	G3BP1	ignlphevdkSelkdffqsyg
IPI00012442	357	354	->CKII	->CKII		10146	G3BP1	ignlphevdkSelkdffqsyg
IPI00012442	357	362	PKA->cdc2	PKA->cdc2+PKA		10146	G3BP1	dkselkdffqSygnvvelrin
IPI00012442	376	373	unsp+PKA->PKA		Uniprot|PhosphoSite	10146	G3BP1	ygnvvelrinSggklpnfgfv
IPI00012446	34	29	PKC->	PKC->		2516	NR5A1	kvsgyhygllTcesckgffkr
IPI00012446	34	32	PKC->	PKC->		2516	NR5A1	gyhygllitceSckgffkrtvq
IPI00012446	38	40		PKC->		2516	NR5A1	cesckgffkrTvqnnkhytct
IPI00012567	327	323	unsp+PKC->unsp+PKC+RSK			7422	VEGFA	kkdrarqekkSvrgkgkgqkr
IPI00012567	332	340	unsp->INSR+unsp			7422	VEGFA	gqkrkrkksrYkswsvyvgar
IPI00012587	402	401		->ATM		5728	PTEN	pfdedqhtqiTkv--------
IPI00012726	104	96	unsp->unsp+PKA		Uniprot|PhosphoSite	8761	PABPC4	wsqrdpslrkSgvgnvfiknl
IPI00012726	104	109	unsp->	unsp->		8761	PABPC4	gnvfiknldkSidnkalydtf
IPI00012750	52	54		PKC->		6230	RPS25	lnnlvlfdkaTydklckevpn
IPI00012750	52	55	unsp->	unsp->		6230	RPS25	nnlvlfdkatYdklckevpny
IPI00012750	66	65	unsp->	unsp->		6230	RPS25	ydklckevpnYklitpavvse
IPI00012750	66	69	p38MAPK->			6230	RPS25	ckevpnykliTpavvserlki
IPI00012750	94	93	PKC->	PKC->ATM+DNAPK		6230	RPS25	laraalqellSkgliklvskh
IPI00012750	94	101	PKC->PKC+cdc2	PKC->PKC+cdc2		6230	RPS25	llskgliklvSkhraqviytr
IPI00012788	176	174	unsp+PKC->PKC	unsp+PKC->PKC	niprot|PHOsphoELM|PhosphoSi	10849	CD3EAP	rsalapnlltSgkkkkemqvt
IPI00012795	264	259		unsp+PKC->PKC		8668	EIF3I	gqeamdvtttStrigkfearf
IPI00012795	264	260	PKC->	PKC->		8668	EIF3I	qeamdvtttsTrigkfearff
IPI00012816	265	264		unsp+PKC->unsp+PKC+DNAPK		10661	KLF1	dpgviaetapSkrgrrswark
IPI00012816	265	270	unsp+PKC+PKA->unsp+PKC			10661	KLF1	etapskrgrrSwarkrqaaht
IPI00012816	274	270	unsp+PKC+PKA->unsp+PKA			10661	KLF1	etapskrgrrSwarkrqaaht
IPI00012816	288	290	INSR->	INSR->		10661	KLF1	tcahpgcgksYtksshlkahl
IPI00012816	288	291	PKC->			10661	KLF1	cahpgcgksyTksshlkahlr
IPI00012816	288	293	PKC->	PKC->		10661	KLF1	hpgcgksytkSshlkahlrth
IPI00012816	288	294	unsp+PKC->unsp+PKC+cdc2	unsp+PKC->unsp+PKC+cdc2		10661	KLF1	pgcgksytksShlkahlrtht
IPI00012837	166	164		->unsp		3799	KIF5B	svhedknrvpYvkgcterfvc
IPI00012912	510	509	unsp+PKC->	unsp+PKC->ATM		1376	CPT2	tetirpasvyTkrcseafvre
IPI00012912	510	513	unsp->	unsp->		1376	CPT2	rpasvytkrcSeafvrepsrh
IPI00012912	537	536		->DNAPK		1376	CPT2	gelqqmmvecSkyhgqltkea
IPI00012912	544	543	unsp+PKC->unsp	unsp+PKC->ATM+unsp+DNAPK		1376	CPT2	vecskyhgqlTkeaamgqgfd
IPI00012972	91	94	EGFR->	EGFR->		4833	NME4	hyqdlrrkpfYpalirymssg
IPI00013070	270	268	unsp->	unsp->		11100	HNRNPUL1	cfemkineeiSvkhlpstepd
IPI00013070	270	275	PKC+cdc2->cdc2	PKC+cdc2->cdc2		11100	HNRNPUL1	eeisvkhlpsTepdphvvrig
IPI00013122	240	248	->INSR	->INSR		11140	CDC37	ftkiktadrqYmegfndelea
IPI00013122	330	322	unsp+CKI->unsp+CKI+cdc2			11140	CDC37	kdvqmlqdaiSkmdptdakyh
IPI00013122	330	327	PKC->	PKC->		11140	CDC37	lqdaiskmdpTdakyhmqrci
IPI00013159	11	2	->cdc2			2120	ETV6	---------mSetpaqcsikq
IPI00013159	11	9	unsp+PKC->	unsp+PKC->		2120	ETV6	--msetpaqcSikqerisytp
IPI00013159	11	16	unsp+PKA->unsp+cdc2	unsp+PKA->unsp+cdc2	niprot|PHOsphoELM|PhosphoSi	2120	ETV6	aqcsikqeriSytppespvps
IPI00013159	302	307	unsp+PKC->unsp+PKC+cdc2	unsp+PKC->unsp+cdc2	PhosphoSite	2120	ETV6	lhregkpinlShredlaymnh
IPI00013160	337	336	->PKA	->ATM+DNAPK		1939	LGTN	qeqiiqvkelSkgvesivavd
IPI00013184	136	137	unsp->SRC+unsp	unsp->SRC+unsp	PhosphoSite	8260	NAA10	nfqisevepkYyadgedayam
IPI00013184	136	138	INSR+unsp->unsp		PhosphoSite	8260	NAA10	fqisevepkyYadgedayamk
IPI00013185	205	200	unsp+PKC->unsp			8242	KDM5C	ykphsiplrqSvqpskfnsyg
IPI00013185	205	204	->PKA	->ATM		8242	KDM5C	siplrqsvqpSkfnsygrrak
IPI00013185	205	208	unsp->	unsp->		8242	KDM5C	rqsvqpskfnSygrrakrlqp
IPI00013205	602	598	PKC->	PKC->		23030	KDM4B	karagegqapStfsklkmeik
IPI00013205	602	599	PKC->			23030	KDM4B	aragegqapsTfsklkmeikk
IPI00013205	602	601		PKC->ATM+PKC+DNAPK		23030	KDM4B	agegqapstfSklkmeikksr
IPI00013205	602	610	unsp+PKC+PKA->unsp+PKC	unsp+PKC+PKA->unsp+PKC		23030	KDM4B	fsklkmeikkSrrhplgrppt
IPI00013215	326	325		PKA->ATM+DNAPK		4998	ORC1L	riilrtriaaSktidireert
IPI00013296	8	2	->PKA			645958	RPS18P9	---------mSlvipekfqhi
IPI00013296	94	95	INSR+unsp->unsp			645958	RPS18P9	nrqkdvkdgkYsqvlangldn
IPI00013396	52	48	unsp+CKI+PKC->unsp+CKI+PKA	unsp+CKI+PKC->unsp+CKI+PKA		6631	SNRPC	yqkwmeeqaqSlidkttaafq
IPI00013396	52	54	->PKC			6631	SNRPC	eqaqslidktTaafqqgkipp
IPI00013404	45	42	PKC->			4089	SMAD4	setfakraieSlvkklkekkd
IPI00013404	428	430	INSR->	INSR->		4089	SMAD4	rapgdavhkiYpsayikvfdl
IPI00013404	428	434	INSR+EGFR->EGFR	INSR+EGFR->EGFR		4089	SMAD4	davhkiypsaYikvfdlrqch
IPI00013404	507	504	cdc2->PKA+cdc2	cdc2->PKA+cdc2		4089	SMAD4	dlrrlcilrmSfvkgwgpdyp
IPI00013415	74	71	PKC->			6201	RPS7	iifvpvpqlkSfqkiqvrlvr
IPI00013452	542	547	unsp->	unsp->		2058	EPRS	pevglkpvwySpkvfiegada
IPI00013452	788	786	unsp+PKC->	unsp+PKC->		2058	EPRS	dvdaavkqllSlkaeykektg
IPI00013468	179	176	unsp+PKC+PKG+cdc2+PKA+RSK->unsp+PKG+PKA+RSK	unsp+PKC+PKG+cdc2+PKA+RSK->unsp+PKG+PKA+RSK		9184	BUB3	nmgyvqqrreSslkyqtrcir
IPI00013468	179	177	unsp+PKC+PKA->PKA+RSK	unsp+PKC+PKA->PKA+DNAPK+RSK		9184	BUB3	mgyvqqrresSlkyqtrcira
IPI00013468	179	180	unsp->	unsp->		9184	BUB3	vqqrresslkYqtrcirafpn
IPI00013485	263	259	PKC->	PKC->		6187	RPS2	syltpdlwkeTvftkspyqef
IPI00013485	263	262	PKC+PKG->	PKC+PKG->DNAPK		6187	RPS2	tpdlwketvfTkspyqeftdh
IPI00013485	263	264	unsp->unsp+p38MAPK		Uniprot|PHOsphoELM|PhosphoSi	6187	RPS2	dlwketvftkSpyqeftdhlv
IPI00013485	263	266	unsp+EGFR->	unsp+EGFR->	PhosphoSite	6187	RPS2	wketvftkspYqeftdhlvkt
IPI00013485	275	276		PKC->unsp+PKC		6187	RPS2	yqeftdhlvkThtrvsvqrtq
IPI00013508	492	490	unsp+PKC->	unsp+PKC->DNAPK		87	ACTN1	cdqwdnlgalTqkrrealert
IPI00013683	58	56	unsp+PKC->	unsp+PKC->		10381	TUBB3	risvyyneasShkyvprailv
IPI00013721	358	354	unsp+PKC+PKA->unsp+PKA	unsp+PKC+PKA->unsp+PKA	niprot|PHOsphoELM|PhosphoSi	8899	PRPF4B	rrpgrspkrrSlspkprdksr
IPI00013721	358	356	unsp+cdc2+p38MAPK->unsp+cdc2+RSK+p38MAPK	unsp+cdc2+p38MAPK->unsp+GSK3+cdk5+cdc2+RSK	niprot|PHOsphoELM|PhosphoSi	8899	PRPF4B	pgrspkrrslSpkprdksrrs
IPI00013721	358	363	unsp+PKC+PKA->unsp+PKC	unsp+PKC+PKA->unsp+PKC		8899	PRPF4B	rslspkprdkSrrsrsplInd
IPI00013721	358	366	unsp+PKA->unsp+cdc2+PKA		Uniprot|PHOsphoELM|PhosphoSi	8899	PRPF4B	spkprdksrrSrsplIndrrs
IPI00013723	46	41		unsp+CKI->unsp		5300	PIN1	asqwerpsgnSssggkngqge
IPI00013723	46	43	PKC+cdc2->	PKC+cdc2->PKC		5300	PIN1	qwerpsgnssSggkngqgepa
IPI00013774	74	67		INSR+unsp->unsp		3065	HDAC1	hkanaeemtkYhsddyikflr
IPI00013774	74	78	unsp+PKC->			3065	HDAC1	hsddyikflrSirpdnmseys
IPI00013774	89	88		->DNAPK		3065	HDAC1	sirpdnmseySkqmqrfnvge
IPI00013774	218	221	unsp->	unsp->	Uniprot|PhosphoSite	3065	HDAC1	lrdigagkgkYyavnyplrdg
IPI00013774	220	221	unsp->		Uniprot|PhosphoSite	3065	HDAC1	lrdigagkgkYyavnyplrdg
IPI00013774	220	222	->unsp	->unsp		3065	HDAC1	rdigagkgkyYavnyplrdgi
IPI00013774	432	434	unsp+PKG+cdc2+PKA+RSK->PKC+cdc2+RSK	unsp+PKG+cdc2+PKA+RSK->cdc2+RSK		3065	HDAC1	eeegeggrknSsnfkkakrvk
IPI00013774	432	435	PKC->cdc2	PKC->PKC+cdc2		3065	HDAC1	eegeggrknsSnfkkakrvkt
IPI00013774	438	435	PKC->	PKC->		3065	HDAC1	eegeggrknsSnfkkakrvkt
IPI00013774	439	434	unsp+PKG+cdc2+PKA+RSK->PKG+cdc2+PKA+RSK	unsp+PKG+cdc2+PKA+RSK->PKG+cdc2+PKA+RSK		3065	HDAC1	eeegeggrknSsnfkkakrvk
IPI00013774	439	435	PKC->	PKC->		3065	HDAC1	eegeggrknsSnfkkakrvkt
IPI00013774	441	435		PKC->PKC+cdc2		3065	HDAC1	eegeggrknsSnfkkakrvkt
IPI00013774	441	445		unsp+PKG+CKII+RSK->unsp+CKII+RSK		3065	HDAC1	snfkkakrvkTedekekdpee
IPI00013808	625	621		->unsp+PKG	PhosphoSite	81	ACTN4	yttvtpqiinSkwekvqqlvp
IPI00013830	115	113	unsp->			22938	SNW1	rqgqskdkviYskytdlvpke
IPI00013830	115	114	->PKA+DNAPK	->ATM+DNAPK		22938	SNW1	qgqskdkviySkytdlvpkev
IPI00013830	115	117		PKC->		22938	SNW1	skdkviyskyTdlvpkevmna
IPI00013847	111	107	unsp+PKA->unsp+PKA+RSK	unsp+PKA->unsp+PKG+PKA		7384	UQCRC1	afkgtknrpgSalekevesmg
IPI00013847	138	132		INSR->		7384	UQCRC1	aystrehtayYikalskdlpk
IPI00013847	138	137	PKC+cdc2->PKC+PKA+cdc2	PKC+cdc2->unsp+ATM+cdc2+DNAPK		7384	UQCRC1	ehtayyikalSkdlpkavell
IPI00013862	169	171	PKG->	PKG->		1841	DTYMK	lrcfhqlmkdTtlnwkmvdas
IPI00013871	17	20	PKC->PKC+cdc2	PKC->PKC+cdc2		6240	RRM1	qervmfdkitSriqklcygln
IPI00013871	376	373	unsp+PKC+cdc2->unsp+cdc2	unsp+PKC+cdc2->unsp		6240	RRM1	wgeefeklyaSyekqgrvrkv
IPI00013871	496	494	unsp+PKC->	unsp+PKC->		6240	RRM1	nyypvpeaclSnkrhrpigig
IPI00013894	8	16	unsp->unsp+cdc2		Uniprot|PhosphoSite	10963	STIP1	elkekgnkalSvgniddalqc
IPI00013894	68	64	PKC+CKII->CKII	PKC+CKII->CKII		10963	STIP1	yqkayedgckTvdlkpdwgkg
IPI00013894	73	76	PKC+cdc2->cdc2			10963	STIP1	dlkpdwgkgySrkaaalefln
IPI00013894	312	309	PKC->PKA			10963	STIP1	iakayarignSyfkeekykda
IPI00013894	312	310	INSR->			10963	STIP1	akayarignsYfkeekykdai
IPI00013894	325	326	PKA->PKA+DNAPK	PKA->PKA+DNAPK		10963	STIP1	ykdaihfynkSlaehrtpdvl
IPI00013894	446	444	INSR+EGFR->	INSR+EGFR->unsp+INSR+EGFR		10963	STIP1	kaaaleamkdYtkamdvyqka
IPI00013894	446	445		->DNAPK		10963	STIP1	aaaleamkdyTkamdvyqkal
IPI00013894	446	451	unsp->unsp+EGFR			10963	STIP1	mkdytkamdvYqkaldldssc
IPI00013895	3	5	PKA->	PKA->	PhosphoSite	6282	S100A11	------makiSsptetercie
IPI00013895	3	6	unsp->unsp+CKII	unsp->unsp+CKII	niprot|PHOsphoELM|PhosphoSi	6282	S100A11	-----makisSptetercies
IPI00013914	983	982		unsp+PKC+PKA->unsp+PKA+DNAPK		5896	RAG1	rrfrkmnarqSkcyemedvlk
IPI00013957	148	147		unsp->unsp+DNAPK		788	SLC25A20	llqiqassgeSkytgtldcak
IPI00013957	244	239	unsp+PKG->unsp+PKG+PKA	unsp+PKG->unsp+PKG+PKA		788	SLC25A20	ppdvlksrfqTappgkypngf

IPI00013991	118	123	unsp->unsp+CKI			7169	TPM2	leeaekaadeSergmkvienr
IPI00013999	246	251	unsp+PKC->PKC	unsp+PKC->PKC		2623	GATA1	lirpkkrlivSkragtqctnc
IPI00013999	312	310	unsp+PKC+PKB->unsp+PKC+PKB+RSK	unsp+PKC+PKB->unsp+PKC+PKB+RSK	niprot|PHOsphoELM|PhosphoSi	2623	GATA1	dgiqtrnrkaSgkgkkkrgss
IPI00013999	314	310	unsp+PKC+PKB->unsp+PKC+PKB+RSK	unsp+PKC+PKB->unsp+PKC+PKB+RSK	niprot|PHOsphoELM|PhosphoSi	2623	GATA1	dgiqtrnrkaSgkgkkkrgss
IPI00014068	455	457	cdc2+PKA->cdc2	cdc2+PKA->cdc2		10298	PAK4	llthdgrvklSdfgfcaqvsk
IPI00014068	467	466		unsp->ATM+unsp+DNAPK		10298	PAK4	lsdfgfcaqvSkevprrkslv
IPI00014198	116	117	unsp->unsp+cdc2	unsp->unsp+cdc2		23016	EXOSC7	ntlyrifnnkSsvdlktlcis
IPI00014198	116	118	PKC+PKA->PKC	PKC+PKA->		23016	EXOSC7	tlyrifnnksSvdlktlcisp
IPI00014199	274	277	PKC+cdc2+PKA->cdc2+PKA			9744	ACAP1	vmeghlfkraSnafktwsrrw
IPI00014213	236	229	->cdc2			23395	LARS2	qvdehgcswrSgakveqkylr
IPI00014230	91	87		CKI+CKII->unsp+CKI+CKII		708	C1QBP	dgdkafvdflSdeikeerkiq
IPI00014266	304	309	PKG+PKA->PKA			8019	BRD3	pqhagkkgklSehlrycdsil
IPI00014305	135	138	unsp+PKC->PKC	unsp+PKC->PKC		7705	ZNF146	fvckecgktfSgksnltehek
IPI00014305	140	136	PKC->	PKC->		7705	ZNF146	kpfvckecgkTfsgksnlteh
IPI00014305	140	138	unsp+PKC->PKC	unsp+PKC->		7705	ZNF146	fvckecgktfSgksnltehek
IPI00014344	105	103	PKC->	PKC->		1859	DYRK1A	lrklsvdlikTykhinevyya
IPI00014361	147	143		unsp->unsp+EGFR		7264	TSTA3	ngpphnsnfgYsyakrmidvq
IPI00014361	147	144	PKC->			7264	TSTA3	gpphnsnfgySyakrmidvqn
IPI00014424	179	175	->unsp	->unsp		1917	EEF1A2	krydeivkevSayikkigynp
IPI00014424	179	183	EGFR->			1917	EEF1A2	evsayikkigYnpatvpfvpi
IPI00014513	174	166	unsp->unsp+cdc2			7528	YY1	agksggggssSsgggrvkkgg
IPI00014575	105	103	unsp+PKC->cdc2	unsp+PKC->		990	CDC6	grrlvfdnqlTikspskrela
IPI00014575	109	103		unsp+PKC->unsp+PKC+cdc2		990	CDC6	grrlvfdnqlTikspskrela
IPI00014575	109	108	unsp->unsp+PKA	unsp->unsp+DNAPK		990	CDC6	fdnqltikspSkrelakvhqn
IPI00014898	187	184	PKC+PKG->			5339	PLEC1	tderdrvqkkTftkwvnkhli
IPI00014898	187	186		PKC->PKC+DNAPK		5339	PLEC1	erdrvqkktfTkwvnkhlika
IPI00014898	2841	2833	->cdc2+PKA			5339	PLEC1	edvrhylqgrSsiaglllkat
IPI00014898	3420	3417	PKC->	PKC->		5339	PLEC1	llrqfrtgkvTvekvikilit
IPI00014903	7	2	->PKA	->PKA		26127	FGFR1OP2	---------mSctiekalada
IPI00014911	405	403	PKC->	PKC->		7226	TRPM2	tftesrivewTkkiqdivrrr
IPI00014911	1104	1101	PKC+GSK3+cdk5+p38MAPK->GSK3+cdk5+p38MAPK	PKC+GSK3+cdk5+p38MAPK->GSK3+cdk5+p38MAPK		7226	TRPM2	qlfikrvvlkTpakrhkqlkn
IPI00014925	376	371	PKC->PKA	PKC->		9401	RECQL4	khyvrgralrSrllrkqawkq
IPI00014925	380	371	PKC->PKC+cdc2			9401	RECQL4	khyvrgralrSrllrkqawkq
IPI00014938	142	138	unsp->unsp+PKA			84324	SARNP	gissvptkglSsdnkpmvnld
IPI00014938	142	139	unsp+PKC->PKA	unsp+PKC->PKC		84324	SARNP	issvptkglsSdnkpmvnldk
IPI00015029	7	5	unsp+PKC->	unsp+PKC->		10728	PTGES3	------mqpaSakwydrrdyv
IPI00015029	33	37	PKC->			10728	PTGES3	vnvnfeksklTfsclggsdnf
IPI00015077	58	54		unsp->INSR+unsp		10209	EIF1	lttvqgiaddYdkkklvkafk
IPI00015105	4	8	unsp->	unsp->		1164	CKS2	---mahkqiyYsdkyfdehye
IPI00015105	4	9	unsp+PKC->PKC			1164	CKS2	--mahkqiyySdkyfdehyey
IPI00015105	4	12	unsp->INSR+unsp	unsp->INSR+unsp		1164	CKS2	ahkqiyysdkYfdehyeyrhv
IPI00015180	1341	1337	unsp+PKC+cdc2->unsp+PKC			357	SHROOM2	dksladildpSvkikttmdlm
IPI00015262	8	3	PKC->			1265	CNN2	--------msStqfnkgpsyg
IPI00015262	8	4	PKC->	PKC->		1265	CNN2	-------mssTqfnkgpsygl
IPI00015262	8	11	unsp->	unsp->		1265	CNN2	msstqfnkgpSyglsaevknr
IPI00015262	8	12	unsp->	unsp->	PhosphoSite	1265	CNN2	sstqfnkgpsYglsaevknrl
IPI00015262	25	24		unsp+PKC+PKA+RSK->ATM+unsp+PKA+DNAPK+RSK		1265	CNN2	lsaevknrllSkydpqkeael
IPI00015361	42	41		unsp->unsp+DNAPK		5204	PFDN5	tsiaqlkvvqTkyveakdcln
IPI00015361	42	43	unsp+EGFR->unsp	unsp+EGFR->unsp		5204	PFDN5	iaqlkvvqtkYveakdclnvl
IPI00015361	112	111		PKC->		5204	PFDN5	dffkrkidflTkqmekiqpal
IPI00015602	185	186	unsp+EGFR->unsp			9868	TOMM70A	ctkavelnpkYvkalfrraka
IPI00015609	61	64	PKC->			54876	DCAF16	wqvkcllkysTtwkplnpnsw
IPI00015609	61	65	unsp+PKC->unsp			54876	DCAF16	qvkcllkystTwkplnpnswl
IPI00015671	401	406	unsp+INSR->unsp	unsp+INSR->unsp		79861	TUBAL3	arldhkfdlmYakraflhwyl
IPI00015671	408	406	unsp+INSR->			79861	TUBAL3	arldhkfdlmYakraflhwyl
IPI00015811	208	210	unsp+PKC+cdc2->PKC+cdc2			5922	RASA2	vgpsrndqkkTkvkkktsnpq
IPI00015811	211	210	unsp+PKC+cdc2->PKC+cdc2	unsp+PKC+cdc2->PKC+cdc2		5922	RASA2	vgpsrndqkkTkvkkktsnpq
IPI00015811	213	210	unsp+PKC+cdc2->PKC+cdc2	unsp+PKC+cdc2->PKC+cdc2		5922	RASA2	vgpsrndqkkTkvkkktsnpq
IPI00015811	213	217	PKC+PKG+PKA->	PKC+PKG+PKA->		5922	RASA2	qkktkvkkktSnpqfneifyf
IPI00015811	214	210	unsp+PKC+cdc2->PKC+cdc2	unsp+PKC+cdc2->PKC+cdc2		5922	RASA2	vgpsrndqkkTkvkkktsnpq
IPI00015811	214	216	PKG->PKC+PKG			5922	RASA2	dqkktkvkkkTsnpqfneify
IPI00015811	214	217	PKC+PKG+PKA->PKG	PKC+PKG+PKA->PKC+PKG		5922	RASA2	qkktkvkkktSnpqfneifyf
IPI00015833	142	141	->EGFR	->EGFR		54927	CHCHD3	drvlkkqdafYkeqlarleer
IPI00015856	41	46	unsp+GSK3+PKA->GSK3	unsp+GSK3+PKA->GSK3+PKG		23549	DNPEP	akellkfvnrSpspfhavaec
IPI00015864	684	689	unsp+PKA+DNAPK->DNAPK	unsp+PKA+DNAPK->DNAPK		6041	RNASEL	kkmklkigdpSlyfqktfpdl
IPI00015865	109	108	unsp->	unsp->EGFR		54936	ADPRHL2	vdmahrfaqeYkkdpdrgyga
IPI00015905	165	160	unsp+PKC->unsp+PKA	unsp+PKC->unsp+PKA		23404	EXOSC2	sdgavslhtrSlkygklgqgv
IPI00015905	165	163	unsp->			23404	EXOSC2	avslhtrslkYgklgqgvlvq
IPI00015911	127	129		PKC->		1738	DLD	nldkmmeqksTavkaltggia
IPI00015911	320	319		PKA->DNAPK		1738	DLD	llvcigrrpfTknlgleelgi
IPI00015911	417	416		->SRC		1738	DLD	eeqlkeegieYkvgkfpfaan
IPI00015911	420	416	->INSR	->INSR		1738	DLD	eeqlkeegieYkvgkfpfaan
IPI00015934	369	363	unsp+RSK->unsp+PKA+RSK	unsp+RSK->unsp+PKA+RSK		688	KLF5	niqpvrynrrSnpdlekrrih
IPI00015934	369	377	->INSR	->INSR	PhosphoSite	688	KLF5	lekrrihycdYpgctkvytks
IPI00015953	779	776	unsp+PKC+PKG->PKG+PKA	unsp+PKC+PKG->unsp+PKC+PKG+PKA		9188	DDX21	nrsqnkgqkrSfskafgq---
IPI00015953	779	778	unsp+PKG+PKA->unsp+PKA+RSK	unsp+PKG+PKA->unsp+PKA+DNAPK		9188	DDX21	sqnkgqkrsfSkafgq-----
IPI00015973	514	510	PKC->PKA	PKC->		2037	EPB41L2	peqppkakflTlgskfrysgr
IPI00015973	514	513		PKC->ATM+PKC+DNAPK		2037	EPB41L2	ppkakfltlgSkfrysgrtqa
IPI00015973	514	518		unsp+PKA->unsp		2037	EPB41L2	fltlgskfrySgrtqaqtrqa
IPI00016284	537	539	unsp+PKC+PKG+PKA->unsp+PKC+PKA	unsp+PKC+PKG+PKA->unsp+PKC+PKA		79893	GGNBP2	kgknkkkkkkSkilkcdehiq
IPI00016284	538	539	unsp+PKC+PKG+PKA->unsp+PKC+PKA	unsp+PKC+PKG+PKA->unsp+PKC+PKA		79893	GGNBP2	kgknkkkkkkSkilkcdehiq
IPI00016287	281	276	unsp+PKA->PKA	unsp+PKA->PKA		79896	THNSL1	fvpakefpklScgewkslvga
IPI00016405	96	93	unsp+PKA->PKA			54940	OCIAD1	cimgyfagklSyvktcqekfk
IPI00016405	96	97	->CKII	->unsp+CKII		54940	OCIAD1	yfagklsyvkTcqekfkklen
IPI00016405	131	134	unsp->			54940	OCIAD1	ppghyyqkskYdssvsgqssf
IPI00016405	131	136	unsp->cdc2	unsp->unsp+cdc2		54940	OCIAD1	ghyyqkskydSsvsgqssfvt
IPI00016457	261	263		->EGFR		1384	CRAT	snhrnswakaYntlikdkvnr
IPI00016458	104	99	PKC->			79944	L2HGDH	qtghnsgvihSgiyykpeslk
IPI00016513	102	101	->PKA	->ATM+DNAPK		10890	RAB10	itngksfeniSkwlrnideha
IPI00016532	27	26	unsp->unsp+PKA			54969	C4orf27	qcekttdvkkSkfceadvssd
IPI00016532	27	35	CKII->cdc2+CKII			54969	C4orf27	kskfceadvsSdlrkevenhy
IPI00016568	186	187	unsp+GSK3->	unsp+GSK3->		205	AK3L1	vakpvielykSrgvlhqfsgt
IPI00016572	16	18	unsp+PKC+PKA->unsp+PKC	unsp+PKC+PKA->unsp+PKC		6637	SNRPG	elkkfmdkklSlklnggrhvq
IPI00016589	23	20	unsp->	unsp->		10869	USP19	prrgppgledTtskkkqkdra
IPI00016589	23	21	unsp+PKC->	unsp+PKC->		10869	USP19	rrgppgledtTskkkqkdran
IPI00016589	23	22		unsp+PKC->unsp+PKC+DNAPK		10869	USP19	rgppgledttSkkkqkdranq
IPI00016610	23	27	unsp+PKC+cdc2->cdc2			5093	PCBP1	rllmhgkevgSiigkkgesvk
IPI00016613	102	106	unsp->			1457	CSNK2A1	tladivkdpvSrtpalvfehv
IPI00016613	102	108	p38MAPK->			1457	CSNK2A1	adivkdpvsrTpalvfehvnn
IPI00016637	174	169	PKC->			54995	OXSM	vvsetalnfqTkgynkvspff
IPI00016637	174	172	EGFR->			54995	OXSM	etalnfqtkgYnkvspffvpk
IPI00016637	174	176	cdk5->cdk5+p38MAPK			54995	OXSM	nfqtkgynkvSpffvpkilvn
IPI00016801	84	79	unsp+PKA+RSK->unsp+PKA	unsp+PKA+RSK->unsp+PKA		2746	GLUD1	mvegffdrgaSivedklvedl
IPI00016801	457	455	unsp+PKC->	unsp+PKC->		2746	GLUD1	nlnhvsygrlTfkyerdsnyh
IPI00016801	503	498	unsp->unsp+PKA	unsp->unsp+PKA		2746	GLUD1	vptaefqdriSgasekdivhs
IPI00016801	503	501	unsp+PKC->unsp	unsp+PKC->unsp		2746	GLUD1	aefqdrisgaSekdivhsgla
IPI00016801	503	508	PKC+PKA->	PKC+PKA->		2746	GLUD1	sgasekdivhSglaytmersa
IPI00016801	527	524	PKC+PKG->PKG	PKC+PKG->PKG		2746	GLUD1	mersarqimrTamkynlgldl
IPI00016801	545	550	->EGFR			2746	GLUD1	vnaiekvfkvYneagvtft--
IPI00016862	401	404		unsp+cdc2->unsp+CKII+cdc2		2936	GSR	ahrlfeykedSkldynniptv
IPI00016862	401	408	unsp+EGFR->INSR+unsp+EGFR			2936	GSR	feykedskldYnniptvvfsh
IPI00016910	558	554		->EGFR		728689	EIF3CL	savlmerlckYiyakdrtdri
IPI00016910	558	556		->unsp		728689	EIF3CL	vlmerlckyiYakdrtdrirt
IPI00017283	189	186	unsp+PKC->unsp		Uniprot|PHOsphoELM|PhosphoSi	55699	IARS2	sameirkkarSfakaaiekqk
IPI00017283	233	239	unsp+PKG->unsp+PKG+cdc2	unsp+PKG->unsp+PKG+cdc2		55699	IARS2	qmydkglvyrSykpvfwspss
IPI00017283	241	239	unsp+PKG->PKG+cdc2	unsp+PKG->PKG		55699	IARS2	qmydkglvyrSykpvfwspss
IPI00017283	661	666	cdc2+PKA->cdc2			55699	IARS2	ftlgekgekmSkslgnvihpd
IPI00017283	664	666	cdc2+PKA->cdc2	cdc2+PKA->cdc2		55699	IARS2	ftlgekgekmSkslgnvihpd
IPI00017283	775	774	unsp->	unsp->		55699	IARS2	qdlankitelYkqydfgkvvr
IPI00017292	19	23	unsp+PKC->PKC		Uniprot|PhosphoSite	1499	CTNNB1	amepdrkaavShwqqqsylds
IPI00017292	49	40	PKC->PKC+cdc2			1499	CTNNB1	yldsgihsgaTttapslsgkg
IPI00017292	49	45	unsp+PKC+CKI->unsp+CKI	unsp+PKC+CKI->unsp+CKI	PHOsphoELM|PhosphoSite	1499	CTNNB1	ihsgatttapSlsgkgnpeee
IPI00017292	49	47	unsp+CKI+PKC->unsp+CKI	unsp+CKI+PKC->unsp+CKI		1499	CTNNB1	sgatttapslSgkgnpeeedv
IPI00017292	345	340	unsp+PKC->unsp	unsp+PKC->unsp		1499	CTNNB1	tytyekllwtTsrvlkvlsvc
IPI00017292	345	341	->PKA			1499	CTNNB1	ytyekllwttSrvlkvlsvcs
IPI00017297	3	2	cdc2->PKA+cdc2+DNAPK	cdc2->cdc2+DNAPK		9782	MATR3	---------mSksfqqsslsr
IPI00017297	3	9	unsp+DNAPK->unsp+cdc2+DNAPK	unsp+DNAPK->unsp+cdc2+DNAPK	PhosphoSite	9782	MATR3	--msksfqqsSlsrdsqghgr
IPI00017297	3	11	unsp->unsp+cdc2			9782	MATR3	msksfqqsslSrdsqghgrdl

IPI00017297	146	150	unsp+PKA+RSK->unsp+PKA	unsp+PKA+RSK->unsp	PhosphoSite	9782	MATR3	qillqlkrrrTeegptlsygr
IPI00017297	473	471	unsp+ATM+PKC->ATM	unsp+ATM+PKC->ATM+DNAPK		9782	MATR3	vfgkpvrvhlSqkykrikkpe
IPI00017297	571	569	unsp+PKC->PKC	unsp+PKC->		9782	MATR3	fqgrcvkvdlSekykklvlri
IPI00017303	555	552	PKC->	PKC->		4436	MSH2	vdiqkngvkfTnskltslnee
IPI00017303	555	554		PKA->ATM+DNAPK		4436	MSH2	iqkngvkftnSkltslneeyt
IPI00017303	555	558	unsp+CKII->CKII	unsp+CKII->CKII		4436	MSH2	gvkftnskltSlneeytknkt
IPI00017305	75	72	->PKA		Uniprot|PhosphoSite	6195	RPS6KA1	fellkvlgqgSfgkvflvrkv
IPI00017344	22	16	unsp->unsp+cdc2	unsp->unsp+cdc2		5869	RAB5B	tarpngqpqaSkicqfklvll
IPI00017381	6	9	->cdc2	->cdc2		5984	RFC4	--mqaflkgtSistkppltkd
IPI00017381	6	11	unsp+PKC->PKC			5984	RFC4	mqaflkgtsiStkppltkdrg
IPI00017381	13	9	->unsp+PKA+CaM-II	->unsp		5984	RFC4	--mqaflkgtSistkppltkd
IPI00017381	13	11	unsp+PKC->cdc2	unsp+PKC->		5984	RFC4	mqaflkgtsiStkppltkdrg
IPI00017381	13	12	PKC->DNAPK	PKC->DNAPK		5984	RFC4	qaflkgtsisTkppltkdrgv
IPI00017381	13	17	unsp->			5984	RFC4	gtsistkpplTkdrgvaasag
IPI00017412	163	162		->DNAPK		5982	RFC2	qalrrtmeiySkttrfalacn
IPI00017412	163	164	PKC->PKC+cdc2	PKC->unsp+PKC+cdc2		5982	RFC2	lrrtmeiyskTtrfalacnas
IPI00017412	163	165		->unsp		5982	RFC2	rrtmeiysktTrfalacnasd
IPI00017412	304	305	->PKC	->PKC		5982	RFC2	iignifrvckTfqmaeylkle
IPI00017448	81	80		->ATM+DNAPK		6227	RPS21	lrlakadgivSknf-------
IPI00017450	407	398	->cdc2			2962	GTF2F1	gtpsaeggstSstlraaaskl
IPI00017450	407	406	unsp+CKI+PKC+cdc2->CKI+cdc2	unsp+CKI+PKC+cdc2->unsp+DNAPK		2962	GTF2F1	stsstlraaaSkleqgkrvse
IPI00017451	55	58	PKC->			10291	SF3A1	evrnivdktaSfvarngpefe
IPI00017469	247	243	unsp+PKC+CKII->unsp+CKII	unsp+PKC+CKII->unsp+CKII		6697	SPR	dckvsaqkllSllekdefksg
IPI00017592	597	591	unsp+CKI+CKII->unsp+CKII	unsp+CKI+CKII->unsp		3954	LETM1	edlqeikkelSktgeekyvee
IPI00017592	597	602	unsp->	unsp->		3954	LETM1	ktgeekyveeSkaskrltkrv
IPI00017596	60	55	PKC->	PKC->		22919	MAPRE1	cqfmdmlfpgSialkkvkfqa
IPI00017596	66	71		unsp+EGFR->unsp		22919	MAPRE1	vkfqakleheYiqnfkilqag
IPI00017617	32	24	->PKA			1655	DDX5	rgfgaprfggSragplsgkkf
IPI00017617	32	30	unsp+PKC->PKC	unsp+PKC->PKC	Uniprot	1655	DDX5	rfggsragplSgkkfgnpgek
IPI00017659	81	83	unsp->		Uniprot	92241	RCSD1	shppkfkvksSpliekIqanl
IPI00017704	102	94	unsp+PKC+RSK->unsp+PKC			23406	COTL1	envsglqrakTgtdktlvkev
IPI00017704	102	99	unsp->	unsp->		23406	COTL1	lqraktgtdkTlvkevvqnfa
IPI00017704	110	115	unsp+CKII->CKII	unsp+CKII->CKII	niprot|PHOsphoELM|PhosphoSi	23406	COTL1	vqnfakefviSdrkeleedfi
IPI00017704	126	127	->CKII	->CKII		23406	COTL1	rkeleedfikSelkkaggany
IPI00017855	520	513		INSR+unsp->unsp	PhosphoSite	50	ACO2	gtlkfnpetdYltgtdgkkfr
IPI00017855	520	515		CKI->		50	ACO2	lkfnpetdylTgtdgkkfrle
IPI00018027	664	662	unsp+PKC->	unsp+PKC->		55109	AGGF1	qlrrthaglgTgkpssfedvh
IPI00018120	175	171	PKC->RSK	PKC->unsp		7818	DAP3	wvkncrdllqSsynkqrfdqp
IPI00018120	175	172	unsp+cdc2->	unsp+cdc2->unsp		7818	DAP3	vkncrdllqsSynkqrfdqpl
IPI00018140	363	359	ATM+CKI+cdc2->ATM+CKI	ATM+CKI+cdc2->unsp+CKI+cdc2		10492	SYNCRIP	vteeilekafSqfgklervkk
IPI00018146	49	45	PKC->PKA			10971	YWHAQ	elsneernllSvayknvvggr
IPI00018146	68	63	unsp+PKG+cdc2+RSK->unsp+PKG+PKA+RSK	unsp+PKG+cdc2+RSK->unsp+PKG+PKA+cdc2+RSK		10971	YWHAQ	ggrrsawrviSsieqktdtsd
IPI00018146	68	64	PKC->			10971	YWHAQ	grrsawrvisSieqktdtsdk
IPI00018146	68	71	unsp+PKC->unsp+PKC+cdc2	unsp+PKC->unsp+PKC+cdc2		10971	YWHAQ	vissieqktdTsdkklqlikd
IPI00018146	68	72	unsp+PKC->unsp			10971	YWHAQ	issieqktdtSdkklqlikdy
IPI00018146	115	110	PKC->			10971	YWHAQ	lldkylianaTnpeskvfylk
IPI00018146	115	114		->ATM+DNAPK		10971	YWHAQ	ylianatnpeSkvfylkmkgd
IPI00018146	115	118	unsp->	unsp->		10971	YWHAQ	natnpeskvfYlkmkgdyfry
IPI00018146	120	118	unsp->			10971	YWHAQ	natnpeskvfYlkmkgdyfry
IPI00018206	150	152	PKG->PKC	PKG->		2806	GOT2	asflqrffkfSrdvflpkptw
IPI00018206	296	292	PKC->	PKC->		2806	GOT2	glygervgafTmvckdadeak
IPI00018206	363	360	PKC+cdc2->	PKC+cdc2->PKC		2806	GOT2	riigmrtqlvSnlkkegsthn
IPI00018206	363	367	PKA->	PKA->		2806	GOT2	qlvsnlkkegSthnwqhitdq
IPI00018206	396	399	unsp->cdc2	unsp->cdc2		2806	GOT2	eqverlikefSiymtkdgris
IPI00018206	404	399		unsp->		2806	GOT2	eqverlikefSiymtkdgris
IPI00018206	404	401		unsp->EGFR	Uniprot|PhosphoSite	2806	GOT2	verlikefsiYmtkdgrisva
IPI00018206	404	403	PKC->	PKC->DNAPK		2806	GOT2	rlikefsiymTkdgrisvagv
IPI00018206	404	409	unsp+PKA->PKA	unsp+PKA->PKA		2806	GOT2	siymtkdgriSvagvtssnvg
IPI00018214	40	42	unsp+cdc2->PKC+cdc2			4149	MAX	erkrrdhikdSfhslrdsvps
IPI00018214	40	45	unsp+PKC->unsp	unsp+PKC->unsp		4149	MAX	rrdhikdsfhSlrdsvpslqg
IPI00018214	66	70	unsp+EGFR->EGFR		PhosphoSite	4149	MAX	raqildkateYiqymrrknht
IPI00018214	153	144	unsp+CKI+CKII->unsp+CKI+cdc2+CKII			4149	MAX	fdggsdssseSepeepqsrkk
IPI00018251	31	29	unsp->unsp+cdc2	unsp->unsp+GSK3	Uniprot	10499	NCOA2	rkecpdqlgpSpkrntekrnr
IPI00018251	636	635	unsp+PKC->unsp	unsp+PKC->ATM+unsp		10499	NCOA2	radgqsrlhdSkgqtkllqll
IPI00018251	636	639	PKC->			10499	NCOA2	qsrlhdskgqTkllqllttks
IPI00018251	640	635	unsp+PKC->unsp	unsp+PKC->unsp		10499	NCOA2	radgqsrlhdSkgqtkllqll
IPI00018251	640	639	PKC->PKA	PKC->		10499	NCOA2	qsrlhdskgqTkllqllttks
IPI00018251	640	647	->cdc2			10499	NCOA2	gqtkllqlltTksdqmepspl
IPI00018251	780	777	unsp->	unsp->		10499	NCOA2	erldsktdpaSntkliamkte
IPI00018251	780	779		->DNAPK		10499	NCOA2	ldsktdpasnTkliamkteke
IPI00018251	788	786	unsp+PKC+CKII->unsp+CKII	unsp+PKC+CKII->unsp+CKII		10499	NCOA2	asntkliamkTekeemsfepg
IPI00018251	788	792	unsp+CKI->unsp+CKI+CKII			10499	NCOA2	iamktekeemSfepgdqpgse
IPI00018278	5	10	unsp+PKC->unsp	unsp+PKC->unsp		94239	H2AFV	-maggkagkdSgkakakavsr
IPI00018278	8	10		unsp+PKC->unsp		94239	H2AFV	-maggkagkdSgkakakavsr
IPI00018278	12	10	unsp+PKC->	unsp+PKC->		94239	H2AFV	-maggkagkdSgkakakavsr
IPI00018278	14	10	unsp+PKC->unsp+PKA	unsp+PKC->unsp		94239	H2AFV	-maggkagkdSgkakakavsr
IPI00018278	14	19	unsp+PKA->	unsp+PKA->PKA		94239	H2AFV	dsgkakakavSrsqraglqfp
IPI00018279	247	241	unsp+GSK3+cdk5+p38MAPK->unsp+GSK3+p38MAPK			50509	COL5A3	atvapqgepeTprprrkgkgk
IPI00018349	220	223	INSR->	INSR->		4173	MCM4	ehiksfdknlYrqlisypqev
IPI00018349	220	228	PKC+PKA->PKC	PKC+PKA->PKC		4173	MCM4	fdknlyrqliSypqeviptfd
IPI00018349	450	453	unsp+cdc2->cdc2	unsp+cdc2->cdc2		4173	MCM4	ekrvellkelSrkpdiyerla
IPI00018349	627	629	PKG->PKC	PKG->unsp		4173	MCM4	piesqwnpkkTtieniqlpht
IPI00018350	392	398	p38MAPK->cdc2+p38MAPK	p38MAPK->cdc2+p38MAPK		4174	MCM5	sqllkfvekcSpigvytsgkg
IPI00018350	396	398	p38MAPK->GSK3+p38MAPK	p38MAPK->GSK3+p38MAPK		4174	MCM5	sqllkfvekcSpigvytsgkg
IPI00018350	396	405	unsp+PKC->unsp+PKC+cdc2	unsp+PKC->unsp+PKC+cdc2		4174	MCM5	ekcspigvytSgkgssaaglt
IPI00018350	696	690	unsp+CKI->unsp			4174	MCM5	krrfaigsqvSehsiikdftk
IPI00018350	696	693	unsp+CKI->			4174	MCM5	faigsqvsehSiikdftkqky
IPI00018350	696	703	unsp->INSR+unsp			4174	MCM5	siikdftkqkYpehaihkvlq
IPI00018352	195	189	CKI+CKII->CKII	CKI+CKII->CKII		7345	UCHL1	mpfpvnhgasSedtllkdaak
IPI00018352	195	192	unsp->			7345	UCHL1	pvnhgassedTllkdaakvcr
IPI00018402	463	468	unsp+cdc2+DNAPK->cdc2+DNAPK	unsp+cdc2+DNAPK->cdc2+DNAPK		6905	TBCE	qkvlekqlpgSmtiqkvkgll
IPI00018452	171	178	->cdc2			8904	CPNE1	gdgkwhlvyrSeviknnlnpt
IPI00018465	172	169	unsp->ATM	unsp->ATM+unsp		10574	CCT7	amtalsskliSqqkaffakmv
IPI00018465	217	219	PKG->PKC	PKG->		10574	CCT7	qlvagvafkkTfsyagfemqp
IPI00018465	217	221	unsp+PKC->			10574	CCT7	vagvafkktfSyagfemqpkk
IPI00018534	6	7	unsp->cdc2	unsp->cdc2		8340	HIST1H2BL	----mpelakSapapkkgskk
IPI00018534	12	7	unsp->	unsp->		8340	HIST1H2BL	----mpelakSapapkkgskk
IPI00018534	12	15	unsp+PKC->PKC	unsp+PKC->PKC	Uniprot	8340	HIST1H2BL	aksapapkkgSkkavtkaqkk
IPI00018534	13	7		unsp->unsp+cdc2		8340	HIST1H2BL	----mpelakSapapkkgskk
IPI00018534	13	15	unsp+PKC->PKC		Uniprot	8340	HIST1H2BL	aksapapkkgSkkavtkaqkk
IPI00018534	16	7	unsp->unsp+cdc2			8340	HIST1H2BL	----mpelakSapapkkgskk
IPI00018534	16	15	unsp+PKC->unsp+PKC+PKA	unsp+PKC->ATM+unsp+PKC+DNAPK	Uniprot	8340	HIST1H2BL	aksapapkkgSkkavtkaqkk
IPI00018534	17	15	unsp+PKC->PKC+PKA	unsp+PKC->PKC+PKA	Uniprot	8340	HIST1H2BL	aksapapkkgSkkavtkaqkk
IPI00018534	21	15	unsp+PKC->unsp+PKC+PKA	unsp+PKC->unsp+PKC+PKA	Uniprot	8340	HIST1H2BL	aksapapkkgSkkavtkaqkk
IPI00018534	21	20		PKC->PKC+DNAPK		8340	HIST1H2BL	apkkgskkavTkaqkkdgkkr
IPI00018534	109	113	unsp->			8340	HIST1H2BL	lpgelakhavSegtkavtkyt
IPI00018534	121	120		->ATM+DNAPK		8340	HIST1H2BL	havsegtkavTkytssk----
IPI00018534	121	123		PKC->		8340	HIST1H2BL	segtkavtkyTssk-------
IPI00018627	140	139	INSR+unsp->unsp			80218	NAA50	feiietkknyYkriepadahv
IPI00018755	82	77	PKC->			1E+08	HMGB1L10	dkahyeremkTyippkgetkk
IPI00018755	82	78	unsp->INSR+unsp	unsp->INSR+unsp+EGFR		1E+08	HMGB1L10	kahyeremktYippkgetkkk
IPI00018755	157	155	unsp->			1E+08	HMGB1L10	ekkaaklkekYekdiaayrak
IPI00018768	187	190	PKC->			7247	TSN	gfrllnlkndSlrkrydglky
IPI00018768	199	200	unsp->	unsp->		7247	TSN	slrkrydglkYdvkkveevvy
IPI00018823	512	509	unsp+cdc2->	unsp+cdc2->unsp		80314	EPC1	ntsetntsdkSfskdlsqilv
IPI00018823	512	511	unsp->	unsp->ATM+unsp+DNAPK		80314	EPC1	setntsdksfSkdlsqilvni
IPI00018842	33	27	unsp->unsp+PKA	unsp->unsp+PKA	Uniprot	9324	HMGN3	kvtkqeptrrSarlsakpapp
IPI00018842	90	93	unsp+PKC+CKII->unsp+CKII		Uniprot|PhosphoSite	9324	HMGN3	tkaeeaqkteSvdnege----
IPI00019148	241	246	PKC->	PKC->		3476	IGBP1	erppvkpfilTrnmaqakvfg
IPI00019226	481	479	unsp->	unsp->		10902	BRD8	kpvplpapemTvkqerldfee
IPI00019353	6	2	PKC->			55750	AGK	---------mTvffktlrnhw
IPI00019353	6	7	unsp+PKC->unsp+CKI+PKC			55750	AGK	----mtvffkTlrnhwkktta
IPI00019380	204	199	CKII->			4686	NCBP1	daemdrifanTesylkrrqkt
IPI00019380	204	202		->unsp		4686	NCBP1	mdrifantesYlkrrqkthvp
IPI00019380	204	209	unsp+PKG+PKA->unsp+CaM-II	unsp+PKG+PKA->unsp+PKG		4686	NCBP1	tesylkrrqkThvpmlqvwta
IPI00019400	58	55	PKC->			7172	TPMT	ghqllkkhldTflkgksglrv
IPI00019450	224	222	unsp->	unsp->		6738	TROVE2	lykekalsveTekllkyleav
IPI00019450	359	360		PKC->unsp+PKC		6738	TROVE2	lkaldaafykTfktveptgkr

IPI00019472	537	539	unsp->	unsp->	PhosphoSite	6510	SLC1A5	gdatvasekeSvm--------
IPI00019502	74	81	unsp+PKG->unsp+PKG+PKA			4627	MYH9	diqkmnppkfSkvedmaeltc
IPI00019502	102	105	INSR->	INSR->		4627	MYH9	asvlhnlkerYysgliytysg
IPI00019502	545	541	unsp+PKG->unsp+PKG+PKA			4627	MYH9	ecwfpkatdkSfvekvmqeqg
IPI00019502	1301	1300	PKC->	PKC->		4627	MYH9	sqsdsksskllTkdfsalesql
IPI00019502	1301	1304	unsp->	unsp->		4627	MYH9	skssklltkdfSalesqlqdtq
IPI00019502	1301	1308	PKC->ATM+PKC			4627	MYH9	kltkdfsaleSqlqdtqellq
IPI00019502	1357	1361	PKC->			4627	MYH9	akhnlekqiaTlhaqvadmkk
IPI00019502	1404	1398		ATM+unsp+CKII->ATM+unsp		4627	MYH9	rklqkdleglSqrheekvaay
IPI00019502	1404	1408	EGFR->		Uniprot|PhosphoSite	4627	MYH9	sqrheekvaaYdklektktrl
IPI00019502	1410	1408	EGFR->		Uniprot|PhosphoSite	4627	MYH9	sqrheekvaaYdklektktrl
IPI00019502	1459	1457	unsp->	unsp->		4627	MYH9	dqllaeektiSakyaeerdra
IPI00019502	1828	1827		->DNAPK		4627	MYH9	aqleeqldneTkerqaackqv
IPI00019548	20	25	unsp+PKC+PKA->PKC	unsp+PKC+PKA->PKC		4087	SMAD2	llgwkksaggSggagggeqng
IPI00019600	72	73	unsp+EGFR->SRC+unsp+EGFR			7336	UBE2V2	yslkvecgpkYpeappsvrfv
IPI00019755	122	125	unsp->GSK3	unsp->		9446	GSTO1	milelfskvpSlvgsfirsqn
IPI00019755	143	139		unsp->unsp+EGFR		9446	GSTO1	sfirsqnkedYaglkeefrke
IPI00019755	160	158	unsp+PKC->	unsp+PKC->		9446	GSTO1	keftkleevlTnkkttffggn
IPI00019812	40	38		->unsp		5536	PPP5C	aeelktqandYfkakdyenai
IPI00019812	40	44	unsp+EGFR->			5536	PPP5C	qandyfkakdYenaikfysqa
IPI00019812	42	38		->EGFR		5536	PPP5C	aeelktqandYfkakdyenai
IPI00019812	42	44	unsp+EGFR->unsp			5536	PPP5C	qandyfkakdYenaikfysqa
IPI00019848	288	293	PKA->	PKA->		3054	HCFC1	vathekewkcTntlaclnldt
IPI00019848	813	808	PKC->			3054	HCFC1	iittkvmtsgTgapakiitav
IPI00019848	2005	2003	unsp+PKC->	unsp+PKC->		3054	HCFC1	patqvrwlqeTskdssgtkpa
IPI00019848	2005	2004		CKI->ATM+CKI+DNAPK		3054	HCFC1	atqvrwlqetSkdssgtkpan
IPI00019848	2005	2007	unsp->CKI	unsp->unsp+CKI	Uniprot	3054	HCFC1	vrwlqetskdSsgtkpankrp
IPI00019868	15	17	unsp+PKA+cdc2->cdc2	unsp+PKA+cdc2->unsp+cdc2		2706	GJB2	qtilggvnkhStsigkiwltv
IPI00019868	15	18	PKC->			2706	GJB2	tilggvnkhsTsigkiwltvl
IPI00019869	27	21	unsp+CKI+PKG+CKII+PKA->unsp+PKG+CKII+PKA	unsp+CKI+PKG+CKII+PKA->unsp+PKG+CKII+PKA		6273	S100A2	avlvttfhkyScqegdkfkls
IPI00019869	27	31		unsp+PKC+PKG+CKII+PKA->unsp+PKC+cdc2+CKII		6273	S100A2	scqegdkfklSkgemkellhk
IPI00019888	126	121	cdc2->PKA+cdc2			7915	ALDH5A1	rwrevsakerSsllrkwynlm
IPI00019888	126	122	unsp+PKC->unsp+PKA	unsp+PKC->unsp+PKA		7915	ALDH5A1	wrevsakersSllrkwynlmi
IPI00019912	139	143	unsp->			3295	HSD17B4	aawehmkkqkYgriimtssas
IPI00019912	140	143	unsp->	unsp->		3295	HSD17B4	aawehmkkqkYgriimtssas
IPI00019912	663	665	->PKC			3295	HSD17B4	tkggnigakwTidlksgsgkv
IPI00019912	669	672	unsp+PKC->PKC	unsp+PKC->PKC		3295	HSD17B4	akwtidlksgSgkvyqgpakg
IPI00019912	707	711	PKC->			3295	HSD17B4	gkldpqkaffSgrlkargnim
IPI00019927	204	203	cdc2->PKA+cdc2	cdc2->cdc2+DNAPK		5713	PSMD7	nqvhglkglnSklldirsyle
IPI00019927	214	217	unsp+PKC->	unsp+PKC->PKC		5713	PSMD7	dirsylekvaTgklpinhqii
IPI00019981	52	48	->PKA			2961	GTF2E2	kkktkvehggSsgskqnsdhs
IPI00019981	52	49	PKC->			2961	GTF2E2	kktkvehggsSgskqnsdhsn
IPI00019981	52	51	unsp+cdc2->unsp+cdc2+DNAPK	unsp+cdc2->ATM+unsp+cdc2+DNAPK		2961	GTF2E2	tkvehggssgSkqnsdhsngs
IPI00019981	52	55	unsp->unsp+cdc2	unsp->unsp+cdc2		2961	GTF2E2	hggssgskqnSdhsngsfnlk
IPI00019992	407	403	PKC->	PKC->		4603	MYBL1	isdaaaspikStpvklmriqh
IPI00019992	407	404	unsp+cdk5+p38MAPK->unsp+p38MAPK			4603	MYBL1	sdaaaspiksTpvklmriqhn
IPI00020008	48	46	PKC->	PKC->		4738	NEDD8	ippqqqrliySgkqmndekta
IPI00020021	326	328	unsp->unsp+PKC			7913	DEK	kpptdeelkeTikkllasanl
IPI00020039	286	280	INSR+unsp->unsp	INSR+unsp->unsp		1538	CYLC1	qnnsknyslkYtkytkkdtkk
IPI00020039	286	281	PKC->			1538	CYLC1	nnsknyslkyTkytkkdtkkn
IPI00020039	286	283		unsp+EGFR->EGFR		1538	CYLC1	sknyslkytkYtkkdtkknak
IPI00020039	286	284	unsp+PKC+PKG->unsp+PKC	unsp+PKC+PKG->unsp+PKC		1538	CYLC1	knyslkytkyTkkdtkknakk
IPI00020042	238	233	PKC->	PKC->		5704	PSMC4	ttaafirvvgSefvqkylgeg
IPI00020042	401	403		unsp->unsp+EGFR		5704	PSMC4	ivlakdfekaYktvikkdeqe
IPI00020042	418	417	unsp->		PhosphoSite	5704	PSMC4	ikkdeqehefYk---------
IPI00020101	6	7		unsp->	Uniprot	8346	HIST1H2BI	----mpepakSapapkkgskk
IPI00020101	12	7	unsp->	unsp->	Uniprot	8346	HIST1H2BI	----mpepakSapapkkgskk
IPI00020101	12	15	unsp+PKC->PKC	unsp+PKC->PKC	Uniprot|PHOsphoELM	8346	HIST1H2BI	aksapapkkgSkkavtkaqkk
IPI00020101	13	15	unsp+PKC->PKC		Uniprot|PHOsphoELM	8346	HIST1H2BI	aksapapkkgSkkavtkaqkk
IPI00020101	16	15	unsp+PKC->unsp+PKC+PKA	unsp+PKC->ATM+unsp+PKC+DNAPK	Uniprot|PHOsphoELM	8346	HIST1H2BI	aksapapkkgSkkavtkaqkk
IPI00020101	17	15	unsp+PKC->PKC+PKA	unsp+PKC->PKC+PKA	Uniprot|PHOsphoELM	8346	HIST1H2BI	aksapapkkgSkkavtkaqkk
IPI00020101	21	15	unsp+PKC->unsp+PKC+PKA	unsp+PKC->unsp+PKC+PKA	Uniprot|PHOsphoELM	8346	HIST1H2BI	aksapapkkgSkkavtkaqkk
IPI00020101	21	20		PKC->PKC+DNAPK		8346	HIST1H2BI	apkkgskkavTkaqkkdgkkr
IPI00020101	109	113	unsp->			8346	HIST1H2BI	lpgelakhavSegtkavtkyt
IPI00020127	163	154		PKC+cdc2->PKC		6117	RPA1	pqngssgmgsTvskaygaskt
IPI00020127	163	162	->PKA	->ATM+DNAPK		6117	RPA1	gstvskaygaSktfgkaagps
IPI00020127	167	162	->PKA			6117	RPA1	gstvskaygaSktfgkaagps
IPI00020127	167	164	PKC->	PKC->		6117	RPA1	tvskaygaskTfgkaagpsls
IPI00020127	259	255		->EGFR		6117	RPA1	ffplievnkvYyfskgtlkia
IPI00020127	259	258	PKC+CKI->CKI+PKA	PKC+CKI->ATM+CKI+DNAPK		6117	RPA1	lievnkvyyfSkgtlkiankq
IPI00020127	259	261	unsp+PKC->PKC			6117	RPA1	vnkvyyfskgTlkiankqfta
IPI00020416	708	711	PKC->cdc2	PKC->PKC+CKII+cdc2		7174	TPP2	yrshefykfcSlpekgtltea
IPI00020416	883	878	->PKA			7174	TPP2	gsgdayphqySlklekgdyti
IPI00020416	883	886	INSR+unsp+EGFR->EGFR	INSR+unsp+EGFR->unsp+EGFR		7174	TPP2	qyslklekgdYtirlqirheq
IPI00020546	406	410	PKA->	PKA->cdc2		55870	ASH1L	vnkdigkklmScplagliskd
IPI00020559	331	327	PKC+cdc2->PKC			199870	FAM76A	rellkqaaalSkskkseksga
IPI00020559	331	329	unsp+PKC->	unsp+PKC->		199870	FAM76A	llkqaaalskSkkseksgait
IPI00020559	331	335	unsp->			199870	FAM76A	alskskksekSgaitsp----
IPI00020567	212	220	PKC->PKC+cdc2			392	ARHGAP1	vlkyddflksTqkspatapkp
IPI00020599	48	44	->unsp	->unsp		811	CALR	tsrwieskhkSdfgkfvlssg
IPI00020599	48	52	PKC->			811	CALR	hksdfgkfvlSsgkfygdeek
IPI00020599	48	53	unsp+PKC->PKC	unsp+PKC->PKC		811	CALR	ksdfgkfvlsSgkfygdeekd
IPI00020599	209	214	unsp+CKII->CKII			811	CALR	ppkkikdpdaSkpedwderak
IPI00020602	97	93		PKA->PKG+PKA		1459	CSNK2A2	vkilenlrggTniikldtvk
IPI00020602	97	101	unsp+PKC->unsp			1459	CSNK2A2	ggtniiklidTvkdpvsktpa
IPI00020618	6	2	PKC->			8369	HIST1H4G	---------mSvrgkagkglg
IPI00020898	81	89	unsp->unsp+cdc2			6197	RPS6KA3	fgkvflvkkiSgsdarqlyam
IPI00020906	49	46	->CKII			3612	IMPA1	ksspvdlvtaTdqkvekmlis
IPI00020956	44	40	->PKA	->PKG		3068	HDGF	idempeaavkStankyqvfff
IPI00020956	44	41	PKC+PKG->PKG			3068	HDGF	dempeaavksTankyqvfffg
IPI00020956	44	45	INSR->			3068	HDGF	eaavkstankYqvfffgthet
IPI00020965	60	65	unsp->	unsp->GSK3		7328	UBE2H	vdlpdkypfkSpsigfmnkif
IPI00020985	77	76		unsp+PKC->ATM+unsp+PKC+DNAPK		2033	EP300	tslgmvqdaaSkhkqlsellr
IPI00020985	77	82	->cdc2	->cdc2		2033	EP300	qdaaskhkqlSellrsgsspn
IPI00020985	79	76	unsp+PKC->			2033	EP300	tslgmvqdaaSkhkqlsellr
IPI00020985	79	82	->cdc2	->cdc2		2033	EP300	qdaaskhkqlSellrsgsspn
IPI00020985	79	87	->cdc2			2033	EP300	khkqlsellrSgsspnlnmgv
IPI00020985	291	285	GSK3+cdk5->GSK3	GSK3+cdk5->GSK3	Uniprot	2033	EP300	qtktvlsnnlSpfamdkkavp
IPI00020985	292	285	GSK3+cdk5->GSK3+cdk5+p38MAPK	GSK3+cdk5->GSK3	Uniprot	2033	EP300	qtktvlsnnlSpfamdkkavp
IPI00020985	373	371	PKC->	PKC->		2033	EP300	vrqcnlphcrTmknvlnhmth
IPI00020985	386	384	PKC->	PKC->		2033	EP300	nvlnhmthcqSgkscqvahca
IPI00020985	404	401	PKC->			2033	EP300	ahcassrqiiShwknctrhdc
IPI00020985	569	564	PKC->			2033	EP300	gpmptaaqpsTtgirkqwhed
IPI00020985	569	565	PKC->			2033	EP300	pmptaaqpstTgirkqwhedi
IPI00020985	636	638		unsp->unsp+EGFR	PhosphoSite	2033	EP300	eyyhllaekiYkiqkeleekr
IPI00020985	1001	1000	unsp+CKII->unsp+CKI+CKII	unsp+CKII->ATM+unsp+CKII+DNAPK		2033	EP300	adtqpediseSkvedckmest
IPI00020985	1024	1031	unsp->unsp+cdc2	unsp->unsp+cdc2		2033	EP300	teikeeedqpStsatqsspap
IPI00020985	1045	1044	unsp+PKC+PKG->unsp+PKC	unsp+PKC+PKG->ATM+unsp		2033	EP300	atqsspapgqSkkkifkpeel
IPI00020985	1046	1044	unsp+PKC+PKG->	unsp+PKC+PKG->		2033	EP300	atqsspapgqSkkkifkpeel
IPI00020985	1047	1044	unsp+PKC+PKG->			2033	EP300	atqsspapgqSkkkifkpeel
IPI00020985	1094	1095	unsp+GSK3+cdk5->unsp+cdk5	unsp+GSK3+cdk5->unsp+cdk5		2033	EP300	gipdyfdivkSpmdlstikrk
IPI00020985	1144	1136	unsp+PKG+PKA+RSK->unsp+PKG+cdc2+PKA			2033	EP300	nnawlynrktSrvykycskls
IPI00020985	1144	1143		->ATM		2033	EP300	rktsrvykycSklsevfeqei
IPI00020985	1167	1171	unsp->cdk5	unsp->unsp+cdk5		2033	EP300	gyccgrklefSpqtlccygkq
IPI00020985	1180	1174	CKI->	CKI->		2033	EP300	cgrklefspqTlccygkqlct
IPI00020985	1228	1224	PKC->	PKC->		2033	EP300	slgddpsqpqTtinkeqfskr
IPI00020985	1228	1225	unsp->	unsp->		2033	EP300	lgddpsqpqtTinkeqfskrk
IPI00020985	1228	1232		unsp+PKC->unsp+PKC+cdc2		2033	EP300	pqttinkeqfSkrkndtldpe
IPI00020985	1331	1329	unsp->CKII	unsp->		2033	EP300	gevtvrvvhaSdktvevkpgm
IPI00020985	1331	1332	->PKC	->unsp+PKC		2033	EP300	tvrvvhasdkTvevkpgmkar
IPI00020985	1427	1430	unsp->	unsp->		2033	EP300	gyleyvkklgYttghiwacpp
IPI00020985	1427	1431	->cdc2	->cdc2		2033	EP300	yleyvkklgyTtghiwacpps
IPI00020985	1499	1496	unsp+PKC->unsp	unsp+PKC->unsp		2033	EP300	ifkqatedrlTsakelpyfeg
IPI00020985	1499	1497	unsp+PKG+RSK->unsp+PKA+RSK	unsp+PKG+RSK->unsp+PKA+RSK		2033	EP300	fkqatedrltSakelpyfegd
IPI00020985	1499	1503	unsp->			2033	EP300	drltsakelpYfegdfwpnvl
IPI00020985	1542	1537	unsp+cdc2->unsp	unsp+cdc2->		2033	EP300	rkreentsneStdvtkgdskn
IPI00020985	1542	1541		CKI+PKC->ATM+CKI+PKC+DNAPK		2033	EP300	entsnestdvTkgdsknakkk
IPI00020985	1546	1537		unsp+cdc2->unsp		2033	EP300	rkreentsneStdvtkgdskn

Table S3

IPI00020985	1546	1541	CKI+PKC->CKI	CKI+PKC->PKC		2033	EP300	entsnestdvTkgdsknakkk
IPI00020985	1546	1545		PKC->DNAPK		2033	EP300	nestdvtkgdSknakkknnkk
IPI00020985	1549	1545	PKC->unsp	PKC->unsp		2033	EP300	nestdvtkgdSknakkknnkk
IPI00020985	1550	1545	PKC->			2033	EP300	nestdvtkgdSknakkknnkk
IPI00020985	1551	1556	unsp+PKC+PKG->unsp+PKC			2033	EP300	knakkknnkkTsknksslsrg
IPI00020985	1554	1556	unsp+PKC+PKG->PKC	unsp+PKC+PKG->unsp+PKC		2033	EP300	knakkknnkkTsknksslsrg
IPI00020985	1554	1557	unsp+PKC->PKC	unsp+PKC->PKC		2033	EP300	nakkknnkktSknksslsrgn
IPI00020985	1555	1556	unsp+PKC+PKG->unsp+PKC	unsp+PKC+PKG->unsp+PKC		2033	EP300	knakkknnkkTsknksslsrg
IPI00020985	1555	1557	unsp+PKC->PKC			2033	EP300	nakkknnkktSknksslsrgn
IPI00020985	1558	1556	unsp+PKC+PKG->PKC	unsp+PKC+PKG->PKC		2033	EP300	knakkknnkkTsknksslsrg
IPI00020985	1558	1557		unsp+PKC->unsp+PKC+DNAPK		2033	EP300	nakkknnkktSknksslsrgn
IPI00020985	1558	1561	unsp+PKC->	unsp+PKC->PKC		2033	EP300	knnkktsknkSslsrgnkkkp
IPI00020985	1558	1562	unsp->			2033	EP300	nnkktsknksSlsrgnkkkpg
IPI00020985	1558	1564	unsp+PKC->unsp+CKI+PKC	unsp+PKC->unsp+CKI+PKC		2033	EP300	kktsknksslSrgnkkkpgmp
IPI00020985	1560	1556	unsp+PKC+PKG->unsp+PKC	unsp+PKC+PKG->PKC+PKG		2033	EP300	knakkknnkkTsknksslsrg
IPI00020985	1560	1557	unsp+PKC->PKC			2033	EP300	nakkknnkktSknksslsrgn
IPI00020985	1560	1561	unsp+PKC->PKC	unsp+PKC->PKC		2033	EP300	knnkktsknkSslsrgnkkkp
IPI00020985	1560	1562	unsp->	unsp->unsp+DNAPK		2033	EP300	nnkktsknksSlsrgnkkkpg
IPI00020985	1560	1564	unsp+PKC->unsp+CKI+PKC	unsp+PKC->unsp+CKI+PKC		2033	EP300	kktsknksslSrgnkkkpgmp
IPI00020985	1568	1562	unsp->unsp+PKA			2033	EP300	nnkktsknksSlsrgnkkkpg
IPI00020985	1568	1564	unsp+PKC->unsp			2033	EP300	kktsknksslSrgnkkkpgmp
IPI00020985	1583	1587	PKC->			2033	EP300	sndlsqklyaTmekhkevffv
IPI00020985	1590	1587	PKC->			2033	EP300	sndlsqklyaTmekhkevffv
IPI00020985	1592	1587	PKC->			2033	EP300	sndlsqklyaTmekhkevffv
IPI00020985	1674	1668	INSR->	INSR->		2033	EP300	lhtqsqdrfvYtcneckhhve
IPI00020985	1699	1696	->EGFR	->EGFR		2033	EP300	cedydlcitcYntknhdhkme
IPI00020985	1699	1698		->DNAPK		2033	EP300	dydlcitcynTknhdhkmekl
IPI00020985	1760	1757	PKC->			2033	EP300	qcrnancslpScqkmkrvvqh
IPI00020985	1762	1757	PKC->			2033	EP300	qcrnancslpScqkmkrvvqh
IPI00020985	1769	1768		PKC->DNAPK		2033	EP300	cqkmkrvvqhTkgckrktngg
IPI00020985	1772	1768	PKC->	PKC->		2033	EP300	cqkmkrvvqhTkgckrktngg
IPI00021048	884	886	unsp+cdc2->cdc2			26509	MYOF	tsglvgrhkfSdvtgkiklkr
IPI00021062	70	62	unsp->unsp+cdc2			1409	CRYAA	lfrtvldsgiSevrsdrdkfv
IPI00021062	78	81	unsp->unsp+GSK3			1409	CRYAA	fvifldvkhfSpedltvkvqd
IPI00021062	88	86	unsp+PKC->	unsp+PKC->		1409	CRYAA	dvkhfspedlTvkvqddfvei
IPI00021062	145	140	unsp+PKC->unsp	unsp+PKC->unsp	PHOsphoELM|PhosphoSite	1409	CRYAA	scslsadgmlTfcgpkiqtgl
IPI00021088	362	353	->cdc2			8514	KCNAB2	lsssiiheidSilgnkpyskk
IPI00021088	362	361	unsp+PKC->unsp	unsp+PKC->ATM+unsp		8514	KCNAB2	idsilgnkpySkkdyrs----
IPI00021088	362	365	INSR+unsp->	INSR+unsp->		8514	KCNAB2	lgnkpyskkdYrs--------
IPI00021088	362	367	unsp->	unsp->		8514	KCNAB2	nkpyskkdyrS----------
IPI00021129	592	591		unsp+PKC->ATM+unsp+PKC+DNAPK		8546	AP3B1	ivpnvksgalSkyakkiflaq
IPI00021175	504	500	unsp+PKC->	unsp+PKC->PKC		51755	CDK12	hlvkdlkaqgTrdskpialke
IPI00021175	504	503	PKA->CKI+PKA	PKA->ATM+PKA+DNAPK		51755	CDK12	kdlkaqgtrdSkpialkeeiv
IPI00021187	2	10	PKC->PKC+cdc2			8607	RUVBL1	-mkieevkstTktqriashsh
IPI00021187	453	454	INSR+unsp->unsp			8607	RUVBL1	akiladqqdkYmk--------
IPI00021263	49	45	PKC->PKA			7534	YWHAZ	elsneernllSvayknvvgar
IPI00021263	68	63	unsp+PKG->unsp+PKG+PKA	unsp+PKG->unsp+PKG+PKA		7534	YWHAZ	garrsswrvvSsieqktegae
IPI00021263	68	64	PKC->	PKC->unsp		7534	YWHAZ	arrsswrvvsSieqktegaek
IPI00021263	68	69		CKII->unsp+CKII		7534	YWHAZ	wrvvssieqkTegaekkqqma
IPI00021263	115	110	unsp+DNAPK->DNAPK	unsp+DNAPK->DNAPK		7534	YWHAZ	llekflipnaSqaeskvfylk
IPI00021263	115	114		->DNAPK		7534	YWHAZ	flipnasqaeSkvfylkmkgd
IPI00021263	115	118	unsp->	unsp->		7534	YWHAZ	nasqaeskvfYlkmkgdyyry
IPI00021263	120	118	unsp->			7534	YWHAZ	nasqaeskvfYlkmkgdyyry
IPI00021263	122	118		unsp->unsp+EGFR		7534	YWHAZ	nasqaeskvfYlkmkgdyyry
IPI00021263	122	125	unsp->	unsp->		7534	YWHAZ	kvfylkmkgdYyrylaevaag
IPI00021263	122	126	unsp->	unsp->	PhosphoSite	7534	YWHAZ	vfylkmkgdyYrylaevaagd
IPI00021266	70	64	unsp+PKG->unsp+PKG+PKA			6147	RPL23A	lrrqpkyprkSaprrnkldhy
IPI00021266	70	74	unsp->			6147	RPL23A	saprrnkldhYaiikfpltte
IPI00021290	86	94	PKC->PKC+cdc2			47	ACLY	wlkprlgqeaTvgkatgflkn
IPI00021290	948	947		->ATM+DNAPK		47	ACLY	galdaaakmfSkafdsgiipm
IPI00021327	6	12	unsp->unsp+CKII			2885	GRB2	eaiakydfkaTaddelsfkrg
IPI00021327	50	52		->EGFR	PhosphoSite	2885	GRB2	ngkdgfipknYiemkphpwff
IPI00021347	131	130		CKI+cdc2->ATM+CKI+cdc2+DNAPK		7332	UBE2L3	plradlaeeySkdrkkfckna
IPI00021370	14	19		unsp+CKI->unsp		3093	UBE2K	ikrefkevlkSeetsknqikv
IPI00021405	97	91	unsp->unsp+PKA	unsp->unsp+PKA		4000	LMNA	yeaelgdarkTldsvakerar
IPI00021405	97	94	unsp+PKC->unsp			4000	LMNA	elgdarktldSvakerarlql
IPI00021405	108	107	unsp+cdc2->cdc2	unsp+cdc2->ATM+unsp+cdc2+DNAPK	PhosphoSite	4000	LMNA	kerarlqlelSkvreefkelk
IPI00021405	233	239	->CKII			4000	LMNA	idngkqrefeSrladalqelr
IPI00021405	311	307	PKC+DNAPK->DNAPK	PKC+DNAPK->DNAPK		4000	LMNA	iridslsaqlSqlqkqlaake
IPI00021405	417	409	PKC+DNAPK->PKC+cdc2+DNAPK		Uniprot|PHOsphoELM|PhosphoSi	4000	LMNA	rgrasshssqTqgggsvtkkr
IPI00021428	52	54	unsp->			58	ACTA1	gvmvgmgqkdSyvgdeaqskr
IPI00021428	52	55	INSR+unsp->	INSR+unsp->unsp	iprot|PHOsphoELM|PhosphoSi	58	ACTA1	vmvgmgqkdsYvgdeaqskrg
IPI00021428	63	54	unsp->unsp+cdc2			58	ACTA1	gvmvgmgqkdSyvgdeaqskr
IPI00021428	63	62	unsp+PKC+cdc2->unsp+CKI+cdc2	unsp+PKC+cdc2->ATM+unsp+cdc2+DNAPK		58	ACTA1	kdsyvgdeaqSkrgiltlkyp
IPI00021428	70	68	unsp+PKC->PKC	unsp+PKC->		58	ACTA1	deaqskrgilTlkypiehgii
IPI00021428	193	188	->PKA			58	ACTA1	mrldlagrdlTdylmkilter
IPI00021428	328	325	unsp+PKC+cdc2->cdc2	unsp+PKC+cdc2->unsp+cdc2		58	ACTA1	mqkeitalapStmkikiiapp
IPI00021428	328	326	PKC->	PKC->		58	ACTA1	qkeitalapsTmkikiiappe
IPI00021428	330	325	unsp+PKC+cdc2->cdc2	unsp+PKC+cdc2->cdc2		58	ACTA1	mqkeitalapStmkikiiapp
IPI00021428	330	326	PKC->			58	ACTA1	qkeitalapsTmkikiiappe
IPI00021435	407	405	unsp+PKC->	unsp+PKC->		5701	PSMC2	fairarrkiaTekdfleavnk
IPI00021439	50	52	unsp->			60	ACTB	gvmvgmgqkdSyvgdeaqskr
IPI00021439	50	53	INSR+unsp->	INSR+unsp->unsp	PhosphoSite	60	ACTB	vmvgmgqkdsYvgdeaqskrg
IPI00021439	61	52	unsp->unsp+cdc2			60	ACTB	gvmvgmgqkdSyvgdeaqskr
IPI00021439	61	60	unsp+PKC+cdc2->unsp+CKI+cdc2	unsp+PKC+cdc2->ATM+unsp+cdc2+DNAPK		60	ACTB	kdsyvgdeaqSkrgiltlkyp
IPI00021439	326	323	unsp+PKC+cdc2->cdc2	unsp+PKC+cdc2->unsp+cdc2		60	ACTB	mqkeitalapStmkikiiapp
IPI00021439	326	324	PKC->	PKC->		60	ACTB	qkeitalapsTmkikiiappe
IPI00021440	50	52	unsp->			71	ACTG1	gvmvgmgqkdSyvgdeaqskr
IPI00021440	50	53	INSR+unsp->	INSR+unsp->unsp	PhosphoSite	71	ACTG1	vmvgmgqkdsYvgdeaqskrg
IPI00021440	61	52	unsp->unsp+cdc2			71	ACTG1	gvmvgmgqkdSyvgdeaqskr
IPI00021440	61	60	unsp+PKC+cdc2->unsp+CKI+cdc2	unsp+PKC+cdc2->ATM+unsp+cdc2+DNAPK		71	ACTG1	kdsyvgdeaqSkrgiltlkyp
IPI00021440	191	186	->PKA			71	ACTG1	lrldlagrdlTdylmkilter
IPI00021440	326	323	unsp+PKC+cdc2->cdc2	unsp+PKC+cdc2->unsp+cdc2	PhosphoSite	71	ACTG1	mqkeitalapStmkikiiapp
IPI00021440	326	324	PKC->	PKC->		71	ACTG1	qkeitalapsTmkikiiappe
IPI00021518	278	280		PKC->CaM-II		1643	DDB2	wdlrqvrgkaSflyslphrhp
IPI00021700	77	73	PKC->	PKC->		5111	PCNA	drnlamgvnlTsmskilkcag
IPI00021700	77	76		->ATM+DNAPK		5111	PCNA	lamgvnltsmSkilkcagned
IPI00021700	80	76	->PKA			5111	PCNA	lamgvnltsmSkilkcagned
IPI00021766	1104	1102	unsp->	unsp->		57142	RTN4	snsalghvncTikelrrlflv
IPI00021805	42	40	unsp+PKC+PKG->PKC	unsp+PKC+PKG->		4257	MGST1	mstatafyrlTrkvfanpedc
IPI00021812	884	886	unsp+PKC->PKC	unsp+PKC->		79026	AHNAK	kmpkmkmpkfSmpgfkaegpe
IPI00021840	30	26	PKC->	PKC->		6194	RPS6	ievdderklrTfyekrmatev
IPI00021840	203	199		unsp+PKC->PKC		6194	RPS6	rrrialkkqrTkknkeeaaey
IPI00021840	211	209	unsp->			6194	RPS6	tkknkeeaaeYakllakrmke
IPI00021885	620	618	unsp+PKC->cdc2	unsp+PKC->	PHOsphoELM	2243	FGA	gseadhegthStkrghaksrp
IPI00021885	625	618	unsp+PKC->unsp+PKC+cdc2		PHOsphoELM	2243	FGA	gseadhegthStkrghaksrp
IPI00021885	625	626	unsp->	unsp->		2243	FGA	thstkrghakSrpvrdcddvl
IPI00021924	47	49	PKA->	PKA->ATM		8971	H1FX	skkknqpgkySqlvvetirrl
IPI00021926	72	74	PKC+PKG->PKC	PKC+PKG->		5706	PSMC6	lteekfivkaTngpryvvgcr
IPI00021926	206	201	PKC->			5706	PSMC6	ldcnflkvvsSsivdkyiges
IPI00021926	206	202	unsp->unsp+PKA			5706	PSMC6	dcnflkvvssSivdkyigesa
IPI00021926	206	207	INSR->		Uniprot|PhosphoSite	5706	PSMC6	kvvsssivdkYigesarlire
IPI00021926	206	211	unsp->	unsp->		5706	PSMC6	ssivdkyigeSarliremfny
IPI00022018	197	204	->PKA			8813	DPM1	vlekliekcvSkgyvfqmemi
IPI00022055	416	411	PKC+cdc2->cdc2			8850	KAT2B	ssleqpnagsSspackassgl
IPI00022055	416	412		->unsp		8850	KAT2B	sleqpnagssSpackassgle
IPI00022055	416	418	PKA->	PKA->		8850	KAT2B	agssspackaSsgleanpgek
IPI00022055	416	419	unsp+CKII->cdc2+CKII	unsp+CKII->cdc2+CKII		8850	KAT2B	gssspackasSgleanpgekr
IPI00022055	428	419	unsp+CKII->unsp+cdc2+CKII			8850	KAT2B	gssspackasSgleanpgekr
IPI00022055	428	434	unsp+CKII->unsp+cdc2+CKII			8850	KAT2B	npgekrkmtdShvleeakkpr
IPI00022055	430	432	PKG+PKA->PKA	PKG+PKA->unsp+PKA		8850	KAT2B	eanpgekrkmTdshvleeakk
IPI00022055	430	434	unsp+CKII->CKII	unsp+CKII->unsp+cdc2+CKII		8850	KAT2B	npgekrkmtdShvleeakkpr
IPI00022055	441	434	unsp+CKII->unsp+cdc2+CKII			8850	KAT2B	npgekrkmtdShvleeakkpr
IPI00022055	733	729	unsp->	unsp->	Uniprot|PhosphoSite	8850	KAT2B	skeprdpdqlYstlksilqqv
IPI00022055	733	731	PKC->cdc2	PKC->		8850	KAT2B	eprdpdqlysTlksilqqvks
IPI00022055	733	734	->PKC	->PKC		8850	KAT2B	dpdqlystlkSilqqvkshqs
IPI00022145	9	13	EGFR->		PhosphoSite	64710	NUCKS1	rpvrnrkvvdYsqfqesddad
IPI00022145	35	30	unsp->unsp+PKA	unsp->unsp+PKA	PhosphoSite	64710	NUCKS1	ddadedygrdSgpptkkirss

IPI00022145	35	34		unsp+PKC->ATM+unsp+PKC	PhosphoSite	64710	NUCKS1	edygrdsgppTkkirssprea
IPI00022145	35	40		unsp+PKC+RSK->unsp+RSK	PhosphoSite	64710	NUCKS1	sgpptkkirsSpreaknkrrs
IPI00022145	52	50	unsp+PKC+PKA->unsp+PKA	unsp+PKC+PKA->unsp+PKA	PhosphoSite	64710	NUCKS1	spreaknkrrSgknsqedsed
IPI00022145	175	177	PKC+PKG->PKC	PKC+PKG->	Uniprot|PhosphoSite	64710	NUCKS1	kkmpkprlkaTvtpspvkgkg
IPI00022145	184	181	unsp+cdk5->unsp	unsp+cdk5->unsp	niprot|PHOsphoELM|PhosphoSi	64710	NUCKS1	kprlkatvtpSpvkgkgkvgr
IPI00022145	188	193		unsp+PKC->unsp		64710	NUCKS1	vkgkgkvgrpTaskaskektp
IPI00022145	196	193	unsp+PKC->unsp	unsp+PKC->unsp		64710	NUCKS1	vkgkgkvgrpTaskaskektp
IPI00022145	196	195	PKC->PKC+DNAPK	PKC->DNAPK		64710	NUCKS1	gkgkvgrptaSkaskektpsp
IPI00022145	196	198		unsp+PKC->unsp		64710	NUCKS1	kvgrptaskaSkektpspkee
IPI00022145	196	202		unsp+GSK3->unsp	niprot|PHOsphoELM|PhosphoSi	64710	NUCKS1	ptaskaskekTpspkeedeep
IPI00022202	209	208	INSR->			5250	SLC25A3	ntlrdaapkmYkeeglkafyk
IPI00022202	214	208	INSR->	INSR->		5250	SLC25A3	ntlrdaapkmYkeeglkafyk
IPI00022215	1035	1031	->PKA			23394	ADNP	gdreqlkwknSsygkvegfws
IPI00022215	1035	1032	unsp+PKC+PKA->PKA	unsp+PKC+PKA->PKC+PKA		23394	ADNP	dreqlkwknsSygkvegfwsk
IPI00022215	1035	1033	unsp+EGFR->unsp			23394	ADNP	reqlkwknssYgkvegfwskd
IPI00022215	1042	1041		->ATM+DNAPK		23394	ADNP	ssygkvegfwSkdqsqwknas
IPI00022215	1042	1045	PKC+DNAPK->DNAPK			23394	ADNP	kvegfwskdqSqwknasende
IPI00022229	2004	2001	unsp+PKC->			338	APOB	eysqdldaynTkdkigveltg
IPI00022276	133	134	unsp+EGFR->unsp			28957	MRPS28	crrpevdgekYqkgtrvrlrl
IPI00022314	68	69	unsp+EGFR->SRC+unsp	unsp+EGFR->SRC+unsp+EGFR		6648	SOD2	vnnlnvteekYqealakgdvt
IPI00022314	132	136	PKG->	PKG->		6648	SOD2	gsfdkfkeklTaasvgvqgsg
IPI00022348	487	482	unsp+PKC+DNAPK->unsp+DNAPK			5371	PML	ttaqkrkcsqTqcprkvikme
IPI00022348	515	512	unsp->unsp+PKA		Uniprot|PhosphoSite	5371	PML	arsspeqprpStskavspphl
IPI00022348	515	513	unsp+PKC->unsp	unsp+PKC->unsp		5371	PML	rsspeqprpsTskavspphld
IPI00022348	515	514	unsp->	unsp->ATM+unsp+DNAPK		5371	PML	sspeqprpstSkavspphldg
IPI00022373	343	338	unsp+PKC+cdk5+p38MAPK->unsp+p38MAPK	unsp+PKC+cdk5+p38MAPK->unsp+p38MAPK		28987	NOB1	nprglryslpTpkggkyainp
IPI00022443	413	415	PKG->	PKG->		174	AFP	iqesqalakrScglfqklgey
IPI00022449	67	62	PKC->			1794	DOCK2	hkmlqgifpkSfihikevtve
IPI00022449	738	736	PKC->	PKC->		1794	DOCK2	rgeqcepilrTlkaleyvfkf
IPI00022465	1721	1720	unsp->unsp+DNAPK	unsp->ATM+unsp+DNAPK		11113	CIT	vvilrynenlSkycirkeiet
IPI00022597	3	6	unsp+PKC->unsp+CKI+PKC			9040	UBE2M	-----miklfSlkqqkkees
IPI00022648	55	52		unsp->		1983	EIF5	vakalnrpptYptkyfgcelg
IPI00022648	55	54		->DNAPK		1983	EIF5	kalnrpptypTkyfgcelgaq
IPI00022744	158	155	PKC->PKA	PKC->PKC+PKA		1434	CSE1L	vingvlrtahSlfkryrhefk
IPI00022744	574	571	unsp->SRC+unsp			1434	CSE1L	ltlpgsseneYimkaimrsfs
IPI00022774	8	3	PKC->	PKC->	Uniprot|PhosphoSite	7415	VCP	--------maSgadskgddls
IPI00022774	8	7	unsp->unsp+CKI	unsp->ATM+unsp+DNAPK	Uniprot|PhosphoSite	7415	VCP	----masgadSkgddlstail
IPI00022774	8	14	PKC->PKC+cdc2	PKC->PKC+cdc2	PhosphoSite	7415	VCP	gadskgddlsTailkqknrpn
IPI00022774	18	14	PKC->	PKC->	PhosphoSite	7415	VCP	gadskgddlsTailkqknrpn
IPI00022774	60	56	->PKA		PhosphoSite	7415	VCP	mdelqlfrgdTvllkgkkrre
IPI00022774	81	78	unsp+CKII->CKII			7415	VCP	vcivlsddtcSdekirmnrvv
IPI00022774	251	249	unsp->	unsp->	PhosphoSite	7415	VCP	rgillygppgTgktliarava
IPI00022774	386	385		cdc2->cdc2+DNAPK	PhosphoSite	7415	VCP	tgrleilqihTknmkladdvd
IPI00022774	505	509		unsp->unsp+p38MAPK	Uniprot|PhosphoSite	7415	VCP	hpdkflkfgmTpskgvlfygp
IPI00022774	505	511	->PKA		PhosphoSite	7415	VCP	dkflkfgmtpSkgvlfygppg
IPI00022774	512	509	unsp->p38MAPK		Uniprot|PhosphoSite	7415	VCP	hpdkflkfgmTpskgvlfygp
IPI00022774	512	511	->PKA	->ATM+DNAPK	PhosphoSite	7415	VCP	dkflkfgmtpSkgvlfygppg
IPI00022774	614	612	unsp+PKC->cdc2	unsp+PKC->	PhosphoSite	7415	VCP	nqiltemdgmStkknvfiiga
IPI00022774	658	664		unsp+GSK3->unsp	PhosphoSite	7415	VCP	vailkanlrkSpvakdvdlef
IPI00022774	663	664	unsp+GSK3->unsp	unsp+GSK3->unsp	PhosphoSite	7415	VCP	vailkanlrkSpvakdvdlef
IPI00022774	668	664	unsp+GSK3->unsp+GSK3+PKA	unsp+GSK3->unsp	PhosphoSite	7415	VCP	vailkanlrkSpvakdvdlef
IPI00022774	696	702	unsp->unsp+PKA		Uniprot|PhosphoSite	7415	VCP	qracklaireSieseirrere
IPI00022793	72	67	unsp+PKC+cdc2->PKC+cdc2	unsp+PKC+cdc2->PKC+cdc2		3032	HADHB	vdgvrtpfllSgtsykdlmph
IPI00022793	188	191	unsp+PKC->	unsp+PKC->PKC		3032	HADHB	klmldlnkakSmgqrlslisk
IPI00022793	201	197	unsp+PKC->unsp+PKC+PKA	unsp+PKC->unsp+PKC+PKA		3032	HADHB	nkaksmgqrlSliskfrfnfl
IPI00022793	201	200		PKC->ATM		3032	HADHB	ksmgqrlsliSkfrfnflape
IPI00022793	348	356	->cdc2			3032	HADHB	dpkdqlllgpTyatpkvleka
IPI00022865	68	69	unsp+PKC+PKG->unsp+PKC	unsp+PKC+PKG->unsp+PKC		890	CCNA2	rglaqqqrpkTrrvaplkdlp
IPI00022865	95	94	->PKA	->ATM+DNAPK		890	CCNA2	hvtvppwkanSkqpaftihvd
IPI00022891	96	95		INSR+unsp->INSR+unsp+EGFR		291	SLC25A4	qalnfafkdkYkqlflggvdr
IPI00023006	63	54	unsp->unsp+cdc2			70	ACTC1	gvmvgmgqkdSyvgdeaqskr
IPI00023006	63	62	unsp+PKC+cdc2->unsp+CKI+cdc2	unsp+PKC+cdc2->ATM+unsp+cdc2+DNAPK		70	ACTC1	kdsyvgdeaqSkrgiltlkyp
IPI00023006	70	68	unsp+PKC->PKC	unsp+PKC->		70	ACTC1	deaqskrgilTlkypiehgii
IPI00023084	456	461	unsp+PKG+PKA->unsp			57567	ZNF319	caaaekplrcTlcerrffsss
IPI00023161	230	225	unsp+CaM-II+RSK->CaM-II	unsp+CaM-II+RSK->RSK		26589	MRPL46	fkfpqamrteSnlgakvfffk
IPI00023161	265	263	unsp->			26589	MRPL46	vwvtkdelgdYlkpkylaqvr
IPI00023234	271	268	PKC->			10054	UBA2	lfkddiryllTmdklwrkrkp
IPI00023234	324	323		PKA+RSK->PKA+DNAPK+RSK		10054	UBA2	ldvksyarlfSksietlrvhl
IPI00023234	617	621	unsp+PKC->unsp			10054	UBA2	krkldekenlSakrsrieqke
IPI00023339	1014	1013		unsp->ATM+unsp+DNAPK		1387	CREBBP	aedtepdpgeSkgeprsemme
IPI00023339	1203	1204	unsp->			1387	CREBBP	qslgyccgrkYefspqtlccy
IPI00023339	1203	1207	->cdk5	->cdk5		1387	CREBBP	gyccgrkyefSpqtlccygkq
IPI00023339	1535	1532	unsp+PKC->unsp	unsp+PKC->unsp		1387	CREBBP	ifkqatedrlTsakelpyfeg
IPI00023339	1535	1533	unsp+PKG+RSK->unsp+PKA+RSK	unsp+PKG+RSK->unsp+PKA+RSK		1387	CREBBP	fkqatedrltSakelpyfegd
IPI00023339	1535	1539	unsp->			1387	CREBBP	drltsakelpYfegdfwpnvl
IPI00023339	1583	1582		PKC->ATM+PKC+DNAPK		1387	CREBBP	settegsqgdSknakkknnkk
IPI00023339	1586	1582	PKC->unsp	PKC->unsp+PKC		1387	CREBBP	settegsqgdSknakkknnkk
IPI00023339	1588	1582		PKC->PKC+cdc2		1387	CREBBP	settegsqgdSknakkknnkk
IPI00023339	1588	1593	PKC+PKG->PKC			1387	CREBBP	knakkknnkkTnknkssisra
IPI00023339	1591	1582	PKC->PKC+cdc2			1387	CREBBP	settegsqgdSknakkknnkk
IPI00023339	1591	1593	PKC+PKG->PKC	PKC+PKG->PKC		1387	CREBBP	knakkknnkkTnknkssisra
IPI00023339	1592	1593	PKC+PKG->PKC	PKC+PKG->PKC		1387	CREBBP	knakkknnkkTnknkssisra
IPI00023339	1595	1593	PKC+PKG->	PKC+PKG->		1387	CREBBP	knakkknnkkTnknkssisra
IPI00023339	1597	1593	PKC+PKG->PKC			1387	CREBBP	knakkknnkkTnknkssisra
IPI00023339	1597	1599	->PKC			1387	CREBBP	nnkktnknksSisrankkkps
IPI00023339	1597	1601	unsp+PKC->			1387	CREBBP	kktnknkssiSrankkkpsmp
IPI00023339	1797	1794	PKC->			1387	CREBBP	qcrnancslpScqkmkrvvqh
IPI00023340	350	347	PKC+cdc2->cdc2	PKC+cdc2->cdc2		7994	MYST3	rpknrlkkqnTvskgpfskvr
IPI00023340	350	349	PKC->	PKC->ATM+DNAPK		7994	MYST3	knrlkkqntvSkgpfskvrtg
IPI00023340	350	354	unsp+PKC->unsp			7994	MYST3	kqntvskgpfSkvrtgpgrgr
IPI00023340	355	354	unsp+PKC->unsp	unsp+PKC->unsp+DNAPK		7994	MYST3	kqntvskgpfSkvrtgpgrgr
IPI00023340	355	358	PKC->			7994	MYST3	vskgpfskvrTgpgrgrkrki
IPI00023340	415	420	unsp+cdk5+p38MAPK->cdk5+p38MAPK	unsp+cdk5+p38MAPK->cdk5+p38MAPK	Uniprot|PhosphoSite	7994	MYST3	idgltkfftpSpdgrkargev
IPI00023340	604	605	->CKII	->CKII		7994	MYST3	llaklfldhkTlyydvepflf
IPI00023340	604	607	unsp->	unsp->SRC+unsp		7994	MYST3	aklfldhktlYydvepflfyv
IPI00023340	815	812	unsp->			7994	MYST3	pqcqereleiSvgksvshenk
IPI00023340	815	816		unsp->		7994	MYST3	ereleisvgkSvshenkeqds
IPI00023340	1007	1006		->DNAPK		7994	MYST3	sprssspilTkptlkrkkpf
IPI00023340	1007	1009		PKC->unsp+PKC		7994	MYST3	ssspiltkpTlkrkkpflhr
IPI00023343	765	759	unsp+PKC->unsp+PKC+PKA	unsp+PKC->unsp+PKC+PKA		1741	DLG3	ealmemnrrqTyeqankiydk
IPI00023591	273	270	PKC->			5813	PURA	pykvwakfghTfckyseemkk
IPI00023598	103	106	unsp->			10382	TUBB4	gagnnwakghYtegaelvdav
IPI00023598	103	107	->CKII			10382	TUBB4	agnnwakghyTegaelvdavl
IPI00023649	149	144	PKC->	PKC->		55206	SBNO1	vsaptvrnamTsapskdqvql
IPI00023649	149	148		PKC->DNAPK		55206	SBNO1	tvrnamtsapSkdqvqlkdll
IPI00023649	413	408		ATM+unsp+cdc2+DNAPK->cdc2+DNAPK		55206	SBNO1	fatyssligeSqsggkyktrl
IPI00023649	413	410	unsp+PKC+cdc2->cdc2	unsp+PKC+cdc2->unsp+CKI+PKC+cdc2		55206	SBNO1	tyssligesqSggkyktrlkq
IPI00023649	413	414	unsp->	unsp->		55206	SBNO1	ligesqsggkYktrlkqllhw
IPI00023649	413	416	PKC->			55206	SBNO1	gesqsggkykTrlkqllhwcg
IPI00023649	415	408	ATM+unsp+cdc2+DNAPK->unsp+cdc2+DNAPK	ATM+unsp+cdc2+DNAPK->unsp+cdc2+DNAPK		55206	SBNO1	fatyssligeSqsggkyktrl
IPI00023649	415	410	unsp+PKC+cdc2->cdc2	unsp+PKC+cdc2->PKC+cdc2		55206	SBNO1	tyssligesqSggkyktrlkq
IPI00023756	378	369	unsp+PKC->unsp+PKC+cdc2			3720	JARID2	dtkpnhhkpsSavnhtisgkt
IPI00023756	378	376	unsp->	unsp->		3720	JARID2	kpssavnhtiSgktessnakt
IPI00023756	378	382	unsp->			3720	JARID2	nhtisgktesSnaktrkqvls
IPI00023860	197	204	unsp->unsp+cdc2			4673	NAP1L1	khlkdikvkfSdagqpmsfvl
IPI00023860	271	269	PKC->	PKC->		4673	NAP1L1	qidwkkgknvTlktikkkqkh
IPI00023919	222	217	unsp->	unsp->		5705	PSMC5	tdctfirvsgSelvqkfigeg
IPI00024067	507	511	p38MAPK->			1213	CLTC	ivlyakkvgyTpdwifllrnv
IPI00024067	1441	1440	PKC->	PKC->DNAPK		1213	CLTC	ldhtravnyfSkvkqlplvkp
IPI00024067	1449	1440	PKC->PKC+cdc2			1213	CLTC	ldhtravnyfSkvkqlplvkp
IPI00024067	1449	1454		PKC->		1213	CLTC	qlplvkpylrSvqnhnnksvn
IPI00024097	101	105		PKA->		26136	TES	tnpvaakknvSintvtyewap
IPI00024157	160	163	unsp+PKC->unsp			2287	FKBP3	akkkknakplSfkvgvgkvir
IPI00024157	201	198		unsp->unsp+EGFR		2287	FKBP3	arleiepewaYgkkgqpdaki
IPI00024163	445	446	unsp->SRC+unsp	unsp->SRC+unsp		11128	POLR3A	nrekmaqelkYgdiverhlid
IPI00024175	227	228	INSR+unsp->	INSR+unsp->INSR		5688	PSMA7	kilnpeeiekYvaeiekekee
IPI00024214	447	442	PKC->	PKC->		7014	TERF2	vqaapdedstTnitkkqkwtv
IPI00024214	488	493	unsp+PKC+PKG->unsp+PKC			7014	TERF2	tavmikdrwrTmkrlgmn---

Table S3

IPI00024291	36	32		->unsp	PhosphoSite	572	BAD	lgpspagdgpSgsgkhhrqap
IPI00024291	36	34	unsp+PKC->	unsp+PKC->	PhosphoSite	572	BAD	pspagdgpsgSgkhhrqapgl
IPI00024316	186	187	unsp->unsp+PKC	unsp->unsp+PKC		4303	FOXO4	wwmlnpeggkSgkaprrraas
IPI00024316	407	404	->PKA			4303	FOXO4	ptllllgglpSssklatgvgl
IPI00024316	407	405	unsp+PKC+cdc2->cdc2	unsp+PKC+cdc2->cdc2		4303	FOXO4	tllllgglpsSsklatgvglc
IPI00024316	407	406		cdc2->cdc2+DNAPK		4303	FOXO4	llllgglpssSklatgvglcp
IPI00024387	241	235		unsp+cdk5+p38MAPK->unsp+GSK3+cdk5+p38MAPK		3169	FOXA1	fndcfvkvarSpdkpgkgsyw
IPI00024387	241	243	PKA->			3169	FOXA1	arspdkpgkgSywtlhpdsgn
IPI00024387	241	244	EGFR->	EGFR->		3169	FOXA1	rspdkpgkgsYwtlhpdsgnm
IPI00024466	1034	1029	unsp->PKA	unsp->		56886	UGGT1	rvfmncqsklSdmplksfyry
IPI00024466	1034	1035	unsp+PKC->PKC	unsp+PKC->PKC		56886	UGGT1	qsklsdmplkSfyryvlepei
IPI00024568	1057	1052	unsp+cdc2->cdc2	unsp+cdc2->cdc2		29998	GLTSCR1	lptlnvakaaSsgpgkpsglq
IPI00024623	284	289	unsp+DNAPK->DNAPK	unsp+DNAPK->DNAPK		36	ACADSB	ighgykyaigSlnegrigiaa
IPI00024662	106	110	unsp+PKA+RSK->PKA+RSK	unsp+PKA+RSK->unsp+RSK	Uniprot|PhosphoSite	23468	CBX5	dikskkkreqSndiargferg
IPI00024664	184	177		unsp+PKC->unsp+PKC+PKA		8078	USP5	qawdgevrqvSkhafslkqld
IPI00024664	184	182	unsp+PKC+PKA->unsp+PKA	unsp+PKC+PKA->unsp+PKA		8078	USP5	evrqvskhafSlkqldnpari
IPI00024670	162	166	PKC->			7905	REEP5	vkdlkdkakeTadaitkeakk
IPI00024670	164	166	PKC->PKC+CKII	PKC->CKII		7905	REEP5	vkdlkdkakeTadaitkeakk
IPI00024672	308	307		PKC->ATM+DNAPK		5599	MAPK8	llskmlvidaSkrisvdealq
IPI00024672	308	311	unsp+PKA->unsp+CKII+PKA	unsp+PKA->unsp+CKII+PKA		5599	MAPK8	mlvidaskriSvdealqhpyi
IPI00024787	291	290		PKC->		11001	SLC27A2	ivagatlalrTkfsasqfwdd
IPI00024787	291	293	PKC+PKG->CKI+PKC	PKC+PKG->CKI		11001	SLC27A2	gatlalrtkfSasqfwddcrk
IPI00024871	11	10		unsp+CKII->ATM+unsp+CKII		865	CBFB	-mprvvpdqrSkfeneeffrk
IPI00024884	261	252		cdc2->		6725	SRMS	gevweglwlgSlpvaikviks
IPI00024933	40	38	unsp+GSK3+cdk5->GSK3+cdk5	unsp+GSK3+cdk5->GSK3+cdk5	ıiprot|PHOsphoELM|PhosphoSi	6136	RPL12	lapkigplglSpkkvgddiak
IPI00024933	54	59	unsp+PKC+PKA->unsp+PKC	unsp+PKC+PKA->unsp+PKC+cdc2+PKA		6136	RPL12	atgdwkglriTvkltiqnrqa
IPI00024971	103	99	PKG->PKA			5007	OSBP	saregwlfkwTnyikgyqrrw
IPI00024971	103	101		->unsp		5007	OSBP	regwlfkwtnYikgyqrrwfv
IPI00024975	797	798	->CKII	->CKII		56992	KIF15	etqtkndflkSevhdlrvvlh
IPI00024975	1009	1007	unsp+PKC->unsp	unsp+PKC->		56992	KIF15	svcektetidTlkqelkdinc
IPI00024993	118	112	INSR->	INSR->		1892	ECHS1	emqnlsfqdcYsskflkhwdh
IPI00024993	118	113		unsp+PKC->PKC		1892	ECHS1	mqnlsfqdcySskflkhwdhl
IPI00024993	118	114	PKC->	PKC->		1892	ECHS1	qnlsfqdcysSkflkhwdhlt
IPI00025019	204	209	PKG+PKA+CKII->CKII	PKG+PKA+CKII->PKG+cdc2+CKII		5689	PSMB1	amrlvkdvfiSaaerdvytgd
IPI00025084	179	183	unsp->	unsp->		826	CAPNS1	rwqaiykqfdTdrsgticsse
IPI00025087	120	116	PKC->			7157	TP53	sygfrlgflhSgtaksvtcty
IPI00025087	120	118	PKC->cdc2	PKC->		7157	TP53	gfrlgflhsgTaksvtctysp
IPI00025087	120	121	unsp->unsp+PKC	unsp->unsp+PKC		7157	TP53	lgflhsgtakSvtctyspaln
IPI00025087	164	163	unsp->			7157	TP53	pgtrvramaiYkqsqhmtevv
IPI00025087	164	166		ATM+PKC+cdc2->ATM+cdc2+DNAPK		7157	TP53	rvramaiykqSqhmtevvrrc
IPI00025087	305	303	unsp+PKC->	unsp+PKC->		7157	TP53	gephhelppgStkralpnnts
IPI00025087	305	313	->cdc2		PhosphoSite	7157	TP53	stkralpnntSsspqpkkkpl
IPI00025087	319	313		->cdc2	PhosphoSite	7157	TP53	stkralpnntSsspqpkkkpl
IPI00025087	319	314	PKC+cdc2->	PKC+cdc2->cdc2	PhosphoSite	7157	TP53	tkralpnntsSspqpkkkpld
IPI00025087	319	315	unsp->unsp+cdk5		PHOsphoELM|PhosphoSite	7157	TP53	kralpnntssSpqpkkkpldg
IPI00025087	320	313	->cdc2		PhosphoSite	7157	TP53	stkralpnntSsspqpkkkpl
IPI00025087	320	315	unsp->	unsp->	PHOsphoELM|PhosphoSite	7157	TP53	kralpnntssSpqpkkkpldg
IPI00025087	321	313	->cdc2		PhosphoSite	7157	TP53	stkralpnntSsspqpkkkpl
IPI00025087	321	314		PKC+cdc2->PKC	PhosphoSite	7157	TP53	tkralpnntsSspqpkkkpld
IPI00025087	370	366	unsp+RSK->unsp+PKA+RSK		PhosphoSite	7157	TP53	gkepggsrahSshlkskkgqs
IPI00025087	372	366		unsp+RSK->unsp	PhosphoSite	7157	TP53	gkepggsrahSshlkskkgqs
IPI00025087	372	376		unsp+PKC->unsp+PKC+cdc2	PHOsphoELM|PhosphoSite	7157	TP53	sshlkskkgqStsrhkklmfk
IPI00025087	373	371	unsp+PKC+cdc2->unsp+cdc2	unsp+PKC+cdc2->unsp+cdc2	PHOsphoELM|PhosphoSite	7157	TP53	gsrahsshlkSkkgqstsrhk
IPI00025087	373	376	unsp+PKC->unsp+PKC+cdc2	unsp+PKC->unsp+PKC+cdc2	PHOsphoELM|PhosphoSite	7157	TP53	sshlkskkgqStsrhkklmfk
IPI00025087	381	378	PKC->		PHOsphoELM|PhosphoSite	7157	TP53	hlkskkgqstSrhkklmfkte
IPI00025087	382	378	PKC->	PKC->unsp+PKC	PHOsphoELM|PhosphoSite	7157	TP53	hlkskkgqstSrhkklmfkte
IPI00025087	386	387		->unsp	PhosphoSite	7157	TP53	tsrhkklmfkTegpdsd----
IPI00025091	38	37	INSR->		Uniprot|PhosphoSite	6205	RPS11	etgkeklpryYkniglgfktp
IPI00025091	45	37	INSR->	INSR->	Uniprot|PhosphoSite	6205	RPS11	etgkeklpryYkniglgfktp
IPI00025252	94	89	PKC->			2923	PDIA3	plakvdctanTntcnkygvsg
IPI00025252	94	95	INSR+unsp->INSR	INSR+unsp->INSR		2923	PDIA3	ctantntcnkYgvsgyptlki
IPI00025252	94	98		->cdc2		2923	PDIA3	ntntcnkygvSgyptlkifrd
IPI00025252	104	98	->PKA	->cdc2+PKA		2923	PDIA3	ntntcnkygvSgyptlkifrd
IPI00025252	104	100		->EGFR		2923	PDIA3	ntcnkygvsgYptlkifrdge
IPI00025252	104	102	unsp+PKC->	unsp+PKC->		2923	PDIA3	cnkygvsgypTlkifrdgeea
IPI00025273	350	348		INSR->unsp+INSR	Uniprot|PhosphoSite	2618	GART	vmaskgypgdYtkgveitgfp
IPI00025273	350	349	unsp->	unsp->DNAPK		2618	GART	maskgypgdyTkgveitgfpe
IPI00025307	123	124	unsp->SRC+unsp	unsp->SRC+unsp		1593	CYP27A1	leqvmrqegkYpvrndmelwk
IPI00025329	80	84	PKC+PKG+PKA->PKG+PKA	PKC+PKG+PKA->PKC+PKG		6143	RPL19	rhmgigkrkgTanarmpekvt
IPI00025341	132	129	->CKII			622	BDH1	evekvveivrSslkdpekgmw
IPI00025341	132	130	unsp+CKI+PKC->unsp+CKI+PKA	unsp+CKI+PKC->unsp+CKI+PKA+DNAPK		622	BDH1	vekvveivrsSlkdpekgmwg
IPI00025346	34	39	unsp->	unsp->cdc2		5195	PEX14	iatavkflqnSrvrqsplatr
IPI00025366	327	324	unsp->unsp+CKII			1431	CS	qlqkevgkdvSdeklrdyiwn
IPI00025366	327	331	INSR+EGFR->INSR			1431	CS	kdvsdeklrdYiwntlnsgrv
IPI00025366	327	335	DNAPK->cdc2+DNAPK			1431	CS	deklrdyiwnTlnsgrvvpgy
IPI00025366	382	381		->EGFR		1431	CS	dpmfklvaqlYkivpnvlleq
IPI00025366	459	463	unsp->			1431	CS	steglmkfvdSksg-------
IPI00025416	62	53	unsp->unsp+cdc2			72	ACTG2	gvmvgmgqkdSyvgdeaqskr
IPI00025416	62	61	unsp+PKC+cdc2->unsp+CKI+cdc2	unsp+PKC+cdc2->ATM+unsp+cdc2+DNAPK		72	ACTG2	kdsyvgdeaqSkrgiltlkyp
IPI00025416	327	324	unsp+PKC+cdc2->cdc2	unsp+PKC+cdc2->unsp+cdc2		72	ACTG2	mqkeitalapStmkikiiapp
IPI00025416	327	325	PKC->	PKC->		72	ACTG2	qkeitalapsTmkikiiappe
IPI00025491	54	56	unsp+PKG+PKA->	unsp+PKG+PKA->unsp		1973	EIF4A1	giyaygfekpSaiqqrailpc
IPI00025491	174	170	->INSR	->INSR		1973	EIF4A1	grvfdmlnrrYlspkyikmfv
IPI00025491	174	172	unsp+PKC+GSK3+RSK->unsp+GSK3+RSK	unsp+PKC+GSK3+RSK->unsp+GSK3+cdk5+p38MAPK+RSK		1973	EIF4A1	vfdmlnrrylSpkyikmfvld
IPI00025491	174	175	unsp->	unsp->		1973	EIF4A1	mlnrrylspkYikmfvldead
IPI00025491	291	289	PKC->	PKC->		1973	EIF4A1	intrrkvdwlTekmhardftv
IPI00025512	123	121	unsp+CKII->CKII	unsp+CKII->CKII		3315	HSPB1	vktkdgvveiTgkheerqdeh
IPI00025546	227	225	PKC->	PKC->		4520	MTF1	yrlkahqrlhTgktfnceseg
IPI00025753	450	449	PKG->	PKG->		1828	DSG1	tgkltlknkvTkeqynmlggk
IPI00025753	450	453	INSR+unsp->	INSR+unsp->unsp		1828	DSG1	tlknkvtkeqYnmlggkyqgt
IPI00025849	101	104	unsp+PKA->unsp	unsp+PKA->unsp+PKC+CKII		723972	LOC723972	nlsgnkikdlStieplkklen
IPI00025874	226	223	->PKA			6184	RPN1	rvklasrnveSytklgnptrs
IPI00025974	6	2	PKC->	PKC->		128866	CHMP4B	---------mSvfgklfgagg
IPI00025974	114	111		unsp+EGFR->EGFR		128866	CHMP4B	tntevlknmgYaakamkaahd
IPI00026089	141	142	unsp->unsp+p38MAPK	unsp->unsp+p38MAPK	Uniprot|PhosphoSite	23451	SF3B1	rldpfadggkTpdpkmnarty
IPI00026089	333	332		unsp+PKC->ATM+unsp	Uniprot|PhosphoSite	23451	SF3B1	sigetptpgaSkrksrwdetp
IPI00026105	132	128	CKI+PKC+PKA->CKI+PKA	CKI+PKC+PKA->CKI+PKA		6342	SCP2	algfekmskgSlgikfsdrti
IPI00026105	132	134	PKC->CKI+PKC	PKC->CKI		6342	SCP2	mskgslgikfSdrtiptdkhv
IPI00026105	142	140	unsp+PKC->	unsp+PKC->		6342	SCP2	gikfsdrtipTdkhvdllink
IPI00026105	173	174	unsp->			6342	SCP2	gyagkehmekYgtkiehfaki
IPI00026105	282	281	unsp->unsp+DNAPK	unsp->ATM+unsp+DNAPK		6342	SCP2	siikmvgfdmSkeaarkcyek
IPI00026105	341	337	PKC->	PKC->		6342	SCP2	gatlvdrgdnTyggkwvinps
IPI00026105	470	473	unsp->	unsp->		6342	SCP2	vkdgpggkeaTwvvdvkngkg
IPI00026156	192	186	ATM+unsp+DNAPK->unsp+DNAPK			3059	HCLS1	dykgetekheSqrdyakgfgg
IPI00026182	273	267		PKC->PKC+cdc2		830	CAPZA2	alrrqlpvtrTkidwnkilsy
IPI00026182	273	276	unsp+PKC->PKC	unsp+PKC->PKC		830	CAPZA2	rtkidwnkilSykigkemqna
IPI00026215	80	83	unsp->			2237	FEN1	rmmengikpvYvfdgkppqlk
IPI00026215	354	349	unsp+PKC+cdc2+DNAPK->PKC+PKA+cdc2+DNAPK	unsp+PKC+cdc2+DNAPK->PKC+PKA+cdc2+DNAPK		2237	FEN1	rlddffkvtgSlssakrkepe
IPI00026215	354	351	PKC->			2237	FEN1	ddffkvtgslSsakrkepepk
IPI00026272	6	2	PKC->		Uniprot	8336	HIST1H2AM	---------mSgrgkqggkar
IPI00026309	27	19	->cdc2+PKA			51053	GMNN	qeeikeniknSsvprrtlkmi
IPI00026309	27	25	unsp+PKC->PKA	unsp+PKC->PKA		51053	GMNN	niknssvprrTlkmiqpsasg
IPI00026309	27	32	unsp->	unsp->cdc2		51053	GMNN	prrtlkmiqpSasgslvgren
IPI00026314	648	651	EGFR->	EGFR->	PhosphoSite	2934	GSN	fwealggkaaYrtsprlkdkk
IPI00026314	648	653	PKC->	PKC->		2934	GSN	ealggkaayrTsprlkdkkmd
IPI00026314	648	654	unsp+GSK3+p38MAPK->unsp+GSK3	unsp+GSK3+p38MAPK->unsp+p38MAPK		2934	GSN	alggkaayrtSprlkdkkmda
IPI00026337	21	27	unsp->unsp+CKII			8498	RANBP3	fvfqkdkgqkSpaeqknlsds
IPI00026337	23	27	unsp->unsp+CKII			8498	RANBP3	fvfqkdkgqkSpaeqknlsds
IPI00026519	167	161		CKII->		10105	PPIF	gsqffictikTdwldgkhvvf
IPI00026559	199	194	unsp+PKC->unsp			7391	USF1	pyspkseaprTtrdekrraqh
IPI00026559	199	195	unsp+PKG+CKII->unsp+CKII			7391	USF1	yspkseaprtTrdekrraqhn
IPI00026665	327	323		unsp+CKII->CKII		5859	QARS	ngicflrfddTnpekeeakff
IPI00026689	6	4	unsp+EGFR->unsp		Uniprot|PhosphoSite	983	CDK1	-------medYtkiekigegt
IPI00026689	6	5		CKII->ATM+CKII	Uniprot|PhosphoSite	983	CDK1	------medyTkiekigegty
IPI00026781	213	207	unsp+CKI->unsp	unsp+CKI->unsp	Uniprot|PhosphoSite	2194	FASN	svqflrlgmlSpegtckafdt
IPI00026781	213	211	unsp+PKC->	unsp+PKC->		2194	FASN	lrlgmlspegTckafdtagng
IPI00026781	235	234		PKC->		2194	FASN	rsegvvavllTkkslarrvya
IPI00026781	235	237	PKA->PKC	PKA->		2194	FASN	gvvavlltkkSlarrvyatil

Table S3

IPI00026781	298	295	unsp+PKC->	unsp+PKC->		2194	FASN	esfeyieahgTgtkvgdpqel
IPI00026781	298	297		unsp+PKC->unsp+PKC+DNAPK		2194	FASN	feyieahgtgTkvgdpqelng
IPI00026781	528	519	unsp+PKA->unsp+cdc2+PKA			2194	FASN	slmrldrfrdSilrsdeavkp
IPI00026781	528	523	unsp+PKC->unsp	unsp+PKC->		2194	FASN	ldrfrdsilrSdeavkpfglk
IPI00026781	1704	1707	unsp+EGFR->	unsp+EGFR->EGFR		2194	FASN	ttvgsaekraYlqarfpqlds
IPI00026781	1752	1747	unsp+CKII->PKA+CKII	unsp+CKII->PKA+CKII		2194	FASN	ggkgvdlvlnSlaeeklqasv
IPI00026781	1771	1775		ATM+DNAPK->ATM+cdc2+DNAPK		2194	FASN	rfleigkfdlSqnhplgmaif
IPI00026781	1878	1874	PKC->PKA	PKC->unsp+PKC+PKA		2194	FASN	vlkgakpklmSaisktfcpah
IPI00026781	1878	1877		->DNAPK		2194	FASN	gakpklmsaiSktfcpahksy
IPI00026781	1878	1879	->cdc2	->cdc2		2194	FASN	kpklmsaiskTfcpahksyii
IPI00026781	1995	1997	PKA->	PKA->		2194	FASN	ffqdvckpkySgtlnldrvtr
IPI00026940	83	89	PKA->cdc2+PKA	PKA->cdc2+PKA		10762	NUP50	gaggkpleglSngnnitsapp
IPI00026940	275	268	unsp->unsp+cdc2			10762	NUP50	ktdpsslgatSasfnfgkkvd
IPI00026940	275	279	unsp+cdc2->cdc2			10762	NUP50	asfnfgkkvdSsvlgslssvp
IPI00026940	275	280	unsp+PKC->	unsp+PKC->cdc2		10762	NUP50	sfnfgkkvdsSvlgslssvpl
IPI00026940	276	268	unsp->unsp+cdc2			10762	NUP50	ktdpsslgatSasfnfgkkvd
IPI00026940	276	279	unsp+cdc2->cdc2	unsp+cdc2->cdc2		10762	NUP50	asfnfgkkvdSsvlgslssvp
IPI00026940	276	280	unsp+PKC->	unsp+PKC->unsp+PKC+cdc2		10762	NUP50	sfnfgkkvdsSvlgslssvpl
IPI00026952	752	757		unsp+PKA->unsp+GSK3+PKA		11187	PKP3	klifikkkrdSpdsekssraa
IPI00026970	139	138		PKC->ATM+PKC+DNAPK		11198	SUPT16H	skngkkigvfSkdkfpgefmk
IPI00026970	513	508	unsp+PKC+PKG+PKA+RSK->unsp+PKG+PKA	unsp+PKC+PKG+PKA+RSK->unsp+PKG+PKA	Uniprot|PhosphoSite	11198	SUPT16H	geqqiqkarkSnvsyknpslm
IPI00026970	513	511	unsp+PKC->unsp	unsp+PKC->unsp		11198	SUPT16H	qiqkarksnvSyknpslmpke
IPI00026970	513	516	unsp->cdc2	unsp->cdc2		11198	SUPT16H	rksnvsyknpSlmpkephire
IPI00026970	786	791	unsp+CKII->unsp+CKI+CKII			11198	SUPT16H	knfiekvealTkeelefevpf
IPI00026970	904	899	unsp->	unsp->		11198	SUPT16H	cdlkytegvqSlnwtkimkti
IPI00026970	904	903		PKC->DNAPK	PhosphoSite	11198	SUPT16H	ytegvqslnwTkimktivddp
IPI00026970	904	908	unsp->	unsp->		11198	SUPT16H	qslnwtkimkTivddpegffe
IPI00026970	907	903	PKC->	PKC->	PhosphoSite	11198	SUPT16H	ytegvqslnwTkimktivddp
IPI00027107	82	77	PKC->	PKC->		7284	TUFM	ghvdhgkttlTaaitkilaeg
IPI00027107	82	81	->CKI			7284	TUFM	hgkttltaaiTkilaegggak
IPI00027107	91	95	unsp->			7284	TUFM	aegggakfkkYeeidnapeer
IPI00027146	457	455	unsp+PKC->	unsp+PKC->		2747	GLUD2	nlnhvsygrlTfkyerdsnyh
IPI00027146	527	524	PKC->	PKC->		2747	GLUD2	mersarqimhTamkynlgldl
IPI00027223	321	319	EGFR->			3417	IDH1	hgtvtrhyrmYqkgqetstnp
IPI00027223	321	325	->cdc2	->cdc2		3417	IDH1	hyrmyqkgqeTstnpiasifa
IPI00027223	321	327	->cdc2			3417	IDH1	rmyqkgqetsTnpiasifawt
IPI00027228	181	176		ATM+cdc2+PKA->cdc2+PKA		5188	PET112L	iygvcagkkqSqvipktvrik
IPI00027230	613	607	unsp+CKI->unsp	unsp+CKI->unsp		7184	HSP90B1	kfdesektkeSreavekefep
IPI00027230	682	678	unsp->INSR+unsp	unsp->INSR+unsp	PhosphoSite	7184	HSP90B1	qtgkdistnyYasqkktfein
IPI00027230	682	680	ATM+unsp+PKC+DNAPK->ATM+PKC+DNAPK	ATM+unsp+PKC+DNAPK->ATM+DNAPK		7184	HSP90B1	gkdistnyyaSqkktfeinpr
IPI00027230	682	684		PKC->		7184	HSP90B1	stnyyasqkkTfeinprhpli
IPI00027232	1088	1089	unsp+PKC->PKC	unsp+PKC->PKC		3480	IGF1R	melmtrgdlkSylrslrpeme
IPI00027270	77	74	->EGFR	->EGFR		6154	RPL26	gqqigkvvqvYrkkyviyier
IPI00027280	367	371	unsp+PKC->PKC			7155	TOP2B	vvkkknkagvSvkpfqvknhi
IPI00027280	373	371	unsp+PKC->PKC	unsp+PKC->PKC		7155	TOP2B	vvkkknkagvSvkpfqvknhi
IPI00027448	24	21	PKC->			10632	ATP5L	ktpalvnaavTyskprlatfw
IPI00027448	24	23	->PKA	->ATM+DNAPK		10632	ATP5L	palvnaavtySkprlatfwyy
IPI00027448	24	29	PKC+PKG->	PKC+PKG->PKG		10632	ATP5L	avtyskprlaTfwyyakvelv
IPI00027448	54	52		PKC->PKC+DNAPK		10632	ATP5L	tpaeipraiqSlkkivnsaqt
IPI00027448	66	62	PKC->			10632	ATP5L	slkkivnsaqTgsfkqltvke
IPI00027448	66	64	PKC+PKA->PKA	PKC+PKA->PKA		10632	ATP5L	kkivnsaqtgSfkqltvkeav
IPI00027451	365	370	unsp+PKC->unsp	unsp+PKC->unsp		2735	GLI1	kpyvcklpgcTkrytdpsslr
IPI00027451	365	373	unsp->INSR+unsp			2735	GLI1	vcklpgctkrYtdpsslrkhv
IPI00027451	371	370	unsp+PKC->	unsp+PKC->unsp		2735	GLI1	kpyvcklpgcTkrytdpsslr
IPI00027497	142	138	unsp+PKG->unsp+PKG+PKA+RSK			2821	GPI	kmksfcqrvrSgdwkgytgkt
IPI00027497	211	210	PKC->PKA+DNAPK	PKC->DNAPK		2821	GPI	npesslfiiaSktfttqetit
IPI00027497	211	214	unsp->	unsp->		2821	GPI	slfiiasktfTtqetitnaet
IPI00027497	252	248	PKC->	PKC->		2821	GPI	avakhfvalsTnttkvkefgi
IPI00027497	252	250	unsp+PKC->PKC	unsp+PKC->		2821	GPI	akhfvalstnTtkvkefgidp
IPI00027497	252	251	unsp+PKC->unsp	unsp+PKC->unsp+DNAPK		2821	GPI	khfvalstntTkvkefgidpq
IPI00027547	68	63	->PKA	->PKA		117159	DCD	rqapkprkqrSsllekgldga
IPI00027547	68	64	unsp+PKC+PKA+RSK->unsp+PKA+RSK			117159	DCD	qapkprkqrsSllekgldgak
IPI00027569	39	38	->DNAPK	->ATM+DNAPK		343069	HNRNPCL1	vkksdveaifSkygkiagcsv
IPI00027626	5	6		->unsp		908	CCT6A	-----maavkTlnpkaevara
IPI00027626	365	371	->PKA			908	CCT6A	tfiekcnnprSvtlliikgpnk
IPI00027626	377	371	->PKA	->PKA		908	CCT6A	tfiekcnnprSvtlliikgpnk
IPI00027626	377	373	->PKA			908	CCT6A	iekcnnprsvTllikgpnkht
IPI00027626	388	383	PKC->	PKC->		908	CCT6A	tllikgpnkhTltqikdavrd
IPI00027626	388	385	PKC->	PKC->		908	CCT6A	likgpnkhtlTqikdavrdgl
IPI00027681	39	35	unsp+PKC+DNAPK->unsp+DNAPK			4837	NNMT	ykfgsrhsaeSqilkhllknl
IPI00027776	415	414	PKC+PKA->PKA	PKC+PKA->DNAPK		2235	FECH	plcvnpvcreTksfftsqql-
IPI00027834	269	267	EGFR->	EGFR->unsp+EGFR		3191	HNRNPL	ysgcctlkieYakptrlnvfk
IPI00027834	269	271	PKG->	PKG->		3191	HNRNPL	ctlkieyakpTrlnvfkndqd
IPI00027834	475	471	cdc2->PKA+cdc2	cdc2->CKII+cdc2		3191	HNRNPL	pgqsygledgScsykdfsesr
IPI00027834	475	473	unsp+PKC+cdc2+CKII->unsp+cdc2+CKII	unsp+PKC+cdc2+CKII->unsp+cdc2+CKII		3191	HNRNPL	qsygledgscSykdfsesrnn
IPI00027834	552	544	PKA->cdc2+PKA			3191	HNRNPL	vfsgkserssSgllewesksd
IPI00027834	552	551	unsp->unsp+DNAPK	unsp->unsp+DNAPK		3191	HNRNPL	rsssglleweSksdaletlgf
IPI00027834	552	553	CKII->PKA+CKII	CKII->PKA+CKII		3191	HNRNPL	ssgllewesk Sdaletlgfln
IPI00027970	55	59	unsp+PKC+cdc2->cdc2			54039	PCBP3	rllmhgkevgSiigkkgetvk
IPI00027987	53	49		unsp->		59082	CARD18	dmnkvrdendTvmdkarvlid
IPI00027987	64	68	unsp->			59082	CARD18	idlvtgkgpkScckfikhlce
IPI00027987	67	68	unsp->	unsp->		59082	CARD18	idlvtgkgpkScckfikhlce
IPI00027988	18	13		CKI->		10664	CTCF	gdaveaiveeSetfikgkerk
IPI00027988	18	15	->CKII			10664	CTCF	aveaiveeseTfikgkerkty
IPI00027988	18	25		INSR->		10664	CTCF	tfikgkerktYqrrreggqee
IPI00027988	20	25	INSR->	INSR->		10664	CTCF	tfikgkerktYqrrreggqee
IPI00027996	472	470	PKC+cdc2->cdc2	PKC+cdc2->cdc2		79585	CORO7	lrslqsllgpSskfrhaqgtv
IPI00027996	472	471		->DNAPK		79585	CORO7	rslqsllgpsSkfrhaqgtvl
IPI00028006	68	76	p38MAPK->cdc2+p38MAPK			5690	PSMB2	lykmrngyelSptaaanftrr
IPI00028031	285	286	unsp->	unsp->		37	ACADVL	tsavpspcgkYytlngsklwi
IPI00028031	285	287	INSR->unsp+INSR	INSR->unsp+INSR		37	ACADVL	savpspcgkyYtlngsklwis
IPI00028031	285	288	PKC->			37	ACADVL	avpspcgkyyTlngsklwisn
IPI00028031	285	292	->cdc2			37	ACADVL	pcgkyytlngSklwisnggla
IPI00028091	240	245	INSR+unsp->unsp			10096	ACTR3	cpdlvkefnkYdtdgskwikq
IPI00028091	251	250	PKC->DNAPK	PKC->ATM+DNAPK		10096	ACTR3	kefnkydtdgSkwikqytgin
IPI00028091	254	250	PKC->	PKC->		10096	ACTR3	kefnkydtdgSkwikqytgin
IPI00028127	8	12	unsp+PKC->unsp			2113	ETS1	kaavdlkptlTiiktekvdle
IPI00028127	15	10	->PKA			2113	ETS1	-mkaavdlkpTltiiktekvd
IPI00028127	15	12	unsp+PKC->			2113	ETS1	kaavdlkptlTiiktekvdle
IPI00028127	18	16	unsp+PKC->	unsp+PKC->		2113	ETS1	dlkptltiikTekvdlelfps
IPI00028127	18	26	->cdc2			2113	ETS1	tekvdlelfpSpdmecadvpl
IPI00028127	305	303	unsp+PKC+PKG->PKG	unsp+PKC+PKG->PKG		2113	ETS1	aalpnhkpkgTfkdyvrdrad
IPI00028127	305	307		->unsp		2113	ETS1	nhkpkgtfkdYvrdradlnkd
IPI00028414	119	126	unsp+CKII->unsp+PKA+CKII			9535	GMFG	eltkvfeirtTddlteawlqe
IPI00028520	81	82		->unsp		4723	NDUFV1	gpdwilgeikTsglrgrggag
IPI00028520	104	100	PKC->PKA	PKC->PKA		4723	NDUFV1	gagfptglkwSfmnkpsdgrp
IPI00028520	104	106	unsp+cdc2->PKC+cdc2			4723	NDUFV1	glkwsfmnkpSdgrpkylvvn
IPI00028888	251	244		INSR+unsp->unsp		3184	HNRNPD	epvkkimekkYhnvglskcei
IPI00028888	251	250	unsp+PKC->unsp	unsp+PKC->ATM+unsp		3184	HNRNPD	mekkyhnvglSkceikvamsk
IPI00028888	341	337		->unsp		3184	HNRNPD	ygygdysnqqSgygkvsrrgg
IPI00028888	341	339		EGFR->unsp+EGFR		3184	HNRNPD	ygdysnqqsgYgkvsrrgghq
IPI00028888	341	343	unsp->unsp+PKC			3184	HNRNPD	snqqsgygkvSrrgghqnsyk
IPI00028888	353	351	unsp+cdc2->cdc2	unsp+cdc2->cdc2		3184	HNRNPD	kvsrrgghqnSykpy------
IPI00028912	362	366	->CKII			7716	VEZF1	vtswpgkqveTlrlweeavka
IPI00028957	393	399	unsp->unsp+cdc2			9354	UBE4A	yqmlknllqlSpetkhcilsw
IPI00029012	68	64	unsp->unsp+PKA+RSK			8661	EIF3A	ylelcvdlrkShlakeglyqy
IPI00029073	69	73	unsp+PKC->unsp			26471	NUPR1	erklvtklqnSerkkrgarr-
IPI00029073	76	73	unsp+PKC->unsp	unsp+PKC->unsp		26471	NUPR1	erklvtklqnSerkkrgarr-
IPI00029073	77	73	unsp+PKC->unsp	unsp+PKC->unsp		26471	NUPR1	erklvtklqnSerkkrgarr-
IPI00029079	9	8		->ATM		8833	GMPS	---malcngdSklenaggdlk
IPI00029081	316	317		->unsp		3980	LIG3	vklllpgvikTvynlndkqiv
IPI00029133	221	226	PKA->	PKA->		515	ATP5F1	inwvekhvvqSistqqeketi
IPI00029196	384	390	PKC->PKC+PKA			5588	PRKCQ	filhkmlgkgSfgkvflaefk
IPI00029400	54	50	PKC->			9406	ZRANB2	teakmmkaggTeigktlaeks
IPI00029400	54	55		PKC->unsp+PKC		9406	ZRANB2	mkaggteigkTlaeksrglfs
IPI00029447	124	118		INSR->		9527	GOSR1	lqrhrdilqdYthefhktkan
IPI00029447	124	125		->unsp+PKC		9527	GOSR1	lqdythefhkTkanfmairer
IPI00029484	439	433	PKC+RSK->PKC+PKA+RSK	PKC+RSK->PKC+cdc2+RSK		9203	ZMYM3	sngsvvhrlcSdscfskfran

IPI00029484	439	438		PKC->		9203	ZMYM3	vhrlcsdscfSkfrankglkt
IPI00029485	230	233	unsp+PKC->PKC	unsp+PKC->PKC		1639	DCTN1	qvrdleekleTlrlkraedka
IPI00029534	81	75		CKII->		5471	PPAT	kgmglvnhvfTednlkklyvs
IPI00029534	81	83	INSR+unsp->unsp	INSR+unsp->unsp		5471	PPAT	vftednlkklYvsnlgightr
IPI00029534	81	85		->cdc2		5471	PPAT	tednlkklyvSnlgightrya
IPI00029561	242	241		->EGFR		4705	NDUFA10	saylqdienaYkktflpemse
IPI00029561	242	244	PKG->	PKG->		4705	NDUFA10	lqdienaykkTflpemsekce
IPI00029561	243	241		->unsp		4705	NDUFA10	saylqdienaYkktflpemse
IPI00029561	243	244		PKG->		4705	NDUFA10	lqdienaykkTflpemsekce
IPI00029601	144	141		->EGFR	Uniprot|PhosphoSite	2017	CTTN	rvdqsavgfeYqgktekhasq
IPI00029601	161	156	unsp->	unsp->		2017	CTTN	ekhasqkdysSgfggkygvqa
IPI00029601	198	191	INSR->	INSR->		2017	CTTN	ktekhesqrdYskgfggkygi
IPI00029601	198	192	unsp->unsp+PKA			2017	CTTN	tekhesqrdySkgfggkygid
IPI00029601	218	215		->EGFR	Uniprot|PhosphoSite	2017	CTTN	kvdksavgfeYqgktekhesq
IPI00029601	272	277	unsp+PKC->PKC	unsp+PKC->		2017	CTTN	tgfggkfgvqSerqdsaavgf
IPI00029601	304	303	unsp->	unsp->ATM+unsp+DNAPK	PhosphoSite	2017	CTTN	lakhesqqdySkgfggkygvq
IPI00029623	102	103	unsp->	unsp->		5687	PSMA6	raryeaanwkYkygyeipvdm
IPI00029623	104	103	unsp->			5687	PSMA6	raryeaanwkYkygyeipvdm
IPI00029623	104	107	unsp->	unsp->		5687	PSMA6	eaanwkykygYeipvdmlckr
IPI00029629	273	272		unsp+PKA->ATM+unsp+PKA+DNAPK		7706	TRIM25	kikeeekrvnSkfdtiyqill
IPI00029629	273	278	INSR+unsp+EGFR->unsp+EGFR	INSR+unsp+EGFR->INSR+unsp	ıiprot|PHOsphoELM|PhosphoSi	7706	TRIM25	krvnskfdtiYqillkkksei
IPI00029629	320	318	unsp+PKG+PKA->PKG+PKA	unsp+PKG+PKA->PKG+PKA		7706	TRIM25	lekasklrgiStkpvyipeve
IPI00029629	320	319	unsp+PKC->PKC+DNAPK	unsp+PKC->DNAPK		7706	TRIM25	ekasklrgisTkpvyipevel
IPI00029629	567	568		PKC->unsp+PKC		7706	TRIM25	isawhnnvekTlpstkatrvg
IPI00029631	12	11	unsp+PKC->unsp	unsp+PKC->unsp		2079	ERH	mshtillvqpTkrpegrtyad
IPI00029697	297	306		unsp+CKII->unsp+cdc2+CKII	ıiprot|PHOsphoELM|PhosphoSi	5393	EXOSC9	fkmekapidtSdveekaeeii
IPI00029731	8	7	PKG+PKA+RSK->PKA+RSK	PKG+PKA+RSK->ATM+PKG+PKA+DNAPK+RSK		6165	RPL35A	----msgrlwSkaifagykrg
IPI00029733	246	251	PKG+PKA+p38MAPK->p38MAPK			1645	AKR1C1	lcalakkhkrTpalialryql
IPI00029737	89	88	unsp+PKC->CKI+PKC	unsp+PKC->ATM+unsp		2182	ACSL4	tldklfdhavSkfgkkdslgt
IPI00029737	92	88	unsp+PKC->unsp	unsp+PKC->unsp		2182	ACSL4	tldklfdhavSkfgkkdslgt
IPI00029737	92	95		PKA->		2182	ACSL4	havskfgkkdSlgtreilsee
IPI00029744	103	99	SRC+unsp->unsp+EGFR	SRC+unsp->SRC+unsp+EGFR	PhosphoSite	6742	SSBP1	vfrpglrdvaYqyvkkgsriy
IPI00029744	103	101		->unsp		6742	SSBP1	rpglrdvayqYvkkgsriyle
IPI00029744	104	99	SRC+unsp->unsp	SRC+unsp->unsp	PhosphoSite	6742	SSBP1	vfrpglrdvaYqyvkkgsriy
IPI00029744	113	116	INSR+EGFR->	INSR+EGFR->		6742	SSBP1	sriylegkidYgeymdknnvr
IPI00029745	276	273	unsp->		PhosphoSite	4774	NFIA	rrslpstsstSstkrlksved
IPI00029745	276	275		unsp+PKC->unsp+PKC+DNAPK		4774	NFIA	slpststssTkrlksvedem
IPI00029750	37	34	PKC->			6229	RPS24	vidvlhpgkaTvpkteirekl
IPI00029764	489	492	unsp->		PhosphoSite	10946	SF3A3	ssgnvvnkktYedlkrqgll-
IPI00029795	280	276	PKC+cdc2->	PKC+cdc2->unsp		4782	NFIC	glrrtlpstsSsgskrhksgs
IPI00029795	280	277	unsp+PKC+cdc2->cdc2			4782	NFIC	lrrtlpstssSgskrhksgsm
IPI00029795	280	279		unsp+PKC->ATM+unsp+PKC+DNAPK		4782	NFIC	rtlpstsssgSkrhksgsmee
IPI00029997	180	178	unsp+GSK3+cdk5->unsp+GSK3+cdk5+p38MAPK	unsp+GSK3+cdk5->unsp+GSK3+cdk5+p38MAPK		25796	PGLS	rekivapisdSpkpppqrvtl
IPI00029997	180	189		PKA->		25796	PGLS	pkpppqrvtlTlpvlnaartv
IPI00030131	207	208	unsp+cdk5+RSK->unsp+cdk5+p38MAPK	unsp+cdk5+RSK->unsp+cdk5	Uniprot|PhosphoSite	7112	TMPO	kreplkgrakTpvtlkqrrve
IPI00030131	207	211	unsp+PKC->PKC	unsp+PKC->PKC	Uniprot|PhosphoSite	7112	TMPO	plkgraktpvTlkqrrvehnq
IPI00030179	161	159	unsp+EGFR->			1E+08	RPL7P32	svneliykrgYgkinkkrial
IPI00030247	390	388	unsp+PKC+PKG->unsp+PKG+PKA	unsp+PKC+PKG->unsp+PKG+PKA		904	CCNT1	nsksvpsakvSlkeyrakhae
IPI00030274	499	490	unsp+PKG+CKII->unsp+PKG+cdc2+CKII			84081	CCDC55	kdkernqekpSnsesslgakh
IPI00030274	499	492	unsp->unsp+cdc2			84081	CCDC55	kernqekpsnSesslgakhrl
IPI00030274	499	495	unsp+PKC->unsp	unsp+PKC->unsp		84081	CCDC55	nqekpsnsesSlgakhrltee
IPI00030274	499	503	unsp->CKI	unsp->CKI		84081	CCDC55	esslgakhrlTeegqekgkeq
IPI00030275	87	84	unsp+PKC->unsp	unsp+PKC->unsp		10131	TRAP1	iisstesvqgStskhefqaet
IPI00030275	87	85	unsp+PKC->	unsp+PKC->		10131	TRAP1	isstesvqgsTskhefqaetk
IPI00030275	87	86	unsp+CKII->unsp	unsp+CKII->ATM+unsp+CKII+DNAPK		10131	TRAP1	sstesvqgstSkhefqaetkk
IPI00030275	332	328	PKC->PKA	PKC->PKA		10131	TRAP1	vaqahdkpryTlhyktdapln
IPI00030275	332	333	->CKII	->CKII		10131	TRAP1	dkprytlhykTdaplnirsif
IPI00030275	382	377	PKC->			10131	TRAP1	srkvliqtkaTdilpkwlrfi
IPI00030275	431	430		unsp+PKC->ATM+unsp+PKC+DNAPK		10131	TRAP1	qrlikffidqSkkdaekyakf
IPI00030275	432	430	unsp+PKC->unsp+CKII	unsp+PKC->unsp		10131	TRAP1	qrlikffidqSkkdaekyakf
IPI00030275	466	470	unsp->	unsp->		10131	TRAP1	vkediakllrYessalpsgql
IPI00030363	83	90		INSR+unsp->unsp		38	ACAT1	gipkeevkeaYmgnvlqggeg
IPI00030363	124	120	PKC->	PKC->		38	ACAT1	gaglpistpcTtinkvcasgm
IPI00030363	124	128	PKC->			38	ACAT1	pcttinkvcaSgmkaimmasq
IPI00030363	181	185	PKC->			38	ACAT1	ledlivkdglTdvynkihmgs
IPI00030363	181	188		INSR->		38	ACAT1	livkdgltdvYnkihmgscae
IPI00030363	190	185	PKC->	PKC->		38	ACAT1	ledlivkdglTdvynkihmgs
IPI00030363	190	188	INSR->			38	ACAT1	livkdgltdvYnkihmgscae
IPI00030363	202	200	unsp->	unsp->		38	ACAT1	kihmgscaenTakklniarne
IPI00030363	251	256		SRC+unsp+EGFR->SRC+unsp		38	ACAT1	pdvvvkedeeYkrvdfskvpk
IPI00030363	263	262	PKC->	PKC->ATM+DNAPK		38	ACAT1	edeeykrvdfSkvpklktvfq
IPI00030702	343	340	unsp->unsp+PKA	unsp->unsp+PKA		3419	IDH3A	acfatikdgkSltkdlggnak
IPI00030702	343	342		->ATM+DNAPK		3419	IDH3A	fatikdgkslTkdlggnakcs
IPI00030706	212	210	unsp+PKG->PKG	unsp+PKG->PKG		10598	AHSA1	vgvkiptckiTlketfltspe
IPI00030915	601	599	unsp+PKC->PKC	unsp+PKC->PKC		9101	USP8	phtsvtgdsgSgkpfkikgqp
IPI00031023	21	16	unsp+PKC->unsp	unsp+PKC->unsp		2314	FLII	vlpfvrgvdlSgndfkggyfp
IPI00031489	58	54		unsp->INSR+unsp		10289	EIF1B	lttvqgiaddYdkkklvkafk
IPI00031519	235	227	unsp+PKC->unsp+PKC+PKA			1786	DNMT1	pritrkstrqTtitshfakgp
IPI00031519	235	231	PKC->	PKC->		1786	DNMT1	rkstrqttitShfakgpakrk
IPI00031519	1173	1167	unsp+GSK3+cdk5->unsp+GSK3		Uniprot|PHOsphoELM|PhosphoSi	1786	DNMT1	sfedppnharSpgnkgkgkgk
IPI00031522	303	298	->INSR			3030	HADHA	ekvrkqtkglYpaplkiidvv
IPI00031522	309	310		CKII->unsp+CKII		3030	HADHA	aplkiidvvkTgieqgsdagy
IPI00031522	326	324	ATM+unsp+cdc2+DNAPK->ATM+cdc2+DNAPK	ATM+unsp+cdc2+DNAPK->ATM+cdc2+DNAPK		3030	HADHA	qgsdagylceSqkfgelvmtk
IPI00031522	569	573	unsp+PKA->CKI+PKA	unsp+PKA->unsp		3030	HADHA	qegvdpkkldSlttsfgfpvg
IPI00031522	570	573	unsp+PKA->PKA	unsp+PKA->		3030	HADHA	qegvdpkkldSlttsfgfpvg
IPI00031522	644	637	INSR+EGFR->EGFR	INSR+EGFR->EGFR		3030	HADHA	flgrksgkgfYiyqegvkrkd
IPI00031522	728	724		->EGFR		3030	HADHA	lggpfrfvdlYgaqkivdrlk
IPI00031522	728	736	->INSR	->INSR		3030	HADHA	aqkivdrlkkYeaaygkqftp
IPI00031526	146	145		unsp+CKI->ATM+unsp+CKI		79002	C19orf43	kqktedevltSkgdawakyma
IPI00031545	608	611	unsp->CKII	unsp->unsp+CKII		3709	ITPR2	nnrkllekhiTakeietfvsl
IPI00031556	462	461		PKC->DNAPK		11338	U2AF2	grkfanrvvvTkycdpdsyhr
IPI00031562	6	2	PKC->		Uniprot	92815	HIST3H2A	---------mSgrgkqggkar
IPI00031570	21	16	cdc2->			84284	C1orf57	fltgppgvgkTtlihkasevl
IPI00031801	96	94	unsp+PKC->PKC	unsp+PKC->PKC		8531	CSDA	kkvlatkvlgTvkwfnvrngy
IPI00031812	64	62	unsp+PKC->PKC	unsp+PKC->PKC		4904	YBX1	kkviatkvlgTvkwfnvrngy
IPI00031812	81	80		unsp->unsp+DNAPK		4904	YBX1	ngygfinrndTkedvfvhqta
IPI00031820	349	346	unsp+p38MAPK->p38MAPK	unsp+p38MAPK->p38MAPK		2193	FARSA	lyrlaqkkpfTpvkyfsidrv
IPI00031820	349	352	unsp->	unsp->		2193	FARSA	kkpftpvkyfSidrvfrnetl
IPI00032164	69	62		INSR->		8048	CSRP3	heseiyckvcYgrrygpkgig
IPI00032214	368	371	unsp+CKI->CKI			23774	BRD1	ymkmepvkelTggttfsvrk
IPI00032214	418	415	unsp+PKG+cdc2+PKA+RSK->unsp+PKG+PKA	unsp+PKG+cdc2+PKA+RSK->unsp+PKG+PKA		23774	BRD1	emkngvcrkeSsvktvrstsk
IPI00032214	418	416	unsp+PKC->	unsp+PKC->		23774	BRD1	mkngvcrkesSvktvrstskv
IPI00032214	418	422	PKG->	PKG->		23774	BRD1	rkessvktvrStskvrkkakk
IPI00032214	519	524	unsp->unsp+EGFR			23774	BRD1	emkaakeklkYwqrlrhdler
IPI00032230	405	401	PKC->PKA	PKC->		23136	EPB41L3	lpeappkkflTlgskfrysgr
IPI00032230	405	404		PKC->PKC+DNAPK		23136	EPB41L3	appkkfltlgSkfrysgrtqa
IPI00032230	405	409		unsp+PKA->unsp+cdc2		23136	EPB41L3	fltlgskfrySgrtqaqtrra
IPI00032313	18	20	unsp+PKG+CKII->unsp+CKI+CKII	unsp+PKG+CKII->unsp+CKI+CKII		6275	S100A4	dvmvstfhkySgkegdkfkln
IPI00032313	22	20	unsp+PKG+CKII->unsp+PKG+PKA+CKII	unsp+PKG+CKII->unsp+PKG+PKA+CKII		6275	S100A4	dvmvstfhkySgkegdkfkln
IPI00032313	35	32	->CKII	->CKII		6275	S100A4	kegdkfklnkSelkelltrel
IPI00032316	528	526	unsp->cdc2	unsp->		23660	ZKSCAN5	ipmkeilgqpSskrmnysevp
IPI00032316	528	527	unsp+PKC->PKC	unsp+PKC->ATM+unsp+PKC+DNAPK		23660	ZKSCAN5	pmkeilgqpsSkrmnysevpy
IPI00032355	830	833	unsp->	unsp->		9698	PUM1	aapgaeakyrSassasslfsp
IPI00032355	830	835	unsp+PKC+PKA->unsp+PKA	unsp+PKC+PKA->unsp+cdc2+PKA		9698	PUM1	pgaeakyrsaSsasslfspss
IPI00032358	714	710	PKC->	PKC->		1E+08	POM121C	saspmfkpifTappksekegp
IPI00032358	714	715	unsp+CKII->unsp+CKI+CKII			1E+08	POM121C	fkpiftappkSekegptppgp
IPI00032598	388	386	unsp->unsp+CKII			2117	ETV3	ipprikvepaSekdpeslrqs
IPI00032598	388	392	unsp+PKC->unsp			2117	ETV3	vepasekdpeSlrqsarekee
IPI00032826	153	156	unsp+PKC+PKA->PKC+PKA	unsp+PKC+PKA->PKC+PKA		6767	ST13	rlailyakraSvfvklqkpna
IPI00032827	29	31	PKG+CKII->CKII	PKG+CKII->CKII		51639	SF3B14	lyirnlpykiTaeemydifgk
IPI00032831	124	130	unsp+p38MAPK->unsp			9342	SNAP29	vnyfkskpveTppeqngtlts
IPI00032875	153	151	PKC->CKII	PKC->CKII		2110	ETFDH	pvtedrfgilTekyripvpil
IPI00032892	25	29		->cdc2		9453	GGPS1	llqlpgkqvrTklsqafnhwl
IPI00032904	6	9	PKC->			6620	SNCB	--mdvfmkglSmakegvvaaa
IPI00032904	12	9	PKC->PKA	PKC->		6620	SNCB	--mdvfmkglSmakegvvaaa
IPI00032955	102	101	unsp+PKC->PKC			55905	RNF114	chgcrknffISkirshvatcs
IPI00032955	102	105	unsp->	unsp->		55905	RNF114	rknfflskirShvatcskyqn
IPI00032955	112	109	PKC->			55905	RNF114	flskirshvaTcskyqnyime

IPI00032955	112	111	PKC->PKC+DNAPK	PKC->ATM+DNAPK		55905	RNF114	skirshvatcSkyqnyimegv
IPI00032955	112	113	unsp->	unsp->		55905	RNF114	irshvatcskYqnyimegvka
IPI00032957	65	68	INSR+unsp+EGFR->INSR+unsp	INSR+unsp+EGFR->INSR+unsp		7329	UBE2I	fklrmlfkddYpssppkckfe
IPI00032957	65	71	GSK3+cdk5->GSK3+cdk5+cdc2			7329	UBE2I	rmlfkddypsSppkckfeppl
IPI00033016	158	151	->cdc2			4609	MYC	kniiiqdcmwSgfsaaaklvs
IPI00033016	158	154	PKC->	PKC->		4609	MYC	iiqdcmwsgfSaaaklvsekl
IPI00033016	158	161	unsp+PKC->	unsp+PKC->PKC		4609	MYC	sgfsaaaklvSeklasyqaar
IPI00033016	163	161	unsp+PKC->	unsp+PKC->		4609	MYC	sgfsaaaklvSeklasyqaar
IPI00033016	163	166	unsp->	unsp->		4609	MYC	aaklvseklaSyqaarkdsgs
IPI00033016	172	174	unsp+PKG+PKA->unsp+PKA			4609	MYC	lasyqaarkdSgspnparghs
IPI00033016	172	176		unsp->unsp+p38MAPK		4609	MYC	syqaarkdsgSpnparghsvc
IPI00033016	290	294	unsp+PKA->unsp	unsp+PKA->unsp		4609	MYC	krqapgkrseSgspsagghsk
IPI00033016	332	329	unsp+PKC->	unsp+PKC->unsp		4609	MYC	thqhnyaappStrkdypaakr
IPI00033016	332	330	unsp+PKC->	unsp+PKC->		4609	MYC	hqhnyaappsTrkdypaakrv
IPI00033016	338	334	->INSR	->INSR+EGFR		4609	MYC	yaappstrkdYpaakrvklds
IPI00033030	21	19	unsp+PKA+RSK->PKA+RSK	unsp+PKA+RSK->PKA+RSK		11047	ADRM1	pslvpgsrgaSnkylvefrag
IPI00033030	21	22	INSR->			11047	ADRM1	vpgsrgasnkYlvefragkms
IPI00033036	427	426		PKA->ATM+DNAPK		10988	METAP2	crrwldrlgeSkylmalknlc
IPI00033054	26	28	unsp+PKA+RSK->unsp+RSK	unsp+PKA+RSK->unsp+RSK	Uniprot	51496	CTDSPL2	qrtarakrkySevddslpsgg
IPI00033054	26	33	unsp->unsp+cdc2			51496	CTDSPL2	akrkysevddSlpsggekpsk
IPI00033054	57	59	PKC+PKA->PKC	PKC+PKA->PKC		51496	CTDSPL2	lssikkfikgStpkeerenps
IPI00033217	707	705	unsp->unsp+PKA	unsp->unsp+PKA		10157	AASS	lnlegypnrdStkyaeiygis
IPI00033217	707	706	unsp->unsp+PKA+DNAPK	unsp->ATM+unsp+DNAPK		10157	AASS	nlegypnrdsTkyaeiygiss
IPI00033486	288	290	->unsp	->unsp		11232	POLG2	deegrkgnklYynfpwgkeli
IPI00033486	288	291	INSR->	INSR->		11232	POLG2	eegrkgnklyYnfpwgkelie
IPI00033487	11	10	unsp+PKC->PKC	unsp+PKC->ATM+unsp+PKC+DNAPK		338917	VSX2	-mtgkagealSkpksetvaks
IPI00034308	884	888	unsp->	unsp->		1757	SARDH	vsldfvksgdYalermgvtyg
IPI00036578	1014	1009	->PKA	->PKA		81792	ADAMTS12	srrvlkpnkgTisngknpptl
IPI00036578	1014	1011	PKC->			81792	ADAMTS12	rvlkpnkgtiSngknpptlkp
IPI00036578	1014	1018	unsp+PKC->			81792	ADAMTS12	gtisngknppTlkpvppptsr
IPI00043429	165	170	PKG+PKA->PKA			148534	TMEM56	ffealkypkfSkaivingilm
IPI00044461	183	179	unsp+PKC+cdc2->unsp+cdc2	unsp+PKC+cdc2->unsp+cdc2		79258	MMEL1	rtlyrscmnqSviekrgsqpl
IPI00044666	376	380	unsp+PKC+CKII->PKC+CKII	unsp+PKC+CKII->PKC		130540	ALS2CR12	ivgnlekmlqTkfaeteekyk
IPI00044761	131	127	PKC->			54517	PUS7	tkfvsshqgfSgilkerysdf
IPI00044761	131	134	unsp->	unsp->		54517	PUS7	qgfsgilkerYsdfvvheigk
IPI00044761	131	135		PKA->		54517	PUS7	gfsgilkerySdfvvheigkd
IPI00045109	6	2	PKC->		Uniprot	221613	HIST1H2AA	---------mSgrgkqggkar
IPI00045914	1050	1049		unsp+PKC+PKA->unsp+PKC+DNAPK		23013	SPEN	pvrkeilkreSkkikldrlnt
IPI00045946	237	234	cdc2->	cdc2->		10730	YME1L1	rlaetqniapSfvkgfllrdr
IPI00046828	100	107	unsp+PKC->unsp+PKC+cdc2			131076	CCDC58	tllkqlrkeqTklkwmqseln
IPI00059242	70	75	unsp+PKA->unsp			94056	SYAP1	asaatkkiteSvaetaqtikk
IPI00059242	191	182	->cdc2			94056	SYAP1	lvmlqedellSkmrfalvpkl
IPI00059279	9	10	INSR->			60412	EXOC4	-maaeaaggkYrstvskskdp
IPI00059279	9	12	PKC->			60412	EXOC4	aaeaaggkyrStvskskdpsg
IPI00059292	116	115	CKII->CKII+DNAPK	CKII->CKII+DNAPK		55110	MAGOHB	kigslidvnqSkdpeglrvfy
IPI00060181	233	237	PKC->			79180	EFHD2	qrkaafkelqStfk-------
IPI00061009	403	401	unsp+PKC->CKII	unsp+PKC->		93594	WDR67	lkqdltgdfeSkknelpdgln
IPI00061206	111	116	unsp+PKA->	unsp+PKA->		140886	PABPC5	gnifiknldkSidnralfylf
IPI00061525	152	154	PKA->	PKA->		64841	GNPNAT1	lskklncykiTleclpqnvgf
IPI00062037	5	2	PKC->			140735	DYNLL2	---------mSdrkaviknad
IPI00063130	149	150	unsp->SRC+unsp			374882	TMEM205	yrqlrekdpkYsalrqnffry
IPI00063130	149	151	PKG+cdc2->cdc2	PKG+cdc2->cdc2		374882	TMEM205	rqlrekdpkySalrqnffryh
IPI00063635	292	285	unsp->unsp+cdc2			57325	CSRP2BP	qkeaagfldrStsstpvkfis
IPI00063635	292	287	unsp+RSK->unsp	unsp+RSK->		57325	CSRP2BP	eaagfldrstSstpvkfisrg
IPI00063635	292	288		PKC->unsp+PKC		57325	CSRP2BP	aagfldrstsStpvkfisrgr
IPI00063635	292	289	unsp+cdk5->unsp+cdk5+p38MAPK			57325	CSRP2BP	agfldrstssTpvkfisrgrr
IPI00063635	292	295	->cdc2	->cdc2		57325	CSRP2BP	stsstpvkfiSrgrrpdvile
IPI00063635	572	570	unsp+PKG->	unsp+PKG->		57325	CSRP2BP	lpsrkgfrhqTtkflyrlvgs
IPI00063903	16	22	INSR->	INSR->	PhosphoSite	84833	USMG5	ftgikkyfnsYtltgrmncvl
IPI00063903	17	13	PKC+cdc2->PKC			84833	USMG5	gpesdaqyqfTgikkyfnsyt
IPI00063903	17	21		PKA->		84833	USMG5	qftgikkyfnSytltgrmncv
IPI00063903	17	22	INSR->	INSR->	PhosphoSite	84833	USMG5	ftgikkyfnsYtltgrmncvl
IPI00063903	17	23	->CKI			84833	USMG5	tgikkyfnsyTltgrmncvla
IPI00064162	408	404	unsp->unsp+PKA			80124	VCPIP1	dggcviggdrSlqdkyllrlv
IPI00064202	599	598		->DNAPK		55534	MAML3	kqhniltygnTkplthfnadl
IPI00064212	490	483		->PKA		283489	ZNF828	rggspdlwksSffiepqkpvf
IPI00064212	617	615	unsp+PKC->	unsp+PKC->	PhosphoSite	283489	ZNF828	kklledtlfpSskklkkdnqe
IPI00064212	617	616		unsp+PKC->ATM+unsp+PKC+DNAPK	Uniprot	283489	ZNF828	klledtlfpsSkklkkdnqes
IPI00064457	928	924	unsp+PKC->unsp	unsp+PKC->		84875	PARP10	fnrsfcgrnaTvygkgvyfar
IPI00064765	208	202	->PKA	->PKA		140801	RPL10L	pdgcgvkyvpShgpldkwrvl
IPI00065500	283	281	SRC+unsp->SRC			148362	C1orf58	irslqeaeklYakaealckey
IPI00068506	304	305	unsp->	unsp->		10061	ABCF2	iihmhnkklkYytgnydqyvk
IPI00069084	2543	2540	PKC->	PKC->		8295	TRRAP	shdraafamvThvkqeprere
IPI00069750	454	445	PKC->PKC+cdc2			22827	PUF60	seqehmsisgSsarhmvmqkl
IPI00069817	416	421	unsp+PKC->PKC	unsp+PKC->PKC+cdc2		9031	BAZ1B	ilngqkstgnSkspkkglktp
IPI00069817	426	421		unsp+PKC->PKC		9031	BAZ1B	ilngqkstgnSkspkkglktp
IPI00069817	426	423	unsp+cdk5->unsp	unsp+cdk5->unsp		9031	BAZ1B	ngqkstgnskSpkkglktpkt
IPI00069817	426	430	unsp+PKC->unsp	unsp+PKC->unsp+PKC+p38MAPK		9031	BAZ1B	nskspkkglkTpktkmkqmtl
IPI00069817	1335	1338	unsp+PKC+PKA->unsp+PKC	unsp+PKC+PKA->unsp+PKC		9031	BAZ1B	delvlqtkrsSrrqslelqkc
IPI00070943	930	931	INSR+unsp->INSR			5297	PI4KA	hkrirrvadkYlsglvdkfph
IPI00070943	930	933	PKC+cdc2->cdc2			5297	PI4KA	rirrvadkylSglvdkfphll
IPI00072377	132	126	unsp+CKI+CKII->unsp+CKII	unsp+CKI+CKII->unsp+CKII		6418	SET	alhyltrvevTefediksgyr
IPI00072377	132	140	->INSR	->INSR		6418	SET	diksgyridfYfdenpyfenk
IPI00072377	150	146	SRC+unsp->unsp		Uniprot|PhosphoSite	6418	SET	ridfyfdenpYfenkvlskef
IPI00072377	172	166	unsp+CKI->unsp	unsp+CKI->unsp		6418	SET	fhlnesgdpsSksteikwksg
IPI00072377	172	169	PKC->			6418	SET	nesgdpssksTeikwksgkdl
IPI00072534	70	74	unsp+EGFR->			55898	UNC45A	raachlkledYdkaeteaska
IPI00072534	483	480	->PKA	->PKA		55898	UNC45A	rasfitangvSllkdlykcse
IPI00072534	483	486	unsp->	unsp->		55898	UNC45A	angvsllkdlYkcsekdsiri
IPI00072534	487	480		->PKA		55898	UNC45A	rasfitangvSllkdlykcse
IPI00072534	487	489	unsp->unsp+PKC			55898	UNC45A	vsllkdlykcSekdsiriral
IPI00074876	843	836	->cdc2			51230	PHF20	lprsveesyiTsehcyqkpra
IPI00074876	843	837		CKII->		51230	PHF20	prsveesyitSehcyqkpray
IPI00075248	22	18	unsp+CKI->unsp+CKI+PKA			808	CALM3	eqiaefkeafSlfdkdgdgti
IPI00075248	76	71	PKC->	PKC->		808	CALM3	ngtidfpeflTmmarkmkdtd
IPI00075248	76	82	unsp+CKII->unsp+CKI+CKII		PhosphoSite	808	CALM3	mmarkmkdtdSeeeireafrv
IPI00075248	116	118	unsp+CKII->CKI+CKII	unsp+CKII->unsp+CKI+CKII	PhosphoSite	808	CALM3	hvmtnlgeklTdeevdemire
IPI00075248	149	147	PKC->	PKC->		808	CALM3	vnyeefvqmmTak--------
IPI00081097	163	165	->PKC			147407	MCART2	lqnhkhhdkfTntyqafkalk
IPI00081097	163	168	INSR->	INSR->		147407	MCART2	hkhhdkftntYqafkalkchg
IPI00081836	6	2	PKC->		Uniprot	8336	HIST1H2AN	---------mSgrgkqggkar
IPI00083708	27	19	->PKA			23215	BAT2L2	tkakdgkkyaTlslfntykgk
IPI00083708	27	25	unsp+PKC->	unsp+PKC->		23215	BAT2L2	kkyatlslfnTykgksletqk
IPI00083708	27	30	unsp+PKA->unsp+CKI	unsp+PKA->unsp		23215	BAT2L2	lslfntykgkSletqkttvaa
IPI00083708	49	46	CKI+PKC->PKC+PKA	CKI+PKC->CKI+PKC+PKA		23215	BAT2L2	ttvaarhglqSlgkvgisrrm
IPI00083708	49	53	unsp+PKC+cdc2->unsp+cdc2	unsp+PKC+cdc2->unsp+cdc2		23215	BAT2L2	glqslgkvgiSrrmpppanlp
IPI00083708	394	392	INSR->	INSR->unsp		23215	BAT2L2	paqpsvakvpYgkgpsfnqer
IPI00084571	126	122		->unsp		54148	MRPL39	pwdmykpltkSceikfltfkd
IPI00084571	126	129	unsp+PKC->PKC			54148	MRPL39	ltksceikflTfkdcdpgevn
IPI00084571	204	203		->DNAPK		54148	MRPL39	mptkenlrsfTkdahaliykd
IPI00084571	234	233	unsp->PKA+DNAPK	unsp->unsp+DNAPK		54148	MRPL39	akvaleifqhSkykvdfieek
IPI00084571	236	233	unsp->PKA			54148	MRPL39	akvaleifqhSkykvdfieek
IPI00093057	371	376	unsp->	unsp->		1371	CPOX	iplvkkhcddSftpqeklwqq
IPI00093057	404	403	unsp+CKI->unsp+CKI+PKA	unsp+CKI->unsp+CKI+DNAPK		1371	CPOX	vefnllydrgTkfglftpgsr
IPI00096066	291	292	unsp->	unsp->		8801	SUCLG2	nepieneaakYdlkyigldgn
IPI00096066	338	343	->EGFR			8801	SUCLG2	lgggvkeaqvYqafklltadp
IPI00098902	970	966	unsp->	unsp->		4967	OGDH	cqeehknqgyYdyvkprlrtt
IPI00098902	970	975	PKC+PKG->PKG	PKC+PKG->PKG		4967	OGDH	yydyvkprlrTtisrakpvwy
IPI00099179	303	301	unsp+PKC+PKG+PKA+RSK->unsp+PKA+RSK	unsp+PKC+PKG+PKA+RSK->unsp+PKA+RSK		51684	SUFU	icigtqprrlSgkdteqiret
IPI00099433	440	445	unsp+cdk5+p38MAPK->cdk5+p38MAPK	unsp+cdk5+p38MAPK->GSK3+cdk5+p38MAPK	PhosphoSite	23522	MYST4	idgltkfftpSpdgrrsrgei
IPI00099433	584	578	unsp+CKI+PKC->unsp+PKC	unsp+CKI+PKC->unsp+PKC		23522	MYST4	pkrmrrktelSstakskahff
IPI00099433	584	579	unsp+PKC->PKC	unsp+PKC->PKC		23522	MYST4	krmrrktelsStakskahffg
IPI00099433	584	580	PKC+cdc2->	PKC+cdc2->cdc2		23522	MYST4	rmrrktelssTakskahffgk
IPI00099433	584	583	unsp+PKC->	unsp+PKC->ATM+unsp+DNAPK		23522	MYST4	rktelsstakSkahffgkrdi
IPI00099433	1038	1032	unsp+cdc2->unsp+PKA+cdc2	unsp+cdc2->unsp+PKA+cdc2		23522	MYST4	eqeilstranSrqspakvqsk
IPI00099433	1038	1041	->PKG+cdc2			23522	MYST4	nsrqspakvqSknkylhspes
IPI00099433	1042	1041		->DNAPK		23522	MYST4	nsrqspakvqSknkylhspes
IPI00099463	353	351	unsp+PKC->	unsp+PKC->		8879	SGPL1	vkgvtsisadThkygyapkgs

Table S3

IPI00099463	353	354	unsp->SRC	unsp->SRC		8879	SGPL1	vtsisadthkYgyapkgsslv
IPI00099463	353	356	unsp->	unsp->		8879	SGPL1	sisadthkygYapkgsslvly
IPI00099463	353	362	->PKA			8879	SGPL1	hkygyapkgsSlvlysdkkyr
IPI00099834	222	218	unsp+PKC+PKG+PKA->unsp+PKA			29855	UBN1	tydkekkskkSkfskagftal
IPI00099834	222	221		unsp+PKC+PKA->unsp+PKC+DNAPK		29855	UBN1	kekkskkskfSkagftalnas
IPI00100160	971	979	unsp+PKC->unsp+PKC+cdc2			55832	CAND1	rlkgylisgsSyarssvvtav
IPI00100460	235	230		unsp->		55157	DARS2	pggakeflvpSrepgkfyslp
IPI00100460	235	238	PKA->cdc2	PKA->cdc2		55157	DARS2	vpsrepgkfySlpqspqqfkq
IPI00100460	382	378		->EGFR		55157	DARS2	kaicipegakYlkrkdiesir
IPI00100460	382	386	unsp+PKC->unsp			55157	DARS2	akylkrkdieSirnfaadhfn
IPI00100630	333	336	PKC->			4298	MLLT1	dkkpakdkssTrgekvkaese
IPI00100656	116	120		PKC->PKC+cdc2		9524	TECR	pfiyghkydfTssrhtvvhla
IPI00100656	116	121	unsp+PKC->unsp	unsp+PKC->unsp		9524	TECR	fiyghkydftSsrhtvvhlac
IPI00100731	69	65	unsp->unsp+PKA			63898	SH2D4A	kprpkkengkSvhwklgadke
IPI00100980	324	320	unsp->unsp+PKA			30846	EHD2	iisylkkempSvfgkenkkkq
IPI00101968	176	178	->PKC		Uniprot|PhosphoSite	28988	DBNL	vseikrvgkdSfwakaekeee
IPI00101968	297	300	unsp+DNAPK->ATM+DNAPK	unsp+DNAPK->ATM+unsp+DNAPK	iniprot|PHOsphoELM|PhosphoSi	28988	DBNL	lrspflqkqlTqpethfgrep
IPI00102069	254	251	PKC->	PKC->		10480	EIF3M	ltifvsaklaSyvkfyqnnkd
IPI00102165	6	2	PKC->		Uniprot	55766	H2AFJ	---------mSgrgkqggkvr
IPI00102339	58	63	unsp->	unsp->		4654	MYOD1	vgallkpeehShfpaavhpap
IPI00102339	102	105	PKC->			4654	MYOD1	lwackackrkTtnadrrkaat
IPI00102339	102	106	unsp+PKG->PKG	unsp+PKG->PKG		4654	MYOD1	wackackrktTnadrrkaatm
IPI00102339	104	105		PKC->unsp+PKC		4654	MYOD1	lwackackrkTtnadrrkaat
IPI00102580	229	232	EGFR->			66036	MTMR9	natlragkrgYiidtrslnva
IPI00102580	229	236	->cdc2			66036	MTMR9	ragkrgyiidTrslnvaqqtr
IPI00102580	229	238	PKC->PKC+cdc2	PKC->PKC+cdc2		66036	MTMR9	gkrgyiidtrSlnvaqqtrak
IPI00102752	450	448	INSR+EGFR->EGFR			64783	RBM15	iiirnpikigYgkatpttrlw
IPI00102752	450	452	p38MAPK->cdk5+p38MAPK	p38MAPK->unsp+cdk5+p38MAPK		64783	RBM15	npikigygkaTpttrlwvggl
IPI00102752	450	454	unsp+PKC->PKC			64783	RBM15	ikigygkatpTtrlwvgglgp
IPI00102815	23	20	unsp+cdc2+PKA->PKA	unsp+cdc2+PKA->unsp+PKA		64318	NOC3L	ipsfrkliktSkvklenklkn
IPI00102815	34	37	PKC->			64318	NOC3L	klknkqfkqqStlkkyrkeqr
IPI00102815	34	38	unsp+PKC->PKC			64318	NOC3L	lknkqfkqqsTlkkyrkeqrk
IPI00102815	40	37	PKC->	PKC->		64318	NOC3L	klknkqfkqqStlkkyrkeqr
IPI00102815	40	38	unsp+PKC->PKC	unsp+PKC->PKC		64318	NOC3L	lknkqfkqqsTlkkyrkeqrk
IPI00103023	298	295	PKC->CKII			26190	FBXW2	lhspgdyillSadkyeikiwp
IPI00103483	519	518	unsp+PKG+cdc2->unsp+PKA+cdc2	unsp+PKG+cdc2->ATM+unsp		25920	COBRA1	lihlhprvapSklealqkale
IPI00104050	221	217	unsp->unsp+RSK	unsp->unsp+RSK		9967	THRAP3	sggtsqdtkaSesskpwpdat
IPI00104050	221	219	unsp+PKC->unsp+CKII+cdc2	unsp+PKC->unsp		9967	THRAP3	gtsqdtkaseSskpwpdatyg
IPI00104050	221	220	unsp+CKI->unsp+CKI+PKA+DNAPK	unsp+CKI->ATM+unsp+CKI+DNAPK		9967	THRAP3	tsqdtkasesSkpwpdatygt
IPI00104050	221	227	->cdc2	->cdc2		9967	THRAP3	sesskpwpdaTygtgsasras
IPI00104050	401	392	PKC->PKC+cdc2			9967	THRAP3	glgdgkmksdSfapktdsekp
IPI00104050	401	399	unsp+PKC->	unsp+PKC->		9967	THRAP3	ksdsfapktdSekpfrgsqsp
IPI00104050	401	406	ATM+unsp+PKC+cdc2+PKA+DNAPK->ATM+unsp+cdc2+DN	ATM+unsp+PKC+cdc2+PKA+DNAPK->ATM+unsp+PKC	iniprot|PHOsphoELM|PhosphoSi	9967	THRAP3	ktdsekpfrgSqspkryklrd
IPI00104050	455	454	->PKA	->DNAPK		9967	THRAP3	sefddepkfmSkviganknqe
IPI00104050	519	524		unsp+PKC->unsp		9967	THRAP3	gfvpeknfrvTaykavqekss
IPI00104050	709	704	SRC+unsp->unsp			9967	THRAP3	demksprepgYkaegkykddp
IPI00104050	811	805	unsp+CKI+cdc2->unsp+CKI+PKA+cdc2	unsp+CKI+cdc2->unsp+CKI+PKA+cdc2		9967	THRAP3	yteeteereeSttgfdksrlg
IPI00104050	811	807		unsp+CKII->CKII		9967	THRAP3	eeteereestTgfdksrlgtk
IPI00104907	113	110	unsp+GSK3+cdk5->GSK3	unsp+GSK3+cdk5->unsp+GSK3	Uniprot|PhosphoSite	55320	C14orf106	dglknkanyeSpgkiflrmke
IPI00105407	95	96	unsp->unsp+PKC	unsp->unsp+PKC		57016	AKR1B10	rplvrkafekTlkdlklsyld
IPI00105407	95	103	unsp+PKA->unsp			57016	AKR1B10	fektlkdlklSyldvylihwp
IPI00105407	98	96	unsp->cdc2	unsp->		57016	AKR1B10	rplvrkafekTlkdlklsyld
IPI00105407	98	103	unsp+PKA->unsp	unsp+PKA->unsp+PKG		57016	AKR1B10	fektlkdlklSyldvylihwp
IPI00105407	179	178	unsp->	unsp->		57016	AKR1B10	ekllnkpglkYkpvtnqvech
IPI00105407	263	266	p38MAPK->			57016	AKR1B10	rnvivipksvTpariveniqv
IPI00105598	418	414	unsp->unsp+PKA	unsp->unsp+PKA		5717	PSMD11	tiqnmskvvdSlynkakklt-
IPI00105598	418	416	EGFR->			5717	PSMD11	qnmskvvdslYnkakklt---
IPI00105598	418	423	PKA->			5717	PSMD11	dslynkakklT----------
IPI00106567	46	42	PKC->	PKC->		55339	WDR33	faqqqamqqlTfdgkrmrkav
IPI00107722	114	106	unsp->unsp+PKA	unsp->unsp+PKA		84693	MCEE	mellhplgrdSpiagflqknk
IPI00107745	78	73	unsp+PKC->	unsp+PKC->PKC		51747	LUC7L3	denlrkqyekSsrfmkvgyer
IPI00107745	78	74	->PKA	->unsp		51747	LUC7L3	enlrkqyeksSrfmkvgyerd
IPI00107745	78	81	INSR->	INSR->		51747	LUC7L3	ekssrfmkvgYerdflrylqs
IPI00107745	166	174	unsp->unsp+cdc2			51747	LUC7L3	qlkeerellrSttstiesfaa
IPI00107745	231	226	PKC->	PKC->		51747	LUC7L3	qhmgyakikaTveelkeklrk
IPI00107745	231	238	unsp+PKG->unsp+PKG+PKA			51747	LUC7L3	eelkeklrkrTeepdrderlk
IPI00140420	641	638	unsp+PKC+PKA+DNAPK->unsp+PKA+DNAPK	unsp+PKC+PKA+DNAPK->unsp+PKA+DNAPK		27044	SND1	skvhftaersSyykssllsaee
IPI00140420	752	755	unsp+p38MAPK->GSK3	unsp+p38MAPK->p38MAPK		27044	SND1	wyrarvekveSpakihvfyid
IPI00143753	98	95	PKC->			23350	SR140	sigkmstakrTlskkeqeelk
IPI00143753	98	97	unsp+PKC+PKG+RSK->unsp+PKC+PKA+RSK	unsp+PKC+PKG+RSK->ATM+unsp+PKC+DNAPK+RSK		23350	SR140	gkmstakrtlSkkeqeelkkk
IPI00143753	105	97	unsp+PKC+PKG+RSK->unsp+PKC+PKG+PKA+RSK			23350	SR140	gkmstakrtlSkkeqeelkkk
IPI00143753	760	759	unsp+CKII->unsp	unsp+CKII->unsp+CKII+DNAPK		23350	SR140	knepifkvapSkweavdesel
IPI00146935	283	282	unsp->unsp+PKA+DNAPK	unsp->unsp+DNAPK		10059	DNM1L	kypslanrngTkylartlnrl
IPI00147874	79	76	unsp+PKC->unsp+PKA	unsp+PKC->unsp+PKA		54187	NANS	alerpytskhSwgktygehkr
IPI00147874	79	80		PKC->unsp+PKC		54187	NANS	pytskhswgkTygehkrhlef
IPI00147874	79	81		->EGFR		54187	NANS	ytskhswgktYgehkrhlefs
IPI00147874	290	294	unsp+PKC->			54187	NANS	emacneklgkSvvakvkipeg
IPI00147874	293	294	unsp+PKC->PKC	unsp+PKC->PKC		54187	NANS	emacneklgkSvvakvkipeg
IPI00149276	270	268	EGFR->			9577	BRE	tnkvqyviqgYhkrreyiaaf
IPI00149276	270	274		->EGFR		9577	BRE	viqgyhkrreYiaaflshfgt
IPI00149849	447	444	PKC->	PKC->		25839	COG4	etvnkavaldTyekgqltssm
IPI00149849	447	445	INSR+unsp->	INSR+unsp->unsp		25839	COG4	tvnkavaldtYekgqltssmv
IPI00149849	447	451	unsp->	unsp->		25839	COG4	aldtyekgqlTssmvddvfyi
IPI00150269	27	34	CKII+DNAPK->cdc2+CKII+DNAPK			9128	PRPF4	vvkkphiyygSleekererla
IPI00152216	202	195	unsp->unsp+cdc2			79608	RIC3	krllhqlreiTrvmkegkfid
IPI00152535	100	91	unsp->unsp+cdc2			26038	CHD5	dleeksesegSdyspnkkkkk
IPI00152535	100	94	unsp+CKI+cdk5+p38MAPK->unsp+cdk5+p38MAPK	unsp+CKI+cdk5+p38MAPK->unsp+cdk5+p38MAPK		26038	CHD5	eksesegsdySpnkkkkkklk
IPI00152535	858	857	->PKA	->DNAPK		26038	CHD5	deahrlknnqSkffrvlnsyk
IPI00152535	858	865	unsp+PKC->unsp+PKC+cdc2	unsp+PKC->unsp+PKC+cdc2		26038	CHD5	nqskffrvlnSykidykllt
IPI00152653	322	321	PKC->	PKC->ATM		1767	DNAH5	avlavlaaakSkllktwremd
IPI00152653	322	326	unsp+PKC->	unsp+PKC->PKC		1767	DNAH5	laaakskllkTwremdiritd
IPI00152688	419	414		unsp->		221078	NSUN6	ggegmrgaglSceqlkqlqrf
IPI00152692	65	64	->PKA	->DNAPK		92675	DTD1	vfedesgkhwSksvmdkqyei
IPI00152692	65	66	unsp+PKC->unsp+PKC+cdc2	unsp+PKC->unsp+PKC+cdc2		92675	DTD1	edesgkhwskSvmdkqyeilc
IPI00152695	98	93	unsp+PKC->unsp+PKA	unsp+PKC->unsp+PKC+PKA		80335	WDR82	nkiddtiryISlhdnkyiryf
IPI00152695	98	99	INSR+unsp+EGFR->EGFR	INSR+unsp+EGFR->INSR+EGFR		80335	WDR82	irylslhdnkYiryfpghskr
IPI00152695	98	102		->INSR		80335	WDR82	lslhdnkyirYfpghskrvva
IPI00152695	181	178	PKC->	PKC->		80335	WDR82	semvklydlrSfdkgpfatfk
IPI00152695	181	186		PKC->		80335	WDR82	lrsfdkgpfaTfkmqydrtce
IPI00152785	12	7	unsp->	unsp->		8348	HIST1H2BC	----mpdpakSapapkkgskk
IPI00152785	12	15	unsp+PKC->PKC	unsp+PKC->PKC		8348	HIST1H2BC	aksapapkkgSkkavtkaqkk
IPI00152785	13	15	unsp+PKC->PKC			8348	HIST1H2BC	aksapapkkgSkkavtkaqkk
IPI00152785	16	15	unsp+PKC->unsp+PKC+PKA	unsp+PKC->ATM+unsp+PKC+DNAPK		8348	HIST1H2BC	aksapapkkgSkkavtkaqkk
IPI00152785	17	15	unsp+PKC->PKC+PKA	unsp+PKC->PKC+PKA		8348	HIST1H2BC	aksapapkkgSkkavtkaqkk
IPI00152785	21	15	unsp+PKC->unsp+PKC+PKA	unsp+PKC->unsp+PKC+PKA		8348	HIST1H2BC	aksapapkkgSkkavtkaqkk
IPI00152785	21	20		PKC->PKC+DNAPK		8348	HIST1H2BC	apkkgskkavTkaqkkdgkkr
IPI00152785	109	113	unsp->			8348	HIST1H2BC	lpgelakhavSegtkavtkyt
IPI00152906	6	5		->DNAPK		3017	HIST1H2BD	------mpepTksapapkkgs
IPI00152906	12	15	unsp+PKC->PKC	unsp+PKC->PKC	Uniprot	3017	HIST1H2BD	tksapapkkgSkkavtkaqkk
IPI00152906	13	15	unsp+PKC->PKC		Uniprot	3017	HIST1H2BD	tksapapkkgSkkavtkaqkk
IPI00152906	16	15	unsp+PKC->unsp+PKC+PKA	unsp+PKC->ATM+unsp+PKC+DNAPK	Uniprot	3017	HIST1H2BD	tksapapkkgSkkavtkaqkk
IPI00152906	21	15	unsp+PKC->unsp+PKC+PKA	unsp+PKC->unsp+PKC+PKA	Uniprot	3017	HIST1H2BD	tksapapkkgSkkavtkaqkk
IPI00152906	21	20		PKC->PKC+DNAPK		3017	HIST1H2BD	apkkgskkavTkaqkkdgkkr
IPI00152906	109	113	unsp->			3017	HIST1H2BD	lpgelakhavSegtkavtkyt
IPI00152906	117	116		unsp+PKC->unsp+PKC+DNAPK		3017	HIST1H2BD	elakhavsegTkavtkytssk
IPI00152906	121	120		->ATM+DNAPK		3017	HIST1H2BD	havsegtkavTkytssk----
IPI00152906	121	123		PKC->		3017	HIST1H2BD	segtkavtkyTssk-------
IPI00152981	608	602		PKA->ATM+PKA		28976	ACAD9	enldeqikkvSqqilekrayi
IPI00152981	608	611	unsp+EGFR->EGFR	unsp+EGFR->EGFR		28976	ACAD9	vsqqilekraYicahpldrtc
IPI00154451	517	520	unsp+cdc2+DNAPK->cdc2+DNAPK	unsp+cdc2+DNAPK->cdc2+DNAPK		64210	MMS19	lyrlsflkedSqscrvaalea
IPI00154451	517	522	unsp->	unsp->		64210	MMS19	rlsflkedsqScrvaaleasg
IPI00155601	103	100	cdc2->	cdc2->		28992	MACROD1	ststdwkeakSflkglsdkqr
IPI00156649	477	480		->PKC		79930	DOK3	pqagfkaklvTllsrerrkgp
IPI00156649	477	483	unsp+PKC->unsp+CKI+PKC			79930	DOK3	gfkaklvtllSrerrkgpapc
IPI00159322	1267	1259	unsp+PKC+PKG+PKB+PKA+RSK->unsp+PKC+PKG+PKB+cdc2+PKA+RSK			6942	TCF20	nplimrrrvrSfispipskrq
IPI00159322	1267	1262	unsp+GSK3+p38MAPK->p38MAPK	unsp+GSK3+p38MAPK->p38MAPK		6942	TCF20	imrrrvrsfiSpipskrqsqd
IPI00159322	1267	1266		unsp+PKC->unsp+PKC+DNAPK		6942	TCF20	rvrsfispipSkrqsqdvkns
IPI00159322	1267	1270	ATM+unsp+PKC+DNAPK->ATM+DNAPK	ATM+unsp+PKC+DNAPK->ATM+PKC+DNAPK		6942	TCF20	fispipskrqSqdvknssted

Table S3

IPI00162563	20	15	PKC+cdc2->	PKC+cdc2->cdc2		9810	RNF40	gnkraagdggSgppekklsre
IPI00162563	20	23	unsp->unsp+CKII	unsp->unsp+CKII		9810	RNF40	ggsgppekklSreekttttli
IPI00163496	801	805	PKC->			8500	PPFIA1	hkapkkkgikSsigrlfgkke
IPI00163496	801	806	unsp+PKA->	unsp+PKA->PKA		8500	PPFIA1	kapkkkgiksSigrlfgkkek
IPI00163496	804	806	unsp+PKA->PKC	unsp+PKA->unsp		8500	PPFIA1	kapkkkgiksSigrlfgkkek
IPI00163505	103	100	PKC->	PKC->	Uniprot|PhosphoSite	9584	RBM39	rfrgryrspySgpkfnsairg
IPI00163505	103	106	unsp+cdc2->cdc2	unsp+cdc2->cdc2		9584	RBM39	rspysgpkfnSairgkiglph
IPI00164352	1117	1112		ATM+DNAPK->DNAPK		23036	ZNF292	vegctrtynsSqsigkhmkta
IPI00164352	1117	1114	unsp+CKI+PKC->			23036	ZNF292	gctrtynssqSigkhmktahp
IPI00164352	1117	1121		PKG->		23036	ZNF292	ssqsigkhmkTahpdqyaafk
IPI00166055	21	19	ATM+PKC+DNAPK->ATM+DNAPK	ATM+PKC+DNAPK->ATM+DNAPK		401466	C8orf59	pksrnvfhiaSqknfkaknka
IPI00166153	108	110	ATM+unsp->ATM	ATM+unsp->ATM+unsp+DNAPK		23070	FTSJD2	fregeglgkySqgrkdiveas
IPI00166293	6	5	->DNAPK	->ATM+DNAPK		128312	HIST3H2BB	------mpdpSksapakkgs
IPI00166293	6	7	unsp->	unsp->		128312	HIST3H2BB	----mpdpskSapapkkgskk
IPI00166293	12	7	unsp->	unsp->		128312	HIST3H2BB	----mpdpskSapapkkgskk
IPI00166293	12	15	unsp+PKC->PKC	unsp+PKC->PKC	Uniprot	128312	HIST3H2BB	sksapapkkgSkkavtkaqkk
IPI00166293	13	15	unsp+PKC->PKC		Uniprot	128312	HIST3H2BB	sksapapkkgSkkavtkaqkk
IPI00166395	439	445	->PKA			197322	ACSF3	eywnkpeetkSaftldgwfkt
IPI00166500	114	112	INSR->			51588	PIAS4	agpnidypvlYgkylnglrl
IPI00166528	582	584	PKG+cdc2+PKA->PKC+cdc2	PKG+cdc2+PKA->cdc2		253260	RICTOR	vrrllyfykpSsklyanldld
IPI00166528	582	585	PKC->			253260	RICTOR	rrllyfykpsSklyanldldf
IPI00166555	511	507	unsp+PKC->unsp	unsp+PKC->PKC+PKG		64062	RBM26	sgepgvptkkTwfdkpnfnrt
IPI00166749	299	305	unsp+RSK->unsp+PKA+RSK	unsp+RSK->unsp+PKA+RSK		23203	PMPCA	ggiaklerdmSnvslgptpip
IPI00167196	54	57	unsp+CKII->cdc2+CKII	unsp+CKII->cdc2+CKII		164153	UBL4B	gqlleddkhlSdycigpnasi
IPI00167535	346	342	PKC->			57634	EP400	sslpltsvgnTgmkkvpkkle
IPI00167535	1634	1633		cdc2->ATM+cdc2+DNAPK		57634	EP400	etpvtlqfqgSkftlshsqlr
IPI00167535	1634	1636	PKC+cdc2->PKC	PKC+cdc2->cdc2		57634	EP400	vtlqfqgskfTlshsqlrqlt
IPI00167535	2356	2358	PKG->	PKG->		57634	EP400	akplptfakpTaepgqdnpew
IPI00167904	103	102		->ATM		160762	CCDC63	nymelrlllqTkedyealiks
IPI00167904	103	106	INSR+unsp->	INSR+unsp->unsp		160762	CCDC63	lrlllqtkedYealikslkvl
IPI00167904	111	102	->cdc2			160762	CCDC63	nymelrlllqTkedyealiks
IPI00167941	1683	1679	unsp+PKC->unsp+PKA			23195	MDN1	ikrlakivrlTeyqknelkiy
IPI00167941	1683	1681	EGFR->	EGFR->unsp+EGFR		23195	MDN1	rlakivrlteYqknelkiydr
IPI00168603	496	490		unsp->unsp+GSK3		55349	CHDH	pfrgkelqpgShiqsdkeida
IPI00168603	496	494	unsp+CKII->unsp+cdc2+CKII			55349	CHDH	kelqpgshiqSdkeidafvra
IPI00168839	153	156	unsp+PKC+PKA->PKC+PKA	unsp+PKC+PKA->PKC+PKA		144106	FAM10A5	rlailyakraSvfvklqkpna
IPI00168899	135	133	unsp+PKC+PKG+PKA->unsp+PKG+PKA	unsp+PKC+PKG+PKA->unsp+PKG+PKA		80174	DBF4B	vdpkgshprpSrkpvdsvpls
IPI00168899	135	139	unsp->			80174	DBF4B	hprpsrkpvdSvplsrgkell
IPI00169383	11	2	CKI+PKA+DNAPK->CKI+cdc2+PKA+DNAPK			5230	PGK1	---------mSlsnkltldkl
IPI00169383	11	8	->PKA			5230	PGK1	---mslsnklTldkldvkgkr
IPI00169383	75	76	unsp->	unsp->	Uniprot|PhosphoSite	5230	PGK1	rpdgvpmpdkYslepvavelk
IPI00169383	75	77	PKA->	PKA->		5230	PGK1	pdgvpmpdkySlepvavelks
IPI00169383	86	87	cdc2->CKI+cdc2			5230	PGK1	slepvavelkSllgkdvlflk
IPI00169383	156	153	unsp+PKA+RSK+DNAPK->PKA+DNAPK	unsp+PKA+RSK+DNAPK->unsp+PKA+DNAPK		5230	PGK1	epakieafraSlsklgdvyvn
IPI00169383	156	155		->ATM+DNAPK		5230	PGK1	akieafraslSklgdvyvnda
IPI00169383	199	196		unsp->	niprot|PHOsphoELM|PhosphoSi	5230	PGK1	ggflmkkelnYfakalesper
IPI00169383	267	271	PKC+CKII->CKII			5230	PGK1	egakivkdlmSkaekngvkit
IPI00169383	291	288	->CKII	->CKII		5230	PGK1	vkitlpvdfvTadkfdenakt
IPI00169383	322	320	unsp+PKC+CKII->CKII	unsp+PKC+CKII->CKII		5230	PGK1	gwmgldcgpeSskkyaeavtr
IPI00169383	322	321	unsp+PKC+cdc2->cdc2	unsp+PKC+cdc2->unsp+cdc2+DNAPK		5230	PGK1	wmgldcgpesSkkyaeavtra
IPI00169383	323	320	unsp+PKC+CKII->unsp+CKII			5230	PGK1	gwmgldcgpeSskkyaeavtr
IPI00169383	323	321	unsp+PKC+cdc2->cdc2	unsp+PKC+cdc2->cdc2		5230	PGK1	wmgldcgpesSkkyaeavtra
IPI00170596	723	724	unsp->	unsp->		25942	SIN3A	nkvwreqnekYylksldhqgi
IPI00170596	723	728		PKC->		25942	SIN3A	reqnekyylkSldhqginfkq
IPI00170596	727	724		unsp->		25942	SIN3A	nkvwreqnekYylksldhqgi
IPI00170596	727	725	unsp->			25942	SIN3A	kvwreqnekyYlksldhqgin
IPI00170596	727	728	PKC->PKC+PKA	PKC->PKC+PKA		25942	SIN3A	reqnekyylkSldhqginfkq
IPI00170596	934	938	unsp+PKG+cdc2+PKA+RSK->unsp+PKG+cdc2+PKA		PhosphoSite	25942	SIN3A	revlgikrdkSdspaiqlrlk
IPI00170596	934	940	unsp+p38MAPK->unsp		Uniprot|PhosphoSite	25942	SIN3A	vlgikrdksdSpaiqlrlkep
IPI00170786	13	6		unsp+PKC+PKA+RSK->unsp+PKC+PKA		51729	WBP11	-----mgrrsTsstksgkfmn
IPI00170786	13	9	CKI+PKC->CKI			51729	WBP11	--mgrrstssTksgkfmnptd
IPI00170786	13	11	unsp+PKC->	unsp+PKC->		51729	WBP11	mgrrstsstkSgkfmnptdqa
IPI00170786	13	18	PKC->	PKC->		51729	WBP11	stksgkfmnpTdqarkearkr
IPI00170867	512	515	unsp+cdc2+PKA->unsp+cdc2	unsp+cdc2+PKA->unsp+cdc2		1616	DAXX	eknlepgkqiSrssgeqqnkg
IPI00171127	126	124	unsp+PKC+CKII->unsp+CKII	unsp+PKC+CKII->unsp+CKII		55833	UBAP2	senkenrekkSekessrgrgn
IPI00171127	126	129	unsp->unsp+cdc2	unsp->unsp+cdc2		55833	UBAP2	nrekksekesSrgrgnnnrkg
IPI00171390	23	19	unsp->			84950	PRPF38A	ahsihgtnpqYlvekiirtri
IPI00171390	23	27	PKC->			84950	PRPF38A	pqylvekiirTriyeskywke
IPI00171611	10	12	unsp+PKC+PKG->PKC	unsp+PKC+PKG->unsp+PKC	Uniprot	653604	HIST2H3D	artkqtarksTggkaprkqla
IPI00171611	15	11		unsp->unsp+PKG	Uniprot	653604	HIST2H3D	martkqtarkStggkaprkql
IPI00171611	15	12	unsp+PKC+PKG->PKG+PKA	unsp+PKC+PKG->PKC+PKG	Uniprot	653604	HIST2H3D	artkqtarksTggkaprkqla
IPI00171611	24	23		PKC->PKC+DNAPK		653604	HIST2H3D	ggkaprkqlaTkaarksapat
IPI00171611	24	29	unsp+PKG+PKA->unsp+PKA			653604	HIST2H3D	kqlatkaarkSapatggvkkp
IPI00171611	57	58	unsp->	unsp->		653604	HIST2H3D	alreirryqkStellirklpf
IPI00171611	80	88	->cdc2			653604	HIST2H3D	dfktdlrfqsSavmalqease
IPI00171692	87	84	->PKA			83451	ABHD11	glfgsktnfnSiakilaqqtg
IPI00171779	260	258	PKC->	PKC->		64431	ACTR6	fckpreemvlSgkyksgeqil
IPI00171779	262	258		PKC->unsp+PKC		64431	ACTR6	fckpreemvlSgkyksgeqil
IPI00171903	239	243	unsp+PKA->PKA	unsp+PKA->unsp		4670	HNRNPM	adiledkdgkSrgigtvtfeq
IPI00172487	101	104	unsp+PKA->unsp	unsp+PKA->unsp+PKC+CKII		23519	ANP32D	nlsgnkikdlStieplkklen
IPI00174775	142	145	unsp+PKC->unsp			319101	KRT73	qikvlnnkfaSfidkvrfleq
IPI00175151	53	60		INSR->		54790	TET2	gdtkwhsfksYygipcmkgsq
IPI00175151	53	61	unsp+EGFR->INSR+unsp+EGFR	unsp+EGFR->INSR+unsp+EGFR		54790	TET2	dtkwhsfksyYgipcmkgsqn
IPI00176157	538	535	PKC->	PKC->		134359	C5orf37	pvtvqtipqaTaakyprtihp
IPI00176706	21	16	CKI+PKC->CKI			84991	RBM17	dlgvetsdskTegwsknfkll
IPI00176706	21	20		->DNAPK		84991	RBM17	etsdsktegwSknfkllqsql
IPI00176903	109	102	unsp->unsp+cdc2			284119	PTRF	elsklgkahaTtsntvsklle
IPI00176903	109	106	PKC->	PKC->		284119	PTRF	lgkahattsnTvskllekvrk
IPI00176903	109	108		->ATM+DNAPK		284119	PTRF	kahattsntvSkllekvrkvs
IPI00177728	9	5	PKC->	PKC->		55748	CNDP2	------maalTtlfkyideng
IPI00177728	9	6	PKC->			55748	CNDP2	-----maaltTlfkyidenqd
IPI00177728	9	10	unsp->	unsp->		55748	CNDP2	-maalttlfkYidenqdryik
IPI00177888	363	360	unsp+PKC+PKG+PKB+PKA+RSK->PKG+PKB+PKA+RSK	unsp+PKC+PKG+PKB+PKA+RSK->unsp+PKG+PKB+PKA+RSK		114823	LENG8	aggagrargnSftkfgnrnvf
IPI00178150	868	859	CKII->cdc2+CKII			24137	KIF4A	rpkqrweniaTileakcalky
IPI00178431	193	197	unsp+PKC+cdc2->cdc2			5965	RECQL	iyvtpekiakSkmfmsrleka
IPI00178431	206	197		unsp+PKC+cdc2->unsp+PKC		5965	RECQL	iyvtpekiakSkmfmsrleka
IPI00178431	206	202	CKII->PKA+CKII	CKII->unsp+PKG+CKII		5965	RECQL	ekiakskmfmSrlekayearr
IPI00178431	206	208	->SRC			5965	RECQL	kmfmsrlekaYearrftriav
IPI00178431	514	511	unsp+p38MAPK->p38MAPK	unsp+p38MAPK->p38MAPK		5965	RECQL	kqaeelneklTplklidswmg
IPI00178431	522	518	cdc2->			5965	RECQL	ekltplklidSwmgkgaaklr
IPI00178440	60	61	unsp+PKC+cdc2->unsp+PKC+cdc2+DNAPK	unsp+PKC+cdc2->unsp+PKC+CKII+cdc2+DNAPK		1933	EEF1B2	halrwynhikSyekekaslpg
IPI00178440	60	68	PKA->	PKA->		1933	EEF1B2	hiksyekekaSlpgvkkalgk
IPI00178440	185	182		->EGFR		1933	EEF1B2	wgssklvpvgYgikklqiqcv
IPI00178512	792	785	->cdc2			55732	C1orf112	anvtveeakrSslqpyakrar
IPI00178744	95	94	unsp+cdc2->cdc2	unsp+cdc2->unsp+cdc2+DNAPK		37	ACADVL	rpyaggaaqeSksfavgmfkg
IPI00178744	263	264	unsp->	unsp->		37	ACADVL	tsavpspcgkYytlngsklwi
IPI00178744	263	265	INSR->unsp+INSR	INSR->unsp+INSR		37	ACADVL	savpspcgkyYtlngsklwis
IPI00178744	263	266	PKC->			37	ACADVL	avpspcgkyyTlngsklwisn
IPI00178744	263	270	->cdc2			37	ACADVL	pcgkyytlngSklwisnggla
IPI00178744	300	296	PKC->	PKC->		37	ACADVL	faktpvtdpaTgavkekitaf
IPI00178750	134	137	unsp+PKC->PKC	unsp+PKC->PKC		80011	FAM192A	enkkevekklTvkpietknkf
IPI00178750	135	137	unsp+PKC->PKC			80011	FAM192A	enkkevekklTvkpietknkf
IPI00178750	139	137	unsp+PKC->	unsp+PKC->		80011	FAM192A	enkkevekklTvkpietknkf
IPI00178750	139	143	unsp+PKC->PKC	unsp+PKC->PKC		80011	FAM192A	ekkltvkpieTknkfsqakll
IPI00178750	144	143		unsp+PKC->unsp+PKC+DNAPK		80011	FAM192A	ekkltvkpieTknkfsqakll
IPI00178750	144	148	unsp+PKA+DNAPK->PKA+DNAPK	unsp+PKA+DNAPK->ATM+DNAPK		80011	FAM192A	vkpietknkfSqakllagavk
IPI00178750	146	143	unsp+PKC->	unsp+PKC->PKC		80011	FAM192A	ekkltvkpieTknkfsqakll
IPI00178750	146	148	unsp+PKA+DNAPK->DNAPK	unsp+PKA+DNAPK->DNAPK		80011	FAM192A	vkpietknkfSqakllagavk
IPI00179330	6	7		PKC->unsp+PKC		7316	UBC	----mqifvkTltgktitlev
IPI00179330	6	12	->PKA			7316	UBC	qifvktltgkTitlevepsdt
IPI00179330	11	7	PKC->	PKC->		7316	UBC	----mqifvkTltgktitlev
IPI00179330	11	9		PKC->		7316	UBC	--mqifvktlTgktitlevep
IPI00179330	113	115	unsp->	unsp->		7316	UBC	yykvdengkiSrlrrecpsde
IPI00179330	152	148	unsp->	unsp->		7316	UBC	rhycgkccltYcfnkpedk--
IPI00179709	40	38	PKC->	PKC->		113457	TUBA3D	hgiqpdgqmpSdktigggdds
IPI00179709	40	41	->CKI	->unsp		113457	TUBA3D	qpdgqmpsdkTigggddsfnt
IPI00179709	163	158	unsp->unsp+PKA			113457	TUBA3D	gfasllmerlSvdygkkskle
IPI00179709	163	165	unsp+PKG+CKII->unsp+CKII	unsp+PKG+CKII->unsp+CKII	PhosphoSite	113457	TUBA3D	erlsvdygkkSklefaiypap

IPI00179709	280	282	EGFR->unsp	EGFR->unsp+EGFR	PhosphoSite	113457	TUBA3D	yapvisaekaYheqlsvaeit
IPI00179709	401	399	unsp->			113457	TUBA3D	arldhkfdlmYakrafvhwyv
IPI00180240	32	31		unsp->ATM+unsp+DNAPK		7117	TMSL3	tetqeknplpSketieqekqa
IPI00180240	32	34		->unsp		7117	TMSL3	qeknplpskeTieqekqages
IPI00180675	40	38	PKC->	PKC->		7846	TUBA1A	hgiqpdgqmpSdktigggdds
IPI00180675	40	41	->CKI	->unsp		7846	TUBA1A	qpdgqmpsdkTigggddsfnt
IPI00180675	164	165	unsp+PKC+PKG+CKII->unsp+PKC+CKII	unsp+PKC+PKG+CKII->unsp+PKC+CKII		7846	TUBA1A	erlsvdygkkSklefsiypap
IPI00180675	401	399	unsp->			7846	TUBA1A	arldhkfdlmYakrafvhwyv
IPI00180764	171	167	unsp+PKC->unsp	unsp+PKC->unsp	PhosphoSite	11143	MYST2	mslkdsgsdlShrpkrrrfhe
IPI00180764	199	197	unsp->CKII	unsp->		11143	MYST2	tpgcnslghlTgkherhfsis
IPI00181728	276	271	unsp+PKC->unsp	unsp+PKC->PKC		55299	BRIX1	spnmhrrvirSitaakyrekq
IPI00182116	118	120	->PKC			55843	ARHGAP15	ssrriefykeSkqqalsnmkt
IPI00182533	101	102	INSR->			6158	RPL28	sirhmirknkYrpdlrmaair
IPI00182632	274	273		PKC->		80764	THAP7	rreqrlrlrlTklqqerarek
IPI00182728	434	437	->CKII	->CKII		9525	VPS4B	ehdllklkkfTedfgqeg---
IPI00182757	287	285	unsp+PKC+CKII->unsp+CKII	unsp+PKC+CKII->unsp+CKII		57805	KIAA1967	lhhpsriqvsSekeaapdaga
IPI00183208	197	200	PKC->			26263	FBXO22	fhfikdpknlTlerhqltevg
IPI00183626	259	258		CKI->ATM+CKI+DNAPK		5725	PTBP1	nacctlridfSkltslnvkyn
IPI00183626	259	261		PKC->		5725	PTBP1	ctlridfsklTslnvkynndk
IPI00183626	266	261	PKC->	PKC->		5725	PTBP1	ctlridfsklTslnvkynndk
IPI00183626	266	262	PKC+cdc2->	PKC+cdc2->cdc2		5725	PTBP1	tlridfskltSlnvkynndks
IPI00183626	554	551	unsp+PKC+PKA->PKC+PKA			5725	PTBP1	dlgenhhlrvSfsksti----
IPI00183626	554	553		->ATM+DNAPK		5725	PTBP1	genhhlrvsfSksti------
IPI00183626	554	555	->PKC	->PKC		5725	PTBP1	nhhlrvsfskSti--------
IPI00183695	28	27		unsp->ATM+unsp+DNAPK		6281	S100A10	hkfagdkgylTkedlrvlmek
IPI00183968	119	124	unsp->unsp+CKI			7170	TPM3	leeaekaadeSergmkvienr
IPI00184284	54	50		unsp->INSR+unsp		51699	VPS29	ctgnlctkesYdylktlagdv
IPI00184284	54	52	INSR+unsp->INSR			51699	VPS29	gnlctkesydYlktlagdvhi
IPI00184330	216	220	unsp+CKII+PKA->unsp+CKII	unsp+CKII+PKA->unsp+cdc2+CKII	PhosphoSite	4171	MCM2	hghnvfkeriSdmckenresl
IPI00184330	896	892		->unsp		4171	MCM2	dselfrmnkfShdlkrkmilq
IPI00184533	245	247	PKA->CKI	PKA->CKI		8237	USP11	qedtrlwaknSegsldrlydt
IPI00184533	245	250	unsp+CKI+DNAPK->CKI+DNAPK	unsp+CKI+DNAPK->CKI+DNAPK		8237	USP11	trlwaknsegSldrlydthit
IPI00185027	1158	1157		PKC+cdc2->ATM+PKC+cdc2+DNAPK		473	RERE	tdlyfmplagSklakkreeai
IPI00185361	517	522	unsp+PKC->PKC	unsp+PKC->PKC		57696	DDX55	rrkfiknkawSkqkakkekkk
IPI00185361	523	522		unsp+PKC->unsp+PKC+DNAPK		57696	DDX55	rrkfiknkawSkqkakkekkk
IPI00185374	221	219	unsp+CKII->CKII	unsp+CKII->CKII		5718	PSMD12	intkffqeenTeklklkyynl
IPI00185374	368	369	INSR->			5718	PSMD12	vehnirimakYytritmkrma
IPI00185374	368	370	INSR->	INSR->		5718	PSMD12	ehnirimakyYtritmkrmaq
IPI00186224	141	142	ATM+unsp+PKC->ATM+unsp+PKC+DNAPK	ATM+unsp+PKC->ATM+unsp+PKC+DNAPK		4261	CIITA	mempaevgqkSqkrpfpeelp
IPI00186224	144	142	ATM+unsp+PKC->ATM+unsp	ATM+unsp+PKC->ATM+unsp+DNAPK		4261	CIITA	mempaevgqkSqkrpfpeelp
IPI00186290	235	232		unsp->		1938	EEF2	aftlkqfaemYvakfaakgeg
IPI00186290	272	265	INSR+unsp->unsp	INSR+unsp->unsp	Uniprot	1938	EEF2	dmmkklwgdrYfdpangkfsk
IPI00186290	275	274		unsp+PKC->ATM+unsp+PKC+DNAPK		1938	EEF2	ryfdpangkfSksatspegkk
IPI00186290	275	278		CKII->PKC+CKII		1938	EEF2	pangkfsksaTspegkklprt
IPI00186290	426	422	PKC->	PKC->		1938	EEF2	afgrvfsglvStglkvrimgp
IPI00215611	22	19	unsp+RSK->PKA+RSK	unsp+RSK->unsp+PKA		1396	CRIP1	kevyfaervtSlgkdwhrpcl
IPI00215637	130	125	->PKA		Uniprot|PhosphoSite	1654	DDX3X	gfgkferggnSrwcdksdedd
IPI00215719	154	158	unsp+cdk5+p38MAPK->cdk5+p38MAPK		Uniprot|PhosphoSite	6141	RPL18	vyrhfgkapgTphshtkpyvr
IPI00215780	23	25	unsp+PKC->PKC	unsp+PKC->PKC		6223	RPS19	vralaaflkkSgklkvpewvd
IPI00215780	77	74	cdc2+DNAPK->DNAPK	cdc2+DNAPK->DNAPK		6223	RPS19	lylrggagvgSmtkiyggrqr
IPI00215780	77	76		->DNAPK		6223	RPS19	lrggagvgsmTkiyggrqrng
IPI00215790	67	59	PKA->cdc2+PKA			6169	RPL38	dkekaeklkqSlppglavkel
IPI00215878	21	16	unsp+PKC+PKA+cdc2+DNAPK->unsp+PKA+cdc2+DNAPK			514	ATP5E	rqaglsyirySqicakavrda
IPI00215911	197	201	unsp+PKC->			328	APEX1	afrkflkglaSrkplvlcgdl
IPI00215914	36	31	PKC->	PKC->		375	ARF1	lmvgldaagkTtilyklklge
IPI00215963	739	738		unsp+PKB+PKA+RSK->unsp+PKB+CaM-II+RSK		23199	KIAA0182	eflqqrrrlvSkldleerrrr
IPI00215965	3	2	CKI+CKII->CKI+DNAPK	CKI+CKII->CKI+CKII+DNAPK	PHOsphoELM	3178	HNRNPA1	---------mSksespkepeq
IPI00215965	3	4		cdc2->CKII+cdc2	niprot|PHOsphoELM|PhosphoSi	3178	HNRNPA1	-------mskSespkepeqlr
IPI00215965	3	6	unsp+GSK3+CKII->unsp+GSK3+cdc2+CKII	unsp+GSK3+CKII->unsp+GSK3+cdc2+CKII	niprot|PHOsphoELM|PhosphoSi	3178	HNRNPA1	-----mskseSpkepeqlrkl
IPI00215965	52	51		unsp+PKC->unsp+PKC+DNAPK		3178	HNRNPA1	tdcvvmrdpnTkrsrgfgfvt
IPI00215965	52	54	unsp+PKG+PKA->unsp+PKG	unsp+PKG+PKA->unsp+PKG		3178	HNRNPA1	vvmrdpntkrSrgfgfvtyat
IPI00215965	113	116	->CKII	->CKII		3178	HNRNPA1	kifvggikedTeehhlrdyfe
IPI00215999	225	223	unsp->	unsp->		984	CDK11B	ahhrtmredySdkvkashwsr
IPI00216008	89	84	unsp+PKC+PKG+PKA+RSK->unsp+PKG+PKA+RSK	unsp+PKC+PKG+PKA+RSK->unsp+PKG+PKA+RSK		2539	G6PD	rltvadirkqSepffkatpee
IPI00216008	449	447	SRC+unsp+EGFR->SRC+unsp		Uniprot|PHOsphoELM|PhosphoSi	2539	G6PD	virvqpneavYtkmmtkkpgm
IPI00216008	449	448	unsp+PKC->	unsp+PKC->		2539	G6PD	irvqpneavyTkmmtkkpgmf
IPI00216047	326	321	unsp+PKC+PKA->unsp+PKA	unsp+PKC+PKA->PKC+PKA		6601	SMARCC2	akkknakkgpStpytkskrgh
IPI00216047	326	325		PKC->PKC+DNAPK		6601	SMARCC2	nakkgpstpyTkskrghreee
IPI00216092	6	8	PKG+PKA->	PKG+PKA->		1414	CRYBB1	---msqaakaSasatvavnpg
IPI00216132	304	301	unsp+RSK->PKA	unsp+RSK->unsp+PKA		6198	RPS6KB1	ppftgenrkkTidkilkckln
IPI00216137	111	107	->PKA+RSK	->PKA		6847	SYCP1	ensegIsrvySklykeaekik
IPI00216138	17	16		unsp+PKC+CKII->ATM+unsp+PKC+CKII+DNAPK		6876	TAGLN	psygmsrevqSkiekkydeel
IPI00216164	219	214	CKII->			1962	EHHADH	qslpnmdsifSeallkmrrqh
IPI00216164	346	342	PKC+cdc2+CKII->CKII	PKC+cdc2+CKII->unsp+cdc2+CKII		1962	EHHADH	qlatankmitSvlekeaskmq
IPI00216164	584	580		->EGFR		1962	EHHADH	rfgqktgkgwYqydkplgrih
IPI00216199	293	291	unsp->		PhosphoSite	2625	GATA3	ghylcnacglYhkmngqnrpl
IPI00216293	14	12	unsp+PKC->	unsp+PKC->		7263	TST	vhqvlyralvStkwlaesirt
IPI00216293	14	13		->DNAPK		7263	TST	hqvlyralvsTkwlaesirtg
IPI00216293	14	19	unsp+CKI->CKI	unsp+CKI->		7263	TST	alvstkwlaeSirtgklgpgl
IPI00216298	39	44	unsp+PKA->	unsp+PKA->		7295	TXN	pckmikpffhSlsekysnvif
IPI00216298	94	90	->unsp	->unsp		7295	TXN	fkkgqkvgefSgankekleat
IPI00216308	20	19	PKC->CKI	PKC->		7416	VDAC1	dlgksardvfTkgygfglikl
IPI00216308	28	33	PKC+PKG->PKC			7416	VDAC1	gfglikldlkTksenglefts
IPI00216308	61	57	unsp+CKI+DNAPK->unsp+CKI+PKA+DNAPK			7416	VDAC1	antettkvtgSletkyrwtey
IPI00216308	61	60		CKI+PKC->CKI+PKC+DNAPK		7416	VDAC1	ettkvtgsleTkyrwteyglt
IPI00216308	61	62	unsp->	unsp->		7416	VDAC1	tkvtgsletkYrwteygltft
IPI00216308	109	101	->PKA		Uniprot|PHOsphoELM|PhosphoSi	7416	VDAC1	larglkltfdSsfspntgkkn
IPI00216308	109	102	->cdc2			7416	VDAC1	arglkltfdsSfspntgkkna
IPI00216308	109	107	unsp+PKC->	unsp+PKC->	Uniprot|PhosphoSite	7416	VDAC1	ltfdssfspnTgkknakiktg
IPI00216318	70	66	PKC->			7529	YWHAB	arrsswrvisSieqkternek
IPI00216318	70	71	unsp->unsp+CKII	unsp->unsp+CKII		7529	YWHAB	wrvissieqkTernekkqqmg
IPI00216318	117	116		->ATM+DNAPK		7529	YWHAB	ylipnatqpeSkvfylkmkgd
IPI00216318	117	120	unsp->	unsp->		7529	YWHAB	natqpeskvfYlkmkgdyfry
IPI00216319	120	119		->ATM+DNAPK		7533	YWHAH	ikncndfqyeSkvfylkmkgd
IPI00216319	120	123	unsp->	unsp->		7533	YWHAH	ndfqyeskvfYlkmkgdyyry
IPI00216383	261	257	unsp+PKC->unsp+PKA	unsp+PKC->unsp+PKC+PKA		5892	RAD51L3	vtnhitrdrdSgrlkpalgrs
IPI00216383	261	267	cdc2->PKA+cdc2			5892	RAD51L3	sgrlkpalgrSwsfvpstril
IPI00216402	10	12	unsp+PKC+PKG->PKC	unsp+PKC+PKG->unsp+PKC	Uniprot	8290	HIST3H3	artkqtarksTggkaprkqla
IPI00216402	15	11		unsp->unsp+PKG	Uniprot	8290	HIST3H3	martkqtarkStggkaprkql
IPI00216402	15	12	unsp+PKC+PKG->PKG+PKA	unsp+PKC+PKG->PKC+PKG	Uniprot	8290	HIST3H3	artkqtarksTggkaprkqla
IPI00216402	24	29	unsp+PKC+PKG+PKA->unsp+PKC+PKA			8290	HIST3H3	kqlatkvarkSapatggvkkp
IPI00216402	28	29	unsp+PKC+PKG+PKA->unsp+PKC+PKA	unsp+PKC+PKG+PKA->unsp+PKC+PKA		8290	HIST3H3	kqlatkvarkSapatggvkkp
IPI00216456	6	2	PKC->		Uniprot|PhosphoSite	8334	HIST1H2AC	---------mSgrgkqggkar
IPI00216457	6	2	PKC->		Uniprot|PhosphoSite	723790	HIST2H2AA	---------mSgrgkqggkar
IPI00216470	150	153	unsp->unsp+CKII	unsp->unsp+CKII		8396	PIP4K2B	ydrrfviktvSsedvaemhni
IPI00216654	33	28		unsp->		9221	NOLC1	vlgflrdnqlSevankfakat
IPI00216654	33	38	PKC->	PKC->		9221	NOLC1	sevankfakaTgatqqdanas
IPI00216654	673	675	PKC+PKG->PKC	PKC+PKG->PKC		9221	NOLC1	geranqvlkfTkgksfrhekt
IPI00216691	38	44	cdk5+p38MAPK->	cdk5+p38MAPK->p38MAPK		5216	PFN1	avpgktfvniTpaevgvlvgk
IPI00216691	105	104		PKC->PKC+DNAPK		5216	PFN1	ggaptfnvtvTktdktlvllm
IPI00216691	105	109		PKA->		5216	PFN1	fnvtvtktdkTlvllmgkegv
IPI00216691	108	104	PKC->			5216	PFN1	ggaptfnvtvTktdktlvllm
IPI00216691	108	109		PKA->unsp+PKA		5216	PFN1	fnvtvtktdkTlvllmgkegv
IPI00216694	335	339	unsp+RSK->unsp			5358	PLS3	netddlkraeSmlqqadklgc
IPI00216704	1262	1267	cdc2+PKA->cdc2			6710	SPTB	rknnekaqeaSvllrdnlelq
IPI00216730	6	2	PKC->		Uniprot	317772	HIST2H2AB	---------mSgrgkqggkar
IPI00216873	99	95	PKC->	PKC->		90459	ERI1	raklsefkleTrgvkdvlkkr
IPI00216932	642	640	unsp+PKC->	unsp+PKC->		84532	ACSS1	lvvkrlpktrSgkvmrrllrk
IPI00216951	74	69	PKC->			1615	DARS	vvwvrarvhtSrakgkqcflv
IPI00216951	374	370		unsp+CKII+p38MAPK->CKII+p38MAPK		1615	DARS	vemgdeddlsTpnekllghlv
IPI00216975	112	108	->PKA			7171	TPM4	eldraqerlaTalqkleeaek
IPI00216975	118	123	unsp->unsp+CKI			7171	TPM4	leeaekaadeSergmkvienr
IPI00216975	161	162	INSR+unsp+EGFR->SRC+unsp+EGFR		Uniprot|PhosphoSite	7171	TPM4	khiaeeadrkYeevarklvil
IPI00216975	213	211	unsp+CKII->CKII			7171	TPM4	tnnlksleaaSekysekedky
IPI00216975	213	215	unsp+CKII->unsp+PKC+CKII			7171	TPM4	ksleaasekySekedkyeeei
IPI00216975	220	221	INSR+SRC+unsp->SRC+unsp			7171	TPM4	sekysekedkYeeeikllsdk
IPI00217013	655	658	unsp+PKC->PKC+cdc2	unsp+PKC->PKC+cdc2		55671	SMEK1	rerqdnpkldSmrsilrnhry
IPI00217018	1259	1263	unsp+PKC+PKG->unsp+PKC	unsp+PKC+PKG->unsp+PKC		54809	SAMD9	yipyltklkfSlkksfdffde

Table S3

IPI00217018	1259	1267	unsp+PKG->unsp+PKG+cdc2			54809	SAMD9	ltklkfslkkSfdffdeyfvl
IPI00217030	22	24	->PKC			6191	RPS4X	apkhwmldklTgvfaprpstg
IPI00217030	134	133		PKC->PKC+DNAPK		6191	RPS4X	lckvrkifvgTkgiphlvthd
IPI00217049	378	375	PKC->	PKC->		4939	OAS2	qidsavniirTflkencfrqs
IPI00217223	79	83	unsp+PKC+cdc2->cdc2			10606	PAICS	knhlegkaaiSnkitscifql
IPI00217240	466	463	PKC->	PKC->		84128	WDR75	aekseqptlvTaskdgyfkvw
IPI00217240	466	465	unsp->CKI+DNAPK	unsp->ATM+unsp+DNAPK		84128	WDR75	kseqptlvtaSkdgyfkvwil
IPI00217240	466	469	INSR+unsp->unsp	INSR+unsp->unsp		84128	WDR75	ptlvtaskdgYfkvwiltdds
IPI00217259	511	508		->EGFR	PhosphoSite	56155	TEX14	drtmnlqdirYilkndlkdft
IPI00217259	515	518	PKC->			56155	TEX14	yilkndlkdfTgaqrtqptes
IPI00217324	482	483		unsp->INSR+unsp		23759	PPIL2	pqtfrqgvgkYinpaateqqr
IPI00217354	231	230	PKC->	PKC->		55738	ARFGAP1	rfasaakegaTkfgsqasqkf
IPI00217354	231	234	PKC+DNAPK->ATM+DNAPK			55738	ARFGAP1	aakegatkfgSqasqkfwghk
IPI00217465	63	55	unsp->unsp+PKA	unsp->unsp+PKA		3006	HIST1H1C	tkavaaskerSgvslaalkka
IPI00217465	63	58	PKC->PKA	PKC->		3006	HIST1H1C	vaaskersgvSlaalkkalaa
IPI00217466	64	56	unsp->unsp+PKA	unsp->unsp+PKA		3007	HIST1H1D	tkavaaskerSgvslaalkka
IPI00217466	64	59	PKC->PKA	PKC->		3007	HIST1H1D	vaaskersgvSlaalkkalaa
IPI00217467	46	51	unsp+PKC+cdc2->unsp+CKI+PKC+cdc2	unsp+PKC+cdc2->unsp+cdc2		3008	HIST1H1E	selitkavaaSkersgvslaa
IPI00217467	63	55	unsp->unsp+PKA	unsp->unsp+PKA		3008	HIST1H1E	tkavaaskerSgvslaalkka
IPI00217467	63	58	PKC->PKA	PKC->		3008	HIST1H1E	vaaskersgvSlaalkkalaa
IPI00217467	90	86	PKC->PKA	PKC->PKA		3008	HIST1H1E	nnsriklglkSlvskgtlvqt
IPI00217467	90	89	->PKA			3008	HIST1H1E	riklglkslvSkgtlvqtkgt
IPI00217467	90	92	unsp+PKC->CKI+PKC	unsp+PKC->unsp+CKI+PKC		3008	HIST1H1E	lglkslvskgTlvqtkgtgas
IPI00217467	168	172	unsp+PKC+RSK->unsp+PKC		PHOsphoELM|PhosphoSite	3008	HIST1H1E	aaaagakkakSpkkakaakpk
IPI00217468	109	105	PKC->PKA	PKC->PKA		3009	HIST1H1B	tlvqtkgtgaSgsfklnkkaa
IPI00217468	109	107	unsp+PKC->PKC	unsp+PKC->PKC		3009	HIST1H1B	vqtkgtgasgSfklnkkaasg
IPI00217468	168	173		unsp+PKC+GSK3+cdk5->unsp+PKC+GSK3+cdk5+p3	PHOsphoELM|PhosphoSite	3009	HIST1H1B	aaagvkkvakSpkkakaaakp
IPI00217469	88	89	->PKC+PKA	->PKC+PKA		3024	HIST1H1A	nnsriklgikSlvskgtlvqt
IPI00217469	88	92	unsp->			3024	HIST1H1A	riklgikslvSkgtlvqtkgt
IPI00217469	93	89	->PKA	->PKA		3024	HIST1H1A	nnsriklgikSlvskgtlvqt
IPI00217469	93	92	unsp->unsp+PKA			3024	HIST1H1A	riklgikslvSkgtlvqtkgt
IPI00217469	93	95	unsp+PKC->CKI+PKC	unsp+PKC->unsp+CKI+PKC		3024	HIST1H1A	lgikslvskgTlvqtkgtgas
IPI00217477	12	14	unsp+PKG+PKA->PKG	unsp+PKG+PKA->unsp+PKG		3149	HMGB3	gdpkkpkgkmSayaffvqtcr
IPI00217477	59	58	unsp->unsp+PKA			3149	HMGB3	rwktmsgkekSkfdemakadk
IPI00217477	145	144		PKC->ATM		3149	HMGB3	lndsekqpyiTkaaklkekye
IPI00217477	145	153	unsp->INSR+unsp			3149	HMGB3	itkaaklkekYekdvadyksk
IPI00217660	113	112		PKC->PKC+DNAPK		84148	MYST1	ewvdknrlalTktvkdavqkn
IPI00217660	238	241	unsp->	unsp->		84148	MYST1	qwrqppgkeiYrksnisvyev
IPI00217686	20	16		->EGFR		117246	FTSJ3	kvgksrrdkfYhlaketgyrs
IPI00217686	20	22	->PKC			117246	FTSJ3	rdkfyhlakeTgyrsrsafkl
IPI00217686	20	24	unsp->			117246	FTSJ3	kfyhlaketgYrsrsafkliq
IPI00217773	701	697	PKC->	PKC->		26009	ZZZ3	lgnrtakqvaSrvqkyfiklt
IPI00217773	725	724		PKC->ATM+PKC+DNAPK		26009	ZZZ3	pgrtpnlyiySkksstsrrqh
IPI00217773	725	727	unsp->unsp+PKC			26009	ZZZ3	tpnlyiyskkSstsrrqhpln
IPI00217773	725	729	unsp+PKC->PKC	unsp+PKC->PKC		26009	ZZZ3	nlyiyskkssTsrrqhplnkh
IPI00217773	726	727	unsp->unsp+PKC	unsp->unsp+PKC		26009	ZZZ3	tpnlyiyskkSstsrrqhpln
IPI00217773	726	729	unsp+PKC->PKC+cdc2	unsp+PKC->PKC		26009	ZZZ3	nlyiyskkssTsrrqhplnkh
IPI00217773	738	744	cdc2->PKA+cdc2			26009	ZZZ3	hplnkhlfkpStfmtsheppv
IPI00217871	402	409	->PKA			8659	ALDH4A1	rikkwleharSspsltilagg
IPI00217872	34	38	PKC->cdc2	PKC->PKC+cdc2		5236	PGM1	apyhdqkpgtSglrkktyyfe
IPI00217872	475	471	cdc2->			5236	PGM1	kdlealmfdrSfvgkqfsand
IPI00217872	475	478	unsp->	unsp->		5236	PGM1	fdrsfvgkqfSandkvytvek
IPI00217920	50	56	unsp+PKA->unsp+cdc2+PKA			126133	ALDH16A1	gkwlkpehrnSvpcqdpitge
IPI00217950	82	77	PKC->	PKC->		3151	HMGN2	nnpaengdakTdqaqkaegag
IPI00217963	162	165	unsp->GSK3+cdc2	unsp->cdc2		3868	KRT16	rqrpseikdySpyfktiedlr
IPI00217975	33	28	unsp+PKC+PKA->unsp+PKA	unsp+PKC+PKA->unsp+PKA	Uniprot|PHOsphoELM	4001	LMNB1	pttplsptrlSrlqekeelre
IPI00217975	123	126	cdc2+PKA->PKG+cdc2+PKA			4001	LMNB1	qlllnyakkeSdlngaqiklr
IPI00217975	124	126	cdc2+PKA->cdc2	cdc2+PKA->cdc2		4001	LMNB1	qlllnyakkeSdlngaqiklr
IPI00217975	157	158		unsp+CKI->CKI		4001	LMNB1	alatalgdkkSlegdledlkd
IPI00217975	483	478	PKC->	PKC->		4001	LMNB1	gwemirkigdTsvsykytsry
IPI00217975	483	479	PKC+cdc2->unsp+PKA+cdc2	PKC+cdc2->unsp+PKA+cdc2		4001	LMNB1	wemirkigdtSvsykytsryv
IPI00217975	483	481	unsp+PKC+cdc2->cdc2	unsp+PKC+cdc2->cdc2		4001	LMNB1	mirkigdtsvSykytsryvlk
IPI00217975	483	485		PKC->unsp+PKC		4001	LMNB1	igdtsvsykyTsryvlkagqt
IPI00218130	316	315	unsp+PKC->PKC+PKA	unsp+PKC->ATM+unsp		5837	PYGM	lqdiirrfksSkfgcrdpvrt
IPI00218187	147	144	->EGFR	->EGFR		5501	PPP1CC	ygfydeckrrYniklwktftd
IPI00218240	779	778	unsp+PKC->unsp+PKA	unsp+PKC->ATM+unsp+DNAPK		7468	WHSC1	hasnpsnrprSkgkmmrcvrc
IPI00218240	781	778	unsp+PKC->unsp+PKA	unsp+PKC->unsp+PKA		7468	WHSC1	hasnpsnrprSkgkmmrcvrc
IPI00218342	56	49	->cdc2			4522	MTHFD1	ailqvgnrddSnlyinvklka
IPI00218342	553	551	PKC->	PKC->		4522	MTHFD1	lrkitigqapTekghtrtaqf
IPI00218343	40	38	PKC->	PKC->		84790	TUBA1C	hgiqpdgqmpSdktigggdds
IPI00218343	40	41	->CKI	->unsp	Uniprot	84790	TUBA1C	qpdgqmpsdkTigggddsfnt
IPI00218343	60	56	PKC->	PKC->		84790	TUBA1C	ddsfntffseTgagkhvprav
IPI00218343	112	109	unsp->			84790	TUBA1C	aannyarghyTigkeiidlvl
IPI00218343	163	158	unsp+cdc2->unsp+PKA		PhosphoSite	84790	TUBA1C	gftsllmerlSvdygkkskle
IPI00218343	163	165	unsp+PKC+PKG+CKII->unsp+PKC+CKII	unsp+PKC+PKG+CKII->unsp+CKII	PhosphoSite	84790	TUBA1C	erlsvdygkkSklefsiypap
IPI00218343	164	165	unsp+PKC+PKG+CKII->unsp+PKC+CKII	unsp+PKC+PKG+CKII->unsp+PKC+CKII	PhosphoSite	84790	TUBA1C	erlsvdygkkSklefsiypap
IPI00218343	280	282	EGFR->unsp+EGFR	EGFR->unsp+EGFR		84790	TUBA1C	yapvisaekaYheqltvaeit
IPI00218343	311	312		->INSR		84790	TUBA1C	mvkcdprhgkYmaccllyrgd
IPI00218343	401	399	unsp->			84790	TUBA1C	arldhkfdlmYakrafvhwyv
IPI00218371	176	179	PKA->	PKA->PKC		221823	PRPS1L1	spdaggakrvTsiadqlnvdf
IPI00218371	176	180	unsp+PKA+RSK->unsp+PKA			221823	PRPS1L1	pdaggakrvtSiadqlnvdfa
IPI00218448	12	10	unsp+PKC->	unsp+PKC->PKA		3015	H2AFZ	-maggkagkdSgkaktkavsr
IPI00218448	14	10	unsp+PKC->unsp+PKA	unsp+PKC->unsp+PKC+PKA		3015	H2AFZ	-maggkagkdSgkaktkavsr
IPI00218448	14	15		PKC->unsp+PKC		3015	H2AFZ	kagkdsgkakTkavsrsqrag
IPI00218448	14	19	unsp->			3015	H2AFZ	dsgkaktkavSrsqraglqfp
IPI00218465	529	525	PKC->	PKC->		9373	PLAA	dpftgnsayrSaasktmniyf
IPI00218465	529	528	unsp+PKC->unsp	unsp+PKC->ATM+unsp+DNAPK		9373	PLAA	tgnsayrsaaSktmniyfpkk
IPI00218488	31	29	PKC->	PKC->		2779	GNAT1	lkedaekdarTvkllllgage
IPI00218488	31	40	unsp+PKC->unsp+PKC+cdc2	unsp+PKC->unsp+PKC+cdc2		2779	GNAT1	vkllllgageSgkstivkqmk
IPI00218591	38	37	unsp->		PhosphoSite	6426	SFRS1	irtkdiedvfYkygairdidl
IPI00218591	179	178		PKC->PKC+DNAPK		6426	SFRS1	mtyavrkldnTkfrshefcls
IPI00218624	16	11	PKC->	PKC->		6651	SON	matnieqifrSfvvskfreiq
IPI00218624	16	15	unsp+PKC->unsp	unsp+PKC->ATM+unsp		6651	SON	ieqifrsfvvSkfreiqqels
IPI00218624	288	282		unsp+CKII->unsp+cdc2+CKII		6651	SON	mksvlksvesTspepskimlv
IPI00218624	288	287	unsp->unsp+PKA	unsp->ATM+unsp+DNAPK		6651	SON	ksvestspepSkimlveppva
IPI00218624	2095	2091	unsp+cdc2->unsp	unsp+cdc2->cdc2		6651	SON	sergrspkrlTdldkaqllei
IPI00218728	53	47	INSR->	INSR->		5048	PAFAH1B1	ldvneeldkkYagllekkwts
IPI00218728	53	57	unsp+PKC->			5048	PAFAH1B1	yagllekkwtSvirlqkkvme
IPI00218748	76	79	unsp->			1415	CRYBB2	geqfvfekgeYprwdswtssr
IPI00218748	76	84	unsp+PKA->unsp			1415	CRYBB2	fekgeyprwdSwtssrrtdsl
IPI00218748	120	118	PKC->	PKC->	niprot|PHOsphoELM|PhosphoSi	1415	CRYBB2	kiilyenpnfTgkkmeiiddd
IPI00218748	121	118	PKC->		Uniprot|PHOsphoELM|PhosphoSi	1415	CRYBB2	kiilyenpnfTgkkmeiiddd
IPI00218823	2296	2295	unsp->			9757	MLL4	sgrsppapppYkaprldedge
IPI00218829	108	105	PKC->	PKC->		2935	GSPT1	myltgmvdkrTlekyereake
IPI00218829	196	192		unsp+CKII->CKII		2935	GSPT1	visarkgefeTgfekggqtre
IPI00218829	196	200	unsp->	unsp->		2935	GSPT1	fetgfekggqTrehamlakta
IPI00218829	469	467	unsp+PKC->PKC	unsp+PKC->PKC		2935	GSPT1	lrtagticleTfkdfpqmgrf
IPI00218831	126	127	unsp->	unsp->		2944	GSTM1	npefeklkpkYleelpeklkl
IPI00218836	36	35	PKC->	PKC->		1622	DBI	kaaeevrhlkTkpsdeemlfi
IPI00218836	36	38	unsp+PKG+CKII->unsp+CKII	unsp+PKG+CKII->unsp+CKII		1622	DBI	eevrhlktkpSdeemlfiygh
IPI00218836	72	68	PKC->			1622	DBI	nterpgmldfTgkakwdawne
IPI00218836	94	91		unsp->		1622	DBI	gtskedamkaYinkveelkkk
IPI00218914	128	129		PKC->unsp+PKC		216	ALDH1A1	ylndlagcikTlrycagwadk
IPI00218914	419	413	unsp->unsp+PKA			216	ALDH1A1	gpvqqimkfkSlddvikrann
IPI00218914	435	434		->DNAPK		216	ALDH1A1	tfyglsagvfTkdidkaitis
IPI00218914	495	497	ATM+unsp->ATM+unsp+PKC			216	ALDH1A1	tevktvtvkiSqkns------
IPI00218914	495	501	->PKA			216	ALDH1A1	tvtvkisqknS----------
IPI00218918	239	241	PKC+cdc2->PKC			301	ANXA1	pqlrrvfqkyTkyskhdmnkv
IPI00218918	239	243	unsp->	unsp->		301	ANXA1	lrrvfqkytkYskhdmnkvld
IPI00218918	239	244	unsp+PKG->unsp			301	ANXA1	rrvfqkytkySkhdmnkvldl
IPI00218918	242	241		PKC+cdc2->PKC		301	ANXA1	pqlrrvfqkyTkyskhdmnkv
IPI00218918	242	243	unsp->	unsp->		301	ANXA1	lrrvfqkytkYskhdmnkvld
IPI00218918	242	244	unsp+PKG->unsp+PKC	unsp+PKG->unsp		301	ANXA1	rrvfqkytkySkhdmnkvldl
IPI00218918	250	241		PKC+cdc2->PKC		301	ANXA1	pqlrrvfqkyTkyskhdmnkv
IPI00218918	312	304	unsp+CKII->unsp+cdc2+CKII			301	ANXA1	kalirimvsrSeidmndikaf
IPI00218918	312	315	EGFR->			301	ANXA1	eidmndikafYqkmygislcq
IPI00218922	527	523		unsp+PKC+PKG->PKC+PKG		11231	SEC63	wqqkskgpkkTakskkkkplk
IPI00218971	202	201	unsp+PKC->unsp	unsp+PKC->ATM+unsp+DNAPK		54704	PDP1	qwhkhpndyfSkeasklyfns

IPI00219005	274	270	cdc2->	cdc2->unsp		2288	FKBP4	emnseekleqStivkergtvy
IPI00219005	274	271	unsp->	unsp->		2288	FKBP4	mnseekleqsTivkergtvyf
IPI00219005	274	278	unsp+PKC->	unsp+PKC->PKC		2288	FKBP4	eqstivkergTvyfkegkykq
IPI00219005	282	278	unsp+PKC->unsp+PKA	unsp+PKC->PKA		2288	FKBP4	eqstivkergTvyfkegkykq
IPI00219018	61	59	unsp+PKC->PKC	unsp+PKC->PKC		2597	GAPDH	ydsthgkfhgTvkaengklvi
IPI00219018	117	122	PKA->	PKA->PKG	Uniprot	2597	GAPDH	lqggakrviiSapsadapmfv
IPI00219018	186	184		DNAPK->ATM+DNAPK	PhosphoSite	2597	GAPDH	lmttvhaitaTqktvdgpsgk
IPI00219018	186	187		->unsp		2597	GAPDH	tvhaitatqkTvdgpsgklwr
IPI00219018	194	192	unsp->	unsp->		2597	GAPDH	tatqktvdgpSgklwrdgrga
IPI00219018	334	333	unsp->PKA	unsp->ATM+unsp+DNAPK		2597	GAPDH	rvvdlmahmaSke--------
IPI00219037	6	2	PKC->		Uniprot	3014	H2AFX	---------mSgrgktggkar
IPI00219037	6	7	unsp->unsp+PKC	unsp->unsp+PKC		3014	H2AFX	----msgrgkTggkarakaks
IPI00219037	10	7	unsp->unsp+PKA			3014	H2AFX	----msgrgkTggkarakaks
IPI00219037	119	121	PKG->	PKG->	niprot|PHOsphoELM|PhosphoSi	3014	H2AFX	niqavllpkkTsatvgpkaps
IPI00219038	10	12	unsp+PKC+PKG->PKC	unsp+PKC+PKG->unsp+PKC	Uniprot	644914	LOC644914	artkqtarksTggkaprkqla
IPI00219038	15	11		unsp->unsp+PKG	niprot|PHOsphoELM|PhosphoSi	644914	LOC644914	martkqtarkStggkaprkql
IPI00219038	15	12	unsp+PKC+PKG->PKG+PKA	unsp+PKC+PKG->PKC+PKG	Uniprot	644914	LOC644914	artkqtarksTggkaprkqla
IPI00219038	24	23		PKC->PKC+DNAPK		644914	LOC644914	ggkaprkqlaTkaarksapst
IPI00219038	28	32	unsp+CKI+PKC->unsp+CKI		PHOsphoELM|PhosphoSite	644914	LOC644914	atkaarksapStggvkkphry
IPI00219038	37	32	unsp+CKI+PKC->unsp+CKI		PHOsphoELM|PhosphoSite	644914	LOC644914	atkaarksapStggvkkphry
IPI00219038	57	58	unsp->	unsp->		644914	LOC644914	alreirryqkStellirklpf
IPI00219068	233	236	unsp->	unsp->		122830	NAA30	dimrlitkdlSepysiytyry
IPI00219072	296	292		->EGFR		6794	STK11	lsdllkgmleYepakrfsirq
IPI00219072	311	307	unsp+PKC->unsp+PKA+RSK	unsp+PKC->unsp+PKC+RSK	PhosphoSite	6794	STK11	rfsirqirqhSwfrkkhppae
IPI00219072	416	419	PKC->			6794	STK11	rapnparkacSasskirrlsa
IPI00219072	416	421	unsp->	unsp->		6794	STK11	pnparkacsaSskirrlsack
IPI00219072	416	422	unsp+PKC->unsp+PKC+cdc2	unsp+PKC->unsp+PKC+cdc2		6794	STK11	nparkacsasSkirrlsackq
IPI00219072	423	419	PKC->	PKC->unsp		6794	STK11	rapnparkacSasskirrlsa
IPI00219072	423	421	unsp->	unsp->		6794	STK11	pnparkacsaSskirrlsack
IPI00219072	423	422	unsp+PKC->PKC	unsp+PKC->unsp+PKC+DNAPK		6794	STK11	nparkacsasSkirrlsackq
IPI00219072	423	428		unsp+PKC+PKG+PKA+RSK->unsp+PKG+PKA+RSK	niprot|PHOsphoELM|PhosphoSi	6794	STK11	csasskirrlSackqq-----
IPI00219072	431	422	unsp+PKC->unsp+PKC+cdc2			6794	STK11	nparkacsasSkirrlsackq
IPI00219072	431	428	unsp+PKC+PKG+PKA+RSK->unsp+PKG+PKA+RSK	unsp+PKC+PKG+PKA+RSK->unsp+PKG+PKA+RSK	niprot|PHOsphoELM|PhosphoSi	6794	STK11	csasskirrlSackqq-----
IPI00219077	73	74	unsp->	unsp->		4048	LTA4H	kvvingqevkYalgerqsykg
IPI00219077	414	419	unsp+PKA->CKI	unsp+PKA->		4048	LTA4H	kayvekfsykSittddwkdfl
IPI00219077	573	568	PKC->PKG			4048	LTA4H	mateqgrmkfTrplfkdlaaf
IPI00219078	464	463		unsp+PKA->unsp		488	ATP2A2	nvfdtelkglSkieranacns
IPI00219097	12	14	unsp+PKG+PKA->	unsp+PKG+PKA->unsp		3148	HMGB2	gdpnkprgkmSsyaffvqtcr
IPI00219097	12	16	unsp->	unsp->		3148	HMGB2	pnkprgkmssYaffvqtcree
IPI00219097	30	35	unsp+PKC->	unsp+PKC->cdc2	Uniprot|PHOsphoELM	3148	HMGB2	eehkkkhpdsSvnfaefskkc
IPI00219097	43	42	unsp+PKC->unsp+CKI+PKC	unsp+PKC->ATM+unsp+PKC+DNAPK		3148	HMGB2	pdssvnfaefSkkcserwktm
IPI00219097	44	35	unsp+PKC->unsp+PKC+cdc2		Uniprot|PHOsphoELM	3148	HMGB2	eehkkkhpdsSvnfaefskkc
IPI00219097	44	42	unsp+PKC->	unsp+PKC->		3148	HMGB2	pdssvnfaefSkkcserwktm
IPI00219097	59	58	unsp+CKII->unsp+PKA			3148	HMGB2	rwktmsakekSkfedmaksdk
IPI00219097	139	137	unsp+PKC->	unsp+PKC->		3148	HMGB2	kklgemwseqSakdkqpyeqk
IPI00219097	157	155	unsp->		Uniprot|PhosphoSite	3148	HMGB2	eqkaaklkekYekdiaayrak
IPI00219097	173	168		unsp->		3148	HMGB2	diaayrakgkSeagkkgpgrp
IPI00219097	182	181		unsp+PKC->ATM+unsp+PKC+DNAPK		3148	HMGB2	gkkgpgrptgSkkknepedee
IPI00219155	93	86		unsp+cdc2+PKA->unsp+PKA		6155	RPL27	nynhlmptrySvdipldktvv
IPI00219155	98	94	cdc2->			6155	RPL27	rysvdipldkTvvnkdvfrdp
IPI00219155	128	124	unsp+PKC->unsp			6155	RPL27	akvkfeerykTgknkwffqkl
IPI00219160	36	44	unsp->unsp+cdc2			6164	RPL34	ytkkvgkapkSacgvcpgrlr
IPI00219160	37	44	unsp->unsp+cdc2			6164	RPL34	ytkkvgkapkSacgvcpgrlr
IPI00219179	289	293	PKC->			2072	ERCC4	slvqdlkilrTllqylsqydc
IPI00219217	82	85	unsp+PKC->	unsp+PKC->PKC		3945	LDHB	tpkivadkdySvtanskivvv
IPI00219217	318	320	PKG->	PKG->		3945	LDHB	kddevaqlkkSadtlwdiqkd
IPI00219217	319	320	PKG->	PKG->		3945	LDHB	kddevaqlkkSadtlwdiqkd
IPI00219219	29	30	->cdc2	->cdc2		3956	LGALS1	vrgevapdakSfvlnlgkdsn
IPI00219299	1543	1544	unsp->unsp+PKC	unsp->unsp+PKC		83660	TLN2	vanstanlvkTikaldgdfse
IPI00219306	114	113	CKII->CKII+DNAPK	CKII->CKII+DNAPK		4116	MAGOH	kigslidvnqSkdpeglrvfy
IPI00219365	79	74	unsp+PKA+p38MAPK+RSK->unsp+PKA+p38MAPK			4478	MSN	kvtaqdvrkeSpllfkfrakf
IPI00219365	139	144	PKA->			4478	MSN	ygdfnkevhkSgylagdkllp
IPI00219365	209	207	unsp+PKC+cdc2->cdc2	unsp+PKC+cdc2->cdc2		4478	MSN	dlemygvnyfSiknkkgselw
IPI00219365	209	214	unsp+PKA->PKA	unsp+PKA->PKA		4478	MSN	nyfsiknkkgSelwlgvdalg
IPI00219365	211	205		INSR->		4478	MSN	aqdlemygvnYfsiknkkgse
IPI00219365	211	207	unsp+PKC+cdc2->unsp			4478	MSN	dlemygvnyfSiknkkgselw
IPI00219365	211	214	unsp+PKA->PKA	unsp+PKA->PKA		4478	MSN	nyfsiknkkgSelwlgvdalg
IPI00219365	254	249	unsp+RSK->unsp	unsp+RSK->unsp		4478	MSN	gfpwseirniSfndkkfvikp
IPI00219365	388	384	unsp+PKG+PKB+CKII+PKA+RSK->unsp+PKB+CKII+PKA+RSK			4478	MSN	eleqerkraqSeaeklakerq
IPI00219368	343	339	PKC->unsp	PKC->unsp		7090	TLE3	pgtsttpglrSmpgkppgmdp
IPI00219381	64	59	->PKA			4695	NDUFA2	pdlpilirecSdvqpklwary
IPI00219383	23	19	unsp->	unsp->unsp+EGFR		4709	NDUFB3	hghhkmelpdYrqwkiegtpl
IPI00219420	105	109	unsp->	unsp->		9126	SMC3	rrvigakkdqYfldkkmvtkn
IPI00219420	106	109	unsp->	unsp->		9126	SMC3	rrvigakkdqYfldkkmvtkn
IPI00219420	114	117		->PKC		9126	SMC3	dqyfldkkmvTkndvmnlles
IPI00219420	140	131	unsp+cdc2->unsp+PKA+cdc2	unsp+cdc2->unsp+PKA+cdc2		9126	SMC3	vmnllesagfSrsnpyyivkq
IPI00219420	140	133	->cdc2			9126	SMC3	nllesagfsrSnpyyivkqgk
IPI00219420	140	136	INSR+unsp->INSR			9126	SMC3	esagfsrsnpYyivkqgkinq
IPI00219420	140	137		INSR+unsp->INSR+unsp+EGFR		9126	SMC3	sagfsrsnpyYivkqgkinqm
IPI00219420	1190	1187		->EGFR		9126	SMC3	pellesadkfYgvkfrnkvsh
IPI00219445	208	204	PKC->unsp	PKC->unsp+PKC		10197	PSME3	ryyitraklvSkiakyphved
IPI00219497	601	602	unsp->SRC	unsp->		4602	MYB	khalaaqeikYgplkmlpqtp
IPI00219525	38	37	unsp->unsp+PKA	unsp->unsp+DNAPK		5226	PGD	fvvcafnrtvSkvddflanea
IPI00219525	59	50		unsp+cdc2->unsp		5226	PGD	ddflaneakgTkvvgaqslke
IPI00219525	59	57	unsp+PKC->CKII	unsp+PKC->		5226	PGD	akgtkvvgaqSlkemvsklkk
IPI00219525	154	155		->unsp		5226	PGD	gnkeawphikTifqgiaakvg
IPI00219532	100	94		PKA+RSK->PKA		9844	ELMO1	naqqlheriqSssmdakleal
IPI00219532	100	95	unsp+PKC+cdc2->unsp+cdc2	unsp+PKC+cdc2->unsp+cdc2		9844	ELMO1	aqqlheriqsSsmdakleakl
IPI00219532	100	96	unsp+PKC+cdc2+CKII->unsp+CKII			9844	ELMO1	qqlheriqssSmdaklealkd
IPI00219532	105	96		unsp+PKC+cdc2+CKII->unsp+PKC+CKII		9844	ELMO1	qqlheriqssSmdaklealkd
IPI00219532	105	109	unsp+PKC->	unsp+PKC->unsp+PKC+cdc2		9844	ELMO1	aklealkdlaSlsrdvtfaqe
IPI00219553	674	678	unsp+PKB+PKA+RSK->PKA+RSK			30811	HUNK	igqmlrkrhqSlqpsadrple
IPI00219563	972	969	unsp+cdc2->	unsp+cdc2->unsp		23236	PLCB1	ylrrraalekSakkdskkkse
IPI00219563	976	969		unsp+cdc2->unsp		23236	PLCB1	ylrrraalekSakkdskkkse
IPI00219563	976	974	unsp+PKC+cdc2->unsp+cdc2	unsp+PKC+cdc2->unsp+cdc2		23236	PLCB1	aaleksakkdSkkksepsspd
IPI00219563	976	978		unsp+PKC->unsp	PhosphoSite	23236	PLCB1	ksakkdskkkSepsspdhgss
IPI00219568	91	87	cdc2->			5232	PGK2	slapvavelkSllgkdvlflk
IPI00219568	156	153	unsp+PKA+RSK+DNAPK->PKA+DNAPK	unsp+PKA+RSK+DNAPK->unsp+PKA+DNAPK		5232	PGK2	epdkieafraSlsklgdvyvn
IPI00219568	156	155		->ATM+DNAPK		5232	PGK2	dkieafraslSklgdvyvnda
IPI00219575	391	390	unsp+PKC->unsp	unsp+PKC->unsp		642	BLMH	sekddqdgafTkwrvenswge
IPI00219622	70	61	->cdc2			5683	PSMA2	kqksilyderSvhkvepitkh
IPI00219678	141	142	INSR+unsp->unsp			1965	EIF2S1	qrtawvfddkYkrpgygayda
IPI00219691	180	178	SRC+unsp->SRC			51621	KLF13	kchyagcekvYgksshlkahl
IPI00219691	225	222	unsp+PKC->			51621	KLF13	delarhyrthTgekkfscpic
IPI00219691	225	228	->cdc2	->cdc2		51621	KLF13	yrthtgekkfScpicekrfmr
IPI00219691	226	222	unsp+PKC->unsp	unsp+PKC->		51621	KLF13	delarhyrthTgekkfscpic
IPI00219691	226	228	->CKI	->CKI		51621	KLF13	yrthtgekkfScpicekrfmr
IPI00219695	38	32	INSR->	INSR->		5771	PTPN2	yleirneshdYphrvakfpen
IPI00219695	312	310	PKC->	PKC->		5771	PTPN2	afdhspnkimTekyngnrigl
IPI00219729	73	70	PKC+cdc2->cdc2			8402	SLC25A11	eyktsfhaltSilkaeglrgi
IPI00219757	128	135	DNAPK->ATM+DNAPK			2950	GSTP1	gqlkpfetllSqnqggktfiv
IPI00219757	191	196		->p38MAPK		2950	GSTP1	arpklkaflaSpeyvnlping
IPI00219894	22	24	->PKC			140032	RPS4Y2	apkhwmldklTgvfaprpstg
IPI00219913	291	288	PKC->	PKC->		9097	USP14	finqevkylfTglklrlqeei
IPI00219913	291	299	->cdc2	->cdc2		9097	USP14	glklrlqeeiTkqsptlqrna
IPI00219913	313	304	->cdc2			9097	USP14	lqeeitkqspTlqrnalyiks
IPI00219913	313	311	unsp->			9097	USP14	qsptlqrnalYiksskisrlp
IPI00219913	313	318	unsp+PKG+PKA->PKG	unsp+PKG+PKA->PKG		9097	USP14	nalyiksskiSrlpayltiqm
IPI00219913	449	456	unsp+CKII->unsp+cdc2+CKII			9097	USP14	ewikfdddkvSivtpedilrl
IPI00219919	28	24	PKC->	PKC->		55929	DMAP1	ggpegdaasgTiskkdiinpd
IPI00219919	28	26	unsp+PKC->unsp	unsp+PKC->unsp		55929	DMAP1	pegdaasgtiSkkdiinpdkk
IPI00219919	35	38	unsp+PKC+PKA->unsp+PKC+PKG	unsp+PKC+PKA->unsp+PKC+PKG		55929	DMAP1	kdiinpdkkkSkkssetltfk
IPI00219919	36	38	unsp+PKC+PKA->unsp+PKC	unsp+PKC+PKA->unsp+PKC		55929	DMAP1	kdiinpdkkkSkkssetltfk
IPI00219919	36	41	unsp+PKA->unsp			55929	DMAP1	inpdkkkskkSsetltfkrpe
IPI00219919	37	41		unsp+PKA->unsp		55929	DMAP1	inpdkkkskkSsetltfkrpe
IPI00219919	37	42	unsp+PKA->unsp			55929	DMAP1	npdkkkskksSetltfkrpeg
IPI00219953	87	79		unsp+DNAPK->ATM+unsp+DNAPK		51727	CMPK1	llrderknpdSqygeliekyi
IPI00220014	233	230	unsp->	unsp->		3422	IDI1	pdpneiksycYvskeelkell

Table S3

IPI00220014	233	232		unsp+CKII->ATM+unsp+CKII+DNAPK		3422	IDI1	pneiksycyvSkeelkellkk
IPI00220219	318	320	unsp->			9276	COPB2	angkiiwakhSevqqanlkam
IPI00220289	186	181	unsp+PKC+RSK->unsp+PKC+PKG			84181	CHD6	sctdsaartkSrkaskeqgpt
IPI00220289	186	185	unsp+PKG+PKA+RSK->unsp+CKI+PKA+RSK	unsp+PKG+PKA+RSK->ATM+unsp+PKG+PKA+DNAPK+RSK		84181	CHD6	saartksrkaSkeqgptpvek
IPI00220301	209	208		->ATM+DNAPK		9588	PRDX6	akklfpkgvfTkelpsgkkyl
IPI00220360	8	6	unsp+PKC->	unsp+PKC->		6638	SNRPN	-----mtvgkSskmlqhidyr
IPI00220360	8	7	unsp+PKC+PKA->PKA	unsp+PKC+PKA->unsp+PKA+DNAPK		6638	SNRPN	----mtvgksSkmlqhidyrm
IPI00220360	32	30	unsp+PKC->PKC	unsp+PKC->PKC		6638	SNRPN	ilqdgrifigTfkafdkhmnl
IPI00220362	56	51	PKC->			3336	HSPE1	vlqatvvavgSgskgkggeiq
IPI00220362	56	53	unsp+CKI+PKC->CKI	unsp+CKI+PKC->CKI+PKC		3336	HSPE1	qatvvavgsgSkgkggeiqpv
IPI00220362	99	100	unsp->	unsp->		3336	HSPE1	lfrdgdilgkYvd--------
IPI00220373	425	433	->INSR	->INSR		3416	IDE	rfkdkerprgYtskiagilhy
IPI00220403	6	5		->DNAPK		3018	HIST1H2BB	------mpepSksapakkgs
IPI00220403	6	7	unsp->	unsp->		3018	HIST1H2BB	----mpepskSapakkgskk
IPI00220403	12	7	unsp->	unsp->		3018	HIST1H2BB	----mpepskSapakkgskk
IPI00220403	16	15	unsp+PKC->unsp+PKC+PKA	unsp+PKC->ATM+unsp+PKC+DNAPK	niprot|PHOsphoELM|PhosphoSi	3018	HIST1H2BB	sksapakkgSkkaitkaqkk
IPI00220403	17	15	unsp+PKC->PKC	unsp+PKC->PKC	niprot|PHOsphoELM|PhosphoSi	3018	HIST1H2BB	sksapakkgSkkaitkaqkk
IPI00220403	47	53		GSK3->		3018	HIST1H2BB	ykvlkqvhpdTgisskamgim
IPI00220403	109	113	unsp->			3018	HIST1H2BB	lpgelakhavSegtkavtkyt
IPI00220403	121	120		->ATM+DNAPK		3018	HIST1H2BB	havsegtkavTkytssk----
IPI00220403	121	123		PKC->		3018	HIST1H2BB	segtkavtkyTssk-------
IPI00220416	12	8		->unsp		7381	UQCRB	---magkqavSasgkwldgir
IPI00220416	12	10	unsp->	unsp->PKA		7381	UQCRB	-magkqavsaSgkwldgirkw
IPI00220477	136	135	PKG+PKA->PKA			10445	MCRS1	apgltkrvkkSkqplqvtkdl
IPI00220484	82	76	unsp+PKC->unsp+PKC+PKA	unsp+PKC->unsp+PKC+PKA		10473	HMGN4	gnnpaknrdaStlqsqkaegt
IPI00220484	82	80	unsp+PKC->PKC	unsp+PKC->PKC+DNAPK	Uniprot|PhosphoSite	10473	HMGN4	aknrdastlqSqkaegtgdak
IPI00220484	82	86	unsp+PKC->CKII			10473	HMGN4	stlqsqkaegTgdak------
IPI00220486	418	419	unsp+PKC->unsp+PKC+CKII	unsp+PKC->unsp+PKC+CKII		10474	TADA3	tkkekdqawkTlkeresilkl
IPI00220487	95	100	unsp+CKII->CKII	unsp+CKII->CKII		10476	ATP5H	vdaeekedvkScaewvslska
IPI00220487	148	143	PKC->	PKC->		10476	ATP5H	iedlneafpeTkldkkkypyw
IPI00220487	149	150	unsp->	unsp->		10476	ATP5H	fpetkldkkkYpywphqpien
IPI00220642	120	115	unsp+CKII+DNAPK->unsp+DNAPK			7532	YWHAG	dnylikncseTqyeskvfylk
IPI00220642	120	119		->DNAPK		7532	YWHAG	ikncsetqyeSkvfylkmkgd
IPI00220642	120	123	unsp->	unsp->		7532	YWHAG	setqyeskvfYlkmkgdyyry
IPI00220656	295	301	->CKI			10693	CCT6B	vinqkgidpfSldslakhgiv
IPI00220710	103	104	unsp->	unsp->		23597	ACOT9	lgsepelrekYltvqntvrfg
IPI00220710	250	244	unsp->unsp+PKA	unsp->unsp+PKA		23597	ACOT9	elnkgrriafSstsllkmaps
IPI00220710	250	245	PKC->PKA	PKC->		23597	ACOT9	lnkgrriafsStsllkmapsa
IPI00220710	250	254	unsp->unsp+CKII			23597	ACOT9	sstsllkmapSaeerttihem
IPI00220710	407	408	unsp->unsp+PKC	unsp->unsp+PKC		23597	ACOT9	ekevplvfpkTygesmlyldg
IPI00220710	407	412	PKA->	PKA->		23597	ACOT9	plvfpktygeSmlyldgqrhf
IPI00220827	15	12	PKC->CKII	PKC->PKC+CKII		9168	TMSB10	adkpdmgeiaSfdkaklkkte
IPI00220827	17	21	PKC->	PKC->	PhosphoSite	9168	TMSB10	asfdkaklkkTetqekntlpt
IPI00220827	26	21	PKC->	PKC->	PhosphoSite	9168	TMSB10	asfdkaklkkTetqekntlpt
IPI00220827	26	23	PKC+DNAPK->DNAPK		Uniprot|PhosphoSite	9168	TMSB10	fdkaklkkteTqekntlptke
IPI00220827	26	28	->CKI	->CKI		9168	TMSB10	lkktetqeknTlptketieqe
IPI00220827	39	41	unsp->			9168	TMSB10	tketieqekrSeis-------
IPI00220827	39	44	PKA->	PKA->		9168	TMSB10	tieqekrseiS----------
IPI00220828	17	16		PKC->PKC+DNAPK		7114	TMSB4X	dmaeiekfdkSklkktetqek
IPI00220828	26	23	PKC+DNAPK->DNAPK		Uniprot|PhosphoSite	7114	TMSB4X	fdksklkkteTqeknplpske
IPI00220828	32	31		unsp->ATM+unsp+DNAPK		7114	TMSB4X	tetqeknplpSketieqekqa
IPI00220828	32	34		->unsp		7114	TMSB4X	qeknplpskeTieqekqages
IPI00220834	144	140	PKC->			7520	XRCC5	rhieiftdlsSrfsksqldii
IPI00220834	144	143		unsp+CKI+PKA->unsp+CKI+PKA+DNAPK		7520	XRCC5	eiftdlssrfSksqldiiihs
IPI00220834	144	145	ATM+unsp+cdc2->ATM+unsp+cdc2+DNAPK	ATM+unsp+cdc2->ATM+unsp+cdc2+DNAPK		7520	XRCC5	ftdlssrfskSqldiiihslk
IPI00220834	155	153	unsp+PKC->PKC+cdc2	unsp+PKC->DNAPK		7520	XRCC5	sksqldiiihSlkkcdislqf
IPI00220834	155	160	PKA->cdc2+PKA	PKA->cdc2+PKA		7520	XRCC5	iihslkkcdiSlqfflpfslg
IPI00220834	156	153	unsp+PKC->PKC+PKA	unsp+PKC->unsp+PKC+PKA		7520	XRCC5	sksqldiiihSlkkcdislqf
IPI00220834	156	160	PKA->cdc2+PKA	PKA->cdc2+PKA		7520	XRCC5	iihslkkcdiSlqfflpfslg
IPI00220834	195	191	->PKA			7520	XRCC5	gpfrlgghgpSfplkgiteqq
IPI00220834	265	258	unsp->unsp+cdc2		Uniprot	7520	XRCC5	pcrltigsnlSiriaayksil
IPI00220834	332	333	unsp->	unsp->		7520	XRCC5	fskvdeeqmkYksegkcfsvl
IPI00220834	338	335	->PKA			7520	XRCC5	kvdeeqmkykSegkcfsvlgf
IPI00220834	338	341	PKC+CKI->CKI+cdc2	PKC+CKI->PKC+CKI+cdc2		7520	XRCC5	mkyksegkcfSvlgfckssqv
IPI00220834	532	531		unsp+PKC->ATM+unsp+PKC+DNAPK		7520	XRCC5	evttksqiplSkiktlfplie
IPI00220871	10	5	PKC->	PKC->		6167	RPL37	------mtkgTssfgkrrnkt
IPI00220871	10	7	unsp+PKC->PKC+PKA			6167	RPL37	----mtkgtsSfgkrrnktht
IPI00220871	10	15	PKG+PKA->			6167	RPL37	tssfgkrrnkThtlcrrcgsk
IPI00220901	477	485	PKA->	PKA->		9882	TBC1D4	raklviqrhlSsltdneqadi
IPI00220906	104	99	unsp+PKC->unsp+PKA	unsp+PKC->unsp+PKC+PKA		10965	ACOT2	apeqpvtlraSlrdekgalfq
IPI00220994	12	11	unsp+PKC+cdc2+PKA->cdc2+PKA	unsp+PKC+cdc2+PKA->unsp+cdc2+DNAPK		55506	H2AFY2	msgrsgkkkmSklsrsaragv
IPI00220994	12	14	unsp+PKA->PKC	unsp+PKA->unsp		55506	H2AFY2	rsgkkkmsklSrsaragvifp
IPI00221088	155	160	unsp+PKC->PKC	unsp+PKC->PKC	PhosphoSite	6203	RPS9	rldsqkhidfSlrspygggrp
IPI00221089	27	24	PKC->			6207	RPS13	salpyrrsvpTwlkltsddvk
IPI00221089	27	29	unsp+CKII->unsp+CKI+PKC+CKII	unsp+CKII->unsp+CKI+CKII	PhosphoSite	6207	RPS13	rrsvptwlklTsddvkeqiyk
IPI00221089	39	38		unsp->unsp+EGFR	Uniprot|PhosphoSite	6207	RPS13	ltsddvkeqiYklakkgltps
IPI00221089	93	89	SRC+unsp->unsp			6207	RPS13	glapdlpedlYhlikkavavr
IPI00221091	124	121	PKC+PKG+PKA->PKA			6210	RPS15A	mdheearrkhTggkilgfff-
IPI00221093	19	20	INSR->			6218	RPS17	kkaarviiekYytrlgndfht
IPI00221093	19	21	INSR+unsp->unsp			6218	RPS17	kaarviiekyYtrlgndfhtn
IPI00221106	275	282	unsp+CKII->unsp+cdc2+CKII	unsp+CKII->unsp+cdc2+CKII		10992	SF3B2	alekilqlkeSrqeemnsqqe
IPI00221222	68	71	unsp->	unsp->		10923	SUB1	dnmfqigkmrYvsvrdfkgkv
IPI00221222	68	73	unsp+PKC+RSK->unsp+PKC	unsp+PKC+RSK->unsp+PKC		10923	SUB1	mfqigkmryvSvrdfkgkvli
IPI00221226	63	61	PKC->cdc2	PKC->		309	ANXA6	snrqrqevcqSykslygkdli
IPI00221226	63	62	INSR+unsp->	INSR+unsp->INSR+EGFR		309	ANXA6	nrqrqevcqsYkslygkdlia
IPI00221226	63	64	unsp->unsp+CKI	unsp->		309	ANXA6	qrqevcqsykSlygkdliadl
IPI00221226	68	61	PKC->PKC+cdc2			309	ANXA6	snrqrqevcqSykslygkdli
IPI00221226	68	62	INSR+unsp->unsp	INSR+unsp->unsp		309	ANXA6	nrqrqevcqsYkslygkdlia
IPI00221226	75	79	unsp+PKC->			309	ANXA6	dliadlkyelTgkferlivgl
IPI00221226	81	79	unsp+PKC->CKII	unsp+PKC->		309	ANXA6	dliadlkyelTgkferlivgl
IPI00221226	299	295	unsp+PKC->unsp	unsp+PKC->CKII		309	ANXA6	dmldireifrTkyekslysmi
IPI00221226	299	297	INSR+unsp->unsp	INSR+unsp->unsp		309	ANXA6	ldireifrtkYekslysmikn
IPI00221226	299	300	CKI+PKA->CKI+PKA+DNAPK	CKI+PKA->CKI+PKA+DNAPK		309	ANXA6	reifrtkyekSlysmikndts
IPI00221226	299	302	unsp->		PhosphoSite	309	ANXA6	ifrtkyekslYsmikndtsge
IPI00221226	299	303	unsp+PKC->			309	ANXA6	frtkyekslySmikndtsgey
IPI00221226	306	300	CKI+PKA->PKA	CKI+PKA->PKA		309	ANXA6	reifrtkyekSlysmikndts
IPI00221226	306	303	unsp+PKC->unsp			309	ANXA6	frtkyekslySmikndtsgey
IPI00221226	306	309	unsp+PKC+CKII->CKII			309	ANXA6	kslysmikndTsgeykktllk
IPI00221226	306	310		unsp->unsp+cdc2		309	ANXA6	slysmikndtSgeykktllkl
IPI00221226	418	415	PKC+CKII->CKII	PKC+CKII->CKII		309	ANXA6	fkshfgrdlmTdlkseisgdl
IPI00221226	418	422		CKII->cdc2+CKII		309	ANXA6	dlmtdlkseiSgdlarlilgl
IPI00221226	483	481	INSR+unsp+EGFR->unsp	INSR+unsp+EGFR->unsp+EGFR		309	ANXA6	raineaykedYhksledalss
IPI00221226	620	617	unsp+PKC->CKII	unsp+PKC->		309	ANXA6	klyksmkgagTdektltrimv
IPI00221226	620	623	PKC->			309	ANXA6	kgagtdektlTrimvsrseid
IPI00221325	123	128	GSK3+p38MAPK->p38MAPK		Uniprot	5903	RANBP2	leraaklfpgSpaiyklkeql
IPI00234252	345	343	unsp+PKC+PKG+cdc2+PKA+RSK->unsp+cdc2+PKA+RSK	unsp+PKC+PKG+cdc2+PKA+RSK->unsp+cdc2+PKA+RSK		6599	SMARCC1	pptptesrkkSgkkgqaslyg
IPI00234252	345	350	unsp+DNAPK->DNAPK			6599	SMARCC1	rkksgkkgqaSlygkrrsqke
IPI00234252	346	343	unsp+PKC+PKG+cdc2+PKA+RSK->unsp+PKC+PKG+PKA+R	unsp+PKC+PKG+cdc2+PKA+RSK->unsp+PKC+PKG+PKA		6599	SMARCC1	pptptesrkkSgkkgqaslyg
IPI00234252	354	350	unsp+DNAPK->unsp+PKA+DNAPK	unsp+DNAPK->unsp+PKA+DNAPK		6599	SMARCC1	rkksgkkgqaSlygkrrsqke
IPI00234252	354	357	ATM+unsp+CKII+PKA+DNAPK->ATM+unsp+PKG+CKII+PKA	ATM+unsp+CKII+PKA+DNAPK->ATM+unsp+PKG+CKI	Uniprot|PhosphoSite	6599	SMARCC1	gqaslygkrrSqkeedeqedl
IPI00234446	374	367		unsp+PKC+cdc2->unsp+PKC	PhosphoSite	1386	ATF2	kflernraaaSrcrqkrkvwv
IPI00240812	1136	1139	PKC->			23047	PDS5B	nvlgavnkplSsagkqsqtks
IPI00240812	1136	1140	PKC->			23047	PDS5B	vlgavnkplsSagkqsqtkss
IPI00245135	264	262	INSR+unsp+EGFR->unsp+EGFR			79621	RNASEH2B	sdepveakedYtkfntkdlkt
IPI00245135	295	292	unsp+PKC->unsp	unsp+PKC->unsp		79621	RNASEH2B	aqkalakvdkSgmksidtffg
IPI00245135	295	296		->PKA		79621	RNASEH2B	lakvdksgmkSidtffgvkkk
IPI00245135	295	299	unsp+CKI+PKC->CKI	unsp+CKI+PKC->CKI+PKC		79621	RNASEH2B	vdksgmksidTffgvkkkler
IPI00246058	48	47		cdc2->cdc2+DNAPK		10015	PDCD6IP	aqycraaeelSklrraavgrp
IPI00246188	47	43	unsp+PKC+CKII->unsp+CKII	unsp+PKC+CKII->unsp+CKII		342541	LOC342541	pktaenfpalStgekgfgyks
IPI00246188	47	44	PKC->	PKC->		342541	LOC342541	ktaenfpalsTgekgfgykss
IPI00246188	47	51	unsp->			342541	LOC342541	alstgekgfgYksscfhriip
IPI00250153	116	115		unsp->unsp+DNAPK		51087	YBX2	ngygfinrndTkedvfvhqta
IPI00255316	6	2	PKC->		Uniprot	3013	HIST1H2AD	---------mSgrgkqggkar
IPI00256605	302	297	unsp+PKC->	unsp+PKC->PKC		51322	WAC	tlsklptptsSvpaqkterke
IPI00257882	493	490	PKC->	PKC->		5184	PEPD	agcdkaftpfSgpk-------
IPI00289034	397	390	unsp+cdk5->unsp+cdk5+p38MAPK	unsp+cdk5->unsp+cdk5+p38MAPK	PhosphoSite	84444	DOT1L	kagaatvkkpSpskarkkkln
IPI00289159	311	308	->EGFR	->EGFR		2744	GLS	ndlgteyvhrYvgkepsglrf
IPI00289271	828	832	PKC->			8499	PPFIA2	hkapkkkgikSsigrlfgkke

IPI00289271	828	833	unsp+PKA->	unsp+PKA->PKA		8499	PPFIA2	kapkkkgiksSigrlfgkkek
IPI00289271	831	833	unsp+PKA->PKC	unsp+PKA->unsp		8499	PPFIA2	kapkkkgiksSigrlfgkkek
IPI00289334	2170	2168	unsp+PKC->PKC	unsp+PKC->		2317	FLNB	vpqemgvhtvSvkyrgqhvtg
IPI00289334	2524	2523	PKC->PKC+PKA	PKC->DNAPK		2317	FLNB	skvtskgaglSkafvgqkssf
IPI00289334	2524	2531	->PKA	->PKA		2317	FLNB	glskafvgqkSsflvdcskag
IPI00289334	2576	2572	PKC->	PKC->		2317	FLNB	khvgnqqynvTyvvkergdyv
IPI00289344	1412	1408	PKC->	PKC->		9611	NCOR1	aikhnvksliTgpsklsrgmp
IPI00289344	1412	1411		PKC->		9611	NCOR1	hnvkslitgpSklsrgmpple
IPI00289344	1412	1414	unsp+PKA->unsp+PKC	unsp+PKA->unsp		9611	NCOR1	kslitgpsklSrgmppleivp
IPI00289499	199	190	unsp+CKII->unsp+cdc2+CKII			471	ATIC	thtaqydeaiSdyfrkqyskg
IPI00289499	199	192	INSR->	INSR->		471	ATIC	taqydeaisdYfrkqyskgvs
IPI00289499	199	198	PKC->PKA	PKC->ATM+DNAPK		471	ATIC	aisdyfrkqySkgvsqmplry
IPI00289499	199	202	unsp->ATM	unsp->ATM		471	ATIC	yfrkqyskgvSqmplrygmnp
IPI00289499	356	352	PKC->	PKC->		471	ATIC	iapgyeeealTilskkkngny
IPI00289499	356	355		PKC->unsp+ATM+PKC+DNAPK		471	ATIC	gyeeealtilSkkkngnycvl
IPI00289499	357	352	PKC->	PKC->		471	ATIC	iapgyeeealTilskkkngny
IPI00289524	246	251	PKG+PKA+p38MAPK->p38MAPK			1109	AKR1C4	lcalakkhkrTpalialryql
IPI00289524	270	271	unsp->unsp+CKII	unsp->unsp+CKII		1109	AKR1C4	lqrgvvvlakSyneqrireni
IPI00289524	270	272		->EGFR		1109	AKR1C4	qrgvvvlaksYneqrireniq
IPI00289601	169	162	INSR+unsp->unsp	INSR+unsp->unsp		3066	HDAC2	hkataeemtkYhsdeyikflr
IPI00289601	169	167	unsp+EGFR->unsp			3066	HDAC2	eemtkyhsdeYikflrsirpd
IPI00289601	169	173	unsp+PKC->			3066	HDAC2	hsdeyikflrSirpdnmseys
IPI00289601	184	183		->DNAPK		3066	HDAC2	sirpdnmseySkqmqrfnvge
IPI00289746	256	262	unsp+PKC->unsp+CKI+PKC			5058	PAK1	eileklrsivSvgdpkkkytr
IPI00289773	265	257		unsp+DNAPK->ATM+unsp+DNAPK		1051	CEBPB	acyagaapapSqvkskakktv
IPI00289773	265	266		PKC->unsp+PKC	PHOsphoELM|PhosphoSite	1051	CEBPB	psqvkskakkTvdkhsdeyki
IPI00289807	402	399	unsp+PKA->PKA		PhosphoSite	51095	TRNT1	sghdirkvgiSsgkeigallq
IPI00289807	402	400	unsp+PKC+CKII->unsp+CKII	unsp+PKC+CKII->unsp+CKII	PhosphoSite	51095	TRNT1	ghdirkvgisSgkeigallqq
IPI00289819	2352	2353	INSR->			3482	IGF2R	crrssnvsykYskvnkeeetd
IPI00289819	2352	2354	->PKC			3482	IGF2R	rrssnvsykySkvnkeeetde
IPI00289866	262	261		PKC+cdc2->ATM+cdc2+DNAPK		2308	FOXO1	rrraasmdnnSkfaksrsraa
IPI00289866	265	261	PKC+cdc2->	PKC+cdc2->cdc2		2308	FOXO1	rrraasmdnnSkfaksrsraa
IPI00290204	118	115	unsp->	unsp->		6625	SNRNP70	lfvarvnydtTesklrrefev
IPI00290204	118	117	PKC->	PKC->DNAPK		6625	SNRNP70	varvnydtteSklrrefevyg
IPI00290204	162	159	unsp->			6625	SNRNP70	ieyeherdmhSaykhadgkki
IPI00290204	162	161		->EGFR		6625	SNRNP70	yeherdmhsaYkhadgkkidg
IPI00290314	633	631	unsp->		PhosphoSite	56897	WRNIP1	aptrlmkdlgYgkgykynpmy
IPI00290416	216	222	->cdc2	->cdc2		29789	OLA1	evlnkhlfltSkpmvyIvnls
IPI00290548	119	117	unsp+cdk5+p38MAPK->cdk5+p38MAPK	unsp+cdk5+p38MAPK->GSK3+cdk5+p38MAPK		1870	E2F2	gkcirvdglpSpktpkspgek
IPI00290548	119	120	unsp+cdk5+p38MAPK->unsp+PKC+cdk5+p38MAPK	unsp+cdk5+p38MAPK->unsp+PKC+cdk5+p38MAPK		1870	E2F2	irvdglpspkTpkspgektry
IPI00290548	119	123	unsp+GSK3+p38MAPK->unsp+GSK3			1870	E2F2	dglpspktpkSpgektrydts
IPI00290548	122	120		unsp+cdk5+p38MAPK->unsp+GSK3+cdk5+p38MAPK		1870	E2F2	irvdglpspkTpkspgektry
IPI00290548	127	123	unsp+GSK3+p38MAPK->unsp+GSK3+cdk5+p38MAPK			1870	E2F2	dglpspktpkSpgektrydts
IPI00290548	127	130	INSR+unsp->	INSR+unsp->unsp	PhosphoSite	1870	E2F2	tpkspgektrYdtslglltkk
IPI00290548	127	132	unsp+PKA->unsp	unsp+PKA->unsp		1870	E2F2	kspgektrydTslglltkkfi
IPI00290566	199	204	ATM+unsp+PKG+PKA->ATM+unsp			6950	TCP1	svnilkahgrSqmesmlisgy
IPI00290652	1050	1053	unsp->	unsp->		51773	RSF1	gggvgrgkdiStitghrgkdi
IPI00290652	1061	1064	unsp+CKII->CKII	unsp+CKII->CKII		51773	RSF1	titghrgkdiStildeerken
IPI00290652	1339	1336	unsp+PKC->unsp	unsp+PKC->unsp	PhosphoSite	51773	RSF1	qprvlpseqeStkkpyriesd
IPI00290652	1339	1337	unsp+PKC->	unsp+PKC->		51773	RSF1	prvlpseqesTkkpyriesde
IPI00290857	208	204	PKC->	PKC->		3850	KRT3	vkaqereqikTlnnkfasfid
IPI00290857	208	211	unsp+PKC->unsp			3850	KRT3	qiktlnnkfaSfidkvrfleq
IPI00291006	239	235		unsp->		4191	MDH2	altgriqeagTevvkakagag
IPI00291006	296	289		->PKA		4191	MDH2	ksqetectyfStplllgkkgi
IPI00291006	297	289	->PKA	->PKA		4191	MDH2	ksqetectyfStplllgkkgi
IPI00291006	314	317	CKII->cdc2+CKII	CKII->cdc2+CKII		4191	MDH2	kvssfeekmiSdaipelkasi
IPI00291006	338	336	unsp+PKC->	unsp+PKC->		4191	MDH2	sikkgedfvkTlk--------
IPI00291131	36	40	unsp+CKII->CKII			80145	THOC7	lvksfikwcnSgsqeegysqy
IPI00291136	121	115	unsp+RSK->unsp	unsp+RSK->unsp		1291	COL6A1	rmpggrdalkSsvdavkyfgk
IPI00291136	121	116	PKC+cdc2->PKA+cdc2	PKC+cdc2->cdc2		1291	COL6A1	mpggrdalksSvdavkyfgkg
IPI00291419	262	261		->DNAPK		39	ACAT2	prhgsnieamSklkpyfltdg
IPI00291467	23	22		->DNAPK		293	SLC25A6	flaggiaaaiSktavapierv
IPI00291467	96	95		INSR+unsp->INSR+unsp+EGFR		293	SLC25A6	qalnfafkdkYkqifggvdk
IPI00291483	246	251	PKG+PKA+p38MAPK->p38MAPK			8644	AKR1C3	lcalakkhkrTpalialryql
IPI00291510	436	432	unsp->unsp+RSK	unsp->unsp+PKG		3615	IMPDH2	khlssqnryfSeadkikvaqg
IPI00291510	511	505	CKI->	CKI->		3615	IMPDH2	ssaqveggvhSlhsyekrlf-
IPI00291608	64	63	PKC->	PKC->ATM		57092	PCNP	saeeeaadlpTkptkiskfgf
IPI00291608	64	66	PKC+PKG->PKC	PKC+PKG->PKC	Uniprot|PhosphoSite	57092	PCNP	eeaadlptkpTkiskfgfaig
IPI00291608	64	69	PKG+PKA->	PKG+PKA->PKG	Uniprot|PhosphoSite	57092	PCNP	adlptkptkiSkfgfaigsqt
IPI00291608	67	63	PKC->	PKC->		57092	PCNP	saeeeaadlpTkptkiskfgf
IPI00291608	67	69	PKG+PKA->	PKG+PKA->	Uniprot|PhosphoSite	57092	PCNP	adlptkptkiSkfgfaigsqt
IPI00291608	70	66	PKC+PKG->PKG		Uniprot|PhosphoSite	57092	PCNP	eeaadlptkpTkiskfgfaig
IPI00291608	70	69		PKG+PKA->ATM+PKG	Uniprot|PhosphoSite	57092	PCNP	adlptkptkiSkfgfaigsqt
IPI00291608	70	77	PKC+cdc2+DNAPK->ATM+PKC+cdc2+DNAPK		Uniprot|PhosphoSite	57092	PCNP	kiskfgfaigSqttkkasais
IPI00291608	152	156		->cdc2		57092	PCNP	nsfnkgkhgfSdnqklwerni
IPI00291643	95	93	unsp+PKC->PKC+cdc2	unsp+PKC->PKC+cdc2		283377	SPRYD4	vtsgrhywevTvkrsqqfrig
IPI00291643	95	97	ATM+PKA+DNAPK->ATM+DNAPK	ATM+PKA+DNAPK->ATM+DNAPK		283377	SPRYD4	rhywevtvkrSqqfrigvadv
IPI00291643	130	133	PKC->			283377	SPRYD4	vftyaqrkwyTmlanekapve
IPI00291646	189	183		CKII->		25902	MTHFD1L	lkpekdvdgvTdinlgklvrg
IPI00291669	71	70	->PKA	->DNAPK		134510	UBLCP1	klgalklkpnTkimmmgtree
IPI00291669	117	119	->PKC			134510	UBLCP1	enreenllkiSrrvkeykvei
IPI00291669	245	243	unsp->		PhosphoSite	134510	UBLCP1	gviwgkfsefYskkntimfdd
IPI00291669	245	244		unsp->ATM+unsp	PhosphoSite	134510	UBLCP1	viwgkfsefySkkntimfddi
IPI00291669	246	243		unsp->	PhosphoSite	134510	UBLCP1	gviwgkfsefYskkntimfdd
IPI00291669	246	244	unsp->	unsp->	PhosphoSite	134510	UBLCP1	viwgkfsefySkkntimfddi
IPI00291764	6	2	PKC->		Uniprot|PHOsphoELM|PhosphoSi	8336	HIST1H2AN	---------mSgrgkqggkar
IPI00291783	1363	1361	unsp+PKC->CKII	unsp+PKC->		25929	GEMIN5	rmlstfkelfSekhaslqnsq
IPI00291800	486	489	unsp+PKC->PKC+cdc2	unsp+PKC->PKC		56478	EIF4ENIF1	gdmtafnklvStmkasgtlps
IPI00291916	1533	1529	PKC->	PKC->		55023	PHIP	eqpstssaakTfitkanasai
IPI00291916	1533	1532	PKC+cdc2->cdc2	PKC+cdc2->cdc2		55023	PHIP	stssaaktfiTkanasaipgk
IPI00291930	643	642		->ATM+DNAPK		9685	CLINT1	qdafanfanfSk---------
IPI00291939	437	441	unsp+EGFR->			8243	SMC1A	nqkriekleeYittskqslee
IPI00291939	713	715		ATM+unsp+DNAPK->ATM+unsp+CaM-II+DNAPK		8243	SMC1A	ahglqmrlkySqsdleqtktr
IPI00292012	614	616	unsp+PKA->PKC	unsp+PKA->		23461	ABCA5	tikdnqakklSggqkrklslg
IPI00292012	620	624	unsp+PKG+PKA+RSK->PKG+PKA+RSK			23461	ABCA5	klsggqkrklSlgiavlgnpk
IPI00292059	384	386	PKG->PKC	PKG->	PhosphoSite	9972	NUP153	atnrsvyfkpSltpsgefrkt
IPI00292059	384	388	unsp+p38MAPK->unsp		PhosphoSite	9972	NUP153	nrsvyfkpslTpsgefrktnq
IPI00292059	718	713	CKI+PKC->CKI	CKI+PKC->PKC		9972	NUP153	sgkttlsasgTgfgdkfkpvi
IPI00292059	954	953	CKI->CKI+PKA	CKI->ATM+DNAPK		9972	NUP153	inpmsegfkfSkpigdfkfgv
IPI00292059	1120	1115	unsp+CKI->CKI	unsp+CKI->		9972	NUP153	ekqqepvtstSlvfgkkadne
IPI00292135	55	59	unsp+PKC->PKC			3930	LBR	lkendikpltSfrqrkggsts
IPI00292238	128	125	unsp+PKC->unsp			1417	CRYBB3	klhlfenpafSgrkmeivddd
IPI00292537	441	438	unsp->	unsp->		4790	NFKB1	nagmkhgtmdTeskkdpegcd
IPI00292537	441	440		unsp+PKC->ATM+unsp+PKC+DNAPK		4790	NFKB1	gmkhgtmdteSkkdpegcdks
IPI00292537	442	435	CKII->cdc2+CKII			4790	NFKB1	tksnagmkhgTmdteskkdpe
IPI00292537	442	438		unsp->		4790	NFKB1	nagmkhgtmdTeskkdpegcd
IPI00292537	442	440	unsp+PKC->unsp	unsp+PKC->unsp		4790	NFKB1	gmkhgtmdteSkkdpegcdks
IPI00292746	249	251	unsp+PKG->	unsp+PKG->unsp		113878	DTX2	hqafapynkpSlsgarsaprl
IPI00292746	249	253	unsp->			113878	DTX2	afapynkpslSgarsaprlnt
IPI00292753	238	240		PKC->		26130	GAPVD1	qqeklfgekgSdrfrqkvqem
IPI00292771	2070	2069		unsp+PKC+PKA+RSK->unsp+PKC+RSK		4926	NUMA1	lgnsllrrgaSkkalskaspn
IPI00292771	2070	2074	unsp+PKC->		Uniprot	4926	NUMA1	lrrgaskkalSkaspntrsgt
IPI00292771	2070	2077	->p38MAPK		Uniprot	4926	NUMA1	gaskkalskaSpntrsgtrrs
IPI00292771	2071	2069	unsp+PKC+PKA+RSK->PKA+RSK	unsp+PKC+PKA+RSK->PKA+RSK		4926	NUMA1	lgnsllrrgaSkkalskaspn
IPI00292771	2071	2074	unsp+PKC->PKC	unsp+PKC->PKC	Uniprot	4926	NUMA1	lrrgaskkalSkaspntrsgt
IPI00293009	282	279	PKC->	PKC->		9135	RABEP1	erqqhnqlkhTwqkandqfle
IPI00293026	528	530		p38MAPK->		79631	EFTUD1	kkifvlgpkySpleflrrvpl
IPI00293276	78	75	cdc2+PKA->PKA	cdc2+PKA->PKA		4282	MIF	igkiggaqnrSyskllcglla
IPI00293331	40	36	unsp+CKII->unsp+PKA+CKII	unsp+CKII->unsp+PKA+CKII		10940	POP1	fvadrgvkhhSggekpfqaqk
IPI00293331	46	54	unsp->unsp+cdc2			10940	POP1	aqkqephpgtSrqrqtrvnph
IPI00293350	68	67	unsp+PKC->PKC	unsp+PKC->ATM+unsp+DNAPK		7257	TSNAX	vklsrditveSkrtifllhri
IPI00293426	455	453	unsp+PKC->	unsp+PKC->		6597	SMARCA4	krqslreariTeklekqqkie
IPI00293464	383	379	cdc2->PKA			1E+08	LOC100290	rqgqgqlvtcSgafkegslri
IPI00293464	1067	1064	unsp+PKC->PKC+PKA			1E+08	LOC100290	mqnrlnkvikSvgkiehsfwr
IPI00293464	1067	1071	unsp->			1E+08	LOC100290	viksvgkiehSfwrsfhterk
IPI00293523	209	212	PKC->			1316	KLF6	chfngcrkvyTksshlkahqr
IPI00293523	213	212	PKC->	PKC->DNAPK		1316	KLF6	chfngcrkvyTksshlkahqr
IPI00293523	213	214	->PKC	->PKC		1316	KLF6	fngcrkvytkSshlkahqrth

IPI00293523	218	214	->PKA			1316	KLF6	fngcrkvytkSshlkahqrth
IPI00293523	218	215	PKC+cdc2->cdc2			1316	KLF6	ngcrkvytksShlkahqrtht
IPI00293568	123	128	GSK3+p38MAPK->p38MAPK			84220	RGPD5	veraaklfpgSpaiyklkeql
IPI00293613	584	592	->INSR			29110	TBK1	ihkfdkqklyYhatkamthft
IPI00293655	268	269	->ATM+DNAPK	->DNAPK		1653	DDX1	skapdgyivkSqhsgnaqvtq
IPI00293655	281	280		PKC->ATM+PKC		1653	DDX1	qhsgnaqvtqTkflpnapkal
IPI00293657	29	20		cdc2->		3660	IRF2	pwleeqinsnTipglkwlnke
IPI00293657	75	76		PKC->unsp+PKC		3660	IRF2	qpgvdkpdpkTwkanfrcamn
IPI00293746	53	49	unsp+PKC->unsp			79169	C1orf35	vgrwqkgrdlTwyakgrapca
IPI00293845	1274	1271	unsp+PKC+PKG+RSK->	unsp+PKC+PKG+RSK->unsp+PKG		55183	RIF1	flpkakqregTfsksdsekiv
IPI00293845	1274	1273	unsp+CKII->unsp+CKI	unsp+CKII->ATM+unsp+CKII+DNAPK		55183	RIF1	pkakqregtfSksdsekivng
IPI00293845	1274	1277	unsp->	unsp->		55183	RIF1	qregtfsksdSekivngtkrs
IPI00293921	581	584	PKC+PKA->	PKC+PKA->PKC		11073	TOPBP1	iikenagkimSllsrtvadya
IPI00293921	581	589	->cdc2			11073	TOPBP1	agkimsllsrTvadyavvpll
IPI00294084	385	376	->cdc2			2034	EPAS1	hlmamnsifdSsgkgavseks
IPI00294084	385	383	unsp+PKC->unsp+CKII	unsp+PKC->unsp		2034	EPAS1	ifdssgkgavSeksnflftkl
IPI00294084	385	391	PKC->PKC+cdc2			2034	EPAS1	avseksnflfTklkeepeela
IPI00294084	685	681	PKC->			2034	EPAS1	vspphvstfkTrsakgfgarg
IPI00294084	685	683	unsp+PKC->	unsp+PKC->		2034	EPAS1	pphvstfktrSakgfgargpd
IPI00294158	269	267	unsp+CKII->CKII	unsp+CKII->CKII		644	BLVRA	ifvqkllgqfSekelaaekkr
IPI00294159	97	88	PKA->cdc2+PKA			6576	SLC25A1	hgvlglyrglSsllygsipka
IPI00294159	97	92	SRC+unsp->unsp			6576	SLC25A1	glyrglsslIYgsipkaavrf
IPI00294159	160	155	PKC->	PKC->		6576	SLC25A1	tikvkfihdqTspnpkyrgff
IPI00294159	255	256	unsp->	unsp->		6576	SLC25A1	trmqgleahkYrntwdcglqi
IPI00294159	255	259	unsp+PKA->cdc2+PKA	unsp+PKA->cdc2		6576	SLC25A1	qgleahkyrnTwdcglqilkk
IPI00294186	264	263	PKC->	PKC->ATM		114294	LACTB	egksnekndfTkfkteqenea
IPI00294186	342	339	unsp->unsp+PKA	unsp->unsp+PKA		114294	LACTB	tllaaiveraSgckyldymqk
IPI00294186	342	343	unsp->	unsp->		114294	LACTB	aiverasgckYldymqkifhd
IPI00294186	380	386	unsp+p38MAPK->unsp			114294	LACTB	vynkkkrlvnTpyvdnsykwa
IPI00294186	459	460	unsp->SRC+unsp	unsp->SRC+unsp		114294	LACTB	temswdkegkYamawgvverk
IPI00294211	260	256	unsp+PKC+PKB+RSK->unsp+PKB+RSK	unsp+PKC+PKB+RSK->unsp+PKB+RSK		9785	DHX38	hrlstrdrdrSvrgkysddtp
IPI00294211	260	262	PKA->	PKA->		9785	DHX38	drdrsvrgkySddtplptpsy
IPI00294211	521	513	unsp->unsp+PKA			9785	DHX38	qkfadhmkrkSeassefakkk
IPI00294211	521	517	unsp+PKC->unsp			9785	DHX38	dhmkrkseasSefakkksile
IPI00294211	521	524	unsp+PKG+PKA->unsp+PKG+cdc2+PKA	unsp+PKG+PKA->unsp+PKG+cdc2+PKA		9785	DHX38	eassefakkkSileqrqylpi
IPI00294211	522	524	unsp+PKG+PKA->unsp	unsp+PKG+PKA->unsp		9785	DHX38	eassefakkkSileqrqylpi
IPI00294495	122	118	PKC->			51506	UFC1	kmyrggkiclTdhfkplwarn
IPI00294603	1282	1280	unsp+PKC->PKC	unsp+PKC->PKC		7750	ZMYM2	cgtdnedkitTgkrkheddep
IPI00294742	581	576	PKC->			51574	LARP7	tkaekirlakTqqaskhirfs
IPI00294742	581	580		->ATM+DNAPK		51574	LARP7	kirlaktqqaSkhirfseyd-
IPI00294742	581	586	unsp+PKA->unsp+CKI			51574	LARP7	tqqaskhirfSeyd-------
IPI00294744	220	216	PKC->	PKC->		5073	PARN	tgfqrkliyqTlswkypkgih
IPI00294744	220	218	unsp+PKC+cdc2->PKC+cdc2	unsp+PKC+cdc2->PKC+cdc2		5073	PARN	fqrkliyqtlSwkypkgihve
IPI00294744	499	497	unsp+PKC->	unsp+PKC->		5073	PARN	qpeqvkiavnTskyaesyriq
IPI00294744	499	498	unsp->unsp+DNAPK	unsp->ATM+unsp+DNAPK		5073	PARN	peqvkiavntSkyaesyriqt
IPI00294744	499	503	unsp+PKC->PKC			5073	PARN	iavntskyaeSyriqtyaeym
IPI00294744	566	562	cdc2->unsp+cdc2	cdc2->unsp+cdc2		5073	PARN	yyrnnsftapStvgkrnlsps
IPI00294744	566	570	unsp+PKA+RSK->unsp+cdc2+PKA	unsp+PKA+RSK->unsp+cdc2+p38MAPK	PhosphoSite	5073	PARN	apstvgkrnlSpsqeeagled
IPI00294779	20	22		->EGFR		7419	VDAC3	kaakdvfnkgYgfgmvkidlk
IPI00294779	62	56	CKII->PKA+CKII	CKII->PKA+CKII		7419	VDAC3	ghaytdtgkaSgnletkykvc
IPI00294779	62	61	PKC->	PKC->DNAPK		7419	VDAC3	dtgkasgnleTkykvcnyglt
IPI00294779	62	63	INSR+unsp->SRC+unsp	INSR+unsp->SRC+INSR+unsp		7419	VDAC3	gkasgnletkYkvcnygltft
IPI00294779	64	56	CKII->PKA+CKII	CKII->PKA+CKII		7419	VDAC3	ghaytdtgkaSgnletkykvc
IPI00294779	64	61	PKC->	PKC->		7419	VDAC3	dtgkasgnleTkykvcnyglt
IPI00294779	64	63	INSR+unsp->unsp	INSR+unsp->SRC+INSR+unsp		7419	VDAC3	gkasgnletkYkvcnygltft
IPI00294779	91	87	CKI+cdc2->unsp+CKI	CKI+cdc2->unsp+CKI		7419	VDAC3	ntdntlgteiSwenklaeglk
IPI00294840	747	750	PKC->			202	AIM1	lekvfdpkvfTfglgkkkesq
IPI00294842	136	133	PKC->			11183	MAP4K5	etlqglaylhTkgkmhrdikg
IPI00294879	26	24	unsp+PKC->PKC	unsp+PKC->		5905	RANGAP1	aktqvaggqlSfkgkslklnt
IPI00294879	26	29	unsp+PKC+PKA->PKC+PKA	unsp+PKC+PKA->PKC+PKA		5905	RANGAP1	aggqlsfkgkSlklntaedak
IPI00294879	528	525	PKC+cdc2->	PKC+cdc2->PKC		5905	RANGAP1	rllvhmgllkSedkvkaianl
IPI00294982	299	301	unsp+PKC+PKG+cdc2+PKA->unsp+PKC+cdc2	unsp+PKC+PKG+cdc2+PKA->unsp+PKC+cdc2		2099	ESR1	lwpsplmikrSkknslalslt
IPI00294982	302	301	unsp+PKC+PKG+cdc2+PKA->unsp+PKC+cdc2+PKA	unsp+PKC+PKG+cdc2+PKA->unsp+PKC+cdc2		2099	ESR1	lwpsplmikrSkknslalslt
IPI00294982	303	294		cdc2->	PhosphoSite	2099	ESR1	gdmraanlwpSplmikrskkn
IPI00294982	303	301	unsp+PKC+PKG+cdc2+PKA->PKG+cdc2+PKA	unsp+PKC+PKG+cdc2+PKA->cdc2+PKA		2099	ESR1	lwpsplmikrSkknslalslt
IPI00294982	303	305	PKA->PKC+PKA	PKA->PKA+DNAPK	PHOsphoELM|PhosphoSite	2099	ESR1	plmikrskknSlalsltadqm
IPI00295004	533	530	PKC->	PKC->		57679	ALS2	llpslrtevwTwgkgkegqlg
IPI00295363	46	44	unsp+PKC->PKC	unsp+PKC->PKC		5009	OTC	kvqlkgrdllTlknftgeeik
IPI00295363	88	90	unsp->			5009	OTC	kslgmifekrStrtrlstetg
IPI00295363	88	91	unsp+PKA->	unsp+PKA->		5009	OTC	slgmifekrsTrtrlstetgf
IPI00295363	88	93	PKC->	PKC->		5009	OTC	gmifekrstrTrlstetgfal
IPI00295363	231	228	->PKA			5009	OTC	atpkgyepdaSvtklaeqyak
IPI00295363	238	236		->unsp		5009	OTC	dasvtklaeqYakengtklll
IPI00295400	366	362	PKC->			7453	WARS	asdpnssiflTdtakqiktkv
IPI00295400	366	364	PKC->	PKC->		7453	WARS	dpnssifltdTakqiktkvnk
IPI00296053	66	67	unsp+EGFR->unsp			2271	FH	fgelkvpndkYygaqtvrstm
IPI00296053	66	68	SRC+EGFR->SRC			2271	FH	gelkvpndkyYgaqtvrstmn
IPI00296053	80	75	unsp+PKC->	unsp+PKC->PKC		2271	FH	dkyygaqtvrStmnfkiggvt
IPI00296053	80	76	PKC->	PKC->		2271	FH	kyygaqtvrsTmnfkiggvte
IPI00296053	256	260	PKC->			2271	FH	gyvqqvkyamTrikaampriy
IPI00296069	867	863	unsp+PKC->unsp			5926	ARID4A	kklkrkilgqSspekkirien
IPI00296183	3	2	->PKA	->ATM+DNAPK		218	ALDH3A1	---------mSkiseavkrar
IPI00296183	178	174		unsp+CKII->CKII		218	ALDH3A1	pvinggvpetTellkerfdhi
IPI00296183	194	187	->cdc2			218	ALDH3A1	lkerfdhilyTgstgvgkiim
IPI00296183	194	189	PKC->	PKC->		218	ALDH3A1	erfdhilytgStgvgkiimta
IPI00296183	194	190		unsp->		218	ALDH3A1	rfdhilytgsTgvgkiimtaa
IPI00296183	269	267	unsp+PKC+PKG+PKA->unsp+PKG+PKA	unsp+PKC+PKG+PKA->unsp+PKG+PKA		218	ALDH3A1	qnqiveklkkSlkefygedak
IPI00296337	117	112	PKC->	PKC->		5591	PRKDC	pysveikntcTsvytkdraak
IPI00296337	117	113	unsp+PKC->unsp+PKA	unsp+PKC->unsp		5591	PRKDC	ysveikntctSvytkdraakc
IPI00296337	117	116	->CKI			5591	PRKDC	eikntctsvyTkdraakckip
IPI00296337	832	837		PKC->PKC+PKG		5591	PRKDC	nkvvlkhlkkTknlssneais
IPI00296337	1057	1056	unsp+PKC->unsp	unsp+PKC->unsp+DNAPK		5591	PRKDC	pqqqekspvnTkslfkrlysl
IPI00296337	1057	1058	->PKC	->PKC		5591	PRKDC	qqekspvntkSlfkrlyslal
IPI00296337	1074	1079	PKA->			5591	PRKDC	hpnafkrlgaSlafnniyref
IPI00296337	1970	1968	unsp+PKC->cdc2+CKII	unsp+PKC->		5591	PRKDC	elkfyqgflfSekpeknllif
IPI00296337	2259	2261	unsp->unsp+PKC			5591	PRKDC	ipyrlifekfSgkdpnskdns
IPI00296337	3260	3266	unsp->unsp+cdc2+PKA	unsp->unsp+cdc2		5591	PRKDC	mkllkelhkeSktrddwlvsw
IPI00296337	3260	3268	->cdc2			5591	PRKDC	llkelhkeskTrddwlvswvq
IPI00296337	3608	3610	SRC+INSR+unsp->SRC+unsp	SRC+INSR+unsp->SRC+unsp		5591	PRKDC	pvnkkniekmYermyaalgdp
IPI00296337	3638	3635	unsp+PKC->PKC	unsp+PKC->PKC		5591	PRKDC	lgafrrkfiqTfgkefdkhfg
IPI00296441	54	57	unsp+PKC->	unsp+PKC->PKC		100	ADA	lnvigmdkplTlpdflakfdy
IPI00296441	232	233	->unsp+PKC	->unsp+PKC		100	ADA	vvkeavdilkTerlghgyhtl
IPI00296441	312	308		unsp->unsp+EGFR	PhosphoSite	100	ADA	lifkstldtdYqmtkrdmgft
IPI00296441	312	311		unsp+PKC->unsp+DNAPK		100	ADA	kstldtdyqmTkrdmgfteee
IPI00296635	68	70	->PKC			2632	GBE1	geneggidkfSrgyesfgvhr
IPI00296907	267	264	unsp+PKC->	unsp+PKC->		51	ACOX1	mkyaqvkpdgTyvkplsnklt
IPI00296907	267	270	unsp+PKC->unsp			51	ACOX1	kpdgtyvkplSnkltygtmvf
IPI00296907	272	270	unsp+PKC->	unsp+PKC->		51	ACOX1	kpdgtyvkplSnkltygtmvf
IPI00296907	272	275	unsp->	unsp->	PhosphoSite	51	ACOX1	yvkplsnkltYgtmvfvrsfl
IPI00296907	437	438	->PKC	->PKC		51	ACOX1	mlqtarflmkSydqvhsgklv
IPI00296907	437	439	INSR->			51	ACOX1	lqtarflmksYdqvhsgklvc
IPI00296907	643	639	p38MAPK->PKA+p38MAPK			51	ACOX1	yenlfewaknSplnkaevhes
IPI00296907	651	649	unsp+PKC->	unsp+PKC->	Uniprot	51	ACOX1	splnkaevheSykhlkslqsk
IPI00296907	651	655	PKC->	PKC->		51	ACOX1	evhesykhlkSlqskl-----
IPI00296913	42	40	PKC->	PKC->		11164	NUDT5	klekttymdpTgktrtwesvk
IPI00296913	42	43		->unsp		11164	NUDT5	kttymdptgkTrtwesvkrtt
IPI00296913	42	45	PKC->			11164	NUDT5	tymdptgktrTwesvkrttrk
IPI00296934	112	105		cdc2->		79576	NKAP	asssvyygsySrpygsdkpwp
IPI00296934	112	110	unsp+PKC->	unsp+PKC->		79576	NKAP	yygsysrpygSdkpwpslldk
IPI00296934	112	116	unsp->			79576	NKAP	rpygsdkpwpSlldkereesl
IPI00297254	1448	1444	PKC->	PKC->unsp+PKC		23094	SIPA1L3	pfqlsasvpkSffskqpvrnk
IPI00297254	1448	1447		->ATM+DNAPK		23094	SIPA1L3	lsasvpksffSkqpvrnkhpt
IPI00297333	319	322	unsp+PKC->unsp			57465	TBC1D24	kqkgitvkqkSvslskrqfvh
IPI00297333	319	324	unsp+PKC+cdc2->PKC+cdc2	unsp+PKC+cdc2->PKC+cdc2		57465	TBC1D24	kgitvkqksvSlskrqfvhla
IPI00297455	257	262	unsp+PKC+PKG+PKA->unsp+PKC+PKA			26993	AKAP8L	fgngmkqmrrTwktwttadfr
IPI00297477	179	178		unsp+PKC+PKA->ATM+unsp+PKC+DNAPK	Uniprot|PhosphoSite	6627	SNRPA1	aqlakdiarrSktfnpgaglp
IPI00297477	179	180	PKG+RSK->PKG	PKG+RSK->unsp		6627	SNRPA1	lakdiarrskTfnpgaglptd
IPI00297550	222	215	INSR+EGFR->EGFR	INSR+EGFR->EGFR		2162	F13A1	yvlndigvifYgevndiktrs

IPI00297550	222	225	unsp+PKA->	unsp+PKA->		2162	F13A1	ygevndiktrSwsygqfedgi
IPI00297550	222	227	unsp+PKA->unsp			2162	F13A1	evndiktrswSygqfedgild
IPI00297550	678	674	PKC->	PKC->		2162	F13A1	vwvhldgpgvTrpmkkmfrei
IPI00297572	576	575	unsp+PKC->unsp	unsp+PKC->unsp		9716	AQR	itvrptkpygTkfdrrrpfie
IPI00297579	10	6	PKC->	PKC->		11335	CBX3	-----masnkTtlqkmgkkqn
IPI00297579	10	7	PKC->	PKC->		11335	CBX3	----masnktTlqkmgkkqng
IPI00297579	21	19	unsp+PKC->unsp	unsp+PKC->unsp		11335	CBX3	qkmgkkqngkSkkveeaepee
IPI00297579	50	55	PKC->	PKC->		11335	CBX3	veyflkwkgfTdadntwepee
IPI00297779	154	150		CKII->unsp+CKII		10576	CCT2	allssavdhgSdevkfrqdlm
IPI00297779	181	180	PKC+cdc2->cdc2	PKC+cdc2->		10576	CCT2	kllthhkdhfTklaveavlrl
IPI00297851	1338	1329		unsp+PKC+cdc2->unsp+PKC		1105	CHD1	kkealsgagsSkrrkarakkn
IPI00297851	1346	1344	unsp+PKC->PKC	unsp+PKC->		1105	CHD1	arakknkamkSikvkeeiksd
IPI00297921	238	230	unsp+RSK->unsp			81856	ZNF611	qkqevhmrekSfqcnksgkaf
IPI00297921	238	236	unsp+PKC->cdc2	unsp+PKC->		81856	ZNF611	mreksfqcnkSgkafncssll
IPI00297931	513	505	PKC->PKC+cdc2			11276	SYNRG	apsllmplpgTkalpsmdkya
IPI00297931	513	510	unsp+PKC->	unsp+PKC->unsp		11276	SYNRG	mplpgtkalpSmdkyavfkgi
IPI00297931	744	739	PKC->	PKC->		11276	SYNRG	gstvkggqnsTaastkydvfr
IPI00297931	744	742	unsp+PKC->	unsp+PKC->		11276	SYNRG	vkggqnstaaStkydvfrqls
IPI00297931	744	743		unsp->unsp+DNAPK		11276	SYNRG	kggqnstaasTkydvfrqlsl
IPI00297931	744	745	unsp->	unsp->		11276	SYNRG	gqnstaastkYdvfrqlsleg
IPI00297931	744	752	unsp+CKI+PKA->unsp+CKI	unsp+CKI+PKA->unsp+CKI	ıniprot|PHOsphoELM|PhosphoSi	11276	SYNRG	stkydvfrqlSlegsglgved
IPI00298202	318	315	unsp->PKA	unsp->unsp+PKA		10005	ACOT8	egvirvkpqvSeskl------
IPI00298202	318	317		->ATM+DNAPK		10005	ACOT8	virvkpqvseSkl--------
IPI00298301	1880	1878	unsp+PKC->unsp	unsp+PKC->unsp		4621	MYH3	lvdklqvkvkSykrqaeeade
IPI00298301	1880	1879		->EGFR		4621	MYH3	vdklqvkvksYkrqaeeadeq
IPI00298308	886	883	PKC->	PKC->		160428	ALDH1L2	kleagtvfinTynktdvaapf
IPI00298308	903	899	->PKA	->PKA		160428	ALDH1L2	vaapfggvkqSgfgkdlgeea
IPI00298406	244	245		p38MAPK->unsp+p38MAPK		3033	HADH	pvmklvevikTpmtsqktfes
IPI00298406	244	248	unsp->	unsp->		3033	HADH	klveviktpmTsqktfeslvd
IPI00298406	261	260	->PKA+DNAPK	->ATM+DNAPK		3033	HADH	qktfeslvdfSkalgkhpvsc
IPI00298406	317	316	unsp->unsp+DNAPK	unsp->ATM+unsp+DNAPK		3033	HADH	glgfslkgdaSkedidtamkl
IPI00298423	194	196	unsp+PKA->	unsp+PKA->unsp		8050	PDHX	paarnilekhSldasqgtatg
IPI00298547	148	142		->cdc2		11315	PARK7	kmmngghytySenrvekdgli
IPI00298558	179	171	unsp->unsp+PKA			11235	PDCD10	ehqkkefvkySksfsdtlkty
IPI00298558	179	175	->PKA	->unsp		11235	PDCD10	kefvkysksfSdtlktyfkdg
IPI00298558	179	177	unsp+PKC->	unsp+PKC->		11235	PDCD10	fvkysksfsdTlktyfkdgka
IPI00298558	179	180		PKC->unsp+PKC		11235	PDCD10	ysksfsdtlkTyfkdgkainv
IPI00298612	192	195		unsp->unsp+PKC		10295	BCKDK	lvryfldktlTsrlgirmlat
IPI00298731	207	204	unsp+PKC->			5514	PPP1R10	kpkslrttapShakfrstgle
IPI00298731	207	211	unsp+CKII+PKA->unsp+CKII	unsp+CKII+PKA->unsp+CKII		5514	PPP1R10	tapshakfrsTgleletpslv
IPI00298731	239	236		unsp->		5514	PPP1R10	nastvvvsdkYnlkpiplkrq
IPI00298731	274	273		->DNAPK		5514	PPP1R10	ykplnttpnaTkeikvkiipp
IPI00298860	321	323	PKG+cdc2->cdc2	PKG+cdc2->cdc2		4057	LTF	sgqkdllfkdSaigfsrvppr
IPI00298935	361	358	unsp+PKC->PKA	unsp+PKC->PKC+PKA		51780	KDM3B	pqinrnirfaTytkengrtlv
IPI00298935	361	360		->DNAPK		51780	KDM3B	inrnirfatyTkengrtlvvq
IPI00298961	568	564	PKC->			7514	XPO1	flrahwkflkTvvnklfefmh
IPI00298961	686	684	unsp+PKC->	unsp+PKC->		7514	XPO1	knvdilkdpeTvkqlgsilkt
IPI00298961	686	690	PKC->			7514	XPO1	kdpetvkqlgSilktnvrack
IPI00298961	693	690	PKC->PKA			7514	XPO1	kdpetvkqlgSilktnvrack
IPI00298994	2043	2040	unsp+DNAPK->DNAPK		Uniprot	7094	TLN1	tkvlvqnaagSqeklaqaaqs
IPI00298994	2043	2050	unsp+PKC->unsp+PKC+cdc2	unsp+PKC->unsp+PKC+cdc2		7094	TLN1	sqeklaqaaqSsvatitrlad
IPI00298994	2115	2117	PKC+PKG->PKC	PKC+PKG->		7094	TLN1	ddpavwqlknSakvmvtnvts
IPI00299095	469	468		CKI->ATM+DNAPK		6643	SNX2	qqgerdfeqiSktirkevgrf
IPI00299145	173	169	PKC->	PKC->		286887	KRT6C	vraeereqikTlnnkfasfid
IPI00299145	173	176	unsp+PKC->unsp			286887	KRT6C	qiktlnnkfaSfidkvrfleq
IPI00299147	11	12		CKII->unsp+CKII		6612	SUMO3	seekpkegvkTendhinlkva
IPI00299147	41	37	unsp+p38MAPK->unsp+PKA+p38MAPK	unsp+p38MAPK->p38MAPK		6612	SUMO3	svvqfkikrhTplsklmkayc
IPI00299147	41	40		PKC->ATM+PKC+DNAPK		6612	SUMO3	qfkikrhtplSklmkaycerq
IPI00299147	44	40	PKC->unsp	PKC->unsp+PKC		6612	SUMO3	qfkikrhtplSklmkaycerq
IPI00299149	11	12		CKII->unsp+CKII		6613	SUMO2	adekpkegvkTenndhinlkv
IPI00299149	42	38	unsp+p38MAPK->unsp+PKA+p38MAPK	unsp+p38MAPK->p38MAPK		6613	SUMO2	svvqfkikrhTplsklmkayc
IPI00299149	42	41		PKC->ATM+PKC+DNAPK		6613	SUMO2	qfkikrhtplSklmkaycerq
IPI00299149	45	41	PKC->unsp	PKC->unsp+PKC		6613	SUMO2	qfkikrhtplSklmkaycerq
IPI00299155	127	121	INSR+unsp+EGFR->unsp+EGFR	INSR+unsp+EGFR->unsp+EGFR		5685	PSMA4	vtalcdikqaYtqfggkrpfg
IPI00299155	176	173	unsp->			5685	PSMA4	cignnsaaavSmlkqdykege
IPI00299155	176	179	INSR+unsp->	INSR+unsp->		5685	PSMA4	aaavsmlkqdYkegemtlksa
IPI00299155	180	179	INSR+unsp->	INSR+unsp->INSR		5685	PSMA4	aaavsmlkqdYkegemtlksa
IPI00299254	784	775	unsp+PKC->unsp+PKC+cdc2			9669	EIF5B	kkspdsdvaaTlkkqkkntkd
IPI00299254	784	783	unsp+PKG+CKII->unsp+CKII	unsp+PKG+CKII->unsp+CKII+DNAPK		9669	EIF5B	aatlkkqkknTkdefeeraka
IPI00299254	923	919		unsp->INSR+unsp+EGFR		9669	EIF5B	pmkelrvknqYekhkeveaaq
IPI00299263	228	231	unsp+PKC+PKA->PKA	unsp+PKC+PKA->PKC+PKA		26286	ARFGAP3	akkglgakkgSlgaqklantc
IPI00299263	229	231	unsp+PKC+PKA->PKC+PKA	unsp+PKC+PKA->unsp+PKA		26286	ARFGAP3	akkglgakkgSlgaqklantc
IPI00299313	158	155	PKC+CKII->CKII	PKC+CKII->CKII		51377	UCHL5	rqqmfefdtkTsakeedafhf
IPI00299402	1090	1086	->PKA			5091	PC	vffelngqlrSilvkdtqamk
IPI00299402	1090	1092		->DNAPK		5091	PC	gqlrsilvkdTqamkemhfhp
IPI00299417	24	28	unsp+PKG+CKII+PKA+RSK->unsp+PKG+CKII+PKA	unsp+PKG+CKII+PKA+RSK->unsp+PKG+CKII+PKA		8563	THOC5	gapaegkrnrSdteqegkyys
IPI00299463	208	207	PKC->DNAPK	PKC->ATM+DNAPK		4683	NBN	yppldepsigSknvdlsgrqe
IPI00299463	334	330	->PKA	->PKA		4683	NBN	knycdpqghpStglktttpgp
IPI00299463	334	331	PKC->	PKC->		4683	NBN	nycdpqghpsTglktttpgps
IPI00299463	334	336	->PKC			4683	NBN	qghpstglktTtpgpslsqgv
IPI00299463	334	337	unsp+cdk5+p38MAPK->unsp+GSK3+cdk5+p38MAPK		PhosphoSite	4683	NBN	ghpstglkttTpgpslsqgvs
IPI00299463	441	438	unsp+PKC->	unsp+PKC->PKC		4683	NBN	nyqlsptklpSinkskdrasq
IPI00299463	504	509	unsp+CKII->CKII	unsp+CKII->CKII	PhosphoSite	4683	NBN	slwknkeqhlSenepvdtnsd
IPI00299463	544	537	->cdc2			4683	NBN	dlksivknsaSkshaaeklrs
IPI00299463	544	539	unsp+PKC->PKC	unsp+PKC->PKC		4683	NBN	ksivknsaskShaaeklrsnk
IPI00299463	665	667	unsp+PKC->PKC			4683	NBN	tefrslviknStsrnpsgind
IPI00299463	665	668	unsp+PKC->PKC	unsp+PKC->PKC		4683	NBN	efrslviknsTsrnpsgindd
IPI00299463	665	669	unsp->			4683	NBN	frslvikn stSrnpsginddy
IPI00299463	715	717	unsp+PKG+PKA+CKII->unsp+CKII	unsp+PKG+PKA+CKII->unsp+CKII		4683	NBN	dliahharknTeleewlrqem
IPI00299465	263	268	PKC->	PKC->		54585	LZTFL1	ekelekkfqqTaayrnmkeil
IPI00299507	640	638	unsp+PKC->PKC	unsp+PKC->PKC		23397	NCAPH	vnkieihyakTakkmdmkklk
IPI00299571	154	152	unsp+PKC->	unsp+PKC->		10130	PDIA6	ggqygvqgfpTikifgsnknr
IPI00299571	154	158	unsp+PKC->PKC			10130	PDIA6	qgfptikifgSnknrpedyqg
IPI00299571	293	291	unsp+PKC->	unsp+PKC->		10130	PDIA6	asrygirgfpTikifqkgesp
IPI00299571	293	300	unsp+GSK3->unsp+GSK3+cdc2			10130	PDIA6	ptikifqkgeSpvdydggrtr
IPI00299573	97	91	->PKA			6130	RPL7A	nqftqaldrqTatqllklahk
IPI00299573	97	93	PKC+DNAPK->DNAPK	PKC+DNAPK->DNAPK		6130	RPL7A	ftqaldrqtaTqllklahkyr
IPI00299608	310	311	GSK3+cdk5+p38MAPK->GSK3+p38MAPK	GSK3+cdk5+p38MAPK->unsp+cdk5+p38MAPK	PHOsphoELM|PhosphoSite	5707	PSMD1	ektssafvgkTpeaspepkdq
IPI00299749	487	489	INSR->	INSR->		7564	ZNF16	hqiihtgekpYrcsvcgkafs
IPI00299749	487	492	PKC+cdc2+PKA->cdc2	PKC+cdc2+PKA->cdc2+PKA		7564	ZNF16	ihtgekpyrcSvcgkafshss
IPI00300026	197	190	->p38MAPK			6817	SULT1A1	ipsgmetlkdTpaprllkthl
IPI00300052	175	171	->PKA			3890	KRT84	vkkdekeqikTlnnkfasfid
IPI00300052	175	178	unsp+PKC->unsp			3890	KRT84	qiktlnnkfaSfidkvrfleq
IPI00300052	182	178	unsp+PKC->unsp	unsp+PKC->unsp		3890	KRT84	qiktlnnkfaSfidkvrfleq
IPI00300060	452	443	unsp->unsp+cdc2			55100	WDR70	pddklivtgtSiqrgcgsgkl
IPI00300060	452	450	PKC+PKA->PKA	PKC+PKA->PKA		55100	WDR70	tgtsiqrgcgSgklvfferrt
IPI00300060	520	524		->cdc2		55100	WDR70	ktqrkakqaeTltqdyiitph
IPI00300078	700	696	PKC+cdc2->	PKC+cdc2->unsp+PKC+cdc2		5822	PWP2	lpgvrkgdmsSrhfkpeirvt
IPI00300078	700	707	unsp+PKC+RSK->unsp+PKC+PKA+RSK			5822	PWP2	rhfkpeirvtSlrfsptgrcw
IPI00300127	426	424	unsp+PKC+PKA->cdc2+PKA+RSK	unsp+PKC+PKA->PKA+RSK		55226	NAT10	ngyegtgrslSlkliqqlrqq
IPI00300186	360	356	unsp->unsp+PKA			79675	FASTKD1	gamgdmesrnSclikrvtsvl
IPI00300186	360	364	unsp+PKC+RSK->			79675	FASTKD1	rnsclikrvtSvlhkhldgyk
IPI00300371	109	106	PKC->			23450	SF3B3	knmfekihqeTfgksgcrriv
IPI00300371	109	110	unsp->	unsp->		23450	SF3B3	ekihqetfgkSgcrrivpgqf
IPI00300386	453	458	unsp->	unsp->		2873	GPS1	nqihvkplptStdlgppggsv
IPI00300567	283	282		->ATM+DNAPK		1632	DCI	advqnfvsfiSkdsiqkslqm
IPI00300567	283	285	unsp->PKC			1632	DCI	qnfvsfiskdSiqkslqmyle
IPI00300631	475	478	unsp+PKC->cdc2	unsp+PKC->PKC+cdc2		6294	SAFB	hktelhgkmiSvekaknepvg
IPI00300631	607	601	unsp->unsp+PKA		Uniprot|PHOsphoELM|PhosphoSi	6294	SAFB	drksasrekrSvvsfdkvkep
IPI00300631	607	604	CKI+PKC->PKC		Uniprot|PHOsphoELM|PhosphoSi	6294	SAFB	sasrekrsvvSfdkvkeprks
IPI00300631	805	801		->EGFR		6294	SAFB	grdsrdgwggYgsdkrmsegr
IPI00300631	805	803	PKC->cdc2+CKII	PKC->		6294	SAFB	dsrdgwggygSdkrmsegrgl
IPI00300631	805	808	unsp+PKA->unsp+cdc2	unsp+PKA->unsp	ıniprot|PHOsphoELM|PhosphoSi	6294	SAFB	wggygsdkrmSegrglppppr
IPI00300725	173	169	PKC->	PKC->		3853	KRT6A	vraeereqikTlnnkfasfid
IPI00300725	173	176	unsp+PKC->unsp			3853	KRT6A	qiktlnnkfaSfidkvrfleq
IPI00301058	283	278	unsp+PKC+PKB->unsp+PKB+PKA	unsp+PKC+PKB->unsp+PKB	ıniprot|PHOsphoELM|PhosphoSi	7408	VASP	namlarrrkaTqvgektpkde
IPI00301058	283	289	unsp->unsp+CKII			7408	VASP	qvgektpkdeSanqeepearv
IPI00301154	259	262	unsp->	unsp->		5042	PABPC3	ngkelngkqiYvgraqkkver

IPI00301224	236	232	unsp->unsp+PKA			55217	TMLHE	yftsdfsrgdTaytklaldrh
IPI00301224	236	234	unsp+EGFR->unsp			55217	TMLHE	tsdfsrgdtaYtklaldrhtd
IPI00301263	747	746		PKA->ATM+DNAPK		790	CAD	cvvkiprwdlSkflrvstkig
IPI00301263	747	752	unsp+PKC+PKA->unsp	unsp+PKC+PKA->unsp+PKA		790	CAD	rwdlskflrvStkigscmksv
IPI00301263	1411	1406	unsp+PKC+cdc2+PKA+RSK->unsp+cdc2+PKA+RSK	unsp+PKC+cdc2+PKA+RSK->unsp+cdc2+PKA+RSK	PHOsphoELM|PhosphoSite	790	CAD	smrgaggrrlSsfvtkgyrtr
IPI00301263	1411	1407	PKC+RSK->PKA+RSK	PKC+RSK->unsp+PKA+RSK		790	CAD	mrgaggrrlsSfvtkgyrtrr
IPI00301323	460	465	PKC+PKG->PKG	PKC+PKG->PKG		8886	DDX18	ihgkqkqnkrTttffqfcnad
IPI00301323	460	466	->PKA			8886	DDX18	hgkqkqnkrtTtffqfcnads
IPI00301323	571	563	ATM+CKII+DNAPK->CKII+DNAPK			8886	DDX18	fswskisdiqSqlekliekny
IPI00301518	149	146	->PKA	->PKA		55233	MOBKL1B	igvpfpknfmSvaktilkrlf
IPI00301609	731	730	PKC+cdc2->PKA+cdc2	PKC+cdc2->ATM+cdc2+DNAPK		91754	NEK9	tilivekvlnSktirsnssgl
IPI00302238	383	391	->cdc2			26574	AATF	lgkgfgaferSiltqidhilm
IPI00302742	133	128	unsp+PKA+RSK->unsp+PKA			79656	BEND5	hikrpegrkpSevahksieav
IPI00302829	427	424		unsp+EGFR->EGFR		5925	RB1	silkrvkdigYifkekfakav
IPI00302829	640	638	unsp+PKC->unsp	unsp+PKC->unsp+DNAPK		5925	RB1	aetqatsafqTqkplkstsls
IPI00302829	640	645		->PKG		5925	RB1	afqtqkplksTslslfykkvy
IPI00302829	640	646	->PKA			5925	RB1	fqtqkplkstSlslfykkvyr
IPI00302829	652	644	->PKA			5925	RB1	safqtqkplkStslslfykkv
IPI00302829	652	646	->PKA	->PKA		5925	RB1	fqtqkplkstSlslfykkvyr
IPI00302829	652	648	PKC+cdc2->PKC+PKA	PKC+cdc2->PKC+PKA+cdc2		5925	RB1	tqkplkstslSlfykkvyrla
IPI00302829	652	655	INSR->	INSR->		5925	RB1	tslslfykkvYrlaylrlntl
IPI00302927	139	136	PKC->			55522	ILK-2	kgihptiiseSfqkalekgie
IPI00302927	288	293	PKG+PKA->			55522	ILK-2	ilnlvkqikkTgcnvlliqks
IPI00302927	302	310	->cdc2			55522	ILK-2	iqksilrdalSdlalhflnkm
IPI00302927	319	310	->cdc2			55522	ILK-2	iqksilrdalSdlalhflnkm
IPI00303133	6	7	unsp->	unsp->		8345	HIST1H2BH	----mpdpakSapapkkgskk
IPI00303133	12	7	unsp->	unsp->		8345	HIST1H2BH	----mpdpakSapapkkgskk
IPI00303133	12	15	unsp+PKC->PKC	unsp+PKC->PKC	Uniprot	8345	HIST1H2BH	aksapapkkgSkkavtkaqkk
IPI00303133	13	15	unsp+PKC->PKC		Uniprot	8345	HIST1H2BH	aksapapkkgSkkavtkaqkk
IPI00303133	16	15	unsp+PKC->unsp+PKC+PKA	unsp+PKC->ATM+unsp+PKC+DNAPK	Uniprot	8345	HIST1H2BH	aksapapkkgSkkavtkaqkk
IPI00303133	21	15	unsp+PKC->unsp+PKC+PKA	unsp+PKC->unsp+PKC+PKA	Uniprot	8345	HIST1H2BH	aksapapkkgSkkavtkaqkk
IPI00303133	21	20		PKC->PKC+DNAPK		8345	HIST1H2BH	apkkgskkavTkaqkkdgkkr
IPI00303133	109	113	unsp->			8345	HIST1H2BH	lpgelakhavSegtkavtkyt
IPI00303133	121	120		->ATM+DNAPK		8345	HIST1H2BH	havsegtkavTkytssk----
IPI00303133	121	123		PKC->		8345	HIST1H2BH	segtkavtkyTssk-------
IPI00303135	244	243		PKC+cdc2->ATM+PKC+cdc2+DNAPK		55247	NEIL3	yrcrkaglalSkhykvykrpn
IPI00303135	247	243	PKC+cdc2->			55247	NEIL3	yrcrkaglalSkhykvykrpn
IPI00303207	121	117	->PKA	->PKA		6059	ABCE1	glvgtngigkStalkilagkq
IPI00303207	121	118	PKC->	PKC->		6059	ABCE1	lvgtngigksTalkilagkqk
IPI00303207	191	186	unsp+PKC->	unsp+PKC->		6059	ABCE1	ipkaakgtvgSildrkdetkt
IPI00303207	191	194	PKC->			6059	ABCE1	vgsildrkdeTktqaivcqql
IPI00303207	349	348	unsp->	unsp->		6059	ABCE1	neeevkkmcmYkypgmkkkmg
IPI00303207	431	423	unsp+PKC->unsp+PKC+PKA			6059	ABCE1	pqkispkstgSvrqllhekir
IPI00303207	431	436		INSR+EGFR->INSR		6059	ABCE1	qllhekirdaYthpqfvtdvm
IPI00303402	112	103	ATM+unsp+DNAPK->ATM+unsp+cdc2+DNAPK			51808	PHAX	kpepfqfgqsSqkppvaggkk
IPI00303476	133	128	->PKA			506	ATP5B	glvrgqkvldSgapikipvgp
IPI00303476	259	263	unsp->			506	ATP5B	sgvinlkdatSkvalvygqmn
IPI00303476	522	528		CKII->		506	ATP5B	akadklaeehSs---------
IPI00303797	418	411	->cdc2			673	BRAF	tppaslpglsITnvkalqkspg
IPI00303832	413	411	unsp+PKC+PKG->	unsp+PKC+PKG->		23168	RTF1	rvaeitgvveTakvyqlggtr
IPI00304306	93	96	unsp+cdk5->GSK3+cdk5+cdc2	unsp+cdk5->cdk5+cdc2		131118	DNAJC19	mllnhpdkggSpyiaakinea
IPI00304409	68	73	PKG+PKA->	PKG+PKA->PKG		23589	CARHSP1	ykgvckcfcrSkghgfitpad
IPI00304417	154	155	PKA->CKI+PKA	PKA->CKI+PKA		3420	IDH3B	dlfanvvhvkSlpgymtrhnn
IPI00304417	201	198	PKC->			3420	IDH3B	rgvieclkivTraksqriakf
IPI00304417	201	202	unsp+PKC+RSK->unsp+PKC+DNAPK+RSK	unsp+PKC+RSK->unsp+PKC+DNAPK+RSK		3420	IDH3B	eclkivtrakSqriakfafdy
IPI00304417	207	202	unsp+PKC+RSK->unsp+RSK	unsp+PKC+RSK->unsp+RSK		3420	IDH3B	eclkivtrakSqriakfafdy
IPI00304417	382	377	PKC+cdc2->cdc2	PKC+cdc2->cdc2		3420	IDH3B	rtrdmggystTtdfiksvigh
IPI00304417	382	378	PKC->	PKC->		3420	IDH3B	trdmggysttTdfiksvighl
IPI00304417	382	383		unsp+PKC->PKC		3420	IDH3B	gystttdfikSvighlqtkgs
IPI00304431	258	255	PKC+PKG+PKA+RSK->PKG+PKA+RSK	PKC+PKG+PKA+RSK->PKG+PKA		79650	C16orf57	rvhteqvrckSgnkffsmplk
IPI00304435	51	46	->PKA	->PKA		8508	NIPSNAP1	skdnegswfrSlfvhkvdprk
IPI00304527	418	427	unsp->unsp+cdc2	unsp->unsp+cdc2	Uniprot|PhosphoSite	222584	FAM83B	kkpsdslsvaSssregyvshh
IPI00304596	5	3	PKC->	PKC->		4841	NONO	--------mqSnktfnlekqn
IPI00304596	11	6	PKC->	PKC->		4841	NONO	-----mqsnkTfnlekqnhtp
IPI00304596	11	15	PKC+p38MAPK->p38MAPK			4841	NONO	ktfnlekqnhTprkhhqhhhq
IPI00304596	68	69	->PKC	->unsp+PKC		4841	NONO	lknfrkpgekTftqrsrlfvg
IPI00304596	68	74	cdc2->PKA+cdc2			4841	NONO	kpgektftqrSrlfvgnlppd
IPI00304596	198	196	unsp+cdc2->cdc2	unsp+cdc2->cdc2		4841	NONO	rpsgkgivefSgkpaarkald
IPI00304612	191	193	->PKC			23521	RPL13A	knvekkidkyTevlkthgllv
IPI00304740	475	477	PKA->	PKA->		6453	ITSN1	divvlkakkkTlefelealnd
IPI00304814	47	40	unsp+PKG->unsp+PKG+cdc2			2879	GPX4	asrddwrcarSmhefsakdid
IPI00304925	88	85	PKC->	PKC->		3304	HSPA1B	grkfgdpvvqSdmkhwpfqvi
IPI00304925	108	106	unsp+PKC->unsp	unsp+PKC->unsp	Uniprot	3304	HSPA1B	ndgdkpkvqvSykgetkafyp
IPI00304925	112	111	PKC->	PKC->DNAPK		3304	HSPA1B	pkvqvsykgeTkafypeeiss
IPI00304925	246	254	DNAPK->cdc2+DNAPK			3304	HSPA1B	efkrkhkkdiSqnkravrrlr
IPI00304925	500	502	PKA->	PKA->	PhosphoSite	3304	HSPA1B	dkstgkankiTitndkgrlsk
IPI00305144	217	216		PKC->PKC+DNAPK		10078	TSSC4	sscgegrvifTkpvrgvearh
IPI00305166	179	177	unsp+PKC->	unsp+PKC->		6389	SDHA	kiyqrafggqSlkfgkggqah
IPI00305166	335	339	PKC->	PKC->PKC+cdc2		6389	SDHA	ryapvakdlaSrdvvsrsmtl
IPI00305166	498	492	CKII->PKA+CKII	CKII->PKA		6389	SDHA	ppikpnageeSvmnldklrfa
IPI00305166	538	540	cdc2+PKA->cdc2	cdc2+PKA->cdc2		6389	SDHA	svlqegcgkiSklygdlkhlk
IPI00305166	541	540		cdc2+PKA->ATM+cdc2+PKA		6389	SDHA	svlqegcgkiSklygdlkhlk
IPI00305289	146	148	cdc2->CKII+cdc2	cdc2->CKII+cdc2		3832	KIF11	rtlhqifeklTdngtefsvkv
IPI00305374	300	304	unsp+PKC->unsp	unsp+PKC->unsp		9984	THOC1	ehvyfakfltSeklmdlqlsd
IPI00305545	128	124	PKC->	PKC->unsp		51163	DBR1	kyrgvriggiSgifkshdyrk
IPI00306043	245	244	PKC->PKC+DNAPK	PKC->ATM+PKC+DNAPK		51441	YTHDF2	pkpaswadiaSkpakqqpklk
IPI00306048	427	426		->DNAPK		83858	ATAD3B	flrkrateeiSkdlratlnaf
IPI00306048	427	432	PKC->	PKC->		83858	ATAD3B	teeiskdlraTlnaflyhmgq
IPI00306332	27	24	PKC->			6152	RPL24	ypghgrryarTdgkvfqflna
IPI00306332	77	86	unsp->unsp+PKA		Uniprot|PhosphoSite	6152	RPL24	vkfqraitgaSladimakrnq
IPI00306332	93	86	unsp->unsp+PKA	unsp->unsp+PKA	Uniprot|PhosphoSite	6152	RPL24	vkfqraitgaSladimakrnq
IPI00306369	586	583	PKC->			54888	NSUN2	nnsekmkvinTgikvwcrnns
IPI00306430	116	112	unsp+PKC+PKG->unsp+PKG+PKA	unsp+PKC+PKG->unsp+PKG+PKA		2115	ETV1	acsqeqpfkfSygekclynvs
IPI00306430	116	113		unsp+EGFR->EGFR		2115	ETV1	csqeqpfkfsYgekclynvsa
IPI00306430	116	119	unsp->	unsp->		2115	ETV1	fkfsygekclYnvsaydqkpq
IPI00306642	201	200		->DNAPK		25879	DCAF13	preyiralnaTklervfakpf
IPI00306708	64	57	unsp+PKA+RSK->unsp+cdc2+PKA+RSK			55872	PBK	ylmkrsprglShspwavkkin
IPI00306708	64	59	unsp+cdc2->	unsp+cdc2->	Uniprot|PhosphoSite	55872	PBK	mkrsprglshSpwavkkinpi
IPI00306708	65	57	unsp+PKA+RSK->unsp+cdc2+PKA+RSK			55872	PBK	ylmkrsprglShspwavkkin
IPI00306708	65	59		unsp+cdc2->unsp+GSK3+cdc2	Uniprot|PhosphoSite	55872	PBK	mkrsprglshSpwavkkinpi
IPI00306723	695	700	unsp->	unsp->		10153	CEBPZ	gkqlnkydpfSrnplfcgaen
IPI00306749	793	794	INSR+unsp->SRC	INSR+unsp->INSR		22950	SLC4A1AP	gdgrthlndkYgy--------
IPI00306959	101	104	unsp+PKC->unsp			3855	KRT7	qiktlnnkfaSfidkvrfleq
IPI00306960	244	243		->ATM+DNAPK		4677	NARS	rhmmirgenmSkilkarsmvt
IPI00307092	169	166	unsp+PKA->PKA			3735	KARS	vagrihakraSggklifydlr
IPI00307162	173	169	PKC->	PKC->		7414	VCL	tytknlgpgmTkmakmiderq
IPI00307162	1070	1067	PKC+cdc2->cdc2	PKC+cdc2->cdc2		7414	VCL	ptistqlkilStvkatmlgrt
IPI00307162	1070	1068	unsp+PKC->PKC	unsp+PKC->PKC		7414	VCL	tistqlkilsTvkatmlgrtn
IPI00307162	1070	1072		PKC->		7414	VCL	qlkilstvkaTmlgrtnisde
IPI00307246	289	284	->PKA	->PKA		1544	CYP1A2	dfdknsvrdiTgalfkhskkg
IPI00307259	84	80	unsp->unsp+PKA			23317	DNAJC13	ltfrkgsgkkSetlkfstehr
IPI00307259	84	82	PKC->	PKC->		23317	DNAJC13	frkgsgkkseTlkfstehrte
IPI00307259	84	86	unsp->			23317	DNAJC13	sgkksetlkfStehrtellte
IPI00307259	84	87	->CKII	->CKII		23317	DNAJC13	gkksetlkfsTehrtelltea
IPI00307259	84	91	CKII->cdc2+CKII			23317	DNAJC13	etlkfstehrTelltealrfr
IPI00307659	240	234		unsp+cdk5->unsp+GSK3+cdk5	PhosphoSite	57332	CBX8	eylkgrklddTpsgagkfpag
IPI00328161	377	376	PKC->CKI+PKC	PKC->PKC+DNAPK		23770	FKBP8	psnktihaelSklvkkhaaqr
IPI00328258	239	238	unsp+PKG->unsp+PKG+PKA	unsp+PKG->unsp+PKG+DNAPK		85403	EAF1	ppppmpfrapTkppvgpktsp
IPI00328306	764	759	unsp+PKA+RSK->unsp+PKA	unsp+PKA+RSK->unsp+PKA	Uniprot|PhosphoSite	9877	ZC3H11A	qmsmktrrlsSastgkpplsv
IPI00328306	764	761	unsp+PKC->unsp		Uniprot|PhosphoSite	9877	ZC3H11A	smktrrlssaStgkpplsved
IPI00328306	764	762	unsp+PKC->PKC	unsp+PKC->	Uniprot|PhosphoSite	9877	ZC3H11A	mktrrlssasTgkpplsvedd
IPI00328306	764	768	unsp+CKII->unsp+CKI+CKII	unsp+CKII->unsp+CKI+CKII	Uniprot|PhosphoSite	9877	ZC3H11A	ssastgkpplSveddfekliw
IPI00328319	22	21	unsp->unsp+EGFR	unsp->unsp+EGFR		5928	RBBP4	aveervineeYkiwkkntpfl
IPI00328415	42	38	cdc2->	cdc2->		1727	CYB5R3	qrstpaitleSpdikyplrli
IPI00328415	120	117	unsp+PKC->	unsp+PKC->PKC		1727	CYB5R3	dlvikvyfkdThpkfpaggkm
IPI00328526	58	62	PKA->	PKA->	Uniprot|PhosphoSite	10776	ARPP19	rlqkgqkyfdSgdynmakakm
IPI00328798	108	105	unsp->	unsp->		283149	BCL9L	knpqagvppfSslkgkvkrdr
IPI00328798	108	106	PKC->	PKC->		283149	BCL9L	npqagvppfsSlkgkvkrdrs

Table S3

IPI00328798	110	105	unsp->	unsp->		283149	BCL9L	knpqagvppfSslkgkvkrdr
IPI00328798	110	106	PKC->	PKC->		283149	BCL9L	npqagvppfsSlkgkvkrdrs
IPI00328798	137	131	->PKA	->PKA		283149	BCL9L	sgeqreagtpSldseakevap
IPI00328798	137	134	unsp->			283149	BCL9L	qreagtpsldSeakevaprsk
IPI00328815	968	962	->PKA	->PKA		84196	USP48	kvrgekallvSanqtlkelki
IPI00328815	968	966	unsp+PKC->unsp	unsp+PKC->unsp		84196	USP48	ekallvsanqTlkelkiqimh
IPI00328918	33	34		unsp+cdc2->unsp+PKC+cdc2		84062	DTNBP1	sdksreakvkSkprtvpflpk
IPI00328918	33	38	PKC->	PKC->GSK3		84062	DTNBP1	reakvkskprTvpflpkysag
IPI00328918	35	34	unsp+cdc2->unsp+PKA+cdc2	unsp+cdc2->unsp+cdc2+DNAPK		84062	DTNBP1	sdksreakvkSkprtvpflpk
IPI00328918	35	38	PKC->GSK3			84062	DTNBP1	reakvkskprTvpflpkysag
IPI00329132	233	238	unsp+PKC+CKII->PKC			79969	C6orf134	aegdikpyssSdreflkvave
IPI00329331	438	434	PKC->PKA	PKC->		7360	UGP2	fptvplvklgSsftkvqdylr
IPI00329331	438	435	PKC->PKA	PKC->		7360	UGP2	ptvplvklgsSftkvqdylrr
IPI00329331	438	442	EGFR->			7360	UGP2	lgssftkvqdYlrrfesipdm
IPI00329338	8	6	PKC->cdc2	PKC->		5130	PCYT1A	-----mdaqcSakvnarkrrk
IPI00329389	210	213	PKC->			6128	RPL6	isnvkipkhlTdayfkkkklr
IPI00329389	239	240	INSR+unsp->unsp			6128	RPL6	geifdtekekYeiteqrkidq
IPI00329512	8	6	unsp+PKC->	unsp+PKC->		6628	SNRPB	-----mtvgkSskmlqhidyr
IPI00329512	8	7	unsp+PKC+PKA->PKA	unsp+PKC+PKA->unsp+PKA+DNAPK		6628	SNRPB	----mtvgksSkmlqhidyrm
IPI00329512	32	30	unsp+PKC->PKC	unsp+PKC->PKC		6628	SNRPB	ilqdgrifigTfkafdkhmnl
IPI00329528	701	697	unsp+PKG->unsp+PKG+PKA	unsp+PKG->unsp+PKG+PKA		9730	VPRBP	ncvcgpdnriSsigkfisgtp
IPI00329528	701	698	unsp+PKC->PKA	unsp+PKC->unsp+PKA		9730	VPRBP	cvcgpdnrisSigkfisgtpr
IPI00329633	243	246	unsp+p38MAPK->unsp+cdk5+p38MAPK			6897	TARS	kcrilnekvnTptttvyrcgp
IPI00329633	243	248	PKG->			6897	TARS	rilnekvntpTttvyrcgpli
IPI00329633	306	301	unsp+PKC->	unsp+PKC->PKC		6897	TARS	metlqriygiSfpdpkmlkew
IPI00329665	6	7	unsp->	unsp->		440689	HIST2H2BF	----mpdpakSapapkkgskk
IPI00329665	12	7	unsp->	unsp->		440689	HIST2H2BF	----mpdpakSapapkkgskk
IPI00329665	12	15	unsp+PKC->PKC	unsp+PKC->PKC	Uniprot	440689	HIST2H2BF	aksapapkkgSkkavtkvqkk
IPI00329665	13	15	unsp+PKC->PKC		Uniprot	440689	HIST2H2BF	aksapapkkgSkkavtkvqkk
IPI00329665	16	15	unsp+PKC->unsp+PKC+PKA	unsp+PKC->ATM+unsp+PKC+DNAPK	Uniprot	440689	HIST2H2BF	aksapapkkgSkkavtkvqkk
IPI00329665	17	15	unsp+PKC->PKC+PKA	unsp+PKC->PKC+PKA	Uniprot	440689	HIST2H2BF	aksapapkkgSkkavtkvqkk
IPI00329665	21	15	unsp+PKC->unsp+PKC+PKA	unsp+PKC->unsp+PKC+PKA	Uniprot	440689	HIST2H2BF	aksapapkkgSkkavtkvqkk
IPI00329665	35	33	unsp+PKC+PKG+PKA+RSK->unsp+PKG+PKA+RSK	unsp+PKC+PKG+PKA+RSK->unsp+PKG+PKA+RSK		440689	HIST2H2BF	qkkdgkkrkrSrkesysvyvy
IPI00329665	35	37		unsp+PKC+PKG+PKB+PKA+RSK->unsp+PKG+PKB+PKA+RSK		440689	HIST2H2BF	gkkrkrsrkeSysvyvykvlk
IPI00329665	35	38	INSR+unsp->	INSR+unsp->	PhosphoSite	440689	HIST2H2BF	kkrkrsrkesYsvyvykvlkq
IPI00329665	35	39	unsp+PKC->unsp	unsp+PKC->unsp+PKC+cdc2		440689	HIST2H2BF	krkrsrkesySvyvykvlkqv
IPI00329665	47	53		GSK3->		440689	HIST2H2BF	ykvlkqvhpdTgisskamgim
IPI00329665	86	79	->cdc2			440689	HIST2H2BF	diferiageaSrlahynkrst
IPI00329665	86	88	PKG->	PKG->		440689	HIST2H2BF	asrlahynkrStitsreiqta
IPI00329665	86	89	unsp+PKC->	unsp+PKC->PKC		440689	HIST2H2BF	srlahynkrsTitsreiqtav
IPI00329665	109	113	unsp->			440689	HIST2H2BF	lpgelakhavSegtkavtkyt
IPI00329665	121	120		->ATM+DNAPK		440689	HIST2H2BF	havsegtkavTkytssk----
IPI00329665	121	123		PKC->		440689	HIST2H2BF	segtkavtkyTssk-------
IPI00329755	380	377	PKC+DNAPK->DNAPK	PKC+DNAPK->DNAPK		2313	FLI1	qalqphptesSmykypsdisy
IPI00329755	380	379	unsp->	unsp->unsp+EGFR		2313	FLI1	lqphptessmYkypsdisymp
IPI00329755	380	383	unsp+PKC+cdc2->cdc2	unsp+PKC+cdc2->PKC+cdc2		2313	FLI1	ptessmykypSdisympsyha
IPI00329791	263	260	PKC->PKA	PKC->PKC+PKA		9879	DDX46	gggnekksgpTvtkvvtvvtt
IPI00329791	271	269	unsp+PKC->PKC	unsp+PKC->PKC		9879	DDX46	ptvtkvvtvvTtkkavvdsdk
IPI00329791	776	771	unsp+PKC->unsp+PKA	unsp+PKC->unsp		9879	DDX46	kaegkiikksSgfsgkgfkfd
IPI00329801	76	71	CKI+cdc2->cdc2	CKI+cdc2->cdc2		308	ANXA5	fgrdllddlkSeltgkfekli
IPI00329801	76	74	unsp+PKC->	unsp+PKC->		308	ANXA5	dllddlkselTgkfeklival
IPI00332376	144	142	unsp->	unsp->DNAPK		7322	UBE2D2	ekynriarewTqkyam-----
IPI00333010	901	894	INSR+unsp->unsp	INSR+unsp->unsp	PhosphoSite	10523	CHERP	kgvgvalddpYenyrrnksys
IPI00333010	901	904	unsp+PKC->PKC	unsp+PKC->PKC	Uniprot|PhosphoSite	10523	CHERP	yenyrrnksySfiarmkarde
IPI00333201	84	82	unsp+PKC->unsp	unsp+PKC->unsp		6996	TDG	qpvepkkpveSkksgksaksk
IPI00333215	84	81	unsp->		Uniprot|PhosphoSite	6917	TCEA1	kswkklldgpStekdldekkk
IPI00333533	631	626	PKC->			367	AR	rlrkcyeagmTlgarklkklg
IPI00333541	504	500		->PKG		2316	FLNA	lqpkgvrvkeTadfkvytkga
IPI00333541	504	506	INSR->	INSR->	PhosphoSite	2316	FLNA	rvketadfkvYtkgagselk
IPI00333541	504	507	PKC->			2316	FLNA	vketadfkvyTkgagsgelkv
IPI00333541	508	506	INSR->	INSR->	PhosphoSite	2316	FLNA	rvketadfkvYtkgagsgelk
IPI00333541	508	507	PKC->	PKC->		2316	FLNA	vketadfkvyTkgagsgelkv
IPI00333541	700	696	unsp+PKC->unsp	unsp+PKC->PKC		2316	FLNA	gvavnkpaefTvdakhggkap
IPI00333541	837	838	INSR->			2316	FLNA	rndndtftvkYtprgagsyti
IPI00333541	837	839		unsp+p38MAPK->unsp+cdk5+p38MAPK		2316	FLNA	ndndtftvkyTprgagsytim
IPI00333541	973	966	unsp+GSK3+cdk5->unsp+GSK3+cdk5+p38MAPK	unsp+GSK3+cdk5->unsp+GSK3+p38MAPK		2316	FLNA	ipkspfsvavSpsldlskikv
IPI00333541	973	968	->PKA			2316	FLNA	kspfsvavspSldlskikvsg
IPI00333541	973	972		PKC->ATM+DNAPK		2316	FLNA	svavspsldlSkikvsglgek
IPI00333541	973	977	unsp->			2316	FLNA	psldlskikvSglgekvdvgk
IPI00333541	994	992	unsp+PKC->PKC	unsp+PKC->PKC		2316	FLNA	kvdvgkdqefTvkskgaggqg
IPI00333541	994	995	->CKI+PKC	->PKC		2316	FLNA	vgkdqeftvkSkgaggqgkva
IPI00333541	1071	1068	PKC+PKG->PKG			2316	FLNA	pleavaptkpSkvkafgpglq
IPI00333541	1538	1533		unsp+GSK3+cdk5+p38MAPK->GSK3+cdk5+p38MAPK	Uniprot|PhosphoSite	2316	FLNA	vlygdeevprSpfkvkvlpth
IPI00333541	2024	2032	->cdc2			2316	FLNA	hvkkngqhvaSspipvvisqs
IPI00333541	2513	2510	PKC+GSK3+cdk5+cdc2+p38MAPK->GSK3+cdc2+p38MAPK	PKC+GSK3+cdk5+cdc2+p38MAPK->PKC+GSK3+p38M	Uniprot|PhosphoSite	2316	FLNA	kyggpyhiggSpfkakvtgpr
IPI00333541	2513	2517	PKC->			2316	FLNA	iggspfkakvTgprlvsnhsl
IPI00333541	2515	2510	PKC+GSK3+cdk5+cdc2+p38MAPK->GSK3+cdk5+cdc2+p38MAPK		Uniprot|PhosphoSite	2316	FLNA	kyggpyhiggSpfkakvtgpr
IPI00333541	2515	2517		PKC->		2316	FLNA	iggspfkakvTgprlvsnhsl
IPI00333541	2607	2599		unsp+GSK3+CKII->unsp+GSK3+cdk5+CKII		2316	FLNA	mllvgvhgprTpceeilvkhv
IPI00333541	2607	2611	PKA->	PKA->		2316	FLNA	ceeilvkhvgSrlysvsyllk
IPI00333541	2621	2615		unsp+PKA+RSK->unsp+PKA+CaM-II+RSK		2316	FLNA	lvkhvgsrlySvsyllkdkge
IPI00333770	834	831	->CKII+PKA	->PKA		55619	DOCK10	qdsasgkhggSdikwvdggkp
IPI00334159	95	93	unsp->			7411	VBP1	dtvlkkldeqYqkykfmelnl
IPI00334190	145	136	->cdc2			30968	STOML2	vtqlaqttmrSelgklsldkv
IPI00334190	233	229	CKII->unsp+CKII	CKII->unsp+CKII		30968	STOML2	egkkqaqilaSeaekaeqinq
IPI00334587	318	313	unsp+PKC->	unsp+PKC->		3182	HNRNPAB	ygpgydysqgStnygksqrrg
IPI00334587	318	314	PKC+cdc2->	PKC+cdc2->cdc2		3182	HNRNPAB	gpgydysqgsTnygksqrrgg
IPI00334715	193	191	PKC->	PKC->		2909	GRLF1	vsnlynqlakTkkpivvvltk
IPI00335001	81	84	PKA->	PKA->		10169	SERF2	rppekprkhdSgaadlervtd
IPI00335385	138	130	->cdc2			28960	DCPS	pprqlndvktTvvypatekhl
IPI00335385	138	136	unsp+PKC->	unsp+PKC->		28960	DCPS	dvkttvvypaTekhlqkylrq
IPI00335421	339	333	CKI->	CKI->		63979	FIGNL1	gvkkslgasrSrgilgkfvpp
IPI00336008	126	121	cdc2->PKA+cdc2			7915	ALDH5A1	rwrevsakerSsllrkwynlm
IPI00336008	126	122	unsp+PKC->unsp+PKA	unsp+PKC->unsp+PKA		7915	ALDH5A1	wrevsakersSllrkwynlmi
IPI00336016	329	327	unsp->	unsp->		79587	CARS2	kmksklknyiTikdflktfsp
IPI00336081	608	606	unsp+PKC+PKA->PKA	unsp+PKC+PKA->PKA		4638	MYLK	awvtvhekksSrkseyllpva
IPI00336081	608	611	unsp->	unsp->		4638	MYLK	hekkssrkseYllpvapskpt
IPI00337315	7	6	unsp->			5930	RBBP6	-----mscvhYkfssklnydt
IPI00337315	7	9	PKC+PKG->PKC	PKC+PKG->PKC		5930	RBBP6	--mscvhykfSsklnydtvtf
IPI00337315	7	10	cdc2+PKA->cdc2	cdc2+PKA->cdc2		5930	RBBP6	-mscvhykfsSklnydtvtfd
IPI00337386	233	231	unsp->			55660	PRPF40A	kcpwkeyksdSgkpyyynsqt
IPI00337386	233	236	INSR+unsp->	INSR+unsp->unsp		55660	PRPF40A	eyksdsgkpyYynsqtkesrw
IPI00337386	907	899	unsp+CKI+PKC->unsp+CKI+PKC+cdc2		PHOsphoELM|PhosphoSite	55660	PRPF40A	sasehsssaeSersykkskkh
IPI00337386	907	906	unsp+PKC+PKG->unsp+PKC	unsp+PKC+PKG->unsp+PKC		55660	PRPF40A	saesersykkSkkhkkkskkr
IPI00337386	910	906	unsp+PKC+PKG->unsp+PKC+PKG+RSK			55660	PRPF40A	saesersykkSkkhkkkskkr
IPI00337386	911	913	unsp+PKC+PKG->unsp+PKC	unsp+PKC+PKG->unsp+PKC		55660	PRPF40A	ykkskkhkkkSkkrrhksdsp
IPI00337397	586	579		unsp+PKC->unsp+PKC+PKA		4928	NUP98	langafmpkkSikklvlknln
IPI00337494	437	436		unsp+PKA+RSK->ATM+unsp+DNAPK+RSK		29957	SLC25A24	nmvglfrriiSkegipglyrg
IPI00337541	70	75	unsp+GSK3+p38MAPK->GSK3+p38MAPK	unsp+GSK3+p38MAPK->GSK3+p38MAPK		23530	NNT	ifqnekrvalSpagvqnlvkq
IPI00337541	100	99	CKII->	CKII->ATM+DNAPK		23530	NNT	vvvesgageaSkfsddhyrva
IPI00337541	100	102	unsp->	unsp->		23530	NNT	esgageaskfSddhyrvagaq
IPI00337541	100	106	INSR+unsp->SRC+INSR+unsp	INSR+unsp->SRC+INSR+unsp		23530	NNT	geaskfsddhYrvagaqiqga
IPI00337541	394	385		cdc2->		23530	NNT	dlpsrmatqaStlysnnitkl
IPI00337541	394	388	INSR+unsp->unsp	INSR+unsp->unsp		23530	NNT	srmatqastlYsnnitkllka
IPI00337541	394	389	PKC->	PKC->		23530	NNT	rmatqastlySnnitkllkai
IPI00339269	502	504	PKA->	PKA->	PhosphoSite	3310	HSPA6	drstgkankiTitndkgrlsk
IPI00339274	6	2	PKC->	PKC->	Uniprot	8338	HIST2H2AC	---------mSgrgkqggkar
IPI00339384	112	104	->cdc2			51109	RDH11	vlvrkldlsdTksirafakgf
IPI00373877	247	249	PKG->	PKG->		284695	ZNF326	rkmmqpfnkpSgtfikkpkla
IPI00374657	125	124		->ATM		9218	VAPA	keakpdelmdSklrcvfempn
IPI00374975	106	103	PKC->			441531	PGAM4	ggltglnkaeTaakhgeaqvk
IPI00374975	113	118	unsp+PKC+PKG+PKA->unsp+PKC+PKA		Uniprot	441531	PGAM4	geaqvkiwrrSydvpppppmep
IPI00375358	634	642	->cdc2			5981	RFC1	fgkfsgkddgSsfkaallsgp
IPI00375380	300	301		unsp->unsp+PKC		5719	PSMD13	rqltfeeiakSakitvnevel
IPI00375731	448	450		unsp+PKC->unsp		8241	RBM10	ktinvefakgSkrdmasnegs
IPI00376005	77	74	unsp+PKC->unsp	unsp+PKC->unsp		1984	EIF5A	kgrpckivemStsktgkhgha
IPI00376005	77	75	unsp+PKC->	unsp+PKC->		1984	EIF5A	grpckivemsTsktgkhghak
IPI00376005	77	76		->ATM+DNAPK	Uniprot	1984	EIF5A	rpckivemstSktgkhghakv

IPI00376005	77	78		PKC->unsp+PKC		1984	EIF5A	ckivemstskTgkhghakvhl
IPI00376005	97	95	PKC->PKC+cdc2+CKII	PKC->PKC+CKII		1984	EIF5A	kvhlvgidifTgkkyedicps
IPI00376005	98	95	PKC->PKC+CKII	PKC->PKC+CKII		1984	EIF5A	kvhlvgidifTgkkyedicps
IPI00376119	314	310	CKII->PKA+CKII			5567	PRKACB	sgkvrfpshfSsdlkdllrnl
IPI00376119	314	311	->CKII	->CKII		5567	PRKACB	gkvrfpshfsSdlkdllrnll
IPI00376379	181	177	unsp+PKC->unsp	unsp+PKC->unsp		374454	KRT77	qimvlnnkfaSfidkvrfleq
IPI00376798	52	47	unsp+cdk5+p38MAPK->unsp+p38MAPK	unsp+cdk5+p38MAPK->unsp+p38MAPK	Uniprot|PhosphoSite	6135	RPL11	akvleqltgqTpvfskarytv
IPI00376798	52	51	PKC->	PKC->DNAPK		6135	RPL11	eqltgqtpvfSkarytvrsfg
IPI00376798	159	158	unsp+PKG+PKA+RSK->unsp+PKA+RSK	unsp+PKG+PKA+RSK->ATM+unsp+DNAPK+RSK		6135	RPL11	tgcigakhriSkeeamrwfqq
IPI00376976	182	175		PKC->CKI+PKC		50807	ASAP1	pfdkawkdyeTkftkiekekr
IPI00376976	182	178	PKC+PKG+CKII->CKII	PKC+PKG+CKII->PKG+CKII		50807	ASAP1	kawkdyetkfTkiekekreha
IPI00377245	1974	1979	unsp+PKA+RSK->PKA+RSK	unsp+PKA+RSK->PKA+RSK		8085	MLL2	pdpflkprcpSldnlavpesp
IPI00377245	2196	2187	unsp+PKG+PKA->unsp+PKG+cdc2+PKA			8085	MLL2	pysrppsrpqSrdpfaplhkp
IPI00377245	2883	2877	->PKA			8085	MLL2	pkvepapaanSlglglkpgqs
IPI00377245	2883	2887	unsp->	unsp->unsp+cdc2		8085	MLL2	slglglkpgqSmmgsrdtrmg
IPI00377245	4244	4235	unsp->unsp+cdc2			8085	MLL2	gleqklqgtpSnkedaaarkp
IPI00377245	4244	4247	unsp+PKC+p38MAPK->unsp+GSK3+p38MAPK			8085	MLL2	kedaaarkplTpkpkrvqkas
IPI00377245	4504	4508		PKA->		8085	MLL2	pvttwekgkgSevsvmltvsa
IPI00382470	191	187	PKC->	PKC->		3320	HSP90AA1	aldkiryeslTdpskldsgke
IPI00382470	191	190		unsp->ATM+unsp+DNAPK		3320	HSP90AA1	kiryesltdpSkldsgkelhi
IPI00382470	222	216	CKI->	CKI->		3320	HSP90AA1	kqdrtltivdTgigmtkadli
IPI00382470	222	221	->PKA	->DNAPK		3320	HSP90AA1	ltivdtgigmTkadlinnlgt
IPI00382470	414	406	unsp+EGFR->unsp	unsp+EGFR->unsp		3320	HSP90AA1	kkkkkkikekYidqeelnktk
IPI00382470	416	415		->DNAPK		3320	HSP90AA1	kyidqeelnkTkpiwtrnpdd
IPI00382470	436	437	CKII->CKI+PKA+CKII	CKII->PKA+CKII		3320	HSP90AA1	tneeygefykSltndwedhla
IPI00382470	529	528		->ATM+DNAPK		3320	HSP90AA1	lnisremlqqSkilkvirknl
IPI00382470	558	556	unsp+EGFR->unsp	unsp+EGFR->unsp		3320	HSP90AA1	ftelaedkenYkkfyeqfskn
IPI00382470	558	560	->unsp	->unsp+EGFR		3320	HSP90AA1	aedkenykkfYeqfskniklg
IPI00382470	565	564		->DNAPK		3320	HSP90AA1	enykkfyeqfSkniklgihed
IPI00382470	580	575	unsp+cdc2+DNAPK->cdc2+DNAPK	unsp+cdc2+DNAPK->cdc2+DNAPK		3320	HSP90AA1	kniklgihedSqnrkklsell
IPI00382470	580	582	PKA->	PKA->		3320	HSP90AA1	hedsqnrkklSellryytsas
IPI00382470	580	588	->INSR			3320	HSP90AA1	rkklsellryYtsasgdemvs
IPI00382470	600	592		unsp+cdc2+CKII->unsp+CKII		3320	HSP90AA1	sellryytsaSgdemvslkdy
IPI00382470	600	598	unsp->unsp+CKII+cdc2			3320	HSP90AA1	ytsasgdemvSlkdyctrmke
IPI00382470	600	604	PKC->	PKC->		3320	HSP90AA1	demvslkdycTrmkenqkhiy
IPI00382470	611	614	unsp->	unsp->	Uniprot|PhosphoSite	3320	HSP90AA1	trmkenqkhiYyitgetkdqv
IPI00382470	611	615	INSR->		PhosphoSite	3320	HSP90AA1	rmkenqkhiyYitgetkdqva
IPI00382470	668	665	PKC->PKC+PKA	PKC->PKC+PKA		3320	HSP90AA1	lkefegktlvSvtkeglelpe
IPI00382470	668	667		->ATM+DNAPK		3320	HSP90AA1	efegktlvsvTkeglelpede
IPI00382470	707	711	PKC->			3320	HSP90AA1	lekkvekvvvSnrlvtspcci
IPI00382470	753	749	->INSR	->INSR	PhosphoSite	3320	HSP90AA1	qalrdnstmgYmaakkhlein
IPI00383046	36	37	unsp+cdk5->unsp+cdk5+p38MAPK	unsp+cdk5->unsp+cdk5+p38MAPK		134147	CMBL	vehikayvtkSpvdagkaviv
IPI00383105	490	488		unsp+PKC->unsp		80205	CHD9	hlclqrqppsSkksdgsgtyt
IPI00383105	490	494	unsp+PKC->unsp			80205	CHD9	qppsskksdgSgtytklqntq
IPI00383105	499	491	unsp->unsp+PKA			80205	CHD9	lqrqppsskkSdgsgtytklq
IPI00383105	499	494	unsp+PKC->unsp+PKA	unsp+PKC->unsp+PKC+PKA		80205	CHD9	qppsskksdgSgtytklqntq
IPI00383105	499	496	PKC->	PKC->		80205	CHD9	psskksdgsgTytklqntqvr
IPI00383105	499	498		PKC->ATM+PKC		80205	CHD9	skksdgsgtyTklqntqvrvm
IPI00383163	81	76	unsp->unsp+PKA			29087	THYN1	rlekgvdvkfSiedlkaqpkq
IPI00384028	657	650	->cdc2			10914	PAPOLA	ppprssgnaaTsgnaatkipt
IPI00384028	657	656	PKC->	PKC->		10914	PAPOLA	gnaatsgnaaTkiptpivgvk
IPI00384028	657	660	->cdk5	->cdk5+p38MAPK		10914	PAPOLA	tsgnaatkipTpivgvkrtss
IPI00384028	666	668	PKC+PKG->PKC	PKC+PKG->		10914	PAPOLA	iptpivgvkrTssphkeespk
IPI00384028	752	754		PKC+PKA->PKA		10914	PAPOLA	panpipviknSiklrlnr---
IPI00384028	756	754	PKC+PKA->PKA	PKC+PKA->PKA		10914	PAPOLA	panpipviknSiklrlnr---
IPI00384202	552	547	unsp->unsp+PKA			221037	JMJD1C	mdpnvsdskhSianakfleta
IPI00384202	552	556	unsp+PKC->PKC			221037	JMJD1C	hsianakfleTakkdsdqswv
IPI00384428	257	262	PKG+PKA->PKA			670	BPHL	adfihkhvkgSrlhlmpegkh
IPI00384456	70	79		unsp+PKG+PKA->unsp+PKG+cdc2+PKA	Uniprot|PhosphoSite	2956	MSH6	aknlngglrrSvapaaptscd
IPI00384456	504	503	unsp->unsp+PKA	unsp->ATM+unsp+DNAPK		2956	MSH6	earcrkmahiSkydrvvrrei
IPI00384471	368	371	unsp+CKI->CKI			23774	BRD1	ymkmepvkelTgggttfsvrk
IPI00384471	418	415	unsp+PKG+cdc2+PKA+RSK->unsp+PKG+PKA	unsp+PKG+cdc2+PKA+RSK->unsp+PKG+PKA		23774	BRD1	emkngvcrkeSsvktvrstsk
IPI00384471	418	416	unsp+PKC->	unsp+PKC->		23774	BRD1	mkngvcrkesSvktvrstskv
IPI00384471	418	422	PKG->	PKG->		23774	BRD1	rkessvktvrStskvrkkakk
IPI00384471	519	524	unsp->unsp+EGFR			23774	BRD1	emkaakeklkYwqrlrhdler
IPI00384471	521	524	unsp->	unsp->		23774	BRD1	emkaakeklkYwqrlrhdler
IPI00384471	887	882	unsp+PKC+PKA+RSK->unsp+PKA+RSK	unsp+PKC+PKA+RSK->unsp+PKA+RSK		23774	BRD1	epasdvnrrtSvlfcksksvs
IPI00384707	486	479	->cdc2			124454	EARS2	lkklseglegTkysnvmkllr
IPI00384707	486	482	->PKA	->PKA		124454	EARS2	lseglegtkySnvmkllrmal
IPI00384857	8	3	PKC->	PKC->		51155	HN1	--------mtTtttfkgvdpn
IPI00384857	8	4	PKC->			51155	HN1	-------mttTttfkgvdpns
IPI00384857	8	5	unsp+PKC->	unsp+PKC->		51155	HN1	------mtttTtfkgvdpnsr
IPI00384857	8	6	unsp+CKI+PKC->CKI	unsp+CKI+PKC->		51155	HN1	-----mttttTfkgvdpnsrn
IPI00384972	104	103		->ATM+DNAPK		284058	KIAA1267	keslklqgvfSkqtvlkshpl
IPI00384972	104	106		PKC->PKC+CaM-II		284058	KIAA1267	lklqgvfskqTvlkshpllsq
IPI00384972	180	173	unsp->unsp+cdc2			284058	KIAA1267	kssthsdhdnStslnggkral
IPI00384972	180	175		CKI+cdc2->cdc2		284058	KIAA1267	sthsdhdnstSlnggkralts
IPI00384972	180	184		unsp+PKA->unsp		284058	KIAA1267	tslnggkralTssalhggemg
IPI00385042	522	518	PKC->	PKC->CKII		23560	GTPBP4	mprtakkvqrTvlekemrslg
IPI00385042	522	526	PKA->	PKA->		23560	GTPBP4	qrtvlekemrSlgvdmddkdd
IPI00385055	890	888	unsp+PKC->PKC	unsp+PKC->PKC		1496	CTNNA2	vltvkasyvaStkyqkvygta
IPI00385055	890	889		unsp+PKC->unsp+PKC+DNAPK		1496	CTNNA2	ltvkasyvasTkyqkvygtaa
IPI00385055	890	891	unsp+EGFR->unsp	unsp+EGFR->INSR+unsp+EGFR		1496	CTNNA2	vkasyvastkYqkvygtaavn
IPI00385267	163	167		unsp+PKC+PKG->unsp+PKC		6734	SRPR	gekpkekaknSkkkgakkegs
IPI00385267	169	167	unsp+PKC+PKG->	unsp+PKC+PKG->		6734	SRPR	gekpkekaknSkkkgakkegs
IPI00385267	170	167	unsp+PKC+PKG->unsp+PKG			6734	SRPR	gekpkekaknSkkkgakkegs
IPI00385449	570	574	unsp+PKC->PKC	unsp+PKC->PKC		5578	PRKCA	eavsickglmTkhpakrlgcg
IPI00386122	140	138	PKC->	PKC->		25843	MOBKL3	tldgaacllnSnkyfpsrvsi
IPI00386189	34	38	PKC->			80155	NAA15	nglkfckqilSnpkfaehget
IPI00386189	262	260	EGFR->			80155	NAA15	lqernpenwaYykglekalkp
IPI00386189	735	732	PKC->	PKC->		80155	NAA15	eskdlsdtvrTvlkqemnrlf
IPI00386189	756	753	PKC->			80155	NAA15	gatnpknfneTflkrnsdslp
IPI00386189	756	761	PKA->	PKA->		80155	NAA15	netflkrnsdSlphrlsaakm
IPI00386448	221	229	->cdc2			5970	RELA	vqkedievyfTgpgweargsf
IPI00386448	310	316	unsp+cdk5->unsp	unsp+cdk5->unsp		5970	RELA	yetfksimkkSpfsgptdprp
IPI00386448	314	311	PKC->	PKC->	PHOsphoELM|PhosphoSite	5970	RELA	krkrtyetfkSimkkspfsgp
IPI00386448	314	316	unsp+cdk5->unsp+cdk5+p38MAPK			5970	RELA	yetfksimkkSpfsgptdprp
IPI00386448	314	319	unsp->unsp+CKI			5970	RELA	fksimkkspfSgptdprpppr
IPI00386448	315	311	PKC->	PKC->	PHOsphoELM|PhosphoSite	5970	RELA	krkrtyetfkSimkkspfsgp
IPI00386448	315	316	unsp+cdk5->unsp+p38MAPK	unsp+cdk5->unsp+cdk5+p38MAPK		5970	RELA	yetfksimkkSpfsgptdprp
IPI00386448	315	319	unsp->unsp+CKI	unsp->unsp+CKI		5970	RELA	fksimkkspfSgptdprpppr
IPI00386803	36	34	unsp+PKC->	unsp+PKC->		3927	LASP1	fwhkacfhceTckmtlnmkny
IPI00386803	128	129	unsp->CKI	unsp->		3927	LASP1	nikyheefekSrmgpsggegm
IPI00386803	128	134	unsp->unsp+PKA			3927	LASP1	eefeksrmgpSggegmeperr
IPI00386998	36	31	PKC->			641522	ARL17B	lilsldtagkTtilyklklge
IPI00387159	181	176	CKI+PKC->PKC	CKI+PKC->PKC		54556	ING3	fkseallstlTsdaskentlg
IPI00387159	181	177	PKC->	PKC->		54556	ING3	kseallstltSdaskentlgc
IPI00387159	181	180	unsp+CKI->unsp+CKI+DNAPK	unsp+CKI->ATM+unsp+CKI+DNAPK		54556	ING3	allstltsdaSkentlgcrnn
IPI00394926	286	284	unsp+PKC->	unsp+PKC->		10714	POLD3	klatpaglkkSskkaepvkvl
IPI00394926	286	285		unsp+PKC+PKA->ATM+unsp+PKC+DNAPK		10714	POLD3	latpaglkksSkkaepvkvlq
IPI00394926	287	285	unsp+PKC+PKA->unsp+PKA	unsp+PKC+PKA->unsp+PKA		10714	POLD3	latpaglkksSkkaepvkvlq
IPI00394926	302	307	unsp+PKC+CKII->unsp+CKII	unsp+PKC+CKII->unsp+CKII	iniprot|PHOsphoELM|PhosphoSi	10714	POLD3	ekkrgkrvalSddetketenm
IPI00395337	294	290		cdc2->unsp+cdc2		81608	FIP1L1	qtstasrkanSsvgkwqdryg
IPI00395337	294	291	unsp+PKC->PKC			81608	FIP1L1	tstasrkansSvgkwqdrygr
IPI00395627	19	21	unsp+PKC->PKC	unsp+PKC->unsp		27101	CACYBP	eevkvllekaTrkrvrdalta
IPI00395627	85	76	PKA->cdc2+PKA			27101	CACYBP	pittgytvkiSnygwdqsdkf
IPI00395627	85	83	PKC->	PKC->		27101	CACYBP	vkisnygwdqSdkfvkiyitl
IPI00395627	85	90	INSR+EGFR->EGFR	INSR+EGFR->INSR		27101	CACYBP	wdqsdkfvkiYitltgvhqvp
IPI00395627	118	124	cdc2->PKA+cdc2			27101	CACYBP	dllvknlngkSysmivnnllk
IPI00395627	134	141		unsp+PKC->unsp+CKI+PKC		27101	CACYBP	nllkpisvegSskkvktdtvl
IPI00395627	134	142	unsp+PKC->unsp+PKC+cdc2			27101	CACYBP	llkpisvegsSkkvktdtvli
IPI00395865	4	3		->ATM+DNAPK	Uniprot	5931	RBBP7	--------maSkemfedtvee
IPI00395865	21	20	unsp->unsp+EGFR	unsp->unsp+EGFR		5931	RBBP7	tveervineeYkiwkkntpfl
IPI00396015	1371	1365	unsp+PKC+PKG->unsp+PKC+PKG+PKA	unsp+PKC+PKG->unsp+PKC+PKG+PKA		31	ACACA	tlvdhgirrlTflvaqkdfrk
IPI00396015	1617	1612	unsp+cdc2->unsp+p38MAPK	unsp+cdc2->unsp+p38MAPK		31	ACACA	qgplhgmlinTpyvtkdllqs
IPI00396015	1617	1614	unsp->unsp+EGFR	unsp->unsp+EGFR		31	ACACA	plhgmlintpYvtkdllqskr
IPI00396015	1617	1616		->ATM		31	ACACA	hgmlintpyvTkdllqskrfq
IPI00396015	1617	1622	unsp+PKC+cdc2->PKC+cdc2	unsp+PKC+cdc2->PKC+cdc2		31	ACACA	tpyvtkdllqSkrfqaqslgt

Table S3

IPI00396089	85	87	unsp+CKII->CKII			8315	BRAP	mksnpdelktTveerksseas
IPI00396154	124	122	unsp+PKC+PKG+PKA->PKG+PKA	unsp+PKC+PKG+PKA->PKG+PKA		51105	PHF20L1	icrdppgqrwSakyrydkewl
IPI00396154	124	127	INSR->	INSR->		51105	PHF20L1	pgqrwsakyrYdkewlnngrm
IPI00396154	296	294	unsp->CKII	unsp->CKII		51105	PHF20L1	hmrsknslqySakehgmpekn
IPI00396174	23	15	INSR->	INSR->		55246	CCDC25	ftsssvnssaYtiymgkdkye
IPI00396258	321	319	unsp->	unsp->		587	BCAT2	gefrvvertiTmkqllralee
IPI00396258	377	381	unsp->			587	BCAT2	rfqkelkeiqYgirahewmfp
IPI00396329	47	44	PKC->			84154	RPF2	mlikggnanaTvtkvlkdvya
IPI00396329	47	46	PKC->	PKC->		84154	RPF2	ikggnanatvTkvlkdvyalk
IPI00396329	56	53	unsp->	unsp->		84154	RPF2	atvtkvlkdvYalkkpygvly
IPI00396341	848	847		cdc2->cdc2+DNAPK		151050	C2orf67	eqarwslweqSkwhrrnsray
IPI00396341	859	858	unsp+cdc2+PKA->cdc2+PKA	unsp+cdc2+PKA->unsp+cdc2+DNAPK		151050	C2orf67	kwhrrnsraySknvegqdlll
IPI00396378	3	4		CKI->unsp+CKI		3181	HNRNPA2B1	-------mekTletvplerkk
IPI00396378	112	110	unsp+PKC->	unsp+PKC->		3181	HNRNPA2B1	eesgkpgahvTvkklfvggik
IPI00396435	17	13	unsp->		PhosphoSite	1665	DHX15	krhrldlgedYpsgkkragtd
IPI00396435	17	15	unsp+PKC->cdc2	unsp+PKC->	Uniprot|PhosphoSite	1665	DHX15	hrldlgedypSgkkragtdgk
IPI00396435	17	22	unsp+PKC->unsp	unsp+PKC->unsp		1665	DHX15	dypsgkkragTdgkdrdrdrd
IPI00396435	18	15	unsp+PKC->unsp	unsp+PKC->unsp	Uniprot|PhosphoSite	1665	DHX15	hrldlgedypSgkkragtdgk
IPI00396435	18	22	unsp+PKC->unsp			1665	DHX15	dypsgkkragTdgkdrdrdrd
IPI00396435	488	486	unsp+PKC->	unsp+PKC->		1665	DHX15	trpgkcfrlyTekayktemqd
IPI00396435	488	490		->unsp		1665	DHX15	kcfrlytekaYktemqdntyp
IPI00396435	754	749	PKC->	PKC->		1665	DHX15	fvlttknyirTctdikpewlv
IPI00396435	754	751	PKC->	PKC->		1665	DHX15	lttknyirtcTdikpewlvki
IPI00396485	55	53	PKC->	PKC->		1915	EEF1A1	ekeaaemgkgSfkyawvldkl
IPI00396485	55	56	unsp->	unsp->		1915	EEF1A1	aaemgkgsfkYawvldklkae
IPI00396485	79	76	cdc2->PKA+cdc2	cdc2->PKA+cdc2		1915	EEF1A1	erergitidiSlwkfetskyy
IPI00396485	79	82	unsp+PKC->PKC	unsp+PKC->PKC		1915	EEF1A1	tidislwkfeTskyyvtiida
IPI00396485	146	142	PKC+cdc2->cdc2	PKC+cdc2->cdc2		1915	EEF1A1	qtrehallayTlgvkqlivgv
IPI00396485	172	175	unsp->	unsp->		1915	EEF1A1	kryeeivkevStyikkigynp
IPI00396485	179	177	EGFR->			1915	EEF1A1	yeeivkevstYikkigynpdt
IPI00396485	180	175	unsp->	unsp->		1915	EEF1A1	kryeeivkevStyikkigynp
IPI00396485	180	176	PKC->			1915	EEF1A1	ryeeivkevsTyikkigynpd
IPI00396485	255	254	INSR->			1915	EEF1A1	kplrlplqdvYkiggigtvpv
IPI00396485	318	316	unsp+PKC->unsp	unsp+PKC->unsp		1915	EEF1A1	dnvgfnvknvSvkdvrrgnva
IPI00396552	279	278	INSR+unsp->unsp	INSR+unsp->INSR+unsp+EGFR		5777	PTPN6	qrpenkgknrYknilpfdhsr
IPI00396552	279	287	->cdc2			5777	PTPN6	ryknilpfdhSrvilqgrdsn
IPI00396577	1619	1617	unsp+PKC->	unsp+PKC->		57492	ARID1B	mspskspflpSmkmqkvmptv
IPI00396627	152	147	EGFR->	EGFR->		60528	ELAC2	vlsgppqlekYleaikifsgp
IPI00397358	70	69	unsp->	unsp->ATM+DNAPK		6232	RPS27	ttypyenmplTkdllhpspee
IPI00397366	322	314	->PKA			8496	PPFIBP1	evqkmkkaveSlmaaneekdr
IPI00397376	357	360	unsp+CKII->CKII	unsp+CKII->CKII		79882	ZC3H14	anknlilkaiSeaqesvtktt
IPI00397383	7	2	PKC->PKA	PKC->		23095	KIF1B	---------mSgasvkvavrv
IPI00397383	7	5	unsp+PKC+cdc2->cdc2	unsp+PKC+cdc2->cdc2		23095	KIF1B	------msgaSvkvavrvrpf
IPI00397571	253	256	PKC->			55968	NSFL1C	vkpkgafkafTgegqklgsta
IPI00397740	466	461	PKC+cdc2->cdc2	PKC+cdc2->cdc2		388567	ZNF749	shlvqhqkihTdafskrsdli
IPI00397740	466	465		unsp+PKC->unsp+PKC+DNAPK		388567	ZNF749	qhqkihtdafSkrsdliqhkr
IPI00397740	466	468	PKG->	PKG->		388567	ZNF749	kihtdafskrSdliqhkridi
IPI00397860	19	13		CKII->		1528	CYB5A	eqsdeavkyyTleeiqkhnhs
IPI00397860	24	23	cdc2+PKA->cdc2+PKA+DNAPK	cdc2+PKA->PKA+DNAPK		1528	CYB5A	tleeiqkhnhSkstwlilhhk
IPI00397860	24	26		PKC->		1528	CYB5A	eiqkhnhsksTwlilhhkvyd
IPI00398406	249	253	PKC->	PKC->		4205	MEF2A	ganslgkvmpTksppppggqn
IPI00398406	282	280	unsp+PKC->	unsp+PKC->		4205	MEF2A	kpdlrvvippSskgmmpplse
IPI00398406	282	281	unsp->GSK3	unsp->unsp+DNAPK		4205	MEF2A	pdlrvvippsSkgmmpplsee
IPI00398406	403	401	unsp+PKC->cdc2	unsp+PKC->unsp		4205	MEF2A	nlsintnqniSiksepisppr
IPI00398406	403	404		->PKC		4205	MEF2A	intnqnisikSepisppdrm
IPI00398406	403	408		unsp+GSK3+cdk5->unsp+GSK3+cdk5+p38MAPK	PHOsphoELM|PhosphoSite	4205	MEF2A	qnisiksepiSpprdrmtpsg
IPI00398749	672	677	unsp+PKA->unsp+cdc2	unsp+PKA->unsp+cdc2+PKA		23369	PUM2	pgaeakyrsaSstsslfssss
IPI00399266	168	164		PKG+PKA->unsp+PKG+PKA	Uniprot|PhosphoSite	7165	TPD52L2	hsismpamrnSatfksfedrv
IPI00402077	50	51	PKG->	PKG->		126669	SHE	wfkefplnlkTvserakpggg
IPI00402077	50	53	unsp+PKC->PKC	unsp+PKC->PKC		126669	SHE	kefplnlktvSerakpggggg
IPI00402759	20	17	PKC->	PKC->		10249	GLYAT	gaqmlqmlekSlrkslpaslk
IPI00402759	20	21		unsp+PKC+PKA->unsp+PKC+PKA+DNAPK		10249	GLYAT	lqmlekslrkSlpaslkvygt
IPI00402759	20	25	unsp+PKC->PKC+cdc2	unsp+PKC->unsp+PKC+cdc2		10249	GLYAT	ekslrkslpaSlkvygtvfhi
IPI00409671	25	22	->CKII	->CKI+CKII		11325	DDX42	rgfgfggfaiSagkkeepklp
IPI00409675	143	138	unsp+PKA->PKA	unsp+PKA->PKA		10933	MORF4L1	mrgaapgkktSglqqknvevk
IPI00409698	862	861		->EGFR		30000	TNPO2	spkddlrdmfYkilhgfkdqv
IPI00409717	293	291	PKC->	PKC->		1974	EIF4A2	lntrrkvdwlTekmhardftv
IPI00409750	232	231	unsp+PKA->PKA	unsp+PKA->ATM+unsp+PKA+DNAPK		55611	OTUB1	lltsglqreSkffehfiegg
IPI00410039	235	234		cdc2->cdc2+DNAPK		51535	PPHLN1	ltekelaeaaSkwaaekleks
IPI00410256	780	778	PKC->	PKC->		9150	CTDP1	gpevriydsnTgklirtgarg
IPI00410287	40	37	PKC->			5562	PRKAA1	yilgdtlgvgTfgkvkvgkhe
IPI00410351	1428	1424	PKC->	PKC->		9611	NCOR1	aikhnvksliTgpsklsrgmp
IPI00410351	1428	1427		PKC->		9611	NCOR1	hnvkslitgpSklsrgmpple
IPI00410351	1428	1430	unsp+PKA->unsp+PKC	unsp+PKA->unsp		9611	NCOR1	kslitgpsklSrgmppleivp
IPI00410402	40	38	PKC->	PKC->		112714	TUBA3E	hgiqpdgqmpSdktiggdds
IPI00410402	40	41	->CKI	->unsp		112714	TUBA3E	qpdgqmpsdkTiggdddsfnt
IPI00410402	60	56	PKC->	PKC->		112714	TUBA3E	ddsfntffseTgagkhvprav
IPI00410496	102	103	unsp+PKC->PKC	unsp+PKC->PKC		392490	FLJ44635	ynkcikdymkSikgkleeqrp
IPI00410616	154	162	unsp+GSK3+cdk5+PKA+p38MAPK->unsp+GSK3+cdk5+p38MAPK			252969	NEIL2	ankrgdwrdpSprlvlhfggg
IPI00410618	108	113	PKC->PKC+cdc2	PKC->PKC+cdc2		57456	KIAA1143	hpsdekysglTasskkkkpne
IPI00410693	211	205	unsp+CKI->unsp	unsp+CKI->unsp		26135	SERBP1	rhsgsdrssfShysglkhedk
IPI00410717	801	799	unsp+cdk5+PKA->unsp+cdk5+PKA+RSK	unsp+cdk5+PKA->unsp+GSK3+cdk5+PKA+RSK		23126	POGZ	hminnhvprkSpkylalfkns
IPI00410717	801	802	unsp->	unsp->		23126	POGZ	nnhvprkspkYlalfknsvsg
IPI00410717	801	809	unsp->unsp+cdc2			23126	POGZ	spkylalfknSvsgiklacts
IPI00411303	342	340	PKC->	PKC->		221016	CCDC7	kektkptnnrTkkavktvkkk
IPI00411303	348	346	unsp+PKC->PKC	unsp+PKC->PKC		221016	CCDC7	tnnrtkkavkTvkkkdkgkse
IPI00411356	8	4	PKC->	PKC->		27183	VPS4A	-------mttStlqkaidlvt
IPI00411356	8	5	PKC->			27183	VPS4A	------mttsTlqkaidlvtk
IPI00411559	363	359		->unsp		10051	SMC4	keineksnilSnemkaknkdv
IPI00411559	381	380	->PKA	->ATM		10051	SMC4	kdtekklnkiTkfieenkekf
IPI00411559	1037	1034		unsp->INSR+unsp		10051	SMC4	hiaehnskikYwhkeiskisl
IPI00411559	1037	1040	PKA->	PKA->		10051	SMC4	skikywhkeiSkislhpiedn
IPI00411614	1127	1122	unsp+PKC->PKC+PKA	unsp+PKC->PKC		11169	WDHD1	pldfstnqklSafafkqe---
IPI00411706	10	7	->PKA			2098	ESD	----malkqiSsnkcfgglqk
IPI00411706	39	42	unsp+PKC->unsp			2098	ESD	favylppkaeTgkcpalywls
IPI00411706	200	197	PKC->	PKC->		2098	ESD	afsgylgtdqSkwkaydathl
IPI00411706	200	205		PKC->		2098	ESD	dqskwkaydaThlvksypgsq
IPI00411937	505	502	unsp+CKI->CKI			10528	NOP56	qengmedpsiSfskpkkkksf
IPI00411937	505	504	PKC+cdc2->CKI+PKC+cdc2	PKC+cdc2->ATM+PKC+cdc2+DNAPK		10528	NOP56	ngmedpsisfSkpkkkksfsk
IPI00411937	507	500	unsp->unsp+cdc2			10528	NOP56	vpqengmedpSisfskpkkkk
IPI00411937	507	502	unsp+CKI->	unsp+CKI->		10528	NOP56	qengmedpsiSfskpkkkksf
IPI00411937	507	511	unsp+PKG+PKA+RSK->unsp	unsp+PKG+PKA+RSK->unsp+RSK	Uniprot|PhosphoSite	10528	NOP56	isfskpkkkkSfskeelmssd
IPI00411937	507	513	unsp->unsp+PKA		PhosphoSite	10528	NOP56	fskpkkkksfSkeelmssdle
IPI00412404	220	223	unsp+PKC->unsp+cdc2	unsp+PKC->unsp+PKC+cdc2		6832	SUPV3L1	ktyhaiqkyfSaksgvycgpl
IPI00412415	973	967		DNAPK->ATM+DNAPK		11177	BAZ1A	rgrssnaydpSqmcaekqlel
IPI00412441	253	250	->CKII			23063	WAPAL	asisscnkliTsdkvenfhee
IPI00412441	253	251	PKC+CKII->CKII	PKC+CKII->CKII		23063	WAPAL	sisscnklitSdkvenfheeh
IPI00412441	689	688	PKC->	PKC->DNAPK		23063	WAPAL	dgvfkapappSkviktvtipt
IPI00412441	689	693	PKC->			23063	WAPAL	apappskvikTvtiptqpyqd
IPI00412579	62	57	unsp+PKC+RSK->unsp+PKC+PKA+RSK	unsp+PKC+RSK->unsp+PKC+PKA+RSK		4736	RPL10A	krfsgtvrlkStprpkfsvcv
IPI00412579	62	64	PKG+PKA->	PKG+PKA->		4736	RPL10A	rlkstprpkfSvcvlgdqqhc
IPI00412607	43	42	PKC->	PKC->DNAPK		11224	RPL35	rvakvtggaaSklskirvvrk
IPI00412607	43	45	unsp+PKC->PKC			11224	RPL35	kvtggaasklSkirvvrksia
IPI00413022	482	476	unsp+CKI+GSK3+p38MAPK->unsp+p38MAPK		PhosphoSite	54476	RNF216	sdaikkwqelSpetsgkrkkr
IPI00413022	482	479	CKI+PKC->			54476	RNF216	ikkwqelspeTsgkrkkrkqm
IPI00413022	482	480	unsp+PKC->	unsp+PKC->		54476	RNF216	kkwqelspetSgkrkkrkqmn
IPI00413265	548	543	unsp+PKC->	unsp+PKC->		23137	SMC5	lrvnaviapkSsyadkapsrs
IPI00413265	548	544	unsp->unsp+PKA			23137	SMC5	rvnaviapksSyadkapsrsl
IPI00413265	548	551	unsp->unsp+cdc2	unsp->unsp+cdc2		23137	SMC5	pkssyadkapSrslnelkqyg
IPI00413265	548	553	unsp+PKA->unsp	unsp+PKA->unsp		23137	SMC5	ssyadkapsrSlnelkqygff
IPI00413324	74	79	PKC->	PKC->		6139	RPL17	rcaqakqwgwTqgrwpkksae
IPI00413344	19	23	unsp+PKC+PKA->unsp+PKA	unsp+PKC+PKA->unsp+PKA		1073	CFL2	kvfndmkvrkSstqeeikkrk
IPI00413344	92	89		unsp->INSR+unsp	Uniprot|PhosphoSite	1073	CFL2	dcryalydatYetkeskkedl
IPI00413344	92	91		CKI+PKC->CKI+DNAPK		1073	CFL2	ryalydatyeTkeskkedlvf
IPI00413344	92	94	unsp+PKC+PKG+CKII->unsp+PKC+CKII	unsp+PKC+PKG+CKII->unsp+CKII		1073	CFL2	lydatyetkeSkkedlvfifw
IPI00413344	96	91	CKI+PKC->	CKI+PKC->PKC		1073	CFL2	ryalydatyeTkeskkedlvf
IPI00413344	96	94	unsp+PKC+PKG+CKII->unsp+CKII	unsp+PKC+PKG+CKII->unsp+PKA+CKII		1073	CFL2	lydatyetkeSkkedlvfifw
IPI00413365	1275	1267		unsp+GSK3+p38MAPK->unsp+GSK3+cdk5+p38MAPK		24149	ZNF318	lqlkeevkkeSptsssfgkfs

IPI00413611	712	719	->cdc2	->cdc2		7150	TOP1	eenkqialgtSklnyldprit
IPI00413627	406	402	PKC->	PKC->		8521	GCM1	tyashphqqySlpsksskwdf
IPI00413627	406	405		PKC->ATM+PKC+DNAPK		8521	GCM1	shphqqyslpSksskwdfeee
IPI00413627	406	408		unsp+PKC->unsp		8521	GCM1	hqqyslpsksSkwdfeeemty
IPI00413627	409	402	PKC->PKC+cdc2			8521	GCM1	tyashphqqySlpsksskwdf
IPI00413627	409	405	PKC->			8521	GCM1	shphqqyslpSksskwdfeee
IPI00413627	409	407	unsp->			8521	GCM1	phqqyslpskSskwdfeeemt
IPI00413627	409	408	unsp+PKC->unsp	unsp+PKC->ATM+unsp+DNAPK		8521	GCM1	hqqyslpsksSkwdfeeemty
IPI00413641	86	82	PKC->			231	AKR1B1	elfivsklwcTyhekglvkga
IPI00413641	95	98	->cdc2	->cdc2		231	AKR1B1	lvkgacqktlSdlkldyldly
IPI00413641	263	264		unsp->		231	AKR1B1	mqrnlvvipkSvtperiaenf
IPI00414320	348	346	INSR->	INSR->unsp+INSR		311	ANXA11	qqillsfktaYgkdlikdlks
IPI00414320	355	359		CKI->CKI+cdc2		311	ANXA11	dlikdlkselSgnfektilal
IPI00414320	579	577	INSR->			311	ANXA11	ldirseykrmYgkslyhdisg
IPI00414320	579	582	unsp->		Uniprot|PhosphoSite	311	ANXA11	eykrmygkslYhdisgdtsgd
IPI00414463	204	199	unsp+CKII->unsp+PKA+CKII			51451	LCMT1	avigadlrdlSeleeklkkcn
IPI00414676	275	276	unsp+EGFR->unsp	unsp+EGFR->unsp		3326	HSP90AB1	kkktkkikekYidqeelnktk
IPI00414676	284	276	unsp+EGFR->unsp	unsp+EGFR->unsp		3326	HSP90AB1	kkktkkikekYidqeelnktk
IPI00414676	306	307	PKA+CKII->CKI+cdc2+PKA+CKII	PKA+CKII->cdc2+PKA+CKII	Uniprot|PhosphoSite	3326	HSP90AB1	tqeeygefykSltndwedhla
IPI00414676	399	398		->ATM+DNAPK		3326	HSP90AB1	lnisremlqqSkilkvirkni
IPI00414676	435	430	->INSR	->INSR		3326	HSP90AB1	aedkenykkfYeafsknlklg
IPI00414676	435	434		->DNAPK		3326	HSP90AB1	enykkfyeafSknlklgihed
IPI00414676	481	479	unsp+PKC+PKG->	unsp+PKC+PKG->		3326	HSP90AB1	lseyvsrmkeTqksiyyitge
IPI00414676	481	482	unsp+PKA->PKA	unsp+PKA->PKA		3326	HSP90AB1	yvsrmketqkSiyyitgeske
IPI00414676	481	484	unsp->	unsp->	Uniprot|PhosphoSite	3326	HSP90AB1	srmketqksiYyitgeskeqv
IPI00414676	573	579	->PKA			3326	HSP90AB1	eildkkvekvTisnrlvsspc
IPI00414676	623	619	->INSR	->INSR	PhosphoSite	3326	HSP90AB1	qalrdnstmgYmmakkhlein
IPI00418169	122	123	->p38MAPK	->p38MAPK		302	ANXA2	letvilgllkTpaqydaselk
IPI00418169	133	130	PKC+cdc2->	PKC+cdc2->		302	ANXA2	llktpaqydaSelkasmkglg
IPI00418169	166	169	INSR+unsp->	INSR+unsp->		302	ANXA2	qeinrvykemYktdlekdiis
IPI00418169	170	169	INSR+unsp->unsp	INSR+unsp->INSR+unsp+EGFR		302	ANXA2	qeinrvykemYktdlekdiis
IPI00418169	170	171		CKII->unsp+CKII		302	ANXA2	inrvykemykTdlekdiisdt
IPI00418169	175	169	INSR+unsp->unsp	INSR+unsp->unsp		302	ANXA2	qeinrvykemYktdlekdiis
IPI00418169	175	171		CKII->PKG+CKII		302	ANXA2	inrvykemykTdlekdiisdt
IPI00418169	175	179	unsp+CKII->CKII	unsp+CKII->unsp+cdc2+CKII		302	ANXA2	yktdlekdiiSdtsgdfrklm
IPI00418169	297	293	unsp->INSR+unsp	unsp->INSR+unsp	Uniprot|PhosphoSite	302	ANXA2	nkplyfadrlYdsmkgkgtrd
IPI00418169	297	295	unsp+PKC->	unsp+PKC->		302	ANXA2	plyfadrlydSmkgkgtrdkv
IPI00418169	297	301	unsp+PKC->PKC	unsp+PKC->PKC		302	ANXA2	rlydsmkgkgTrdkvlirimv
IPI00418169	299	295	unsp+PKC->unsp			302	ANXA2	plyfadrlydSmkgkgtrdkv
IPI00418169	299	301	unsp+PKC->PKC			302	ANXA2	rlydsmkgkgTrdkvlirimv
IPI00418169	331	329	INSR->	INSR->unsp+INSR		302	ANXA2	lkirsefkrkYgkslyyyiqq
IPI00418169	331	332	PKA->PKC+PKA	PKA->PKC+PKA		302	ANXA2	rsefkrkygkSlyyyiqqdtk
IPI00418174	40	32	PKG->PKG+PKA			154791	C7orf55	saatgrpyrdTaayrylvkaf
IPI00418213	329	330	PKG+CKII->CKII	PKG+CKII->CKII		27342	RABGEF1	fieflktfhkTgqeiykqtkl
IPI00418213	348	352	unsp+PKA+RSK->unsp	unsp+PKA+RSK->unsp+RSK		27342	RABGEF1	legmhykrdlSieeqsecaqd
IPI00418316	440	439	->PKA	->ATM+DNAPK		23613	ZMYND8	dmtaspkilmSkpvlsggtgr
IPI00418316	440	444		unsp->unsp+cdc2		23613	ZMYND8	pkilmskpvlSggtgrrisls
IPI00418316	440	447	PKC->PKC+cdc2			23613	ZMYND8	lmskpvlsggTgrrislsdmp
IPI00418471	445	436	->cdc2		PhosphoSite	7431	VIM	tnldslplvdThskrtllikt
IPI00418471	445	441	PKC->PKA	PKC->PKC+PKA		7431	VIM	lplvdthskrTlliktvetrd
IPI00419235	137	138	unsp->	unsp->		2949	GSTM5	dpdfeklkpkYleelpeklkl
IPI00419237	221	225	PKC->			51056	LAP3	faeiieknlkSasssktevhir
IPI00419237	221	227	unsp->unsp+cdc2	unsp->unsp+cdc2		51056	LAP3	eiieknlksaSsktevhirpk
IPI00419249	57	56	CKII->PKA	CKII->ATM+CKII+DNAPK		5684	PSMA3	vvfgveklvlSklyeegsnkr
IPI00419249	206	199	INSR+unsp->unsp	INSR+unsp->unsp		5684	PSMA3	divkevakiiYivhdevkdka
IPI00419249	238	243	unsp+PKC+PKG+PKA+CKII->unsp+CKI+PKC+CKII	unsp+PKC+PKG+PKA+CKII->unsp+PKG+PKA+CKII	Uniprot|PHOsphoELM	5684	PSMA3	reeaekyakeSlkeedesddd
IPI00419258	12	14	unsp+PKG+PKA->	unsp+PKG+PKA->unsp		3146	HMGB1	gdpkkprgkmSsyaffvqtcr
IPI00419258	12	16	unsp->	unsp->		3146	HMGB1	pkkprgkmssYaffvqtcree
IPI00419258	30	35	unsp+PKC->	unsp+PKC->	Uniprot|PhosphoSite	3146	HMGB1	eehkkkhpdaSvnfsefskkc
IPI00419258	43	39	PKC->unsp	PKC->unsp+PKC	PhosphoSite	3146	HMGB1	kkhpdasvnfSefskkcserw
IPI00419258	43	42		unsp+CKI+PKC->ATM+unsp+CKI+PKC+DNAPK	PhosphoSite	3146	HMGB1	pdasvnfsefSkkcserwktm
IPI00419258	44	39	PKC->		PhosphoSite	3146	HMGB1	kkhpdasvnfSefskkcserw
IPI00419258	44	42	unsp+CKI+PKC->CKI	unsp+CKI+PKC->CKI	PhosphoSite	3146	HMGB1	pdasvnfsefSkkcserwktm
IPI00419258	82	77	PKC->			3146	HMGB1	dkaryeremkTyipgkgetkk
IPI00419258	82	78	unsp->INSR+unsp	unsp->INSR+unsp+EGFR	PhosphoSite	3146	HMGB1	karyeremktYippkgetkkk
IPI00419258	146	144	INSR+unsp->unsp			3146	HMGB1	nntaaddkqpYekkaaklkek
IPI00419258	154	155	unsp->	unsp->		3146	HMGB1	ekkaaklkekYekdiaayrak
IPI00419258	157	155	unsp->			3146	HMGB1	ekkaaklkekYekdiaayrak
IPI00419273	641	642	unsp+GSK3->unsp+GSK3+p38MAPK	unsp+GSK3->unsp+p38MAPK		8451	CUL4A	acgkarvlikSpkgkevedgd
IPI00419373	134	137	->CKII	->CKII		220988	HNRNPA3	kifvggikedTeeynlrdyfe
IPI00419373	148	145		->EGFR		220988	HNRNPA3	edteeynlrdYfekygkieti
IPI00419373	148	149	unsp->	unsp->INSR+unsp		220988	HNRNPA3	eynlrdyfekYgkietievme
IPI00419373	148	154		CKII->		220988	HNRNPA3	dyfekygkieTievmedrqsg
IPI00419541	404	401	PKC->	PKC->		115361	GBP4	nhefqkklvdTiekkkgdfvl
IPI00419585	44	40	unsp+PKC+PKG+PKA+RSK->unsp+PKG+PKA+RSK	unsp+PKC+PKG+PKA+RSK->unsp+PKG+PKA+RSK		5478	PPIA	pktaenfralStgekgfgykg
IPI00419585	44	41	PKC->			5478	PPIA	ktaenfralsTgekgfgykgs
IPI00419585	49	51	PKA->PKC	PKA->		5478	PPIA	tgekgfgykgScfhriipgfm
IPI00419585	76	73	PKC->			5478	PPIA	qggdftrhngTggksiygekf
IPI00419585	76	79	EGFR->			5478	PPIA	rhngtggksiYgekfedenfi
IPI00419585	118	119		->CKII		5478	PPIA	gsqffictakTewldgkhvvf
IPI00419643	221	215		->cdc2		253558	LCLAT1	tdltensksrSnafaeknglq
IPI00419833	6	7		unsp->		85236	HIST1H2BK	----mpepakSapakkgskk
IPI00419833	12	7	unsp->	unsp->		85236	HIST1H2BK	----mpepakSapakkgskk
IPI00419833	12	15	unsp+PKC->PKC	unsp+PKC->PKC	Uniprot	85236	HIST1H2BK	aksapakkgSkkavtkaqkk
IPI00419833	13	15	unsp+PKC->PKC		Uniprot	85236	HIST1H2BK	aksapakkgSkkavtkaqkk
IPI00419833	16	15	unsp+PKC->unsp+PKC+PKA	unsp+PKC->ATM+unsp+PKC+DNAPK	Uniprot	85236	HIST1H2BK	aksapakkgSkkavtkaqkk
IPI00419833	21	15	unsp+PKC->unsp+PKC+PKA	unsp+PKC->unsp+PKC+PKA	Uniprot	85236	HIST1H2BK	aksapakkgSkkavtkaqkk
IPI00419833	21	20		PKC->PKC+DNAPK		85236	HIST1H2BK	apkkgskkavTkaqkkdgkkr
IPI00419833	109	113	unsp->			85236	HIST1H2BK	lpgelakhavSegtkavtkyt
IPI00419844	428	426	unsp+CKI+PKG->CKI+CKII	unsp+CKI+PKG->CKII		10713	USP39	fnilakfngiTekeyktyken
IPI00419844	428	433	INSR->	INSR->		10713	USP39	ngitekeyktYkenflkrfql
IPI00419880	27	26	unsp+PKC->unsp	unsp+PKC->unsp+DNAPK		6189	RPS3A	gakkkvvdpfSkkdwydvkap
IPI00419880	249	246	->CKII			6189	RPS3A	sssgkatgdeTgakveradgy
IPI00419884	10	12	unsp+PKC+PKG->PKC	unsp+PKC+PKG->unsp+PKC	Uniprot	440093	H3F3C	artkqtarksTggkaprkqla
IPI00419884	15	11		unsp->unsp+PKG	Uniprot	440093	H3F3C	martkqtarkStggkaprkql
IPI00419884	15	12	unsp+PKC+PKG->PKG+PKA	unsp+PKC+PKG->PKC+PKG	Uniprot	440093	H3F3C	artkqtarksTggkaprkqla
IPI00419919	33	29		unsp->unsp+EGFR		387101	RPL29P4	ngikkprsqrYeslkgvdpkf
IPI00419919	33	31	unsp->unsp+cdc2+PKA	unsp->unsp+cdc2+PKA	PhosphoSite	387101	RPL29P4	ikkprsqryeSlkgvdpkflr
IPI00419928	110	105	PKC+PKA->PKA			339745	SPOPL	ksevrakfkfSllnakreetk
IPI00419979	38	36	unsp+PKC->	unsp+PKC->DNAPK		5062	PAK2	ggkdplsanhSlkplpsvpee
IPI00419979	38	42	unsp->CKII			5062	PAK2	sanhslkplpSvpeekkprhk
IPI00419979	128	132		->cdc2		5062	PAK2	avldvlkfydSntvkqkylsf
IPI00420014	1176	1173	PKG->			23020	SNRNP200	elirmpkmgkTihkyvhlfpk
IPI00420065	378	376	unsp+PKC->	unsp+PKC->		84129	ACAD11	idttgqlfvqTrkgqevlikv
IPI00420071	396	388	unsp+PKC+PKG->unsp+PKC+PKG+PKA			4135	MAP6	svqsskpkktSashkptrkak
IPI00420071	396	394	PKC+PKG->PKC	PKC+PKG->		4135	MAP6	pkktsashkpTrkakdkqavs
IPI00430411	190	189		PKC->		814	CAMK4	aplkiadfglSkivehqvlmk
IPI00430472	572	571		unsp+cdc2+CKII->ATM+unsp+cdc2+CKII+DNAPK		10973	ASCC3	vkeltgdmqlSkseilrtqml
IPI00430472	572	573	->cdc2	->cdc2		10973	ASCC3	eltgdmqlskSeilrtqmlvt
IPI00430803	134	132	EGFR->			391356	C2orf79	rpypkeevgqYlkkfrlfk--
IPI00430812	103	99	->INSR	->INSR+EGFR		7555	CNBP	epkrereqccYncgkpghlar
IPI00433834	77	74	->EGFR	->EGFR		6154	RPL26	gqqigkvvqvYrkkyviyier
IPI00438229	266	269	PKC->			10155	TRIM28	lvkrlgdkhaTlqkstkevrs
IPI00438701	422	420	unsp+PKC+cdc2->cdc2	unsp+PKC+cdc2->cdc2		4152	MBD1	ssarrhhlgpTlkptlatrta
IPI00438701	422	424	PKG+PKA->	PKG+PKA->		4152	MBD1	rhhlgptlkpTlatrtaqpdh
IPI00439194	109	104	unsp+PKC->unsp+PKA	unsp+PKC->unsp		53615	MBD3	dlntalpvrqTasifkqpvtk
IPI00439194	109	106	PKC->PKA			53615	MBD3	ntalpvrqtaSifkqpvtkit
IPI00439194	141	144	PKA->	PKA->	Uniprot	53615	MBD3	prqlfwekklSglnafdiaee
IPI00439548	392	389	unsp+PKC+PKG+PKA->PKG+PKA	unsp+PKC+PKG+PKA->unsp+CKI+PKG+PKA		54880	BCOR	vssefpaarlSngkypkapeg
IPI00439548	392	393	unsp+EGFR->unsp			54880	BCOR	fpaarlsngkYpkapeggega
IPI00440484	1096	1089	ATM+unsp+PKC+PKA+DNAPK->unsp+PKC+PKA+DNAPK	ATM+unsp+PKC+PKA+DNAPK->unsp+PKC+PKA+DNAPK		5187	PER1	gstsasitrsSqsshtskyfg
IPI00440484	1096	1091	unsp+PKC->unsp			5187	PER1	tsasitrssqSshtskyfgsi
IPI00440484	1096	1092	unsp+CKI+PKC->unsp+CKI	unsp+CKI+PKC->unsp+CKI		5187	PER1	sasitrssqsShtskyfgsid
IPI00440484	1096	1095	unsp+cdc2->cdc2	unsp+cdc2->ATM+unsp+cdc2+DNAPK		5187	PER1	itrssqsshtSkyfgsidsse
IPI00440484	1096	1104	unsp+CKII->unsp+cdc2+CKII		Uniprot	5187	PER1	tskyfgsidsSeaeagaargg
IPI00440493	132	134	PKG+PKA->	PKG+PKA->		498	ATP5A1	likegdivkrTgaivdvpvge
IPI00440493	230	225	PKC->	PKC->		498	ATP5A1	qtgktsiaidTiinqkrfndg
IPI00440493	239	236	unsp+PKC->CKII	unsp+PKC->unsp		498	ATP5A1	iinqkrfndgSdekkklyciy

IPI00440493	239	243	INSR->			498	ATP5A1	ndgsdekkklYciyvaigqkr
IPI00440493	240	236	unsp+PKC->unsp	unsp+PKC->unsp		498	ATP5A1	iinqkrfndgSdekkklyciy
IPI00440493	240	243	INSR->	INSR->		498	ATP5A1	ndgsdekkklYciyvaigqkr
IPI00440493	261	254	PKA->cdc2+PKA			498	ATP5A1	ciyvaigqkrStvaqlvkrlt
IPI00440493	261	264	unsp->			498	ATP5A1	stvaqlvkrlTdadamkytiv
IPI00440493	427	423	PKC->			498	ATP5A1	svsrvgsaaqTramkqvagtm
IPI00440493	434	432	PKC+cdc2->cdc2	PKC+cdc2->cdc2		498	ATP5A1	qtramkqvagTmklelaqyre
IPI00440493	498	502	->CKI	->CKI		498	ATP5A1	vrgyldklepSkitkfenafl
IPI00440493	498	505	unsp->unsp+cdc2			498	ATP5A1	yldklepskiTkfenaflshv
IPI00440493	506	502	->PKA	->unsp		498	ATP5A1	vrgyldklepSkitkfenafl
IPI00440493	506	505	unsp->	unsp->		498	ATP5A1	yldklepskiTkfenaflshv
IPI00440493	539	536	->CKII			498	ATP5A1	iradgkiseqSdaklkeivtn
IPI00440727	1114	1117	unsp->GSK3	unsp->	niprot|PHOsphoELM|PhosphoSi	23476	BRD4	lvvvkeekihSpiirsepfsp
IPI00441473	200	199	unsp->PKA	unsp->ATM+unsp+DNAPK		10419	PRMT5	whnfrtlcdySkriavaleig
IPI00442073	108	107	unsp+PKC->unsp	unsp+PKC->unsp+DNAPK		1465	CSRP1	ghrpttnpnaSkfaqkiggse
IPI00442073	112	107	unsp+PKC->unsp			1465	CSRP1	ghrpttnpnaSkfaqkiggse
IPI00442073	112	116	unsp+PKC->			1465	CSRP1	askfaqkiggSercprcsqav
IPI00442073	131	123		PKG+PKA+DNAPK->ATM+PKG+PKA+DNAPK		1465	CSRP1	iggsercprcSqavyaaekvi
IPI00444452	148	152	unsp+PKC->unsp			4343	MOV10	rldlnrkevlTlrlrnggtqs
IPI00444646	647	639	PKC->PKC+cdc2			11276	SYNRG	apsllmplpgTkalpsmdkya
IPI00444646	647	644	unsp+PKC->	unsp+PKC->unsp		11276	SYNRG	mplpgtkalpSmdkyavfkgi
IPI00444646	878	873	PKC->	PKC->		11276	SYNRG	gstvkggqnsTaastkydvfr
IPI00444646	878	876	unsp+PKC->	unsp+PKC->		11276	SYNRG	vkggqnstaaStkydvfrqls
IPI00444646	878	877		unsp->unsp+DNAPK		11276	SYNRG	kggqnstaasTkydvfrqlsl
IPI00444646	878	879	unsp->	unsp->		11276	SYNRG	gqnstaastkYdvfrqlsleg
IPI00444646	878	886	unsp+CKI+PKA->unsp+CKI	unsp+CKI+PKA->unsp+CKI		11276	SYNRG	stkydvfrqlSlegsglgved
IPI00446767	237	234	->CKII			7091	TLE4	dysseskkqkTeekeiaaryd
IPI00446767	281	282	->PKC	->PKC		7091	TLE4	hsprengldkTrllkkdapis
IPI00446986	3	4	unsp->	unsp->	PhosphoSite	2533	FYB	-------makYntggnptedv
IPI00446986	149	155	->PKA			2533	FYB	dhdlkplgpkSgptpptsene
IPI00446986	265	256		cdc2+DNAPK->DNAPK		2533	FYB	enkdhageisSlpfpgvvlkp
IPI00446986	265	269	unsp->	unsp->unsp+cdc2		2533	FYB	fpgvvlkpaaSrggpglskng
IPI00448465	39	36	unsp+PKC+DNAPK->unsp+DNAPK			23253	ANKRD12	ygrkskdkiaSysktpkiers
IPI00448465	39	38	PKC->CKI	PKC->ATM+DNAPK		23253	ANKRD12	rkskdkiasySktpkiersdv
IPI00448798	8	3	PKC->	PKC->	Uniprot|PhosphoSite	1452	CSNK1A1	--------maSssgskaefiv
IPI00448798	8	4	CKI+PKC->CKI	CKI+PKC->CKI		1452	CSNK1A1	-------masSsgskaefivg
IPI00448798	8	5	unsp+PKC+cdc2->cdc2	unsp+PKC+cdc2->unsp+cdc2		1452	CSNK1A1	------massSgskaefivgg
IPI00448798	8	7	unsp+PKC->unsp+DNAPK	unsp+PKC->ATM+unsp+DNAPK		1452	CSNK1A1	----masssgSkaefivggky
IPI00449049	105	104	PKC+CKII->CKII+DNAPK	PKC+CKII->CKII+DNAPK		142	PARP1	vtgkgqdgigSkaektlgdfa
IPI00449049	498	499		unsp+PKA+RSK->PKA+RSK		142	PARP1	pvevvaprgkSgaalskksk g
IPI00449049	505	504		unsp+PKC->unsp+PKC+DNAPK		142	PARP1	aprgksgaalSkkskgqvkee
IPI00449049	505	507	unsp+CKI+PKC->CKI+PKC			142	PARP1	gksgaalskkSkgqvkeegin
IPI00449049	508	507	unsp+CKI+PKC->CKI+PKC+PKA			142	PARP1	gksgaalskkSkgqvkeegin
IPI00449049	521	519	unsp+PKC->	unsp+PKC->		142	PARP1	gqvkeeginkSekrmkltlkg
IPI00449049	521	526	unsp+PKC+PKG+PKA->unsp+PKC	unsp+PKC+PKG+PKA->unsp+PKC+PKG+cdc2		142	PARP1	inksekrmklTlkggaavdpd
IPI00449049	524	526	unsp+PKC+PKG+PKA->unsp+PKC	unsp+PKC+PKG+PKA->unsp+PKC		142	PARP1	inksekrmklTlkggaavdpd
IPI00449049	548	554	->cdc2+PKA			142	PARP1	hvlekggkvfSatlglvdivk
IPI00449049	551	554	->cdc2	->cdc2		142	PARP1	hvlekggkvfSatlglvdivk
IPI00449049	600	598	PKC+cdc2->CKII+cdc2	PKC+cdc2->CKII+cdc2		142	PARP1	swgrvgtvigSnkleqmpske
IPI00449049	600	606	unsp->unsp+CKI+cdc2			142	PARP1	igsnkleqmpSkedaiehfmk
IPI00449197	352	353		->unsp		51292	GMPR2	fptlrasegkTvevpfkgdve
IPI00449923	774	776	PKA->	PKA->		10743	RAI1	tkgleqggkaSdgiskgdthe
IPI00449923	1083	1077		unsp+cdk5+p38MAPK->unsp+GSK3+cdk5+p38MAPK		10743	RAI1	rtpgppglttTpappdklggk
IPI00450472	65	68	INSR+unsp+EGFR->INSR+unsp	INSR+unsp+EGFR->INSR+unsp		7329	UBE2I	fklrmlfkddYpsspkckfe
IPI00450472	65	71	GSK3+cdk5->GSK3+cdk5+cdc2			7329	UBE2I	rmlfkddypsSppkckfeppl
IPI00451401	156	159	PKA->	PKA->		7167	TPI1	kviadnvkdwSkvvlayepvw
IPI00452463	663	665	PKC+PKG->PKC	PKC+PKG->PKC		9666	DZIP3	vkskqrkkkkTknkknkdske
IPI00452463	664	665	PKC+PKG->PKC	PKC+PKG->PKC		9666	DZIP3	vkskqrkkkkTknkknkdske
IPI00453473	6	2	PKC->		Uniprot|PHOsphoELM|PhosphoSi	121504	HIST4H4	---------mSgrgkggkglg
IPI00453473	32	31	PKC+cdc2->cdc2	PKC+cdc2->cdc2		121504	HIST4H4	kvlrdniqgiTkpairrlarr
IPI00453473	92	97	PKC+PKA->PKC	PKC+PKA->PKC		121504	HIST4H4	vvyalkrqgrTlygfgg----
IPI00454695	12	15	unsp+PKC->PKC	unsp+PKC->PKC	Uniprot			akfapakkgSkkavtkaqkk
IPI00454695	13	15	unsp+PKC->PKC		Uniprot			akfapakkgSkkavtkaqkk
IPI00455210	115	111		unsp+CKI+cdk5+p38MAPK->CKI+cdk5+p38MAPK		1108	CHD4	lrsdsegsdyTpgkkkkkklg
IPI00455210	304	303		PKC->ATM+PKC+DNAPK		1108	CHD4	plkiklggfgSkrkrsssedd
IPI00455210	304	309	unsp+PKA->unsp		PHOsphoELM|PhosphoSite	1108	CHD4	ggfgskrkrsSsedddldves
IPI00455210	884	883	->PKA	->DNAPK		1108	CHD4	deahrlknnqSkffrvlngys
IPI00455210	1671	1676	unsp+PKA->unsp	unsp+PKA->unsp		1108	CHD4	aadvekveekSaidltpivve
IPI00455423	146	145	PKC+cdc2->cdc2	PKC+cdc2->cdc2+DNAPK		440577	LOC440577	sgkrsapgggSkvpqkkvkla
IPI00455620	181	177	unsp->unsp+PKA	unsp->unsp+PKA		22902	RUFY3	lrlalmqkklSeymkalinkk
IPI00455620	181	179	INSR->	INSR->		22902	RUFY3	lalmqkklseYmkalinkkel
IPI00455623	136	137	unsp->SRC+unsp	unsp->SRC+unsp		84779	NAA11	nfqisevepkYyadgedayam
IPI00455623	136	138	INSR+unsp->unsp			84779	NAA11	fqisevepkyYadgedayamk
IPI00456429	88	81		->PKA		7311	UBA52	lrlrggiiepSlrqlaqkync
IPI00456620	902	897	unsp+PKC+PKA+RSK->unsp+PKA+RSK	unsp+PKC+PKA+RSK->unsp+PKA+RSK		7862	BRPF1	tpahevgrrtSvlfskknpkt
IPI00456758	47	48	INSR+unsp->INSR	INSR+unsp->INSR		6157	RPL27A	lhhhrinfdkYhpgyfgkvgm
IPI00456758	55	52		->INSR	PhosphoSite	6157	RPL27A	rinfdkyhpgYfgkvgmkhyh
IPI00456758	110	106	->unsp+PKA	->unsp		6157	RPL27A	gaapiidvvrSgyykvlgkgk
IPI00456758	110	108	INSR+EGFR->INSR			6157	RPL27A	apiidvvrsgYykvlgkgklp
IPI00456758	110	109	INSR+unsp->unsp			6157	RPL27A	piidvvrsgyYkvlgkgklpk
IPI00456887	553	554		cdc2->PKC+cdc2		221092	HNRNPUL2	gqrrklllfkTfsrkvvvvvp
IPI00456969	371	362	PKC->PKC+cdc2			1778	DYNC1H1	kirqalvaifThlrkirntky
IPI00456969	371	370	unsp+PKC+PKG->unsp+PKC+PKA+DNAPK	unsp+PKC+PKG->unsp+PKC+DNAPK		1778	DYNC1H1	ifthlrkirnTkypiqralrl
IPI00456969	371	372	unsp->	unsp->INSR		1778	DYNC1H1	thlrkirntkYpiqralrlve
IPI00456969	394	385		cdc2->		1778	DYNC1H1	qralrlveaiSrdlssqllkv
IPI00456969	394	390	PKC->	PKC->		1778	DYNC1H1	lveaisrdlsSqllkvlgtrk
IPI00456969	754	745	->cdc2			1778	DYNC1H1	lkvnflpeiiTlskevrnlkw
IPI00456969	1125	1122	unsp->			1778	DYNC1H1	vqskvnlkydSwhkevlskfg
IPI00456969	1286	1281	unsp+cdc2+CKII->unsp			1778	DYNC1H1	lrpeealqalTiyegkfgrlk
IPI00456969	3480	3476	->PKA			1778	DYNC1H1	aaveakvnrsTallkslsaer
IPI00456969	3480	3481		unsp->		1778	DYNC1H1	kvnrstallkSlsaererwek
IPI00456969	4283	4280	unsp->PKA			1778	DYNC1H1	erlfttrsfdSefklackvdg
IPI00456970	556	558	unsp+PKC->PKC			8621	CDK13	vennlivdkaTkkavivgkes
IPI00464979	78	75	->EGFR	->EGFR		8803	SUCLA2	qeagvsvpkgYvakspdeaya
IPI00464979	78	79	unsp+GSK3+cdk5+CKII->unsp+cdc2+CKII	unsp+GSK3+cdk5+CKII->unsp+cdk5+cdc2+CKII		8803	SUCLA2	vsvpkgyvakSpdeayaiakk
IPI00465028	275	273	unsp->PKA	unsp->PKA		7167	TPI1	dvdgflvggaSlkpefvdiin
IPI00465044	92	91		unsp->unsp+DNAPK		55920	RCC2	aavvitepehTkervklegsk
IPI00465044	293	290	->PKA	->PKA		55920	RCC2	cpeygqlghnSdgkfiaraqr
IPI00465070	10	12	unsp+PKC+PKG->PKC	unsp+PKC+PKG->unsp+PKC	niprot|PHOsphoELM|PhosphoSi	8356	HIST1H3J	artkqtarksTggkaprkqla
IPI00465070	15	11		unsp->unsp+PKG	niprot|PHOsphoELM|PhosphoSi	8356	HIST1H3J	martkqtarkStggkaprkql
IPI00465070	15	12	unsp+PKC+PKG->PKG+PKA	unsp+PKC+PKG->PKC+PKG	niprot|PHOsphoELM|PhosphoSi	8356	HIST1H3J	artkqtarksTggkaprkqla
IPI00465070	24	23		PKC->PKC+DNAPK		8356	HIST1H3J	ggkaprkqlaTkaarksapat
IPI00465070	24	29	unsp+PKG+PKA->unsp+PKA		PHOsphoELM|PhosphoSite	8356	HIST1H3J	kqlatkaarkSapatggvkkp
IPI00465070	28	29	unsp+PKG+PKA->unsp+PKC+PKG+PKA	unsp+PKG+PKA->unsp+PKC+PKG+PKA	PHOsphoELM|PhosphoSite	8356	HIST1H3J	kqlatkaarkSapatggvkkp
IPI00465070	57	58	unsp->	unsp->		8356	HIST1H3J	alreirryqkStellirklpf
IPI00465070	116	108	->cdc2			8356	HIST1H3J	eaylvglfedTnlcaihakrv
IPI00465070	116	119	unsp+PKA->	unsp+PKA->		8356	HIST1H3J	nlcaihakrvTimpkdiqlar
IPI00465070	123	119	unsp+PKA->PKA	unsp+PKA->PKA		8356	HIST1H3J	nlcaihakrvTimpkdiqlar
IPI00465170	248	241	INSR+unsp->unsp	INSR+unsp->unsp		51559	NT5DC3	neyflknnidYepvhlykdvk
IPI00465170	248	247	unsp->			51559	NT5DC3	nnidyepvhlYkdvkdsirdv
IPI00465233	488	491	->cdc2	->cdc2		51386	EIF3L	epflqqlkvfSdevqqqaqls
IPI00465233	508	512	PKC->	PKC->PKC+cdc2		51386	EIF3L	tirsflklytTmpvaklagfl
IPI00465248	60	63	PKC->		Uniprot	2023	ENO1	dktrymgkgvSkavehinkti
IPI00465248	64	63	PKC->PKA	PKC->DNAPK	Uniprot	2023	ENO1	dktrymgkgvSkavehinkti
IPI00465248	193	189		unsp->unsp+EGFR	PhosphoSite	2023	ENO1	reamrigaevYhnlknvikek
IPI00465248	193	200		INSR+unsp->unsp		2023	ENO1	hnlknvikekYgkdatnvgde
IPI00465248	197	200	INSR+unsp->	INSR+unsp->		2023	ENO1	hnlknvikekYgkdatnvgde
IPI00465248	199	200	INSR+unsp->unsp			2023	ENO1	hnlknvikekYgkdatnvgde
IPI00465248	202	200	INSR+unsp->unsp	INSR+unsp->unsp		2023	ENO1	hnlknvikekYgkdatnvgde
IPI00465248	233	236	unsp->	unsp->		2023	ENO1	llktaigkagYtdkvvigmdv
IPI00465248	233	237	unsp+PKC->			2023	ENO1	lktaigkagyTdkvvigmdva
IPI00465248	256	254	unsp->	unsp->	Uniprot|PhosphoSite	2023	ENO1	mdvaaseffrSgkydldfksp
IPI00465248	281	282	->PKC	->PKC		2023	ENO1	spdqladlykSfikdypvvsi
IPI00465248	306	310	PKA->	PKA->		2023	ENO1	dwgawqkftaSagiqvvgddl
IPI00465248	326	321	PKC->p38MAPK	PKC->		2023	ENO1	agiqvvgddlTvtnpkriaka
IPI00465248	326	323	PKC->	PKC->		2023	ENO1	iqvvgddltvTnpkriakavn
IPI00465248	406	407	EGFR->			2023	ENO1	apcrserlakYnqllrieeel
IPI00465248	420	419		->DNAPK		2023	ENO1	qllrieeelgSkakfagrnfr

IPI00465256	34	25	PKA->cdc2+PKA			50808	AK3	apgsgkgtvsSritthfelkh
IPI00465256	34	29	unsp+RSK->unsp			50808	AK3	gkgtvssritThfelkhlssg
IPI00465256	34	37	unsp+PKA->unsp	unsp+PKA->unsp		50808	AK3	itthfelkhlSsgdllrdnml
IPI00465256	34	38	PKA->	PKA->		50808	AK3	tthfelkhlsSgdllrdnmlr
IPI00465275	405	403	PKC->	PKC->		10048	RANBP9	tdqtvleelaSiknrqriqkl
IPI00465294	626	621	unsp->INSR+unsp	unsp->INSR+unsp		988	CDC5L	ehitylehnpYekfskeelkk
IPI00465294	626	625	unsp+CKII->unsp+CKI+CKII	unsp+CKII->ATM+unsp+CKII+DNAPK		988	CDC5L	ylehnpyekfSkeelkkaqdv
IPI00465345	170	166	->unsp	->unsp		25766	PRPF40B	lsqcpwkeykSdtgkpyyynn
IPI00465345	170	168	unsp+PKC->	unsp+PKC->		25766	PRPF40B	qcpwkeyksdTgkpyyynnqs
IPI00465345	170	172	INSR->	INSR->		25766	PRPF40B	keyksdtgkpYyynnqskesr
IPI00465345	170	173	INSR+unsp->INSR			25766	PRPF40B	eyksdtgkpyYynnqskesrw
IPI00465361	174	170	unsp+PKG+CKII->unsp+PKG+PKA+CKII			6137	RPL13	vykkekarviTeeeknfkafa
IPI00465361	177	181	unsp+PKC->			6137	RPL13	eeeknfkafaSlrmaranarl
IPI00465363	7	5	unsp->	unsp->		255626	HIST1H2BA	------mpevSskgatiskkg
IPI00465363	7	6		unsp->ATM+unsp+DNAPK		255626	HIST1H2BA	-----mpevsSkgatiskkgf
IPI00465363	7	12	unsp+CKI+PKC->CKI+PKC	unsp+CKI+PKC->PKC		255626	HIST1H2BA	pevsskgatiSkkgfkkavvk
IPI00465363	13	12		unsp+CKI+PKC->ATM+unsp+CKI+PKC+DNAPK		255626	HIST1H2BA	pevsskgatiSkkgfkkavvk
IPI00465363	14	10	PKC->			255626	HIST1H2BA	-mpevsskgaTiskkgfkkav
IPI00465363	14	12	unsp+CKI+PKC->CKI+PKC	unsp+CKI+PKC->PKC		255626	HIST1H2BA	pevsskgatiSkkgfkkavvk
IPI00465363	17	12	unsp+CKI+PKC->unsp+PKC	unsp+CKI+PKC->PKC		255626	HIST1H2BA	pevsskgatiSkkgfkkavvk
IPI00465363	18	12	unsp+CKI+PKC->unsp+PKC	unsp+CKI+PKC->unsp+PKC		255626	HIST1H2BA	pevsskgatiSkkgfkkavvk
IPI00465363	25	23	unsp+PKC->	unsp+PKC->		255626	HIST1H2BA	kkgfkkavvkTqkkegkkrkr
IPI00465363	110	114	unsp->			255626	HIST1H2BA	lpgelakhavSegtkavtkyt
IPI00465363	122	121		->ATM+DNAPK		255626	HIST1H2BA	havsegtkavTkytssk----
IPI00465363	122	124		PKC->		255626	HIST1H2BA	segtkavtkyTssk-------
IPI00465373	108	105	cdc2->PKA+cdc2	cdc2->PKA		56267	CCBL2	nqytrgfghpSlvkalsylye
IPI00465373	116	111	PKC->			56267	CCBL2	fghpslvkalSylyeklyqkq
IPI00465373	116	118		->EGFR		56267	CCBL2	kalsylyeklYqkqidsnkei
IPI00465428	3538	3537		->DNAPK		54832	VPS13C	gvvggvtgiiTkpvegakkeg
IPI00465436	237	236	unsp->	unsp->INSR+unsp		847	CAT	ngeavyckfhYktdqgiknls
IPI00465439	13	9		unsp+GSK3+p38MAPK->unsp+p38MAPK	Uniprot|PhosphoSite	226	ALDOA	--mpyqypalTpeqkkelsdi
IPI00465439	42	39	PKC->		Uniprot|PhosphoSite	226	ALDOA	gilaadestgSiakrlqsigt
IPI00465439	42	46	PKA+RSK->PKA		Uniprot|PHOsphoELM|PhosphoSi	226	ALDOA	stgsiakrlqSigtenteenr
IPI00470502	53	56		->PKC		27068	PPA2	qnyrlffknvTghyispfhdi
IPI00470502	231	230		->DNAPK		27068	PPA2	iainandpeaSkfhdiddvkk
IPI00470502	291	286	PKC->			27068	PPA2	kafaleviksThqcwkallmk
IPI00470528	83	80	PKC->			6138	RPL15	grkrpvpkgaTygkpvhhgvn
IPI00470528	153	152		PKC->ATM+PKC+DNAPK		6138	RPL15	irrnpdtqwiTkpvhkhremr
IPI00470573	327	320	CKII->cdc2+CKII			10097	ACTR2	ggstmypglpSrlerelkqly
IPI00470883	607	601		CKII->		10735	STAG2	lpqyfdleiyTtgrlekhlda
IPI00470883	607	602	unsp+PKC->unsp			10735	STAG2	pqyfdleiytTgrlekhldal
IPI00470891	81	79	unsp+PKC+PKG->	unsp+PKC+PKG->		7812	CSDE1	vefevssdrrTgkpiavklvk
IPI00470891	434	431	PKC->CKII			7812	CSDE1	hshsdhrflgTvekeatfsnp
IPI00472160	447	440	unsp+PKC+PKA->unsp+PKC+cdc2+PKA			9181	ARHGEF2	ktysefcsrhSkalklykely
IPI00472160	447	450	unsp->			9181	ARHGEF2	skalklykelYardkrfqqfi
IPI00472724	172	175	unsp->	unsp->		158078	EEF1AL3	kryeeivkevStyikkigynp
IPI00472724	179	177	EGFR->			158078	EEF1AL3	yeeivkevstYikkigynpdt
IPI00472724	318	316	unsp+PKC->unsp	unsp+PKC->unsp		158078	EEF1AL3	dnvgfkvknvSvkdvrrgnva
IPI00472782	435	434	unsp+CKI->unsp+CKI+DNAPK	unsp+CKI->unsp+CKI+DNAPK		9678	PHF14	rpvtltemnySkygakecsfc
IPI00472782	435	436	->SRC			9678	PHF14	vtltemnyskYgakecsfced
IPI00472939	169	170	INSR+unsp->unsp			9789	SPCS2	ssslkrfddkYtlkltfisgr
IPI00472939	169	171	unsp+PKC->PKC	unsp+PKC->unsp		9789	SPCS2	sslkrfddkyTlkltfisgrt
IPI00472939	191	190		CKI->DNAPK		9789	SPCS2	rtkqqreaefTksiakffdhs
IPI00473014	114	113	->PKA	->ATM		11034	DSTN	lwapelaplkSkmiyasskda
IPI00473014	114	117	unsp->		PhosphoSite	11034	DSTN	elaplkskmiYasskdaikkk
IPI00473047	273	272		->DNAPK		5571	PRKAG1	ktynnldvsvTkalqhrshyf
IPI00473136	866	864	unsp+PKC->PKC	unsp+PKC->PKC		1495	CTNNA1	vqtvkasyvaStkyqksqgma
IPI00473136	866	865		unsp+PKC->unsp+PKC+DNAPK		1495	CTNNA1	qtvkasyvasTkyqksqgmas
IPI00473136	866	867	unsp+EGFR->unsp			1495	CTNNA1	vkasyvastkYqksqgmasln
IPI00473136	866	870	unsp+DNAPK->DNAPK			1495	CTNNA1	syvastkyqkSqgmaslnlpa
IPI00473136	866	875	->cdc2	->cdc2		1495	CTNNA1	tkyqksqgmaSlnlpavswkm
IPI00477040	38	30	unsp+PKG+CKII+RSK+DNAPK->unsp+PKG+cdc2+CKII+RSK+DNAPK			23511	NUP188	llgrsalrelSqieaelnkhw
IPI00477259	93	98		PKC->		54511	HMGCLL1	glqnekvivpTdikiefinrl
IPI00477313	39	38	->CKI+DNAPK	->ATM+DNAPK		3183	HNRNPC	vkksdveaifSkygkivgcsv
IPI00477313	50	47	PKC->PKA	PKC->PKC+PKA		3183	HNRNPC	fskygkivgcSvhkgfafvqy
IPI00477313	170	162	unsp->unsp+PKA	unsp->unsp+PKA	Uniprot|PHOsphoELM	3183	HNRNPC	avvpskrqrvSgntsrrgksg
IPI00477313	170	166	unsp+PKC->unsp	unsp+PKC->unsp		3183	HNRNPC	skrqrvsgntSrrgksgfnsk
IPI00477313	170	175	unsp+cdc2->cdc2			3183	HNRNPC	tsrrgksgfnSksgqrgssks
IPI00477495	6	7		unsp->		54145	H2BFS	----mpepakSapapkkgskk
IPI00477495	12	7	unsp->	unsp->		54145	H2BFS	----mpepakSapapkkgskk
IPI00477495	12	15	unsp+PKC->PKC	unsp+PKC->PKC	Uniprot	54145	H2BFS	aksapapkkgSkkavtkaqkk
IPI00477495	13	15	unsp+PKC->PKC		Uniprot	54145	H2BFS	aksapapkkgSkkavtkaqkk
IPI00477495	16	15	unsp+PKC->unsp+PKC+PKA	unsp+PKC->ATM+unsp+PKC+DNAPK	Uniprot	54145	H2BFS	aksapapkkgSkkavtkaqkk
IPI00477495	21	15	unsp+PKC->unsp+PKC+PKA	unsp+PKC->unsp+PKC+PKA	Uniprot	54145	H2BFS	aksapapkkgSkkavtkaqkk
IPI00477495	21	20		PKC->PKC+DNAPK		54145	H2BFS	apkkgskkavTkaqkkdgrkr
IPI00477495	109	113	unsp->			54145	H2BFS	lpgelakhavSegtkavtkyt
IPI00477505	106	105		PKA->ATM+PKA		23243	ANKRD28	lsgarvnakdSkwltplhrav
IPI00477505	106	109	cdk5+p38MAPK->cdk5			23243	ANKRD28	rvnakdskwlTplhravascs
IPI00477686	22	26	unsp+EGFR->	unsp+EGFR->EGFR		2963	GTF2F2	tgvwlvkvpkYlsqwakasg
IPI00477686	33	28	ATM+cdc2+DNAPK->ATM+DNAPK	ATM+cdc2+DNAPK->cdc2+DNAPK		2963	GTF2F2	vwlvkvpkylSqqwakasgrg
IPI00477686	33	35		PKC->		2963	GTF2F2	kylsqqwakaSgrgevgklri
IPI00477686	137	135	unsp->	unsp->		2963	GTF2F2	mrlkrlqieeSskpvrlsqql
IPI00477686	137	136	cdc2->cdc2+DNAPK	cdc2->cdc2+DNAPK		2963	GTF2F2	rlkrlqieesSkpvrlsqqld
IPI00477686	137	142	ATM+unsp+PKA+DNAPK->ATM+DNAPK	ATM+unsp+PKA+DNAPK->ATM+PKA+DNAPK	Uniprot|PhosphoSite	2963	GTF2F2	ieesskpvrlSqqldkvvttn
IPI00477923	636	627	unsp+GSK3+cdk5+p38MAPK->unsp+GSK3+cdk5+cdc2+p38MAPK		Uniprot|PHOsphoELM|PhosphoSi	1822	ATN1	asspagyktaSppgppygkr
IPI00477923	636	634	EGFR->			1822	ATN1	ktasppgpppYgkrapsgay
IPI00477923	636	640	unsp+GSK3+cdk5+PKA->unsp+GSK3+cdk5	unsp+GSK3+cdk5+PKA->unsp+GSK3+cdk5+cdc2	niprot|PHOsphoELM|PhosphoSi	1822	ATN1	gpppygkrapSgayktatpp
IPI00477923	786	785		PKC+cdc2->cdc2+DNAPK		1822	ATN1	sdlyfvplegSklakkradlv
IPI00478410	55	47	unsp+PKC->unsp+PKC+PKA	unsp+PKC->unsp+PKC+PKA		509	ATP5C1	sikniqkitkSmkmvaaakya
IPI00478410	55	56	EGFR->			509	ATP5C1	ksmkmvaaakYaraerelkpa
IPI00478410	79	73	->PKA	->PKA		509	ATP5C1	lkpariyglgSlalyekadik
IPI00478410	115	109		->cdc2		509	ATP5C1	drglcgaihsSiakqmkseva
IPI00478410	115	116	unsp->	unsp->		509	ATP5C1	ihssiakqmkSevatltaagk
IPI00478410	115	120	PKC->	PKC->		509	ATP5C1	iakqmksevaTltaagkevml
IPI00478410	197	192	unsp+PKC+PKA->PKC+PKA	unsp+PKC+PKA->PKC+PKA		509	ATP5C1	egsiifnkfrSvisykteekp
IPI00478410	197	195	unsp+PKC->CKII	unsp+PKC->CKII		509	ATP5C1	iifnkfrsviSykteekpifs
IPI00478410	197	198		->unsp		509	ATP5C1	nkfrsvisykTeekpifslnt
IPI00478410	197	205	PKA+DNAPK->DNAPK	PKA+DNAPK->DNAPK		509	ATP5C1	sykteekpifSlntvasadsm
IPI00478718	28	33	unsp+PKC->	unsp+PKC->		164395	TTLL9	qnqnykghglSkgkereqras
IPI00478772	17	15	EGFR->			56905	C15orf39	rplrtlgpvmYgklprletds
IPI00479125	227	223	unsp+PKC+PKG+PKA->unsp+PKG+PKA			23380	SRGAP2	vrieekhvrrSsvkkiekmke
IPI00479145	97	93	unsp+PKC+RSK->unsp+PKA+RSK	unsp+PKC+RSK->unsp+RSK		3880	KRT19	tmqnlndrlaSyldkvralea
IPI00479186	62	60	unsp+CKI+PKC->CKI+CKII	unsp+CKI+PKC->CKII		5315	PKM2	tigpasrsveTlkemiksgmn
IPI00479186	62	67	PKA->	PKA->		5315	PKM2	svetlkemikSgmnvarlnfs
IPI00479186	89	87	unsp->	unsp->		5315	PKM2	shgtheyhaeTiknvrtates
IPI00479186	135	127	CKII+PKA->cdc2+CKII+PKA			5315	PKM2	peirtglikgSgtaevelkkg
IPI00479186	135	139	unsp+PKC+PKA->PKC	unsp+PKC+PKA->PKC		5315	PKM2	taevelkkgaTlkitldnaym
IPI00479186	266	269	unsp+PKC+CKII->PKC+CKII	unsp+PKC+CKII->PKC+CKII		5315	PKM2	gekgknikiiSkienhegvrr
IPI00479186	433	425	->PKA	->PKA		5315	PKM2	gaveasfkccSgaiivltksg
IPI00479186	433	432		PKC->PKC+DNAPK		5315	PKM2	kccsgaiivlTksgrsahqva
IPI00479186	433	437	unsp+PKA->	unsp+PKA->unsp		5315	PKM2	aiivltksgrSahqvaryrpr
IPI00479262	1096	1093	unsp->unsp+PKA	unsp->unsp+PKA	niprot|PHOsphoELM|PhosphoSi	1981	EIF4G1	nqlfapggrlSwgkgssggsg
IPI00479262	1096	1098	->PKC			1981	EIF4G1	pggrlswgkgSsggsgakpsd
IPI00479262	1096	1099	->cdc2	->cdc2		1981	EIF4G1	ggrlswgkgsSggsgakpsda
IPI00479262	1189	1188	unsp->unsp+PKA	unsp->ATM+unsp+DNAPK	niprot|PHOsphoELM|PhosphoSi	1981	EIF4G1	artpatkrsfSkeveersrer
IPI00479385	583	580	->CKII			8623	ASMTL	lmqslnmlvqTegkerslgey
IPI00479385	583	586	unsp+CKI+PKA->CKI+PKA	unsp+CKI+PKA->CKI+PKA		8623	ASMTL	mlvqtegkerSlgeyqcllel
IPI00479545	470	461	->cdc2			55818	KDM3A	napevkagvnSdspnncsgkk
IPI00479545	470	468	PKC+cdc2->cdc2	PKC+cdc2->cdc2		55818	KDM3A	gvnsdspnncSgkkvepsala
IPI00479545	895	891	CKI+PKC->CKI	CKI+PKC->unsp+CKI		55818	KDM3A	nsgflrnllnSstgktenglk
IPI00479545	895	892	unsp->			55818	KDM3A	sgflrnllnsStgktenglkn
IPI00479571	33	34		unsp+cdc2->unsp+PKC+cdc2		84062	DTNBP1	sdksreakvkSkprtvpflpk
IPI00479571	33	38	PKC->	PKC->GSK3		84062	DTNBP1	reakvkskprTvpflpkysag
IPI00479571	35	34	unsp+cdc2->unsp+PKA+cdc2	unsp+cdc2->unsp+cdc2+DNAPK		84062	DTNBP1	sdksreakvkSkprtvpflpk
IPI00479571	35	38	PKC->GSK3			84062	DTNBP1	reakvkskprTvpflpkysag
IPI00479997	9	4	CKI+PKC+cdc2->	CKI+PKC+cdc2->PKC+cdc2		3925	STMN1	-------masSdiqvkelekr
IPI00479997	95	94		CKI+CKII->unsp+CKII+DNAPK		3925	STMN1	qkaieennnfSkmaeeklthk
IPI00479997	100	94	CKI+CKII->CKII	CKI+CKII->CKII		3925	STMN1	qkaieennnfSkmaeeklthk

IPI00513768	320	317	unsp->	unsp->		219293	ATAD3C	arlvrmylneYvlkpategkr
IPI00513791	1962	1965	PKA->	PKA->		85440	DOCK7	gelheqfkrkTilttshafpy
IPI00513827	334	333		->DNAPK		34	ACADM	glaqraldeaTkyalerktfg
IPI00513827	334	335	unsp->	unsp->		34	ACADM	aqraldeatkYalerktfgkl
IPI00514053	309	306	unsp+cdc2->PKA+cdc2	unsp+cdc2->unsp+PKA+cdc2		372	ARCN1	elhgmimlriSddkygrirlh
IPI00514053	309	310	INSR+unsp->SRC+unsp	INSR+unsp->SRC+INSR+unsp		372	ARCN1	mimlrisddkYgrirlhvene
IPI00514648	604	602	unsp->	unsp->		6595	SMARCA2	dlpvkvthteTgkvlfgpeap
IPI00514648	994	992	unsp+PKC->unsp	unsp+PKC->unsp		6595	SMARCA2	qakgilltdgSekdkkgkgga
IPI00514648	996	992	unsp+PKC->unsp			6595	SMARCA2	qakgilltdgSekdkkgkgga
IPI00514648	996	1004	unsp+PKA->unsp	unsp+PKA->unsp		6595	SMARCA2	kdkkgkggakTlmntimqlrk
IPI00514648	997	992	unsp+PKC->unsp			6595	SMARCA2	qakgilltdgSekdkkgkgga
IPI00514648	999	1004	unsp+PKA->unsp	unsp+PKA->unsp		6595	SMARCA2	kdkkgkggakTlmntimqlrk
IPI00514983	274	280	unsp->unsp+PKA			10808	HSPH1	qeceklkklmSsnstdlplni
IPI00514983	274	281	PKA->cdc2+PKA	PKA->cdc2+PKA		10808	HSPH1	eceklkklmsSnstdlplnie
IPI00514983	274	283		->cdc2		10808	HSPH1	eklkklmssnStdlplniecf
IPI00515061	6	7		unsp->		8970	HIST1H2BJ	----mpepakSapapkkgskk
IPI00515061	12	7	unsp->	unsp->		8970	HIST1H2BJ	----mpepakSapapkkgskk
IPI00515061	12	15	unsp+PKC->PKC	unsp+PKC->PKC	Uniprot	8970	HIST1H2BJ	aksapapkkgSkkavtkaqkk
IPI00515061	13	15	unsp+PKC->PKC		Uniprot	8970	HIST1H2BJ	aksapapkkgSkkavtkaqkk
IPI00549171	33	24		ATM+unsp+cdc2+DNAPK->ATM+unsp+DNAPK		29803	REPIN1	laqprllsgpSqespqtlgke
IPI00549171	33	30	unsp+PKC->unsp	unsp+PKC->unsp		29803	REPIN1	lsgpsqespqTlgkesrglrq
IPI00549171	33	35	PKA->	PKA->		29803	REPIN1	qespqtlgkeSrglrqqgtsv
IPI00549171	276	274	unsp+PKC+PKG+PKA->cdc2+PKA	unsp+PKC+PKG+PKA->PKA		29803	REPIN1	hqcpecgkrfTnkpyltshrr
IPI00549171	276	280	unsp+PKC->	unsp+PKC->PKC		29803	REPIN1	gkrftnkpylTshrrihtgek
IPI00549189	538	529	PKA->cdc2+PKA			7064	THOP1	lrmsrhyrtgSavprellekl
IPI00549189	538	542		->cdc2		7064	THOP1	prelleklieSrqantglfnl
IPI00549205	270	269	unsp->	unsp->ATM+unsp+DNAPK		10111	RAD50	nrlkeiehnlSkimkldneik
IPI00549232	384	383	PKC->	PKC->DNAPK		22862	FNDC3A	ipnpprianrTknsltlqwka
IPI00549232	384	386	PKA->CKI+PKA	PKA->CKI+PKA		22862	FNDC3A	pprianrtknSltlqwkapsd
IPI00549248	27	29	->unsp	->unsp	PhosphoSite	4869	NPM1	fgcelkadkdYhfkvdndene
IPI00549248	141	137	unsp+PKC->unsp	unsp+PKC->unsp	Uniprot|PhosphoSite	4869	NPM1	deeeedvkllSisgkrsapgg
IPI00549248	141	139	unsp->cdc2	unsp->	Uniprot|PhosphoSite	4869	NPM1	eeedvkllsiSgkrsapgggs
IPI00549248	141	143	unsp+PKG->unsp	unsp+PKG->unsp		4869	NPM1	vkllsisgkrSapgggskvpq
IPI00549248	150	149	PKC+cdc2->cdc2	PKC+cdc2->cdc2+DNAPK		4869	NPM1	sgkrsapgggSkvpqkkvkla
IPI00549248	154	149	PKC+cdc2->	PKC+cdc2->cdc2		4869	NPM1	sgkrsapgggSkvpqkkvkla
IPI00549248	212	207		unsp->		4869	NPM1	rdtpaknaqkSnqngkdskps
IPI00549248	212	214	unsp->PKC			4869	NPM1	aqksnqngkdSkpsstprskg
IPI00549248	212	217	unsp+PKG->	unsp+PKG->PKG		4869	NPM1	snqngkdskpSstprskgqes
IPI00549248	215	214	unsp->PKA	unsp->unsp+DNAPK		4869	NPM1	aqksnqngkdSkpsstprskg
IPI00549248	215	217	unsp+PKG->PKC	unsp+PKG->unsp		4869	NPM1	snqngkdskpSstprskgqes
IPI00549248	215	219	unsp+PKC+cdk5+p38MAPK->unsp+cdk5+p38MAPK		PHOsphoELM|PhosphoSite	4869	NPM1	qngkdskpssTprskgqesfk
IPI00549248	229	227	unsp+PKC->unsp	unsp+PKC->unsp	PHOsphoELM	4869	NPM1	sstprskgqeSfkkqektpkt
IPI00549248	229	234		unsp->unsp+p38MAPK	ıiprot|PHOsphoELM|PhosphoSi	4869	NPM1	gqesfkkqekTpktpkgpssv
IPI00549248	230	227	unsp+PKC->unsp		PHOsphoELM	4869	NPM1	sstprskgqeSfkkqektpkt
IPI00549248	230	234		unsp->unsp+p38MAPK	ıiprot|PHOsphoELM|PhosphoSi	4869	NPM1	gqesfkkqekTpktpkgpssv
IPI00549248	257	260	->cdc2	->cdc2	Uniprot|PhosphoSite	4869	NPM1	kmqasiekggSlpkveakfin
IPI00549248	267	260	->cdc2	->PKA	Uniprot|PhosphoSite	4869	NPM1	kmqasiekggSlpkveakfin
IPI00549248	273	271		->unsp		4869	NPM1	lpkveakfinYvkncfrmtdq
IPI00549248	292	293	PKA+RSK->PKC+PKA	PKA+RSK->PKC+PKA		4869	NPM1	aiqdlwqwrkSl---------
IPI00549357	272	276	unsp+PKA+DNAPK->PKA+DNAPK	unsp+PKA+DNAPK->unsp+DNAPK		55572	FOXRED1	ihevhvkmdrSleyqpvecai
IPI00549664	889	893	unsp+PKC->unsp			54881	TEX10	lvqqiiknitTlksgsvqeqw
IPI00549664	889	898		unsp+PKA->unsp		54881	TEX10	iknittlksgSvqeqwltdlh
IPI00549672	298	299		unsp->unsp+PKC		5719	PSMD13	rqltfeeiakSakitvnevel
IPI00549725	106	103	PKC->			5223	PGAM1	ggltglnkaeTaakhgeaqvk
IPI00549725	113	118	unsp+PKC+PKG+PKA->unsp+PKC+PKA		Uniprot|PHOsphoELM|PhosphoSi	5223	PGAM1	geaqvkiwrrSydvppppmep
IPI00549730	206	203	PKC->	PKC->		51074	APIP	rrhgvyvwgeTwekaktmcec
IPI00549730	206	209	unsp+CKII->CKII			51074	APIP	vwgetwekakTmcecydylfd
IPI00549730	208	203	PKC->	PKC->		51074	APIP	rrhgvyvwgeTwekaktmcec
IPI00549955	377	378		PKC->unsp+PKC		55329	MNS1	akeeeenfrkTmlakfaeddr
IPI00550020	4	5	unsp->unsp+CKI			5763	PTMS	------msekSveaaaelsak
IPI00550020	92	96	unsp+PKG+RSK->unsp+PKG	unsp+PKG+RSK->unsp+RSK		5763	PTMS	edeadpkrqkTengasa----
IPI00550021	294	291		->EGFR		6122	RPL3	nkkiykigqgYlikdgklikn
IPI00550021	373	370	unsp+PKC->unsp			6122	RPL3	lekidlkfidTtskfghgrfq
IPI00550021	373	371	PKC->	PKC->		6122	RPL3	ekidlkfidtTskfghgrfqt
IPI00550021	373	372		PKC->ATM+PKC+DNAPK		6122	RPL3	kidlkfidttSkfghgrfqtm
IPI00550243	26	22		->EGFR		55623	THUMPD1	gggkrkgkaqYvlakrarrcd
IPI00550364	492	489	unsp+PKC->unsp			55276	PGM2	asyfichdqeTikklfenlrn
IPI00550451	147	144	->EGFR	->EGFR		5499	PPP1CA	ygfydeckrrYniklwktftd
IPI00550655	7	3	unsp->unsp+cdk5	unsp->unsp+cdk5		10927	SPIN1	--------mkTpfgktpgqrs
IPI00550655	7	8	unsp+cdk5->unsp+cdk5+p38MAPK	unsp+cdk5->unsp+cdk5+p38MAPK		10927	SPIN1	---mktpfgkTpgqrsradag
IPI00550689	496	491	unsp+PKC->PKC	unsp+PKC->PKC		51493	C22orf28	vvntchdagiSkkaiklrpia
IPI00550703	750	749	unsp->unsp+PKA	unsp->ATM+unsp+DNAPK		54872	PIGG	gsvrfpwrpdSkdiskgiiea
IPI00550703	750	753	unsp->cdc2	unsp->cdc2		54872	PIGG	fpwrpdskdiSkgiiearfvy
IPI00550703	754	749	unsp->unsp+PKA	unsp->unsp+PKA		54872	PIGG	gsvrfpwrpdSkdiskgiiea
IPI00550703	754	753	unsp->unsp+PKA	unsp->ATM+unsp+DNAPK		54872	PIGG	fpwrpdskdiSkgiiearfvy
IPI00550746	239	232	unsp+CKII->unsp+cdc2+CKII			10726	NUDC	lynevkveesSwliedgkvvt
IPI00550746	239	242	PKC->PKC+CKII	PKC->PKC+CKII		10726	NUDC	swliedgkvvTvhlekinkme
IPI00550900	93	98	unsp+PKC->PKC	unsp+PKC->PKC		7178	TPT1	ykkyikdymkSikgkleeqrp
IPI00550917	19	24	unsp+PKC+PKA+RSK->unsp+PKC+RSK			11344	TWF2	keffakaragSvrlikvvied
IPI00550991	260	259		unsp+PKC->ATM+unsp+PKC+DNAPK		12	SERPINA3	qdthqsrfylSkkkwvmvpmm
IPI00550991	261	259	unsp+PKC->PKC	unsp+PKC->PKC		12	SERPINA3	qdthqsrfylSkkkwvmvpmm
IPI00550991	441	440		->DNAPK		12	SERPINA3	ptdtqniffmSkvtnpkqa--
IPI00550991	441	443	->PKC			12	SERPINA3	tqniffmskvTnpkqa-----
IPI00552413	161	158	unsp->PKA			1025	CDK9	gevfkarhrkTgqkvalkkvl
IPI00552440	126	132	DNAPK->cdc2+DNAPK	DNAPK->cdc2+DNAPK		51426	POLK	elkdkpiavgSmsmlstsnyh
IPI00552701	797	791	unsp+PKC->unsp+PKC+PKA	unsp+PKC->unsp+PKC+PKA		1741	DLG3	ealmemnrrqTyeqankiydk
IPI00552873	6	2	PKC->		Uniprot	8336	HIST1H2AM	---------mSgrgkqggkar
IPI00552897	812	809	unsp+PKC->unsp+PKC+PKA	unsp+PKC->unsp+PKC+PKA		9656	MDC1	pmgiqgrgrqTvdkvmgipke
IPI00552897	1402	1399	unsp+PKC+PKA->unsp+PKA	unsp+PKC+PKA->unsp+PKA	Uniprot|PhosphoSite	9656	MDC1	sratrgrknrSsgktpetlvp
IPI00552897	1402	1400	unsp+PKC+PKA->unsp+cdc2+PKA	unsp+PKC+PKA->unsp+PKA	Uniprot|PhosphoSite	9656	MDC1	ratrgrknrsSgktpetlvpt
IPI00552897	1402	1403	GSK3+cdk5->GSK3+cdk5+p38MAPK	GSK3+cdk5->unsp+cdk5+p38MAPK	Uniprot|PhosphoSite	9656	MDC1	rgrknrssgkTpetlvptapk
IPI00552978	607	601		CKII->		10735	STAG2	lpqyfdleiyTtgrlekhlda
IPI00552978	607	602	unsp+PKC->unsp			10735	STAG2	pqyfdleiytTgrlekhldal
IPI00553185	248	243		unsp+CKII->CKII		7203	CCT3	iknprivlldSsleykkgesq
IPI00553185	248	244		DNAPK->unsp+DNAPK	Uniprot|PhosphoSite	7203	CCT3	knprivlldsSleykkgesqt
IPI00553185	248	247	unsp->			7203	CCT3	rivlldssleYkkgesqtdie
IPI00554436	605	601	cdc2+DNAPK->DNAPK	cdc2+DNAPK->unsp+DNAPK		10147	SFRS14	dqlvkrviegSlspkertllk
IPI00554560	442	437	PKC+cdc2->cdc2			400506	C16orf88	kysrgaglgfStapnkifyid
IPI00554560	442	445	unsp->	unsp->		400506	C16orf88	gfstapnkifYidrnasksvk
IPI00554648	11	13	unsp->unsp+PKC		Uniprot	3856	KRT8	irvtqksykvStsgprafssr
IPI00554648	11	14	unsp->		Uniprot	3856	KRT8	rvtqksykvsTsgprafssrs
IPI00554648	101	104	unsp+PKC->unsp			3856	KRT8	qiktlnnkfaSfidkvrfleq
IPI00554648	117	121	PKC->			3856	KRT8	fleqqnkmleTkwsllqqqkt
IPI00554648	122	121	PKC->	PKC->		3856	KRT8	fleqqnkmleTkwsllqqqkt
IPI00554648	122	124	PKA->	PKA->		3856	KRT8	qqnkmletkwSllqqqktars
IPI00554648	472	475	unsp->	unsp->	ıiprot|PHOsphoELM|PhosphoSi	3856	KRT8	kietrdgklvSessdvlpk--
IPI00554652	455	451	PKC->	PKC->		4046	LSP1	psgkrykfvaTghgkyekvlv
IPI00554652	455	456	unsp->	unsp->		4046	LSP1	ykfvatghgkYekvlveggpa
IPI00554681	30	29	PKC->PKA	PKC->		4698	NDUFA5	tpherlrilyTkildvleeip
IPI00554681	46	48		->unsp		4698	NDUFA5	ipknaayrkyTeqitneklam
IPI00554723	121	125	PKC->			6134	RPL10	mrgafgkpqgTvarvhigqvi
IPI00554761	3	7	unsp+PKG+PKA+RSK->unsp+PKG+PKA		Uniprot|PhosphoSite	643790	LOC643790	----mpkrkvSsaegaakeep
IPI00554761	5	7		unsp+PKG+PKA+RSK->unsp+PKG+PKA	Uniprot|PhosphoSite	643790	LOC643790	----mpkrkvSsaegaakeep
IPI00554761	27	25	unsp+PKC+PKG->unsp+PKG	unsp+PKC+PKG->unsp	ıiprot|PHOsphoELM|PhosphoSi	643790	LOC643790	eepkrrsarlSakppakveak
IPI00554761	42	45	unsp->	unsp->PKC		643790	LOC643790	kpkkaaakdkSsdkkvqtkgk
IPI00554761	48	45	unsp->			643790	LOC643790	kpkkaaakdkSsdkkvqtkgk
IPI00554761	48	46	unsp+PKC->PKC	unsp+PKC->PKC		643790	LOC643790	pkkaaakdksSdkkvqtkgkr
IPI00554761	48	52	unsp+PKC->PKC			643790	LOC643790	kdkssdkkvqTkgkrgakgkq
IPI00554761	53	52		unsp+PKC->unsp+PKC+DNAPK		643790	LOC643790	kdkssdkkvqTkgkrgakgkq
IPI00554761	55	52	unsp+PKC->PKC			643790	LOC643790	kdkssdkkvqTkgkrgakgkq
IPI00554761	82	81	unsp+CKII->CKII	unsp+CKII->CKII		643790	LOC643790	kedlpaengeTkteespasde
IPI00554761	82	83		->unsp	PhosphoSite	643790	LOC643790	dlpaengetkTeespasdeag
IPI00554777	385	390	unsp+CKII->CKII	unsp+CKII->CKII		440	ASNS	apspekaeeeSerllrelylf
IPI00554788	81	86	unsp+cdc2+PKA->cdc2+PKA	unsp+cdc2+PKA->cdc2+PKA		3875	KRT18	giqneketmqSlndrlasyld
IPI00554788	111	110	unsp+PKC+PKA->unsp+PKC+PKA+RSK	unsp+PKC+PKA->unsp+PKC+PKA+RSK		3875	KRT18	sletenrrleSkirehlekkg
IPI00554788	131	127	unsp+PKA->unsp+PKA+RSK	unsp+PKA->unsp+PKG+PKA+RSK		3875	KRT18	ekkgpqvrdwShyfkiiedlr
IPI00554788	167	168	INSR->			3875	KRT18	rlaaddfrvkYetelamrqsv
IPI00554788	167	170	->CKII	->CKII		3875	KRT18	aaddfrvkyeTelamrqsven

IPI00554788	426	420		unsp+CKII->unsp	Uniprot	3875	KRT18	trrivdgkvvSetndtkvlrh
IPI00554798	12	15	unsp+PKC->PKC	unsp+PKC->PKC	Uniprot	8342	HIST1H2BM	vksapvpkkgSkkainkaqkk
IPI00554798	13	15	unsp+PKC->PKC	unsp+PKC->PKC	Uniprot	8342	HIST1H2BM	vksapvpkkgSkkainkaqkk
IPI00554798	16	15	unsp+PKC->PKC	unsp+PKC->ATM+unsp+PKC+DNAPK	Uniprot	8342	HIST1H2BM	vksapvpkkgSkkainkaqkk
IPI00554798	17	15	unsp+PKC->PKC	unsp+PKC->PKC	Uniprot	8342	HIST1H2BM	vksapvpkkgSkkainkaqkk
IPI00554798	109	113	unsp->			8342	HIST1H2BM	lpgelakhavSegtkavtkyt
IPI00554798	121	120		->ATM+DNAPK		8342	HIST1H2BM	havsegtkavTkytssk----
IPI00554798	121	123		PKC->		8342	HIST1H2BM	segtkavtkyTssk-------
IPI00554811	35	27	ATM+DNAPK->DNAPK			26140	TTLL3	qaalclenfsSqvverhnkpe
IPI00555744	71	67		->unsp		9045	RPL14	ltdfilkfphSahqkyvrqaw
IPI00555744	71	72	unsp->	unsp->		9045	RPL14	lkfphsahqkYvrqawqkadi
IPI00555744	85	84		PKC->DNAPK		9045	RPL14	rqawqkadinTkwaatrwakk
IPI00555876	266	274	unsp+PKA->unsp	unsp+PKA->unsp		730211	HSP90AA5P	lekkvkkvvvSncmedpqrht
IPI00555902	41	40	->PKA			132299	OCIAD2	sklhihraeiSkimrecqees
IPI00556369	173	168	unsp+PKC->unsp+PKC+PKA			23049	SMG1	reddrdrrlaTvkqlkefiqq
IPI00556553	252	244	->cdc2			22	ABCB7	lhnldlgfhlSrqtgalskai
IPI00556553	252	247	PKC->PKA			22	ABCB7	ldlgfhlsrqTgalskaidrg
IPI00556553	252	251		->ATM+DNAPK		22	ABCB7	fhlsrqtgalSkaidrgtrgi
IPI00604400	324	321	unsp->		Uniprot	23174	ZCCHC14	hkyypvfkqlSmekflsltee
IPI00604400	324	329		CKI+CKII->CKII		23174	ZCCHC14	qlsmekflslTeedlnkfesl
IPI00604590	37	32	PKC->			4831	NME2	dtgtmancerTfiaikpdgvq
IPI00604590	125	124		unsp+CKI->unsp+DNAPK		4831	NME2	vmlgetnpadSkpgtirgdfc
IPI00604590	189	184	unsp+PKA+RSK->unsp+PKA	unsp+PKA+RSK->unsp+PKA		4831	NME2	rlvamkflraSeehlkqhyid
IPI00604590	189	192	unsp->	unsp->		4831	NME2	raseehlkqhYidlkdrpffp
IPI00604590	264	260	PKC+cdc2->PKA	PKC+cdc2->		4831	NME2	iqvgrniihgSdsvksaekei
IPI00604590	264	262	unsp+PKC+cdc2->unsp+cdc2	unsp+PKC+cdc2->unsp+cdc2		4831	NME2	vgrniihgsdSvksaekeisl
IPI00604590	268	260		PKC+cdc2->PKC		4831	NME2	iqvgrniihgSdsvksaekei
IPI00604620	79	76	unsp+cdk5+p38MAPK->unsp+p38MAPK	unsp+cdk5+p38MAPK->unsp+p38MAPK	niprot|PHOsphoELM|PhosphoSi	4691	NCL	vsptkkvavaTpakkaavtpg
IPI00604620	79	84		unsp->unsp+p38MAPK		4691	NCL	vatpakkaavTpgkkaaatpa
IPI00604620	102	97	unsp+PKC->unsp+PKC+PKG			4691	NCL	kkaaatpakkTvtpakavttp
IPI00604620	102	99	unsp->	unsp->	niprot|PHOsphoELM|PhosphoSi	4691	NCL	aaatpakktvTpakavttpgk
IPI00604620	102	105	PKC->		Uniprot|PHOsphoELM|PhosphoSi	4691	NCL	kktvtpakavTtpgkkgatpg
IPI00604620	102	106	unsp+PKC+cdk5+p38MAPK->unsp+cdk5+p38MAPK		Uniprot|PhosphoSite	4691	NCL	ktvtpakavtTpgkkgatpgk
IPI00604620	109	105	PKC->	PKC->	niprot|PHOsphoELM|PhosphoSi	4691	NCL	kktvtpakavTtpgkkgatpg
IPI00604620	109	106	unsp+PKC+cdk5+p38MAPK->unsp+cdk5+p38MAPK	unsp+PKC+cdk5+p38MAPK->unsp+cdk5+p38MAPK	Uniprot|PhosphoSite	4691	NCL	ktvtpakavtTpgkkgatpgk
IPI00604620	109	113	unsp+p38MAPK->p38MAPK	unsp+p38MAPK->unsp+cdc2+p38MAPK	Uniprot	4691	NCL	avttpgkkgaTpgkalvatpg
IPI00604620	116	113	unsp+p38MAPK->p38MAPK		Uniprot	4691	NCL	avttpgkkgaTpgkalvatpg
IPI00604620	124	121	GSK3+cdk5+p38MAPK->GSK3+p38MAPK	GSK3+cdk5+p38MAPK->GSK3+p38MAPK	niprot|PHOsphoELM|PhosphoSi	4691	NCL	gatpgkalvaTpgkkgaaipa
IPI00604620	318	319		unsp->		4691	NCL	lfvgnlnfnkSapelktgisd
IPI00604620	333	328	cdc2->PKA+cdc2			4691	NCL	ksapelktgiSdvfakndlav
IPI00604620	377	386	unsp+PKG+PKA->unsp+PKG+cdc2+PKA	unsp+PKG+PKA->unsp+PKG+cdc2		4691	NCL	iklekpkgkdSkkerdartll
IPI00604620	398	394	PKC->			4691	NCL	kdskkerdarTllaknlpykv
IPI00604620	398	402	unsp->	unsp->	PhosphoSite	4691	NCL	artllaknlpYkvtqdelkev
IPI00604620	403	402	unsp->	unsp->	PhosphoSite	4691	NCL	artllaknlpYkvtqdelkev
IPI00604620	444	445	unsp+CKII->unsp+CKI+CKII			4691	NCL	efkteadaekTfeekqgteid
IPI00604620	467	462	->INSR	->INSR	PhosphoSite	4691	NCL	teidgrsislYytgekgqnqd
IPI00604620	513	510	PKC->			4691	NCL	etlqevfekaTfikvpqnqng
IPI00604620	572	571	->PKA	->DNAPK		4691	NCL	rgspnarsqpSktlfvkglse
IPI00604620	572	573		PKC->unsp+PKC		4691	NCL	spnarsqpskTlfvkglsedt
IPI00604620	572	580	unsp+PKA+CKII->unsp+CKII	unsp+PKA+CKII->unsp+CKII	niprot|PHOsphoELM|PhosphoSi	4691	NCL	psktlfvkglSedtteetlke
IPI00604620	577	568	DNAPK->cdc2+DNAPK			4691	NCL	qgprgspnarSqpsktlfvkg
IPI00604620	577	571	->PKA			4691	NCL	rgspnarsqpSktlfvkglse
IPI00604620	577	573	PKC->	PKC->		4691	NCL	spnarsqpskTlfvkglsedt
IPI00604620	577	580	unsp+PKA+CKII->unsp+CKI+CKII	unsp+PKA+CKII->unsp+CKII	niprot|PHOsphoELM|PhosphoSi	4691	NCL	psktlfvkglSedtteetlke
IPI00604620	577	583	unsp+CKII->unsp+CKI+CKII			4691	NCL	tlfvkglsedTteetlkesfd
IPI00604624	109	107	unsp+PKC->PKC	unsp+PKC->		60491	NIF3L1	frpmkritwnTwkerlviral
IPI00607584	71	68	PKG+cdc2+PKA->cdc2+PKA			10514	MYBBP1A	leylrgrpkgSemkyalkrli
IPI00607584	1317	1314	PKC+cdc2->PKC	PKC+cdc2->PKC	niprot|PHOsphoELM|PhosphoSi	10514	MYBBP1A	lvirspsllqSgakkkaqtlr
IPI00607584	1317	1322		unsp+PKC->unsp+PKC+cdc2		10514	MYBBP1A	lqsgakkkaqTlrftissskk
IPI00607591	421	413		cdc2->		5910	RAP1GDS1	vteavlkflkSemppvqfkll
IPI00607591	421	425	PKC->			5910	RAP1GDS1	mppvqfkllgTlrmlidaqae
IPI00607708	5	3	unsp+PKC->	unsp+PKC->		3939	LDHA	--------maTlkdqliynll
IPI00607708	81	79	unsp+PKC->unsp	unsp+PKC->unsp		3939	LDHA	slflrtpkivSgkdynvtans
IPI00607708	222	220	unsp+PKC->PKC+CKII	unsp+PKC->CKII		3939	LDHA	slktlhpdlgTdkdkeqwkec
IPI00607708	318	319	PKG+PKA->PKA	PKG+PKA->PKA		3939	LDHA	tseeearlkkSadtlwgiqke
IPI00607708	318	322	->CKI	->CKI		3939	LDHA	eearlkksadTlwgiqkelqf
IPI00607799	48	49	unsp->	unsp->		56898	BDH2	nesklqelekYpgiqtrvldv
IPI00607799	151	149	unsp+PKC->PKC	unsp+PKC->PKC		56898	BDH2	kgvvnrcvysTtkaavigltk
IPI00607799	151	150	PKC->	PKC->DNAPK		56898	BDH2	gvvnrcvystTkaavigltks
IPI00640088	231	228	unsp+cdk5->p38MAPK			1460	CSNK2B	lqlqaasnfkSpvktir----
IPI00640136	704	702	unsp+PKC->	unsp+PKC->		64864	RFX7	svkkdqkvphSgktegstaga
IPI00640136	704	705	unsp->unsp+CKI			64864	RFX7	kdqkvphsgkTegstagaqip
IPI00641743	288	293	PKA->	PKA->		3054	HCFC1	vathekewkcTntlaclnldt
IPI00641743	813	808	PKC->			3054	HCFC1	iittkvmtsgTgapakiitav
IPI00641743	2050	2048	unsp+PKC->	unsp+PKC->		3054	HCFC1	patqvrwlqeTskdssgtkpa
IPI00641743	2050	2049		CKI->ATM+CKI+DNAPK		3054	HCFC1	atqvrwlqetSkdssgtkpan
IPI00641743	2050	2052	unsp->CKI	unsp->unsp+CKI		3054	HCFC1	vrwlqetskdSsgtkpankrp
IPI00641743	2073	2070	PKC->	PKC->		3054	HCFC1	krpmsspemkSapkkskadgq
IPI00641743	2073	2075	unsp+PKG->unsp	unsp+PKG->unsp		3054	HCFC1	spemksapkkSkadgq-----
IPI00641743	2074	2070	PKC->	PKC->		3054	HCFC1	krpmsspemkSapkkskadgq
IPI00641788	73	69	unsp+CKI+PKC->unsp+CKI+PKA	unsp+CKI+PKC->unsp+CKI+PKA		6631	SNRPC	yqkwmeeqaqSlidkttaafq
IPI00641788	73	75	->PKC			6631	SNRPC	eqaqslidktTaafqqkipp
IPI00641829	36	38	unsp+PKC+PKA->PKC	unsp+PKC+PKA->	PhosphoSite	7919	BAT1	eapakkdvkgSyvsihssgfr
IPI00641829	36	39	INSR+unsp->	INSR+unsp->unsp	PhosphoSite	7919	BAT1	apakkdvkgsYvsihssgfrd
IPI00641829	36	41	unsp+PKC->CKI	unsp+PKC->	PhosphoSite	7919	BAT1	akkdvkgsyvSihssgfrdfl
IPI00641829	349	345		->EGFR		7919	BAT1	gmpqeerlsrYqqfkdfqrri
IPI00642097	237	234	PKC->	PKC->		23708	GSPT2	mfltgmvdkrTlekyereake
IPI00642097	325	321		unsp+CKII->CKII		23708	GSPT2	visarkgefeTgfekggqtre
IPI00642097	325	329	unsp->	unsp->		23708	GSPT2	fetgfekggqTrehamlakta
IPI00642156	486	479	->cdc2			124454	EARS2	lkklseglegTkysnvmkllr
IPI00642156	486	482	->PKA	->PKA		124454	EARS2	lseglegtkySnvmkllrmal
IPI00642186	951	956	PKA->	PKA->		9742	IFT140	elyvnkmkdkTlwrwwaqyle
IPI00642211	454	457	PKA->	PKA->		6051	RNPEP	kayvhefkfrSiladdfldfy
IPI00642329	36	34	PKC->	PKC->		9927	MFN2	vnasplkhfvTakkkingife
IPI00642329	243	245	unsp->			9927	MFN2	qtekhffhkvSerlsrpnifi
IPI00642374	170	167	PKC+CKII->CKII	PKC+CKII->CKII		51377	UCHL5	rqqmfefdtkTsakeedafhf
IPI00642550	649	645	unsp+PKG+cdc2+PKA->unsp+PKA+RSK	unsp+PKG+cdc2+PKA->unsp+PKG+cdc2+PKA+RSK		728118	FAM22A	apsrgtarldSssskfaagqg
IPI00642550	649	647	unsp+PKC->	unsp+PKC->		728118	FAM22A	srgtarldssSskfaagqgae
IPI00642550	649	648	PKC->CKI+PKC	PKC->ATM+DNAPK		728118	FAM22A	rgtarldsssSkfaagqgaer
IPI00642904	104	96	unsp->unsp+PKA			8761	PABPC4	wsqrdpslrkSgvgnvfiknl
IPI00642904	104	109	unsp->	unsp->		8761	PABPC4	gnvfiknldkSidnkalydtf
IPI00643027	892	886		->ATM		23367	LARP1	shellkengfTqhvyhkyrrr
IPI00643027	1010	1014		->EGFR		23367	LARP1	nldidpklqeYlgkfrrledf
IPI00643027	1017	1014	->EGFR	->EGFR		23367	LARP1	nldidpklqeYlgkfrrledf
IPI00643041	99	97	unsp+PKC->	unsp+PKC->		221547	RANP1	iimfdvtsrvTyknvpnwhrd
IPI00643041	159	150	->cdc2			221547	RANP1	rkknlqyydiSaksnynfekp
IPI00643041	159	155		->EGFR	Uniprot|PhosphoSite	221547	RANP1	qyydisaksnYnfekpflwla
IPI00643435	539	540	INSR+unsp->unsp		PhosphoSite	55210	ATAD3A	erlvrmyfdkYvlkpategkq
IPI00643591	101	99	ATM+unsp+PKC+DNAPK->ATM+cdc2+DNAPK	ATM+unsp+PKC+DNAPK->ATM+DNAPK		164	AP1G1	gqleclkliaSqkftdkrigy
IPI00643591	167	172	PKG+PKA->			164	AP1G1	agevekllktSnsylrkkaal
IPI00643722	997	995	unsp+PKC->unsp	unsp+PKC->unsp		8289	ARID1A	adgtpkteskSkksssssttn
IPI00643722	997	998	unsp+PKC+PKA->unsp+PKC+cdc2+PKA	unsp+PKC+PKA->unsp+PKC+cdc2+PKA		8289	ARID1A	tpkteskskkSsssttneki
IPI00643722	997	999	unsp+PKA->unsp+PKC+PKA			8289	ARID1A	pkteskskksSssttnekit
IPI00643722	997	1001	unsp->unsp+CKI	unsp->unsp+CKI+cdc2		8289	ARID1A	teskskksssSttnekitkl
IPI00643722	1007	998	unsp+PKC+PKA->unsp+PKC+cdc2+PKA			8289	ARID1A	tpkteskskkSsssttneki
IPI00643722	1007	1002	PKC->			8289	ARID1A	eskskksssSTttnekitkly
IPI00643722	1007	1003		unsp+CKII->CKII		8289	ARID1A	skskksssstTtnekitklye
IPI00643722	1007	1004	PKC->	PKC->		8289	ARID1A	kskksssstTnekitklyel
IPI00643722	1612	1609	PKC->	PKC->		8289	ARID1A	tspskspflhSgmkmqkagpp
IPI00643722	1905	1908	PKA->PKG	PKA->		8289	ARID1A	ppdgppekriTatmddmlstr
IPI00643722	1905	1910	PKC->	PKC->		8289	ARID1A	dgppekritaTmddmlstrss
IPI00643920	6	3	PKC->	PKC->		7086	TKT	--------meSyhkpdqqklq
IPI00643920	144	146	->PKC			7086	TKT	aytgkyfdkaSlpsswdysyr
IPI00643920	144	149	unsp->	unsp->		7086	TKT	gkyfdkaslpSswdysyrvyc
IPI00644055	369	363	unsp+CKI+CKII->unsp+CKII	unsp+CKI+CKII->unsp+cdc2+CKII		10236	HNRNPR	ttvteeilekSfsefgklerv
IPI00644055	369	365		CKI->unsp+CKI		10236	HNRNPR	vteeileksfSefgklervkk
IPI00644712	31	33		unsp+PKC->unsp		2547	XRCC6	nleasgdykySgrdsliflvd
IPI00644712	317	319	ATM+unsp+PKG->ATM	ATM+unsp+PKG->ATM+unsp+DNAPK		2547	XRCC6	glllpsdtkrSqiygsrqiil

Table S3

IPI00644712	317	322	INSR->EGFR			2547	XRCC6	lpsdtkrsqiYgsrqiileke
IPI00644712	317	324	->ATM			2547	XRCC6	sdtkrsqiygSrqiilekeet
IPI00644712	461	455	unsp+cdk5+p38MAPK->unsp+p38MAPK	unsp+cdk5+p38MAPK->unsp+p38MAPK	Uniprot|PhosphoSite	2547	XRCC6	kmpftekimaTpeqvgkmkai
IPI00644712	542	541		unsp+PKC->ATM+unsp+PKC		2547	XRCC6	ppdynpegkvTkrkhdnegsg
IPI00644712	544	541	unsp+PKC->unsp			2547	XRCC6	ppdynpegkvTkrkhdnegsg
IPI00644712	553	552	unsp+PKC->PKC	unsp+PKC->ATM+unsp+PKC+DNAPK		2547	XRCC6	krkhdnegsgSkrpkveysee
IPI00644712	556	552	unsp+PKC->unsp			2547	XRCC6	krkhdnegsgSkrpkveysee
IPI00644712	556	559	INSR+unsp->	INSR+unsp->		2547	XRCC6	gsgskrpkveYseeelkthis
IPI00645078	671	668	PKC->CKII			7317	UBA1	qpaenvnqylTdpkfvertlr
IPI00645078	671	676	unsp+PKC->unsp	unsp+PKC->unsp		7317	UBA1	yltdpkfverTlrlagtqple
IPI00645078	980	981		CKII->unsp+CKII		7317	UBA1	tlkqfldyfkTehkleitmls
IPI00645616	507	511	unsp+PKA->PKA	unsp+PKA->unsp+cdc2		414189	AGAP6	wpvelrkvmsSivndlansiw
IPI00645616	530	532	unsp+PKC+PKG->unsp+PKC	unsp+PKC+PKG->unsp		414189	AGAP6	egssqgqtkpSekstreeker
IPI00645616	530	535	unsp+PKC+CKII->unsp+CKI+PKC+CKII	unsp+PKC+CKII->unsp+CKII		414189	AGAP6	sqgqtkpsekStreekerwir
IPI00645643	398	394	unsp+PKC->unsp			80114	BICC1	ikpkpkqpskSvivksverna
IPI00646058	475	478	unsp+PKC->cdc2	unsp+PKC->PKC+cdc2		6294	SAFB	hktelhgkmiSvekaknepvg
IPI00646058	607	601	unsp->unsp+PKA			6294	SAFB	drksasrekrSvvsfdkvkep
IPI00646058	607	604	CKI+PKC->PKC			6294	SAFB	sasrekrsvvSfdkvkeprks
IPI00646377	944	952	unsp->INSR+unsp	unsp->INSR+unsp		8672	EIF4G3	fekakprmdqYfnqmekivke
IPI00646645	1647	1655	PKA->cdc2			23064	SETX	aqkpvgemknScnvlhpqspn
IPI00646689	78	76	unsp->			84817	TXNDC17	fiycqvgekpYwkdpnndfrk
IPI00646762	42	40	PKC->	PKC->		11164	NUDT5	klekttymdpTgktrrnkekt
IPI00646839	559	555		->EGFR		728689	EIF3CL	savlmerlckYiyakdrtdri
IPI00646839	559	557		->unsp		728689	EIF3CL	vlmerlckyiYakdrtdrirt
IPI00646909	401	399	unsp->			51807	TUBA8	arldhkfdlmYakrafvhwyv
IPI00646917	23	18	unsp+PKC->unsp	unsp+PKC->unsp		11051	NUDT21	rsqtgwprgvTqfgnkyiqqt
IPI00646917	23	24	unsp+EGFR->	unsp+EGFR->EGFR		11051	NUDT21	prgvtqfgnkYiqqtkpltle
IPI00646917	23	28		unsp+PKC->unsp		11051	NUDT21	tqfgnkyiqqTkpltlertin
IPI00646917	29	24	unsp+EGFR->unsp	unsp+EGFR->unsp		11051	NUDT21	prgvtqfgnkYiqqtkpltle
IPI00646917	29	28	unsp+PKC->unsp+PKA	unsp+PKC->unsp+DNAPK		11051	NUDT21	tqfgnkyiqqTkpltlertin
IPI00646917	56	58	unsp+cdc2->cdc2			11051	NUDT21	gtkeplyekdSsvaarfqrmr
IPI00646917	56	59	PKC->			11051	NUDT21	tkeplyekdsSvaarfqrmre
IPI00647217	51	54		->CKII		23517	SKIV2L2	agkrfdgklqSestnngknkr
IPI00647217	51	56	unsp->	unsp->		23517	SKIV2L2	krfdgklqseStnngknkrdv
IPI00647217	51	57	->CKI	->CKI		23517	SKIV2L2	rfdgklqsesTnngknkrdvd
IPI00647217	79	87	CKII->cdc2+CKII			23517	SKIV2L2	gkkprieesiTedlsladlmp
IPI00647217	198	203		unsp+EGFR->unsp		23517	SKIV2L2	alsnqkyremYeefqdvglmt
IPI00647491	512	513	unsp+PKC->ATM+unsp+PKC			9692	KIAA0391	dhkaclpdakTqrlffkwqqg
IPI00654555	91	88	unsp+PKC->PKC			4839	NOP2	pkgisagavqTagkkgpqslf
IPI00654555	91	96	unsp+PKA->	unsp+PKA->		4839	NOP2	vqtagkkgpqSlfnaprgkkr
IPI00654555	682	679	->PKA	->PKA		4839	NOP2	lqkqqhpkkaSfqklngiskg
IPI00654731	858	854	unsp->unsp+PKA			23586	DDX58	srphpkpkqfSsfekrakifc
IPI00654731	909	906	PKC->			23586	DDX58	diatgvqtlySkwkdfhfeki
IPI00654777	253	248	CKI->CKI+p38MAPK	CKI->		8665	EIF3F	vpgrtmgvmfTpltvkyayyd
IPI00654777	253	251	unsp+PKC->PKC	unsp+PKC->PKC		8665	EIF3F	rtmgvmftplTvkyayydter
IPI00656021	360	351	->cdc2			55275	VPS53	vklllfaiqrTtnfegflakr
IPI00656021	360	363	unsp+PKA->PKA	unsp+PKA->PKA		55275	VPS53	nfegflakrfSgctltdgtlk
IPI00657687	371	368	PKC->	PKC->	Uniprot	54443	ANLN	skdksttpggTgikpflerfg
IPI00657967	52	48	unsp+PKC->unsp	unsp+PKC->unsp		65979	PHACTR4	tpptkrkskfSgfgkifkpwk
IPI00658023	198	197	unsp->	unsp->		5781	PTPN11	fdsltdlvehYkknpmvetlg
IPI00658023	199	197	unsp->			5781	PTPN11	fdsltdlvehYkknpmvetlg
IPI00658023	280	279	INSR+unsp->INSR+unsp+EGFR	INSR+unsp->INSR+unsp+EGFR	PhosphoSite	5781	PTPN11	qrqenknknrYknilpfdhtr
IPI00658210	121	119		->unsp		57599	WDR48	cmstlrthkdYvkalayakdk
IPI00658210	214	210	->PKA	->PKA		57599	WDR48	caklmklkghTdnvkalllnr
IPI00658210	578	574	PKC->	PKC->		57599	WDR48	kipfylqphaSsgaktlkkdr
IPI00658210	581	579	unsp+PKC->	unsp+PKC->		57599	WDR48	lqphassgakTlkkdrlsasd
IPI00658210	581	586		unsp+PKA->unsp+PKG+PKA		57599	WDR48	gaktlkkdrlSasdmlqvrkv
IPI00658210	581	588	->cdc2			57599	WDR48	ktlkkdrlsaSdmlqvrkvme
IPI00718888	179	183	unsp+PKA+RSK->unsp+PKA	unsp+PKA+RSK->unsp+PKA		5634	PRPS2	pdaggakrvtSiadrlnvefa
IPI00719285	1763	1762	unsp->unsp+DNAPK	unsp->ATM+unsp+DNAPK		11113	CIT	vvilrynenlSkycirkeiet
IPI00719680	176	180	PKC->			8943	AP3D1	mykvflkypeSlrpafprlke
IPI00719752	213	212		CKI+PKC->CKI+PKC+DNAPK		8662	EIF3B	klknvihkifSkfgkitndfy
IPI00719752	213	218	PKG->			8662	EIF3B	hkifskfgkiTndfypeedgk
IPI00719752	436	432	PKG->PKG+PKA			8662	EIF3B	ssahwpifkwShdgkffarmt
IPI00719752	729	728	->PKA			8662	EIF3B	kqikkdlkkySkifeqkdrls
IPI00740057	1773	1769	PKC->	PKC->		256006	ANKRD31	vtwnyawskvTylgkellryv
IPI00740057	1773	1770	unsp->	unsp->		256006	ANKRD31	twnyawskvtYlgkellryvs
IPI00740961	175	167	unsp+PKC->unsp+PKC+cdc2			26173	INTS1	algskgqaneSktastllkpa
IPI00740961	175	171	PKC->PKA	PKC->PKA		26173	INTS1	kgqanesktaStllkpapsgl
IPI00740961	175	172	PKC+cdc2->cdc2	PKC+cdc2->cdc2		26173	INTS1	gqanesktasTllkpapsglp
IPI00742682	457	453	PKC->PKA	PKC->PKA		7175	TPR	eyeraqkavaSlsvkleqamk
IPI00742682	457	455	unsp+PKC->PKC	unsp+PKC->		7175	TPR	eraqkavaslSvkleqamkei
IPI00742682	713	712		unsp+PKC->unsp+DNAPK		7175	TPR	eqvtdlrsqnTkistqldfas
IPI00742682	713	715	cdc2+PKA->cdc2	cdc2+PKA->cdc2		7175	TPR	tdlrsqntkiStqldfaskry
IPI00742682	713	716	DNAPK->ATM+cdc2+DNAPK	DNAPK->cdc2+DNAPK		7175	TPR	dlrsqntkisTqldfaskrye
IPI00742682	723	716	DNAPK->cdc2+DNAPK			7175	TPR	dlrsqntkisTqldfaskrye
IPI00742682	723	722	PKC->	PKC->DNAPK		7175	TPR	tkistqldfaSkryemlqdnv
IPI00742682	748	750		PKC->		7175	TPR	tslhernqklTattqkqeqii
IPI00742682	748	752	PKC->			7175	TPR	lhernqkltaTtqkqeqiint
IPI00742682	755	750	PKC->PKG	PKC->		7175	TPR	tslhernqklTattqkqeqii
IPI00742682	755	752	PKC->	PKC->		7175	TPR	lhernqkltaTtqkqeqiint
IPI00742682	755	753	unsp+PKC->	unsp+PKC->		7175	TPR	hernqkltatTqkqeqiintm
IPI00742743	1672	1670	unsp+PKC->PKC	unsp+PKC->		7158	TP53BP1	esprasmgvlSgkrklitsee
IPI00743143	112	119	unsp+CKII->unsp+cdc2+CKII	unsp+CKII->unsp+cdc2+CKII		51147	ING4	adlkekqiesSdydsssskgk
IPI00743143	127	119	unsp+CKII->unsp+cdc2+CKII			51147	ING4	adlkekqiesSdydsssskgk
IPI00743143	127	123	unsp+PKC->unsp	unsp+PKC->unsp		51147	ING4	ekqiessdydSssskgkkkgr
IPI00743143	127	124	unsp+PKC+cdc2->cdc2	unsp+PKC+cdc2->unsp		51147	ING4	kqiessdydsSsskgkkkgrt
IPI00743143	127	125	unsp+CKI+PKC->unsp+CKI+PKC+cdc2			51147	ING4	qiessdydssSskgkkkgrtq
IPI00743143	127	126		unsp+CKI+PKC+cdc2->ATM+unsp+CKI+PKC+cdc2+DNAPK		51147	ING4	iessdydsssSkgkkkgrtqk
IPI00743143	129	124	unsp+PKC+cdc2->cdc2	unsp+PKC+cdc2->cdc2		51147	ING4	kqiessdydsSsskgkkkgrt
IPI00743143	129	126	unsp+CKI+PKC+cdc2->unsp+PKC+cdc2			51147	ING4	iessdydsssSkgkkkgrtqk
IPI00743143	130	125		unsp+CKI+PKC->unsp+PKC		51147	ING4	qiessdydssSskgkkkgrtq
IPI00743143	130	126	unsp+CKI+PKC+cdc2->unsp+CKI+PKC			51147	ING4	iessdydsssSkgkkkgrtqk
IPI00743143	131	125	unsp+CKI+PKC->unsp+PKC	unsp+CKI+PKC->unsp+PKC+cdc2		51147	ING4	qiessdydssSskgkkkgrtq
IPI00743143	131	126	unsp+CKI+PKC+cdc2->unsp+PKC+cdc2	unsp+CKI+PKC+cdc2->unsp+PKC+cdc2		51147	ING4	iessdydsssSkgkkkgrtqk
IPI00743143	131	134	unsp+PKC->unsp+PKC+PKG			51147	ING4	ssskgkkkgrTqkekkaarar
IPI00743143	146	145		unsp+PKC+RSK->ATM+unsp+PKC+DNAPK+RSK		51147	ING4	qkekkaararSkgknsdeeap
IPI00743143	148	145	unsp+PKC+RSK->unsp+PKA	unsp+PKC+RSK->unsp+PKC		51147	ING4	qkekkaararSkgknsdeeap
IPI00743143	156	157	->PKC	->PKC		51147	ING4	gknsdeeapkTaqkklklvrt
IPI00743143	160	168	unsp+PKA->unsp	unsp+PKA->unsp		51147	ING4	aqkklklvrtSpeygmpsvtf
IPI00743157	790	789	->PKA	->ATM+DNAPK		54904	WHSC1L1	vrkfptaifeSkgfrcpqhcc
IPI00743335	704	708	unsp->			4641	MYO1C	vrhlgykpeeYkmgrtkifir
IPI00743509	168	169		unsp+GSK3+cdk5+p38MAPK->unsp+cdk5+p38MAPK		1871	E2F3	aalrspdspkTpkspsektry
IPI00743509	171	166	unsp+GSK3+cdk5+p38MAPK->unsp+GSK3+p38MAPK	unsp+GSK3+cdk5+p38MAPK->unsp+GSK3+p38MAPK		1871	E2F3	kgraalrspdSpktpkspsek
IPI00743509	171	172	unsp+GSK3->unsp+GSK3+p38MAPK	unsp+GSK3->unsp+p38MAPK		1871	E2F3	rspdspktpkSpsektrydts
IPI00743509	176	172	unsp+GSK3->unsp+GSK3+cdk5			1871	E2F3	rspdspktpkSpsektrydts
IPI00743509	176	174	unsp->	unsp->		1871	E2F3	pdspktpkspSektrydtslg
IPI00743509	176	179	INSR+unsp->	INSR+unsp->unsp	PhosphoSite	1871	E2F3	tpkspsektrYdtslglltkk
IPI00743509	176	181	unsp+PKA->unsp	unsp+PKA->unsp		1871	E2F3	kspsektrydTslglltkkfi
IPI00743594	372	369	PKC->	PKC->		54443	ANLN	skdksttpggTgikpflerfg
IPI00743879	409	405	PKC->PKA	PKC->PKA		89953	KLC4	nvartknnlaScylkqgkyae
IPI00743879	409	413	unsp+EGFR->			89953	KLC4	lascylkqgkYaeaetlykei
IPI00744507	102	106	unsp->			1457	CSNK2A1	tladivkdpvSrtpalvfehv
IPI00744507	102	108	p38MAPK->			1457	CSNK2A1	adivkdpvsrTpalvfehvnn
IPI00744692	219	216	PKC->	PKC->		6888	TALDO1	yepledpgvkSvtkiynyykk
IPI00744692	219	218	PKC->	PKC->		6888	TALDO1	pledpgvksvTkiynyykkfs
IPI00744692	286	282	CKII->unsp+CKII	CKII->unsp+CKII		6888	TALDO1	pvlsakaaqaSdlekihldek
IPI00744711	264	266	PKG->	PKG->		87178	PNPT1	iqgiqqlvkeTgvtkrtpqkl
IPI00744711	275	278		->p38MAPK		87178	PNPT1	vtkrtpqklfTpspeivkyth
IPI00744711	275	280	unsp->	unsp->		87178	PNPT1	krtpqklftpSpeivkythkl
IPI00744711	285	278	->p38MAPK			87178	PNPT1	vtkrtpqklfTpspeivkyth
IPI00744711	285	280	unsp->	unsp->		87178	PNPT1	krtpqklftpSpeivkythkl
IPI00744711	285	287		PKC->unsp+PKC		87178	PNPT1	ftpspeivkyThklamerlya
IPI00744711	289	287	PKC->	PKC->		87178	PNPT1	ftpspeivkyThklamerlya
IPI00744711	591	594	unsp+PKC->unsp+PKC+cdc2	unsp+PKC->unsp+PKC+cdc2		87178	PNPT1	eilqimnktiSkprasrkeng
IPI00744851	15	14		unsp->ATM+unsp+DNAPK		3159	HMGA1	ssskssqplaSkqekdgtekr
IPI00745087	200	196	cdc2->			79843	FAM124B	lslkqlppgmSvdpkessvlq
IPI00745087	200	202	PKG->	PKG->		79843	FAM124B	ppgmsvdpkeSsvlqfkvqei

IPI00745087	200	203	PKC->PKC+cdc2	PKC->PKC+cdc2		79843	FAM124B	pgmsvdpkesSvlqfkvqeig
IPI00745266	465	469	PKC->	PKC->PKC+cdc2		51386	EIF3L	tirsflklytTmpvaklagfl
IPI00745568	207	208	->cdc2+CKII	->cdc2+CKII		261726	TIPRL	dtrlyheadkTymlreytsre
IPI00745568	207	209	INSR->	INSR->	Uniprot|PhosphoSite	261726	TIPRL	trlyheadktYmlreytsres
IPI00745793	73	69	unsp+PKG->unsp+PKG+PKA		Uniprot|PhosphoSite	891	CCNB1	kmpmkkeakpSatgkvidkkl
IPI00745793	73	71	PKC->	PKC->		891	CCNB1	pmkkeakpsaTgkvidkklpk
IPI00745872	223	217		unsp+PKC->unsp+PKC+cdc2		213	ALB	delrdegkasSakqrlkcasl
IPI00745872	229	226	PKC->PKA	PKC->PKC+PKA		213	ALB	ssakqrlkcaSlqkfgerafk
IPI00745872	438	436	unsp+PKC+cdc2->cdc2	unsp+PKC+cdc2->cdc2		213	ALB	kfqnallvryTkkvpqvstpt
IPI00745872	499	504	PKA->			213	ALB	sdrvtkccteSlvnrrpcfsa
IPI00746165	81	79	unsp+PKC->	unsp+PKC->		9948	WDR1	sgfyiasgdvSgklriwdttq
IPI00746165	115	114		->DNAPK		9948	WDR1	kikdiawtedSkriavvgegr
IPI00746165	180	184	PKC->			9948	WDR1	fegppfkfkfTigdhsrfvnc
IPI00746165	182	184		PKC->		9948	WDR1	fegppfkfkfTigdhsrfvnc
IPI00746165	480	478	unsp+PKC->	unsp+PKC->		9948	WDR1	nvrlysilgtTlkdegkllea
IPI00746251	12	15	unsp+PKC->PKC	unsp+PKC->PKC	Uniprot	337874	HIST2H2BD	akfapakkgSkkavtkaqkk
IPI00746251	13	15	unsp+PKC->PKC		Uniprot	337874	HIST2H2BD	akfapakkgSkkavtkaqkk
IPI00746310	7	4	PKC->	PKC->		3159	HMGA1	-------mseSsskssqplas
IPI00746310	7	5	unsp+PKC->cdc2	unsp+PKC->		3159	HMGA1	------msesSskssqplask
IPI00746310	7	6	unsp+PKC->unsp+DNAPK	unsp+PKC->unsp+DNAPK		3159	HMGA1	-----msessSkssqplaskq
IPI00746310	7	8	unsp->	unsp->		3159	HMGA1	---msessskSsqplaskqek
IPI00746310	7	9	ATM+unsp+DNAPK->ATM+PKC+DNAPK			3159	HMGA1	--msessksSqplaskqekd
IPI00746310	15	14		unsp->ATM+unsp+DNAPK		3159	HMGA1	ssskssqplaSkqekdgtekr
IPI00746310	23	21	unsp->	unsp->	PhosphoSite	3159	HMGA1	plaskqekdgTekrgrgrprk
IPI00746310	31	36		unsp+GSK3->unsp+GSK3+p38MAPK	Uniprot|PhosphoSite	3159	HMGA1	rgrprkqppvSpgtalvgsqk
IPI00746310	46	44	ATM+unsp+DNAPK->ATM+unsp+cdc2+CKII+DNAPK		Uniprot|PhosphoSite	3159	HMGA1	pvspgtalvgSqkepsevptp
IPI00746310	46	53	unsp+GSK3+cdk5->unsp+GSK3+cdk5+p38MAPK		Uniprot|PHOsphoELM|PhosphoSi	3159	HMGA1	gsqkepsevpTpkrprgrpkg
IPI00746310	55	53	unsp+GSK3+cdk5->GSK3+cdk5+p38MAPK	unsp+GSK3+cdk5->GSK3+cdk5+p38MAPK	ıiprot|PHOsphoELM|PhosphoSi	3159	HMGA1	gsqkepsevpTpkrprgrpkg
IPI00746310	55	64	unsp+PKC+PKG+PKA->unsp+PKC+PKG+cdc2+PKA	unsp+PKC+PKG+PKA->unsp+PKC+PKG+cdc2	PhosphoSite	3159	HMGA1	pkrprgrpkgSkknwrrrkrr
IPI00746310	62	64	unsp+PKC+PKG+PKA->unsp+PKC	unsp+PKC+PKG+PKA->unsp+PKC	PhosphoSite	3159	HMGA1	pkrprgrpkgSkknwrrrkrr
IPI00746310	65	64	unsp+PKC+PKG+PKA->unsp+PKC+PKA	unsp+PKC+PKG+PKA->unsp+PKG+DNAPK	PhosphoSite	3159	HMGA1	pkrprgrpkgSkknwrrrkrr
IPI00746351	922	916	->cdc2	->cdc2		22894	DIS3	kvkvkimldsSnlqhqkirms
IPI00746412	1392	1395	unsp->			9169	SFRS2IP	qevklaikpfYqnkditkeey
IPI00746655	689	684	->PKA	->PKA		23344	ESYT1	rflgglvkgkSdpyvklklag
IPI00746655	827	830	unsp->unsp+GSK3	unsp->unsp+p38MAPK	PhosphoSite	23344	ESYT1	lplrkgtkhlSpyatltvgds
IPI00746777	366	364	unsp->	unsp->		642443	ADH5P4	einkafelmhSgksirtvvki
IPI00746777	366	367	unsp+PKC->unsp+CKI+PKC	unsp+PKC->PKC		642443	ADH5P4	kafelmhsgkSirtvvki---
IPI00746777	366	370	PKC->			642443	ADH5P4	elmhsgksirTvvki------
IPI00746934	1084	1077	unsp+PKC->unsp+PKC+cdc2			23352	UBR4	hassllelasTtkcssvkydv
IPI00746934	1084	1081	PKG->PKG+PKA	PKG->PKG+PKA		23352	UBR4	llelasttkcSsvkydveive
IPI00746934	1084	1082	unsp+PKC->PKC	unsp+PKC->		23352	UBR4	lelasttkcsSvkydveivee
IPI00747810	471	465	INSR+unsp+EGFR->unsp+EGFR	INSR+unsp+EGFR->unsp+EGFR		6624	FSCN1	pvdfffefcdYnkvaikvggr
IPI00748360	819	823	unsp+PKC->PKC	unsp+PKC->PKC		54914	KIAA1797	ipnfilkmyeTnkqpglkpgl
IPI00748532	616	615		PKC->PKC+DNAPK		8202	NCOA3	gaenqrgpleSkghkkllqll
IPI00748532	619	615	PKC->	PKC->unsp+PKC		8202	NCOA3	gaenqrgpleSkghkkllqll
IPI00749113	179	174	unsp+PKC->unsp	unsp+PKC->PKC		1854	DUT	sgcygrvaprSglaakhfidv
IPI00749237	14	17	INSR+unsp+EGFR->	INSR+unsp+EGFR->SRC+unsp		9556	C14orf2	kniwipmkpyYtkvyqeiwig
IPI00749406	10	6	PKC->			80176	SPSB1	-----mgqkvTggiktvdmrd
IPI00749406	10	11	->unsp	->unsp		80176	SPSB1	mgqkvtggikTvdmrdptyrp
IPI00759562	235	240	unsp+PKA->PKA	unsp+PKA->PKA		54890	ALKBH5	ckfqfkpirvSepvlslpvrr
IPI00759691	496	497	unsp+PKC->unsp+CKI+PKC			676	BRDT	mceqmrlkekSkrnqpkkrkq
IPI00759749	503	500	unsp->	unsp->		8505	PARG	ragevpkpfpThykdlwdnkh
IPI00761080	154	149	unsp+PKC+cdc2->unsp+cdc2	unsp+PKC+cdc2->cdc2		23286	WWC1	lgsqvslvsgSsssskydpei
IPI00761080	154	150		->unsp		23286	WWC1	gsqvslvsgsSssskydpeil
IPI00761080	154	151	unsp->			23286	WWC1	sqvslvsgssSsskydpeilk
IPI00761080	154	152	unsp+PKC+cdc2->cdc2	unsp+PKC+cdc2->cdc2		23286	WWC1	qvslvsgsssSskydpeilka
IPI00761080	154	153	unsp+CKII+cdc2->unsp+CKI+cdc2+DNAPK	unsp+CKII+cdc2->ATM+unsp+CKII+cdc2+DNAPK		23286	WWC1	vslvsgssssSkydpeilkae
IPI00782935	114	118	unsp+CKII->CKII		Uniprot|PHOsphoELM|PhosphoSi	84289	ING5	eadlkdkmegSdfessggrgl
IPI00782935	154	148	unsp+CKI+PKB+RSK->unsp+PKB+PKA+RSK	unsp+CKI+PKB+RSK->unsp+PKB+PKA+RSK		84289	ING5	rgsrgrgrrtSeedtpkkkkh
IPI00782950	614	607	unsp->unsp+PKA	unsp->unsp+PKA		5587	PRKD1	givyggkhrkTgrdvaikiid
IPI00782966	1350	1346	CKI+PKC->CKI			64397	ZFP106	qaestltsaeTrgskkkkklr
IPI00782966	1350	1349		unsp+PKC->unsp+PKC+DNAPK		64397	ZFP106	stltsaetrgSkkkkklrkkk
IPI00782966	1358	1360	unsp+PKC+PKA->unsp+PKC	unsp+PKC+PKA->unsp+PKC		64397	ZFP106	kkkkklrkkkSlraahvpens
IPI00782992	169	172	unsp+cdc2->cdc2	unsp+cdc2->cdc2		23524	SRRM2	qpapeppkpySlvresssrs
IPI00782992	169	177	unsp+PKA->unsp+cdc2+PKA			23524	SRRM2	ppkpyslvreSsssrsptpkq
IPI00782992	1186	1188		p38MAPK->	ıiprot|PHOsphoELM|PhosphoSi	23524	SRRM2	aspprqkdkfSpfpvqdrpes
IPI00782992	1467	1460	unsp->unsp+cdc2			23524	SRRM2	gsgtpsrhslSgsspgmkdip
IPI00782992	1467	1463	unsp+cdc2->unsp		PhosphoSite	23524	SRRM2	tpsrhslsgsSpgmkdiprtp
IPI00783004	943	941	unsp+PKC->PKC	unsp+PKC->PKC		5332	PLCB4	hlkkqqkelnSlkkkhakehs
IPI00783017	412	420	unsp+cdc2+CKII+PKA->unsp+cdc2+CKII	unsp+cdc2+CKII+PKA->unsp+cdc2+CKII		9839	ZEB2	rnklengkplSmseqtgllki
IPI00783017	417	420	unsp+cdc2+CKII+PKA->unsp+cdc2+CKII	unsp+cdc2+CKII+PKA->unsp+cdc2+CKII		9839	ZEB2	rnklengkplSmseqtgllki
IPI00783017	417	422		->cdc2		9839	ZEB2	klengkplsmSeqtgllkikt
IPI00783097	501	500	->CKI	->ATM+DNAPK		2617	GARS	ktvnvvqfepSkgaigkaykk
IPI00783271	155	152	PKC->PKA	PKC->PKA		10128	LRPPRC	rtefahriwdTlqklgavydv
IPI00783271	292	289	unsp+PKC->			10128	LRPPRC	ekgdidhvkqTlekvekselh
IPI00783271	750	743	->cdc2			10128	LRPPRC	lkeefdrldsSavldtgkyvg
IPI00783271	750	748	PKC->	PKC->		10128	LRPPRC	drldssavldTgkyvglvrvl
IPI00783271	750	751	unsp->	unsp->		10128	LRPPRC	dssavldtgkYvglvrvlakh
IPI00783302	126	123	PKC+PKG+DNAPK->PKA+DNAPK	PKC+PKG+DNAPK->PKC+PKG+PKA+DNAPK		55037	PTCD3	genvakfiinSypkyfqkdia
IPI00783559	454	461	unsp+DNAPK->ATM+unsp+DNAPK			55770	EXOC2	fvekltklvlSqlpnfwklwi
IPI00783835	305	299	->PKA	->PKA		26046	RNF160	eldsleekfkSllsqnkfwky
IPI00783835	305	302	PKC+DNAPK->DNAPK			26046	RNF160	sleekfksllSqnkfwkygkh
IPI00783835	308	313	unsp+PKC+PKA->unsp+PKC	unsp+PKC+PKA->unsp		26046	RNF160	qnkfwkygkhSvpqirsayfe
IPI00783874	179	175	unsp+PKC->	unsp+PKC->PKC		84064	HDHD2	aleyatdtkaTvvgkpektff
IPI00784090	20	23	unsp+cdc2+CKII->cdc2+CKII	unsp+cdc2+CKII->cdc2+CKII	Uniprot|PhosphoSite	10694	CCT8	qmlkegakhfSgleeavyrni
IPI00784090	318	317		unsp+PKA+RSK->unsp+PKA+DNAPK+RSK		10694	CCT8	kynimlvrlnSkwdlrrlckt
IPI00784090	400	398	unsp+PKC->	unsp+PKC->		10694	CCT8	ieravddgvnTfkvltrdkrl
IPI00784090	466	465		->ATM		10694	CCT8	nsgvkaneviSklyavhqegn
IPI00784090	466	468	INSR->			10694	CCT8	vkaneviskIYavhqegnknv
IPI00784154	87	83		->unsp		3329	HSPD1	vtkdgvtvakSidlkdkykni
IPI00784154	125	122	unsp->	unsp->		3329	HSPD1	gtttatvlarSiakegfekis
IPI00784154	130	132	unsp+PKA->PKC	unsp+PKA->		3329	HSPD1	siakegfekiSkganpveirr
IPI00784154	156	159	PKC+PKA->PKA			3329	HSPD1	daviaelkkqSkpvttpeeia
IPI00784154	157	159		PKC+PKA->PKA		3329	HSPD1	daviaelkkqSkpvttpeeia
IPI00784154	202	200	unsp+PKC->	unsp+PKC->		3329	HSPD1	mkkvgrkgviTvkdgktlnde
IPI00784154	233	227	INSR->	INSR->	ıiprot|PHOsphoELM|PhosphoSi	3329	HSPD1	mkfdrgyispYfintskgqkc
IPI00784154	233	231	unsp+PKC->	unsp+PKC->		3329	HSPD1	rgyispyfinTskgqkcefqd
IPI00784154	233	232	PKC->CKI	PKC->ATM+DNAPK		3329	HSPD1	gyispyfintSkgqkcefqda
IPI00784154	236	232	PKC->PKA	PKC->		3329	HSPD1	gyispyfintSkgqkcefqda
IPI00784154	249	247	unsp+PKC->PKC+RSK	unsp+PKC->PKC+RSK		3329	HSPD1	cefqdayvllSekkissiqsi
IPI00784154	250	252	unsp+PKA->unsp	unsp+PKA->unsp		3329	HSPD1	ayvllsekkiSsiqsivpale
IPI00784154	352	351		unsp+CKII->unsp+CKII+DNAPK		3329	HSPD1	dlgkvgevivTkddamllkgk
IPI00784154	389	381	CKII->cdc2+CKII			3329	HSPD1	iqeiieqldvTtseyekekln
IPI00784154	389	385	unsp->	unsp->SRC		3329	HSPD1	ieqldvttseYekeklnerla
IPI00784154	396	398	unsp->			3329	HSPD1	eklnerlaklSdgvavlkvgg
IPI00784154	462	453	->cdc2			3329	HSPD1	allrcipaldSltpanedqki
IPI00784154	469	471		PKC->		3329	HSPD1	qkigieiikrTlkipamtiak
IPI00784154	473	471	PKC->PKA	PKC->PKA		3329	HSPD1	qkigieiikrTlkipamtiak
IPI00784154	523	522	PKC->	PKC->DNAPK		3329	HSPD1	nmvekgiidpTkvvrtallda
IPI00784161	743	740	unsp->unsp+EGFR	unsp->unsp+EGFR		6830	SUPT6H	knkllaeakeYvikacsrkly
IPI00784161	743	746	PKC->PKC+cdc2	PKC->PKC+cdc2		6830	SUPT6H	eakeyvikacSrklynwlrva
IPI00784161	1676	1668	->cdc2			6830	SUPT6H	rqrqqqpksnShaaidwgkma
IPI00784224	509	512	unsp+PKC->	unsp+PKC->PKC		51663	ZFR	infvggnklqStgnkaedikg
IPI00784224	509	513	PKC->	PKC->		51663	ZFR	nfvggnklqsTgnkaedikgt
IPI00784414	49	45	SRC+unsp->	SRC+unsp->SRC+unsp+EGFR	PhosphoSite	6774	STAT3	apwiesqdwaYaaskeshatl
IPI00784414	49	48		cdc2->ATM+DNAPK		6774	STAT3	iesqdwayaaSkeshatlvfh
IPI00784414	49	51	PKA->	PKA->		6774	STAT3	qdwayaaskeShatlvfhnll
IPI00784473	3523	3522		->DNAPK		55187	VPS13D	lpppfridnfSkvpvvftqhg
IPI00784614	431	434	unsp+PKC+PKA->unsp+PKA			10801	9-Sep	pldiefmkrlSkvvnivpvia
IPI00785015	1148	1147		cdc2->ATM+cdc2		254048	UBN2	lpptknlqapSkltnssstgt
IPI00785015	1148	1153	unsp+CKI->CKI	unsp+CKI->		254048	UBN2	lqapskltnsSstgtvgknsl
IPI00785110	880	884	unsp+p38MAPK->p38MAPK	unsp+p38MAPK->p38MAPK		2186	BPTF	rrhlahkfclTpagefkwngs
IPI00785113	568	564	PKC->	PKC->		9208	LRRFIP1	ksdqqgealdSsqkktknkkk
IPI00785113	568	565	ATM+unsp+PKC+DNAPK->ATM+unsp+DNAPK			9208	LRRFIP1	sdqqgealdsSqkktknkkkk
IPI00788907	144	149	unsp+PKC->	unsp+PKC->		5827	PXMP2	glkfnkivhsSmtraiettdi
IPI00788907	191	188	unsp->			5827	PXMP2	gapiepdppvShwkpeavqyy
IPI00788925	44	42	ATM+unsp+PKC+DNAPK->ATM+DNAPK	ATM+unsp+PKC+DNAPK->ATM+DNAPK		587	BCAT2	fkaadlqlemTqkphkkpgpg
IPI00788925	47	42	ATM+unsp+PKC+DNAPK->ATM+unsp+DNAPK			587	BCAT2	fkaadlqlemTqkphkkpgpg

IPI00788925	322	320	unsp->	unsp->		587	BCAT2	gefrvvertiTmkqllralee
IPI00788925	378	382	unsp->			587	BCAT2	rfqkelkeiqYgirahewmfp
IPI00789041	238	237	unsp+PKG->unsp+PKA	unsp+PKG->unsp+DNAPK		5411	PNN	akiikyirtkTkphlfyipgr
IPI00789101	37	41	PKC->			10728	PTGES3	vnvnfeksklTfsclggsdnf
IPI00789101	39	41		PKC->		10728	PTGES3	vnvnfeksklTfsclggsdnf
IPI00789159	108	102	unsp+PKG->unsp+PKG+PKA			6147	RPL23A	lrrqpkyprkSaprrnkldhy
IPI00789159	108	112	unsp->			6147	RPL23A	saprrnkldhYaiikfpltte
IPI00789442	488	490	INSR->	INSR->		7564	ZNF16	hqiihtgekpYrcsvcgkafs
IPI00789442	488	493	PKC+cdc2+PKA->cdc2	PKC+cdc2+PKA->cdc2+PKA		7564	ZNF16	ihtgekpyrcSvcgkafshss
IPI00789551	3	2	cdc2->PKA+cdc2+DNAPK	cdc2->cdc2+DNAPK		9782	MATR3	---------mSksfqqsslsr
IPI00789551	3	9	unsp+DNAPK->unsp+cdc2+DNAPK	unsp+DNAPK->unsp+cdc2+DNAPK		9782	MATR3	--msksfqqsSlsrdsqghgr
IPI00789551	3	11	unsp->unsp+cdc2			9782	MATR3	msksfqqsslSrdsqghgrdl
IPI00789551	473	471	unsp+ATM+PKC->ATM	unsp+ATM+PKC->ATM+DNAPK		9782	MATR3	vfgkpvrvhlSqkykrikkpe
IPI00789551	571	569	unsp+PKC->PKC	unsp+PKC->		9782	MATR3	fqgrcvkvdlSekykklvlri
IPI00789551	877	875	unsp->			9782	MATR3	knthcsslphYqklkkflnkl
IPI00789551	879	875	unsp->			9782	MATR3	knthcsslphYqklkkflnkl
IPI00789798	774	776	PKA->	PKA->		10743	RAI1	tkgleqggkaSdgiskgdthe
IPI00789941	244	243		->ATM		132660	LIN54	prtptsgpviTklifakpins
IPI00789941	249	253	PKC->			132660	LIN54	tklifakpinSkavtgqttqv
IPI00789941	357	356	unsp->	unsp->ATM+unsp+DNAPK		132660	LIN54	pnvqqiqvpgSkfhyvrlvta
IPI00789941	357	360	unsp->			132660	LIN54	qiqvpgskfhYvrlvtatsas
IPI00790342	211	214	PKC->			6128	RPL6	isnvkipkhlTdayfkkkklr
IPI00790342	219	214	PKC->	PKC->		6128	RPL6	isnvkipkhlTdayfkkkklr
IPI00790342	240	241	INSR+unsp->unsp			6128	RPL6	geifdtekekYeiteqrkidq
IPI00790503	1268	1271	unsp+PKC->PKC	unsp+PKC->PKC		4628	MYH10	vkvlqqvkaeSehkrkkldaq
IPI00790503	1366	1368	unsp+PKA+CKII->CKII	unsp+PKA+CKII->unsp+CKII		4628	MYH10	rirqleeeknSlqeqqeeeee
IPI00791574	29	33	unsp+PKC->			7871	SLMAP	yldepikigrSvarcrpaqnn
IPI00792352	57	62	PKC+PKA->	PKC+PKA->		221547	RANP1	tgefekkyvaTlgvevhplvf
IPI00792352	58	62	PKC+PKA->	PKC+PKA->PKC		221547	RANP1	tgefekkyvaTlgvevhplvf
IPI00792352	119	117	unsp+PKC->	unsp+PKC->		221547	RANP1	iimfdvtsrvTyknvpnwhrd
IPI00792352	179	170	->cdc2			221547	RANP1	rkknlqyydiSaksnynfekp
IPI00792352	179	175		->EGFR		221547	RANP1	qyydisaksnYnfekpflwla
IPI00792743	401	392		cdc2->		79811	SLTM	stkniwvsglSsntkaadlkn
IPI00792743	1024	1019	unsp->unsp+PKA	unsp->unsp+PKA	PhosphoSite	79811	SLTM	qisgnsmprgSgsgfkpfkgg
IPI00792743	1024	1021	PKC->	PKC->PKC+PKA	PhosphoSite	79811	SLTM	sgnsmprgsgSgfkpfkggpp
IPI00792984	45	41		CKII->PKG+CKII		10776	ARPP19	nsleemedkvTspekaeeakl
IPI00792984	45	42	unsp+CKI+p38MAPK->unsp+CKI+CKII+p38MAPK	unsp+CKI+p38MAPK->unsp+CKI+CKII+p38MAPK		10776	ARPP19	sleemedkvtSpekaeeaklk
IPI00792984	50	42	unsp+CKI+p38MAPK->unsp+CKI+cdc2+p38MAPK			10776	ARPP19	sleemedkvtSpekaeeaklk
IPI00792984	128	123	unsp+PKG+PKA+RSK->unsp+PKG+PKA	unsp+PKG+PKA+RSK->unsp+PKG+PKA		10776	ARPP19	tpqdlpqrkpSlvasklag--
IPI00792984	128	127		->ATM+DNAPK		10776	ARPP19	lpqrkpslvaSklag------
IPI00793199	215	218	ATM+unsp+DNAPK->ATM+unsp+cdc2+DNAPK	ATM+unsp+DNAPK->ATM+unsp+PKC+cdc2+DNAPK		307	ANXA4	lhvfdeykriSqkdieqsiks
IPI00793199	227	218	ATM+unsp+DNAPK->ATM+unsp+cdc2+DNAPK			307	ANXA4	lhvfdeykriSqkdieqsiks
IPI00793199	227	225	unsp+PKC->	unsp+PKC->		307	ANXA4	krisqkdieqSiksetsgsfe
IPI00793199	227	230	PKC->cdc2	PKC->PKC+cdc2		307	ANXA4	kdieqsikseTsgsfedalla
IPI00793199	227	233	unsp->unsp+cdc2	unsp->unsp+cdc2		307	ANXA4	eqsiksetsgSfedallaivk
IPI00793199	295	298	unsp->	unsp->		307	ANXA4	hfkrlygkslYsfikgdtsgd
IPI00793199	295	299	unsp+PKC+cdc2->CKI+cdc2	unsp+PKC+cdc2->unsp+CKI+cdc2		307	ANXA4	fkrlygkslySfikgdtsgdy
IPI00793199	302	296	->PKA	->PKA		307	ANXA4	rahfkrlygkSlysfikgdts
IPI00793199	302	298	unsp->	unsp->		307	ANXA4	hfkrlygkslYsfikgdtsgd
IPI00793199	302	299	unsp+PKC+cdc2->cdc2	unsp+PKC+cdc2->unsp+CKI+cdc2		307	ANXA4	fkrlygkslySfikgdtsgdy
IPI00793199	302	305	unsp->			307	ANXA4	kslysfikgdTsgdyrkvllv
IPI00793199	302	306	unsp+CKII->CKII	unsp+CKII->unsp+cdc2+CKII		307	ANXA4	slysfikgdtSgdyrkvllvl
IPI00793199	302	309		INSR->		307	ANXA4	sfikgdtsgdYrkvllvlcgg
IPI00793201	57	54	PKC+cdc2->PKA+cdc2	PKC+cdc2->cdc2		9255	AIMP1	qiieylkqqvSllkekailqa
IPI00793375	173	169		->PKG		7511	XPNPEP1	rvgvdpliipTdywkkmakvl
IPI00793375	174	181	->PKA	->PKA		7511	XPNPEP1	ywkkmakvlrSaghhlipvke
IPI00793375	347	355	->INSR			7511	XPNPEP1	ipkdhrccmpYtpiciakavk
IPI00793696	77	86	unsp->unsp+PKA			6152	RPL24	vkfqraitgaSladimakrnq
IPI00794402	186	189	INSR+unsp->INSR			396	ARHGDIA	rkgvkidktdYmvgsygprae
IPI00794402	223	219	unsp+PKC+DNAPK->unsp+PKA+DNAPK	unsp+PKC+DNAPK->unsp+PKC+PKA+DNAPK		396	ARHGDIA	eapkgmlargSysiksrftdd
IPI00794402	223	221	unsp+PKC->	unsp+PKC->		396	ARHGDIA	pkgmlargsySiksrftdddk
IPI00794402	223	224	unsp->	unsp->		396	ARHGDIA	mlargsysikSrftdddktdh
IPI00794402	223	227	unsp+PKC+CKII->unsp+CKII			396	ARHGDIA	rgsysiksrfTdddktdhlsw
IPI00794461	6	5		->DNAPK		8341	HIST1H2BN	------mpepSksapapkkgs
IPI00794461	6	7	unsp->	unsp->		8341	HIST1H2BN	----mpepskSapapkkgskk
IPI00794461	12	7	unsp->	unsp->		8341	HIST1H2BN	----mpepskSapapkkgskk
IPI00794461	12	15	unsp+PKC->PKC	unsp+PKC->PKC	Uniprot	8341	HIST1H2BN	sksapapkkgSkkavtkaqkk
IPI00794461	13	15	unsp+PKC->PKC		Uniprot	8341	HIST1H2BN	sksapapkkgSkkavtkaqkk
IPI00794461	16	15	unsp+PKC->unsp+PKC+PKA	unsp+PKC->ATM+unsp+PKC+DNAPK	Uniprot	8341	HIST1H2BN	sksapapkkgSkkavtkaqkk
IPI00794461	17	15	unsp+PKC->PKC+PKA	unsp+PKC->PKC+PKA	Uniprot	8341	HIST1H2BN	sksapapkkgSkkavtkaqkk
IPI00794461	21	15	unsp+PKC->unsp+PKC+PKA	unsp+PKC->unsp+PKC+PKA	Uniprot	8341	HIST1H2BN	sksapapkkgSkkavtkaqkk
IPI00794461	21	20		PKC->PKC+DNAPK		8341	HIST1H2BN	apkkgskkavTkaqkkdgkkr
IPI00794461	109	113	unsp->			8341	HIST1H2BN	lpgelakhavSegtkavtkyt
IPI00794461	121	120		->ATM+DNAPK		8341	HIST1H2BN	havsegtkavTkytsskkrkr
IPI00795015	943	940	unsp+PKC+DNAPK->DNAPK			26278	SACS	inhssdqgisSytklkgckvl
IPI00795015	943	941	INSR->	INSR->		26278	SACS	nhssdqgissYtklkgckvlh
IPI00795015	945	940	unsp+PKC+DNAPK->PKC+DNAPK	unsp+PKC+DNAPK->PKC+DNAPK		26278	SACS	inhssdqgisSytklkgckvl
IPI00795015	945	953	->cdc2			26278	SACS	klkgckvlhhTaklpadlrls
IPI00795043	354	356	PKG->	PKG->		6720	SREBF1	asaqsrgekrTahnaiekryr
IPI00795043	363	367	unsp+PKG+PKA+RSK->unsp+PKG+PKA			6720	SREBF1	ahnaiekryrSsindkiielk
IPI00795043	363	368		unsp+PKC+PKA->unsp+PKA	PhosphoSite	6720	SREBF1	hnaiekryrsSindkiielkd
IPI00795292	127	122	PKC->	PKC->		4831	NME2	vgrtmanlerTfiaikpdgvq
IPI00795292	164	159	unsp+PKA+RSK->unsp+PKA	unsp+PKA+RSK->unsp+PKA	PHOsphoELM	4831	NME2	rlvamkflraSeehlkqhyid
IPI00795292	164	167	unsp->	unsp->	Uniprot	4831	NME2	raseehlkqhYidlkdrpffp
IPI00795292	215	214		unsp+CKI->unsp+DNAPK		4831	NME2	vmlgetnpadSkpgtirgdfc
IPI00795292	239	235	PKC+cdc2->PKA	PKC+cdc2->		4831	NME2	iqvgrniihgSdsvksaekei
IPI00795292	239	237	unsp+PKC+cdc2->unsp+cdc2	unsp+PKC+cdc2->unsp+cdc2		4831	NME2	vgrniihgsdSvksaekeisl
IPI00795292	243	235		PKC+cdc2->PKC		4831	NME2	iqvgrniihgSdsvksaekei
IPI00795922	138	139	unsp+EGFR->unsp			28957	MRPS28	crrpevdgekYqkgtrvrlrl
IPI00796333	96	93	PKC->			226	ALDOA	gilaadestgSiakrlqsigt
IPI00796333	96	100	PKA+RSK->PKA			226	ALDOA	stgsiakrlqSigtenteenr
IPI00796513	294	285		unsp+PKC+PKG+cdc2+PKA+RSK->unsp+PKC+PKG+PKA+RSK		22807	IKZF2	ltgnmgkrksStpqkfvgekl
IPI00796864	232	239	->PKA			8813	DPM1	vlekliekcvSkgyvfqmemi
IPI00796934	33	29		unsp->unsp+EGFR		387101	RPL29P4	ngikkprsqrYeslkgvdpkf
IPI00796934	33	31	unsp->unsp+cdc2+PKA	unsp->unsp+cdc2+PKA		387101	RPL29P4	ikkprsqryeSlkgvdpkflr
IPI00797279	412	406	ATM+unsp+DNAPK->unsp+DNAPK			29128	UHRF1	kakmasatssSqrdwgkgmac
IPI00797279	559	558	unsp+PKA->PKA	unsp+PKA->unsp+PKA+DNAPK		29128	UHRF1	vvrnvkggknSkyapaegnry
IPI00797574	473	478	PKC->	PKC->		259282	BOD1L	hayvhkpylySkyysdsddel
IPI00797720	208	210	unsp+PKC+cdc2->PKC+cdc2			5922	RASA2	vgpsrndqkkTkvkkktsnpq
IPI00797720	211	210	unsp+PKC+cdc2->PKC+cdc2	unsp+PKC+cdc2->PKC+cdc2		5922	RASA2	vgpsrndqkkTkvkkktsnpq
IPI00797720	213	210	unsp+PKC+cdc2->PKC+cdc2	unsp+PKC+cdc2->PKC+cdc2		5922	RASA2	vgpsrndqkkTkvkkktsnpq
IPI00797720	213	217	PKC+PKG+PKA->	PKC+PKG+PKA->		5922	RASA2	qkktkvkkktSnpqfneifyf
IPI00797945	399	393	ATM+unsp+DNAPK->unsp+DNAPK			29128	UHRF1	kakmasatssSqrdwgkgmac
IPI00797945	546	545	unsp+PKA->PKA	unsp+PKA->unsp+PKA+DNAPK		29128	UHRF1	vvrnvkggknSkyapaegnry
IPI00798011	11	12		CKII->unsp+CKII		6612	SUMO3	seekpkegvkTendhinlkva
IPI00798155	6	7		PKC->unsp+PKC		7316	UBC	----mqifvkTltgktitlev
IPI00798155	6	12	->PKA			7316	UBC	qifvktltgkTitlevepsdt
IPI00807573	530	533	unsp->	unsp->		5295	PIK3R1	rimhnydklkSriseiidsrr
IPI00807573	530	536	unsp->unsp+cdc2			5295	PIK3R1	hnydklksriSeiidsrrrle
IPI00807625	66	65	SRC+unsp->SRC+unsp+EGFR	SRC+unsp->SRC+unsp+EGFR		23048	FNBP1	pkknskeeeeYkytsckafis
IPI00807625	66	68	unsp+cdc2->cdc2			23048	FNBP1	nskeeeeykyTsckafisnln
IPI00807625	66	69	unsp+PKC->unsp+PKC+cdc2	unsp+PKC->unsp+PKC+cdc2		23048	FNBP1	skeeeeykytSckafisnlne
IPI00807625	110	115	unsp+PKA->unsp	unsp+PKA->unsp		23048	FNBP1	yvqelkqerkSnfhdgrkaqq
IPI00807625	132	136	unsp+PKC->PKC			23048	FNBP1	hietcwkqleSskrrferdck
IPI00807625	132	137	unsp+PKC->unsp+PKC+cdc2	unsp+PKC->unsp+PKC+cdc2		23048	FNBP1	ietcwkqlesSkrrferdcke
IPI00815642	30	26	PKC->	PKC->		7114	TMSB4X	scaslrfssaTmsdkpdmaei
IPI00815642	30	28	unsp+PKC->CKII	unsp+PKC->		7114	TMSB4X	aslrfssatmSdkpdmaeiek
IPI00815642	52	49	PKC+DNAPK->DNAPK			7114	TMSB4X	fdksklkkteTqeknplpske
IPI00815642	58	57		unsp->ATM+unsp+DNAPK		7114	TMSB4X	tetqeknplpSketieqekqa
IPI00815642	58	60		->unsp		7114	TMSB4X	qeknplpskeTieqekqages
IPI00815707	1685	1679	unsp+cdk5->unsp	unsp+cdk5->unsp	Uniprot|PhosphoSite	404734	ANKHD1-EI	sspniklnltSpkrgqkreeg
IPI00815713	155	153	unsp+PKC->	unsp+PKC->	Uniprot|PhosphoSite	6949	TCOF1	atgktvanllSgksprksaep
IPI00815713	155	156	unsp+cdk5+p38MAPK->unsp+p38MAPK		Uniprot|PhosphoSite	6949	TCOF1	ktvanllsgkSprksaepsan
IPI00815713	155	160	unsp+PKG+PKA->unsp+PKG	unsp+PKG+PKA->unsp+PKG	Uniprot	6949	TCOF1	nllsgksprkSaepsanttlv
IPI00815713	296	289	ATM+DNAPK->ATM+cdc2+DNAPK			6949	TCOF1	seeeapagtrSqvkasekilq
IPI00815713	296	294	unsp->	unsp->		6949	TCOF1	pagtrsqvkaSekilqvraas
IPI00815713	755	750	PKC->PKC+PKA			6949	TCOF1	apvlpgktgpTvtqvkaekqe
IPI00815713	755	752	unsp->			6949	TCOF1	vlpgktgptvTqvkaekqeds

IPI00815713	1451	1447	ATM+CKI+PKC+DNAPK->unsp+ATM+CKI+DNAPK	ATM+CKI+PKC+DNAPK->unsp+ATM+CKI+PKC+DNAP	Uniprot|PhosphoSite	6949	TCOF1	kekkgkgslgSqgakdepeee
IPI00816288	32	31		unsp->ATM+unsp+DNAPK		7115	TMSL1	tetqeknplpSketieqekqa
IPI00816288	32	34		->unsp		7115	TMSL1	qeknplpskeTieqekqages
IPI00827535	15	10	unsp+PKC+CKI->unsp+CKI	unsp+PKC+CKI->	Uniprot	150928	PTMAP5	-msdaavdtsSeittkdlkek
IPI00827535	15	13	unsp+CKI+PKC->CKI+CKII	unsp+CKI+PKC->CKI	Uniprot|PHOsphoELM	150928	PTMAP5	daavdtsseiTtkdlkekkev
IPI00827535	15	14		unsp+CKII->unsp+CKII+DNAPK	Uniprot|PHOsphoELM	150928	PTMAP5	aavdtsseitTkdlkekkevv
IPI00827535	18	13	unsp+CKI+PKC->unsp+CKI	unsp+CKI+PKC->unsp	Uniprot|PHOsphoELM	150928	PTMAP5	daavdtsseiTtkdlkekkev
IPI00827535	18	14		unsp+CKII->CKII	Uniprot|PHOsphoELM	150928	PTMAP5	aavdtsseitTkdlkekkevv
IPI00827535	103	102	unsp+PKC+CKII->unsp+PKC		PhosphoSite	150928	PTMAP5	aeddedddvdTkkqktdedd-
IPI00827535	104	102	unsp+PKC+CKII->PKC+CKII	unsp+PKC+CKII->PKC+CKII	PhosphoSite	150928	PTMAP5	aeddedddvdTkkqktdedd-
IPI00827930	421	429	unsp+PKC->unsp+PKC+cdc2			125488	TTC39C	krknnqieqfSvkkaerfrkq
IPI00828125	507	505		unsp->unsp+cdk5		55627	SMPD4	lnralrtdlvSpkhalmvfrv
IPI00829826	15	10	unsp+CKI+cdc2+CKII->unsp+cdc2+CKII	unsp+CKI+cdc2+CKII->unsp+cdc2+CKII		5469	MED1	-mkaqgeteeSeklskmssll
IPI00829826	15	14		unsp+PKA->ATM+unsp		5469	MED1	qgeteeseklSkmssllerlh
IPI00829826	15	17	PKA->	PKA->		5469	MED1	teeseklskmSsllerlhakf
IPI00829826	1076	1071	PKC+cdc2->cdc2			5469	MED1	pkitiqipkgTvmvgkpsshs
IPI00829826	1076	1078	unsp+PKG->	unsp+PKG->unsp		5469	MED1	pkgtvmvgkpSshsqytssgs
IPI00829826	1076	1079	unsp+PKC->	unsp+PKC->PKC		5469	MED1	kgtvmvgkpsShsqytssgsv
IPI00829826	1076	1081	ATM+unsp+DNAPK->ATM+unsp+cdc2+DNAPK	ATM+unsp+DNAPK->ATM+unsp+cdc2+DNAPK		5469	MED1	tvmvgkpsshSqytssgsvss
IPI00829826	1177	1179	unsp+PKG->	unsp+PKG->		5469	MED1	stkmkpqgkpSslmnpslskp
IPI00829826	1311	1316	unsp+PKC->unsp	unsp+PKC->unsp		5469	MED1	vidklkhgvvTsgpggedpld
IPI00829826	1354	1358	unsp+RSK->unsp	unsp+RSK->unsp		5469	MED1	ggefqgkrekSdkdkskvsts
IPI00829826	1529	1525	unsp+PKC+RSK->unsp+PKA+RSK	unsp+PKC+RSK->unsp+RSK		5469	MED1	drdkdrdkkkShsikpeswsk
IPI00829826	1529	1527	unsp+PKC+cdc2->unsp+cdc2	unsp+PKC+cdc2->unsp+cdc2		5469	MED1	dkdrdkkkshSikpeswsksp
IPI00829826	1529	1532	unsp+PKC->unsp+PKC+GSK3			5469	MED1	kkkshsikpeSwskspissdq
IPI00843975	139	144	unsp+PKA->	unsp+PKA->		7430	EZR	fgdynkevhkSgylsserlip
IPI00844000	571	576	PKA->CKI			23376	KIAA0776	qaaltkhllkSvctditnlif
IPI00844214	91	93	unsp+PKC+PKA->unsp+PKC	unsp+PKC+PKA->unsp		56259	CTNNBL1	kkmiltfekrSyknqelrikf
IPI00844214	91	94	INSR+unsp->	INSR+unsp->		56259	CTNNBL1	kmiltfekrsYknqelrikfp
IPI00844264	127	125	unsp+PKC+PKG->	unsp+PKC+PKG->		7812	CSDE1	vefevssdrrTgkpiavklvk
IPI00844508	579	573		PKC->PKC+cdc2		57530	CGN	etseetghwqSmfqknkedlr
IPI00844508	579	585	unsp->unsp+PKA			57530	CGN	fqknkedlraTkqellqlrme
IPI00844578	1024	1025	->cdc2	->cdc2		1660	DHX9	tegrnalihkSsvncpfssqd
IPI00844578	1024	1026	cdc2->PKC+cdc2			1660	DHX9	egrnalihksSvncpfssqdm
IPI00845227	185	181	PKC->	PKC->		128061	C1orf131	arlevhrfgiTgygkgkeril
IPI00845355	967	962	unsp+PKC+CKII->CKII	unsp+PKC+CKII->CKII		546	ATRX	ktckkvqdglSdiaekflkkd
IPI00845355	1930	1921	unsp+DNAPK->unsp+cdc2+DNAPK			546	ATRX	iasdsdetsmSlssddytkkk
IPI00845355	1930	1924		unsp+PKC+CKII->unsp+PKC	PhosphoSite	546	ATRX	dsdetsmslsSddytkkkkkg
IPI00845355	1930	1927		INSR+unsp->INSR+unsp+EGFR		546	ATRX	etsmslssddYtkkkkkgkkg
IPI00845355	1930	1928	unsp+PKC->PKC	unsp+PKC->PKC	PhosphoSite	546	ATRX	tsmslssddyTkkkkkgkkgk
IPI00845355	1936	1941	unsp+cdc2+PKA->unsp+cdc2	unsp+cdc2+PKA->unsp+cdc2		546	ATRX	kkkgkkgkkdSsssgsgsdnd
IPI00845355	1936	1943	unsp->unsp+cdc2			546	ATRX	kgkkgkkdssSsgsgsdndve
IPI00845355	1939	1941	unsp+cdc2+PKA->unsp+cdc2	unsp+cdc2+PKA->unsp+cdc2		546	ATRX	kkkgkkgkkdSsssgsgsdnd
IPI00845479	471	476	unsp->cdc2	unsp->cdc2		10659	CUGBP2	kvfidkqtnlSkcfgfvsydn
IPI00847436	196	190	unsp->unsp+PKA			10765	KDM5B	npynlflsgdSlrclqkpnlt
IPI00848226	130	128	unsp->PKA	unsp->PKA		10399	GNB2L1	rqivsgsrdkTiklwntlgvc
IPI00848226	130	134	PKC+DNAPK->DNAPK	PKC+DNAPK->DNAPK		10399	GNB2L1	srdktiklwnTlgvckytvqd
IPI00852603	1053	1047	->PKA	->PKA		164045	HFM1	dnqvvylhkiTdsvllkagsw
IPI00852603	1059	1056	PKC+cdc2->cdc2			164045	HFM1	itdsvllkagSwakkiavkra
IPI00852685	1057	1048		cdc2->		1729	DIAPH1	pdelahvekaSrvsaenlqkn
IPI00852685	1103	1101		->unsp		1729	DIAPH1	tsfvkdaqeqYnklrmmhsnm
IPI00853009	462	467	unsp->cdc2	unsp->cdc2		10658	CUGBP1	kvfidkqtnlSkcfgfvsydn
IPI00853077	738	736	unsp+PKC+PKG+PKA->PKG+PKA	unsp+PKC+PKG+PKA->PKG+PKA		51105	PHF20L1	icrdppgqrwSakyrydkewl
IPI00853077	738	741	INSR->	INSR->		51105	PHF20L1	pgqrwsakyrYdkewlnngrm
IPI00853077	910	908	unsp->CKII	unsp->CKII		51105	PHF20L1	hmrsknslqySakehgmpekn
IPI00853240	266	265	PKC->	PKC->DNAPK		83860	TAF3	sqmptakpleTksftpktktk
IPI00853240	266	269		CKI->		83860	TAF3	takpletksfTpktktktssp
IPI00853240	776	775		->ATM		83860	TAF3	vgagqdkiviSkvvpapeakp
IPI00853400	92	85	INSR->	INSR->		23307	FKBP15	lvatavhayrYtngqyvkqgk
IPI00853400	92	86	->PKA			23307	FKBP15	vatavhayryTngqyvkqgkf
IPI00854642	974	977	unsp->	unsp->		23244	PDS5A	harqcllkniSirreyikqnp
IPI00854642	1146	1149	unsp->	unsp->		23244	PDS5A	gvlgavnkplSatgrkpyvrs
IPI00854642	1211	1208	cdk5->	cdk5->	iniprot|PHOsphoELM|PhosphoSi	23244	PDS5A	eenpvriisvTpvknidpvkn
IPI00855767	422	414		cdc2->		5910	RAP1GDS1	vteavlkflkSemppvqfkll
IPI00855767	422	426	PKC->			5910	RAP1GDS1	mppvqfkllgTlrmlidaqae
IPI00855856	251	250		PKC->ATM+PKC+DNAPK		8539	API5	qctrqavplfSknvhstrfvt
IPI00855856	251	255	unsp+PKC->			8539	API5	avplfsknvhStrfvtyfceq
IPI00855924	68	65	PKC->PKC+CKII	PKC->PKC+CKII		143244	EIF5AL1	kvhlvgidifTgkkyedicps
IPI00855980	91	94	EGFR->	EGFR->		4833	NME4	hyqdlrrkpfYpalirymssg
IPI00855998	1673	1668	ATM+CKI+DNAPK->ATM+DNAPK	ATM+CKI+DNAPK->ATM+DNAPK		1063	CENPF	slekleekmeSqgimknkeiq
IPI00855998	2757	2756	unsp+PKC+cdc2+PKA->cdc2+PKA	unsp+PKC+cdc2+PKA->unsp+cdc2+DNAPK		1063	CENPF	lqllleeiksSkdqlkeltle
IPI00856049	70	68	unsp+PKC->	unsp+PKC->		200916	RPL22L1	erfknkitvvSekqfskrylk
IPI00856115	90	81		ATM+unsp+cdc2+DNAPK->ATM+unsp+DNAPK		29803	REPIN1	laqprllsgpSqespqtlgke
IPI00856115	90	87	unsp+PKC->unsp	unsp+PKC->unsp		29803	REPIN1	lsgpsqespqTlgkesrglrq
IPI00856115	90	92	PKA->	PKA->		29803	REPIN1	qespqtlgkeSrglrqqgtsv
IPI00856115	333	331	unsp+PKC+PKG+PKA->cdc2+PKA	unsp+PKC+PKG+PKA->PKA		29803	REPIN1	hqcpecgkrfTnkpyltshrr
IPI00856115	333	337	unsp+PKC->	unsp+PKC->PKC		29803	REPIN1	gkrftnkpylTshrrihtgek
IPI00869087	21	19	PKC->	PKC->		220832	FABP5L3	ciitcdsknlTiktestlktt
IPI00869087	21	24	unsp+PKC->PKC	unsp+PKC->PKC		220832	FABP5L3	dsknltikteStlkttqfsgt
IPI00869087	21	25	unsp+PKC->unsp+CKI	unsp+PKC->unsp+CKI+PKC		220832	FABP5L3	sknltiktesTlkttqfsgtl
IPI00871134	28	27	->PKA					qcektvdvkkSkfceadvssd
IPI00871134	28	36	CKII->cdc2+CKII					kskfceadvsSdlrkevenhy
IPI00871535	1519	1517		->unsp		6709	SPTAN1	fadqliaaghYakgdissrrn
IPI00871535	1519	1523	unsp+PKC->unsp			6709	SPTAN1	aaghyakgdiSsrrnevldrw
IPI00871535	1519	1524	unsp+PKC->PKC	unsp+PKC->		6709	SPTAN1	aghyakgdisSrrnevldrwr
IPI00871535	2057	2056	->PKA	->DNAPK		6709	SPTAN1	dqllaakhvqSkaiearhasl
IPI00871535	2426	2423	PKC->			6709	SPTAN1	eiesafralsSegkpyvtkee
IPI00871535	2431	2428		unsp->	Uniprot|PhosphoSite	6709	SPTAN1	fralssegkpYvtkeelyqnl
IPI00871535	2431	2430		unsp+CKII->ATM+unsp+CKII		6709	SPTAN1	alssegkpyvTkeelyqnltr
IPI00871535	2431	2435	unsp+EGFR->unsp		PhosphoSite	6709	SPTAN1	gkpyvtkeelYqnltreqady
IPI00871535	2431	2439	->cdc2			6709	SPTAN1	vtkeelyqnlTreqadycvsh
IPI00871539	405	403	PKC->	PKC->		7226	TRPM2	tftesrivewTkkiqdivrrr
IPI00871539	1104	1101	PKC+GSK3+cdk5+p38MAPK->GSK3+cdk5+p38MAPK	PKC+GSK3+cdk5+p38MAPK->GSK3+cdk5+p38MAPK		7226	TRPM2	qlfikrvvlkTpakrhkqlkn
IPI00871851	225	223	unsp+PKC+CKII->CKII	unsp+PKC+CKII->CKII		4735	2-Sep	vpviakadtlTkererlkkr
IPI00871870	56	58		->unsp		10094	ARPC3	yfkanvffknYeikneadrtl
IPI00872359	235	238	unsp+PKC->PKC	unsp+PKC->PKC		1639	DCTN1	qvrdleekleTlrlkraedka
IPI00872762	54	46	->PKA			8802	SUCLG1	ngirhcsytaSrqhlyvdknt
IPI00872762	54	56		PKC->		8802	SUCLG1	srqhlyvdknTkiicqgftgk
IPI00872780	213	216	ATM+unsp+DNAPK->ATM+unsp+cdc2+DNAPK	ATM+unsp+DNAPK->ATM+unsp+PKC+cdc2+DNAPK		307	ANXA4	lhvfdeykriSqkdieqsiks
IPI00872780	225	216	ATM+unsp+DNAPK->ATM+unsp+cdc2+DNAPK			307	ANXA4	lhvfdeykriSqkdieqsiks
IPI00872780	225	223	unsp+PKC->	unsp+PKC->		307	ANXA4	krisqkdieqSiksetsgsfe
IPI00872780	225	228	PKC->cdc2	PKC->PKC+cdc2		307	ANXA4	kdieqsikseTsgsfedalla
IPI00872780	225	231	unsp->unsp+cdc2	unsp->unsp+cdc2		307	ANXA4	eqsiksetsgSfedallaivk
IPI00872780	293	296	unsp->	unsp->		307	ANXA4	hfkrlygkslYsfikgdtsgd
IPI00872780	293	297	unsp+PKC+cdc2->CKI+cdc2	unsp+PKC+cdc2->unsp+CKI+cdc2		307	ANXA4	fkrlygkslySfikgdtsgdy
IPI00872780	300	294	->PKA	->PKA		307	ANXA4	rahfkrlygkSlysfikgdts
IPI00872780	300	296	unsp->	unsp->		307	ANXA4	hfkrlygkslYsfikgdtsgd
IPI00872780	300	297	unsp+PKC+cdc2->cdc2	unsp+PKC+cdc2->unsp+CKI+cdc2		307	ANXA4	fkrlygkslySfikgdtsgdy
IPI00872780	300	303	unsp->			307	ANXA4	kslysfikgdTsgdyrkvllv
IPI00872780	300	304	unsp+CKII->CKII	unsp+CKII->unsp+cdc2+CKII		307	ANXA4	slysfikgdtSgdyrkvllvl
IPI00872780	300	307		INSR->		307	ANXA4	sfikgdtsgdYrkvllvlcgg
IPI00872874	51	52	unsp->	unsp->		6303	SAT1	ilrlikelakYeymeeqvilt
IPI00872874	51	54	unsp+EGFR->	unsp+EGFR->		6303	SAT1	rlikelakyeYmeeqviltek
IPI00873029	681	674	unsp+PKA->unsp+cdc2+PKA			4299	AFF1	ppssekkkhkSslpapskals
IPI00873029	681	675	unsp+PKC+PKA->unsp+PKC+cdc2+PKA	unsp+PKC+PKA->unsp+PKC+cdc2+PKA		4299	AFF1	pssekkkhksSlpapskalsg
IPI00873029	681	680		->DNAPK		4299	AFF1	kkhksslpapSkalsgpepak
IPI00873029	681	684	unsp->unsp+CKII	unsp->unsp+CKII		4299	AFF1	sslpapskalSgpepakdnve
IPI00873029	809	804	unsp+PKC->unsp+PKA	unsp+PKC->unsp		4299	AFF1	gkkhssekrsSdsssklakkr
IPI00873029	809	808		unsp+PKC->unsp+PKC+DNAPK		4299	AFF1	ssekrssdssSklakkrkgea
IPI00873518	61	60	->cdc2+PKA+DNAPK	->ATM+DNAPK		8379	MAD1L1	qleeraeqirSkshliqvere
IPI00873518	61	62	unsp+PKA+RSK->PKA+RSK	unsp+PKA+RSK->PKA+RSK		8379	MAD1L1	eeraeqirskShliqverekm
IPI00873622	81	79	unsp+PKC->	unsp+PKC->		9948	WDR1	sgfyiasgdvSgklriwdttq
IPI00873622	115	114		->DNAPK		9948	WDR1	kikdiawtedSkriavvgegr
IPI00873622	481	479	unsp+PKC->	unsp+PKC->		9948	WDR1	nvrlysilgtTlkdegkllea
IPI00873622	512	511		->ATM+DNAPK		9948	WDR1	dgaflavcdaSkvvtvfsvad
IPI00873622	512	515	PKC->			9948	WDR1	lavcdaskvvTvfsvadgyse
IPI00873703	1462	1465	unsp+PKC->cdc2	unsp+PKC->PKC+cdc2		29994	BAZ2B	ntnlflqkpgSfsklskllev
IPI00873703	1462	1470	PKA->cdc2+PKA			29994	BAZ2B	lqkpgsfsklSkllevakmpp

Table S3

IPI00874020	125	129	PKC->			84105	PCBD2	lakfiekaaaSv---------
IPI00876931	47	39	unsp+PKC->unsp+PKC+cdc2			26173	INTS1	algskgqaneSktastllkpa
IPI00876931	47	43	PKC->PKA	PKC->PKA		26173	INTS1	kgqanesktaStllkpapsgl
IPI00876931	47	44	PKC+cdc2->cdc2	PKC+cdc2->cdc2		26173	INTS1	gqanesktasTllkpapsglp
IPI00878183	452	451		unsp+PKC->unsp+PKC+DNAPK		388567	ZNF749	qhekihtdafSkrsdliqhkr
IPI00878183	452	454	PKG->	PKG->		388567	ZNF749	kihtdafskrSdliqhkridl
IPI00878910	32	24	unsp->unsp+cdc2			23338	PHF15	nsdttdshatStsasrcsklp
IPI00878910	32	28	unsp+PKC->unsp	unsp+PKC->unsp		23338	PHF15	tdshatstsaSrcsklpsstk
IPI00878910	32	31	unsp->unsp+PKA	unsp->ATM+unsp+DNAPK		23338	PHF15	hatstsasrcSklpsstksgw
IPI00878910	32	36	unsp+PKC->unsp+cdc2	unsp+PKC->unsp+PKC+cdc2		23338	PHF15	sasrcsklpsStksgwprqne
IPI00878910	38	35	PKC->			23338	PHF15	tsasrcsklpSstksgwprqn
IPI00878910	38	36	unsp+PKC->cdc2	unsp+PKC->unsp		23338	PHF15	sasrcsklpsStksgwprqne
IPI00878910	38	37		->DNAPK		23338	PHF15	asrcsklpssTksgwprqnek
IPI00878910	298	297		->ATM		23338	PHF15	igcpekmepiTkishipasrw
IPI00878910	298	305	PKC->PKC+PKA			23338	PHF15	pitkishipaSrwalscslck
IPI00878931	150	149	unsp+PKG->unsp+PKG+PKA	unsp+PKG->unsp+PKG+DNAPK		85403	EAF1	ppppmpfrapTkppvgpktsp
IPI00879588	891	887		->EGFR	PhosphoSite	63967	CLSPN	flnvrnhrnqYqalkprlpla
IPI00879588	925	920	PKC->	PKC->		63967	CLSPN	elldlctgkfTsqaekhlprk
IPI00879588	925	921	ATM+unsp+DNAPK->ATM+unsp+PKA+DNAPK			63967	CLSPN	lldlctgkftSqaekhlprks
IPI00879588	925	931	unsp->unsp+PKA			63967	CLSPN	sqaekhlprkSdkkenmeell
IPI00879819	27	22	unsp+PKC+PKA->unsp+PKA	unsp+PKC+PKA->unsp+PKA		84823	LMNB2	patplsptrlSrlqekeelre
IPI00879819	61	59	unsp+CKII->cdc2+CKII	unsp+CKII->CKII		84823	LMNB2	lendrlllkiSekeevttrev
IPI00879819	500	498		->p38MAPK	Uniprot	84823	LMNB2	legeeiaykfTpkyilragqm
IPI00880048	252	248	unsp+PKC->unsp+PKA	unsp+PKC->		129563	DIS3L2	ilekkhsraaTgflklladkn
IPI00880048	252	259	->cdc2			129563	DIS3L2	gflklladknSelfrkyalfs
IPI00883857	352	351		->ATM		3192	HNRNPU	tekipvrhlyTkdidihevri
IPI00883857	609	613	unsp+EGFR->unsp			3192	HNRNPU	avvvcpkdedYkqrtqkkaev
IPI00883857	814	817	ATM+unsp+DNAPK->ATM+DNAPK	ATM+unsp+DNAPK->ATM+DNAPK		3192	HNRNPU	qgqfwgqkpwSqhyhqgyy--
IPI00884896	243	247	unsp+PKC->unsp			7296	TXNRD1	srnygwkveeTvkhdwdrmie
IPI00885078	536	533	PKC->			201175	SH3D20	swtvleggvlTffkdsktsaa
IPI00885078	536	538	unsp+PKG+PKA->unsp	unsp+PKG+PKA->unsp		201175	SH3D20	eggvltffkdSktsaagglrq
IPI00885078	536	540	PKC->			201175	SH3D20	gvltffkdskTsaagglrqps
IPI00885078	536	541	unsp->	unsp->		201175	SH3D20	vltffkdsktSaagglrqpsk
IPI00885104	163	164	unsp->unsp+CKI+p38MAPK		Uniprot|PHOsphoELM|PhosphoSi	54737	MPHOSPH8	kkklrqreekSpddlkkkkak
IPI00885104	177	182	unsp+PKA->unsp			54737	MPHOSPH8	kakagklkdkSkpdlessles
IPI00888429	527	518	unsp+PKC->unsp+PKC+cdc2			283008	FAM22E	qhsraapsrgTarldsssskf
IPI00888429	527	523	unsp+PKG+cdc2+PKA->unsp+PKA+RSK	unsp+PKG+cdc2+PKA->unsp+PKG+cdc2+PKA+RSK		283008	FAM22E	apsrgtarldSssskfaagqg
IPI00888429	527	525	unsp+PKC->	unsp+PKC->		283008	FAM22E	srgtarldssSskfaagqgae
IPI00888429	527	526	PKC->CKI+PKC	PKC->ATM+DNAPK		283008	FAM22E	rgtarldsssSkfaagqgaer
IPI00890837	1349	1350	unsp->unsp+CKII	unsp->CKII		23347	SMCHD1	tlqvkaiynkSiiegpiiklm
IPI00893197	112	115	PKC->			5524	PPP2R4	lnegvkgkklTfeyrvseaie
IPI00895801	305	304		->DNAPK		34	ACADM	glaqraldeaTkyalerktfg
IPI00895801	305	306	unsp->	unsp->		34	ACADM	aqraldeatkYalerktfgkl
IPI00902533	317	314	PKC->	PKC->	Uniprot	9883	POM121	pdpcaketvlSalkekekkrt
IPI00902533	319	314	PKC->	PKC->	Uniprot	9883	POM121	pdpcaketvlSalkekekkrt
IPI00902560	54	59		PKC->		7417	VDAC2	gfglvkldvkTkscsgvefst
IPI00902560	54	61	unsp->unsp+cdc2			7417	VDAC2	glvkldvktkScsgvefstsg
IPI00902560	87	86		CKI+PKC->CKI+PKC+DNAPK		7417	VDAC2	dtgkvtgtleTkykwceyglt
IPI00902560	87	88	unsp->SRC			7417	VDAC2	gkvtgtletkYkwceygltft
IPI00902560	89	83	CKI+DNAPK->DNAPK	CKI+DNAPK->DNAPK		7417	VDAC2	sntdtgkvtgTletkykwcey
IPI00902560	89	86	CKI+PKC->CKI			7417	VDAC2	dtgkvtgtleTkykwceyglt
IPI00902560	135	130	unsp+CKI+p38MAPK->CKI+p38MAPK	unsp+CKI+p38MAPK->CKI+p38MAPK	niprot|PHOsphoELM|PhosphoSi	7417	VDAC2	glkltfdttfSpntgkksgki
IPI00902560	135	133	unsp+CKI+PKC->CKI+PKC	unsp+CKI+PKC->CKI+PKC		7417	VDAC2	ltfdttfspnTgkksgkikss
IPI00902560	135	137	unsp+PKC->PKC			7417	VDAC2	ttfspntgkkSgkikssykre
IPI00902560	135	142	PKC->PKC+cdc2			7417	VDAC2	ntgkksgkikSsykrecinlg
IPI00908586	241	237	unsp->unsp+PKA	unsp->unsp+PKA		22902	RUFY3	lrlalmqkklSeymkalinkk
IPI00908586	241	239	INSR->	INSR->		22902	RUFY3	lalmqkklseYmkalinkkel
IPI00909140	58	51	INSR->	INSR->	PhosphoSite	203068	TUBB	dlqldrisvyYneatggkyvp
IPI00909140	58	59	unsp->	unsp->		203068	TUBB	vyyneatggkYvprailvdle
IPI00909140	103	106	unsp->		PhosphoSite	203068	TUBB	gagnnwakghYtegaelvdsv
IPI00909140	103	107	->CKII			203068	TUBB	agnnwakghyTegaelvdsvl
IPI00909140	379	382	unsp+PKA->PKA	unsp+PKA->PKA	PhosphoSite	203068	TUBB	taiqelfkriSeqftamfrrk
IPI00910610	7	3	PKC->			140739	UBE2F	--------mlTlasklkrddg
IPI00914566	123	117	INSR->	INSR->		2224	FDPS	aiarlkevleYnaiggkynrg
IPI00914566	353	349	unsp->			2224	FDPS	qclqratpeqYqilkenygqk
IPI00914930	720	718		unsp+PKC->unsp		29123	ANKRD11	ekewlfkdekSlkrikdtnkd
IPI00914930	720	725		unsp+PKC->unsp+PKC+cdc2		29123	ANKRD11	dekslkrikdTnkdisrsfre
IPI00915324	118	123	unsp->unsp+CKI			7168	TPM1	leeaekaadeSergmkviesr
IPI00915334	1168	1161	unsp+cdk5->unsp+cdk5+p38MAPK			130507	UBR3	riieeicrkvTppvppkkvta
IPI00915334	1168	1170		PKC+PKG->PKG		130507	UBR3	vtppvppkkvTaaekktldke
IPI00915334	1174	1170	PKC+PKG->PKG	PKC+PKG->PKG		130507	UBR3	vtppvppkkvTaaekktldke
IPI00915334	1174	1176		CKII->unsp+CKII		130507	UBR3	pkkvtaaekkTldkeerrqka
IPI00915400	263	262		CKI->ATM+CKI+DNAPK		9991	ROD1	nacctlridfSkltslnvkyn
IPI00915400	263	265		PKC->		9991	ROD1	ctlridfsklTslnvkynndk
IPI00915400	270	265	PKC->	PKC->		9991	ROD1	ctlridfsklTslnvkynndk
IPI00915400	270	266	PKC+cdc2->	PKC+cdc2->cdc2		9991	ROD1	tlridfskltSlnvkynndks
IPI00915400	429	434	PKC+cdc2+PKA->PKC+cdc2	PKC+cdc2+PKA->PKC+cdc2		9991	ROD1	qrlygkvlraTlskhqavqlp
IPI00915400	429	436	PKC->PKC+cdc2			9991	ROD1	lygkvlratlSkhqavqlpre
IPI00915400	555	552	unsp+PKC+PKA->PKC+PKA			9991	ROD1	dlgenhhlrvSfsksti----
IPI00915400	555	554		->ATM+DNAPK		9991	ROD1	genhhlrvsfSksti------
IPI00915400	555	556	->PKC	->PKC		9991	ROD1	nhhlrvsfskSti--------
IPI00916111	136	129	ATM->	ATM->		4190	MDH1	llkanvkifkSqgaaldkyak
IPI00916111	136	137	unsp->		PhosphoSite	4190	MDH1	fksqgaaldkYakksvkvivv
IPI00916111	316	314	PKC->	PKC->		4190	MDH1	ysfpvviknkTwkfveglpin
IPI00916144	267	271	PKC->			8497	PPFIA4	hkgakrkgikSsigrlfgkke
IPI00916144	267	272	unsp+PKA->PKA	unsp+PKA->PKA		8497	PPFIA4	kgakrkgiksSigrlfgkkek
IPI00916144	270	272	unsp+PKA->PKC	unsp+PKA->unsp		8497	PPFIA4	kgakrkgiksSigrlfgkkek
IPI00916332	758	753	unsp+cdc2->cdc2	unsp+cdc2->cdc2		58508	MLL3	ptiegcvkdvSyqggksikls
IPI00916332	758	764	unsp+CKII->unsp+cdc2+CKII	unsp+CKII->unsp+cdc2+CKII		58508	MLL3	yqggksiklsSetessfsssa
IPI00916332	827	831		unsp+PKC->unsp+PKC+cdc2		58508	MLL3	pkigmgkpaiTkrkfspgrpr
IPI00916332	1772	1770	unsp+PKC->	unsp+PKC->DNAPK		58508	MLL3	kskqqakieaTqkleqvkneq
IPI00916332	2009	2004		unsp+cdc2->cdc2		58508	MLL3	takgpiaagtSdhftkpspra
IPI00916332	2009	2008	PKC->	PKC->		58508	MLL3	piaagtsdhfTkpspradvfq
IPI00916332	2009	2011	unsp+p38MAPK->p38MAPK	unsp+p38MAPK->unsp		58508	MLL3	agtsdhftkpSpradvfqrqr
IPI00916332	2809	2807	unsp+PKC->	unsp+PKC->		58508	MLL3	keqenktlvlSdkhspqkkst
IPI00916332	2809	2811	unsp+GSK3+cdk5->GSK3+cdk5			58508	MLL3	nktlvlsdkhSpqkkstvtne
IPI00916332	2814	2811	unsp+GSK3+cdk5->GSK3	unsp+GSK3+cdk5->unsp+GSK3		58508	MLL3	nktlvlsdkhSpqkkstvtne
IPI00916332	2814	2816	unsp->			58508	MLL3	lsdkhspqkkStvtnevktev
IPI00916332	2832	2828	unsp+GSK3+p38MAPK->unsp+GSK3+cdk5+p38MAPK			58508	MLL3	vtnevktevlSpnskveskce
IPI00916332	2832	2831		unsp->unsp+DNAPK		58508	MLL3	evktevlspnSkveskcetek
IPI00916332	3714	3708	unsp+CKI->unsp	unsp+CKI->unsp		58508	MLL3	tyansevdklSmetpakteei
IPI00916332	3714	3715	CKI->CKI+CKII	CKI->CKI+CKII		58508	MLL3	dklsmetpakTeeiklekaet
IPI00921422	48	43	unsp+GSK3->unsp+GSK3+p38MAPK	unsp+GSK3->unsp+GSK3+p38MAPK		9793	CKAP5	kifqkikdekSpewskflgli
IPI00921422	48	47		PKA->ATM+PKA+DNAPK		9793	CKAP5	kikdekspewSkflglikkfv
IPI00921844	264	267	unsp+DNAPK->DNAPK	unsp+DNAPK->DNAPK		701	BUB1B	irvggalkapSqnrglqnpfp
IPI00924816	97	95	unsp+PKC->cdc2	unsp+PKC->		136319	MTPN	klllskgadkTvkgpdgltaf
IPI00925046	309	305		unsp+CKII->CKII		5859	QARS	ngicflrfddTnpekeeakff
IPI00925255	28	32	unsp+PKC+cdc2->unsp+cdc2	unsp+PKC+cdc2->unsp+cdc2		222194	RSBN1L	ekepfgklqlSsrdppgslsa
IPI00926625	265	259	unsp+GSK3+cdk5+cdc2->unsp+cdk5+cdc2		Uniprot|PHOsphoELM|PhosphoSi	7791	ZYX	ntqprgppasSpapapkfspv
IPI00926625	265	267	unsp+GSK3+cdk5->GSK3+cdk5+p38MAPK		Uniprot|PHOsphoELM|PhosphoSi	7791	ZYX	asspapapkfSpvtpkftpva
IPI00926625	272	267	unsp+GSK3+cdk5->cdk5+p38MAPK	unsp+GSK3+cdk5->cdk5+p38MAPK	niprot|PHOsphoELM|PhosphoSi	7791	ZYX	asspapapkfSpvtpkftpva
IPI00926625	272	270	unsp+cdk5+p38MAPK->cdk5+p38MAPK	unsp+cdk5+p38MAPK->cdk5+p38MAPK	Uniprot|PhosphoSite	7791	ZYX	papapkfspvTpkftpvaskf
IPI00926625	272	274	unsp+cdk5+p38MAPK->GSK3+cdk5+p38MAPK	unsp+cdk5+p38MAPK->unsp+GSK3+cdk5+p38MAPK	Uniprot|PhosphoSite	7791	ZYX	pkfspvtpkfTpvaskfspga
IPI00926625	279	278		->ATM+DNAPK	PhosphoSite	7791	ZYX	pvtpkftpvaSkfspgapggs
IPI00926625	279	281	unsp+CKI+GSK3+cdk5+p38MAPK->CKI+GSK3+cdk5+p38MAPK		Uniprot|PHOsphoELM|PhosphoSi	7791	ZYX	pkftpvaskfSpgapggsgsq
IPI00926625	279	288	->cdc2	->cdc2	Uniprot|PhosphoSite	7791	ZYX	skfspgapggSgsqpnqklgh
IPI00929648	86	82	unsp->unsp+PKA+RSK	unsp->unsp+PKA		2029	ENSA	lglkevlrmkSveqkyfdsgd
IPI00929648	86	90		CKII+PKA->	niprot|PHOsphoELM|PhosphoSi	2029	ENSA	mksveqkyfdSgdynmakakm
IPI00930609	51	47	unsp+PKC+PKA->unsp+PKA	unsp+PKC+PKA->unsp+PKA		29968	PSAT1	isvlemshrsSdfakiinnte
IPI00930609	333	331	unsp->	unsp->		29968	PSAT1	ldkalelnmlSlkghrsvggi
IPI00930609	333	337		->cdc2		29968	PSAT1	lnmlslkghrSvggiraslyn
IPI00930688	40	38	PKC->	PKC->		10376	TUBA1B	hgiqpdgqmpSdktigggdds
IPI00930688	40	41	->CKI	->unsp	Uniprot	10376	TUBA1B	qpdgqmpsdkTigggddsfnt
IPI00930688	163	158	unsp+cdc2->unsp+PKA			10376	TUBA1B	gftsllmerlSvdygkkskle
IPI00930688	163	165	unsp+PKC+PKG+CKII->unsp+PKC+CKII	unsp+PKC+PKG+CKII->unsp+CKII		10376	TUBA1B	erlsvdygkkSklefsiypap
IPI00930688	280	282	EGFR->unsp	EGFR->unsp+EGFR		10376	TUBA1B	yapvisaekaYheqlsvaeit
IPI00930688	311	312		->INSR		10376	TUBA1B	mvkcdprhgkYmaccllyrgd
IPI00930688	401	399	unsp->		PhosphoSite	10376	TUBA1B	arldhkfdlmYakrafvhwyv

Table S3

IPI00936931	267	263		unsp->SRC+unsp		10613	ERLIN1	rekakadaeyYaahkyatsnk
IPI00936931	267	268	unsp->			10613	ERLIN1	adaeyyaahkYatsnkhkltp
IPI00937545	1136	1139	PKC->			23047	PDS5B	nvlgavnkplSsagkqsqtks
IPI00937545	1136	1140	PKC->			23047	PDS5B	vlgavnkplsSagkqsqtkss
IPI00939163	68	65	PKC->	PKC->		10808	HSPH1	qqithanntvSnfkrfhgraf
IPI00939163	272	278	unsp->unsp+PKA			10808	HSPH1	qeceklkklmSsnstdlplni
IPI00939163	272	279	PKA->cdc2+PKA	PKA->cdc2+PKA		10808	HSPH1	eceklkklmsSnstdlplnie
IPI00939163	272	281		->cdc2		10808	HSPH1	eklkklmssnStdlplniecf
IPI00939163	430	429	->PKA	->DNAPK		10808	HSPH1	vfsrnhaapfSkvltflrrgp
IPI00939238	51	45	->cdc2	->cdc2	PhosphoSite	861	RUNX1	stsrrftppsTalspgkmsea
IPI00939238	51	53	PKA->	PKA->		861	RUNX1	pstalspgkmSealplgapda
IPI00939238	70	73	unsp+PKC->PKC	unsp+PKC->PKC		861	RUNX1	agaalagklrSgdrsmvevla
IPI00939265	262	259		unsp+EGFR->EGFR	PhosphoSite	55303	GIMAP4	erekarireeYeekirkledk
IPI00939320	173	168	unsp+PKC->unsp+PKC+PKA			23049	SMG1	reddrdrrlaTvkqlkefiqq
IPI00939493	189	183		CKII->		25902	MTHFD1L	lkpekdvdgvTdinlgklvrg
IPI00939879	144	149	PKG+PKA->			164	AP1G1	agevekllktSnsylrkkaal
IPI00940148	54	61	unsp+GSK3+cdk5+p38MAPK->unsp+GSK3+p38MAPK	unsp+GSK3+cdk5+p38MAPK->unsp+GSK3+p38MAPK	Uniprot|PhosphoSite	2665	GDI2	dlykrfkipgSppesmgrgrd
IPI00940148	112	108	CKI+PKC->CKI+PKA	CKI+PKC->CKI		2665	GDI2	ryldfkvtegSfvykggkiyk
IPI00940237	35	37	PKC+PKA->PKC	PKC+PKA->PKC	PhosphoSite	10212	DDX39	pappkkdikgSyvsihssgfr
IPI00940237	35	38	INSR+unsp->	INSR+unsp->unsp	PhosphoSite	10212	DDX39	appkkdikgsYvsihssgfrd
IPI00940237	35	40	unsp+PKC->	unsp+PKC->	Uniprot|PhosphoSite	10212	DDX39	pkkdikgsyvSihssgfrdfl
IPI00940237	187	183	PKA->PKA+RSK	PKA->PKG+PKA+RSK		10212	DDX39	grilalvrnrSfslknvkhfv
IPI00940237	187	185	unsp+PKC+PKB+PKA+RSK->unsp+PKA+RSK	unsp+PKC+PKB+PKA+RSK->unsp+PKA+RSK		10212	DDX39	ilalvrnrsfSlknvkhfvld
IPI00940237	333	329		->EGFR		10212	DDX39	gmaqeerlsrYqqfkdfqrri
IPI00940535	61	60	->cdc2+PKA+DNAPK	->ATM+DNAPK		8379	MAD1L1	qleeraeqirSkshliqvere
IPI00940535	61	62	unsp+PKA+RSK->PKA+RSK	unsp+PKA+RSK->PKA+RSK		8379	MAD1L1	eeraeqirskShliqverekm
IPI00940842	101	100	unsp+PKC+CKII->unsp+PKC			728026	LOC728026	aeddedddvdTkkqktdkdd-
IPI00940842	101	105	unsp+PKC+CKII->unsp+CKII			728026	LOC728026	dddvdtkkqkTdkdd------
IPI00941161	815	813	unsp+PKC->CKII	unsp+PKC->		8615	USO1	sqsveitklqTekqellqkte
IPI00941255	238	233	CKI->CKI+p38MAPK	CKI->		8665	EIF3F	vpgrtmgvmfTpltvkyayyd
IPI00941255	238	236	unsp+PKC->PKC	unsp+PKC->PKC		8665	EIF3F	rtmgvmftplTvkyayydter
IPI00941331	196	190	unsp+CKI+CKII->unsp+PKA+CKII	unsp+CKI+CKII->unsp+CKI+PKA+CKII		23157	6-Sep	ipiiakadaiSkseltkfkik
IPI00941501	394	390	PKC->	PKC->		4603	MYBL1	isdaaaspikStpvklmriqh
IPI00941501	394	391	unsp+cdk5+p38MAPK->unsp+p38MAPK			4603	MYBL1	sdaaaspiksTpvklmriqhn
IPI00941928	496	499	unsp+cdc2+DNAPK->cdc2+DNAPK	unsp+cdc2+DNAPK->cdc2+DNAPK		64210	MMS19	lyrlsflkedSqscrvaalea
IPI00941928	496	501	unsp->	unsp->		64210	MMS19	rlsflkedsqScrvaaleasg
IPI00942092	147	151	unsp+PKA+DNAPK->PKA+DNAPK			158	ADSL	rladfakeraSlptlgfthfq
IPI00942246	516	514	unsp+PKC->unsp+PKA	unsp+PKC->unsp+PKA		271	AMPD2	eifiktdnrvSgkyfahiike
IPI00942458	510	506	unsp+PKC->unsp	unsp+PKC->PKC+PKG		64062	RBM26	sgepgvptkkTwfdkpnfnrt
IPI00942495	236	234	unsp+PKC->CaM-II	unsp+PKC->		10001	MED6	ettknvqqtvSakgppekrmr
IPI00942668	44	43		->DNAPK		79156	PLEKHF1	grvllgegvlTkecrkkakpr
IPI00942760	340	339	PKC+cdc2->PKC+PKA+cdc2			23107	MRPS27	qylerfkalhSklqalgkies
IPI00942976	43	51	->cdc2			2053	EPHX2	fqkggpegatTrlmkgeitls
IPI00942979	6	3	PKC->	PKC->		7086	TKT	--------meSyhkpdqqklq
IPI00942979	232	236		PKG->		7086	TKT	kafgqakhqpTaiiaktfkgr
IPI00942979	241	249	unsp+PKA->unsp	unsp+PKA->unsp		7086	TKT	iaktfkgrgiTgvedkeswhg
IPI00942979	352	348	unsp+CKI+PKC->unsp+CKI			7086	TKT	ldgdtknstfSeifkkehpdr
IPI00942979	456	454	unsp+PKC+CKII->CKII	unsp+PKC+CKII->CKII		7086	TKT	tvfypsdgvaTekavelaant
IPI00942979	597	595	unsp+PKC->	unsp+PKC->unsp		7086	TKT	thlavnrvprSgkpaellkmf
IPI00943074	459	461	unsp+PKC->PKC			5589	PRKCSH	swigpdhdkfSamkyeqgtgc
IPI00943093	305	301	unsp+PKC+CKII->unsp+CKII	unsp+PKC+CKII->unsp+CKII		23526	HMHA1	nmakymkdliSylekrttlem
IPI00943093	305	307	PKG+CKII->CKII	PKG+CKII->CKII		23526	HMHA1	kdlisylekrTtlemefakgl
IPI00945964	175	171	PKC->	PKC->		85476	GFM1	qmkrynvpflTfinkldrmgs
IPI00945964	175	181	RSK->PKA+RSK	RSK->PKA+RSK		85476	GFM1	tfinkldrmgSnparalqqmr
IPI00946154	168	166	unsp->			2247	FGF2	pghfkdpkrlYcknggfflri
IPI00946154	261	257		->EGFR		2247	FGF2	tyrsrkytswYvalkrtgqyk
IPI00946154	267	263	->PKA	->PKG		2247	FGF2	ytswyvalkrTgqyklgsktg
IPI00946154	267	270	PKC->PKC+cdc2	PKC->PKC+cdc2		2247	FGF2	lkrtgqyklgSktgpgqkail
IPI00946154	267	272		->cdc2		2247	FGF2	rtgqyklgskTgpgqkailfl
IPI00946154	271	270	PKC->PKC+PKA	PKC->PKC+DNAPK		2247	FGF2	lkrtgqyklgSktgpgqkail
IPI00946154	277	270	PKC->PKC+cdc2			2247	FGF2	lkrtgqyklgSktgpgqkail
IPI00953461	724	727	unsp+PKC+PKA->unsp+PKC	unsp+PKC+PKA->unsp+PKC	Uniprot|PhosphoSite	23185	LARP4B	pspsamgkrlSreqstppksp
IPI00953625	353	352		CKI->DNAPK		339287	MSL1	tpvkklapefSkvktktpkhs
IPI00953625	353	358		unsp+PKC+p38MAPK->unsp+PKC+GSK3+p38MAPK		339287	MSL1	apefskvktkTpkhspikeep
