# Supplementary material for: Bioinformatic Analysis and Post-Translational Modification Crosstalk Prediction of Lysine Acetylation
Source: PLoS One. 2011 Dec 2;6(12):e28228. doi: 10.1371/journal.pone.0028228 (PMC3229533; doi:10.1371/journal.pone.0028228)
Supplement: Table S4 — Methylation sites affected. We studied the effects on methylation using BPB-PPMS (cutoff 0.8). The contribution of lysine substitution by either leucine or glutamine was studied. (PDF) [file pone.0028228.s007.pdf]

Table S4

IPI	KacPos	PhosphPos	Kac->L	Kac->Q	KnownPhosph	GeneID
IPI00005630	117	120		K->		1869
IPI00005630	120	120	K->	K->		1869
IPI00005630	125	120	K->	K->		1869
IPI00005996	26	29		->R		8091
IPI00007913	128	132	->K	->K		23092
IPI00010700	1196	1195	K->	K->		7916
IPI00012011	114	112	->K			1072
IPI00012011	121	121	K->	K->		1072
IPI00012011	132	127	K->	K->		1072
IPI00012199	212	213		K->		79080
IPI00012199	213	213	K->	K->		79080
IPI00013999	316	314	->K			2623
IPI00013999	316	315	K->			2623
IPI00018279	251	254	->K			50509
IPI00018279	255	260	K->	K->		50509
IPI00019755	143	143	K->	K->		9446
IPI00019755	148	143		K->		9446
IPI00021175	504	509	K->	K->		51755
IPI00021924	179	180		K->		8971
IPI00021924	180	180	K->	K->		8971
IPI00021924	182	179	->K	->K		8971
IPI00021924	182	180		K->		8971
IPI00021924	185	180	K->	K->		8971
IPI00026089	1067	1070	->K			23451
IPI00027415	947	951	K->	K->		170506
IPI00028888	341	345		->R	Uniprot	3184
IPI00074893	843	842		K->		57473
IPI00152653	322	320	->K			1767
IPI00171611	10	10	K->	K->	Uniprot	653604
IPI00171611	15	10	K->	K->	Uniprot	653604
IPI00216402	10	10	K->	K->		8290
IPI00216402	15	10	K->	K->		8290
IPI00216402	28	28	K->	K->	Uniprot	8290
IPI00217465	63	63	K->	K->		3006
IPI00217466	64	64	K->	K->		3007
IPI00217467	63	63	K->	K->		3008
IPI00217467	90	85		K->		3008
IPI00217467	136	137	->K			3008
IPI00217467	168	169	->K			3008
IPI00217468	207	204	->K	->K		3009
IPI00217469	88	88	K->	K->		3024
IPI00217469	93	88	K->	K->		3024
IPI00219038	10	10	K->	K->	Uniprot	644914
IPI00219038	15	10	K->	K->	Uniprot	644914
IPI00219038	28	28	K->	K->	Uniprot	644914
IPI00219038	37	37	K->	K->	Uniprot	644914
IPI00219038	38	37	K->	K->	Uniprot	644914
IPI00220289	198	196	->K			84181
IPI00220403	13	16		K->		3018
IPI00220403	16	16	K->	K->		3018
IPI00220403	17	16	K->	K->		3018

Table S4

IPI00220403	21	16	K->	K->		3018
IPI00220403	24	24	K->	K->		3018
IPI00221394	474	477	->K			1736
IPI00292771	2070	2070	K->	K->		4926
IPI00292771	2071	2070	K->	K->		4926
IPI00333533	631	631	K->	K->		367
IPI00333533	633	631		K->		367
IPI00377245	4244	4244	K->	K->		8085
IPI00383105	490	490	K->	K->		80205
IPI00385267	169	173		->K		6734
IPI00385267	170	173	->K			6734
IPI00385267	170	174		->K		6734
IPI00394926	287	282		K->		10714
IPI00396485	386	385	K->	K->		1915
IPI00409671	25	25	K->	K->		11325
IPI00419884	10	10	K->	K->		440093
IPI00419884	15	10	K->	K->		440093
IPI00453473	6	9	->K			121504
IPI00455210	115	116		K->		1108
IPI00455210	115	118	->K			1108
IPI00455210	116	116	K->	K->		1108
IPI00465070	10	10	K->	K->	Uniprot	8356
IPI00465070	15	10	K->	K->	Uniprot	8356
IPI00465070	28	28	K->	K->	Uniprot	8356
IPI00465070	37	37	K->	K->	Uniprot	8356
IPI00473014	114	112	->K			11034
IPI00479571	35	33	->K			84062
IPI00554798	13	16		K->		8342
IPI00554798	16	16	K->	K->		8342
IPI00554798	17	16	K->	K->		8342
IPI00554798	21	16	K->	K->		8342
IPI00554798	24	24	K->	K->		8342
IPI00645192	461	461	K->	K->		4204
IPI00743143	127	129		K->		51147
IPI00743143	129	129	K->	K->		51147
IPI00743143	130	129	K->	K->		51147
IPI00743143	131	129		K->		51147
IPI00743143	156	156	K->	K->		51147
IPI00743143	161	156	K->	K->		51147
IPI00746310	23	24	R->			3159
IPI00746310	55	58	R->		Uniprot	3159
IPI00782966	1350	1351	->K			64397
IPI00815713	155	159	K->	K->		6949
IPI00885104	170	171	->K			54737

Table S4

Symbol	Seq
E2F1	grgrhpgkgvKspgeksryet
E2F1	grgrhpgkgvKspgeksryet
E2F1	grgrhpgkgvKspgeksryet
HMGA2	paapapqkrgRgrprkqqqep
ARHGAP26	eqigaakeakKkydketekyc
BAT2	wsppakslapKkpptgplpps
CFL1	ifwapesaplKskmiyasskd
CFL1	lkskmiyassKdaikkkltgi
CFL1	yasskdaikkKltgikhelqa
CCDC86	etvtggfgakKrkgsssqapa
CCDC86	etvtggfgakKrkgsssqapa
GATA1	trnrkasgkgKkkrgsslggt
GATA1	rnrkasgkgkKkrgsslggtg
COL5A3	prrkgkgkgrKkgrgrkgkgr
COL5A3	gkgrkkgrgrKgkgrkknkei
GSTO1	sqnkedyaglKeefrkeftkl
GSTO1	sqnkedyaglKeefrkeftkl
CDK12	gtrdskpialKeeivtpkete
H1FX	rshkkgagakKdkggkakkta
H1FX	rshkkgagakKdkggkakkta
H1FX	qrshkkgagaKkdkggkakkt
H1FX	rshkkgagakKdkggkakkta
H1FX	rshkkgagakKdkggkakkta
SF3B1	fellellkahKkairratvnt
DHX36	riahlvkelrKeldillqeki
HNRNPD	qqsgygkvsrRgghqnsykpy
ZNF512B	kekkknlaggKkrgrkpkert
DNAH5	kavlavlaaaKskllktwrem
HIST2H3D	-martkqtarKstggkaprkq
HIST2H3D	-martkqtarKstggkaprkq
HIST3H3	-martkqtarKstggkaprkq
HIST3H3	-martkqtarKstggkaprkq
HIST3H3	rkqlatkvarKsapatggvkk
HIST1H1C	ersgvslaalKkalaaagydv
HIST1H1D	ersgvslaalKkalaaagydv
HIST1H1E	ersgvslaalKkalaaagydv
HIST1H1E	knnsriklglKslvskgtlvq
HIST1H1E	kakkpagaakKpkkatgaatp
HIST1H1E	kkpaaaagakKakspkkakaa
HIST1H1B	kavkpkaakpKaakpkaakpk
HIST1H1A	knnsriklgiKslvskgtlvq
HIST1H1A	knnsriklgiKslvskgtlvq
LOC644914	-martkqtarKstggkaprkq
LOC644914	-martkqtarKstggkaprkq
LOC644914	rkqlatkaarKsapstggvkk
LOC644914	rksapstggvKkphryrpgtv
LOC644914	rksapstggvKkphryrpgtv
CHD6	keqgptpvekKkkgkrksett
HIST1H2BB	ksapapkkgsKkaitkaqkkd
HIST1H2BB	ksapapkkgsKkaitkaqkkd
HIST1H2BB	ksapapkkgsKkaitkaqkkd

Table S4

HIST1H2BB	ksapapkkgsKkaitkaqkkd
HIST1H2BB	gskkaitkaqKkdgkkrkrsr
DKC1	kkekkkskkdKkakaglesga
NUMA1	gnsllrrgasKkalskaspnt
NUMA1	gnsllrrgasKkalskaspnt
AR	yeagmtlgarKlkklgnlklq
AR	yeagmtlgarKlkklgnlklq
MLL2	psnkedaaarKpltpkpkrvq
CHD9	clqrqppsskKsdgsgtytkl
SRPR	kaknskkkgaKkegsdgplat
SRPR	kaknskkkgaKkegsdgplat
SRPR	aknskkkgakKegsdgplats
POLD3	epklatpaglKksskkaepvk
EEF1A1	lkekidrrsgKkledgpkflk
DDX42	gfggfaisagKkeepklpqqs
H3F3C	-martkqtarKstggkaprkq
H3F3C	-martkqtarKstggkaprkq
HIST4H4	--msgrgkggKglgkggakrh
CHD4	egsdytpgkkKkkklgpkkek
CHD4	sdytpgkkkkKklgpkkekks
CHD4	egsdytpgkkKkkklgpkkek
HIST1H3J	-martkqtarKstggkaprkq
HIST1H3J	-martkqtarKstggkaprkq
HIST1H3J	rkqlatkaarKsapatggvkk
HIST1H3J	rksapatggvKkphryrpgtv
DSTN	flwapelaplKskmiyasskd
DTNBP1	lsdksreakvKskprtvpflp
HIST1H2BM	ksapvpkkgsKkainkaqkkd
HIST1H2BM	ksapvpkkgsKkainkaqkkd
HIST1H2BM	ksapvpkkgsKkainkaqkkd
HIST1H2BM	ksapvpkkgsKkainkaqkkd
HIST1H2BM	gskkainkaqKkdgkkrkrsr
MECP2	avataataaeKykhrgegerk
ING4	sdydsssskgKkkgrtqkekk
ING4	sdydsssskgKkkgrtqkekk
ING4	sdydsssskgKkkgrtqkekk
ING4	sdydsssskgKkkgrtqkekk
ING4	kgknsdeeapKtaqkklklvr
ING4	kgknsdeeapKtaqkklklvr
HMGA1	skqekdgtekRgrgrprkqpp
HMGA1	psevptpkrpRgrpkgskknw
ZFP106	ltsaetrgskKkkklrkkksl
TCOF1	anllsgksprKsaepsanttl
MPHOSPH8	eekspddlkkKkakagklkdk
