# Supplementary material for: Bioinformatic Analysis and Post-Translational Modification Crosstalk Prediction of Lysine Acetylation
Source: PLoS One. 2011 Dec 2;6(12):e28228. doi: 10.1371/journal.pone.0028228 (PMC3229533; doi:10.1371/journal.pone.0028228)
Supplement: Table S5 — Ubiquitination sites affected. We studied the effects on ubiquitination using UbPred (cutoff 0.84). The contribution of lysine substitution by either leucine or glutamine was studied. (PDF) [file pone.0028228.s008.pdf]

Table S5

IPI	KacPos	PhosphPos	Kac->L	Kac->Q	KnownPhosph	GeneID
IPI00000057	3	3	1->	1->		22796
IPI00000057	3	10	1->			22796
IPI00000494	221	241	1->			6125
IPI00000712	536	533		->1		4780
IPI00000712	538	533		->1		4780
IPI00000712	548	574	1->			4780
IPI00000712	554	574	1->			4780
IPI00000712	555	574	1->			4780
IPI00001735	878	857	->1			9612
IPI00001735	878	863	->1			9612
IPI00001735	878	876	->1	->1		9612
IPI00001735	2037	2037	1->	1->		9612
IPI00002335	444	440		->1		3064
IPI00002335	444	442		->1		3064
IPI00002564	256	260	->1			7515
IPI00002564	260	256	->1			7515
IPI00002966	603	609	1->	1->		3308
IPI00002966	603	612	1->	1->		3308
IPI00003362	548	580	1->	1->		3309
IPI00003362	586	574	1->			3309
IPI00003362	586	582	->1	->1		3309
IPI00003362	586	592	->1	->1		3309
IPI00003442	389	377	1->			3091
IPI00003442	389	392		->1		3091
IPI00003443	86	116	->1	->1		3428
IPI00003817	21	21	1->	1->		397
IPI00003817	25	30	->1	->1		397
IPI00003817	33	30	->1	->1		397
IPI00003817	33	33	1->	1->		397
IPI00003865	512	507		->1		3312
IPI00003865	512	512	1->	1->		3312
IPI00003865	524	524	1->	1->		3312
IPI00003865	524	526	->1	->1		3312
IPI00003865	524	561	1->			3312
IPI00003865	526	512	1->			3312
IPI00003865	526	531	->1	->1		3312
IPI00003865	526	561	1->			3312
IPI00003865	531	526	->1	->1		3312
IPI00003865	531	535	->1	->1		3312
IPI00003865	531	561	1->			3312
IPI00003865	589	561	1->			3312
IPI00004233	1178	1178	1->	1->		4288
IPI00004233	1185	1178	1->			4288
IPI00004233	1185	1185	1->	1->		4288
IPI00004233	1639	1643		->1		4288
IPI00004233	2005	1982	1->	1->		4288
IPI00005630	117	125	->1	->1		1869
IPI00005630	120	125	->1	->1		1869
IPI00005648	293	293	1->	1->		9667
IPI00005657	21	15	->1	->1		10471
IPI00005657	21	21	1->	1->		10471

Table S5

IPI00005744	30	17		->1	6473
IPI00006079	335	332		->1	9774
IPI00006079	437	439		->1	9774
IPI00006091	1017	1004	1->		1756
IPI00006108	188	189	1->		9794
IPI00006108	405	407	->1	->1	9794
IPI00006108	407	405	->1	->1	9794
IPI00006160	327	321		->1	7161
IPI00006160	331	321		->1	7161
IPI00006176	207	226	1->		9146
IPI00006213	399	393	1->		5108
IPI00006252	33	63	1->	1->	9255
IPI00006379	441	444		->1	51602
IPI00006601	153	153	1->	1->	1114
IPI00006601	153	159	->1		1114
IPI00006658	57	52	->1		5303
IPI00006987	71	75	->1	->1	57062
IPI00007074	474	474	1->	1->	8565
IPI00007156	95	95	1->	1->	51176
IPI00007311	71	81	1->		4097
IPI00007311	76	81	1->	1->	4097
IPI00007311	76	83	1->		4097
IPI00007334	717	731	->1		22985
IPI00007675	429	428	->1	->1	51143
IPI00007675	429	435	1->	1->	51143
IPI00007765	567	555	1->	1->	3313
IPI00007765	567	563	1->	1->	3313
IPI00007765	595	555	1->		3313
IPI00007765	595	595	1->	1->	3313
IPI00007765	610	612		->1	3313
IPI00007765	675	653	1->		3313
IPI00007927	222	211	1->	1->	10592
IPI00007927	321	320		->1	10592
IPI00007927	330	332	->1		10592
IPI00007993	333	296		1->	3090
IPI00008054	550	546		->1	27154
IPI00008477	305	269	1->		22974
IPI00008477	305	279	1->		22974
IPI00009032	116	105	1->	1->	6741
IPI00009032	360	363	->1	->1	6741
IPI00009286	1133	1130	->1		4297
IPI00009328	60	19	1->		9775
IPI00009713	61	61	1->	1->	6662
IPI00009713	253	242	->1		6662
IPI00009713	398	398	1->	1->	6662
IPI00009904	533	485	->1	->1	9601
IPI00009960	211	211	1->	1->	10989
IPI00009960	211	216	1->		10989
IPI00009960	451	442	1->		10989
IPI00009960	506	506	1->	1->	10989
IPI00010085	356	353	1->		5326
IPI00010085	356	356	1->	1->	5326

Table S5

IPI00010193	399	399	1->	1->	3455
IPI00010196	286	286	1->	1->	8204
IPI00010196	310	310	1->	1->	8204
IPI00010196	481	474	1->		8204
IPI00010196	481	481	1->	1->	8204
IPI00010196	481	508	1->		8204
IPI00010196	528	508	1->		8204
IPI00010196	606	606	1->	1->	8204
IPI00010252	763	763	1->	1->	51592
IPI00010252	763	769	->1	->1	51592
IPI00010252	769	774	->1	->1	51592
IPI00010415	168	192	->1	->1	11332
IPI00010415	198	192	->1	->1	11332
IPI00010586	522	515	1->	1->	7110
IPI00010700	1196	1206	->1	->1	7916
IPI00010740	472	462	1->		6421
IPI00010740	472	466	1->		6421
IPI00011857	494	506	1->		8208
IPI00011875	98	98	1->	1->	3550
IPI00011875	386	386	1->	1->	3550
IPI00011875	386	388	->1	->1	3550
IPI00012079	591	583	->1		1975
IPI00012149	609	609	1->	1->	10199
IPI00012149	609	612	->1	->1	10199
IPI00012820	379	391	1->		604
IPI00013122	154	149	->1	->1	11140
IPI00013297	132	137	->1	->1	11333
IPI00013452	535	528	1->		2058
IPI00013452	788	788	1->	1->	2058
IPI00013452	788	792	->1	->1	2058
IPI00013830	115	141	1->	1->	22938
IPI00013894	100	100	1->	1->	10963
IPI00013991	118	112	->1		7169
IPI00014533	61	35	1->		7343
IPI00014938	142	142	1->	1->	84324
IPI00014938	142	149		->1	84324
IPI00015180	1341	1315	->1	->1	357
IPI00015286	1605	1610		->1	1793
IPI00015833	142	136	1->		54927
IPI00015947	44	35		->1	3337
IPI00015947	44	37	1->		3337
IPI00015947	44	44	1->	1->	3337
IPI00015947	44	46	1->		3337
IPI00015947	46	37	1->		3337
IPI00015947	46	44	1->		3337
IPI00015947	46	46	1->	1->	3337
IPI00016405	131	133	->1		54940
IPI00017305	75	42	1->	1->	6195
IPI00017451	251	266	1->	1->	10291
IPI00017592	597	603		->1	3954
IPI00017659	81	88	1->	1->	92241
IPI00017855	520	521	1->	1->	50

Table S5

IPI00018251	31	31	1->	1->	10499
IPI00018251	636	640		->1	10499
IPI00018251	640	636	->1	->1	10499
IPI00018251	780	785	->1	->1	10499
IPI00018251	785	780	->1	->1	10499
IPI00018251	788	785	->1	->1	10499
IPI00018251	788	788	1->	1->	10499
IPI00018823	512	512	1->	1->	80314
IPI00019226	481	481	1->	1->	10902
IPI00019226	481	493	1->		10902
IPI00019380	698	698	1->	1->	4686
IPI00019502	992	995		->1	4627
IPI00019502	1024	1014	->1	->1	4627
IPI00019502	1024	1022		->1	4627
IPI00019502	1357	1357	1->	1->	4627
IPI00019502	1404	1404	1->	1->	4627
IPI00019502	1410	1413	->1	->1	4627
IPI00019502	1459	1459	1->	1->	4627
IPI00019502	1828	1788	->1		4627
IPI00019502	1828	1815	1->		4627
IPI00019502	1828	1828	1->	1->	4627
IPI00019812	40	32	1->		5536
IPI00019812	42	32	1->		5536
IPI00019848	2005	2011		->1	3054
IPI00020567	80	43		->1	392
IPI00020599	206	209		->1	811
IPI00020599	209	206		->1	811
IPI00020599	209	207	->1	->1	811
IPI00020599	238	232	->1	->1	811
IPI00020985	77	77	1->	1->	2033
IPI00020985	77	79	1->		2033
IPI00020985	79	77	1->		2033
IPI00020985	79	79	1->	1->	2033
IPI00020985	291	291	1->	1->	2033
IPI00020985	292	291	1->		2033
IPI00020985	292	292	1->	1->	2033
IPI00020985	970	970	1->	1->	2033
IPI00020985	970	1001	->1		2033
IPI00020985	977	970	1->		2033
IPI00020985	977	977	1->	1->	2033
IPI00020985	977	1001	->1		2033
IPI00020985	981	981	1->	1->	2033
IPI00020985	981	1001	->1		2033
IPI00020985	1020	1001	->1		2033
IPI00020985	1024	1001	->1		2033
IPI00020985	1024	1024	1->	1->	2033
IPI00020985	1228	1228	1->	1->	2033
IPI00020985	1228	1233		->1	2033
IPI00020985	1228	1235		->1	2033
IPI00020985	1518	1518	1->	1->	2033
IPI00020985	1528	1518	1->		2033
IPI00021175	504	496		->1	51755

Table S5

IPI00021175	504	504	1->	1->	51755
IPI00021175	504	509	1->		51755
IPI00021263	49	75	1->		7534
IPI00021405	97	90		->1	4000
IPI00021405	97	97	1->	1->	4000
IPI00021405	108	97	1->	1->	4000
IPI00021405	108	108	1->	1->	4000
IPI00021405	108	114	->1	->1	4000
IPI00021405	114	97	1->	1->	4000
IPI00021405	114	108	1->	1->	4000
IPI00021405	114	117	->1	->1	4000
IPI00021405	233	233	1->	1->	4000
IPI00021405	260	261		->1	4000
IPI00021405	260	270		->1	4000
IPI00021405	261	260	->1	->1	4000
IPI00021405	270	265		->1	4000
IPI00021405	378	378	1->	1->	4000
IPI00021885	620	599		->1	2243
IPI00021885	625	599		->1	2243
IPI00021926	15	7	->1	->1	5706
IPI00021926	15	14	1->		5706
IPI00021926	15	20		->1	5706
IPI00021926	20	15	->1	->1	5706
IPI00022055	428	428	1->	1->	8850
IPI00022055	430	430	1->	1->	8850
IPI00022348	515	515	1->	1->	5371
IPI00022542	647	647	1->	1->	6093
IPI00022542	718	719	->1		6093
IPI00022542	718	721	->1		6093
IPI00022542	719	709	1->		6093
IPI00022865	112	108	->1	->1	890
IPI00022865	112	113		->1	890
IPI00022865	112	119	1->		890
IPI00023340	815	815	1->	1->	7994
IPI00023649	149	149	1->	1->	55206
IPI00023649	149	155	1->		55206
IPI00024568	1057	1049	->1	->1	29998
IPI00024975	797	792	1->		56992
IPI00024975	1009	982	1->		56992
IPI00025087	292	292	1->	1->	7157
IPI00025087	305	305	1->	1->	7157
IPI00025087	319	320	->1	->1	7157
IPI00025087	319	321	->1	->1	7157
IPI00025087	320	319	->1	->1	7157
IPI00025087	320	321	->1	->1	7157
IPI00025087	321	319	->1	->1	7157
IPI00025087	321	320	->1	->1	7157
IPI00025087	381	386	->1	->1	7157
IPI00025087	382	386		->1	7157
IPI00025807	551	551	1->	1->	6670
IPI00026089	141	141	1->	1->	23451
IPI00026089	141	146	1->		23451

Table S5

IPI00026089	141	182	1->		23451
IPI00026089	333	335		->1	23451
IPI00026156	123	96	1->		3059
IPI00026156	123	109	1->		3059
IPI00026156	123	133	1->		3059
IPI00026156	241	224	1->		3059
IPI00026156	241	249	->1	->1	3059
IPI00026156	241	251	->1		3059
IPI00026156	241	257		1->	3059
IPI00026309	27	17	->1	->1	51053
IPI00026337	21	9	1->		8498
IPI00026337	21	23	->1	->1	8498
IPI00026337	21	26	->1	->1	8498
IPI00026337	23	21		->1	8498
IPI00026337	23	26	->1	->1	8498
IPI00026337	23	32	->1		8498
IPI00026559	199	199	1->	1->	7391
IPI00026940	83	110		1->	10762
IPI00026940	275	276	1->	1->	10762
IPI00026940	276	276	1->	1->	10762
IPI00027230	613	603		->1	7184
IPI00027230	613	613	1->	1->	7184
IPI00027569	39	8	1->	1->	343069
IPI00027834	59	62	->1	->1	3191
IPI00027988	18	20		->1	10664
IPI00027988	20	18		->1	10664
IPI00029196	384	366	1->	1->	5588
IPI00029400	54	46		->1	9406
IPI00029485	230	264	->1	->1	1639
IPI00029601	87	70	1->		2017
IPI00029601	87	97	1->		2017
IPI00029601	198	181	1->	1->	2017
IPI00029601	203	181	1->	1->	2017
IPI00029601	203	218	1->		2017
IPI00029601	218	218	1->	1->	2017
IPI00029601	235	218	1->		2017
IPI00029629	273	249	1->	1->	7706
IPI00029629	273	262	1->		7706
IPI00029697	297	311	1->		5393
IPI00029764	489	489	1->	1->	10946
IPI00029764	489	490	1->	1->	10946
IPI00029795	280	283	->1	->1	4782
IPI00030247	492	492	1->	1->	904
IPI00030247	492	502	->1	->1	904
IPI00030274	499	488	->1		84081
IPI00030274	499	499	1->	1->	84081
IPI00030274	499	511	1->		84081
IPI00030275	87	87	1->	1->	10131
IPI00030915	601	606		->1	9101
IPI00031519	235	239	->1	->1	1786
IPI00032358	714	714	1->	1->	1E+08
IPI00032358	714	717		->1	1E+08

Table S5

IPI00032358	717	714	1->		1E+08
IPI00032598	388	388	1->	1->	2117
IPI00032831	124	126	1->	1->	9342
IPI00032904	6	21	1->	1->	6620
IPI00032904	6	23	1->	1->	6620
IPI00032904	12	21	1->	1->	6620
IPI00032904	12	23	1->	1->	6620
IPI00033016	163	172		->1	4609
IPI00033487	11	13	->1	->1	338917
IPI00036742	166	160	->1		25962
IPI00045550	774	774	1->	1->	84687
IPI00045550	774	775		->1	84687
IPI00045914	1998	1999	->1		23013
IPI00045914	1998	2005	->1	->1	23013
IPI00045914	1999	2005	->1	->1	23013
IPI00045914	2001	1999	->1	->1	23013
IPI00045914	2001	2005	->1	->1	23013
IPI00060181	233	226	->1	->1	79180
IPI00060473	484	484	1->	1->	115704
IPI00061009	403	402	1->		93594
IPI00061009	403	403	1->	1->	93594
IPI00061009	413	403	1->		93594
IPI00062037	5	5		1->	140735
IPI00069084	2543	2543	1->	1->	8295
IPI00083708	394	389		->1	23215
IPI00099810	144	154	1->		51538
IPI00100160	55	36	1->	1->	55832
IPI00102661	305	269	1->		22974
IPI00102661	305	279	1->		22974
IPI00103483	519	525	1->		25920
IPI00103654	252	246	->1		151987
IPI00104050	221	215		->1	9967
IPI00104050	221	221	1->	1->	9967
IPI00104050	401	396	->1	->1	9967
IPI00104050	420	427	->1	->1	9967
IPI00104050	421	420	->1	->1	9967
IPI00104050	421	421	1->	1->	9967
IPI00104050	421	427	->1	->1	9967
IPI00104050	455	455	1->	1->	9967
IPI00104050	455	461		->1	9967
IPI00104050	519	527		->1	9967
IPI00104050	811	811	1->	1->	9967
IPI00107745	166	151	1->		51747
IPI00107745	233	231		->1	51747
IPI00143753	106	111	->1	->1	23350
IPI00143753	760	749	1->		23350
IPI00156649	477	475		->1	79930
IPI00159322	1267	1274	1->		6942
IPI00160622	1048	1036	->1		11190
IPI00162563	20	20	1->	1->	9810
IPI00164352	1117	1152	->1		23036
IPI00165189	290	258	1->	1->	28981

IPI00166153	108	114		->1	23070
IPI00168899	135	126	->1	->1	80174
IPI00170867	512	494	->1		1616
IPI00171903	277	285	1->		4670
IPI00173359	562	568		->1	55075
IPI00174345	156	148	->1	->1	200162
IPI00178611	304	304	1->	1->	55013
IPI00178611	304	306	->1	->1	55013
IPI00179709	40	40	1->	1->	113457
IPI00179851	123	112	->1		745
IPI00179851	123	123	1->	1->	745
IPI00180240	32	4	->1	->1	7117
IPI00180240	32	26	->1	->1	7117
IPI00180240	39	4	->1	->1	7117
IPI00180240	39	32	->1	->1	7117
IPI00180240	39	39	1->	1->	7117
IPI00180675	40	40	1->	1->	7846
IPI00182757	287	287	1->	1->	57805
IPI00183968	13	16	->1	->1	7170
IPI00183968	119	113	->1		7170
IPI00186224	141	144	->1	->1	4261
IPI00186224	141	156	1->		4261
IPI00186224	144	141		->1	4261
IPI00215911	3	7		->1	328
IPI00215911	6	7		->1	328
IPI00215965	144	183	->1	->1	3178
IPI00216047	326	328	->1		6601
IPI00216318	70	87	1->		7529
IPI00216318	117	87	1->		7529
IPI00216704	1258	1262	1->		6710
IPI00216704	1262	1262	1->	1->	6710
IPI00216975	118	112	->1		7171
IPI00216975	161	152	1->		7171
IPI00216975	161	198	1->		7171
IPI00216975	213	213	1->	1->	7171
IPI00216975	213	217	->1	->1	7171
IPI00216975	220	217	->1	->1	7171
IPI00216975	220	220	1->	1->	7171
IPI00216975	220	226	->1	->1	7171
IPI00217354	273	263	1->		55738
IPI00217732	538	539	->1	->1	406
IPI00217801	533	523	->1	->1	10014
IPI00217801	533	525		->1	10014
IPI00217801	533	537	->1	->1	10014
IPI00217975	123	145	1->	1->	4001
IPI00217975	124	145	1->	1->	4001
IPI00217975	157	145	1->	1->	4001
IPI00217975	157	156		1->	4001
IPI00217975	181	145	1->	1->	4001
IPI00217975	181	156	1->		4001
IPI00217975	182	145	1->	1->	4001
IPI00218343	40	40	1->	1->	84790

Table S5

IPI00218624	288	273		1->	6651
IPI00218624	288	288	1->	1->	6651
IPI00218728	53	88	1->	1->	5048
IPI00218823	2296	2296	1->	1->	9757
IPI00219005	274	266	1->		2288
IPI00219097	30	28		->1	3148
IPI00219097	30	29	->1	->1	3148
IPI00219097	59	57	->1	->1	3148
IPI00219097	59	68	->1	->1	3148
IPI00219097	139	114	1->		3148
IPI00219097	139	141		1->	3148
IPI00219097	157	114	1->		3148
IPI00219173	482	490		->1	9156
IPI00219365	388	400	->1		4478
IPI00219368	343	343	1->	1->	7090
IPI00219420	336	340	->1	->1	9126
IPI00219420	336	342	->1	->1	9126
IPI00219919	28	27		->1	55929
IPI00219919	35	27	->1	->1	55929
IPI00219919	35	28		->1	55929
IPI00219919	36	27	->1	->1	55929
IPI00219919	36	28	->1	->1	55929
IPI00219919	37	27	->1	->1	55929
IPI00219919	37	28		->1	55929
IPI00220484	82	82	1->	1->	10473
IPI00220827	4	4	1->	1->	9168
IPI00220827	17	26	->1	->1	9168
IPI00220827	39	39	1->	1->	9168
IPI00220828	4	4	1->	1->	7114
IPI00220828	12	32	->1		7114
IPI00220828	17	26	->1	->1	7114
IPI00220828	17	32	->1		7114
IPI00220828	26	32	->1		7114
IPI00220828	32	26	->1	->1	7114
IPI00220828	39	32	->1	->1	7114
IPI00220828	39	39	1->	1->	7114
IPI00221106	275	268	->1	->1	10992
IPI00234252	359	359	1->	1->	6599
IPI00234446	357	357	1->	1->	1386
IPI00234446	374	382		->1	1386
IPI00256605	302	302	1->	1->	51322
IPI00256605	302	306	1->		51322
IPI00289034	397	380	->1		84444
IPI00289034	398	380	->1		84444
IPI00289034	401	380	->1		84444
IPI00289344	1336	1366	1->		9611
IPI00289344	1412	1389	1->		9611
IPI00289773	265	275	->1	->1	1051
IPI00289776	2422	2409	1->	1->	23077
IPI00289776	2422	2420	->1	->1	23077
IPI00289819	2352	2355	->1	->1	3482
IPI00290204	118	87	1->		6625

Table S5

IPI00290547	1228	1231	->1	->1	4863
IPI00290548	119	122	->1	->1	1870
IPI00290548	119	127	->1	->1	1870
IPI00290548	122	127	->1	->1	1870
IPI00290548	127	122		->1	1870
IPI00290652	1339	1339	1->	1->	51773
IPI00291608	64	64	1->	1->	57092
IPI00291608	152	150	->1	->1	57092
IPI00291939	282	285	->1	->1	8243
IPI00291939	713	713	1->	1->	8243
IPI00292025	161	161	1->	1->	1050
IPI00292059	384	410	->1		9972
IPI00292059	1120	1120	1->	1->	9972
IPI00292537	432	442	->1	->1	4790
IPI00292537	441	441	1->	1->	4790
IPI00292537	441	442	->1	->1	4790
IPI00292537	441	449	->1	->1	4790
IPI00292537	442	441		1->	4790
IPI00292537	442	449	->1	->1	4790
IPI00292753	238	221	1->		26130
IPI00292753	238	233	1->		26130
IPI00292771	379	373	1->		4926
IPI00292771	379	379	1->	1->	4926
IPI00292771	379	386	1->		4926
IPI00292771	1511	1511	1->	1->	4926
IPI00292771	1511	1518	1->		4926
IPI00293331	46	40	->1	->1	10940
IPI00293331	46	46	1->	1->	10940
IPI00293845	1274	1264	->1	->1	55183
IPI00293845	1274	1266		->1	55183
IPI00293845	1274	1279	->1	->1	55183
IPI00294186	264	259	->1	->1	114294
IPI00294211	260	260	1->	1->	9785
IPI00294211	521	504	1->		9785
IPI00294211	521	510	1->		9785
IPI00294211	521	512	->1	->1	9785
IPI00294211	522	504	1->		9785
IPI00294211	522	510	1->		9785
IPI00294211	522	512	->1	->1	9785
IPI00294603	1282	1284	1->	1->	7750
IPI00294742	325	329		->1	51574
IPI00296337	2702	2703		->1	5591
IPI00296337	2703	2702		->1	5591
IPI00296830	297	287	->1	->1	7798
IPI00296830	297	289	->1	->1	7798
IPI00297931	513	506	1->	1->	11276
IPI00297931	513	524	1->	1->	11276
IPI00297931	744	733	->1		11276
IPI00298301	1877	1880		->1	4621
IPI00298520	274	282	->1	->1	372
IPI00299095	447	439	1->		6643
IPI00299095	469	439	1->		6643

Table S5

IPI00299147	11	5	->1	->1	6612
IPI00299147	11	7	->1	->1	6612
IPI00299147	11	11	1->	1->	6612
IPI00299149	11	7	->1	->1	6613
IPI00299149	11	11	1->	1->	6613
IPI00299263	228	236		->1	26286
IPI00299263	229	236		->1	26286
IPI00299417	24	24	1->	1->	8563
IPI00299463	441	443		->1	4683
IPI00299463	504	502		->1	4683
IPI00299463	544	568	->1	->1	4683
IPI00299463	665	653	1->	1->	4683
IPI00299463	690	652	->1	->1	4683
IPI00299463	698	652	->1	->1	4683
IPI00299465	259	244	1->		54585
IPI00299465	259	263		->1	54585
IPI00299465	259	264		->1	54585
IPI00299465	263	244	1->		54585
IPI00299465	263	259		->1	54585
IPI00299465	263	264		->1	54585
IPI00299608	310	310	1->	1->	5707
IPI00299608	310	319	1->		5707
IPI00300631	294	294	1->	1->	6294
IPI00300631	475	481	->1	->1	6294
IPI00300631	475	483	->1	->1	6294
IPI00301058	283	252	->1	->1	7408
IPI00301364	163	142	1->		6500
IPI00302829	896	896	1->	1->	5925
IPI00302829	896	900	->1	->1	5925
IPI00303402	112	105	->1	->1	51808
IPI00303402	183	184		->1	51808
IPI00303402	183	188	->1		51808
IPI00304527	418	417		->1	222584
IPI00304596	11	5	->1		4841
IPI00304596	64	60		->1	4841
IPI00304740	472	463	1->	1->	6453
IPI00304740	472	488	1->		6453
IPI00304740	472	489	1->		6453
IPI00304740	475	463	1->	1->	6453
IPI00304740	475	488	1->		6453
IPI00304740	475	489	1->		6453
IPI00304740	495	463	1->	1->	6453
IPI00304740	495	488	1->	1->	6453
IPI00304740	495	489	1->		6453
IPI00306043	245	281	1->		51441
IPI00306048	135	135	1->	1->	83858
IPI00306380	79	68	->1		26354
IPI00306380	79	75	->1		26354
IPI00306959	296	331	->1	->1	3855
IPI00328306	764	764	1->	1->	9877
IPI00328319	4	4	1->	1->	5928
IPI00328798	36	43	->1	->1	283149

Table S5

IPI00328798	36	49	->1	->1	283149
IPI00328798	137	137	1->	1->	283149
IPI00328918	33	21	1->		84062
IPI00329132	233	233	1->	1->	79969
IPI00329389	239	239		1->	6128
IPI00329791	263	256		->1	9879
IPI00333533	631	639		->1	367
IPI00333533	633	639	->1	->1	367
IPI00333533	634	639		->1	367
IPI00333533	634	659	1->		367
IPI00333541	994	982		1->	2316
IPI00333541	994	987		->1	2316
IPI00333913	1057	1063	1->		51594
IPI00335001	81	81	1->	1->	10169
IPI00337315	1571	1567	->1	->1	5930
IPI00337315	1571	1571	1->	1->	5930
IPI00337386	233	207	1->	1->	55660
IPI00374657	125	138	1->	1->	9218
IPI00375127	291	291	1->	1->	7458
IPI00375127	291	294		->1	7458
IPI00377245	2196	2196	1->	1->	8085
IPI00382470	346	346	1->	1->	3320
IPI00382470	414	414	1->	1->	3320
IPI00382470	414	416		->1	3320
IPI00382470	416	414	1->		3320
IPI00382470	436	414	1->	1->	3320
IPI00382470	668	668	1->	1->	3320
IPI00384028	657	666	->1	->1	10914
IPI00384028	666	673	->1	->1	10914
IPI00384028	752	731	1->		10914
IPI00384028	756	731	1->		10914
IPI00384202	552	545	1->	1->	221037
IPI00384857	8	8	1->	1->	51155
IPI00384972	180	180	1->	1->	284058
IPI00386448	310	303	->1	->1	5970
IPI00386448	310	314		->1	5970
IPI00386448	310	315		->1	5970
IPI00386448	314	315		->1	5970
IPI00386448	315	314		->1	5970
IPI00387159	164	165		->1	54556
IPI00387159	164	167	->1	->1	54556
IPI00387159	165	164		->1	54556
IPI00387159	165	167		->1	54556
IPI00395627	8	8	1->	1->	27101
IPI00395627	8	14		->1	27101
IPI00396008	582	582	1->	1->	144108
IPI00396089	85	85	1->	1->	8315
IPI00396154	296	303	1->		51105
IPI00397376	357	352	->1	->1	79882
IPI00397383	7	25	1->	1->	23095
IPI00398406	249	254	->1	->1	4205
IPI00398406	282	282	1->	1->	4205

Table S5

IPI00398406	403	403	1->	1->	4205
IPI00399266	168	201	->1	->1	7165
IPI00409671	25	26	->1	->1	11325
IPI00409671	25	30	->1	->1	11325
IPI00410039	235	240		->1	51535
IPI00410039	240	235		->1	51535
IPI00410351	1352	1382	1->		9611
IPI00410351	1428	1405	1->		9611
IPI00410590	291	294		->1	26065
IPI00410618	108	102		->1	57456
IPI00410693	140	145	->1		26135
IPI00411559	381	387	->1	->1	10051
IPI00411559	381	389	->1	->1	10051
IPI00411614	962	993	1->		11169
IPI00411614	1127	1120	1->	1->	11169
IPI00412441	253	253	1->	1->	23063
IPI00412441	689	682	1->		23063
IPI00413611	712	700	1->	1->	7150
IPI00414127	150	154	->1		5902
IPI00414127	183	173	->1	->1	5902
IPI00414127	183	183	1->	1->	5902
IPI00414676	284	286		->1	3326
IPI00414872	698	698	1->	1->	9701
IPI00418238	7	6		->1	3268
IPI00418238	173	173	1->	1->	3268
IPI00418471	139	143		->1	7431
IPI00419249	206	230	1->	1->	5684
IPI00419249	230	230	1->	1->	5684
IPI00419249	230	238	->1	->1	5684
IPI00419249	238	230	1->	1->	5684
IPI00419249	238	241	->1	->1	5684
IPI00419880	249	249		1->	6189
IPI00419979	38	38	1->	1->	5062
IPI00439548	392	395	->1	->1	54880
IPI00440484	1096	1096	1->	1->	5187
IPI00440727	1111	1114	1->	1->	23476
IPI00440727	1114	1114	1->	1->	23476
IPI00444646	647	640	1->	1->	11276
IPI00444646	647	658	1->	1->	11276
IPI00444646	878	867	->1		11276
IPI00446767	237	203		1->	7091
IPI00446767	237	237	1->	1->	7091
IPI00446767	281	287		->1	7091
IPI00446986	3	3	1->	1->	2533
IPI00446986	149	149	1->	1->	2533
IPI00448465	39	42	->1	->1	23253
IPI00449049	105	108		->1	142
IPI00449049	521	524		->1	142
IPI00455210	1671	1645	->1	->1	1108
IPI00455210	1671	1671	1->	1->	1108
IPI00455518	767	770	->1	->1	22880
IPI00455518	767	771	->1	->1	22880

Table S5

IPI00455623	136	166		->1	84779
IPI00456970	556	547		->1	8621
IPI00457284	1142	1134	->1	->1	23131
IPI00470779	194	188	1->	1->	200081
IPI00470779	195	188	1->	1->	200081
IPI00470891	434	434	1->		7812
IPI00470891	434	442	->1		7812
IPI00479545	470	470	1->	1->	55818
IPI00479545	470	485	1->	1->	55818
IPI00479571	33	21	1->		84062
IPI00479997	9	9	1->	1->	3925
IPI00479997	80	70	->1	->1	3925
IPI00479997	80	75	->1	->1	3925
IPI00479997	80	80	1->	1->	3925
IPI00479997	95	95	1->	1->	3925
IPI00479997	100	100	1->	1->	3925
IPI00479997	100	104	->1	->1	3925
IPI00479997	119	126		->1	3925
IPI00479997	128	126		->1	3925
IPI00514053	233	241	->1	->1	372
IPI00514648	604	596	1->	1->	6595
IPI00514648	1555	1564	->1	->1	6595
IPI00549205	270	236	->1	->1	10111
IPI00549205	270	279	->1	->1	10111
IPI00549248	229	248	->1		4869
IPI00549248	230	248	->1		4869
IPI00549248	257	248	->1	->1	4869
IPI00549248	267	248	->1		4869
IPI00549248	273	248	->1		4869
IPI00549955	377	377	1->	1->	55329
IPI00550021	366	399	->1	->1	6122
IPI00550021	373	399	->1	->1	6122
IPI00552897	812	812	1->	1->	9656
IPI00554579	443	443	1->	1->	256714
IPI00554648	347	347	1->	1->	3856
IPI00554648	347	352		->1	3856
IPI00554648	472	472	1->	1->	3856
IPI00554761	3	5		->1	643790
IPI00554761	14	5	->1		643790
IPI00604620	9	15	->1		4691
IPI00604620	9	16	->1		4691
IPI00604620	15	16	->1		4691
IPI00604620	572	577	1->		4691
IPI00604620	577	577	1->	1->	4691
IPI00641743	2050	2056		->1	3054
IPI00642550	649	649	1->	1->	728118
IPI00642971	383	425	->1		1936
IPI00642971	483	483	1->	1->	1936
IPI00643435	183	183	1->	1->	55210
IPI00643465	2884	2884	1->	1->	10579
IPI00643465	2884	2912	->1		10579
IPI00643722	997	974		1->	8289

Table S5

IPI00643722	1007	974		1->	8289
IPI00643722	1007	1007	1->	1->	8289
IPI00643722	1007	1010	1->		8289
IPI00643722	1612	1615	->1	->1	8289
IPI00643722	1905	1905	1->	1->	8289
IPI00643722	1905	1938	->1		8289
IPI00643920	6	6		1->	7086
IPI00643920	6	11		->1	7086
IPI00644055	103	114	1->		10236
IPI00644055	103	126	1->		10236
IPI00644712	539	542	->1		2547
IPI00644712	539	565	1->	1->	2547
IPI00644712	542	565	1->	1->	2547
IPI00644712	544	539		->1	2547
IPI00644712	544	542	->1	->1	2547
IPI00644712	544	553	->1	->1	2547
IPI00644712	544	565	1->	1->	2547
IPI00644712	553	565	1->	1->	2547
IPI00644712	556	553	->1	->1	2547
IPI00644712	556	556	1->	1->	2547
IPI00644712	556	565	1->	1->	2547
IPI00645616	530	540	->1	->1	414189
IPI00646058	475	481	->1	->1	6294
IPI00646058	475	483	->1	->1	6294
IPI00646645	1647	1647	1->	1->	23064
IPI00647217	51	61	1->	1->	23517
IPI00647217	51	79	1->		23517
IPI00647217	78	61	1->	1->	23517
IPI00647217	78	78	1->	1->	23517
IPI00647217	78	79	1->	1->	23517
IPI00647217	79	61	1->	1->	23517
IPI00647217	79	78	1->	1->	23517
IPI00647217	79	79	1->	1->	23517
IPI00657642	127	111	1->	1->	253635
IPI00657642	127	123	->1	->1	253635
IPI00737057	1024	1031	->1	->1	9498
IPI00737057	1024	1039	1->		9498
IPI00738216	1218	1211		->1	23379
IPI00738216	1218	1218	1->	1->	23379
IPI00738216	1218	1221	1->		23379
IPI00740961	175	175	1->	1->	26173
IPI00742682	312	290	1->		7175
IPI00742682	312	299	1->	1->	7175
IPI00742682	312	322	->1	->1	7175
IPI00742682	315	290	1->		7175
IPI00742682	315	299	1->	1->	7175
IPI00742682	315	322	->1	->1	7175
IPI00742682	345	340	->1	->1	7175
IPI00742682	345	364	1->		7175
IPI00742682	428	438		->1	7175
IPI00742682	457	474	->1		7175
IPI00743143	112	112	1->	1->	51147

Table S5

IPI00743509	168	171	->1	->1	1871
IPI00743509	168	176	->1	->1	1871
IPI00743509	171	176	->1	->1	1871
IPI00743509	176	171	->1	->1	1871
IPI00748532	619	616	->1	->1	8202
IPI00748532	620	616	->1	->1	8202
IPI00761080	154	154	1->	1->	23286
IPI00761160	712	712	1->	1->	831
IPI00782935	114	114		1->	84289
IPI00783017	412	417	1->		9839
IPI00783017	417	412	->1	->1	9839
IPI00783017	417	417	1->	1->	9839
IPI00784154	551	551		1->	3329
IPI00784154	554	554		1->	3329
IPI00784224	509	509	1->	1->	51663
IPI00785113	568	548	->1		9208
IPI00785113	568	606		1->	9208
IPI00785113	570	548	->1		9208
IPI00785113	570	606		1->	9208
IPI00790342	240	240		1->	6128
IPI00790503	1245	1240	->1	->1	4628
IPI00790503	1245	1247	->1	->1	4628
IPI00792984	45	45	1->	1->	10776
IPI00792984	45	50	->1	->1	10776
IPI00792984	45	52	->1	->1	10776
IPI00792984	50	52	->1	->1	10776
IPI00792984	128	121	->1		10776
IPI00793201	57	87	1->	1->	9255
IPI00794779	990	998	->1	->1	10721
IPI00797574	473	464	->1	->1	259282
IPI00797574	2430	2430	1->	1->	259282
IPI00797574	2430	2433	->1	->1	259282
IPI00797574	2430	2450	1->	1->	259282
IPI00798011	11	5	->1	->1	6612
IPI00798011	11	7	->1	->1	6612
IPI00798011	11	11	1->	1->	6612
IPI00807625	132	166	1->	1->	23048
IPI00815642	30	30	1->	1->	7114
IPI00815642	30	58	->1		7114
IPI00815642	38	58	->1		7114
IPI00815642	41	58	->1		7114
IPI00815642	52	58	->1	->1	7114
IPI00815642	58	52	->1	->1	7114
IPI00815642	65	58	->1	->1	7114
IPI00815642	65	65	1->	1->	7114
IPI00815707	1685	1662	->1		404734
IPI00815713	155	146		->1	6949
IPI00815713	155	159	->1	->1	6949
IPI00815713	296	292	->1		6949
IPI00815713	296	331	->1		6949
IPI00815713	600	600	1->	1->	6949
IPI00815713	755	746	->1	->1	6949

Table S5

IPI00815713	755	755	1->	1->	6949
IPI00816288	32	26	->1	->1	7115
IPI00816288	39	32	->1	->1	7115
IPI00816288	39	39	1->	1->	7115
IPI00816773	38	19	1->		84106
IPI00816773	38	34	->1	->1	84106
IPI00816773	38	35	->1	->1	84106
IPI00816773	43	19	1->		84106
IPI00816773	43	46	->1		84106
IPI00829826	1354	1382	->1		5469
IPI00829826	1502	1474	1->		5469
IPI00829826	1504	1474	1->		5469
IPI00844214	91	95		->1	56259
IPI00844214	91	102	1->	1->	56259
IPI00844508	579	577	1->		57530
IPI00844508	579	579	1->	1->	57530
IPI00844508	579	586		->1	57530
IPI00845355	967	970	->1	->1	546
IPI00845355	967	971	->1	->1	546
IPI00845508	222	228	->1	->1	57597
IPI00852685	1057	1083	1->		1729
IPI00852685	1103	1083	1->		1729
IPI00853077	910	917	1->		51105
IPI00853077	917	910	->1	->1	51105
IPI00853077	917	917	1->	1->	51105
IPI00854642	1211	1217	1->		23244
IPI00854642	1211	1219	1->		23244
IPI00855998	1673	1673	1->	1->	1063
IPI00855998	2875	2881	->1	->1	1063
IPI00856045	1510	1510	1->	1->	113146
IPI00867714	171	180	->1	->1	124801
IPI00872359	235	269	->1	->1	1639
IPI00873495	1126	1134	->1	->1	10721
IPI00873518	61	61	1->	1->	8379
IPI00873518	61	71	->1	->1	8379
IPI00876931	47	47	1->	1->	26173
IPI00878910	38	47	->1	->1	23338
IPI00878910	38	48		->1	23338
IPI00888429	527	527	1->	1->	283008
IPI00902533	317	309	1->	1->	9883
IPI00915324	118	112	->1		7168
IPI00915324	118	128	->1		7168
IPI00916332	758	761	->1	->1	58508
IPI00916332	2009	2009	1->	1->	58508
IPI00916332	2809	2802	->1	->1	58508
IPI00916332	2809	2814	->1	->1	58508
IPI00916332	2814	2809	->1		58508
IPI00916332	2814	2823	->1	->1	58508
IPI00916332	2832	2823	->1	->1	58508
IPI00916332	2832	2832	1->	1->	58508
IPI00916332	3714	3714	1->	1->	58508
IPI00916332	3714	3719	->1	->1	58508

Table S5

IPI00921844	264	303		1->	701
IPI00925255	28	28	1->	1->	222194
IPI00926625	272	265	->1	->1	7791
IPI00926625	272	279	->1	->1	7791
IPI00930688	40	40	1->	1->	10376
IPI00937615	212	220	->1	->1	64852
IPI00939238	51	51	1->	1->	861
IPI00939238	51	70	1->		861
IPI00939238	70	70	1->	1->	861
IPI00940437	135	145		->1	57511
IPI00940535	61	61	1->	1->	8379
IPI00940535	61	71	->1	->1	8379
IPI00940685	18	8	->1	->1	9410
IPI00941161	815	810	1->		8615
IPI00941161	815	815	1->	1->	8615
IPI00941161	815	821	1->		8615
IPI00941161	815	827	1->		8615
IPI00941972	40	41		->1	7008
IPI00942979	6	6		1->	7086
IPI00942979	6	11		->1	7086

Table S5

Symbol	Seq
COG2	--------meKsrmnlpkgpd
COG2	-meksrmnlpKgpdtlcfdkd
RPL5	vtpdmmeemyKkahaairenp
NFE2L2	veleqdldhlKdekekllkek
NFE2L2	veleqdldhlKdekekllkek
NFE2L2	vfsmlrdedgKpyspseyslq
NFE2L2	vfsmlrdedgKpyspseyslq
NFE2L2	vfsmlrdedgKpyspseyslq
NCOR2	aaeelavdtgKaeepvksect
NCOR2	vdtgkaeepvKsecteeaeeg
NCOR2	cteeaeegpaKgkdaeaaeat
NCOR2	sdphrektqsKpfsiqelelr
HTT	gsscspvlsrKqkgkvllgee
HTT	scspvlsrkqKgkvllgeeea
XRCC1	lnqeekktpsKppaqlspsvp
XRCC1	rkldlnqeekKtpskppaqls
HSPA4	enegkmimqdKlekerndakn
HSPA4	gkmimqdkleKerndaknave
HSPA5	ayslknqigdKeklggklsse
HSPA5	nelesyayslKnqigdkeklg
HSPA5	slknqigdkeKlggklssedk
HSPA5	klggklssedKetmekaveek
HIF1A	sdmkmtqlftKvesedtsslf
HIF1A	dtsslfdklkKepdaltllap
IFI16	spapstsstvKtegaeatpga
ARHGDIB	eeddddeldsKlnykpppqks
ARHGDIB	sklnykpppqKslkelqemdk
ARHGDIB	sklnykpppqKslkelqemdk
ARHGDIB	nykpppqkslKelqemdkdde
HSPA8	kenkititndKgrlskedier
HSPA8	titndkgrlsKediermvqea
HSPA8	diermvqeaeKykaedekqrd
HSPA8	ermvqeaekyKaedekqrdkv
HSPA8	atvedeklqgKindedkqkil
HSPA8	titndkgrlsKediermvqea
HSPA8	eaekykaedeKqrdkvsskns
HSPA8	atvedeklqgKindedkqkil
HSPA8	ermvqeaekyKaedekqrdkv
HSPA8	ykaedekqrdKvssknslesy
HSPA8	atvedeklqgKindedkqkil
HSPA8	atvedeklqgKindedkqkil
MKI67	psagkamltpKpaggdekdik
MKI67	psagkamltpKpaggdekdik
MKI67	ltpkpaggdeKdikafmgtpv
MKI67	lktslgkvgvKeellavgklt
MKI67	tddkitevscKspqpdpvktp
E2F1	pgkgvkspgeKsryetslnlt
E2F1	pgkgvkspgeKsryetslnlt
SAFB2	skadsllavvKrepaeqpgdg
PFDN6	iqkklqgeveKyqqlqkdlsk
PFDN6	gevekyqqlqKdlsksmsgrq

Table S5

SHOX	fvsksfdqksKdgnggggggg
BCLAF1	ypdggdqetaKtgkflkrftd
BCLAF1	rnteeeglkyKskvslkgnre
DMD	sglyylsttvKemskkapsei
MAML1	hslgldslnkKrladsslhln
MAML1	hqlqqiaakqKreqmlqnpqq
MAML1	sahqlqqiaaKqkreqmlqnp
TP73	eqqalnessaKngaaskrafk
TP73	eqqalnessaKngaaskrafk
HGS	yeqlnrkaegKatsttelppe
PCM1	psaserlpdeKvelfskmrvl
AIMP1	aklkkeieelKqeliqaeiqn
NOP58	stlptcskkrKieqvdkedei
CHGB	psdsqvseevKtrhseksqre
CHGB	seevktrhseKsqredeeeee
PIN4	aasgsdsadkKaqgpkgggna
DDX24	psslfskeapKrkaqavseee
YARS	gehvfvkgyeKgqpdeelkpk
LEF1	dkarehpddgKhpdgglynkg
MAFG	krvtqkeeleKqkaelqqeve
MAFG	krvtqkeeleKqkaelqqeve
MAFG	vtqkeelekqKaelqqevekl
ACIN1	qrlqpergspKkceaeeaepp
DYNC1LI1	asvspipagsKkidpnmkaga
DYNC1LI1	agskkidpnmKagatsegvla
HSPA9	iviqssgglsKddienmvkna
HSPA9	lskddienmvKnaekyaeedr
HSPA9	iviqssgglsKddienmvkna
HSPA9	aegiihdtetKmeefkdqlpa
HSPA9	qlpadecnklKeeiskmrell
HSPA9	aslklfemayKkmaseregsg
SMC2	eeitptiqklKeerssyleyq
SMC2	ntksqsafdlKkknlaceesk
SMC2	knlaceeskrKeleknmveds
HIC1	lpslpplpfqKleeaappsdp
BRPF3	reqdektsavKeelkywqklr
TPX2	dfhfrtderiKqhpknqeeyk
TPX2	kqhpknqeeyKevnftselrk
SSB	kplpevtdeyKndvknrsvyi
SSB	gkvqfqgkktKfasddehdeh
MLL	edaeplappiKpikpvtrnka
EIF4A3	tsgsarkrllKeedmtkvefe
SOX9	ntfpkgepdlKkeseedkfpv
SOX9	qgpptppttpKtdvqpgkadl
SOX9	epgqsqrthiKteqlspshys
PDIA4	naaildesgkKfamepeefds
IMMT	evaarlaqqeKqeqvkiesla
IMMT	laqqekqeqvKieslaksled
IMMT	lalekqkleeKrafdsavaka
IMMT	dvlrvqeqelKsefeqnlsek
PLAGL2	qlgstsylpdKlpkvevdsfl
PLAGL2	stsylpdklpKvevdsflael

Table S5

IFNAR2	ellsgpcerrKsplqdpfpee
NRIP1	lqqysrehalKtqnanqaase
NRIP1	amarlqengqKdvgsyqlpkg
NRIP1	tqsllntwdpKvpdvdikedq
NRIP1	wdpkvpdvdiKedqdtsknsk
NRIP1	vtllqlllghKneenveknts
NRIP1	vtllqlllghKneenveknts
NRIP1	snhsmdltksKdppgekpaqn
TRIM33	scgssgrtaeKtslsfksdqv
TRIM33	rtaektslsfKsdqvkvkqep
TRIM33	tslsfksdqvKvkqepgtede
ACOT7	srqeqeeegrKryeaqklerm
ACOT7	srqeqeeegrKryeaqklerm
TMF1	eftqriaeaeKkvqlackerd
BAT2	kpptgplppsKeplkeklipg
SFPQ	qlddedglpeKlaqknpmyqk
SFPQ	edglpeklaqKnpmyqkeret
CHAF1B	tprrinltplKtdtppssvpt
IK	eiererelaeKyrdrakerrd
IK	eekkrhsyfeKpkvddepmdv
IK	kkrhsyfekpKvddepmdvdk
EIF4B	qdsrsapepkKpeenpaskfs
MPHOSPH1(	lekssvdqagKysktvasekl
MPHOSPH1(	ssvdqagkysKtvaseklkql
BCL6	ivlnslnqnaKpegpeqaelg
CDC37	dseevreqkhKtfvekyekqi
PDAP1	mhlagkteqaKadlarlaiir
EPRS	vnvpeaqeemKevakhpknpe
EPRS	daavkqllslKaeykektgqe
EPRS	kqllslkaeyKektgqeykpg
SNW1	dlqrpdeeaiKeitektrval
STIP1	eakrtyeeglKheannpqlke
TPM2	aqerlatalqKleeaekaade
UBTF	edmltllecmKnnlpsndssk
SARNP	vptkglssdnKpmvnldklke
SARNP	sdnkpmvnldKlkeraqrfgl
SHROOM2	kakektvedlKseelareivg
DOCK1	cfkqlkekveKeygvrimpss
CHCHD3	qleekdrvlkKqdafykeqla
DNAJB1	rrqalryhpdKnkepgaeekf
DNAJB1	qalryhpdknKepgaeekfke
DNAJB1	dknkepgaeeKfkeiaeaydv
DNAJB1	nkepgaeekfKeiaeaydvls
DNAJB1	qalryhpdknKepgaeekfke
DNAJB1	dknkepgaeeKfkeiaeaydv
DNAJB1	nkepgaeekfKeiaeaydvls
OCIAD1	sppghyyqksKydssvsgqss
RPS6KA1	lqpskdegvlKeisithhvka
SF3A1	erkkeeeekeKervayaqidw
LETM1	tgeekyveesKaskrltkrvq
RCSD1	fkvkssplieKlqanltfdpa
ACO2	tdyltgtdgkKfrleapdade

Table S5

NCOA2	ecpdqlgpspKrntekrnreq
NCOA2	srlhdskgqtKllqllttksd
NCOA2	adgqsrlhdsKgqtkllqllt
NCOA2	pasntkliamKtekeemsfep
NCOA2	dsktdpasntKliamktekee
NCOA2	pasntkliamKtekeemsfep
NCOA2	ntkliamkteKeemsfepgdq
EPC1	etntsdksfsKdlsqilvnik
BRD8	vplpapemtvKqerldfeete
BRD8	erldfeetenKgihelvdire
NCBP1	leeqierlqeKvesaqseqkn
MYH9	dqncklakekKlledriaeft
MYH9	fttnlteeeeKskslaklknk
MYH9	eekskslaklKnkheamitdl
MYH9	eeeeakhnleKqiatlhaqva
MYH9	leglsqrheeKvaaydklekt
MYH9	ekvaaydkleKtktrlqqeld
MYH9	llaeektisaKyaeerdraea
MYH9	narqqlerqnKelkvklqeme
MYH9	ykasitaleaKiaqleeqldn
MYH9	qleeqldnetKerqaackqvr
PPP5C	dgalkraeelKtqandyfkak
PPP5C	dgalkraeelKtqandyfkak
HCFC1	qetskdssgtKpankrpmssp
ARHGAP1	wpsdempdfpKsddsksssspe
CALR	dwdflppkkiKdpdaskpedw
CALR	leddwdflppKkikdpdaskp
CALR	eddwdflppkKikdpdaskpe
CALR	rakiddptdsKpedwdkpehi
EP300	slgmvqdaasKhkqlsellrs
EP300	gmvqdaaskhKqlsellrsgs
EP300	slgmvqdaasKhkqlsellrs
EP300	gmvqdaaskhKqlsellrsgs
EP300	snnlspfamdKkavpgggmpn
EP300	snnlspfamdKkavpgggmpn
EP300	nnlspfamdkKavpgggmpnm
EP300	tevnsqaiaeKqpsqevkmea
EP300	dtqpedisesKvedckmeste
EP300	tevnsqaiaeKqpsqevkmea
EP300	iaekqpsqevKmeakmevdqp
EP300	dtqpedisesKvedckmeste
EP300	qpsqevkmeaKmevdqpepad
EP300	dtqpedisesKvedckmeste
EP300	dtqpedisesKvedckmeste
EP300	dtqpedisesKvedckmeste
EP300	erstelkteiKeeedqpstsa
EP300	dpsqpqttinKeqfskrkndt
EP300	qttinkeqfsKrkndtldpel
EP300	tinkeqfskrKndtldpelfv
EP300	fwpnvleesiKeleqeeeerk
EP300	fwpnvleesiKeleqeeeerk
CDK12	nsekhlvkdlKaqgtrdskpi

Table S5

CDK12	dlkaqgtrdsKpialkeeivt
CDK12	gtrdskpialKeeivtpkete
YWHAZ	ieqktegaekKqqmareyrek
LMNA	ayeaelgdarKtldsvakera
LMNA	darktldsvaKerarlqlels
LMNA	darktldsvaKerarlqlels
LMNA	erarlqlelsKvreefkelka
LMNA	lelskvreefKelkarntkke
LMNA	darktldsvaKerarlqlels
LMNA	erarlqlelsKvreefkelka
LMNA	skvreefkelKarntkkegdl
LMNA	etrlveidngKqrefesrlad
LMNA	qhedqveqykKelektysakl
LMNA	kkelektysaKldnarqsaer
LMNA	aqhedqveqyKkelektysak
LMNA	qveqykkeleKtysakldnar
LMNA	aldmeihayrKllegeeerlr
FGA	ynrgdstfesKsykmadeags
FGA	ynrgdstfesKsykmadeags
PSMC6	----madprdKalqdyrkkll
PSMC6	prdkalqdyrKkllehkeidg
PSMC6	qdyrkkllehKeidgrlkelr
PSMC6	rdkalqdyrkKllehkeidgr
KAT2B	ssgleanpgeKrkmtdshvle
KAT2B	gleanpgekrKmtdshvleea
PML	speqprpstsKavspphldgp
ROCK1	evkhlkhnleKvegerkeaqd
ROCK1	ksvamcemekKlkeerearek
ROCK1	vamcemekklKeerearekae
ROCK1	tdkhqsieeaKsvamcemekk
CCNA2	aftihvdeaeKeaqkkpaesq
CCNA2	vdeaekeaqkKpaesqkiere
CCNA2	eaqkkpaesqKieredalafn
MYST3	qereleisvgKsvshenkeqd
SBNO1	vrnamtsapsKdqvqlkdllk
SBNO1	sapskdqvqlKdllknnslne
GLTSCR1	asnlptlnvaKaassgpgkps
KIF15	lekqlqetqtKndflksevhd
KIF15	sleksrdsdkKvvadlmnqiq
TP53	rrteeenlrkKgephhelppg
TP53	phhelppgstKralpnntsss
TP53	nntssspqpkKkpldgeyftl
TP53	ntssspqpkkKpldgeyftlq
TP53	pnntssspqpKkkpldgeyft
TP53	ntssspqpkkKpldgeyftlq
TP53	pnntssspqpKkkpldgeyft
TP53	nntssspqpkKkpldgeyftl
TP53	stsrhkklmfKtegpdsd---
TP53	stsrhkklmfKtegpdsd---
SP3	nadspadiriKeeepdpeewq
SF3B1	erldpfadggKtpdpkmnart
SF3B1	fadggktpdpKmnartymdvm

Table S5

SF3B1	laekakagelKvvngaaasqp
SF3B1	etptpgaskrKsrwdetpasq
HCLS1	rfgverdrmdKsavgheyvae
HCLS1	vgheyvaeveKhssqtdaakg
HCLS1	kygverdradKsavgfdykge
HCLS1	emeapttaykKttpieaassg
HCLS1	kakfesmaeeKrkreeeekaq
HCLS1	kfesmaeekrKreeeekaqqv
HCLS1	eekrkreeeeKaqqvarrqqe
GMNN	qkqeeikeniKnssvprrtlk
RANBP3	--madlaneeKpaiappvfvf
RANBP3	appvfvfqkdKgqkspaeqkn
RANBP3	vfvfqkdkgqKspaeqknlsd
RANBP3	aiappvfvfqKdkgqkspaeq
RANBP3	vfvfqkdkgqKspaeqknlsd
RANBP3	dkgqkspaeqKnlsdsgeepr
USF1	seaprttrdeKrraqhnever
NUP50	fasakaaadpKvafgslaang
NUP50	atsasfnfgkKvdssvlgsls
NUP50	atsasfnfgkKvdssvlgsls
HSP90B1	kegvkfdeseKtkesreavek
HSP90B1	ktkesreaveKefepllnwmk
HNRNPCL1	---masnvtnKmdphsmnsrv
HNRNPL	seggrapkrlKtdnagdqhgg
CTCF	veesetfikgKerktyqrrre
CTCF	aiveesetfiKgkerktyqrr
PRKCQ	mchlpepelnKerpslqiklk
ZRANB2	rektteakmmKaggteigktl
DCTN1	qleqvqewksKmqeqqadlqr
CTTN	envfqehqtlKekeletgpka
CTTN	kfgveqdrmdKsavgheyqsk
CTTN	ksavgfdyqgKtekhesqrdy
CTTN	ksavgfdyqgKtekhesqrdy
CTTN	ksavgfeyqgKtekhesqkdy
CTTN	ksavgfeyqgKtekhesqkdy
CTTN	ksavgfeyqgKtekhesqkdy
TRIM25	eqlqqeytemKalldasetts
TRIM25	ldasettstrKikeeekrvns
EXOSC9	apidtsdveeKaeeiiaeaep
SF3A3	yedssgnvvnKktyedlkrqg
SF3A3	edssgnvvnkKtyedlkrqgl
NFIC	stsssgskrhKsgsmeedvdt
CCNT1	mrikvhaaadKhnsvedsvtk
CCNT1	khnsvedsvtKsrehkekhkt
CCDC55	makdkernqeKpsnsesslga
CCDC55	psnsesslgaKhrlteegqek
CCDC55	rlteegqekgKeqerppeavs
TRAP1	stesvqgstsKhefqaetkkl
USP8	dsgsgkpfkiKgqpesgilrt
DNMT1	itshfakgpaKrkpqeesera
POM121C	mfkpiftappKsekegptppg
POM121C	piftappkseKegptppgpsv

Table S5

POM121C	mfkpiftappKsekegptppg
ETV3	prikvepaseKdpeslrqsar
SNAP29	fgglvnyfksKpvetppeqng
SNCB	akegvvaaaeKtkqgvteaae
SNCB	egvvaaaektKqgvteaaekt
SNCB	akegvvaaaeKtkqgvteaae
SNCB	egvvaaaektKqgvteaaekt
MYC	eklasyqaarKdsgspnparg
VSX2	gkagealskpKsetvakstsg
KIAA1429	qpqpslkrnpKhadgekedqf
PPP1R9B	dyqqkeieflKketaqrrvle
PPP1R9B	yqqkeieflkKetaqrrvlee
SPEN	taagggpqgkKgknepkvdat
SPEN	pqgkkgknepKvdatrpeatt
SPEN	pqgkkgknepKvdatrpeatt
SPEN	taagggpqgkKgknepkvdat
SPEN	pqgkkgknepKvdatrpeatt
EFHD2	eerkkqaeemKqrkaafkelq
EVI5L	tlgalremqdKvldmekrnss
WDR67	kqdltgdfesKknelpdglnk
WDR67	qdltgdfeskKnelpdglnkk
WDR67	qdltgdfeskKnelpdglnkk
DYNLL2	------msdrKaviknadmse
TRRAP	raafamvthvKqeprerense
BAT2L2	sqipaqpsvaKvpygkgpsfn
ZCCHC17	kdcfmqpggtKyslipdeeee
CAND1	atndlmtelqKdsikldddse
TPX2	dfhfrtderiKqhpknqeeyk
TPX2	kqhpknqeeyKevnftselrk
COBRA1	vapsklealqKaleptgqsge
PPP4R2	daveaeghevKrlrfdkegev
THRAP3	tfsggtsqdtKasesskpwpd
THRAP3	sqdtkasessKpwpdatygtg
THRAP3	gkmksdsfapKtdsekpfrgs
THRAP3	dfekkmadfhKeemddqdkdk
THRAP3	kryklrddfeKkmadfhkeem
THRAP3	ryklrddfekKmadfhkeemd
THRAP3	dfekkmadfhKeemddqdkdk
THRAP3	efddepkfmsKviganknqee
THRAP3	kfmskviganKnqeeeksgkw
THRAP3	peknfrvtayKavqekssspp
THRAP3	ereesttgfdKsrlgtkdfvg
LUC7L3	qqieelgsegKveeaqgmmkl
LUC7L3	akikatveelKeklrkrteep
SR140	qeelkkkedeKaaaeiyeefl
SR140	vpldatedskKnepifkvaps
DOK3	tgrpdpqagfKaklvtllsre
TCF20	ipskrqsqdvKnsstedkgrl
CEP250	qrlveqevqeKlretqeynri
RNF40	agdggsgppeKklsreekttt
ZNF292	annlntpnngKfvyflpspvn
IFT81	ksmrqaaadaKpeslmkrlee

Table S5

FTSJD2	eglgkysqgrKdiveassqkg
DBF4B	rvetsamvdpKgshprpsrkp
DAXX	eeaaagkdgdKspmsslqisn
HNRNPM	hvkmderalpKgdffpperpq
UACA	aevgklrnqiKqnemiveefk
SPAG17	fkdqqrreneKkviedkpkle
CCDC109B	qqhfdvqqynKlkedlakake
CCDC109B	hfdvqqynklKedlakakesl
TUBA3D	iqpdgqmpsdKtigggddsfn
C11orf9	nnnngmgaapKpfpggtgppi
C11orf9	pfpggtgppiKaepkapyapg
TMSL3	-------msdKpdmaeiekfd
TMSL3	pklkktetqeKnplpsketie
TMSL3	-------msdKpdmaeiekfd
TMSL3	etqeknplpsKetieqekqag
TMSL3	lpsketieqeKqages-----
TUBA1A	iqpdgqmpsdKtigggddsfn
KIAA1967	hpsriqvsseKeaapdagaep
TPM3	kkkmqmlkldKenaldraeqa
TPM3	aqerlatalqKleeaekaade
CIITA	mpaevgqksqKrpfpeelpad
CIITA	pfpeelpadlKhwkpaepptv
CIITA	smempaevgqKsqkrpfpeel
APEX1	----mpkrgkKgavaedgdel
APEX1	----mpkrgkKgavaedgdel
HNRNPA1	hncevrkalsKqemasasssq
SMARCC2	kgpstpytksKrghreeeqed
YWHAB	kqqmgkeyreKieaelqdicn
YWHAB	kqqmgkeyreKieaelqdicn
SPTB	iedrhrknneKaqeasvllrd
SPTB	iedrhrknneKaqeasvllrd
TPM4	aqerlatalqKleeaekaade
TPM4	eiqemqlkeaKhiaeeadrky
TPM4	lkcgdleeelKnvtnnlksle
TPM4	nlksleaaseKysekedkyee
TPM4	leaasekyseKedkyeeeikl
TPM4	leaasekyseKedkyeeeikl
TPM4	asekysekedKyeeeikllsd
TPM4	ekedkyeeeiKllsdklkeae
ARFGAP1	lghslnenvlKpaqekvkegk
ARNTL	ppdasspggkKilnggtpdip
HDAC5	mqqqhqqfleKqkqqqlqlgk
HDAC5	qqhqqflekqKqqqlqlgkil
HDAC5	qqlqlgkiltKtgelprqptt
LMNB1	lreyeaalnsKdaalatalgd
LMNB1	lreyeaalnsKdaalatalgd
LMNB1	lreyeaalnsKdaalatalgd
LMNB1	daalatalgdKkslegdledl
LMNB1	lreyeaalnsKdaalatalgd
LMNB1	daalatalgdKkslegdledl
LMNB1	lreyeaalnsKdaalatalgd
TUBA1C	iqpdgqmpsdKtigggddsfn

Table S5

SON	vvtmsveyqmKsvlksvests
SON	svestspepsKimlveppvak
PAFAH1B1	eftsggplgqKrdpkewiprp
MLL4	grsppapppyKaprldedgea
FKBP4	keswemnseeKleqstivker
HMGB2	ffvqtcreehKkkhpdssvnf
HMGB2	fvqtcreehkKkhpdssvnfa
HMGB2	erwktmsakeKskfedmaksd
HMGB2	skfedmaksdKarydremkny
HMGB2	lfcsehrpkiKsehpglsigd
HMGB2	emwseqsakdKqpyeqkaakl
HMGB2	lfcsehrpkiKsehpglsigd
EXO1	rnkfatflqrKneesgavvvp
MSN	akerqeaeeaKeallqasrdq
TLE3	ttpglrsmpgKppgmdpigim
SMC3	rqkllekieeKqkelaetepk
SMC3	kllekieekqKelaetepkfn
DMAP1	egdaasgtisKkdiinpdkkk
DMAP1	egdaasgtisKkdiinpdkkk
DMAP1	gdaasgtiskKdiinpdkkks
DMAP1	egdaasgtisKkdiinpdkkk
DMAP1	gdaasgtiskKdiinpdkkks
DMAP1	egdaasgtisKkdiinpdkkk
DMAP1	gdaasgtiskKdiinpdkkks
HMGN4	nrdastlqsqKaegtgdak--
TMSB10	-------madKpdmgeiasfd
TMSB10	aklkktetqeKntlptketie
TMSB10	lptketieqeKrseis-----
TMSB4X	-------msdKpdmaeiekfd
TMSB4X	etqeknplpsKetieqekqag
TMSB4X	sklkktetqeKnplpsketie
TMSB4X	etqeknplpsKetieqekqag
TMSB4X	etqeknplpsKetieqekqag
TMSB4X	sklkktetqeKnplpsketie
TMSB4X	etqeknplpsKetieqekqag
TMSB4X	lpsketieqeKqages-----
SF3B2	remddpsvgpKipqalekilq
SMARCC1	aslygkrrsqKeedeqedltk
ATF2	anedpdekrrKflernraaas
ATF2	krkvwvqsleKkaedlsslng
WAC	ptptssvpaqKterkestsgd
WAC	ssvpaqkterKestsgdkpvs
DOT1L	apmdsgaeeeKagaatvkkps
DOT1L	apmdsgaeeeKagaatvkkps
DOT1L	apmdsgaeeeKagaatvkkps
NCOR1	rqdiltqesrKtpevvqstrp
NCOR1	egsisqgtpiKfdnnsgqsai
CEBPB	ktvdkhsdeyKirrernniav
MYCBP2	glevkvkdppKgmippgtqlv
MYCBP2	gmippgtqlvKpksepqpnkv
IGF2R	rssnvsykysKvnkeeetden
SNRNP70	rrqqevetelKmwdphndpna

Table S5

NPAT	lsvgtavkdlKqeqtksassl
E2F2	vdglpspktpKspgektrydt
E2F2	spktpkspgeKtrydtslgll
E2F2	spktpkspgeKtrydtslgll
E2F2	vdglpspktpKspgektrydt
RSF1	vlpseqestkKpyriesdeee
PCNP	aeeeaadlptKptkiskfgfa
PCNP	ptsagpnsfnKgkhgfsdnqk
SMC1A	reqqqiekeiKekdselnqkr
SMC1A	sqahglqmrlKysqsdleqtk
CEBPA	gapalrplviKqepreedeak
NUP153	idnkcstgyeKnmtpgqnreq
NUP153	pvtstslvfgKkadneepkcq
NFKB1	khgtmdteskKdpegcdksdd
NFKB1	mkhgtmdtesKkdpegcdksd
NFKB1	khgtmdteskKdpegcdksdd
NFKB1	eskkdpegcdKsddkntvnlf
NFKB1	mkhgtmdtesKkdpegcdksd
NFKB1	eskkdpegcdKsddkntvnlf
GAPVD1	dedhletdpnKlierfspsqq
GAPVD1	ierfspsqqeKlfgekgsdrf
NUMA1	ekelsaalqdKkcleekneil
NUMA1	alqdkkcleeKneilqgklsq
NUMA1	leekneilqgKlsqleehlsq
NUMA1	tarelevmtaKyegakvkvle
NUMA1	mtakyegakvKvleerqrfqe
POP1	rgvkhhsggeKpfqaqkqeph
POP1	sggekpfqaqKqephpgtsrq
RIF1	etmiktdflpKakqregtfsk
RIF1	miktdflpkaKqregtfsksd
RIF1	egtfsksdseKivngtkrssr
LACTB	eqekegksneKndftkfkteq
DHX38	trdrdrsvrgKysddtplptp
DHX38	dgkvdyrteqKfadhmkrkse
DHX38	rteqkfadhmKrkseassefa
DHX38	eqkfadhmkrKseassefakk
DHX38	dgkvdyrteqKfadhmkrkse
DHX38	rteqkfadhmKrkseassefa
DHX38	eqkfadhmkrKseassefakk
ZMYM2	nedkittgkrKheddepvfeq
LARP7	vkkiiqkdiiKeaseaskenr
PRKDC	lksvgpdfgkKrlglpgdevd
PRKDC	plksvgpdfgKkrlglpgdev
LUZP1	neknrnqednKvkdlnqeiek
LUZP1	knrnqednkvKdlnqeieklk
SYNRG	psllmplpgtKalpsmdkyav
SYNRG	yavfkgiaadKssentvppgd
SYNRG	pltsnvgstvKggqnstaast
MYH3	dklqvkvksyKrqaeeadeqa
ARCN1	kgkevdnfvdKlksegetims
SNX2	eaeakmmvanKpdkiqqakne
SNX2	eaeakmmvanKpdkiqqakne

Table S5

SUMO3	------mseeKpkegvktend
SUMO3	----mseekpKegvktendhi
SUMO3	mseekpkegvKtendhinlkv
SUMO2	----madekpKegvktenndh
SUMO2	madekpkegvKtenndhinlk
ARFGAP3	gakkgslgaqKlantcfneie
ARFGAP3	gakkgslgaqKlantcfneie
THOC5	irsdgapaegKrnrsdteqeg
NBN	ptklpsinksKdrasqqqqtn
NBN	qtqpatpslwKnkeqhlsene
NBN	iedevleqlfKdtkpeleidv
NBN	lqddsemlpkKllltefrslv
NBN	klqddsemlpKklllltefrsl
NBN	klqddsemlpKklllltefrsl
LZTFL1	ksleenlataKhdllrvqeql
LZTFL1	qlhmaekeleKkfqqtaayrn
LZTFL1	lhmaekelekKfqqtaayrnm
LZTFL1	ksleenlataKhdllrvqeql
LZTFL1	rvqeqlhmaeKelekkfqqta
LZTFL1	lhmaekelekKfqqtaayrnm
PSMD1	eektssafvgKtpeaspepkd
PSMD1	gktpeaspepKdqtlkmikil
SAFB	skadsllavvKrepaeqpgdg
SAFB	elhgkmisveKaknepvgkkt
SAFB	hgkmisvekaKnepvgkktsd
VASP	eeasggptapKaesgrsgggg
SKP1	peeirktfniKndfteeeeaq
RB1	dgskhlpgesKfqqklaemts
RB1	hlpgeskfqqKlaemtstrtr
PHAX	epfqfgqssqKppvaggkkin
PHAX	eldeymhggkKmgskeeengq
PHAX	ymhggkkmgsKeeengqghlk
FAM83B	nrtnnppgnwKkpsdslsvas
NONO	------mqsnKtfnlekqnht
NONO	sqnegltidlKnfrkpgektf
ITSN1	rrqellnqrnKeqedivvlka
ITSN1	lefelealndKkhqlegklqd
ITSN1	efelealndkKhqlegklqdi
ITSN1	rrqellnqrnKeqedivvlka
ITSN1	lefelealndKkhqlegklqd
ITSN1	efelealndkKhqlegklqdi
ITSN1	rrqellnqrnKeqedivvlka
ITSN1	lefelealndKkhqlegklqd
ITSN1	efelealndkKhqlegklqdi
YTHDF2	hnmdigtwdnKgpvakapsqa
ATAD3B	qhqaraqyqdKlarqryedql
GNL3	eallreaelrKqrleelkqqq
GNL3	elrkqrleelKqqqkldrqke
KRT7	eidniknqraKleaaiaeaee
ZC3H11A	trrlssastgKpplsveddfe
RBBP4	-------madKeaafddavee
BCL9L	apakpmhpenKltnhgktgng

Table S5

BCL9L	hpenkltnhgKtgnggaqsqh
BCL9L	agtpsldseaKevaprskrrc
DTNBP1	svqqdftsglKtlsdksreak
C6orf134	lppkraegdiKpysssdrefl
RPL6	egeifdtekeKyeiteqrkid
DDX46	svkggggnekKsgptvtkvvt
AR	arklkklgnlKlqeegeasst
AR	arklkklgnlKlqeegeasst
AR	arklkklgnlKlqeegeasst
AR	ttspteettqKltvshiegye
FLNA	skikvsglgeKvdvgkdqeft
FLNA	sglgekvdvgKdqeftvkskg
NBAS	hglekpisfvKntqssseear
SERF2	gperppekprKhdsgaadler
RBBP6	etksvdknpcKdrekhvlear
RBBP6	vdknpckdreKhvlearnnke
PRPF40A	ntetkqstweKpddlktpaeq
VAPA	cvfempnendKlgitppgnap
EIF4H	garpreevvqKeqk-------
EIF4H	preevvqkeqK----------
MLL2	qsrdpfaplhKpprpqppeva
HSP90AA1	igypitlfveKerdkevsdde
HSP90AA1	ekyidqeelnKtkpiwtrnpd
HSP90AA1	yidqeelnktKpiwtrnpddi
HSP90AA1	ekyidqeelnKtkpiwtrnpd
HSP90AA1	ekyidqeelnKtkpiwtrnpd
HSP90AA1	fegktlvsvtKeglelpedee
PAPOLA	tkiptpivgvKrtssphkees
PAPOLA	vgvkrtssphKeespkktkte
PAPOLA	qtaasllasqKtsstdlsdip
PAPOLA	qtaasllasqKtsstdlsdip
JMJD1C	qkmdpnvsdsKhsianakfle
HN1	---mtttttfKgvdpnsrnss
KIAA1267	hdnstslnggKraltssalhg
RELA	ddrhrieekrKrtyetfksim
RELA	rtyetfksimKkspfsgptdp
RELA	tyetfksimkKspfsgptdpr
RELA	tyetfksimkKspfsgptdpr
RELA	rtyetfksimKkspfsgptdp
ING3	htttdhipekKfkseallstl
ING3	ttdhipekkfKseallstlts
ING3	hhtttdhipeKkfkseallst
ING3	ttdhipekkfKseallstlts
CACYBP	---maseelqKdleevkvlle
CACYBP	eelqkdleevKvllekatrkr
SPTY2D1	pqrlpfptgyKrqreyeeedd
BRAP	etmksnpdelKttveerksse
PHF20L1	ysakehgmpeKnpaegntvfv
ZC3H14	pslppskqanKnlilkaisea
KIF1B	fnsretskesKciiqmqgnst
MEF2A	anslgkvmptKsppppgggnl
MEF2A	dlrvvippssKgmmpplseee

Table S5

MEF2A	sintnqnisiKsepispprdr
TPD52L2	nlpssagsgdKplsdpapf--
DDX42	fggfaisagkKeepklpqqsh
DDX42	aisagkkeepKlpqqshsafg
PPHLN1	aeaaskwaaeKleksdesnlp
PPHLN1	tekelaeaasKwaaekleksd
NCOR1	rqdiltqesrKtpevvqstrp
NCOR1	egsisqgtpiKfdnnsgqsai
LSM14A	irrdgpmkfeKdfdfesanaq
KIAA1143	dgriiyrkpvKhpsdekysgl
SERBP1	errfekpleeKgeggefsvdr
SMC4	nkitkfieenKekftqldled
SMC4	itkfieenkeKftqldledvq
WDHD1	nsqtnkteevKeenlknvlse
WDHD1	qkpldfstnqKlsafafkqe-
WAPAL	sscnklitsdKvenfheehek
WAPAL	epnqkddgvfKapappskvik
TOP1	avqrleeqlmKlevqatdree
RANBP1	naenaqkfktKfeecrkeiee
RANBP1	eerekkagsgKndhaekvaek
RANBP1	kndhaekvaeKlealsvkeet
HSP90AB1	yidqeelnktKpiwtrnpddi
SAPS2	pgagappapgKkeappvegds
AGFG2	-----mvmaaKkgpgpgggvs
AGFG2	tpvqgsipegKplrtllgdpa
VIM	aeleqlkgqgKsrlgdlyeee
PSMA3	ltngrheivpKdireeaekya
PSMA3	ltngrheivpKdireeaekya
PSMA3	vpkdireeaeKyakeslkeed
PSMA3	ltngrheivpKdireeaekya
PSMA3	direeaekyaKeslkeedesd
RPS3A	gkatgdetgaKveradgyepp
PAK2	kdplsanhslKplpsvpeekk
BCOR	aarlsngkypKapeggegaqp
PER1	trssqsshtsKyfgsidssea
BRD4	pqplvvvkeeKihspiirsep
BRD4	pqplvvvkeeKihspiirsep
SYNRG	psllmplpgtKalpsmdkyav
SYNRG	yavfkgiaadKssentvppgd
SYNRG	pltsnvgstvKggqnstaast
TLE4	ndhqrdrdsiKsssvspsasf
TLE4	seskkqkteeKeiaarydsdg
TLE4	ngldktrllkKdapispasia
FYB	--------maKyntggnpted
FYB	lhsvnqdhdlKplgpksgptp
ANKRD12	dkiasysktpKiersdvskem
PARP1	gqdgigskaeKtlgdfaaeya
PARP1	eginksekrmKltlkggaavd
CHD4	vvveppegeeKvekaevkert
CHD4	tepkgaadveKveeksaidlt
MORC2	krgrfvvkeeKkdsnelsdsa
MORC2	rgrfvvkeekKdsnelsdsag

Table S5

NAA11	delrrqmdlkKggyvvlgsre
CDK13	aktkpplqvtKvennlivdka
GPATCH8	kdppqgyfgpKlppslgnkpv
TXLNA	tlntlstpeeKlaalckkyae
TXLNA	tlntlstpeeKlaalckkyae
CSDE1	sdhrflgtveKeatfsnpktt
CSDE1	vekeatfsnpKttspnkgkek
KDM3A	nsdspnncsgKkvepsalacr
KDM3A	salacrsqnlKessvkvdnes
DTNBP1	svqqdftsglKtlsdksreak
STMN1	--massdiqvKelekrasgqa
STMN1	rrksheaevlKqlaekrehek
STMN1	eaevlkqlaeKrehekevlqk
STMN1	kqlaekreheKevlqkaieen
STMN1	kaieennnfsKmaeeklthkm
STMN1	nnnfskmaeeKlthkmeanke
STMN1	skmaeeklthKmeankenrea
STMN1	maaklerlreKdkhieevrkn
STMN1	maaklerlreKdkhieevrkn
ARCN1	kgkevdnfvdKlksegetims
SMARCA2	essqmsdlpvKvthtetgkvl
SMARCA2	gkkrpnrgkaKpvvsdfdsde
RAD50	aceirdqitsKeaqltsskei
RAD50	skimkldneiKaldsrkkqme
NPM1	pkgpssvediKakmqasiekg
NPM1	pkgpssvediKakmqasiekg
NPM1	pkgpssvediKakmqasiekg
NPM1	pkgpssvediKakmqasiekg
NPM1	pkgpssvediKakmqasiekg
MNS1	aakeeeenfrKtmlakfaedd
RPL3	mgplkkdriaKeega------
RPL3	mgplkkdriaKeega------
MDC1	iqgrgrqtvdKvmgipketae
MAP7D2	agttdageaaKilaekrrqar
KRT8	daeqrgelaiKdanaklsele
KRT8	gelaikdanaKlseleaalqr
KRT8	vvkkietrdgKlvsessdvlp
LOC643790	------mpkrKvssaegaake
LOC643790	------mpkrKvssaegaake
NCL	akagknqgdpKkmapppkeve
NCL	kagknqgdpkKmapppkevee
NCL	kagknqgdpkKmapppkevee
NCL	rsqpsktlfvKglsedtteet
NCL	rsqpsktlfvKglsedtteet
HCFC1	qetskdssgtKpankrpmssp
FAM22A	gtarldssssKfaagqgaerd
EEF1D	diarareniqKslagssgpga
EEF1D	klearlnvleKsspghratap
ATAD3A	qhqaraqyqdKlarqryedql
TACC2	yqalkvhaeeKldranaeiaq
TACC2	eqaahqaslrKeqlrvdaler
ARID1A	spemmglgdvKltpatkmnnk

Table S5

ARID1A	spemmglgdvKltpatkmnnk
ARID1A	kssssttneKitklyelgge
ARID1A	sstttnekitKlyelggeper
ARID1A	pflhsgmkmqKagppvpashi
ARID1A	egpppdgppeKritatmddml
ARID1A	ksseaikessKfpfgispaqs
TKT	-----mesyhKpdqqklqalk
TKT	mesyhkpdqqKlqalkdtanr
HNRNPR	tyrqrekqgsKvqestkgpde
HNRNPR	qestkgpdeaKikallertgy
XRCC6	pdynpegkvtKrkhdnegsgs
XRCC6	pkveyseeelKthiskgtlgk
XRCC6	pkveyseeelKthiskgtlgk
XRCC6	vyppdynpegKvtkrkhdneg
XRCC6	pdynpegkvtKrkhdnegsgs
XRCC6	rkhdnegsgsKrpkveyseee
XRCC6	pkveyseeelKthiskgtlgk
XRCC6	pkveyseeelKthiskgtlgk
XRCC6	rkhdnegsgsKrpkveyseee
XRCC6	dnegsgskrpKveyseeelkt
XRCC6	pkveyseeelKthiskgtlgk
AGAP6	kpsekstreeKerwirskyee
SAFB	elhgkmisveKaknepvgkkt
SAFB	hgkmisvekaKnepvgkktsd
SETX	vpqpvpliaqKpvgemknscn
SKIV2L2	klqsestnngKnkrdvdfegt
SKIV2L2	egtdepifgkKprieesited
SKIV2L2	klqsestnngKnkrdvdfegt
SKIV2L2	fegtdepifgKkprieesite
SKIV2L2	egtdepifgkKprieesited
SKIV2L2	klqsestnngKnkrdvdfegt
SKIV2L2	fegtdepifgKkprieesite
SKIV2L2	egtdepifgkKprieesited
CCDC75	epiplniktgKsgigheaslk
CCDC75	gigheaslkrKaeeklesyrk
SLC4A8	kkakeeeeaeKmleiggdkfp
SLC4A8	aekmleiggdKfplesrklls
KIAA0947	tlskemnkelKaseigekyrk
KIAA0947	kelkaseigeKyrkqpceeet
KIAA0947	kaseigekyrKqpceeetlgt
INTS1	nesktastllKpapsglpser
TPR	nahiklsnlyKsaaddseaks
TPR	yksaaddseaKsneltravee
TPR	kllkeageanKaiqdhlleve
TPR	nahiklsnlyKsaaddseaks
TPR	yksaaddseaKsneltravee
TPR	kllkeageanKaiqdhlleve
TPR	eveqskdqmeKemlekigrle
TPR	enandllsatKrkgailseee
TPR	keveakapilKrqreeyeraq
TPR	eiqrlqedtdKankqssvler
ING4	tdlarfeadlKekqiessdyd

Table S5

E2F3	lrspdspktpKspsektrydt
E2F3	spktpkspseKtrydtslgll
E2F3	spktpkspseKtrydtslgll
E2F3	lrspdspktpKspsektrydt
NCOA3	aenqrgplesKghkkllqllt
NCOA3	aenqrgplesKghkkllqllt
WWC1	slvsgsssssKydpeilkaei
CAST	rhllddngqdKpvkpptkkse
ING5	larfeadlkdKmegsdfessg
ZEB2	tqlrnklengKplsmseqtgl
ZEB2	tnsaitqlrnKlengkplsms
ZEB2	tqlrnklengKplsmseqtgl
HSPD1	taevvvteipKeekdpgmgam
HSPD1	vvvteipkeeKdpgmgamggm
ZFR	tpkinfvggnKlqstgnkaed
LRRFIP1	vdtqnepldmKepdeeksdqq
LRRFIP1	tyqntdlseiKeeeqvkstdr
LRRFIP1	vdtqnepldmKepdeeksdqq
LRRFIP1	tyqntdlseiKeeeqvkstdr
RPL6	egeifdtekeKyeiteqrkid
MYH10	seqleqakrfKanleknkqgl
MYH10	krfkanleknKqgletdnkel
ARPP19	emedkvtspeKaeeaklkary
ARPP19	vtspekaeeaKlkaryphlgq
ARPP19	spekaeeaklKaryphlgqkp
ARPP19	spekaeeaklKaryphlgqkp
ARPP19	iptpqdlpqrKpslvasklag
AIMP1	aklkkeieelKqeliqaeiqn
POLQ	arkrasldinKekpgasqneg
BOD1L	qtsdssegktKsvrhayvhkp
BOD1L	qghpsavcaeKeekhgkecpe
BOD1L	psavcaekeeKhgkecpeigp
BOD1L	eigpfagrgqKestlhlinae
SUMO3	------mseeKpkegvktend
SUMO3	----mseekpKegvktendhi
SUMO3	mseekpkegvKtendhinlkv
FNBP1	ekmdadinvtKadvekarqqa
TMSB4X	lrfssatmsdKpdmaeiekfd
TMSB4X	etqeknplpsKetieqekqag
TMSB4X	etqeknplpsKetieqekqag
TMSB4X	etqeknplpsKetieqekqag
TMSB4X	etqeknplpsKetieqekqag
TMSB4X	sklkktetqeKnplpsketie
TMSB4X	etqeknplpsKetieqekqag
TMSB4X	lpsketieqeKqages-----
KHD1-EIF4E	vtpnslstsyKtvslplsspn
TCOF1	gnsmphpatgKtvanllsgks
TCOF1	anllsgksprKsaepsanttl
TCOF1	eapagtrsqvKasekilqvra
TCOF1	gkagavasqtKagkpeedses
TCOF1	qkagpvavqvKaekpmdnses
TCOF1	gqgtapvlpgKtgptvtqvka

Table S5

TCOF1	gktgptvtqvKaekqedsess
TMSL1	sklkktetqeKnplpsketie
TMSL1	etqeknplpsKetieqekqag
TMSL1	lpsketieqeKqages-----
PRAM1	meshqdfrsiKakfqasqpep
PRAM1	asqpepsdlpKkppkpefgkl
PRAM1	sqpepsdlpkKppkpefgklk
PRAM1	meshqdfrsiKakfqasqpep
PRAM1	ppkpefgklkKfsqpelsehp
MED1	vdsskktsesKnvgstgvaki
MED1	esesgssiaeKsyqnspssdd
MED1	esesgssiaeKsyqnspssdd
CTNNBL1	miltfekrsyKnqelrikfpd
CTNNBL1	rsyknqelriKfpdnpekfme
CGN	etghwqsmfqKnkedlratkq
CGN	ghwqsmfqknKedlratkqel
CGN	qknkedlratKqellqlrmek
ATRX	glsdiaekflKkdqsdetsed
ATRX	lsdiaekflkKdqsdetsedd
BAHCC1	flvgkelgreKagkaaegker
DIAPH1	vqnfpaatdeKdkfvekmtsf
DIAPH1	vqnfpaatdeKdkfvekmtsf
PHF20L1	ysakehgmpeKnpaegntvfv
PHF20L1	rsknslqysaKehgmpeknpa
PHF20L1	ysakehgmpeKnpaegntvfv
PDS5A	vtpvknidpvKnkeinsdqat
PDS5A	pvknidpvknKeinsdqatqg
CENPF	eekmesqgimKnkeiqeleql
CENPF	mdnlkyvnqlKkeneraqgkm
AHNAK2	mpsfgvsapgKsieasvdvsa
LSM12	ekhfrdvesqKilqrsqaqqp
DCTN1	qleqvqewksKmqeqqadlqr
POLQ	arkrasldinKekpgasqneg
MAD1L1	leeraeqirsKshliqverek
MAD1L1	kshliqvereKmqmelshkra
INTS1	nesktastllKpapsglpser
PHF15	tksgwprqneKkpsevfrtdl
PHF15	ksgwprqnekKpsevfrtdli
FAM22E	gtarldssssKfaagqgaerd
POM121	pssnapdpcaKetvlsalkek
TPM1	aqerlatalqKleeaekaade
TPM1	kaadesergmKviesraqkde
MLL3	dvsyqggksiKlssetessfs
MLL3	iaagtsdhftKpspradvfqr
MLL3	vepkkkeqenKtlvlsdkhsp
MLL3	lvlsdkhspqKkstvtnevkt
MLL3	qenktlvlsdKhspqkkstvt
MLL3	qkkstvtnevKtevlspnskv
MLL3	qkkstvtnevKtevlspnskv
MLL3	vktevlspnsKveskcetekn
MLL3	vdklsmetpaKteeiklekae
MLL3	metpakteeiKlekaetescp

Table S5

BUB1B	adeastaelsKptvqpwiapp
RSBN1L	atvsekepfgKlqlssrdppg
ZYX	ppasspapapKfspvtpkftp
ZYX	vtpkftpvasKfspgapggsg
TUBA1B	iqpdgqmpsdKtigggddsfn
TUT1	cekmaqfdakKfaetqpkkdt
RUNX1	tppstalspgKmsealplgap
RUNX1	apdagaalagKlrsgdrsmve
RUNX1	apdagaalagKlrsgdrsmve
COG6	kttklqsesqKleiraqvada
MAD1L1	leeraeqirsKshliqverek
MAD1L1	kshliqvereKmqmelshkra
SNRNP40	---mieqqkrKgpelplvpvk
USO1	qlnsqsveitKlqtekqellq
USO1	sveitklqteKqellqkteaf
USO1	lqtekqellqKteafaksvev
USO1	ellqkteafaKsvevqgetet
TEF	lsgsfplvlkKlmenpprear
TKT	-----mesyhKpdqqklqalk
TKT	mesyhkpdqqKlqalkdtanr
